# Supplementary material for: Repressing PTBP1 fails to convert reactive astrocytes to dopaminergic neurons in a 6-hydroxydopamine mouse model of Parkinson’s disease
Source: eLife. 2022 May 10;11:e75636. doi: 10.7554/eLife.75636 (PMC9208759; doi:10.7554/eLife.75636)
Supplement: Figure 1—source data 1. [file elife-75636-fig1-data1.zip › Fig1 source data 1 for Fig1 B&C/description of source data for Fig1B.docx]

Brain slices co-stained with PTBP1 (red) and GFP (green) at indicated time points after AAV-sh*Ptbp1* or AAV-shscramble delivery in the substantia nigra
