# Supplementary figures and images for: Repressing PTBP1 fails to convert reactive astrocytes to dopaminergic neurons in a 6-hydroxydopamine mouse model of Parkinson’s disease (part 1 of 4)
Source: eLife. 2022 May 10;11:e75636. doi: 10.7554/eLife.75636 (PMC9208759; doi:10.7554/eLife.75636)

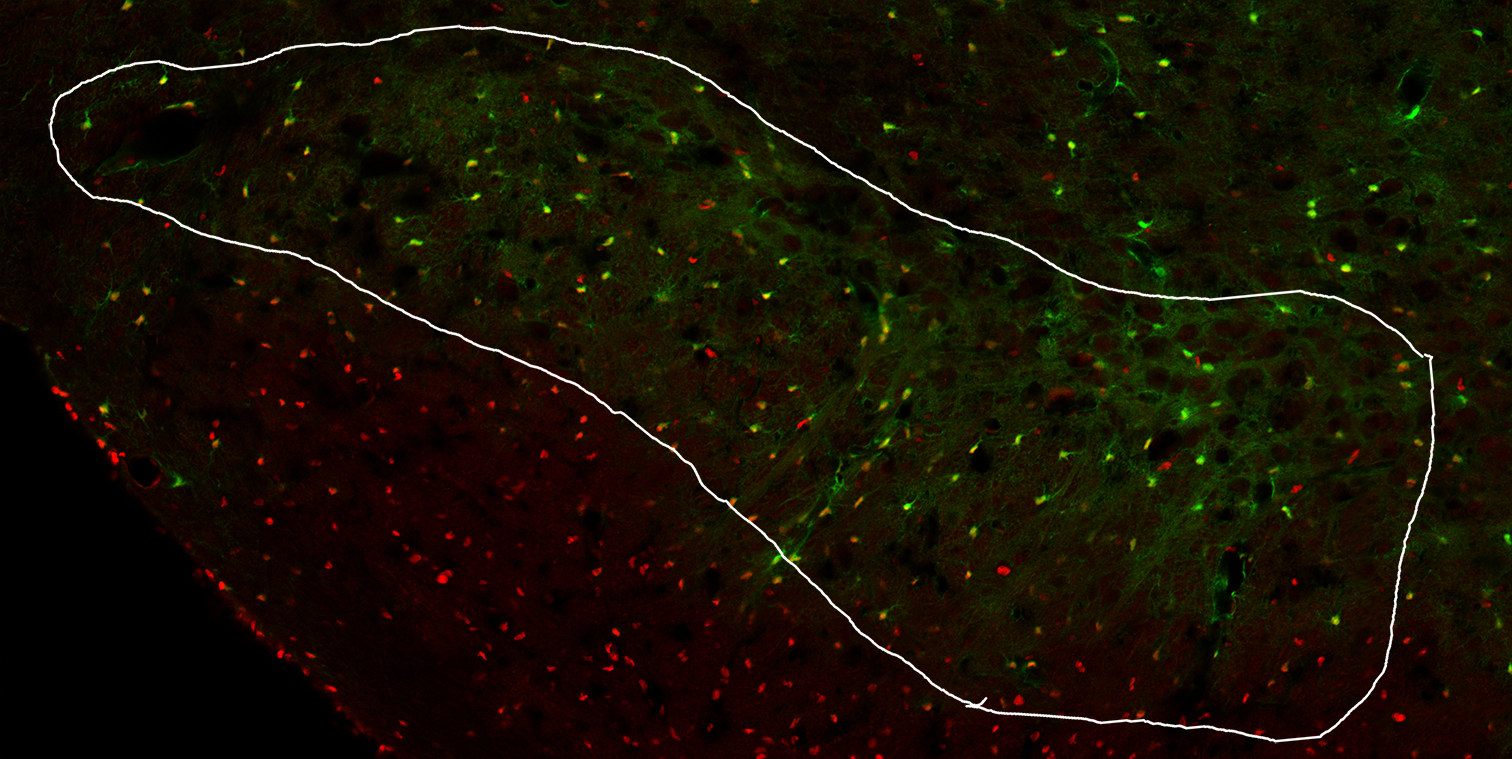

Supplement: Figure 1—source data 1. [file elife-75636-fig1-data1.zip › Fig1 source data 1 for Fig1 B&C/SN shscramble 1M MZ1.jpg]

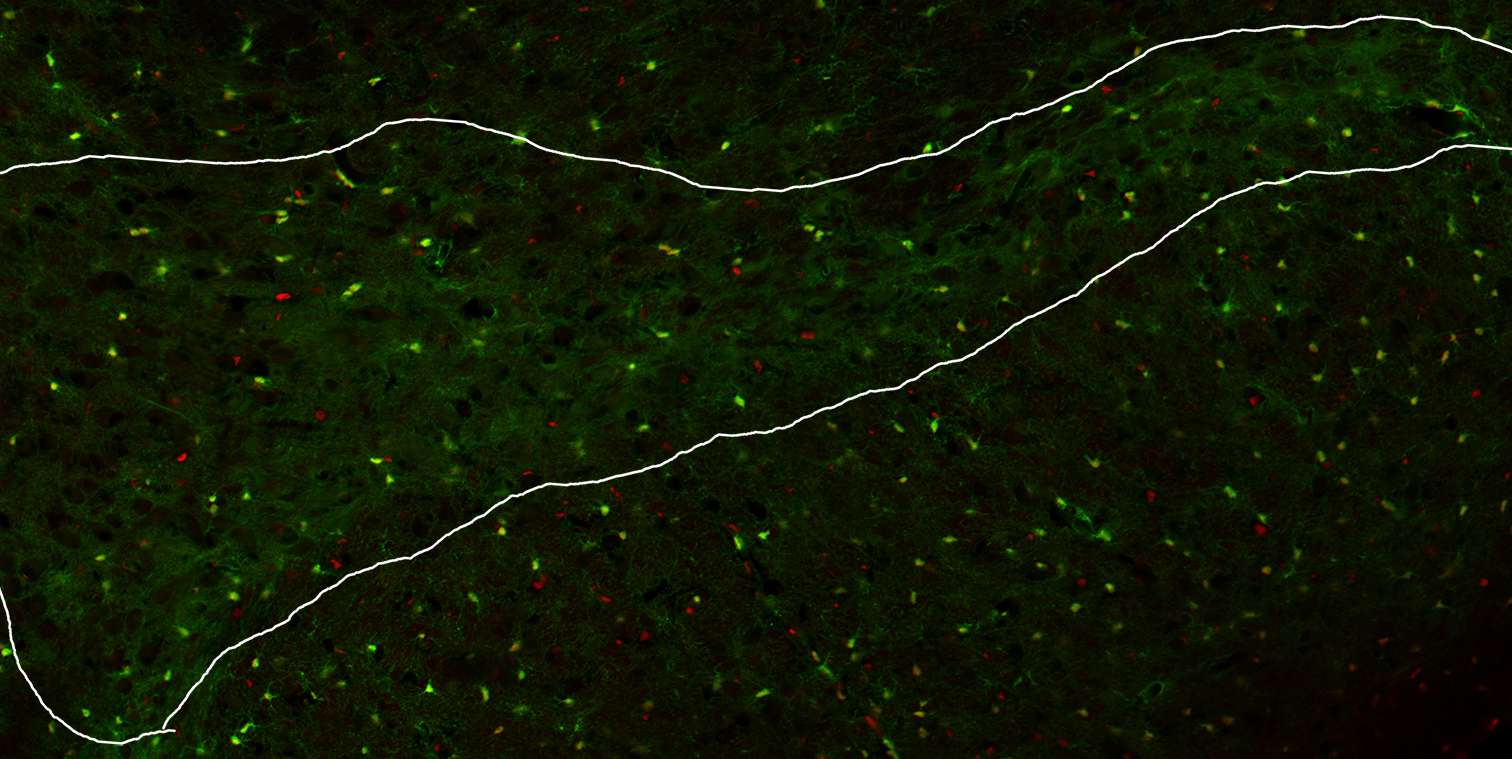

Supplement: Figure 1—source data 1. [file elife-75636-fig1-data1.zip › Fig1 source data 1 for Fig1 B&C/SN shscramble 1M MZ2.jpg]

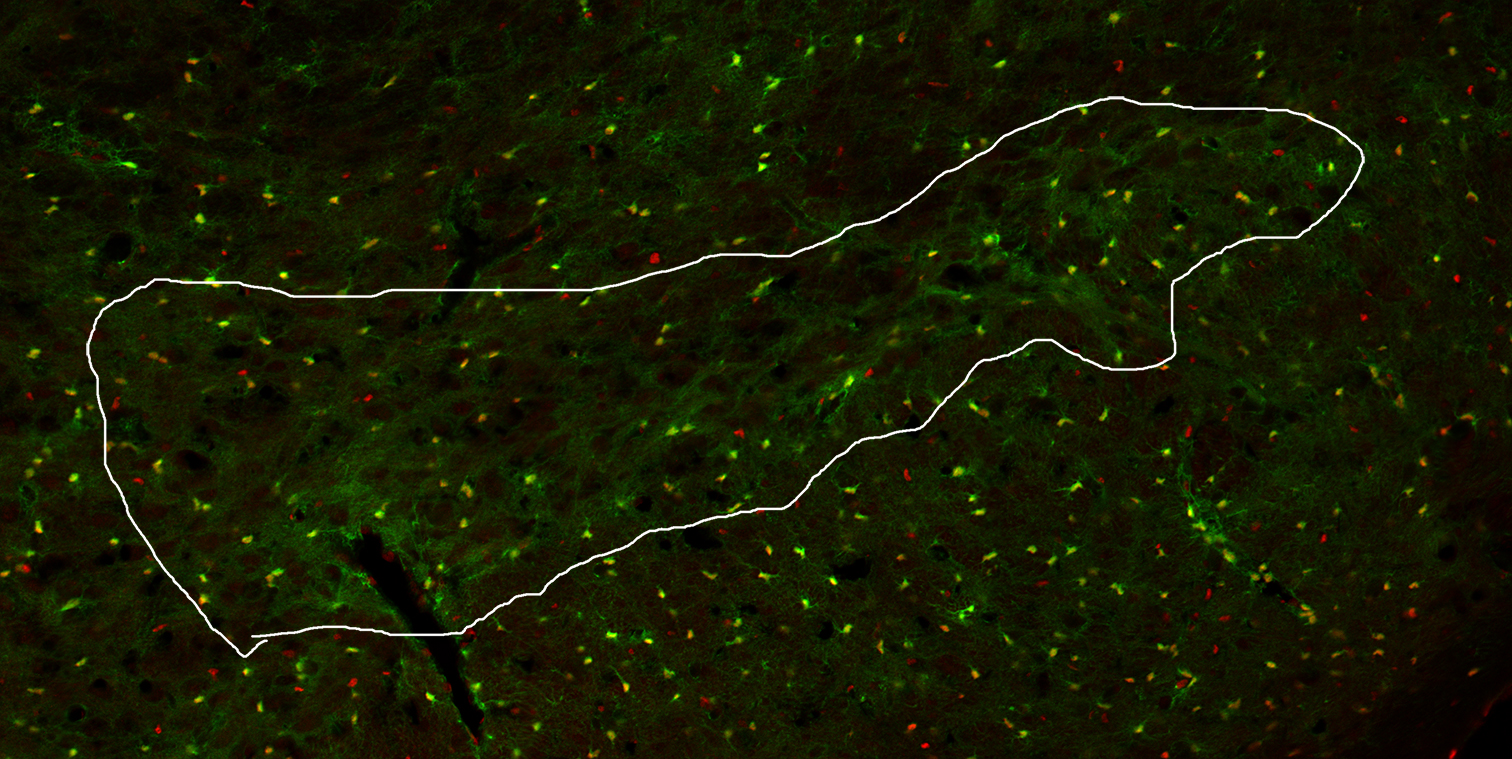

Supplement: Figure 1—source data 1. [file elife-75636-fig1-data1.zip › Fig1 source data 1 for Fig1 B&C/SN shscramble 1M MZ3.jpg]

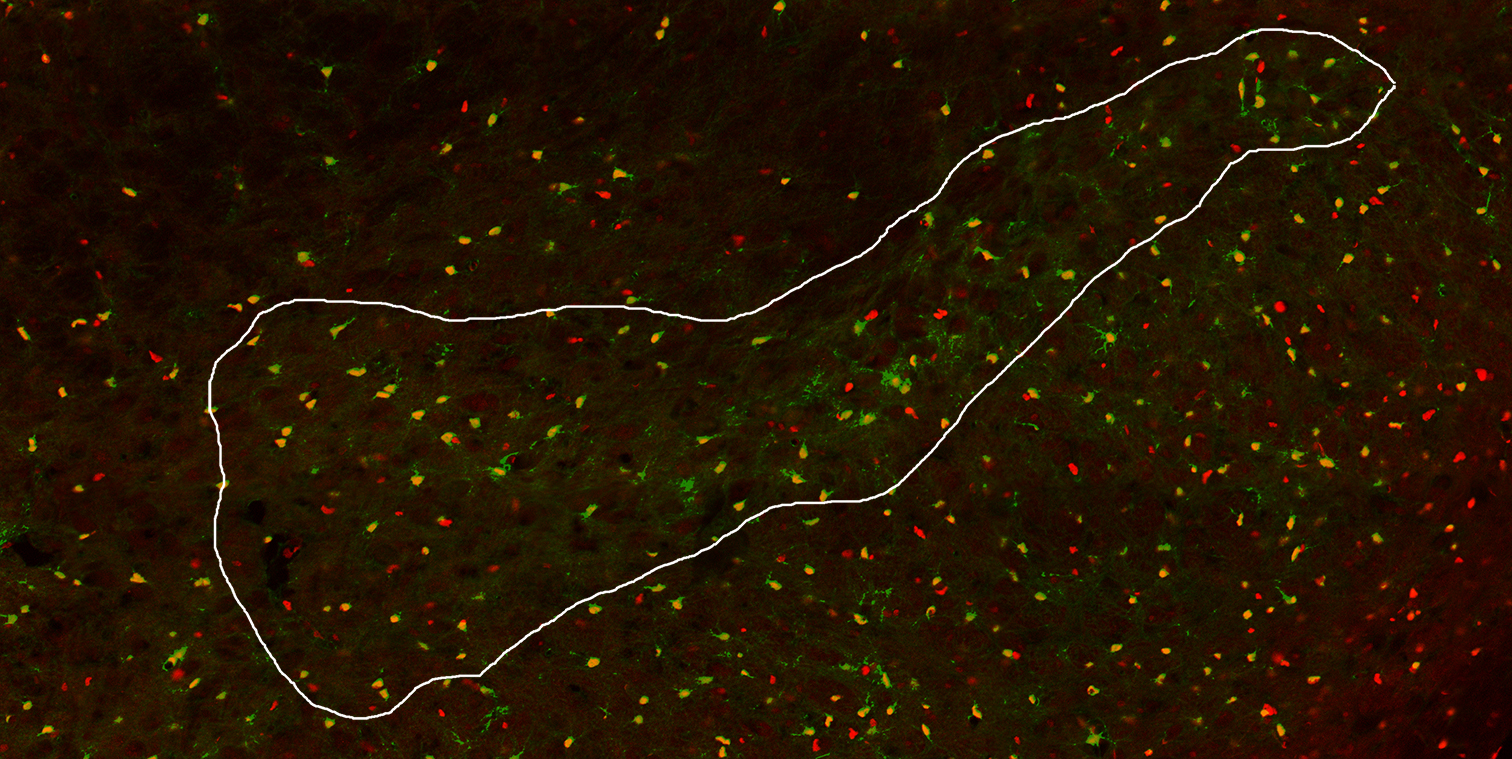

Supplement: Figure 1—source data 1. [file elife-75636-fig1-data1.zip › Fig1 source data 1 for Fig1 B&C/SN shscramble 2M MZ1.jpg]

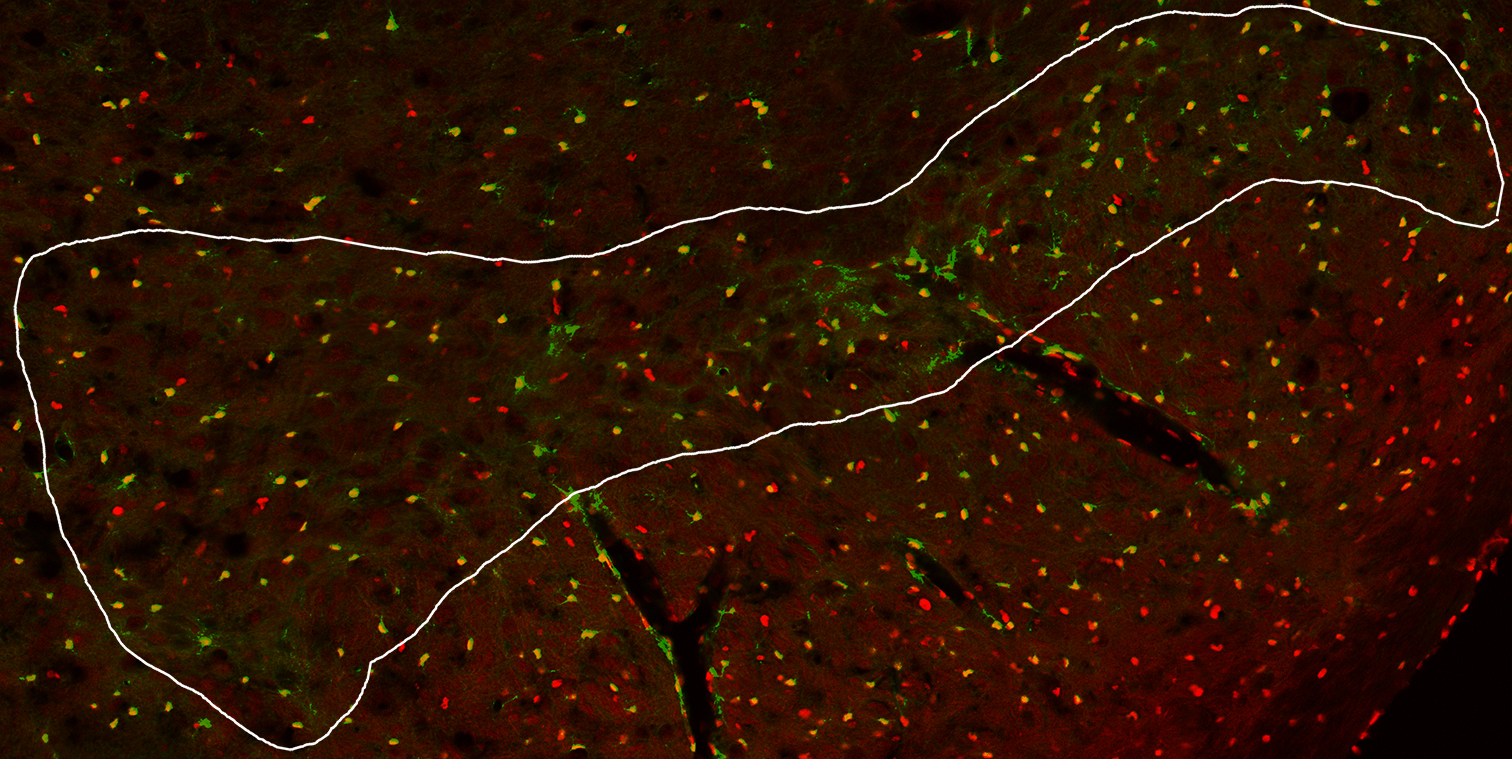

Supplement: Figure 1—source data 1. [file elife-75636-fig1-data1.zip › Fig1 source data 1 for Fig1 B&C/SN shscramble 2M MZ2.jpg]

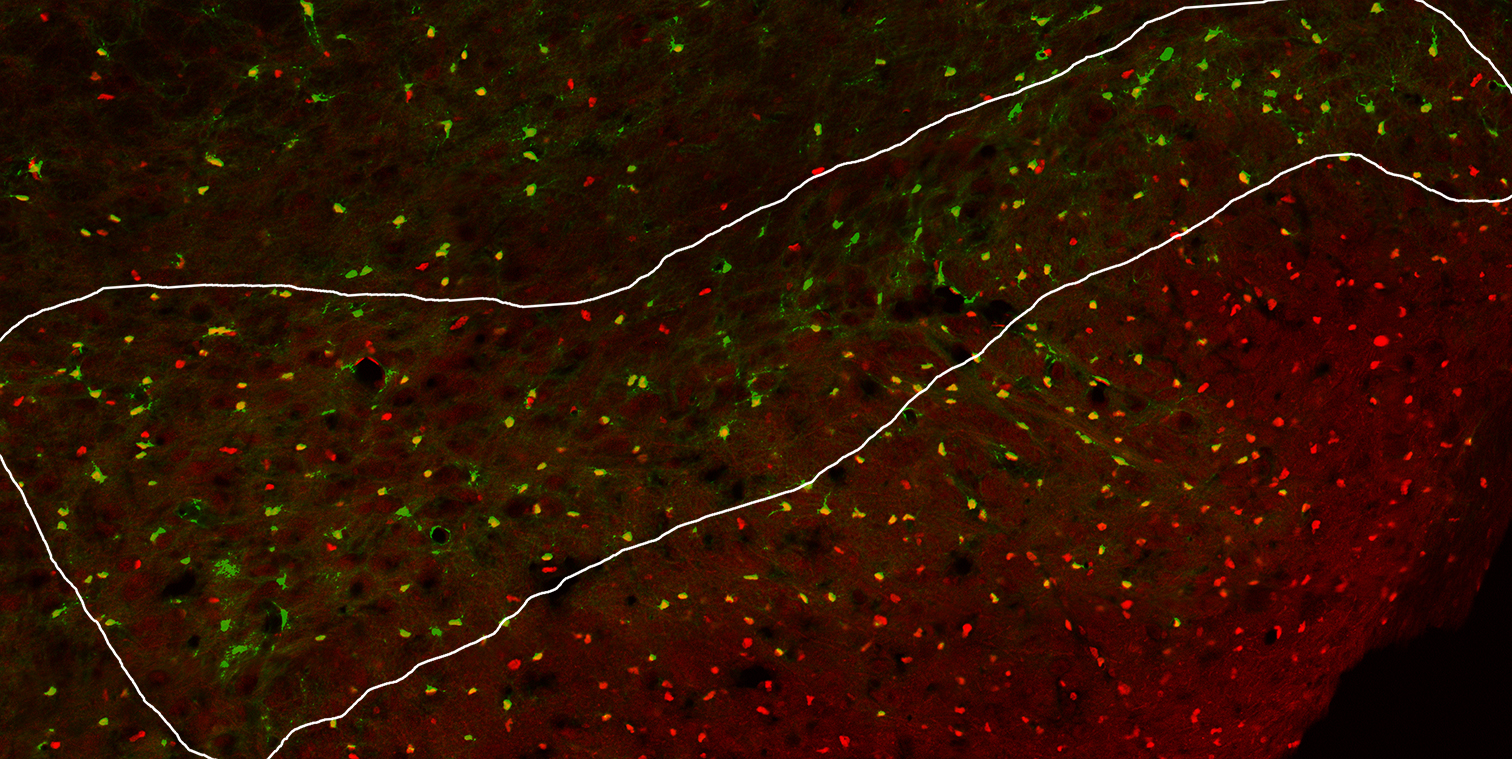

Supplement: Figure 1—source data 1. [file elife-75636-fig1-data1.zip › Fig1 source data 1 for Fig1 B&C/SN shscramble 2M MZ3.jpg]

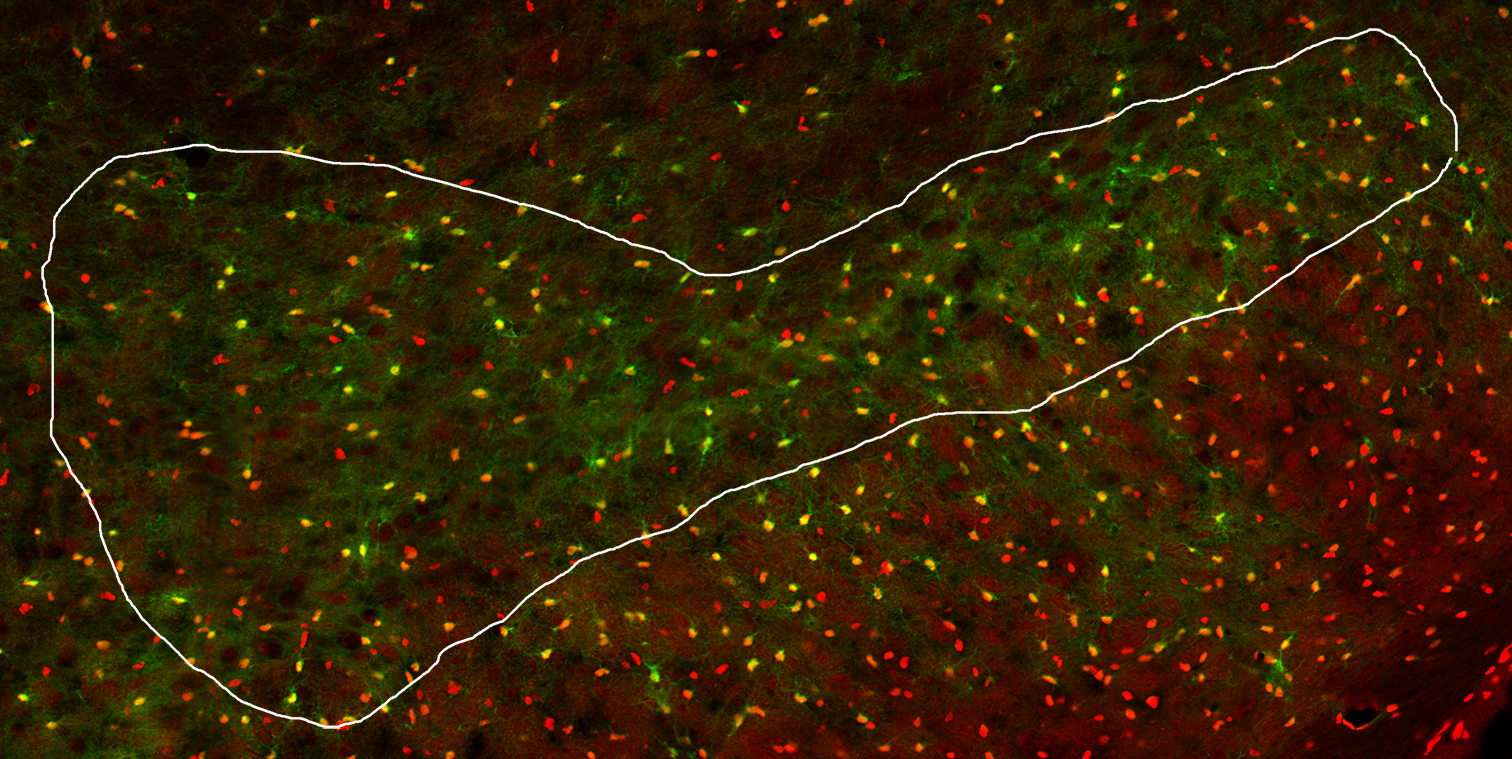

Supplement: Figure 1—source data 1. [file elife-75636-fig1-data1.zip › Fig1 source data 1 for Fig1 B&C/SN shscramble 3M MZ1.jpg]

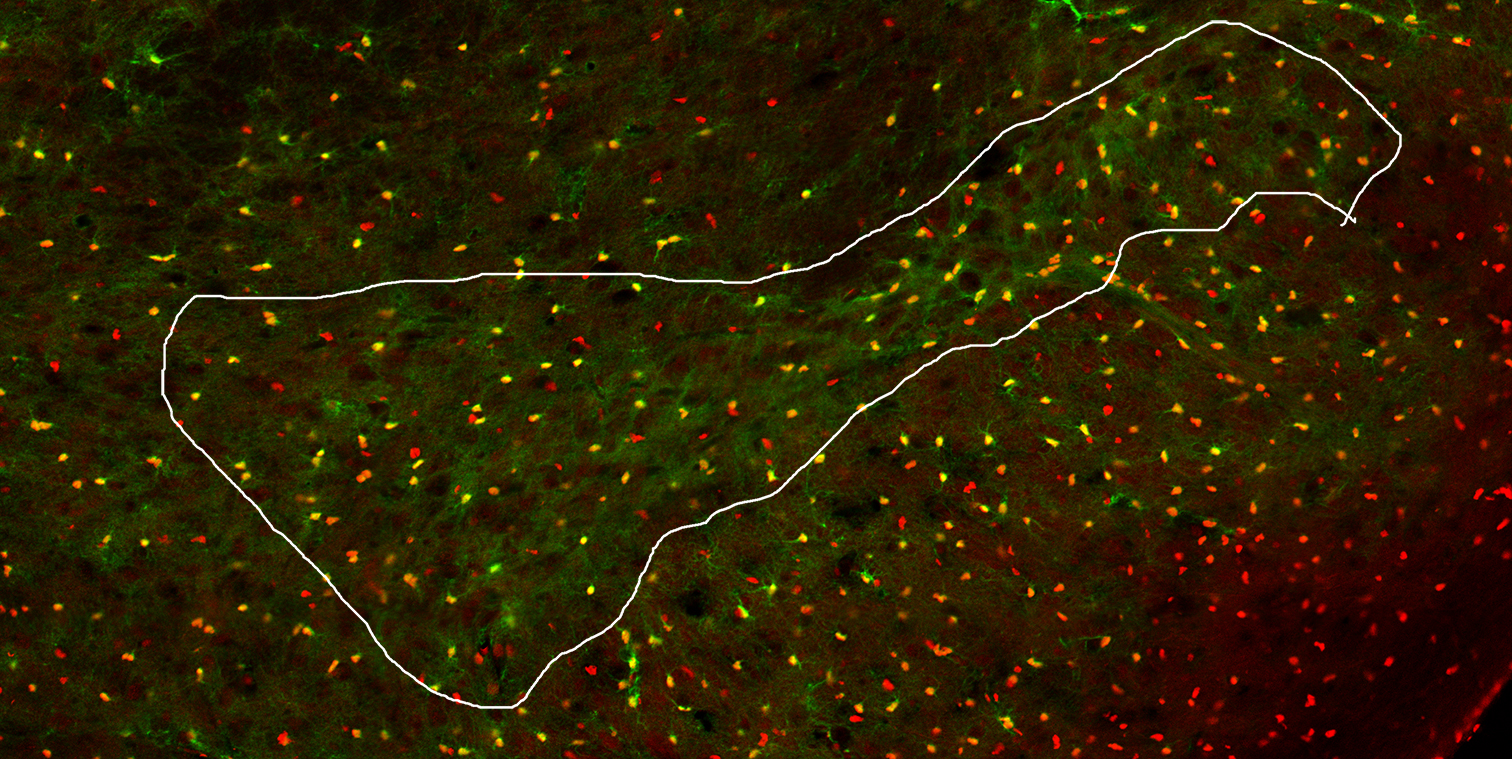

Supplement: Figure 1—source data 1. [file elife-75636-fig1-data1.zip › Fig1 source data 1 for Fig1 B&C/SN shscramble 3M MZ2.jpg]

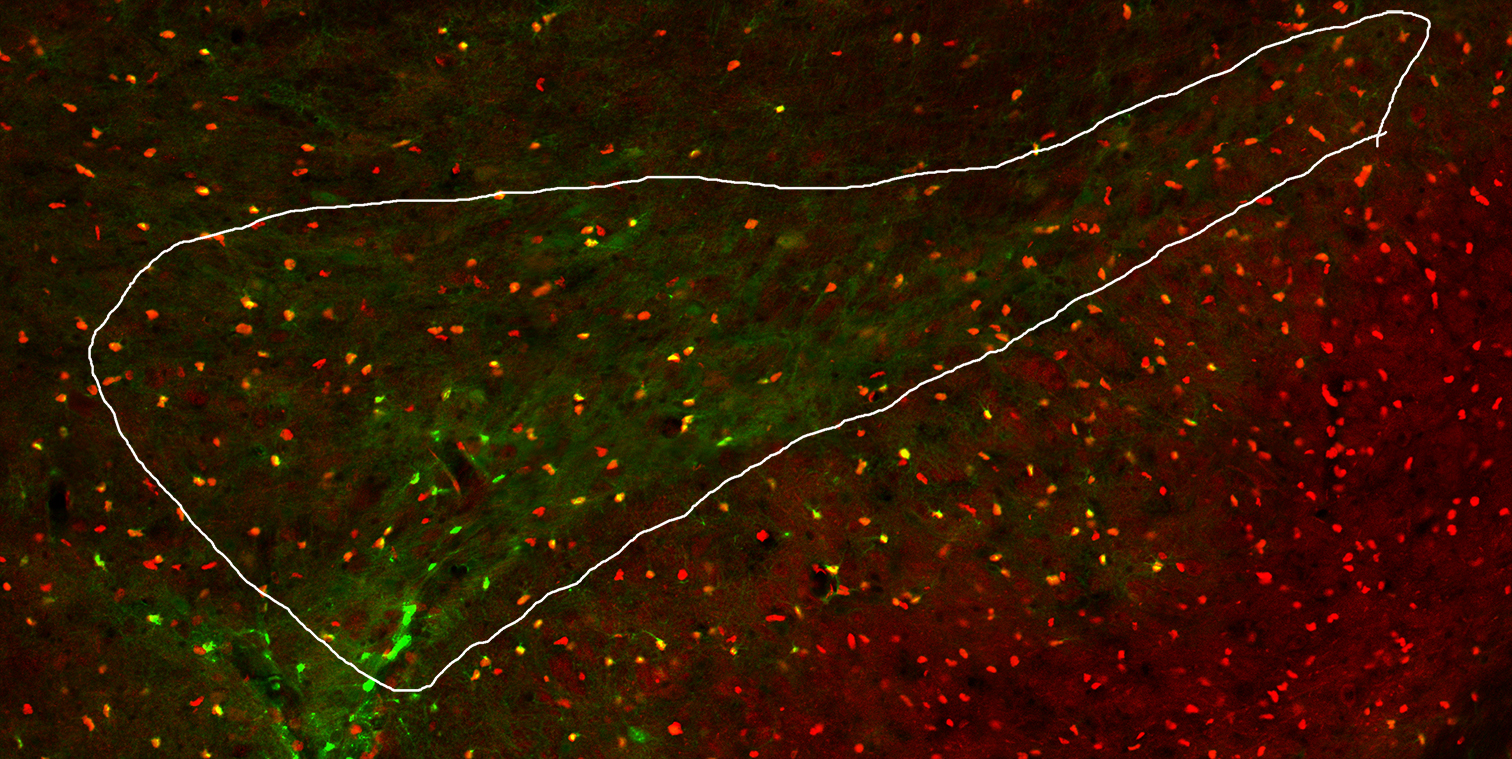

Supplement: Figure 1—source data 1. [file elife-75636-fig1-data1.zip › Fig1 source data 1 for Fig1 B&C/SN shscramble 3M MZ3.jpg]

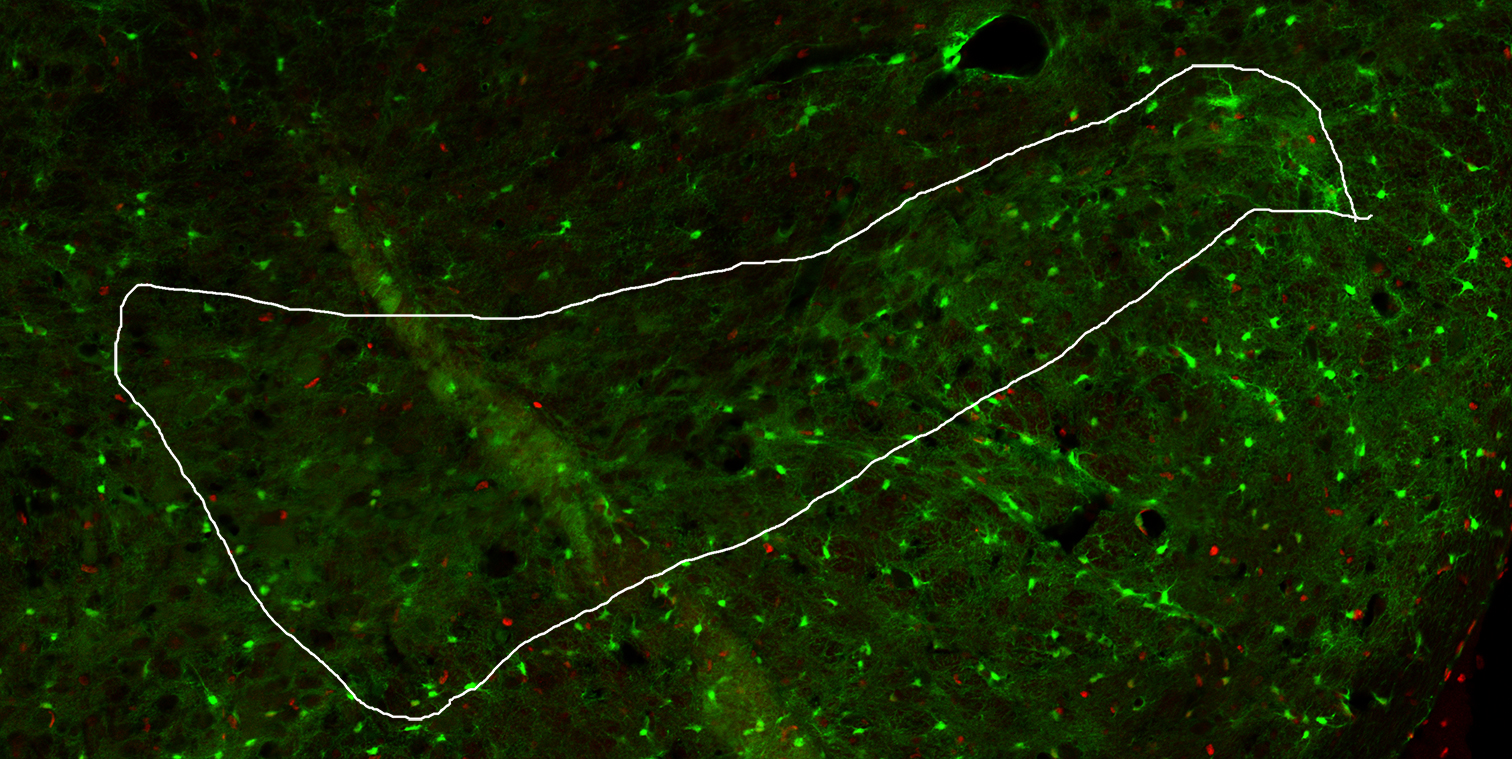

Supplement: Figure 1—source data 1. [file elife-75636-fig1-data1.zip › Fig1 source data 1 for Fig1 B&C/SN shsPTB 1M MZ1.jpg]

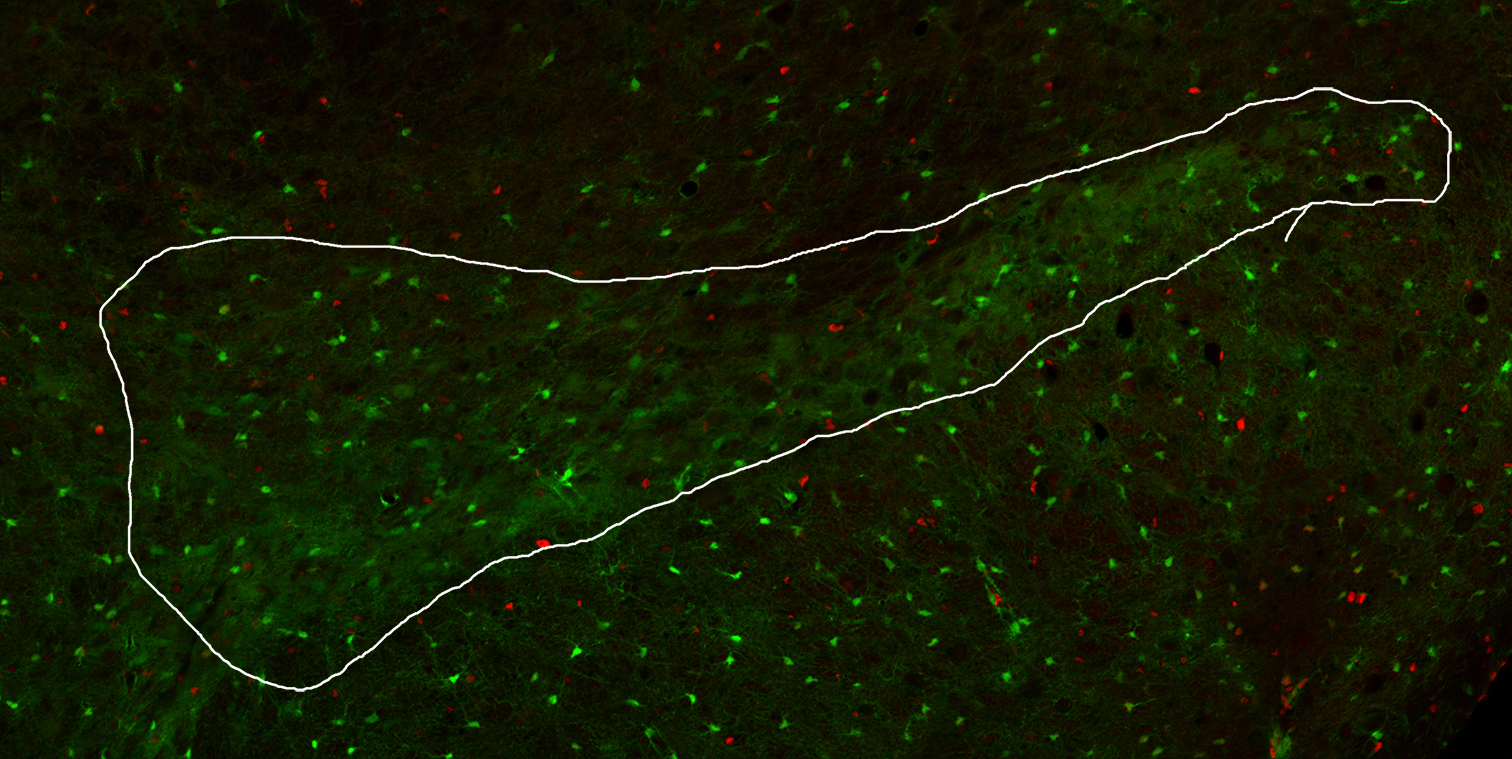

Supplement: Figure 1—source data 1. [file elife-75636-fig1-data1.zip › Fig1 source data 1 for Fig1 B&C/SN shsPTB 1M MZ2.jpg]

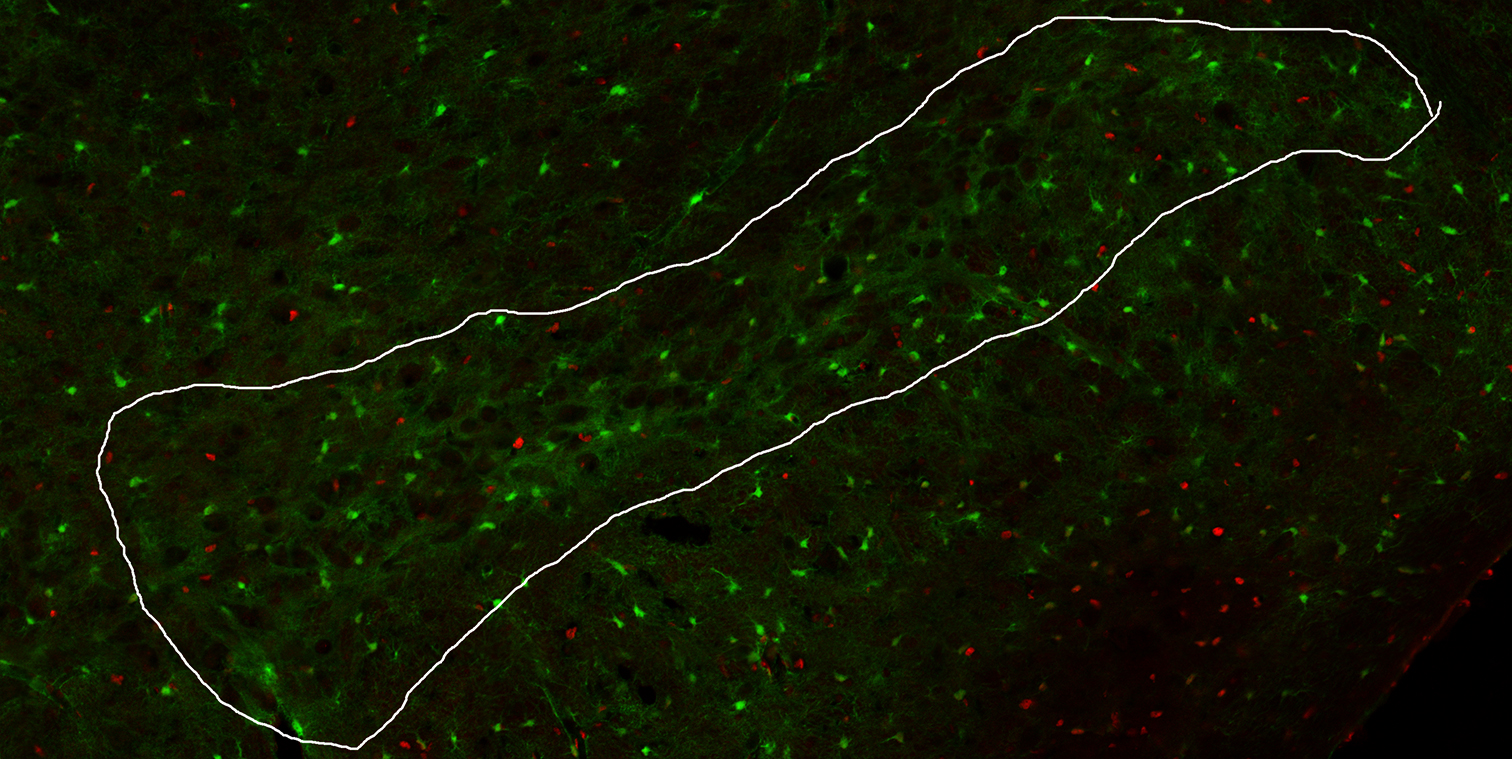

Supplement: Figure 1—source data 1. [file elife-75636-fig1-data1.zip › Fig1 source data 1 for Fig1 B&C/SN shsPTB 1M MZ3.jpg]

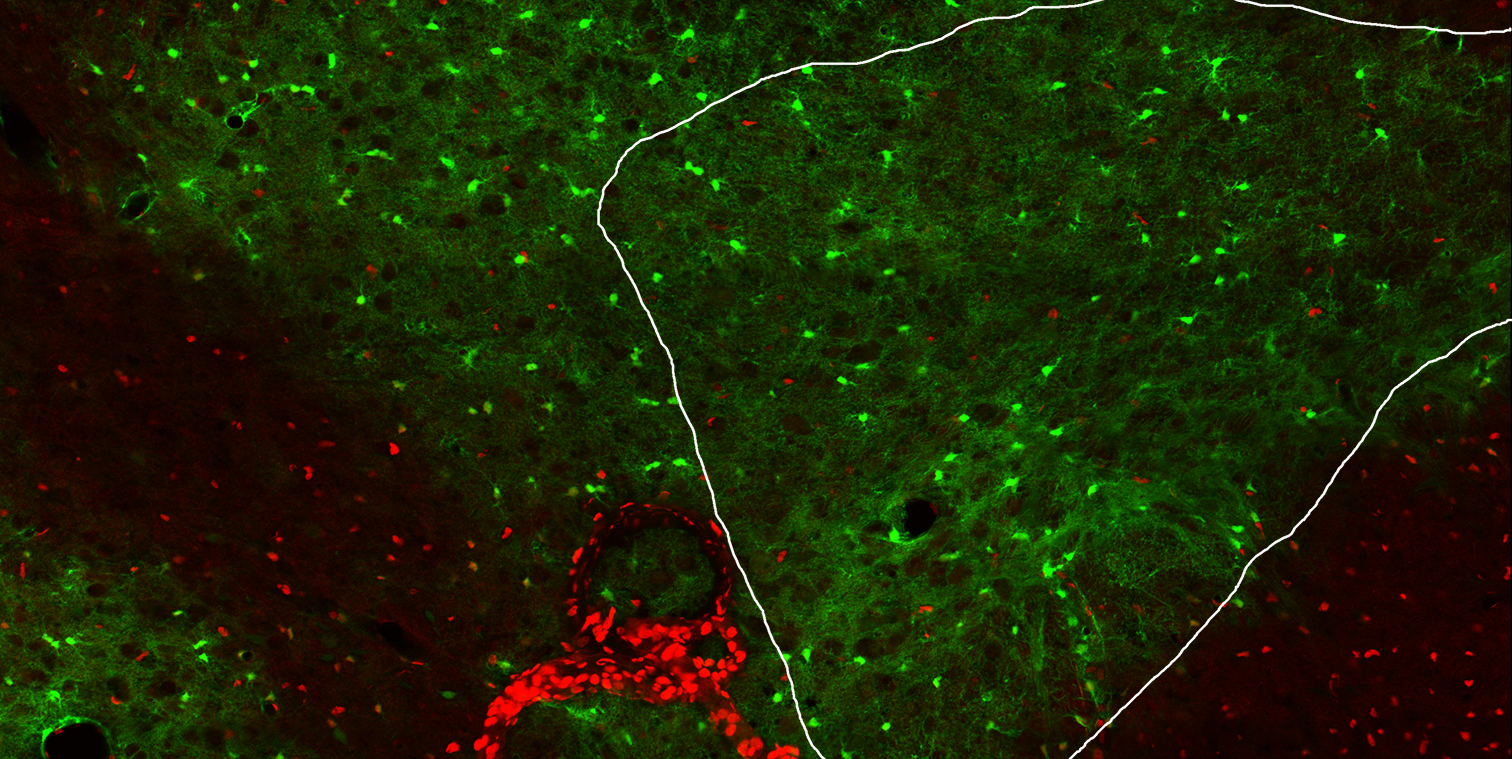

Supplement: Figure 1—source data 1. [file elife-75636-fig1-data1.zip › Fig1 source data 1 for Fig1 B&C/SN shsPTB 2M MZ1.jpg]

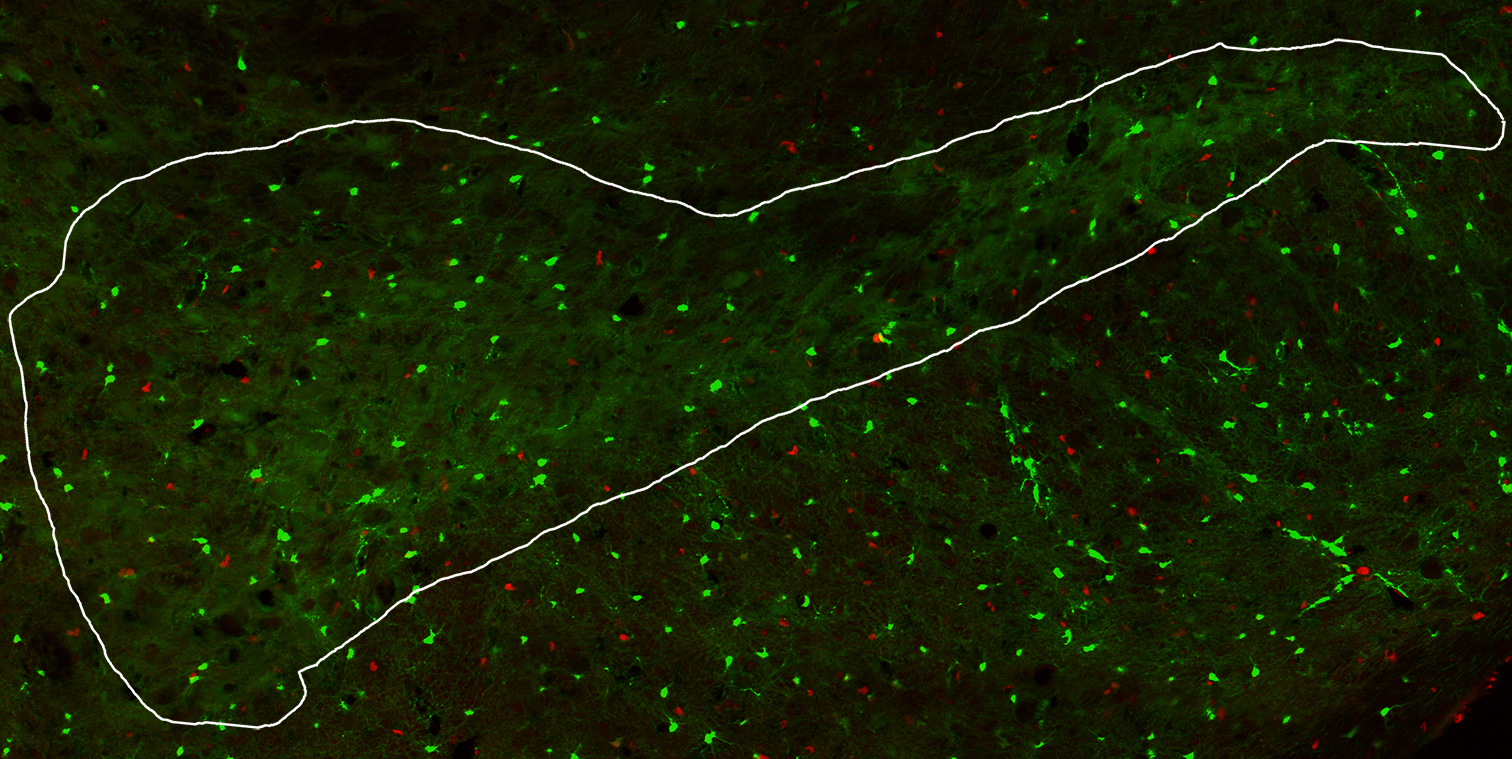

Supplement: Figure 1—source data 1. [file elife-75636-fig1-data1.zip › Fig1 source data 1 for Fig1 B&C/SN shsPTB 2M MZ2.jpg]

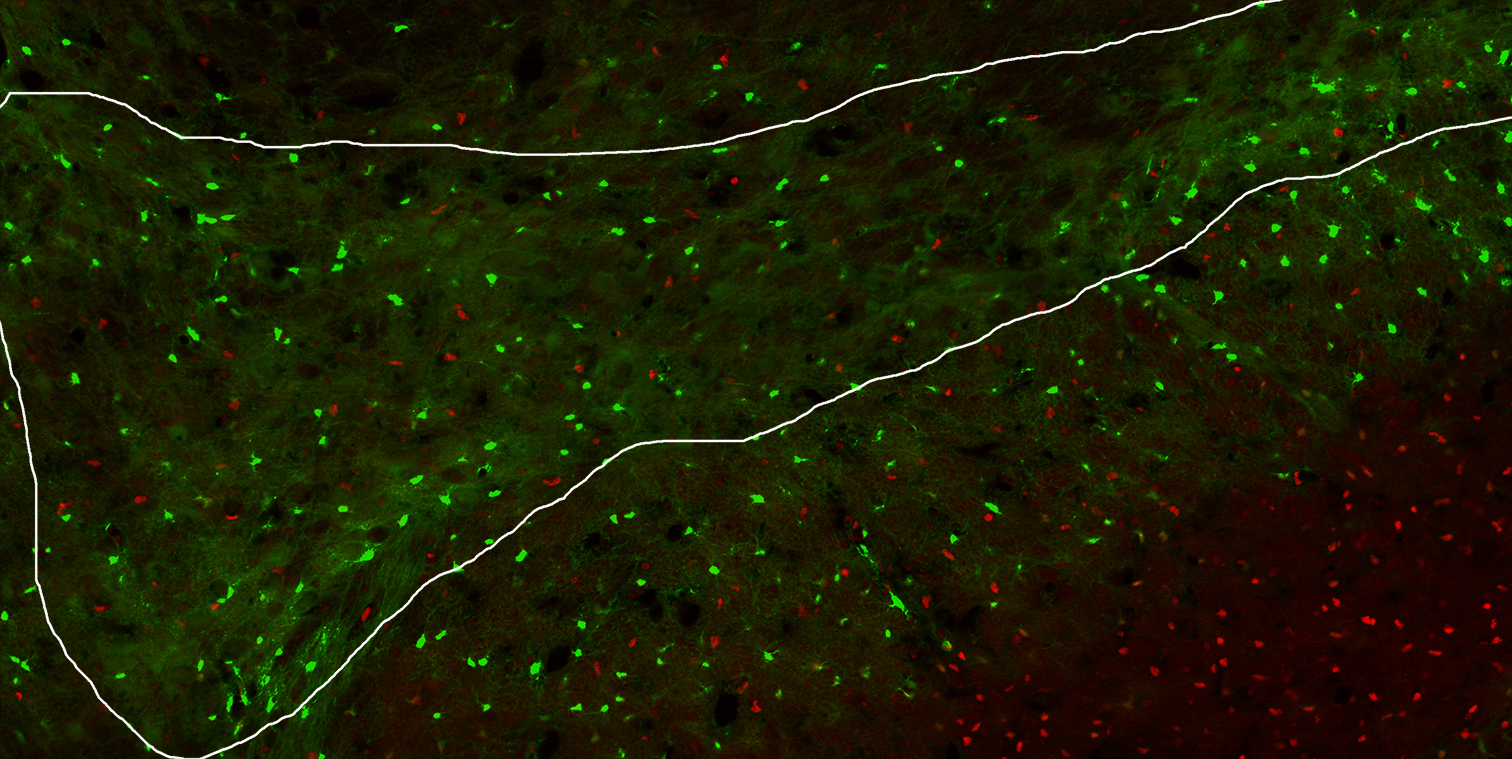

Supplement: Figure 1—source data 1. [file elife-75636-fig1-data1.zip › Fig1 source data 1 for Fig1 B&C/SN shsPTB 2M MZ3.jpg]

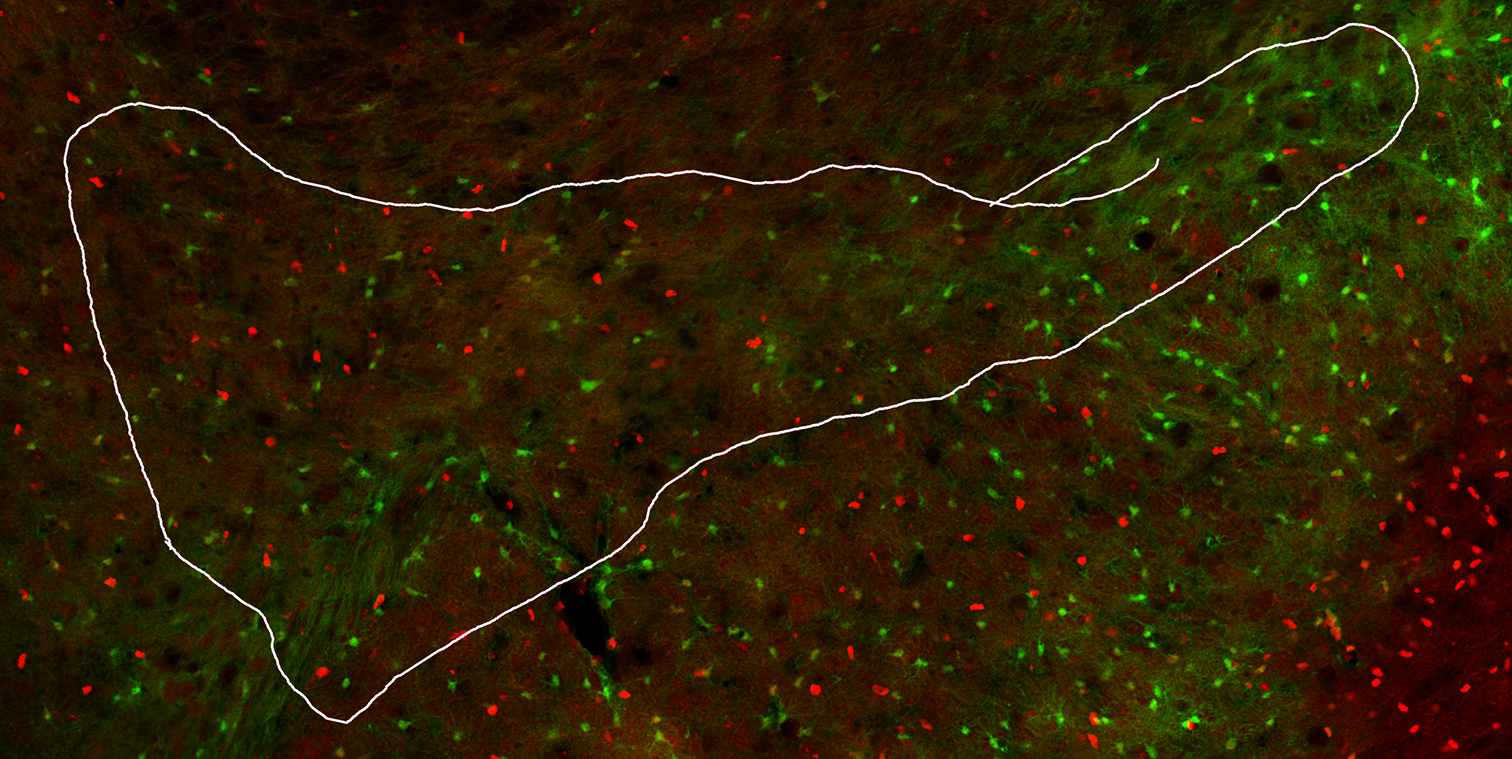

Supplement: Figure 1—source data 1. [file elife-75636-fig1-data1.zip › Fig1 source data 1 for Fig1 B&C/SN shsPTB 3M MZ1.jpg]

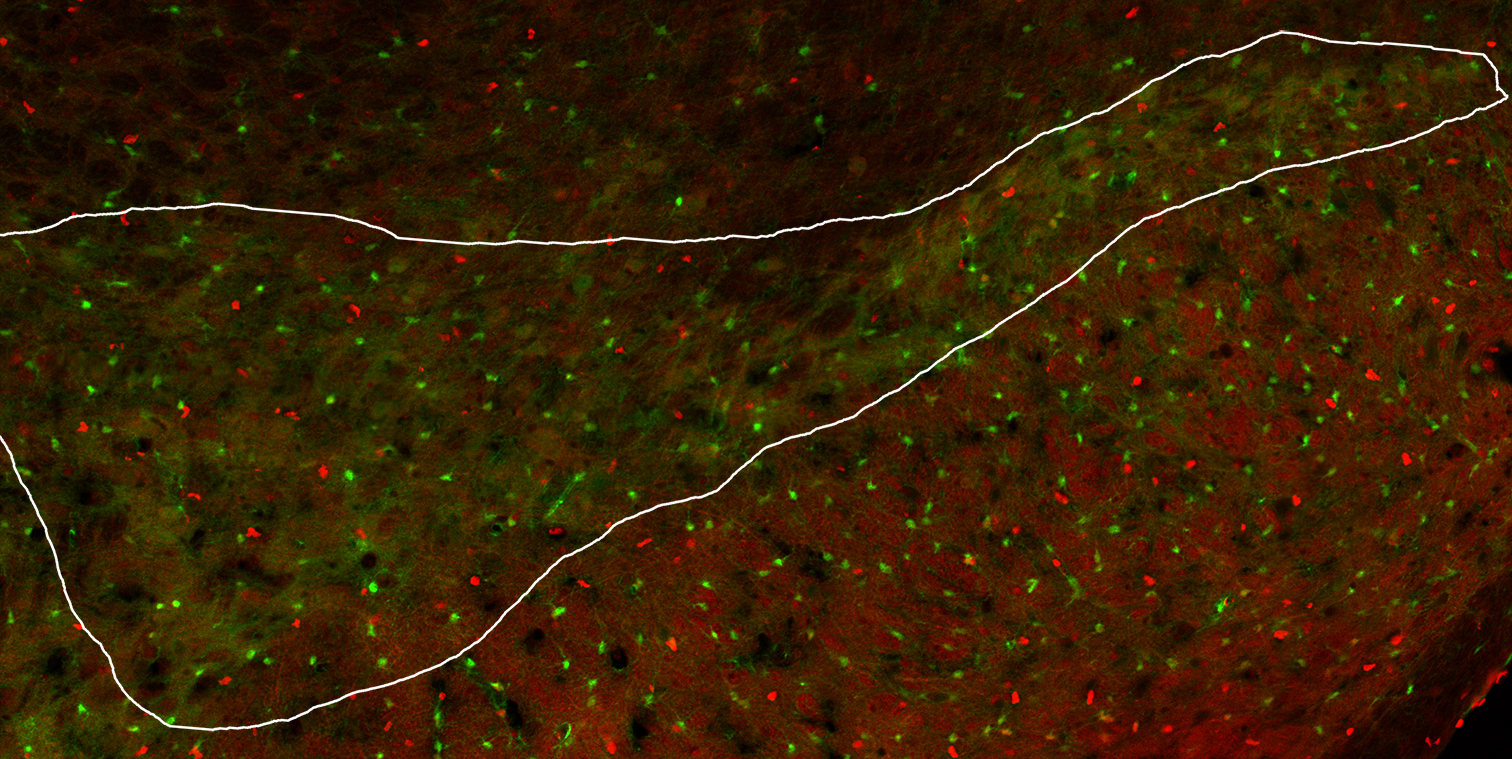

Supplement: Figure 1—source data 1. [file elife-75636-fig1-data1.zip › Fig1 source data 1 for Fig1 B&C/SN shsPTB 3M MZ2.jpg]

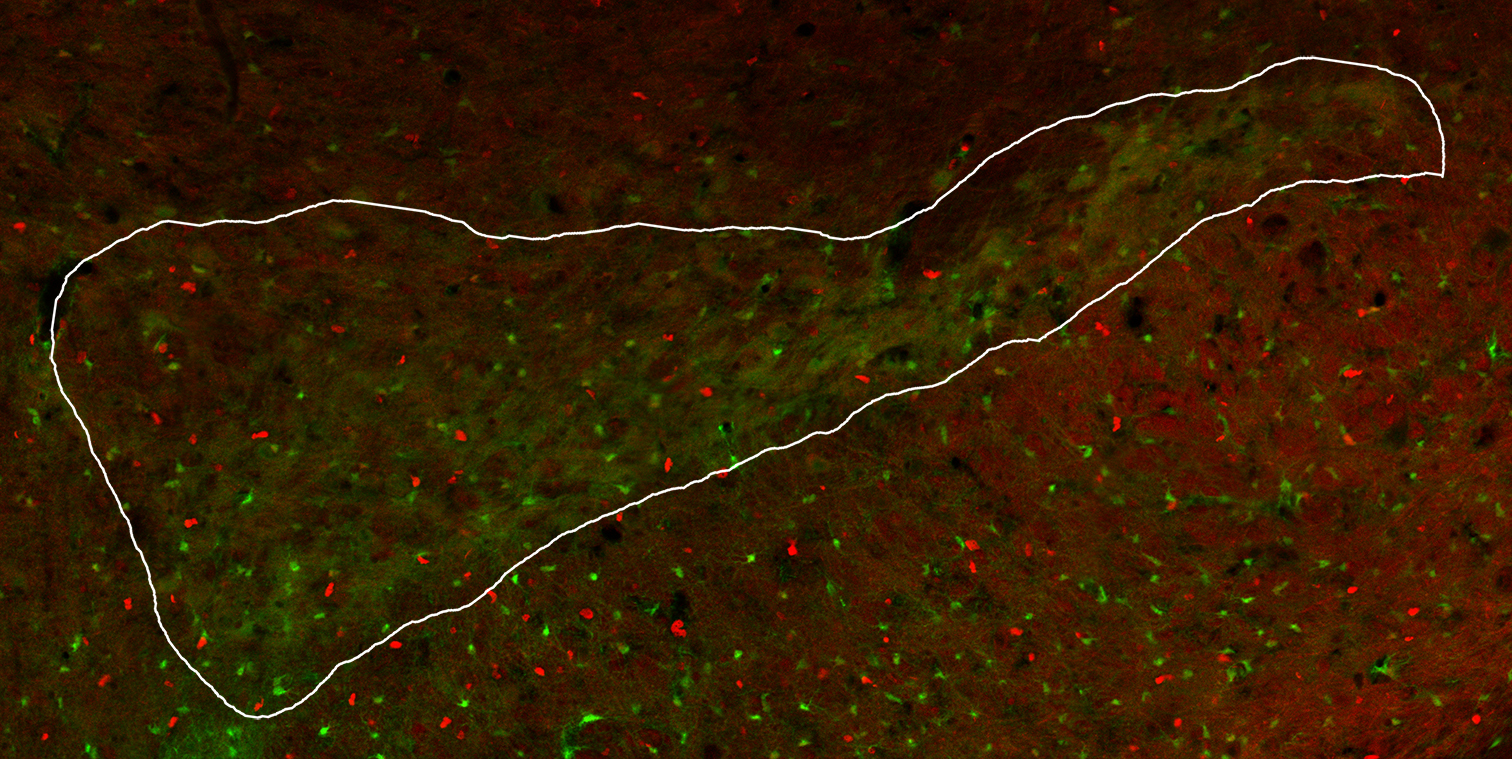

Supplement: Figure 1—source data 1. [file elife-75636-fig1-data1.zip › Fig1 source data 1 for Fig1 B&C/SN shsPTB 3M MZ3.jpg]

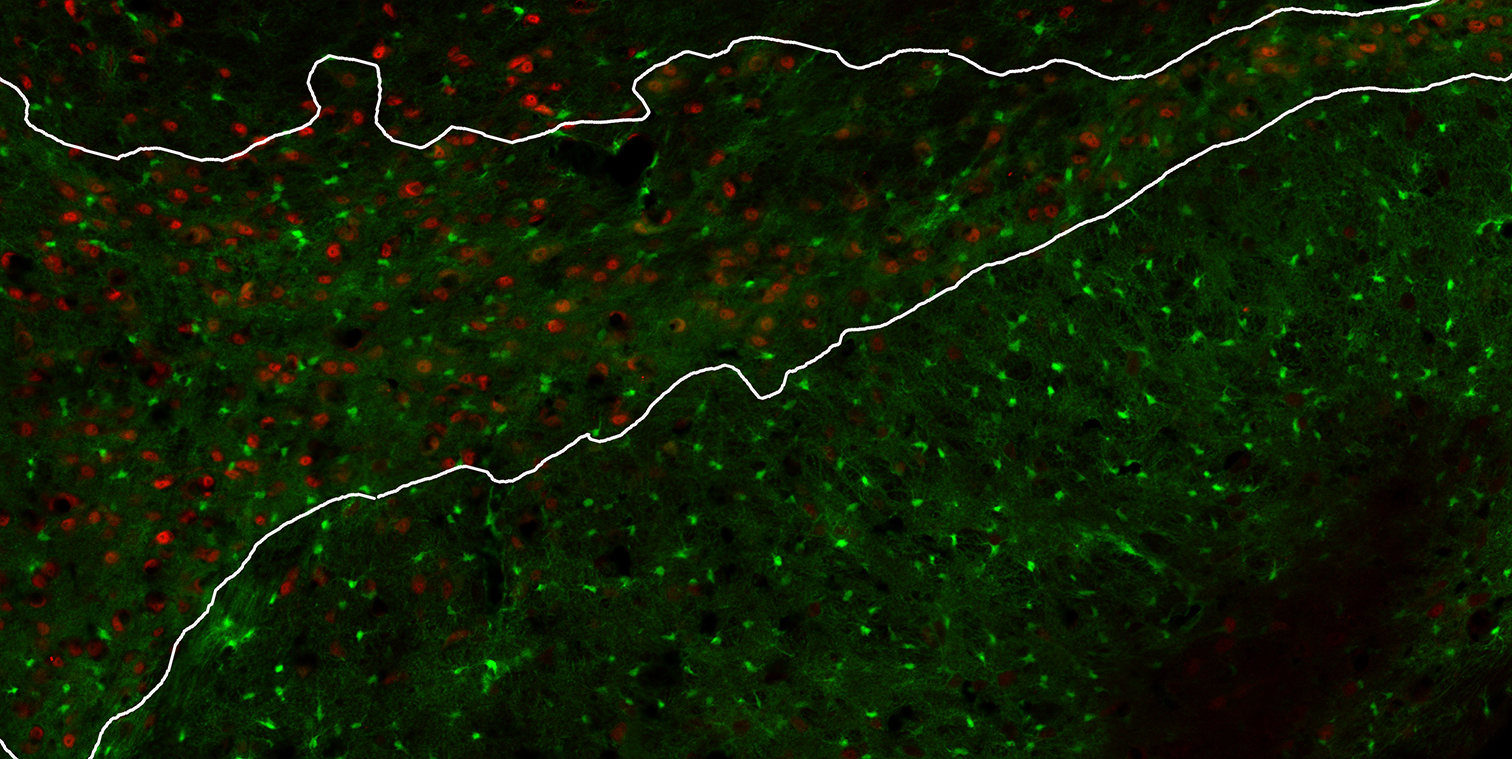

Supplement: Figure 1—source data 2. [file elife-75636-fig1-data2.zip › Fig1 source data 2 for Fig1 D&E/20X/SN shptb 1M MZ1 GFP+neun.jpg]

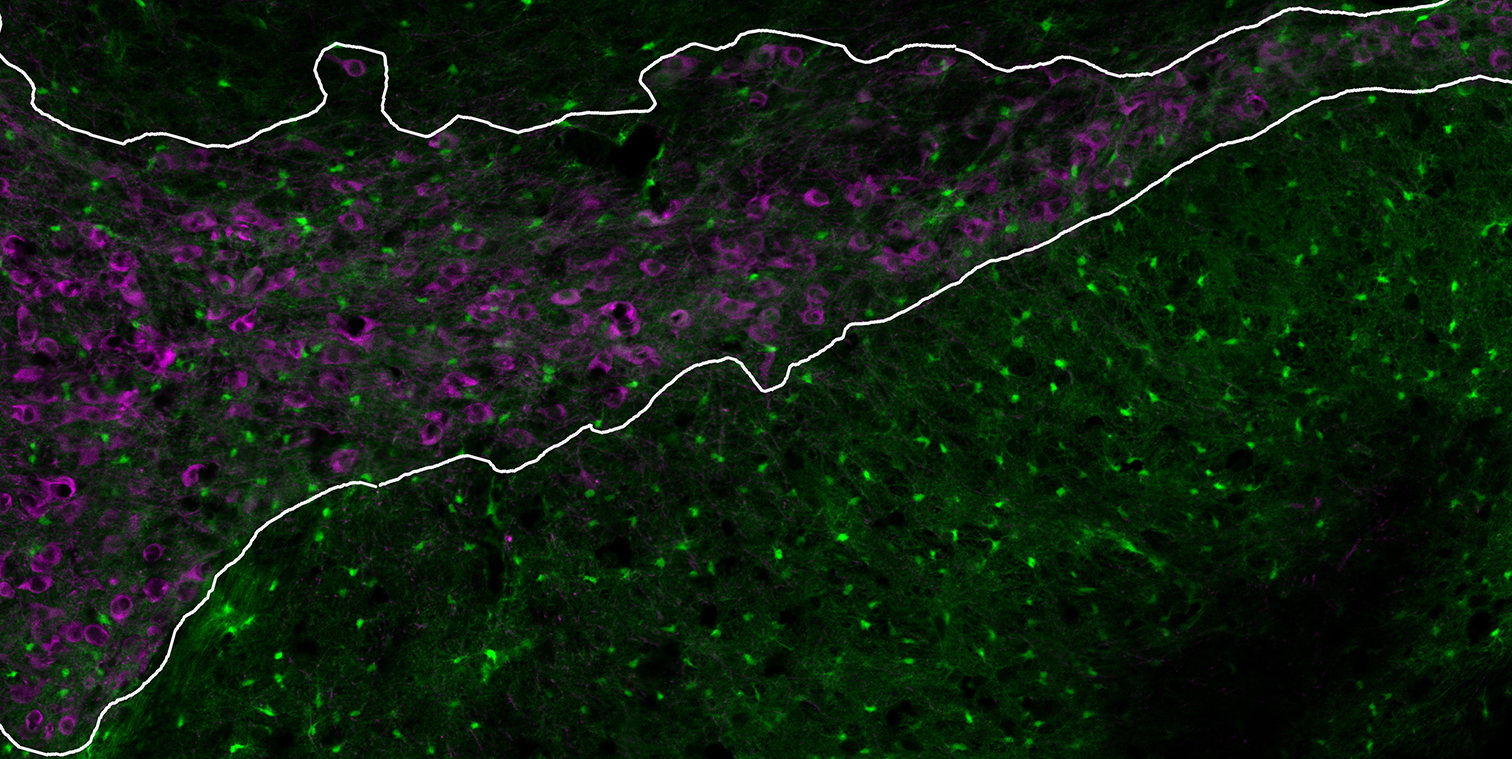

Supplement: Figure 1—source data 2. [file elife-75636-fig1-data2.zip › Fig1 source data 2 for Fig1 D&E/20X/SN shptb 1M MZ1 GFP+TH.jpg]

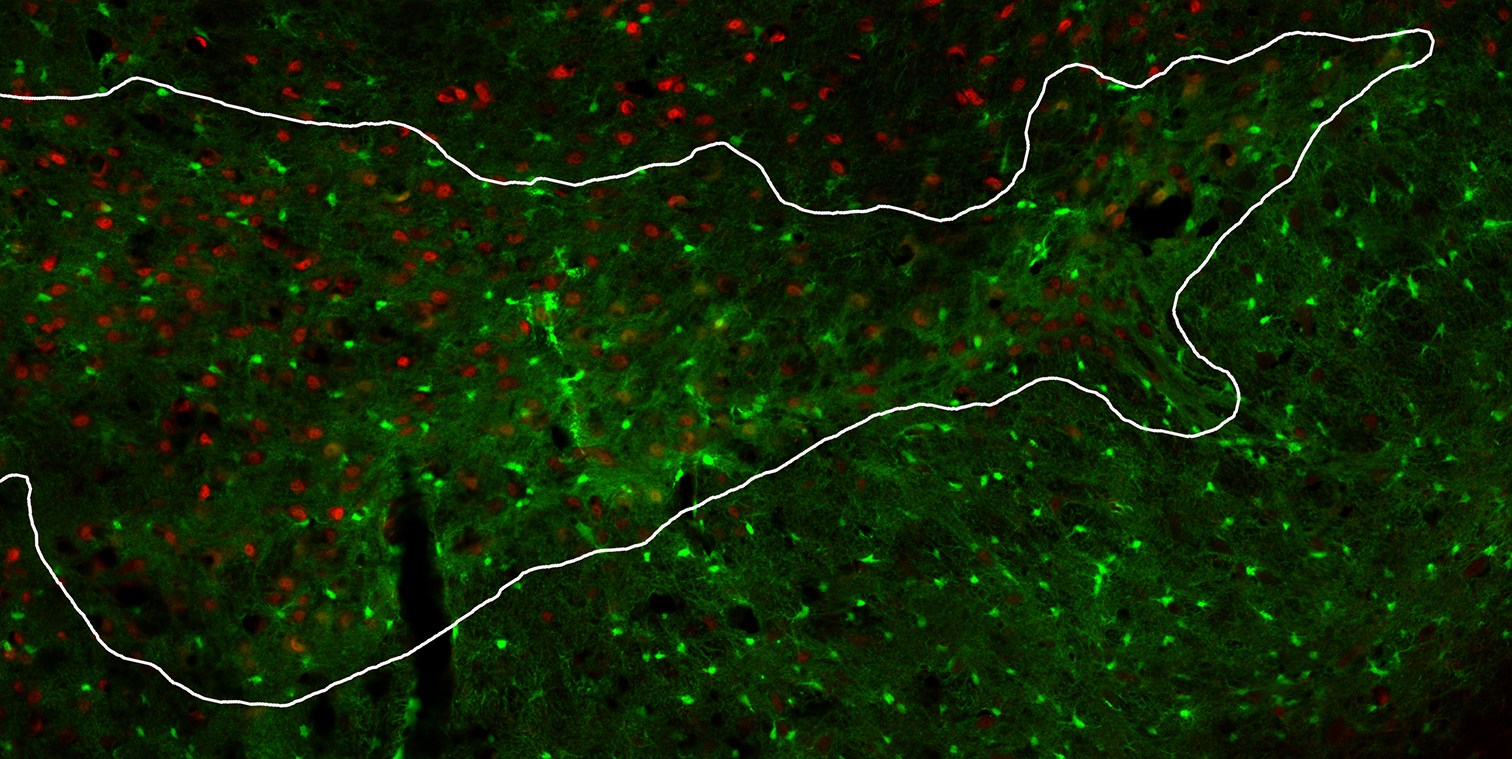

Supplement: Figure 1—source data 2. [file elife-75636-fig1-data2.zip › Fig1 source data 2 for Fig1 D&E/20X/SN shptb 1M MZ2 GFP+neun.jpg]

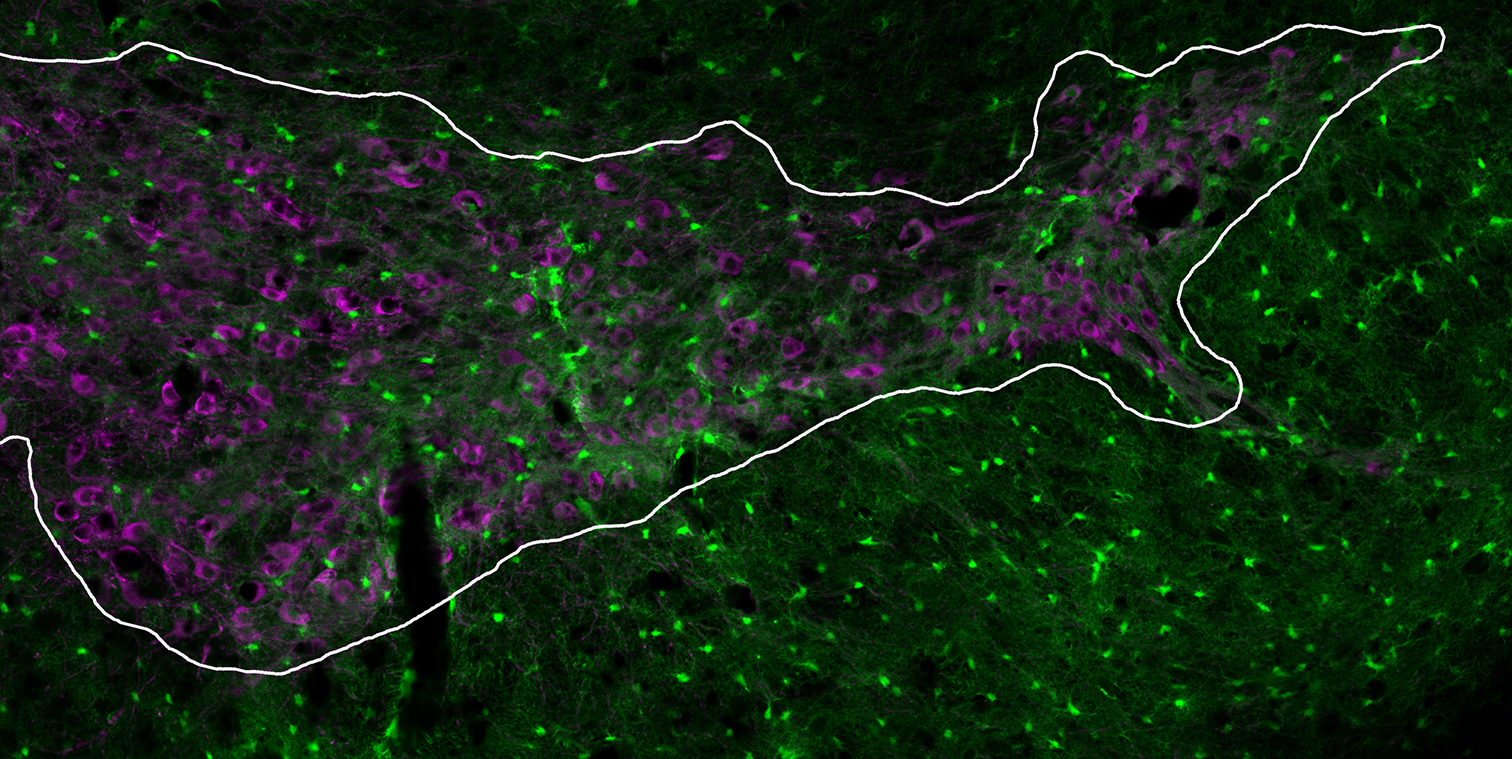

Supplement: Figure 1—source data 2. [file elife-75636-fig1-data2.zip › Fig1 source data 2 for Fig1 D&E/20X/SN shptb 1M MZ2 GFP+TH.jpg]

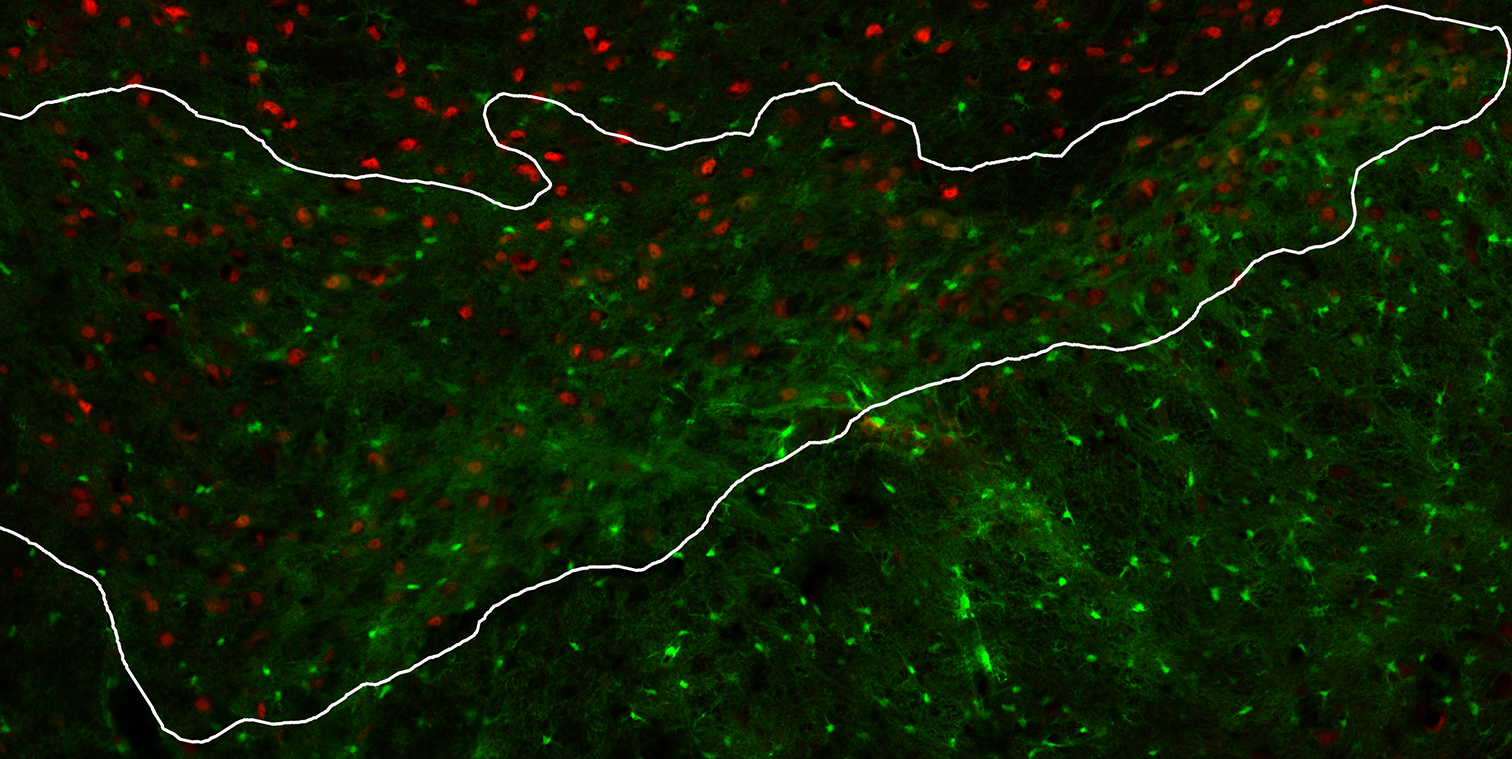

Supplement: Figure 1—source data 2. [file elife-75636-fig1-data2.zip › Fig1 source data 2 for Fig1 D&E/20X/SN shptb 1M MZ3 GFP+neun.jpg]

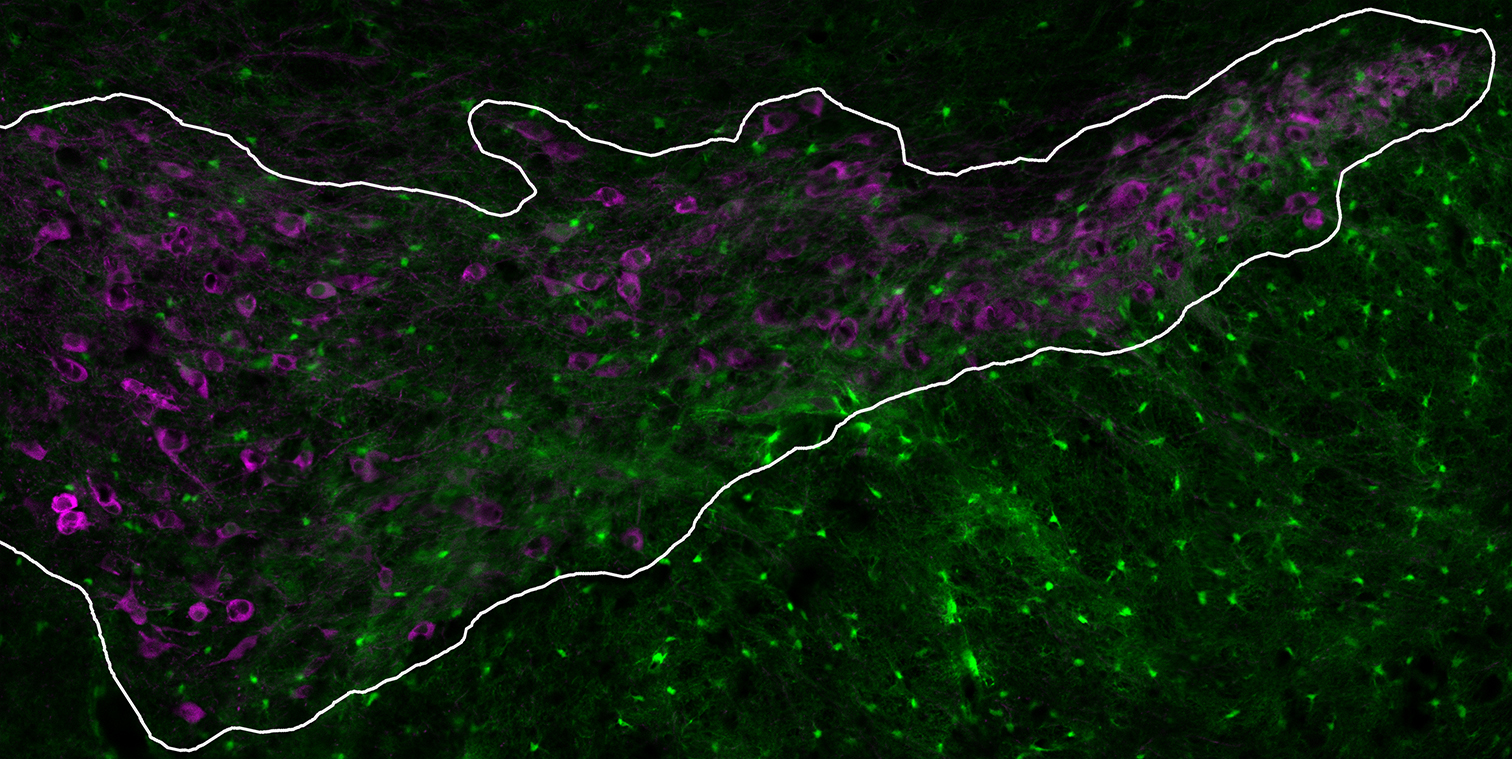

Supplement: Figure 1—source data 2. [file elife-75636-fig1-data2.zip › Fig1 source data 2 for Fig1 D&E/20X/SN shptb 1M MZ3 GFP+TH.jpg]

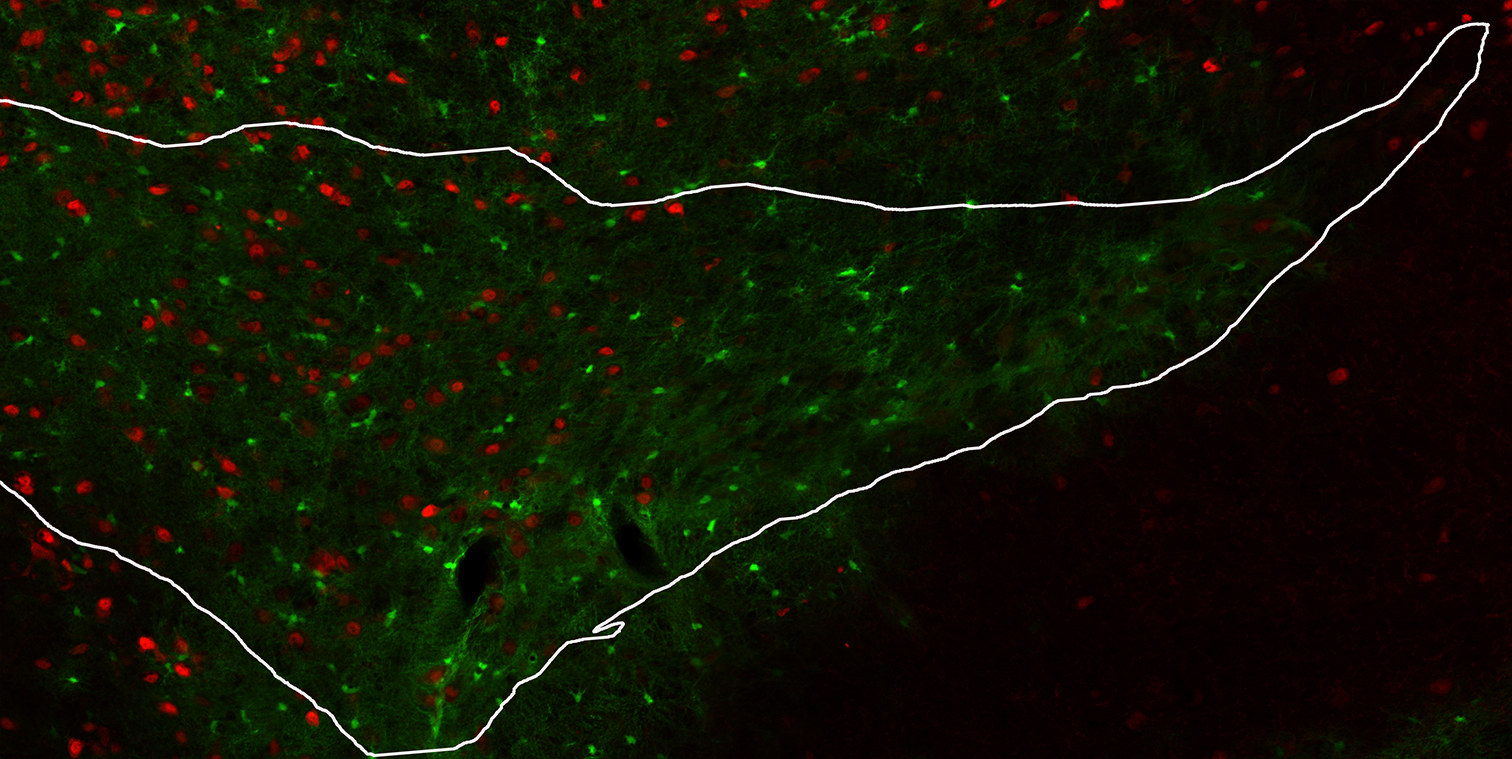

Supplement: Figure 1—source data 2. [file elife-75636-fig1-data2.zip › Fig1 source data 2 for Fig1 D&E/20X/SN shptb 2M MZ1 GFP+neun.jpg]

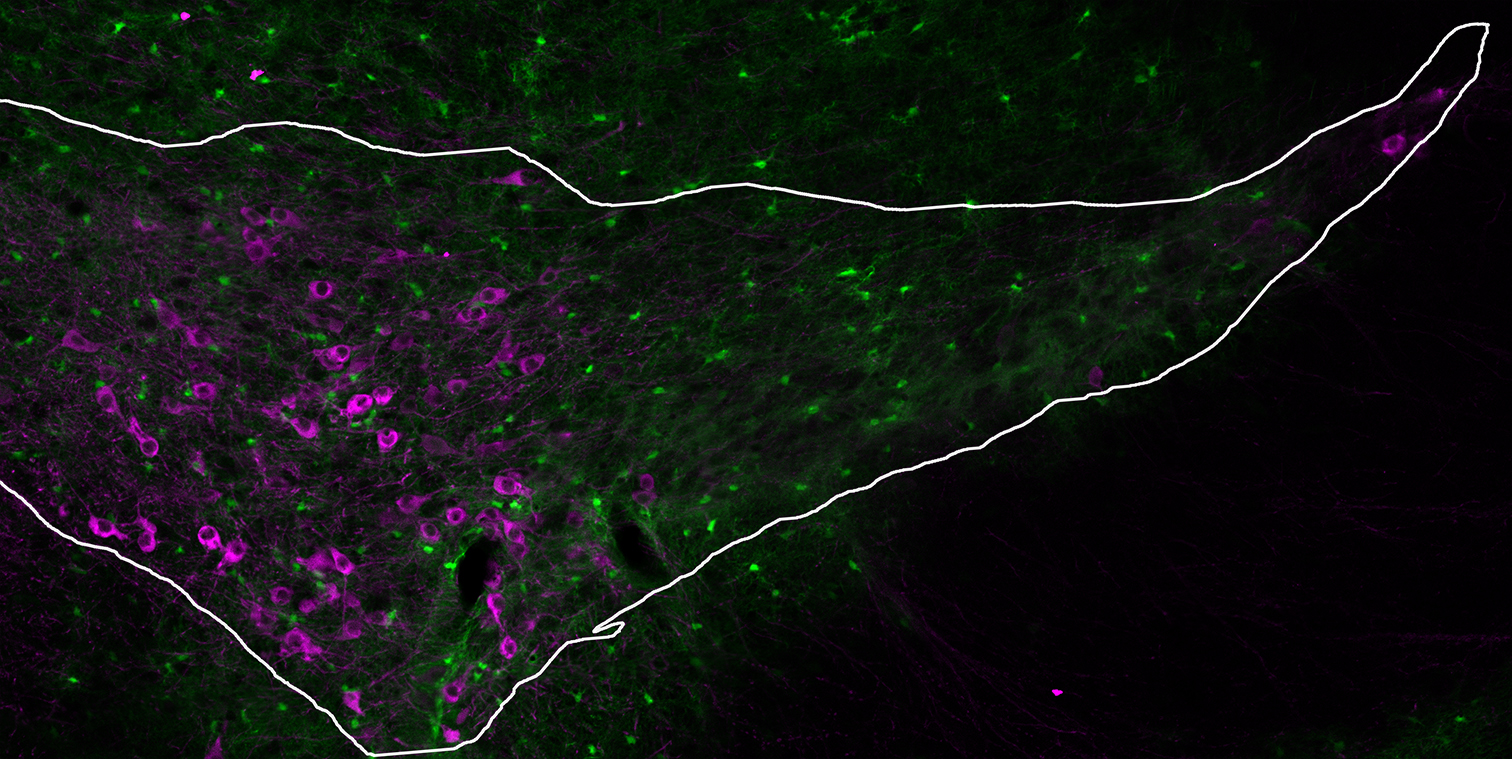

Supplement: Figure 1—source data 2. [file elife-75636-fig1-data2.zip › Fig1 source data 2 for Fig1 D&E/20X/SN shptb 2M MZ1 GFP+TH.jpg]

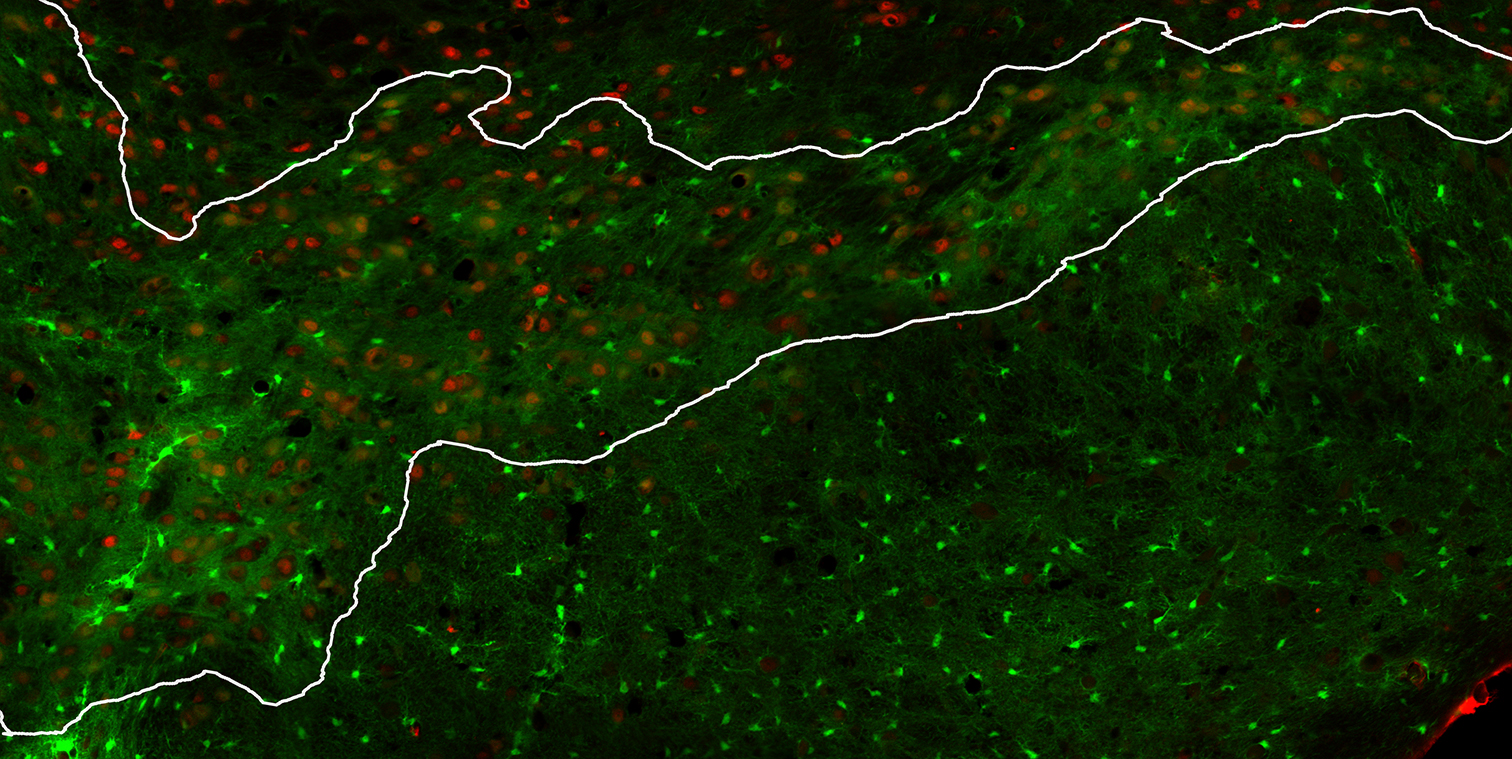

Supplement: Figure 1—source data 2. [file elife-75636-fig1-data2.zip › Fig1 source data 2 for Fig1 D&E/20X/SN shptb 2M MZ2 GFP+neun.jpg]

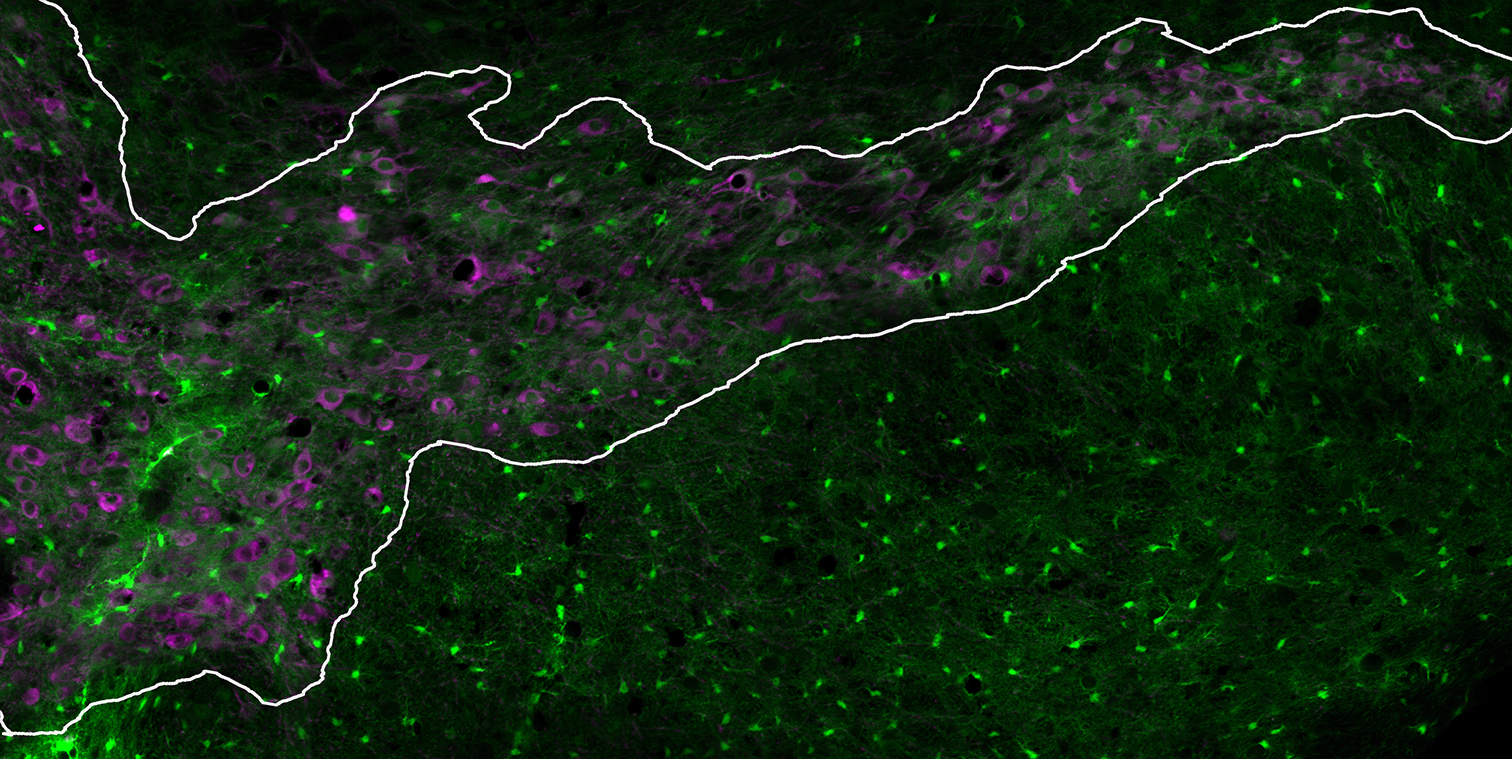

Supplement: Figure 1—source data 2. [file elife-75636-fig1-data2.zip › Fig1 source data 2 for Fig1 D&E/20X/SN shptb 2M MZ2 GFP+TH.jpg]

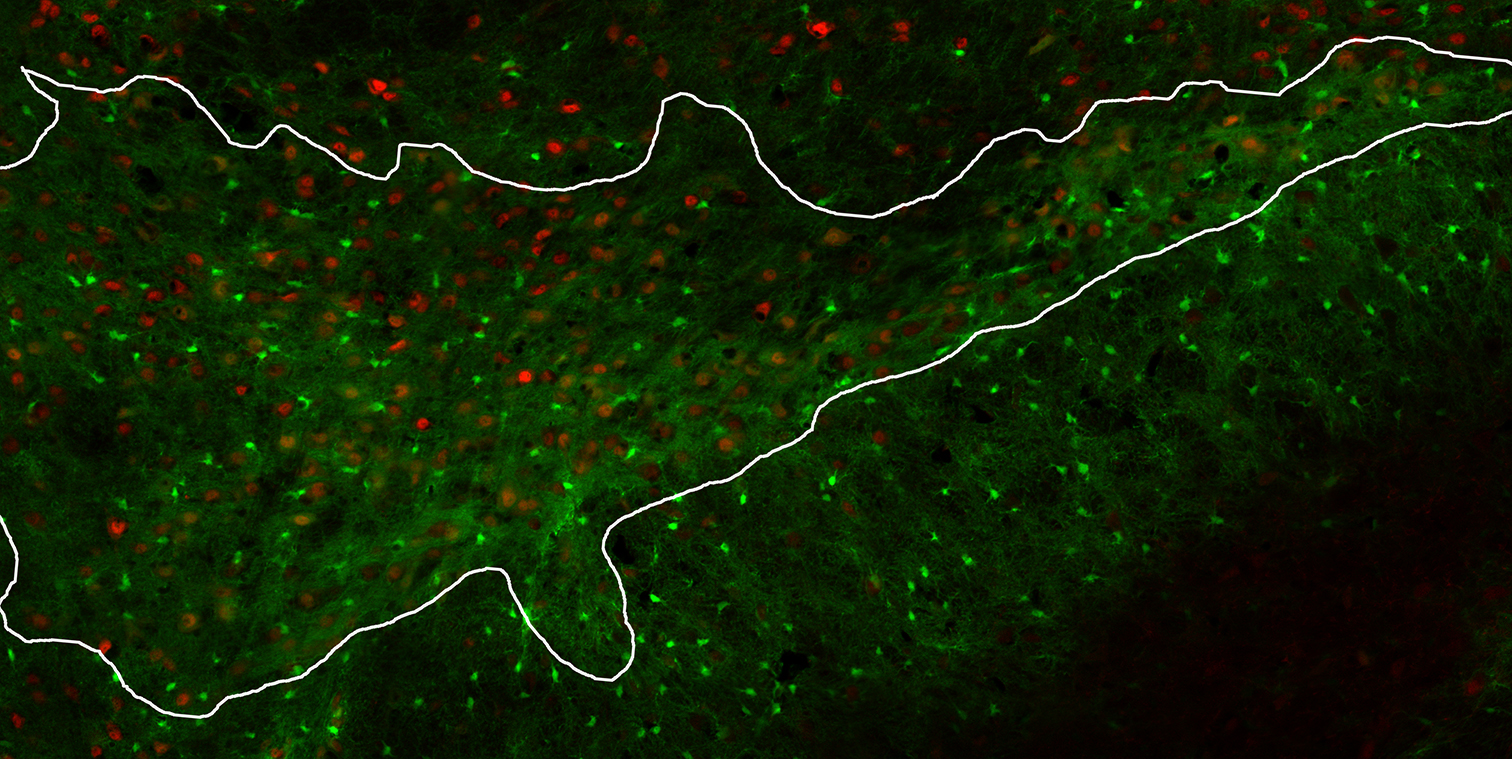

Supplement: Figure 1—source data 2. [file elife-75636-fig1-data2.zip › Fig1 source data 2 for Fig1 D&E/20X/SN shptb 2M MZ3 GFP+neun.jpg]

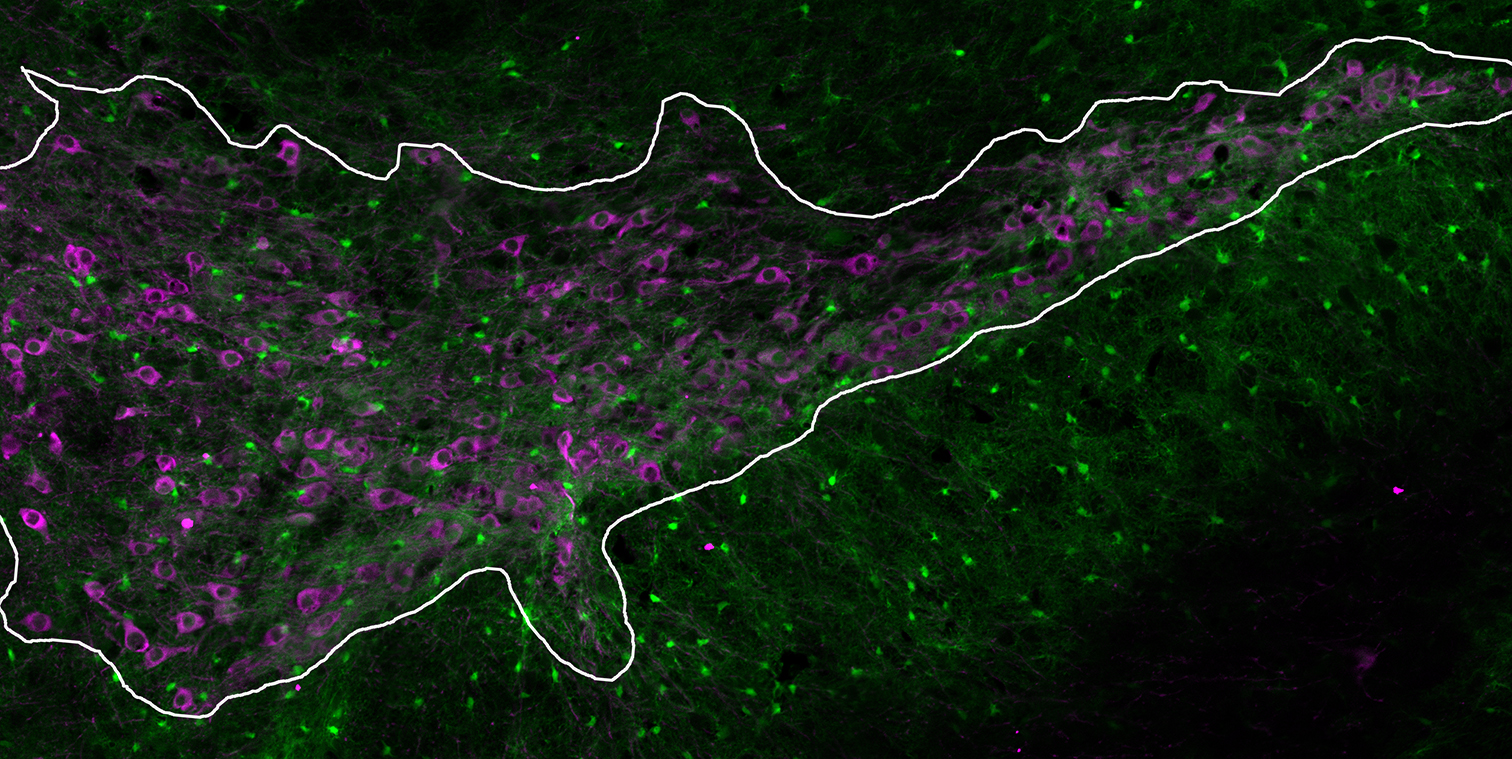

Supplement: Figure 1—source data 2. [file elife-75636-fig1-data2.zip › Fig1 source data 2 for Fig1 D&E/20X/SN shptb 2M MZ3 GFP+TH.jpg]

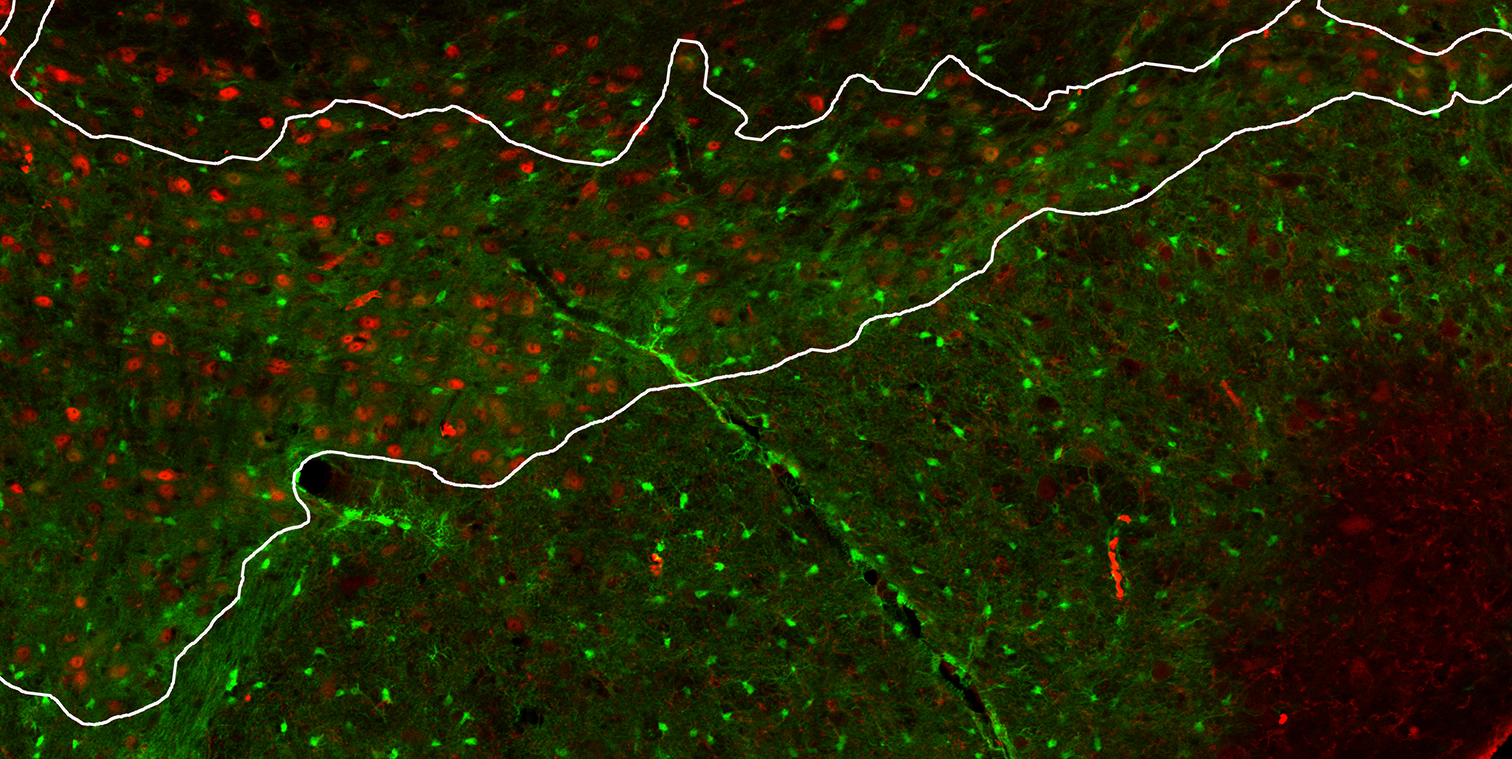

Supplement: Figure 1—source data 2. [file elife-75636-fig1-data2.zip › Fig1 source data 2 for Fig1 D&E/20X/SN shptb 3M MZ1 GFP+neun.jpg]

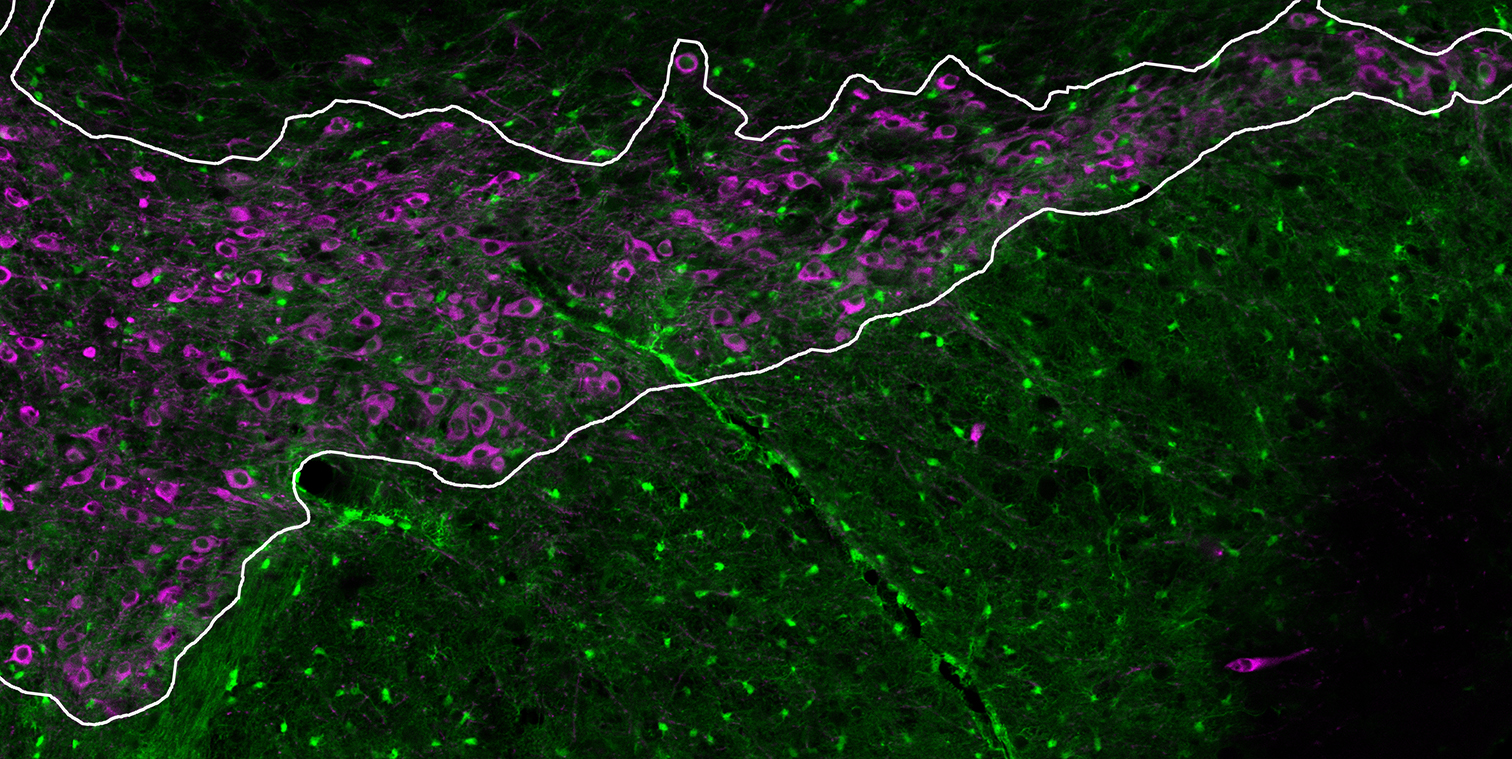

Supplement: Figure 1—source data 2. [file elife-75636-fig1-data2.zip › Fig1 source data 2 for Fig1 D&E/20X/SN shptb 3M MZ1 GFP+TH.jpg]

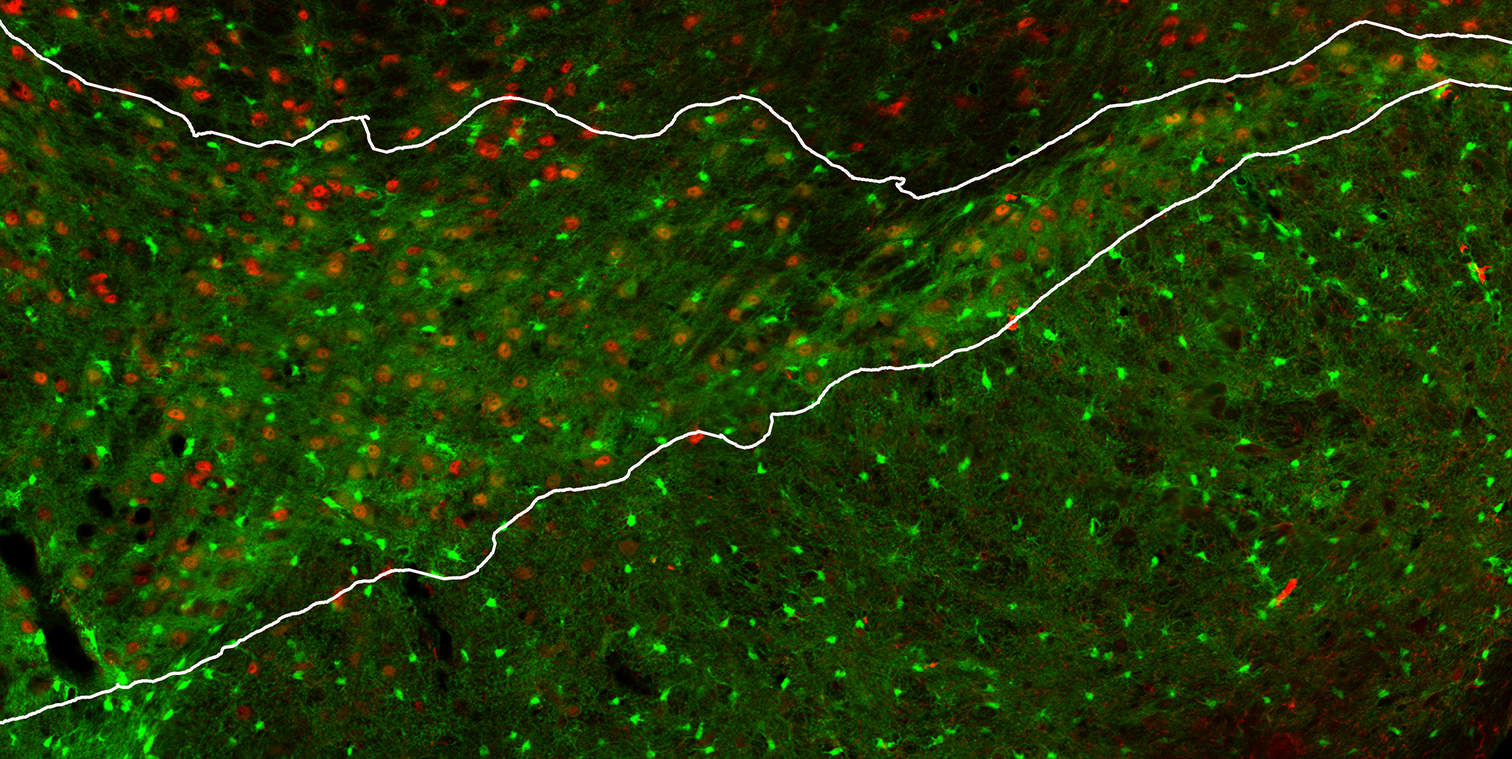

Supplement: Figure 1—source data 2. [file elife-75636-fig1-data2.zip › Fig1 source data 2 for Fig1 D&E/20X/SN shptb 3M MZ2 GFP+neun.jpg]

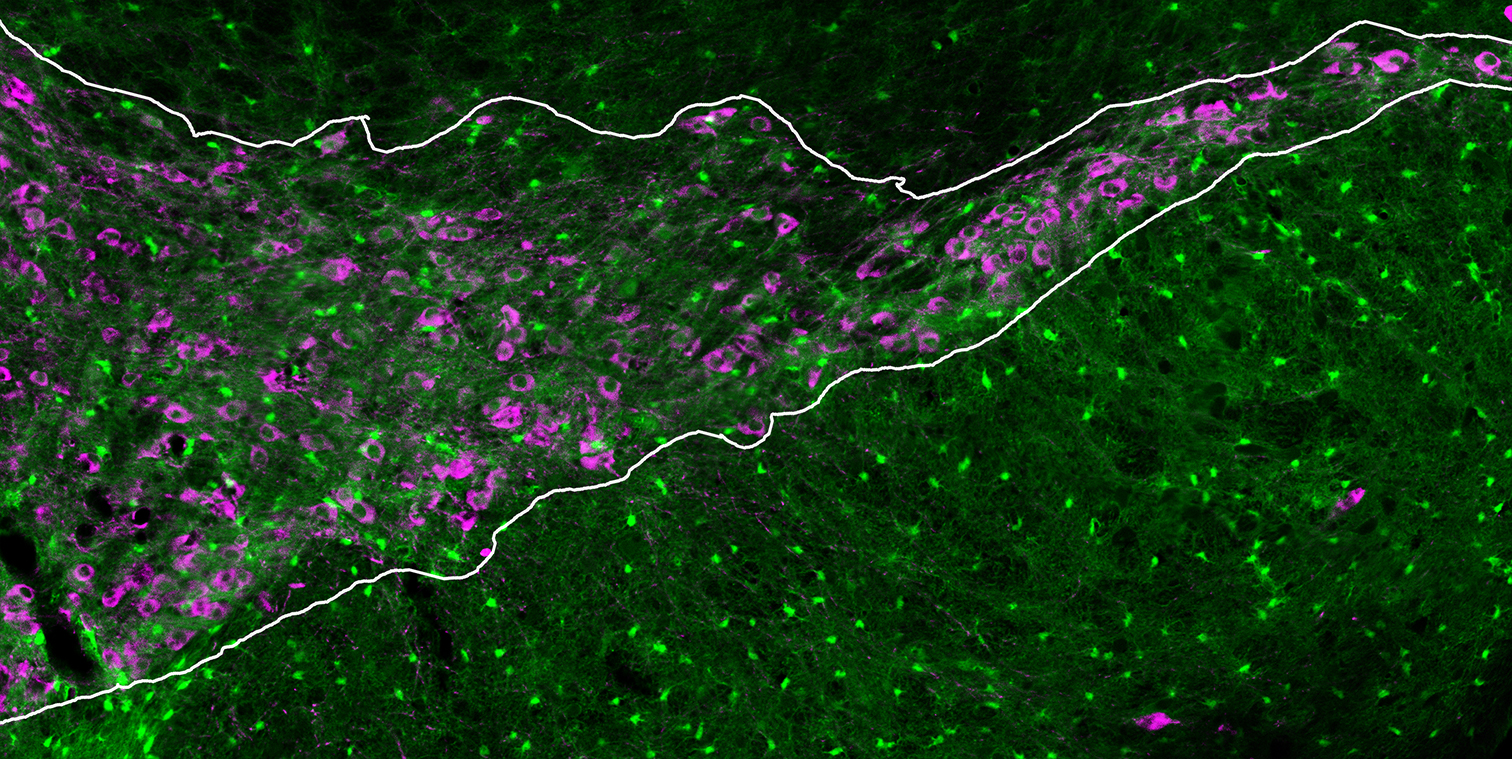

Supplement: Figure 1—source data 2. [file elife-75636-fig1-data2.zip › Fig1 source data 2 for Fig1 D&E/20X/SN shptb 3M MZ2 GFP+TH.jpg]

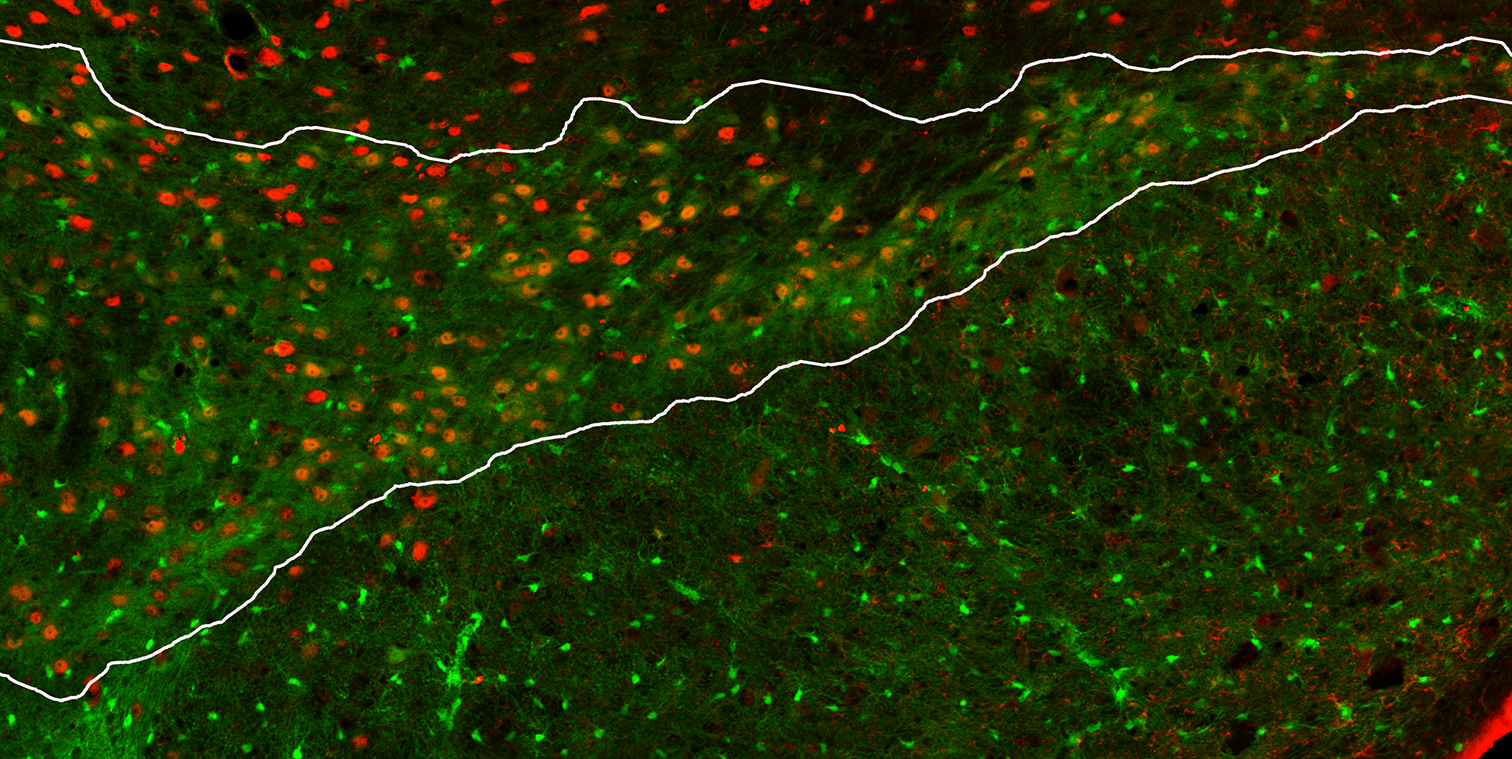

Supplement: Figure 1—source data 2. [file elife-75636-fig1-data2.zip › Fig1 source data 2 for Fig1 D&E/20X/SN shptb 3M MZ3 GFP+neun.jpg]

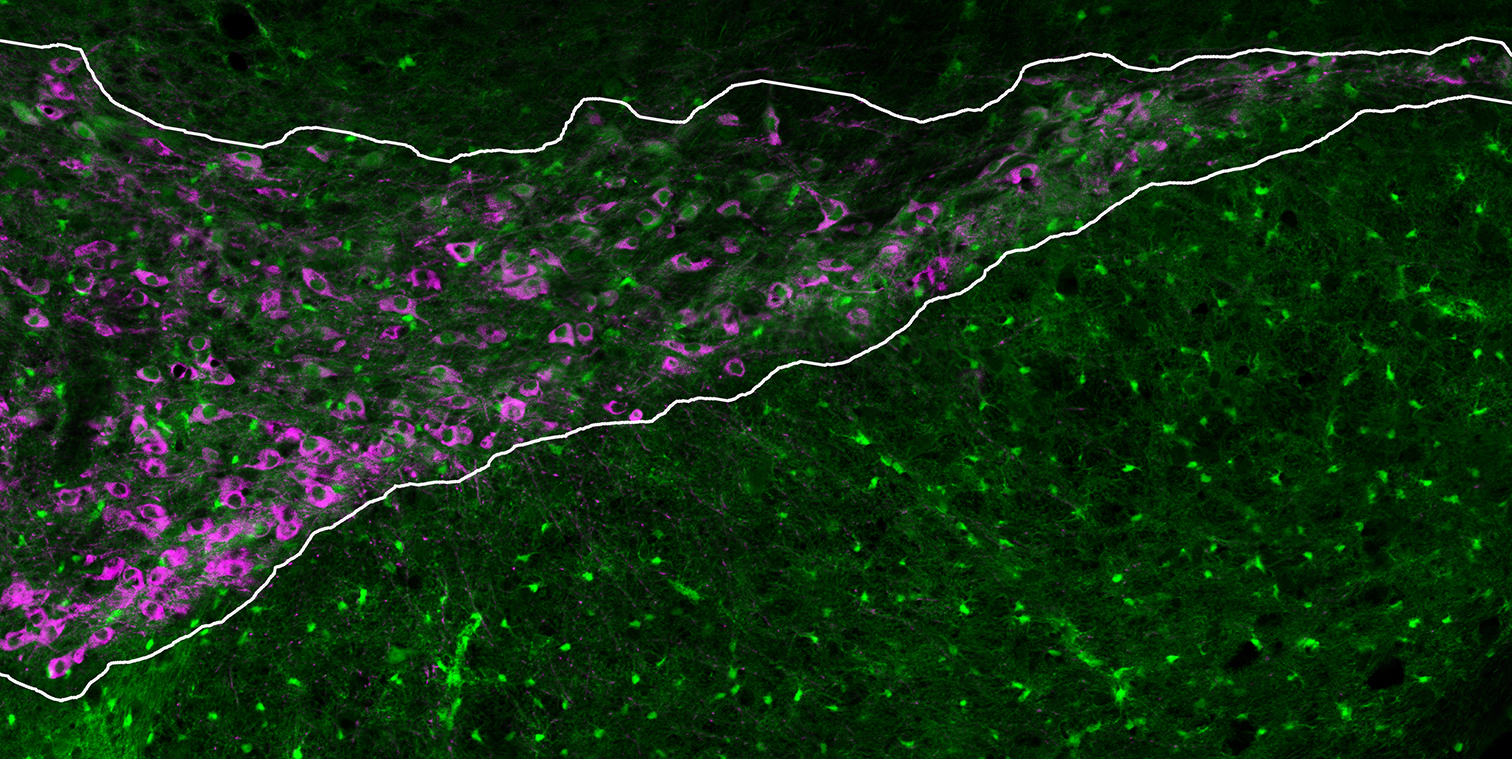

Supplement: Figure 1—source data 2. [file elife-75636-fig1-data2.zip › Fig1 source data 2 for Fig1 D&E/20X/SN shptb 3M MZ3 GFP+TH.jpg]

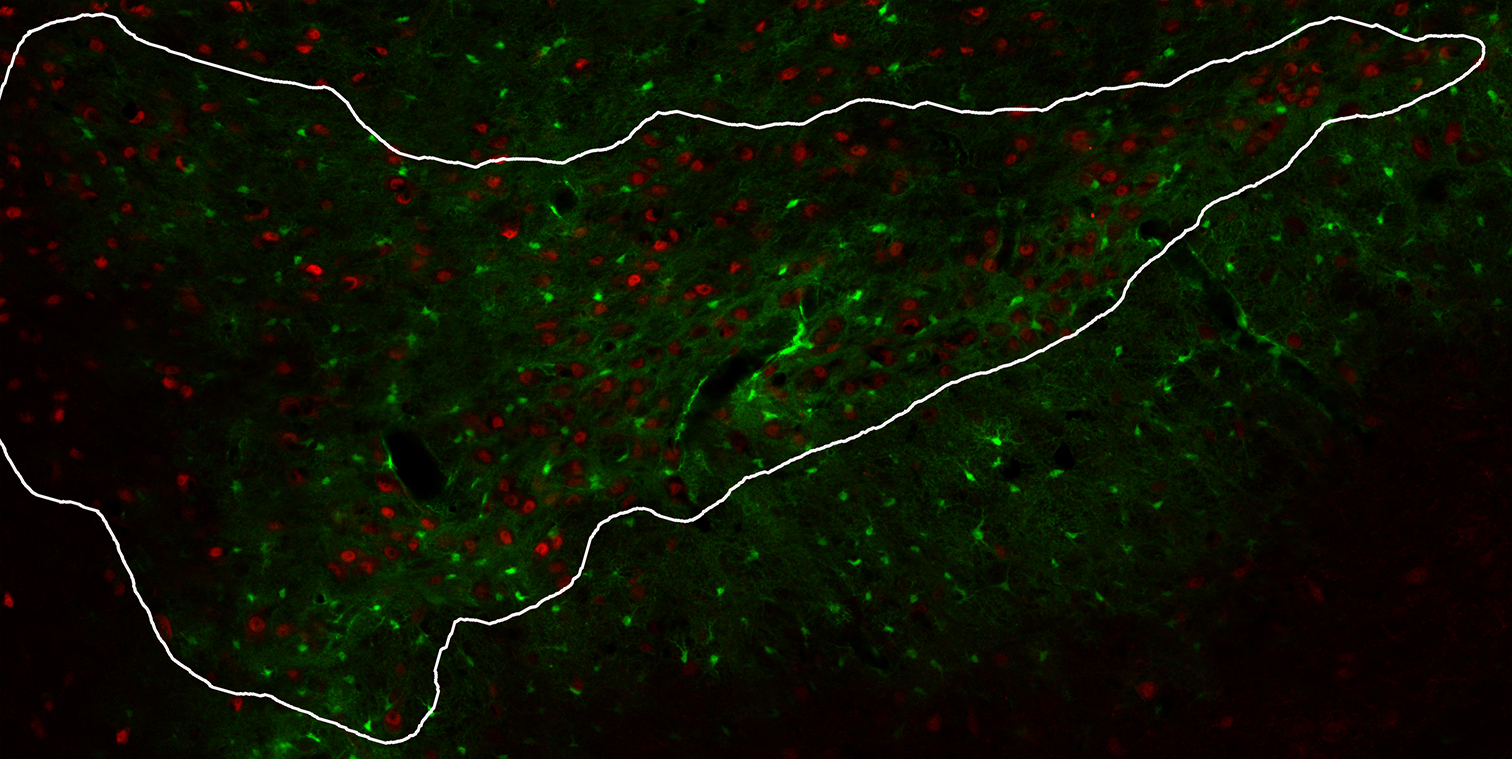

Supplement: Figure 1—source data 2. [file elife-75636-fig1-data2.zip › Fig1 source data 2 for Fig1 D&E/20X/SN shscramble 1M MZ1 GFP+neun.jpg]

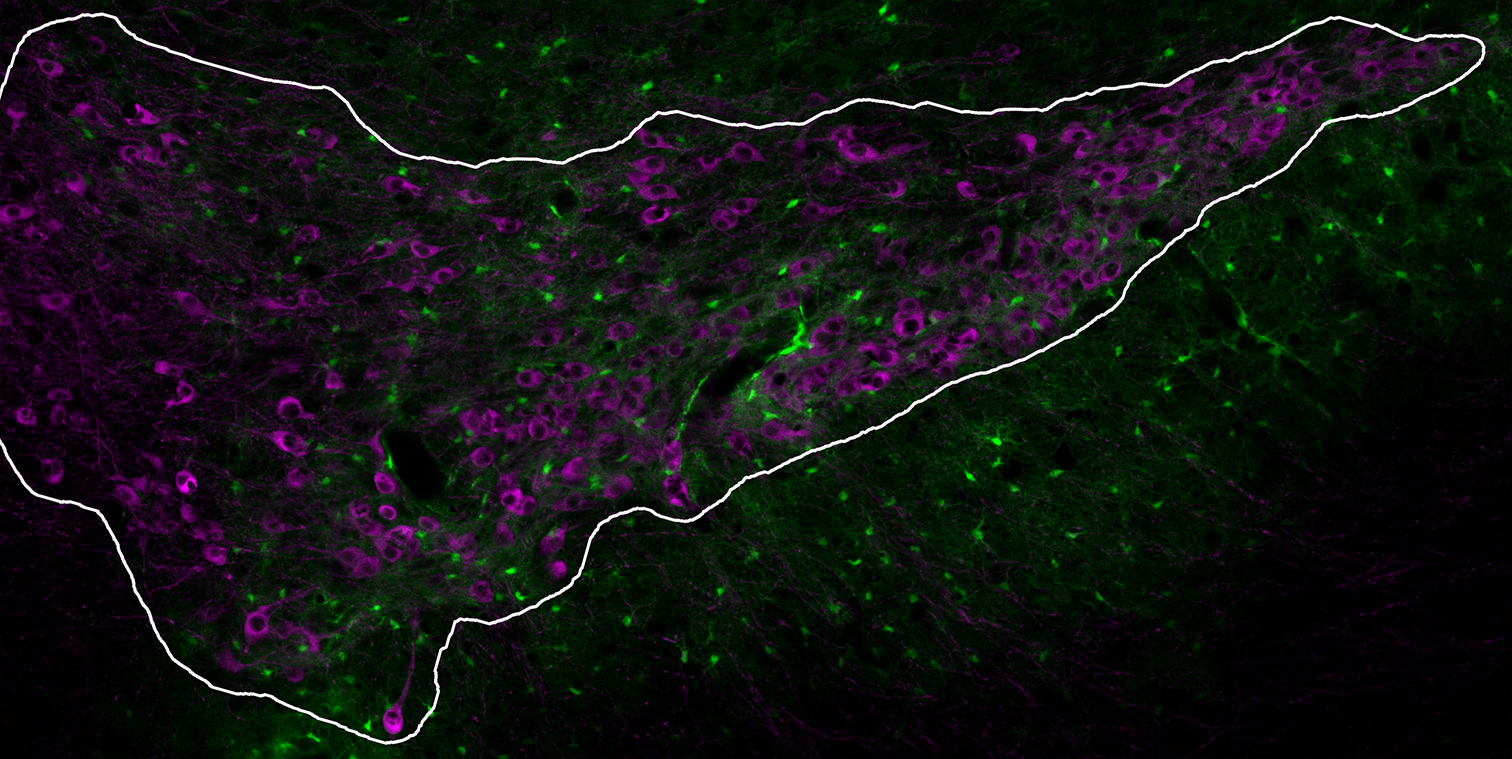

Supplement: Figure 1—source data 2. [file elife-75636-fig1-data2.zip › Fig1 source data 2 for Fig1 D&E/20X/SN shscramble 1M MZ1 GFP+TH.jpg]

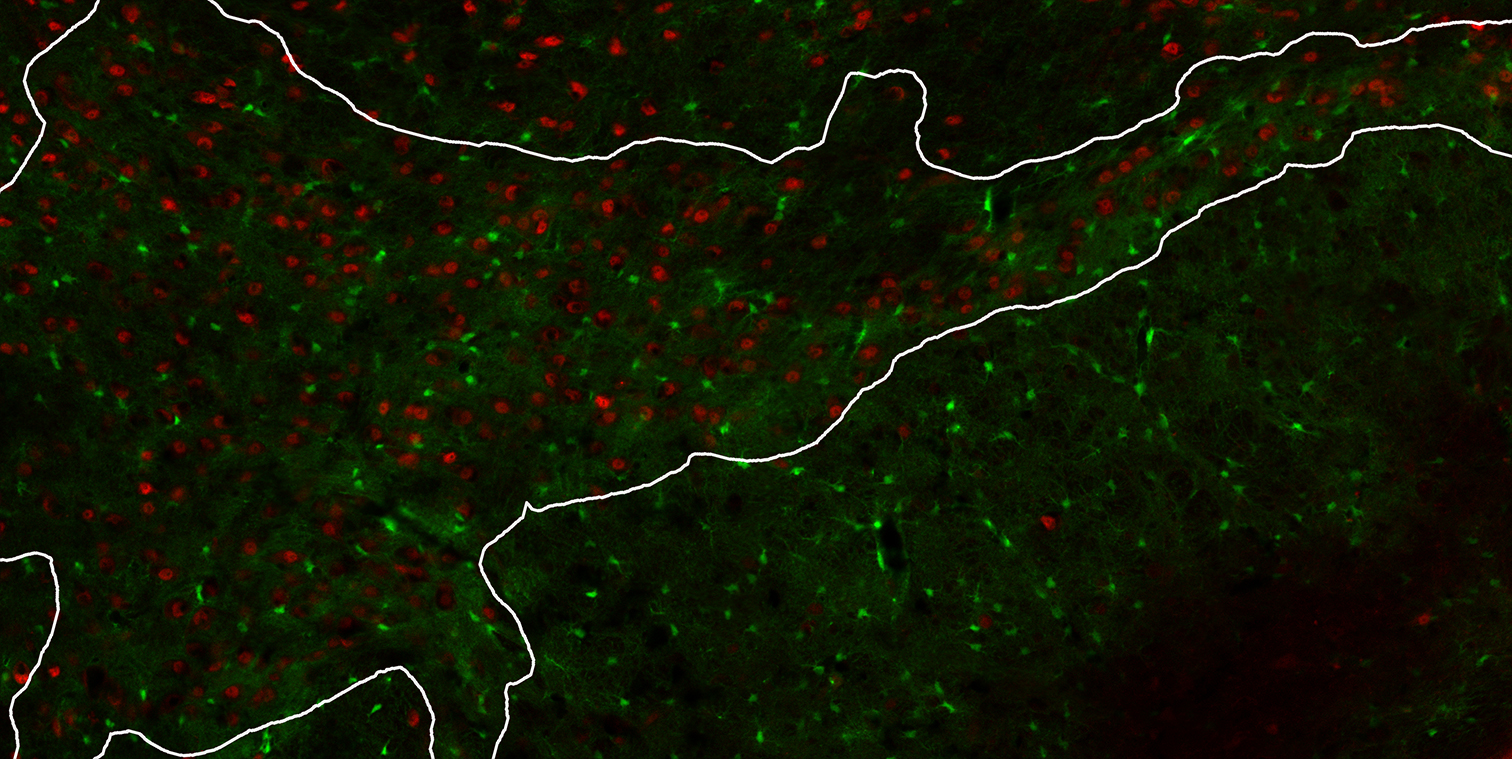

Supplement: Figure 1—source data 2. [file elife-75636-fig1-data2.zip › Fig1 source data 2 for Fig1 D&E/20X/SN shscramble 1M MZ2 GFP+neun.jpg]

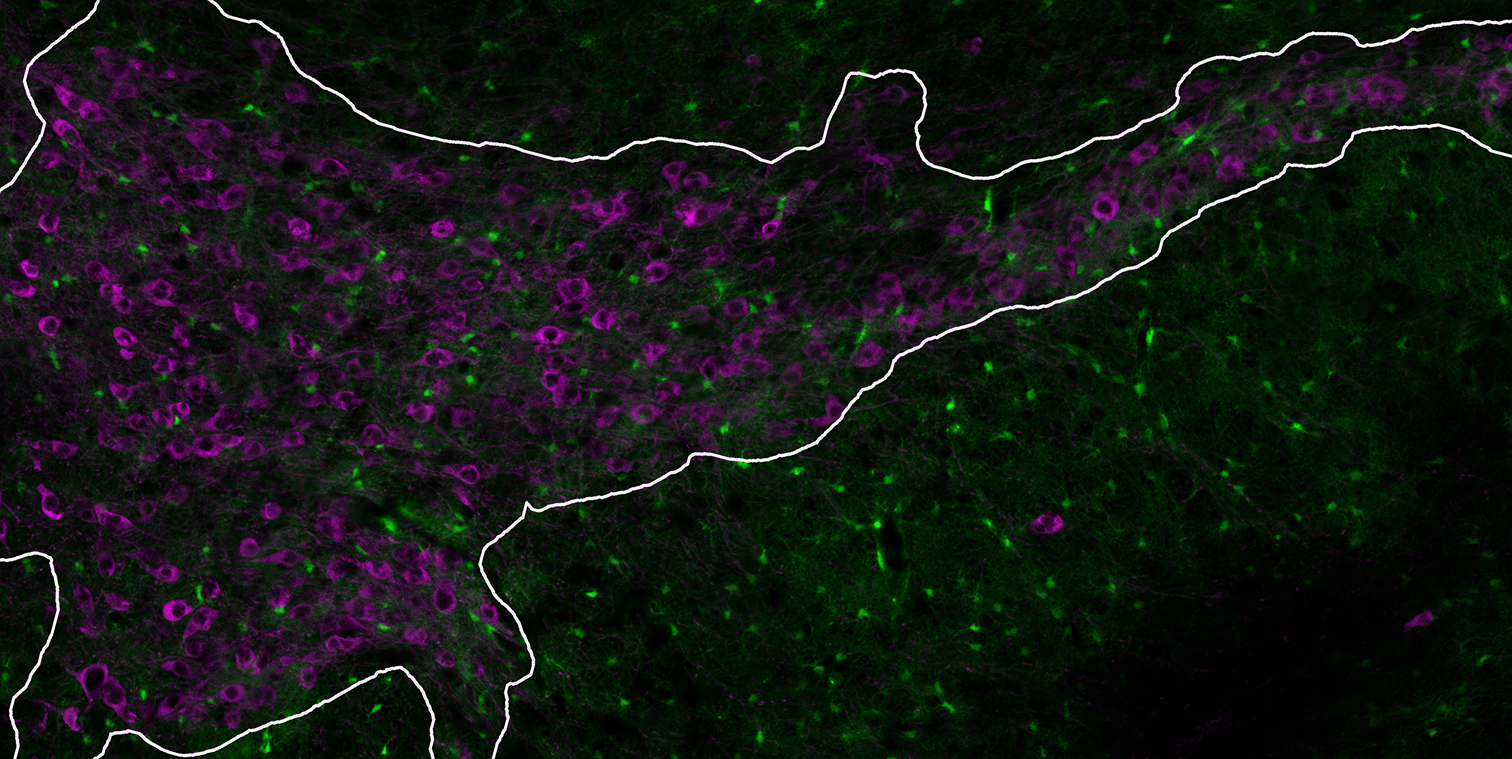

Supplement: Figure 1—source data 2. [file elife-75636-fig1-data2.zip › Fig1 source data 2 for Fig1 D&E/20X/SN shscramble 1M MZ2 GFP+TH.jpg]

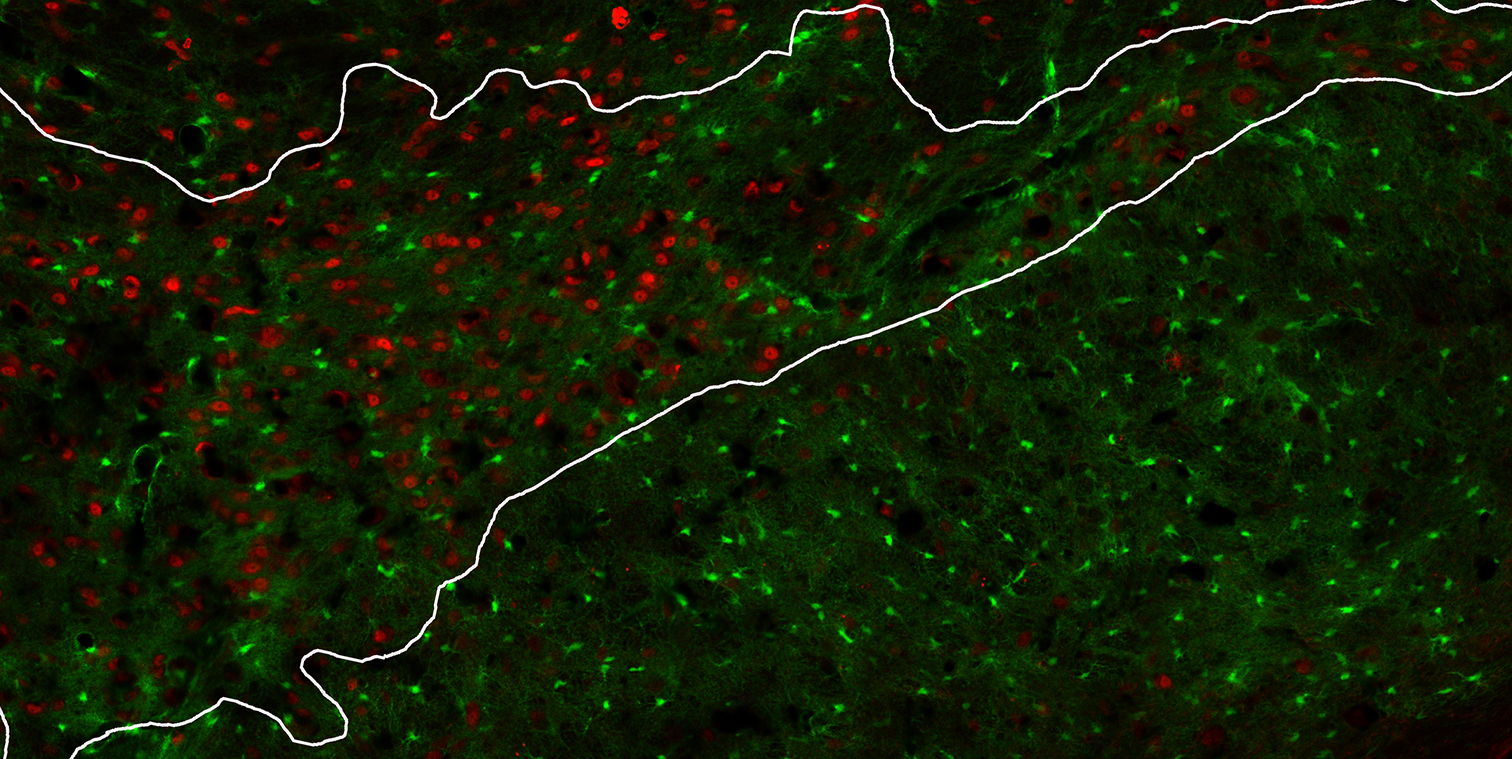

Supplement: Figure 1—source data 2. [file elife-75636-fig1-data2.zip › Fig1 source data 2 for Fig1 D&E/20X/SN shscramble 1M MZ3 GFP+neun.jpg]

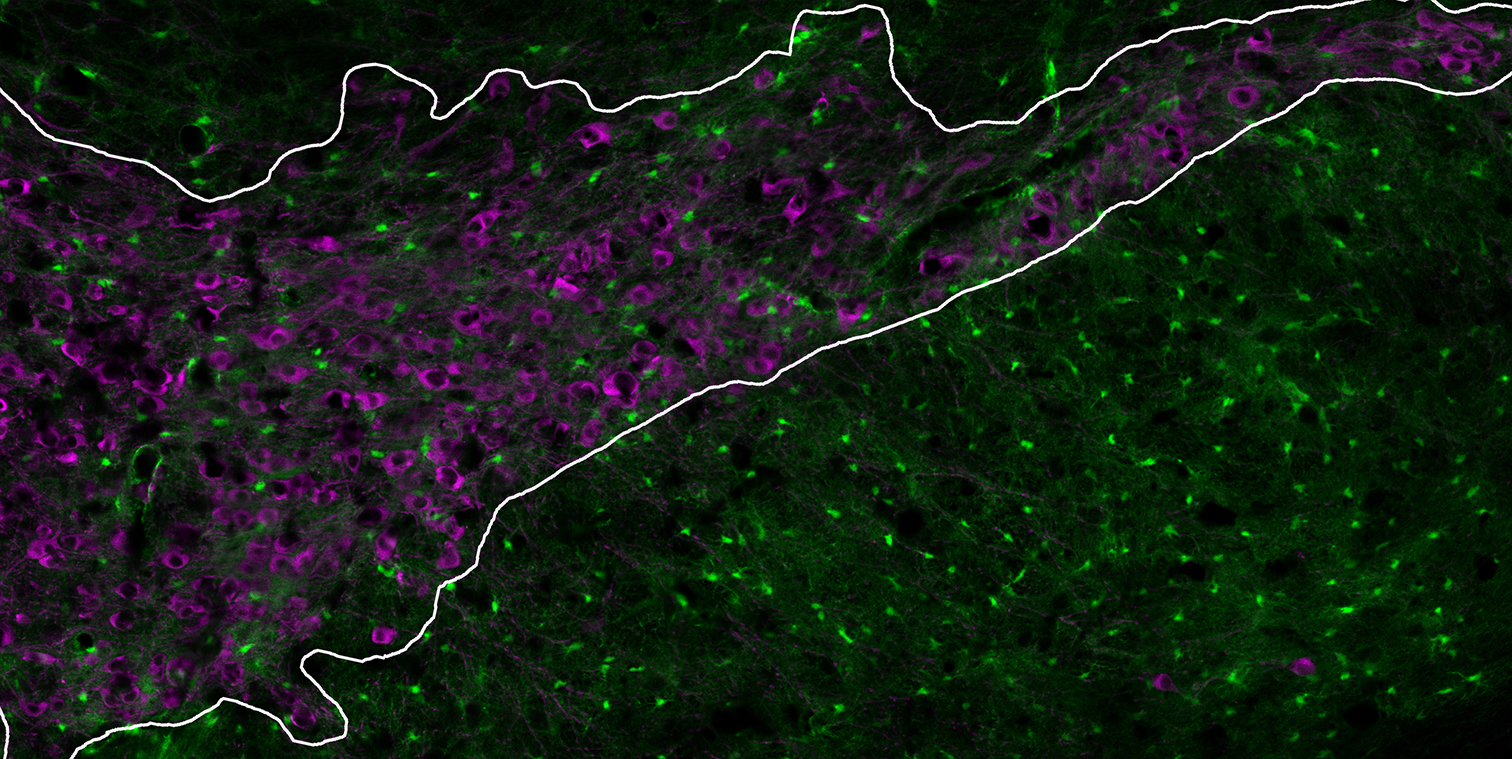

Supplement: Figure 1—source data 2. [file elife-75636-fig1-data2.zip › Fig1 source data 2 for Fig1 D&E/20X/SN shscramble 1M MZ3 GFP+TH.jpg]

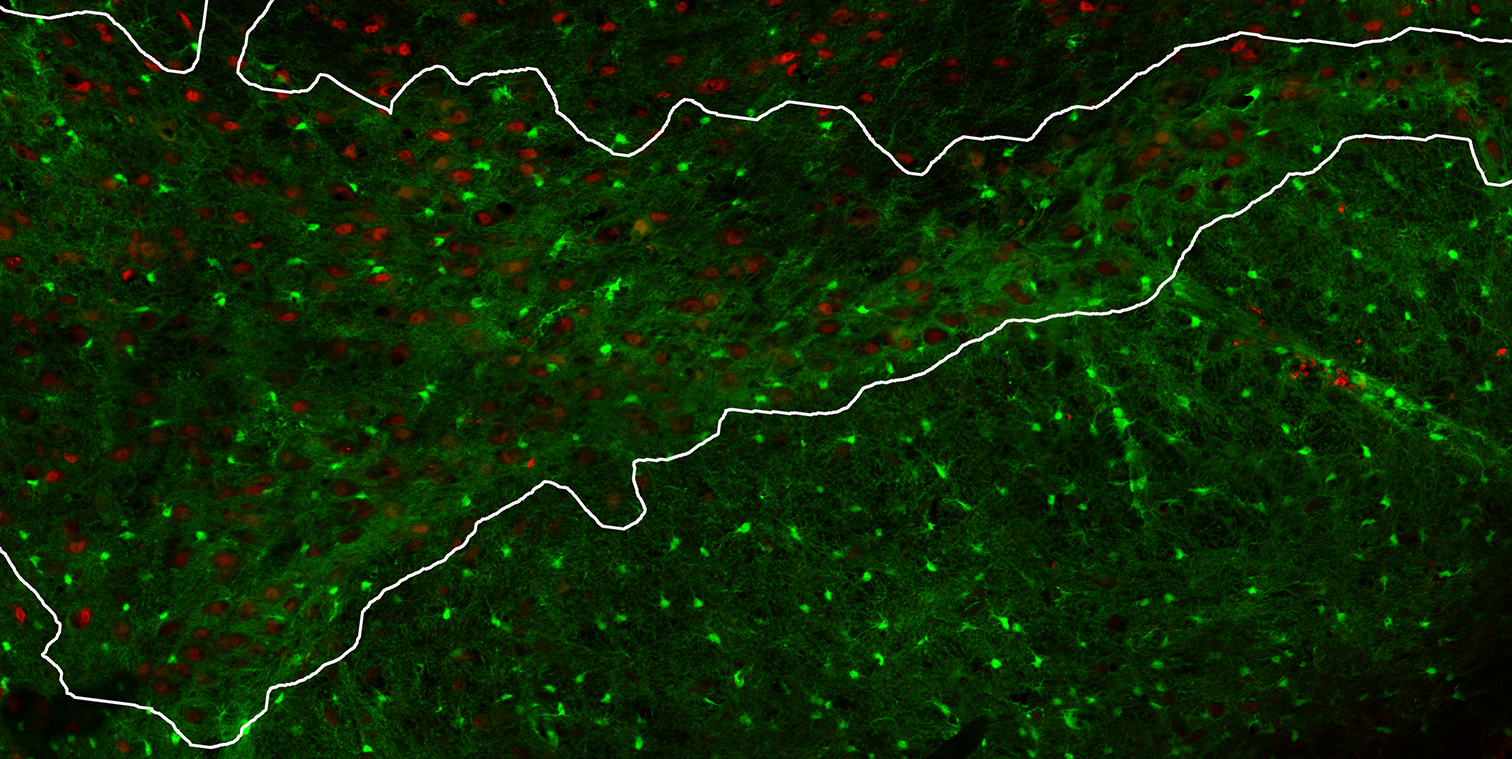

Supplement: Figure 1—source data 2. [file elife-75636-fig1-data2.zip › Fig1 source data 2 for Fig1 D&E/20X/SN shscramble 2M MZ1 GFP+neun.jpg]

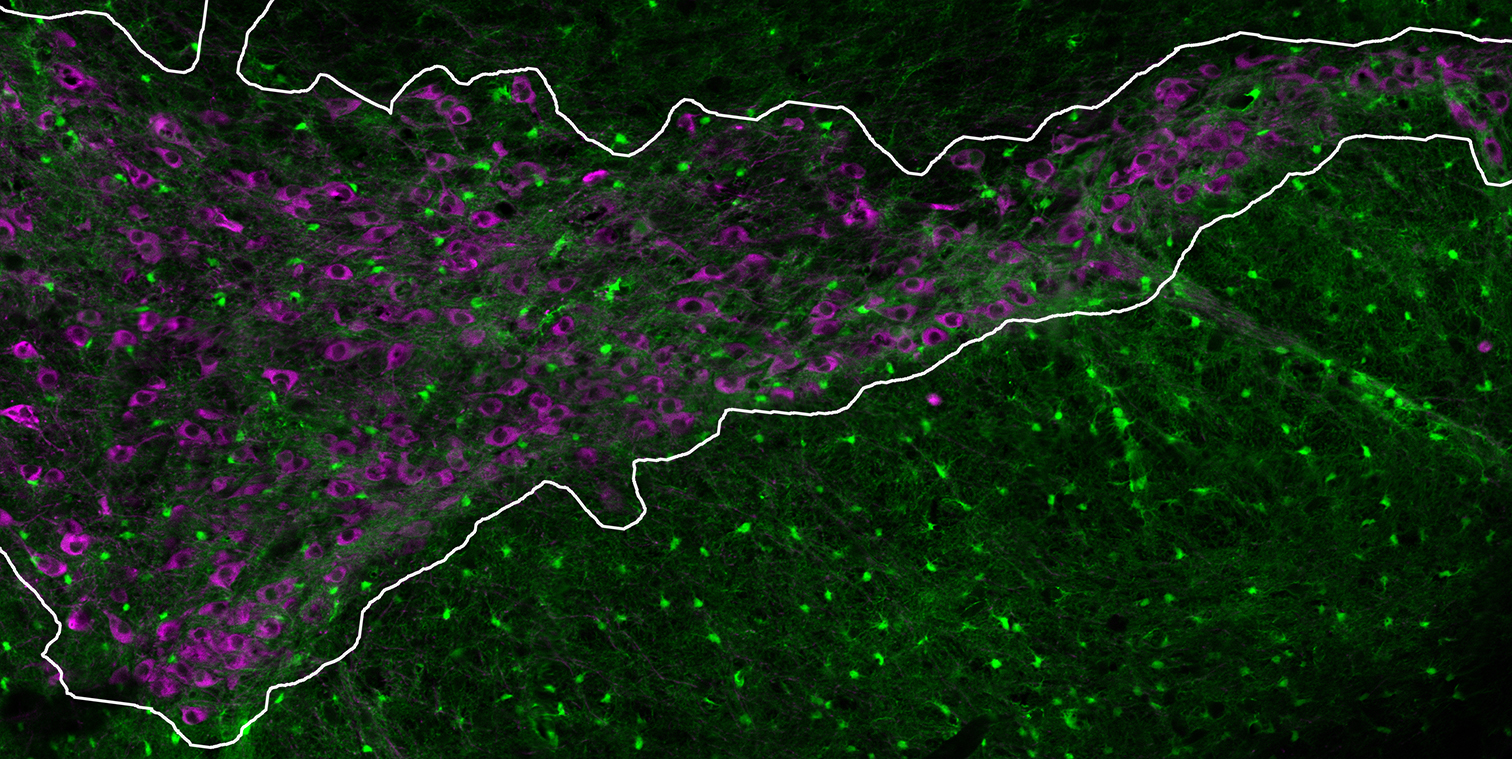

Supplement: Figure 1—source data 2. [file elife-75636-fig1-data2.zip › Fig1 source data 2 for Fig1 D&E/20X/SN shscramble 2M MZ1 GFP+TH.jpg]

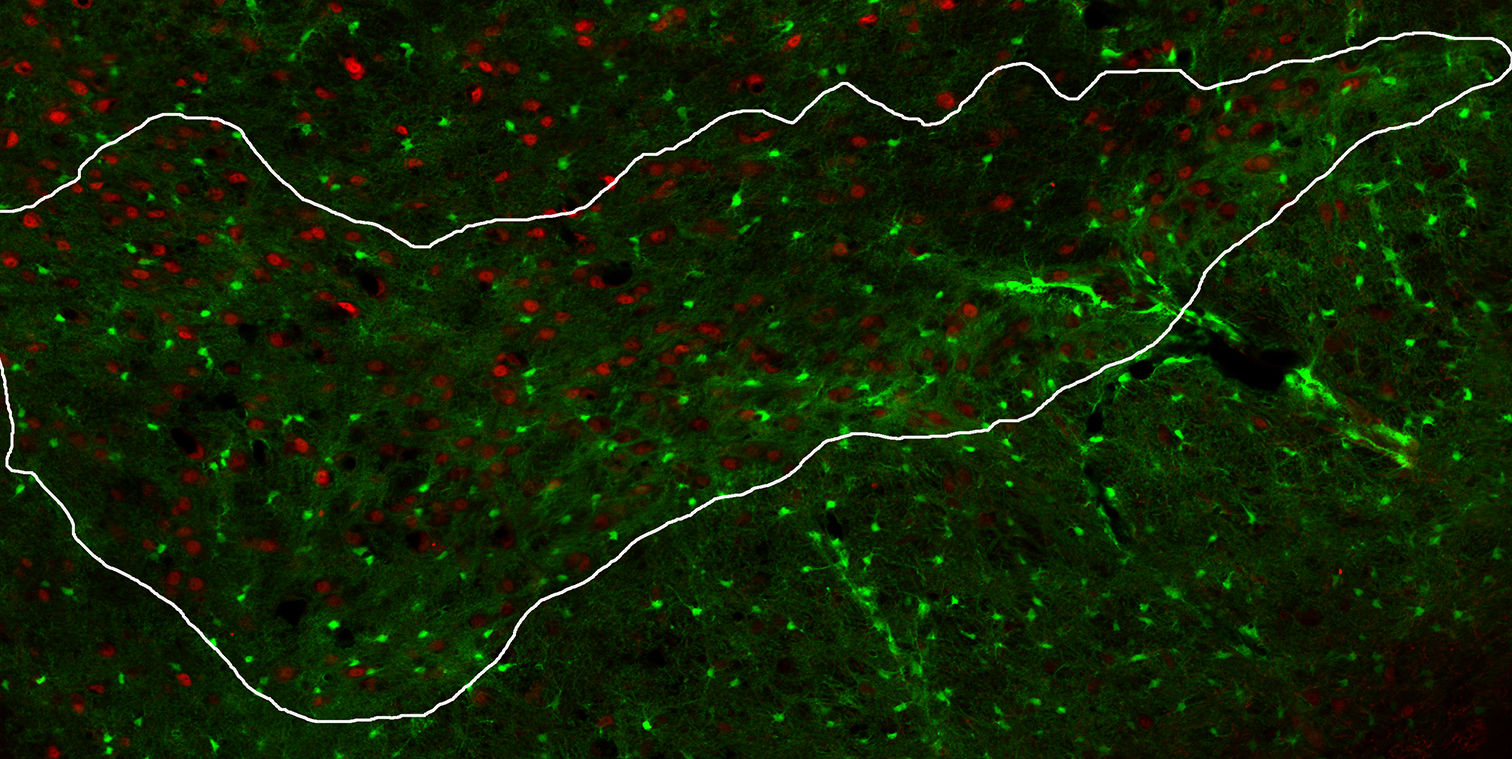

Supplement: Figure 1—source data 2. [file elife-75636-fig1-data2.zip › Fig1 source data 2 for Fig1 D&E/20X/SN shscramble 2M MZ2 GFP+neun.jpg]

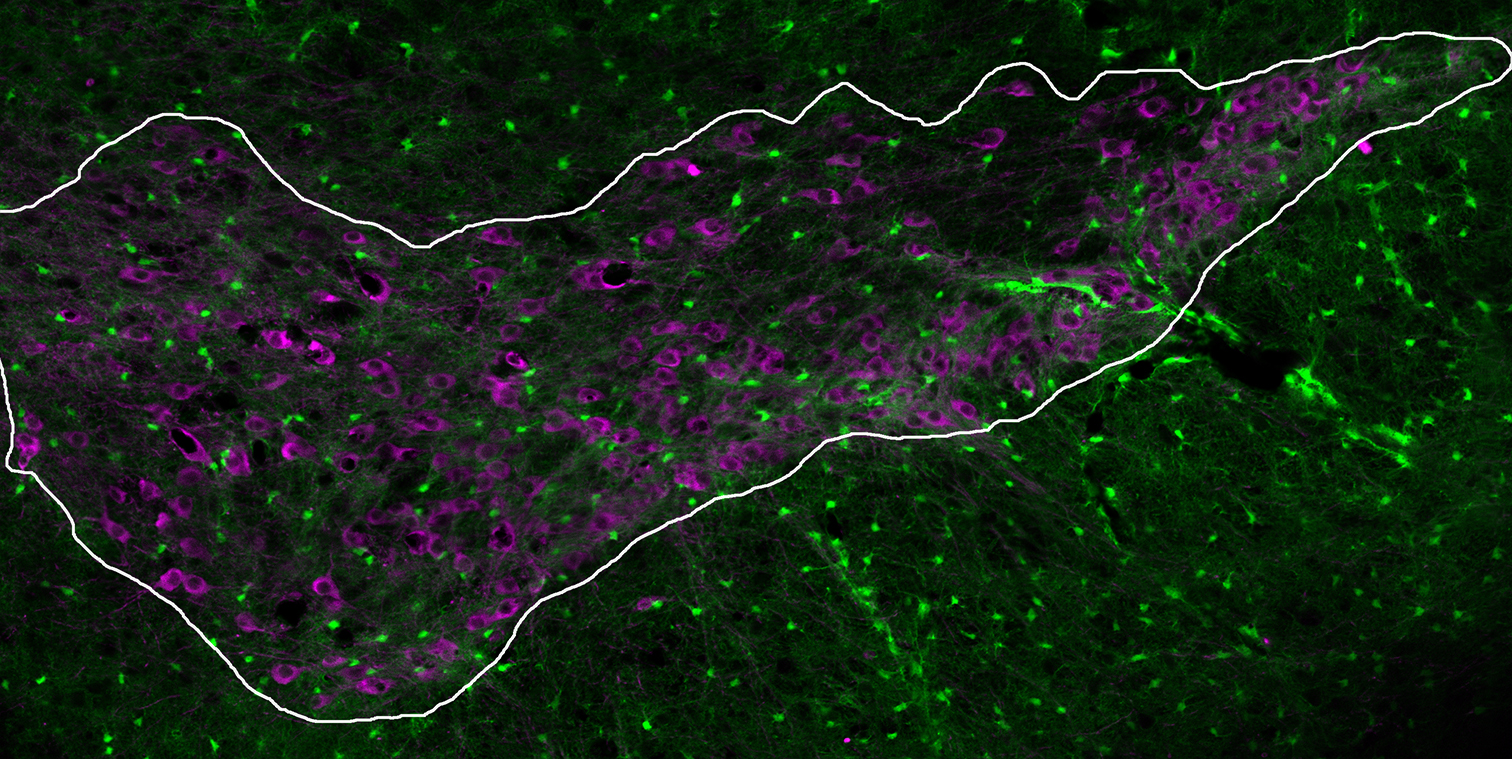

Supplement: Figure 1—source data 2. [file elife-75636-fig1-data2.zip › Fig1 source data 2 for Fig1 D&E/20X/SN shscramble 2M MZ2 GFP+TH.jpg]

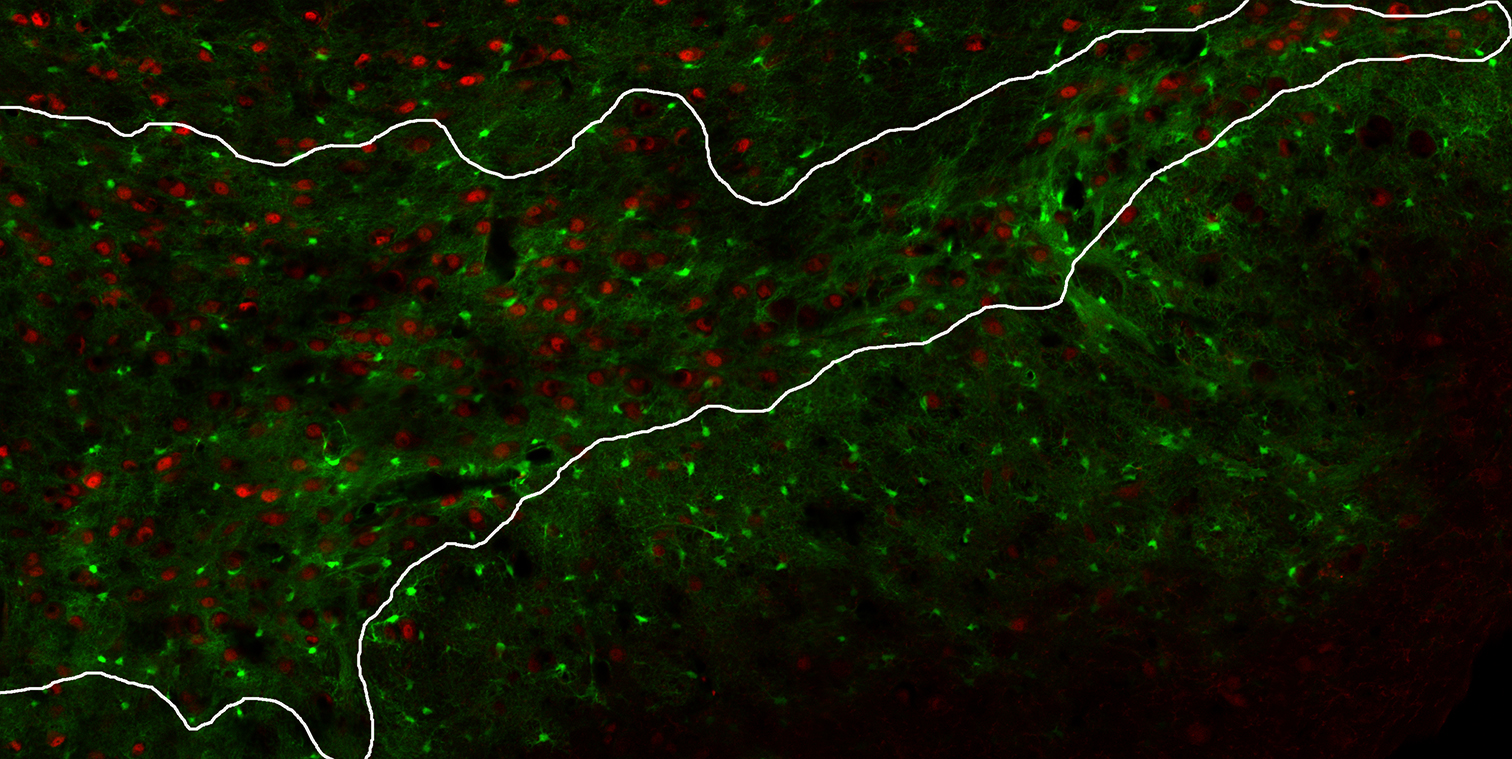

Supplement: Figure 1—source data 2. [file elife-75636-fig1-data2.zip › Fig1 source data 2 for Fig1 D&E/20X/SN shscramble 2M MZ3 GFP+neun.jpg]

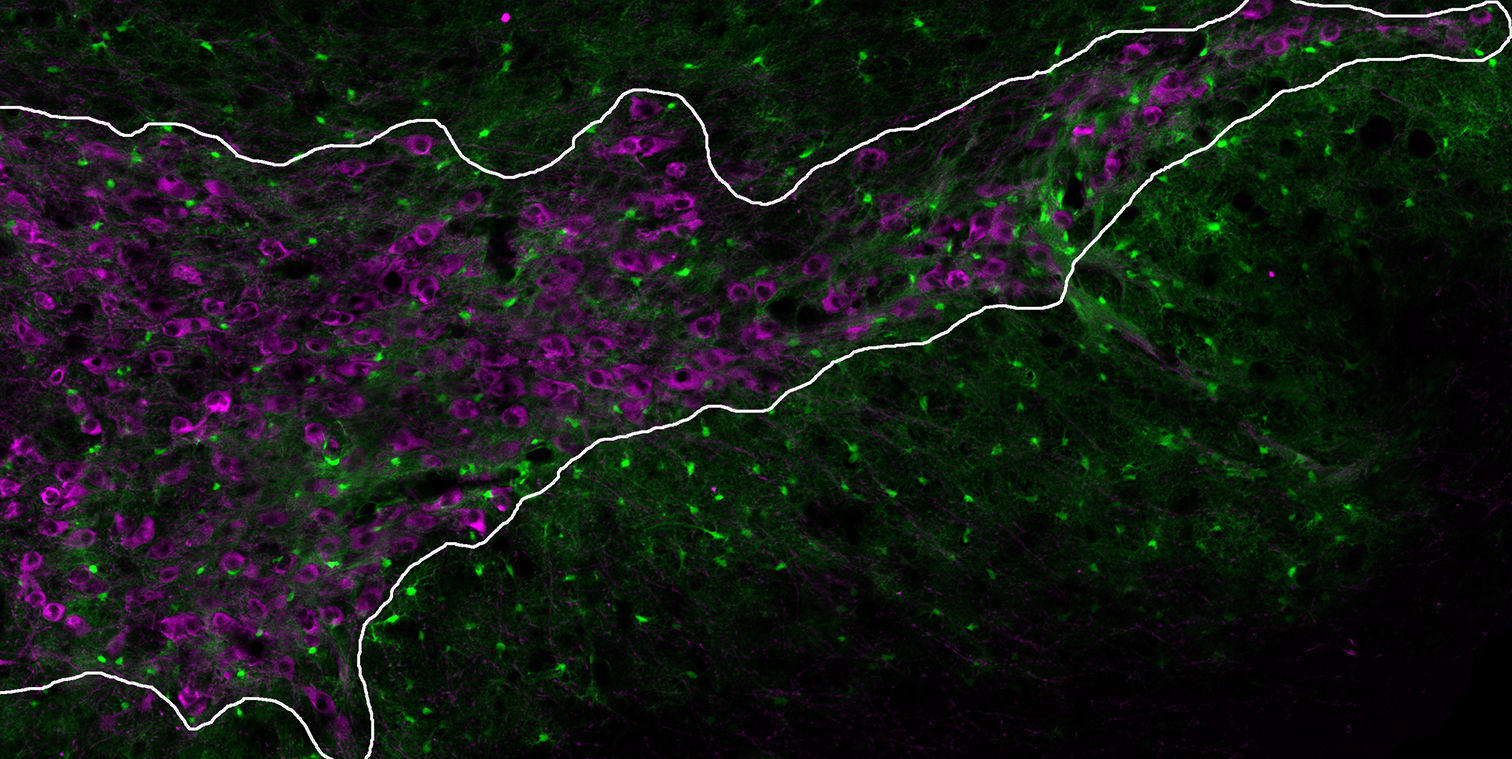

Supplement: Figure 1—source data 2. [file elife-75636-fig1-data2.zip › Fig1 source data 2 for Fig1 D&E/20X/SN shscramble 2M MZ3 GFP+TH.jpg]

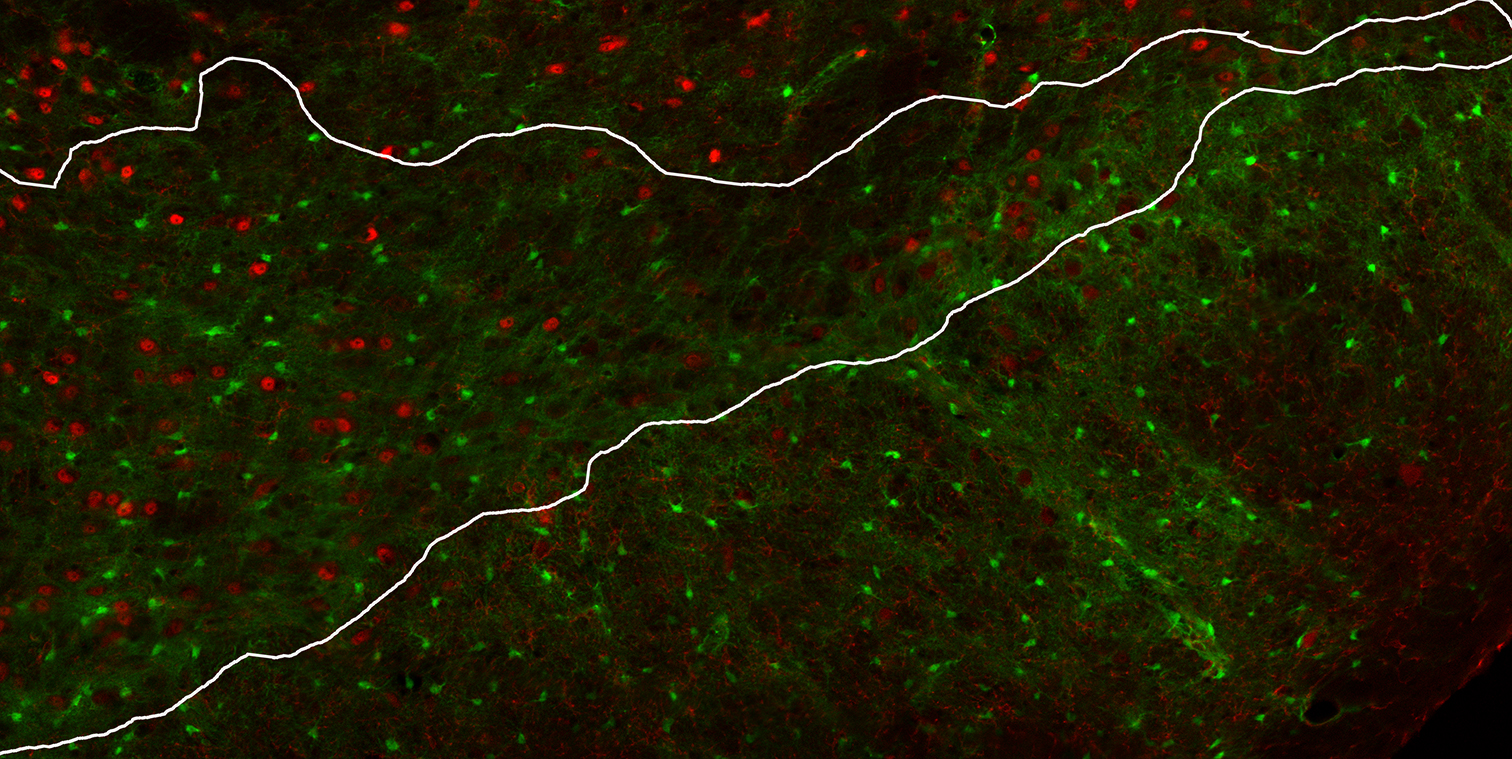

Supplement: Figure 1—source data 2. [file elife-75636-fig1-data2.zip › Fig1 source data 2 for Fig1 D&E/20X/SN shscramble 3M MZ1 GFP+neun.jpg]

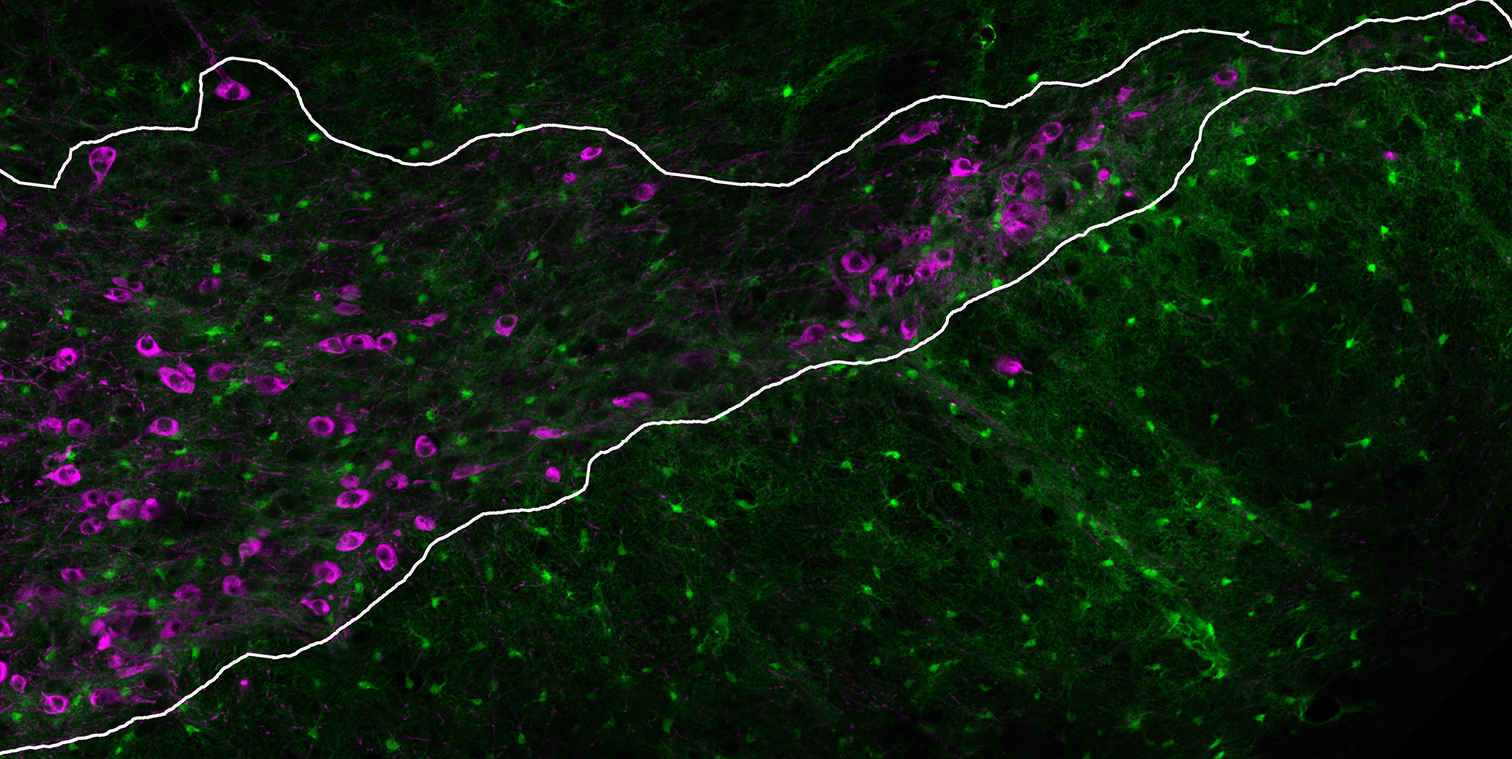

Supplement: Figure 1—source data 2. [file elife-75636-fig1-data2.zip › Fig1 source data 2 for Fig1 D&E/20X/SN shscramble 3M MZ1 GFP+TH.jpg]

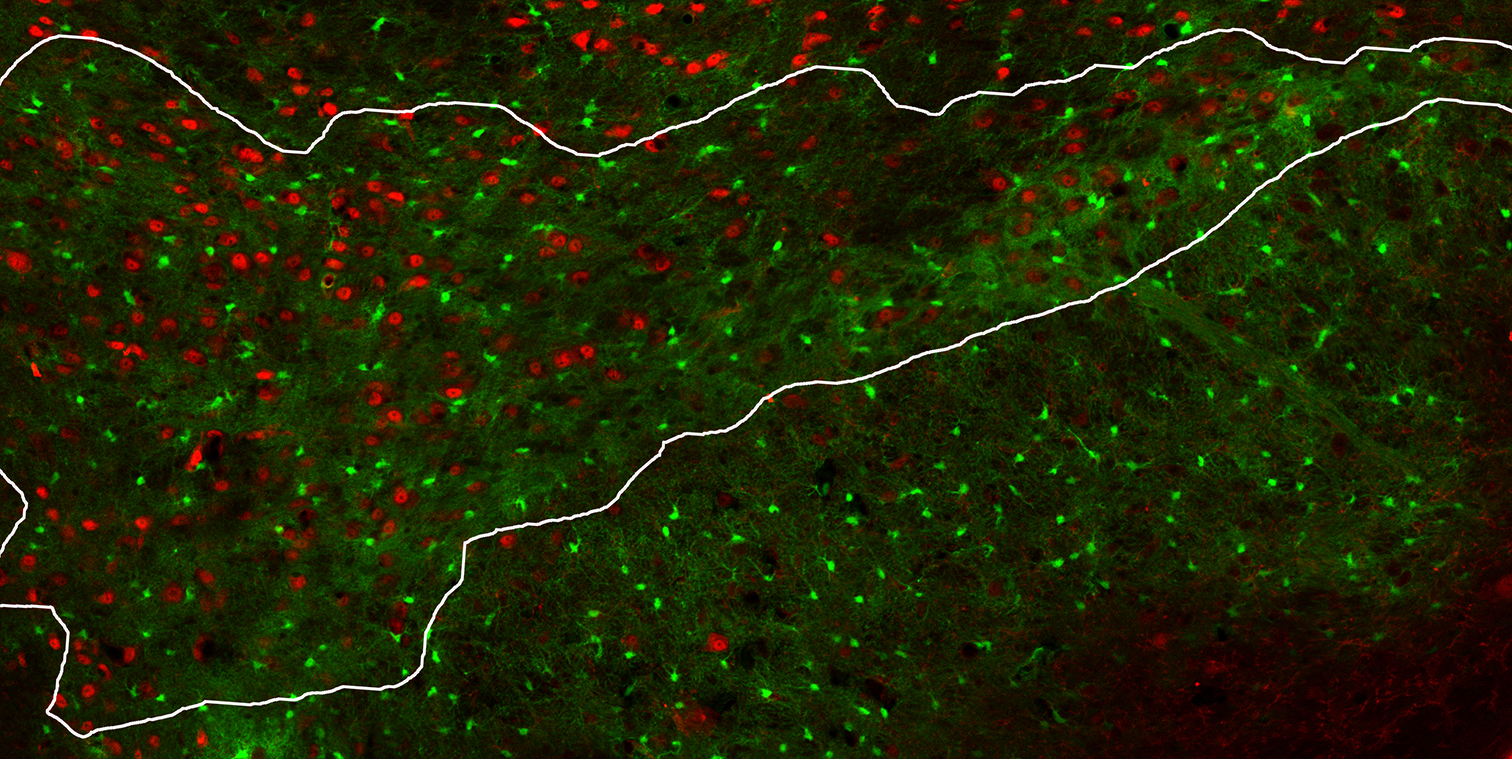

Supplement: Figure 1—source data 2. [file elife-75636-fig1-data2.zip › Fig1 source data 2 for Fig1 D&E/20X/SN shscramble 3M MZ2 GFP+neun.jpg]

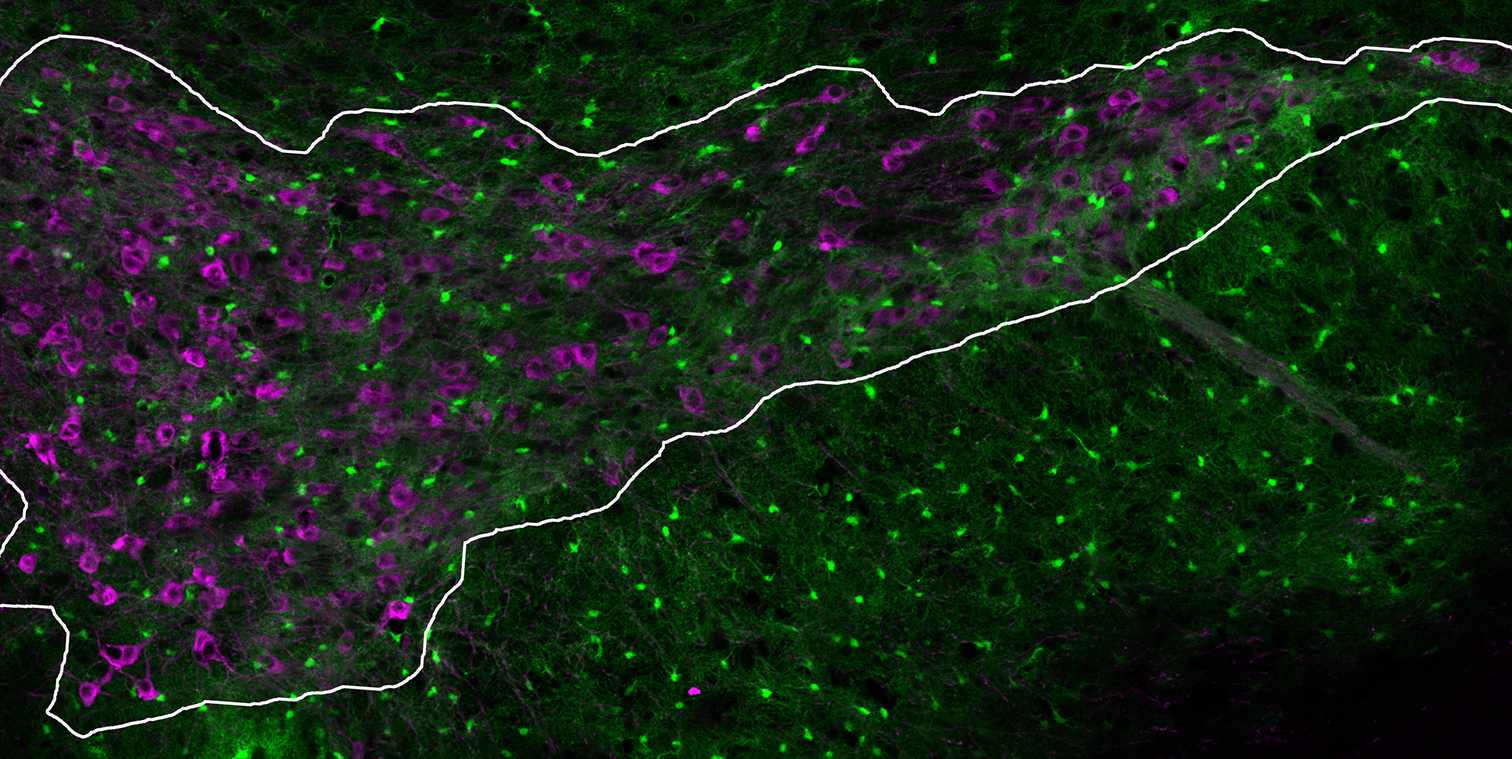

Supplement: Figure 1—source data 2. [file elife-75636-fig1-data2.zip › Fig1 source data 2 for Fig1 D&E/20X/SN shscramble 3M MZ2 GFP+TH.jpg]

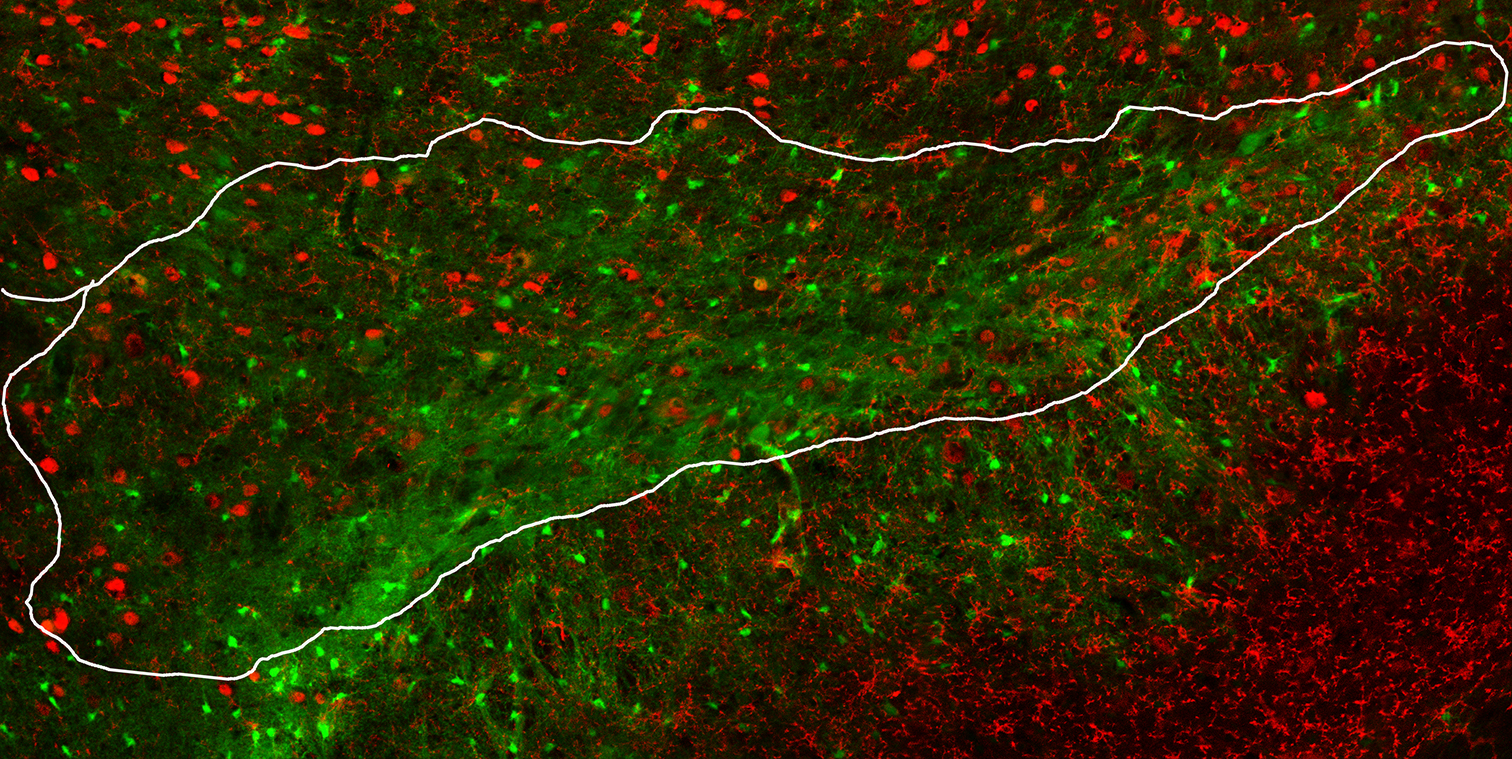

Supplement: Figure 1—source data 2. [file elife-75636-fig1-data2.zip › Fig1 source data 2 for Fig1 D&E/20X/SN shscramble 3M MZ3 GFP+neun.jpg]

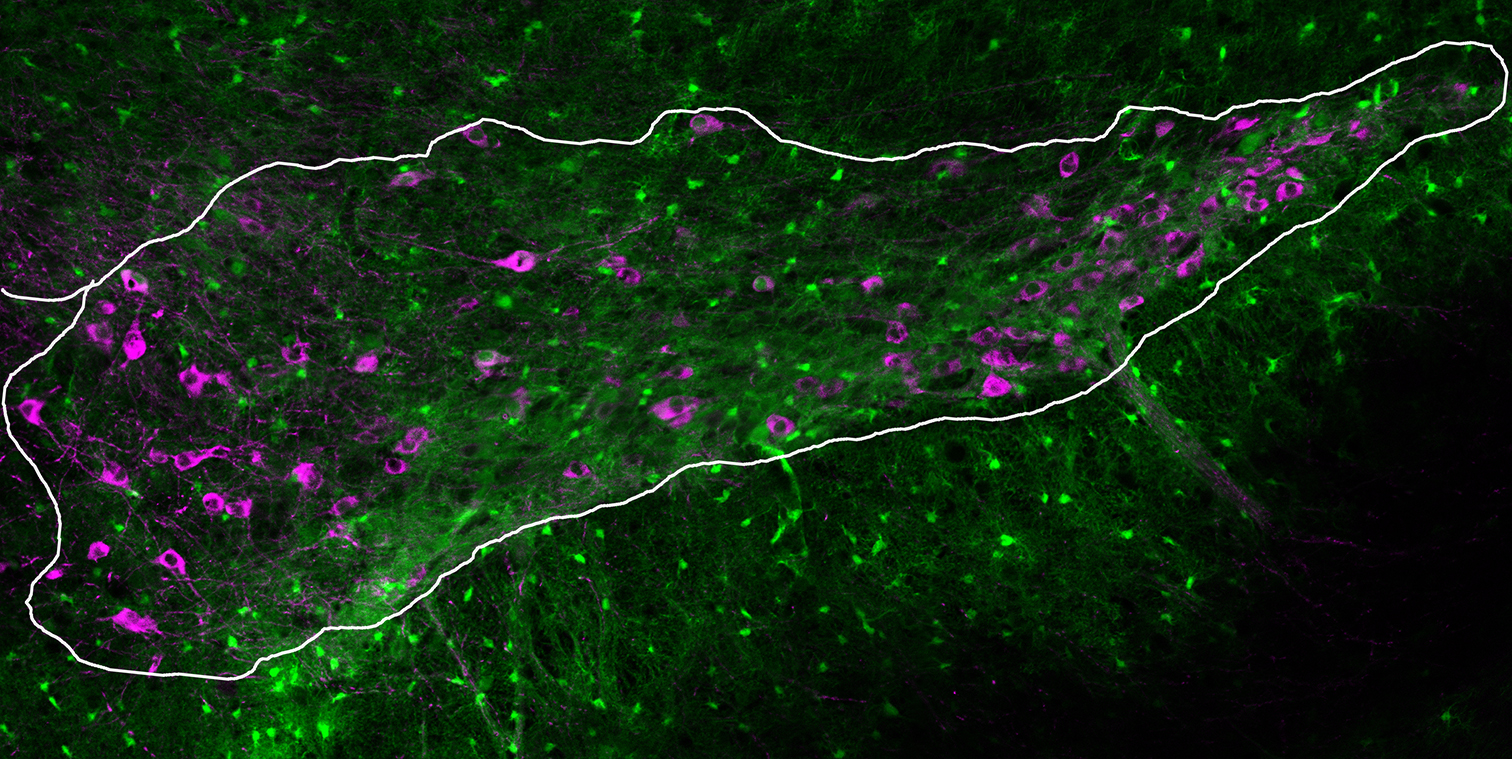

Supplement: Figure 1—source data 2. [file elife-75636-fig1-data2.zip › Fig1 source data 2 for Fig1 D&E/20X/SN shscramble 3M MZ3 GFP+TH.jpg]

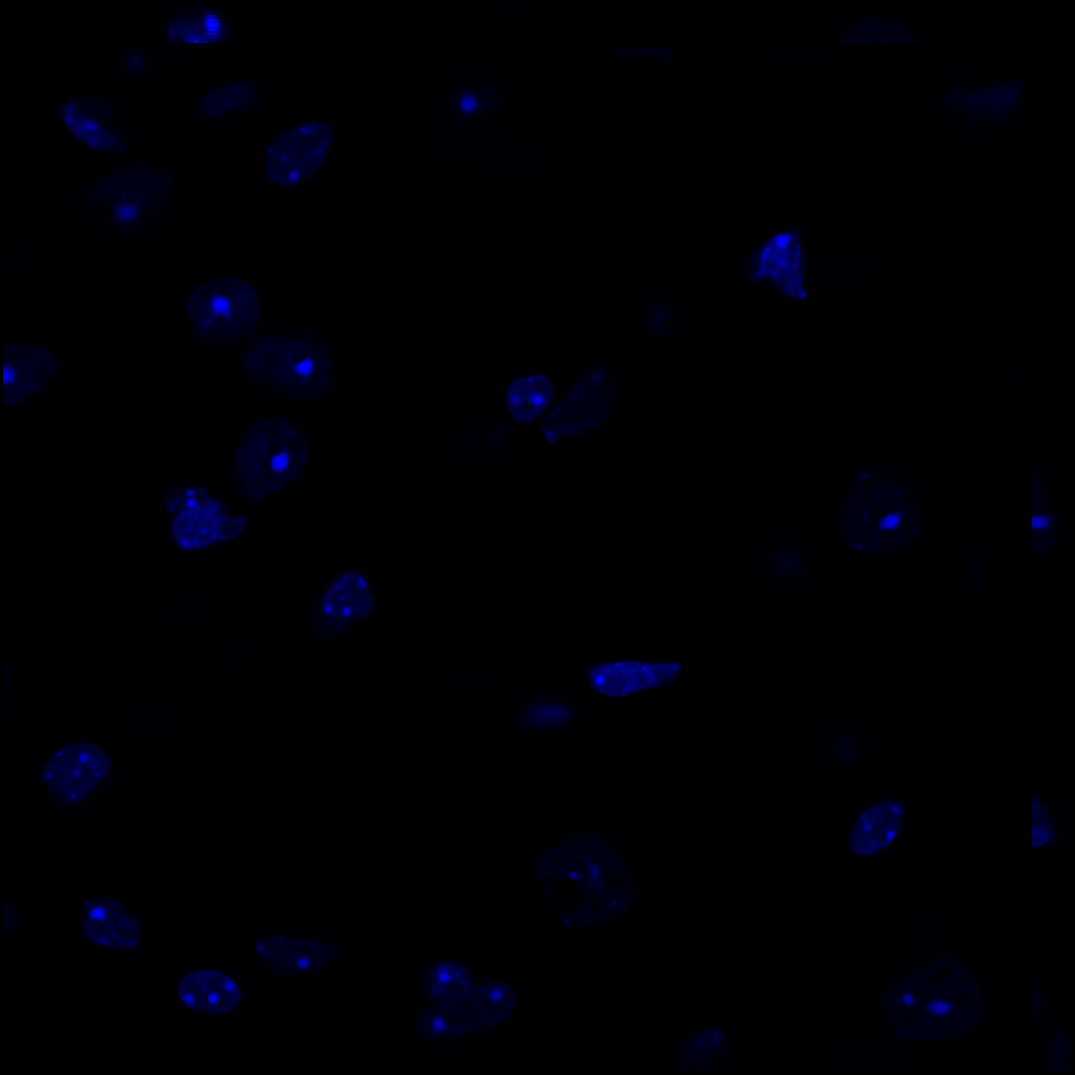

Supplement: Figure 1—source data 2. [file elife-75636-fig1-data2.zip › Fig1 source data 2 for Fig1 D&E/3D/SN 1M MZ2-3/Untitled10_c1.tif]

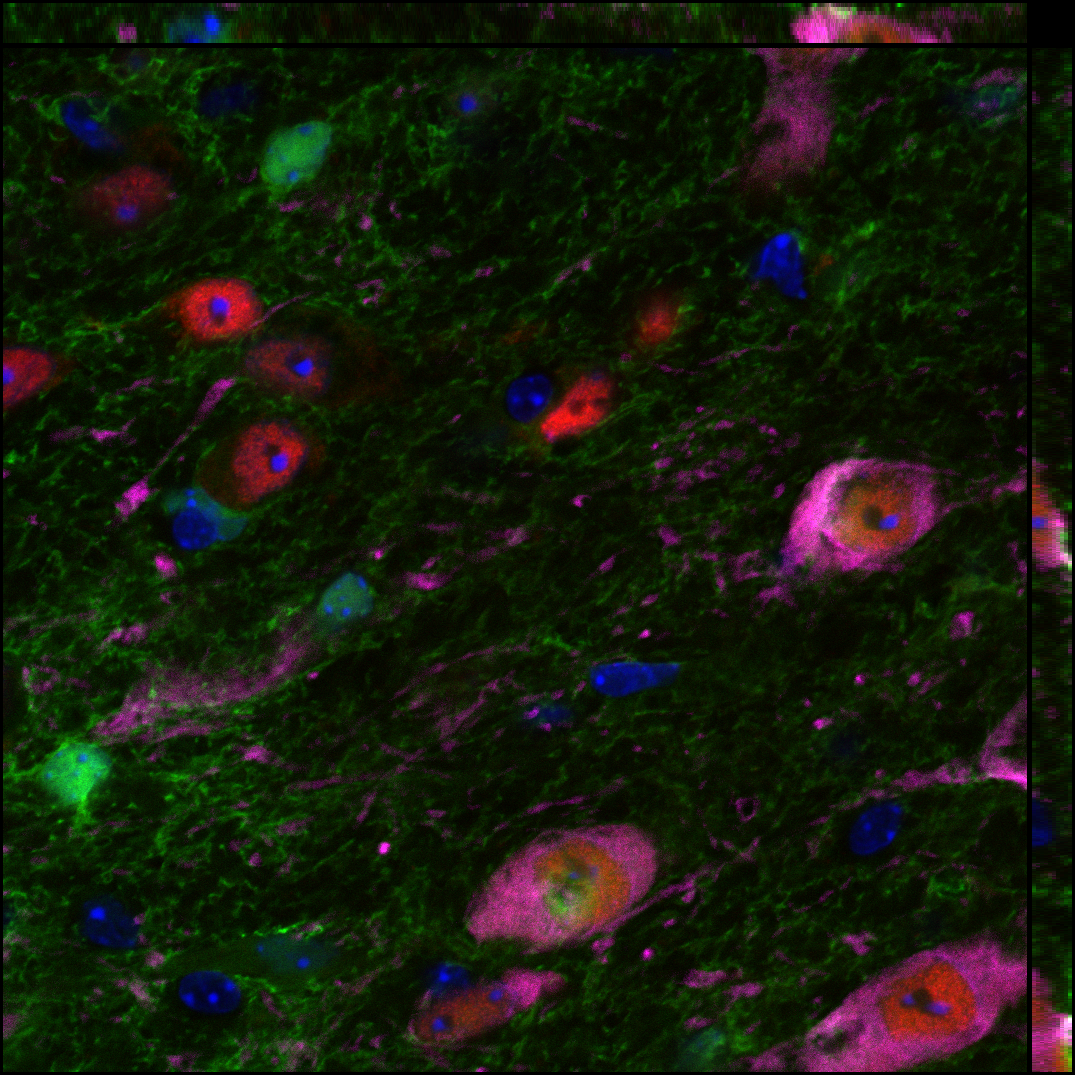

Supplement: Figure 1—source data 2. [file elife-75636-fig1-data2.zip › Fig1 source data 2 for Fig1 D&E/3D/SN 1M MZ2-3/Untitled10_c1+2+3+4.tif]

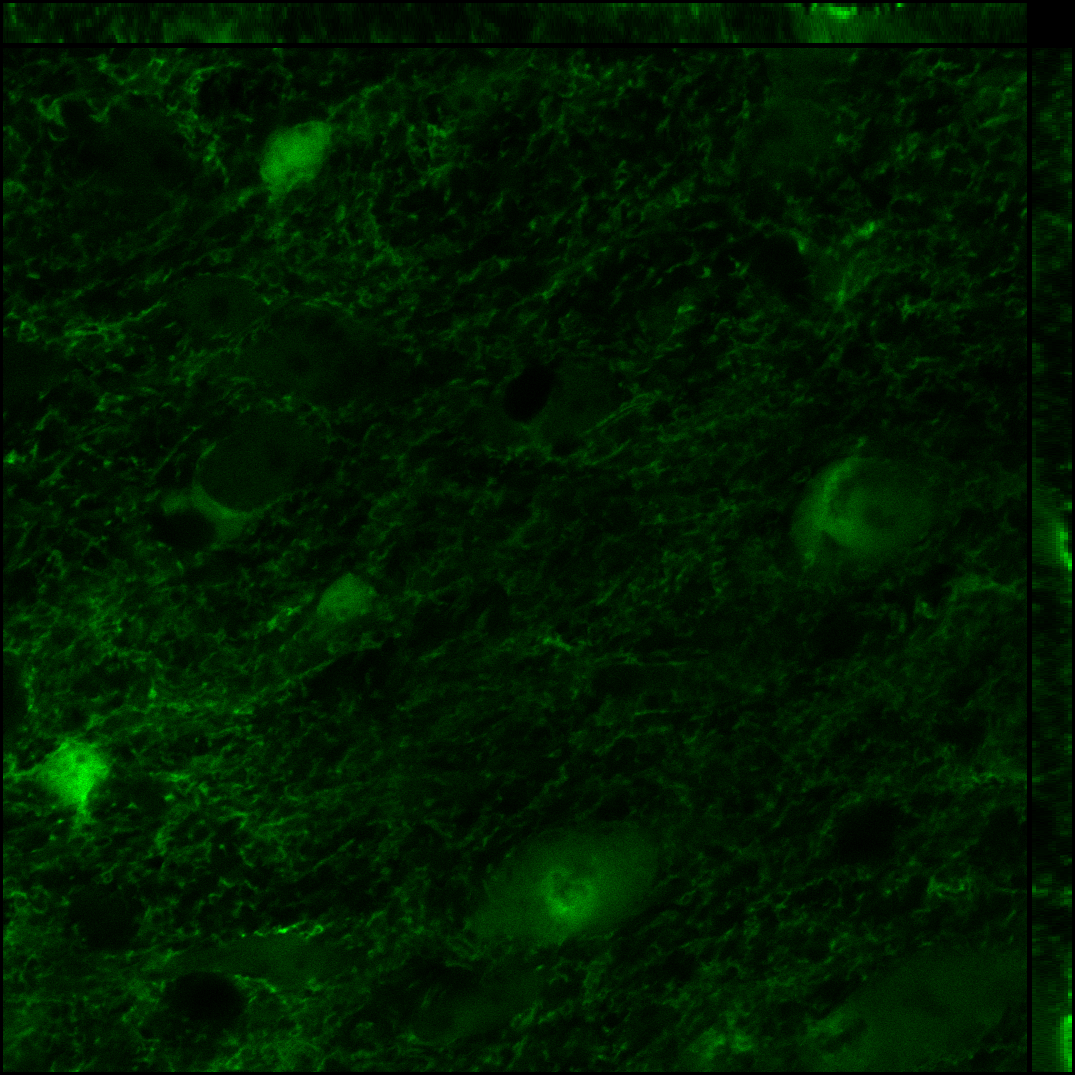

Supplement: Figure 1—source data 2. [file elife-75636-fig1-data2.zip › Fig1 source data 2 for Fig1 D&E/3D/SN 1M MZ2-3/Untitled10_c2.tif]

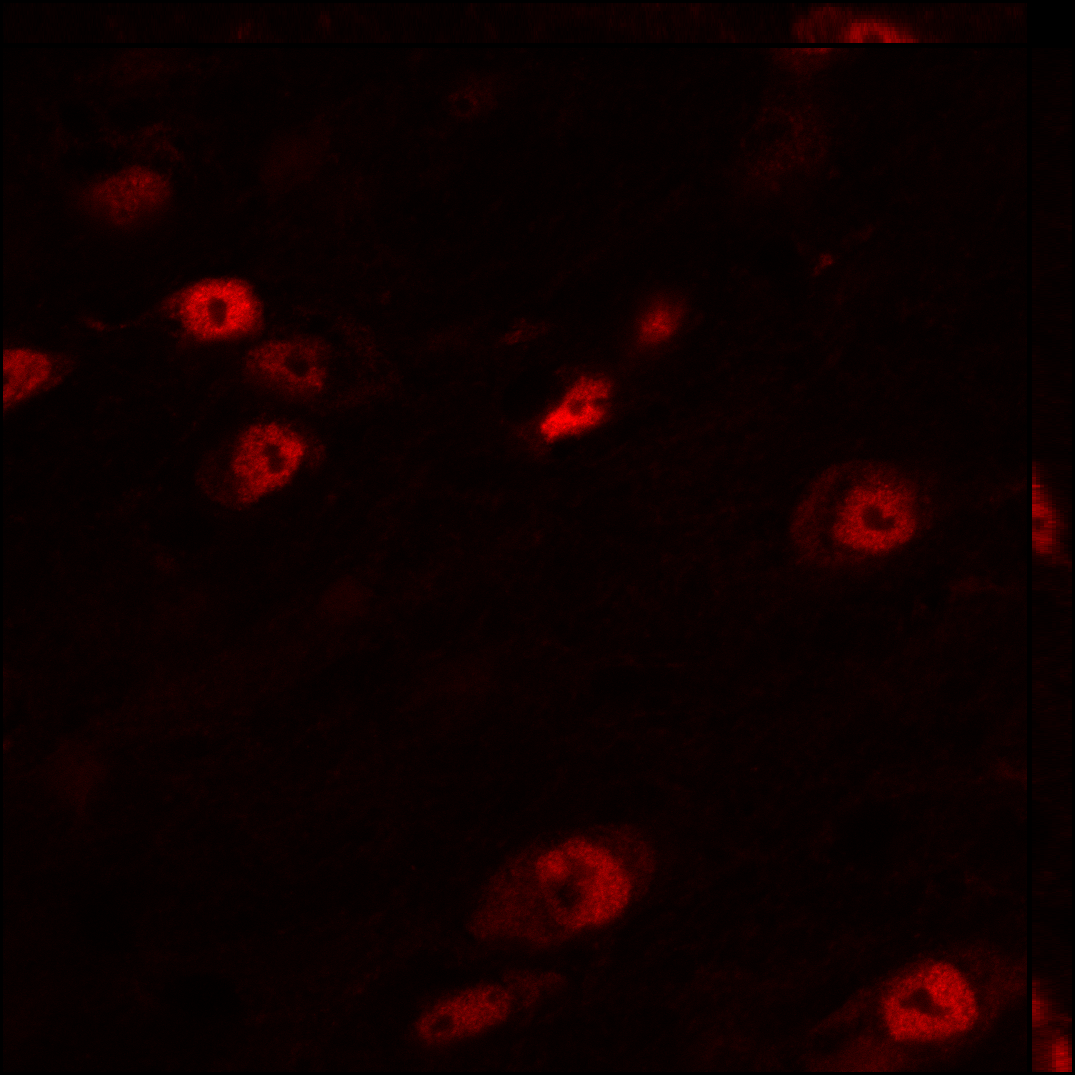

Supplement: Figure 1—source data 2. [file elife-75636-fig1-data2.zip › Fig1 source data 2 for Fig1 D&E/3D/SN 1M MZ2-3/Untitled10_c3.tif]

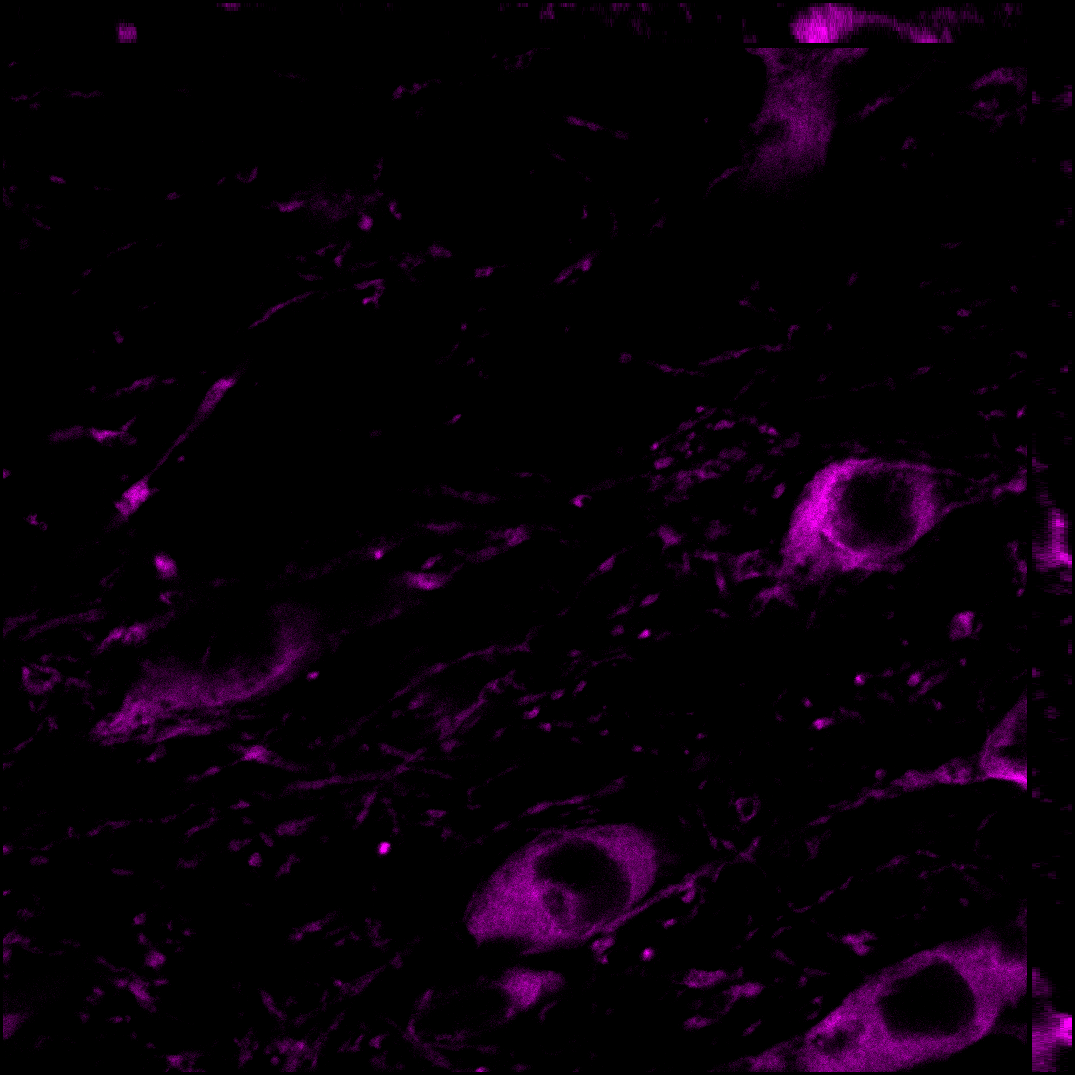

Supplement: Figure 1—source data 2. [file elife-75636-fig1-data2.zip › Fig1 source data 2 for Fig1 D&E/3D/SN 1M MZ2-3/Untitled10_c4.tif]

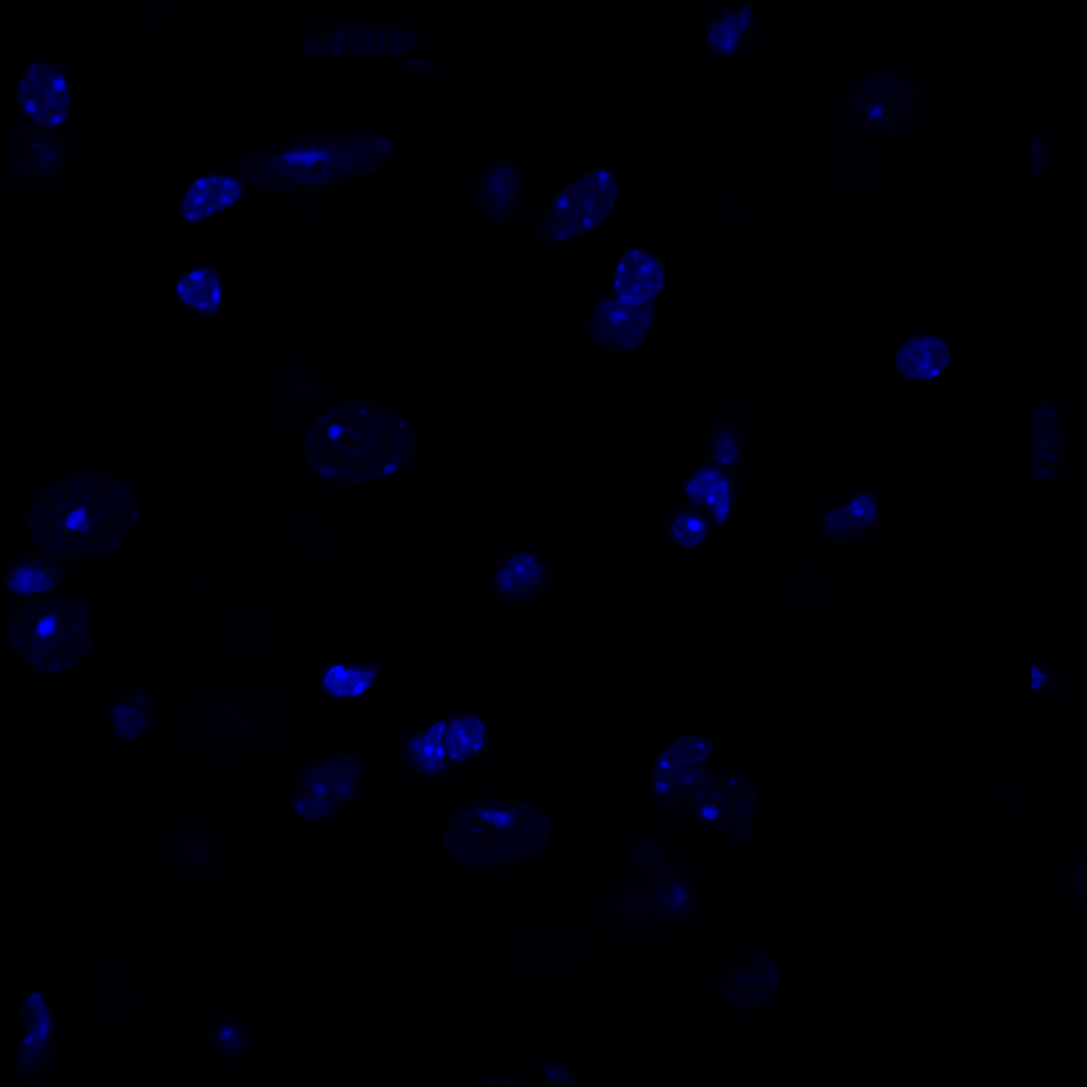

Supplement: Figure 1—source data 2. [file elife-75636-fig1-data2.zip › Fig1 source data 2 for Fig1 D&E/3D/SN 2M MZ3-1/Untitled17_c1.tif]

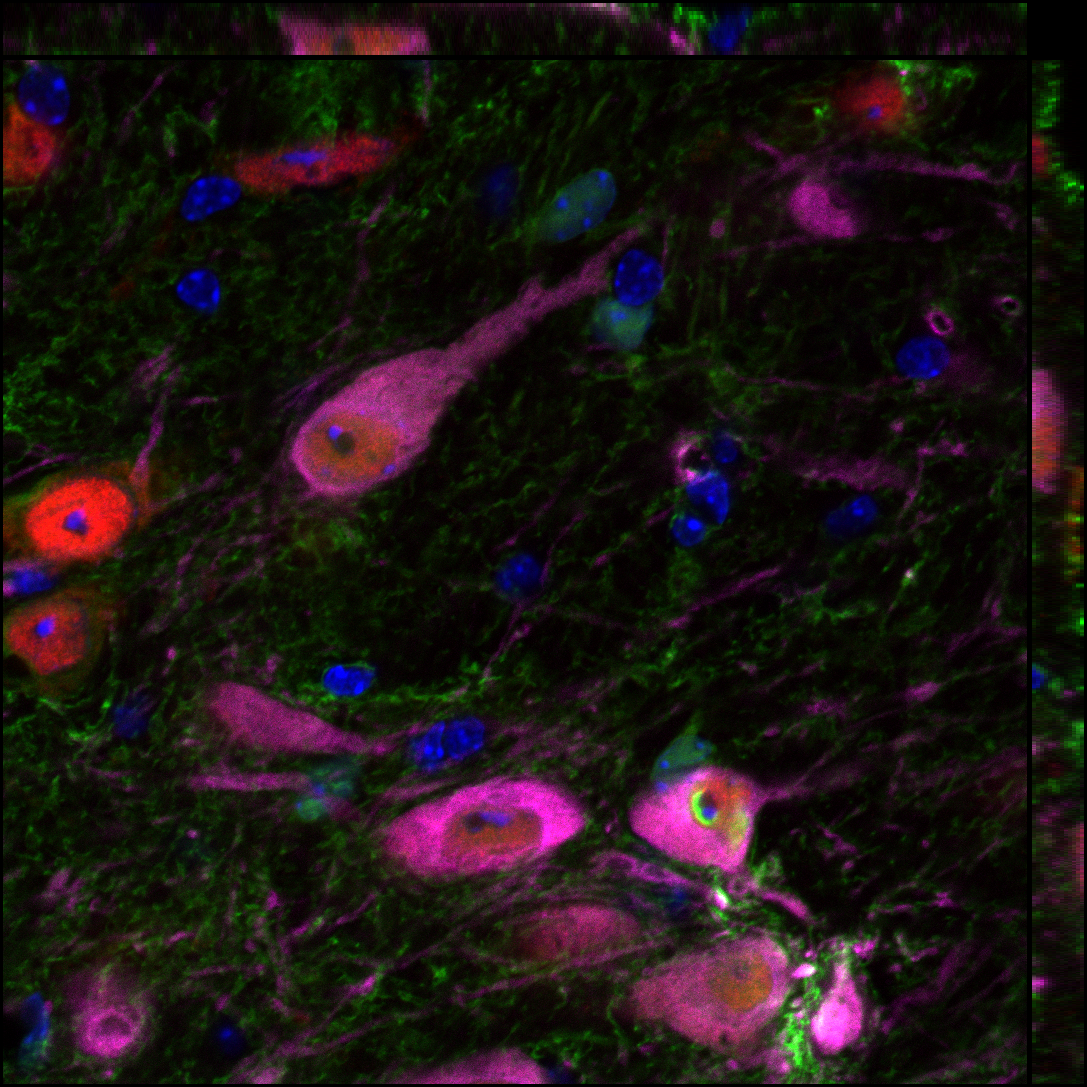

Supplement: Figure 1—source data 2. [file elife-75636-fig1-data2.zip › Fig1 source data 2 for Fig1 D&E/3D/SN 2M MZ3-1/Untitled17_c1+2+3+4.tif]

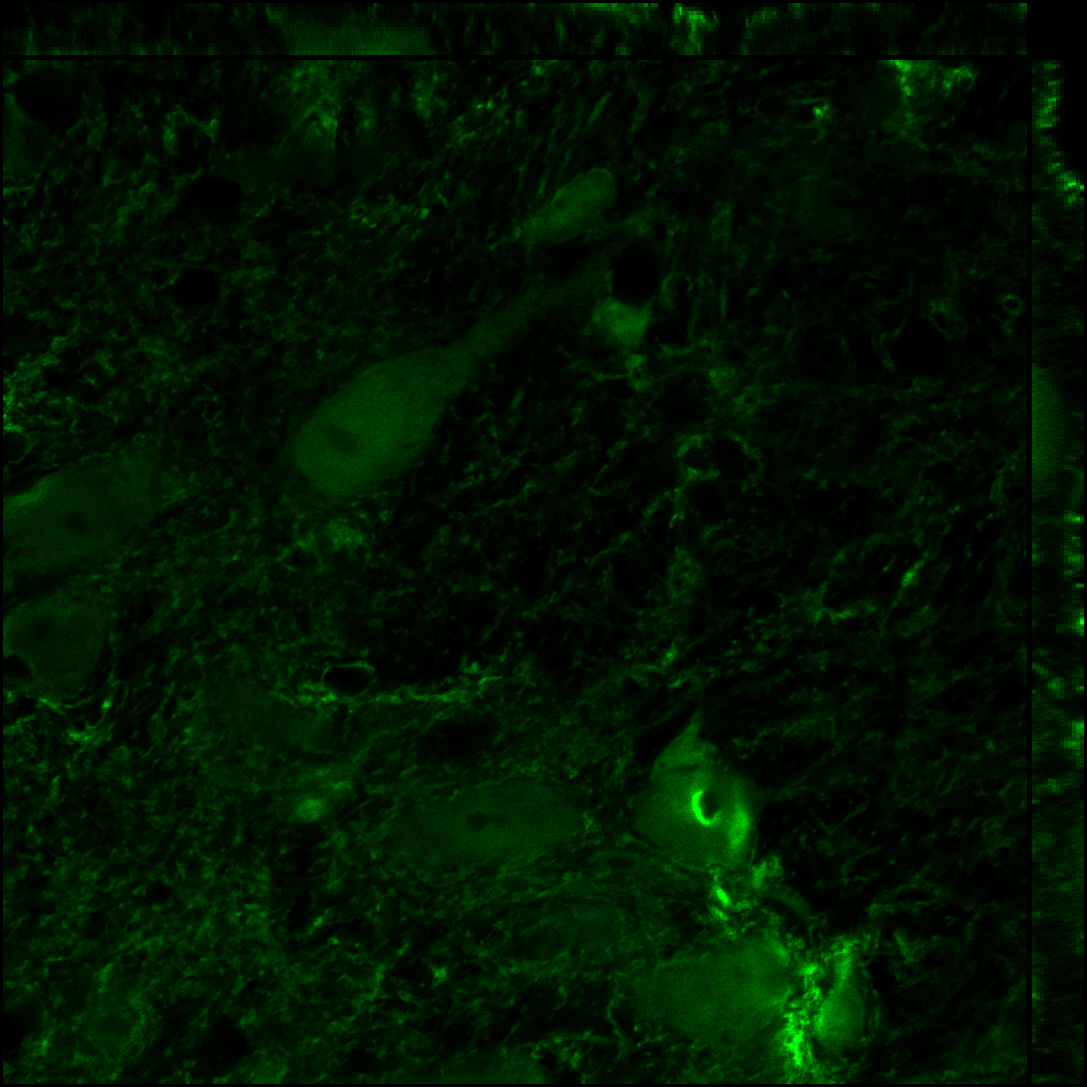

Supplement: Figure 1—source data 2. [file elife-75636-fig1-data2.zip › Fig1 source data 2 for Fig1 D&E/3D/SN 2M MZ3-1/Untitled17_c2.tif]

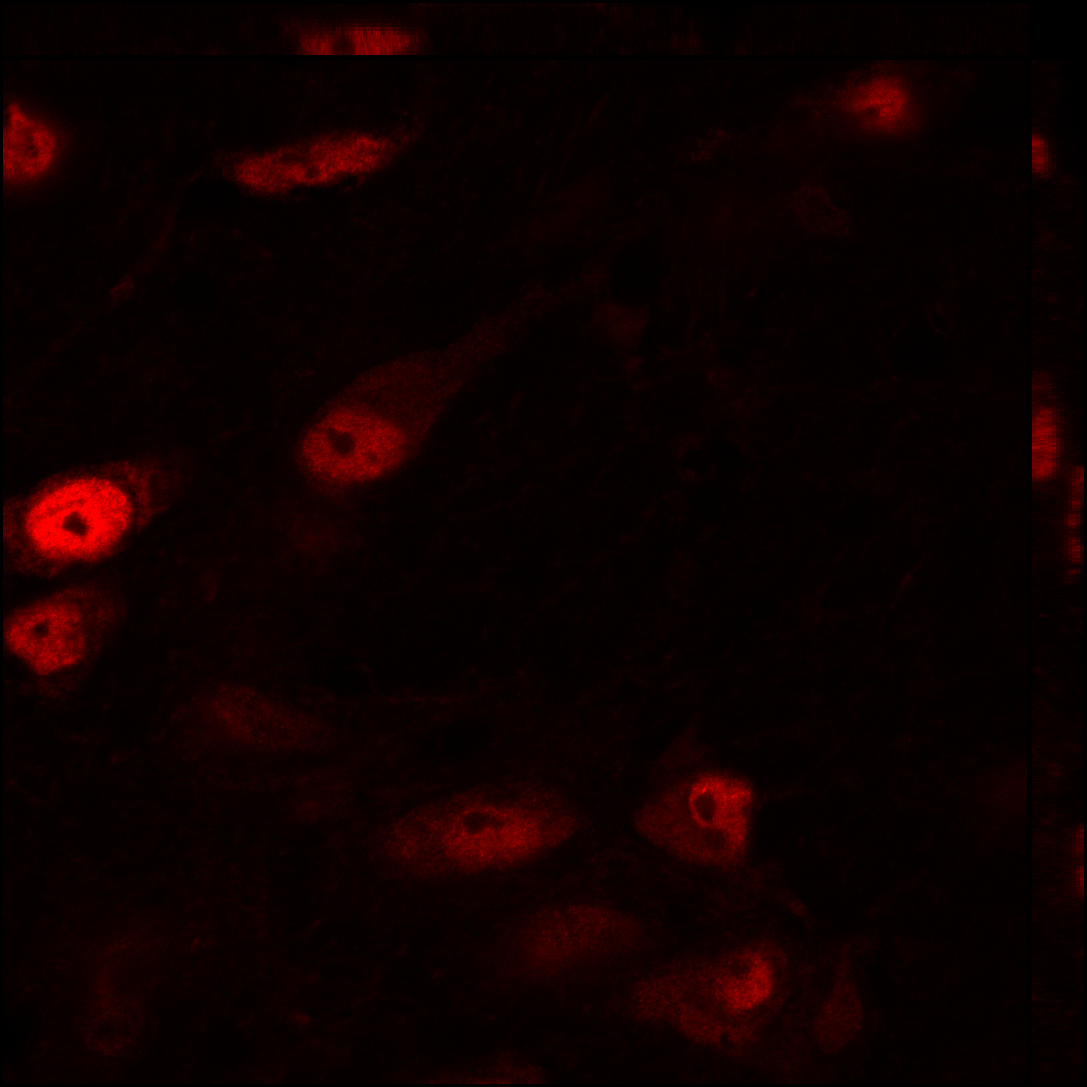

Supplement: Figure 1—source data 2. [file elife-75636-fig1-data2.zip › Fig1 source data 2 for Fig1 D&E/3D/SN 2M MZ3-1/Untitled17_c3.tif]

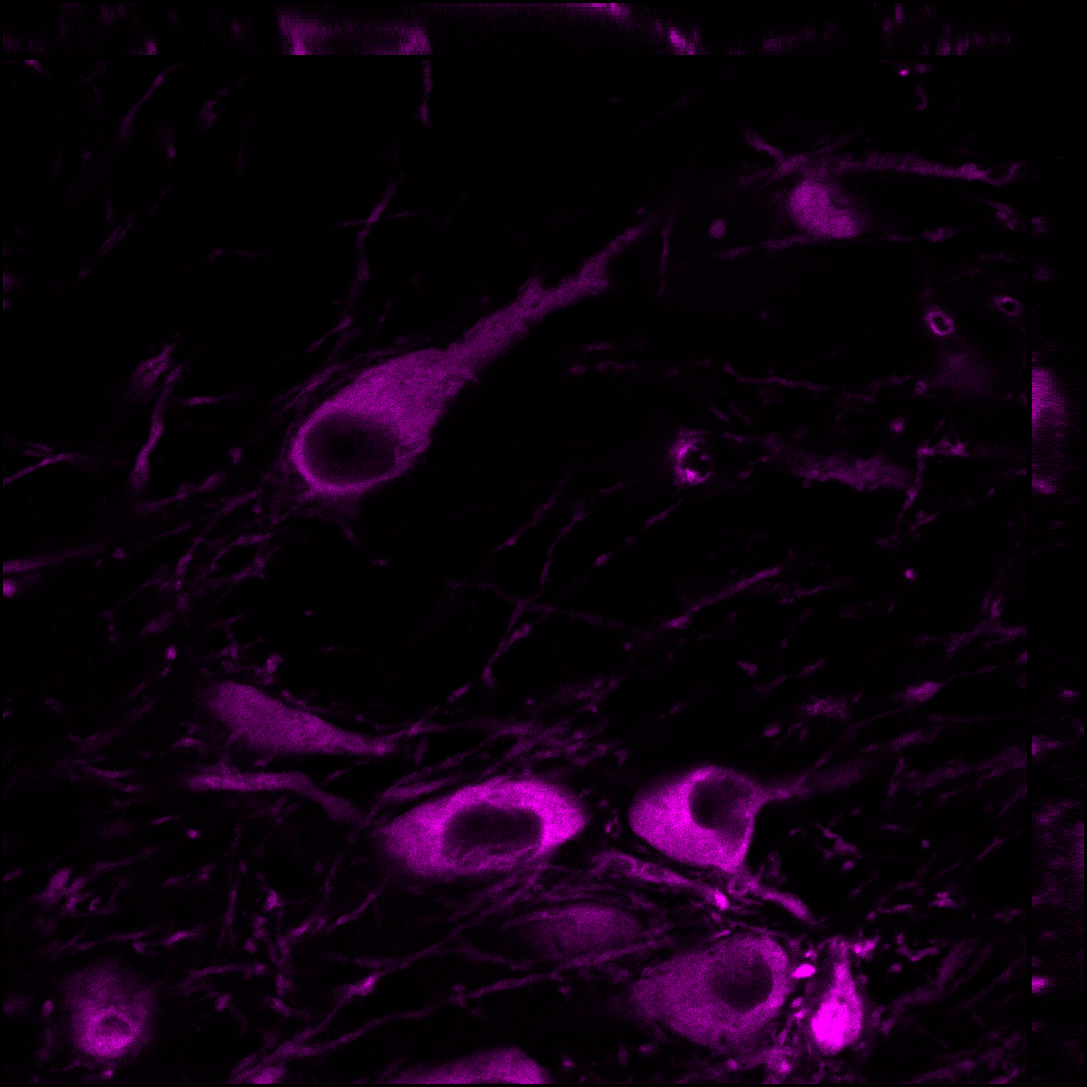

Supplement: Figure 1—source data 2. [file elife-75636-fig1-data2.zip › Fig1 source data 2 for Fig1 D&E/3D/SN 2M MZ3-1/Untitled17_c4.tif]

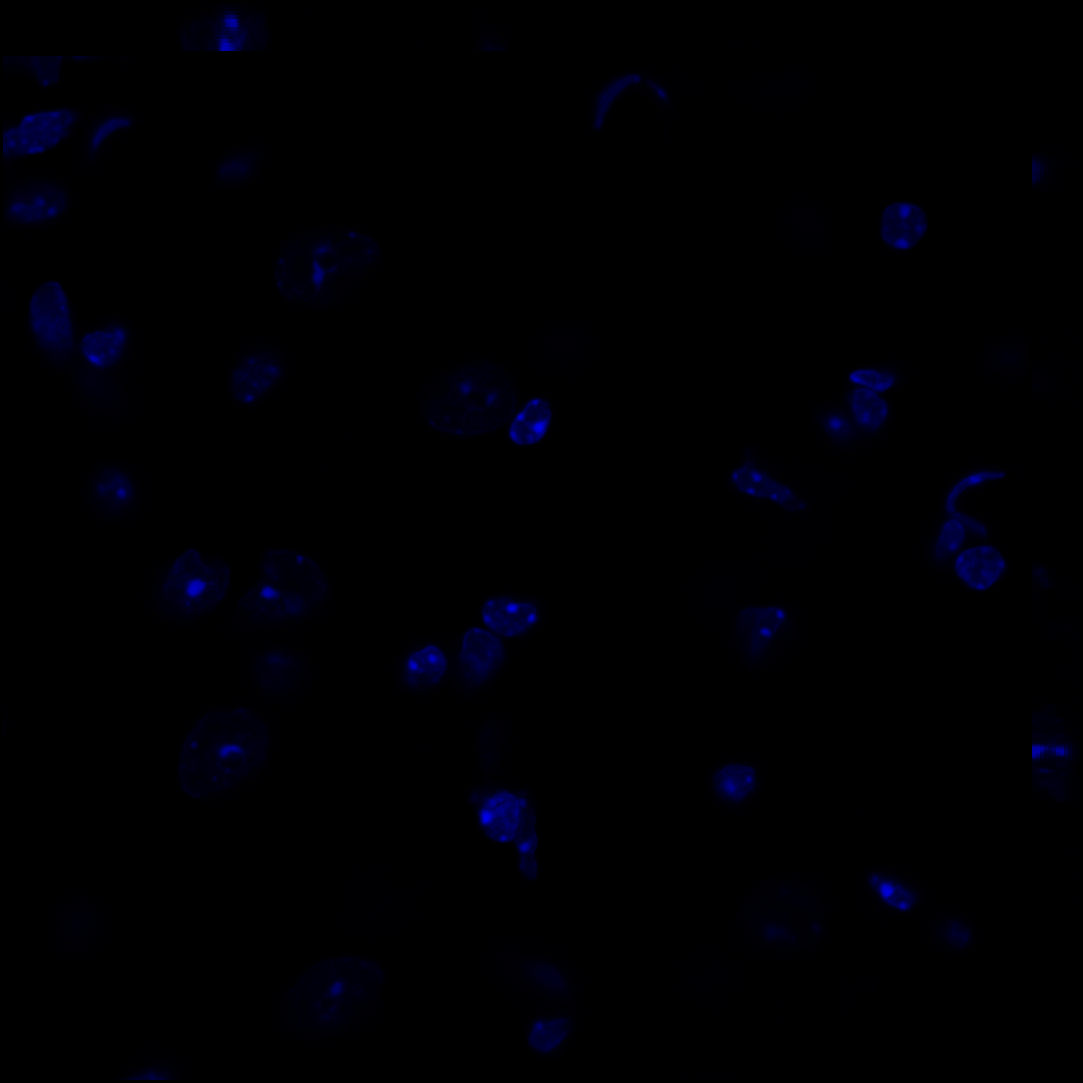

Supplement: Figure 1—source data 2. [file elife-75636-fig1-data2.zip › Fig1 source data 2 for Fig1 D&E/3D/SN 3M MZ2-1/Untitled19_c1.tif]

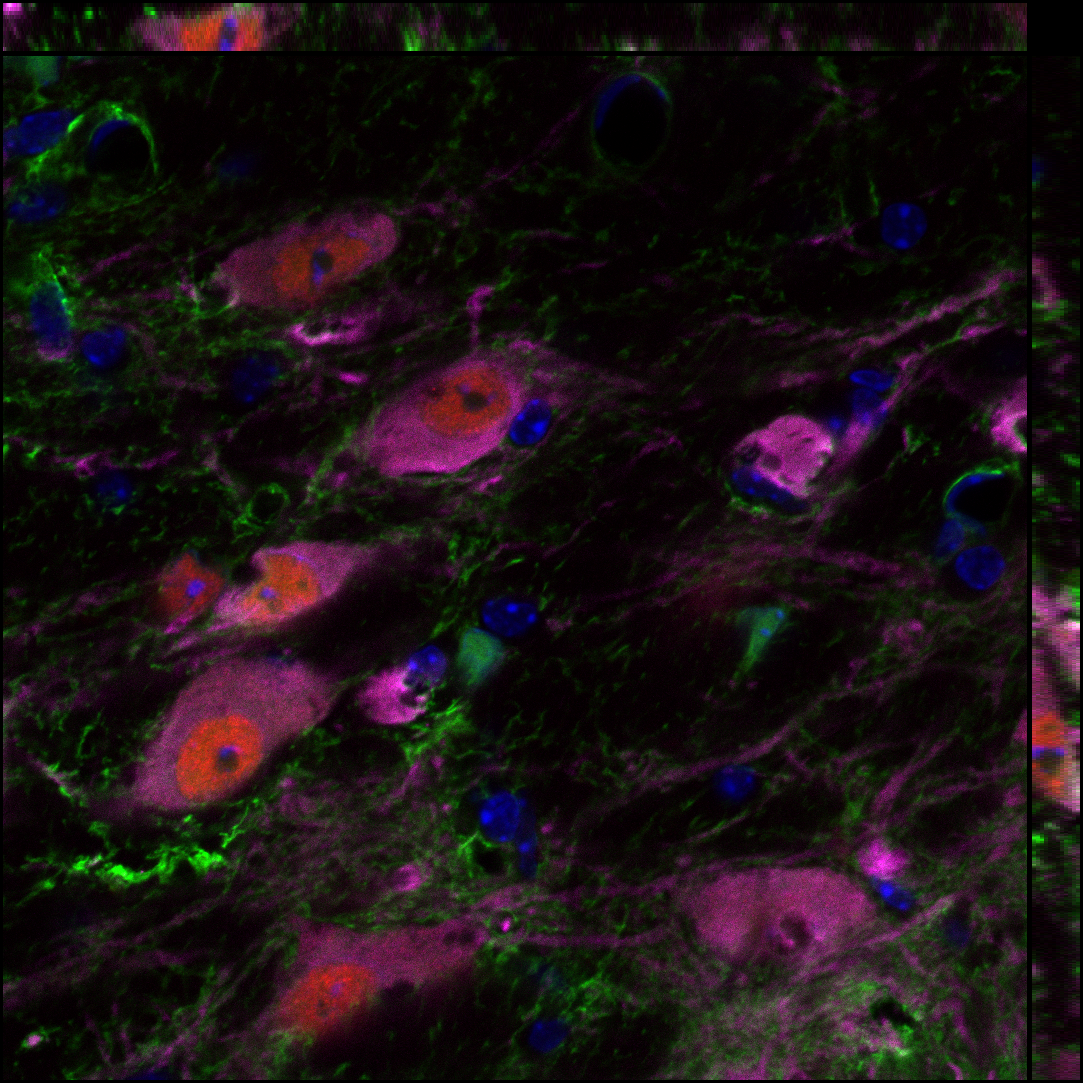

Supplement: Figure 1—source data 2. [file elife-75636-fig1-data2.zip › Fig1 source data 2 for Fig1 D&E/3D/SN 3M MZ2-1/Untitled19_c1+2+3+4.tif]

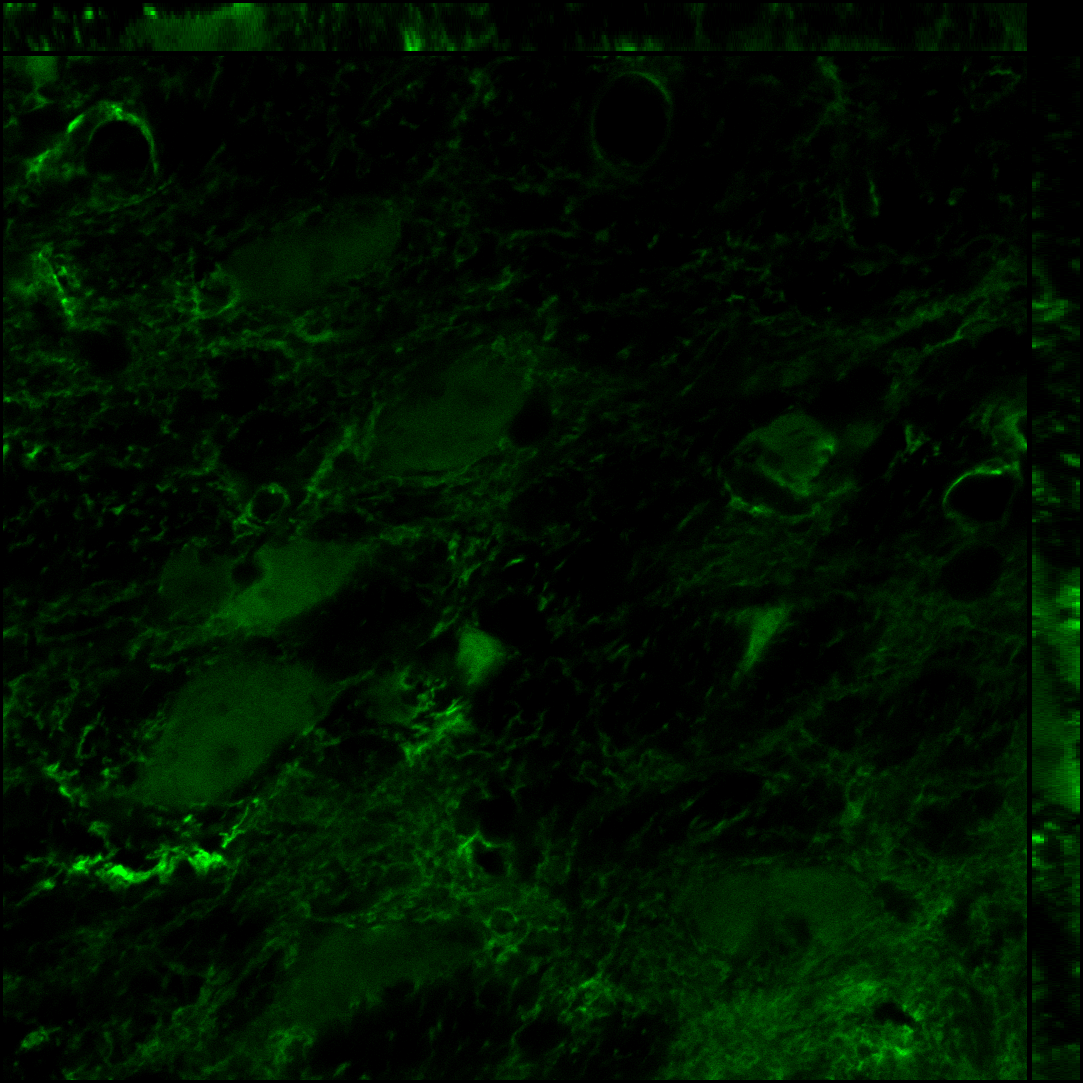

Supplement: Figure 1—source data 2. [file elife-75636-fig1-data2.zip › Fig1 source data 2 for Fig1 D&E/3D/SN 3M MZ2-1/Untitled19_c2.tif]

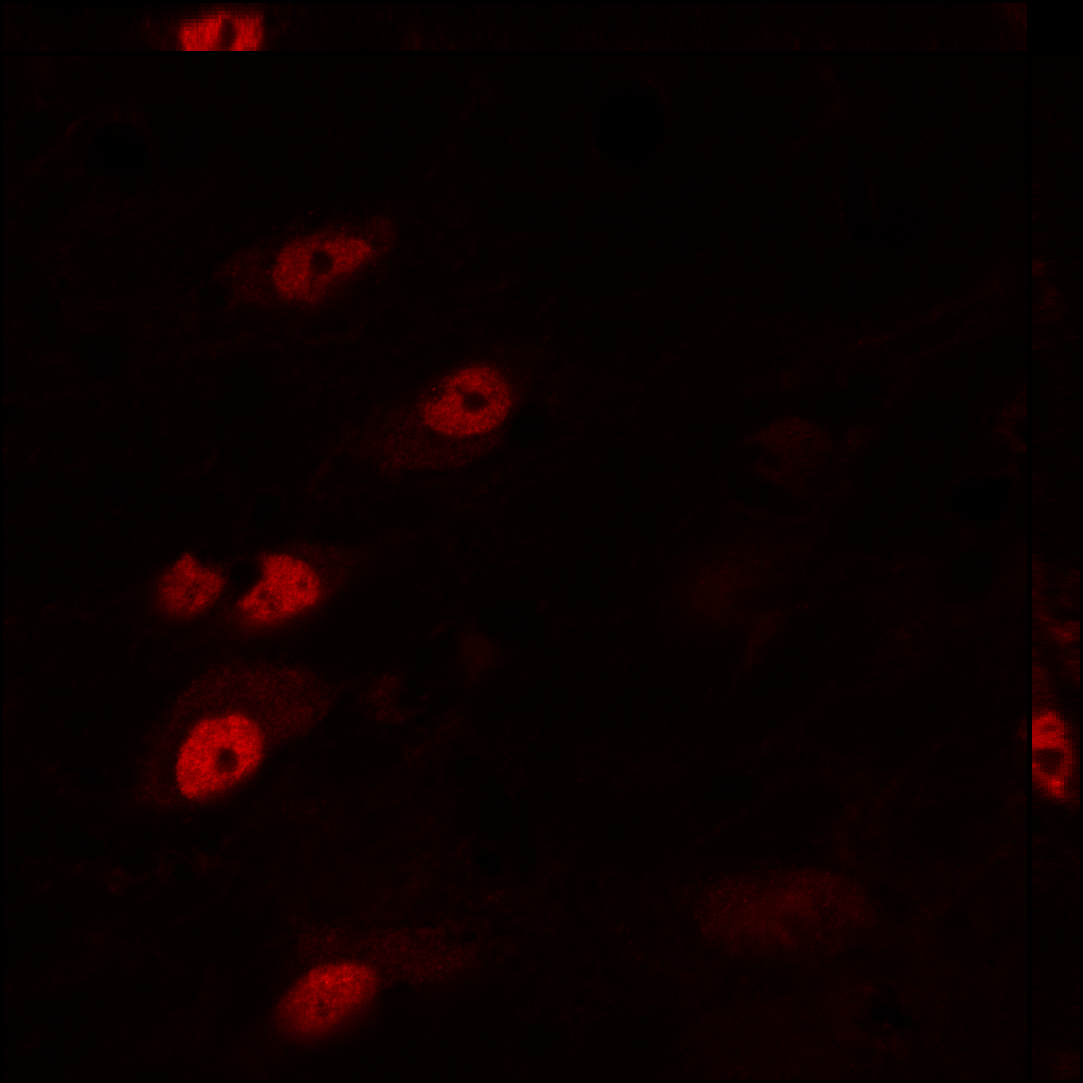

Supplement: Figure 1—source data 2. [file elife-75636-fig1-data2.zip › Fig1 source data 2 for Fig1 D&E/3D/SN 3M MZ2-1/Untitled19_c3.tif]

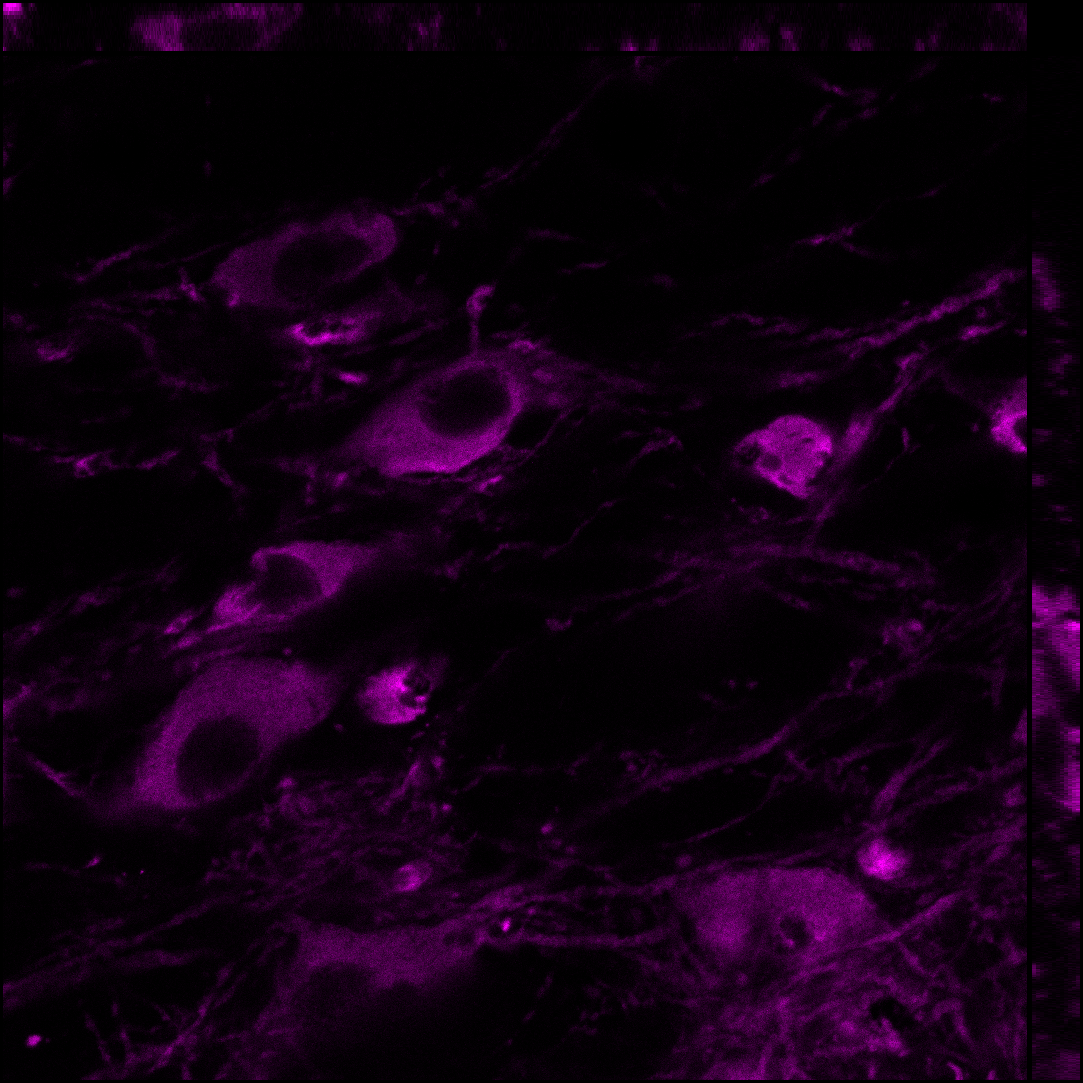

Supplement: Figure 1—source data 2. [file elife-75636-fig1-data2.zip › Fig1 source data 2 for Fig1 D&E/3D/SN 3M MZ2-1/Untitled19_c4.tif]

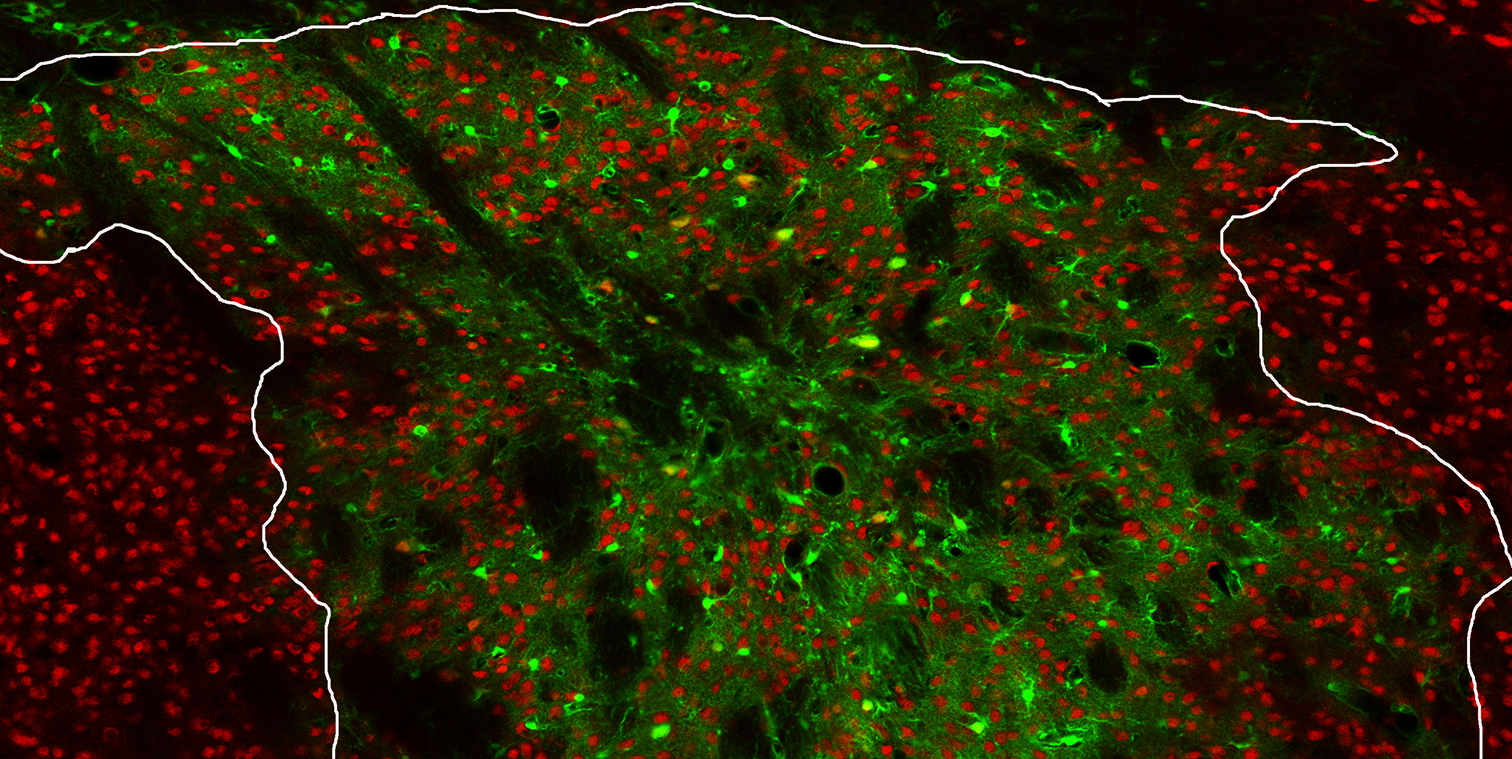

Supplement: Figure 1—source data 3. [file elife-75636-fig1-data3.zip › Fig1 source data 3 for Fig1 F&G/20X/STR shptb 1M MZ1 GFP+neun.jpg]

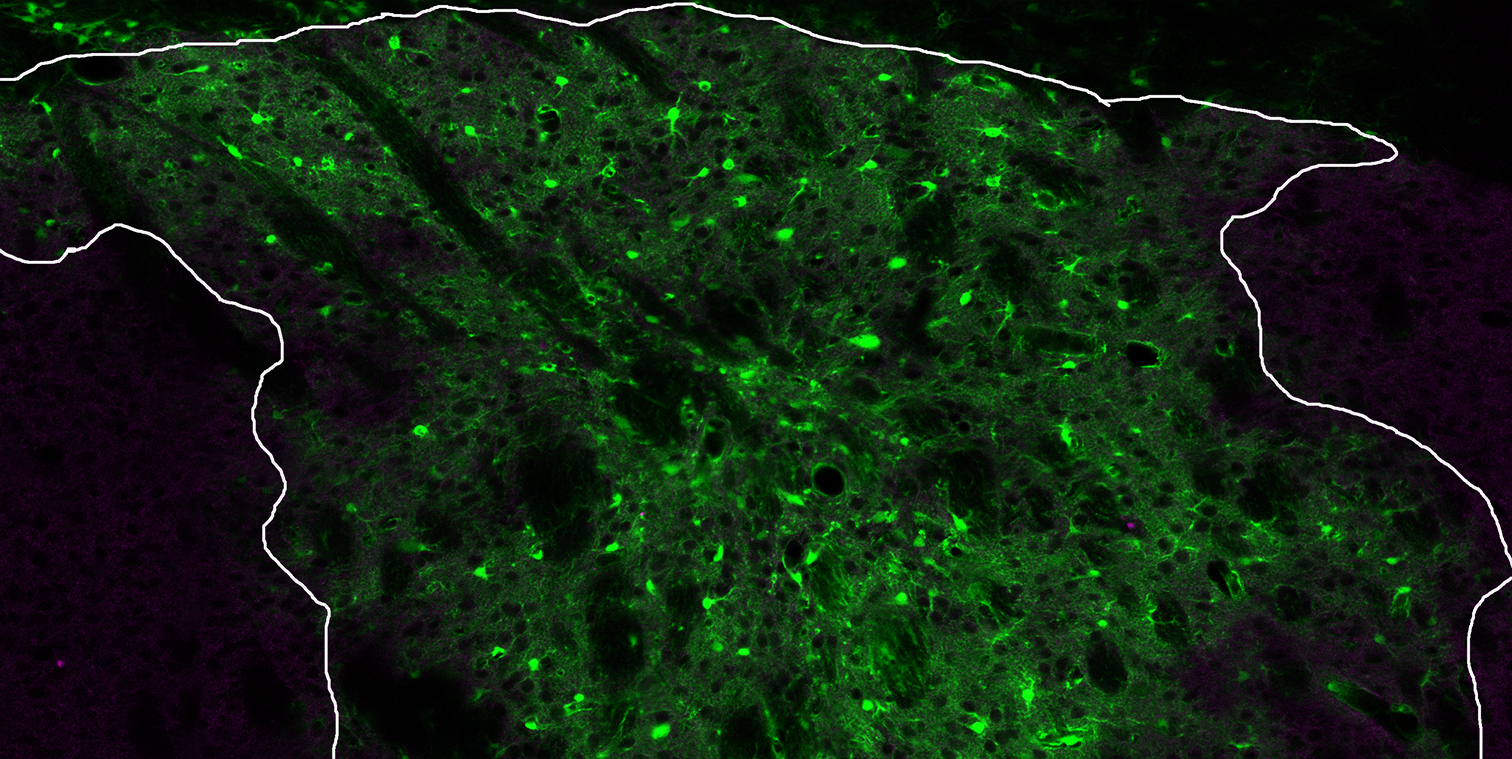

Supplement: Figure 1—source data 3. [file elife-75636-fig1-data3.zip › Fig1 source data 3 for Fig1 F&G/20X/STR shptb 1M MZ1 GFP+TH.jpg]

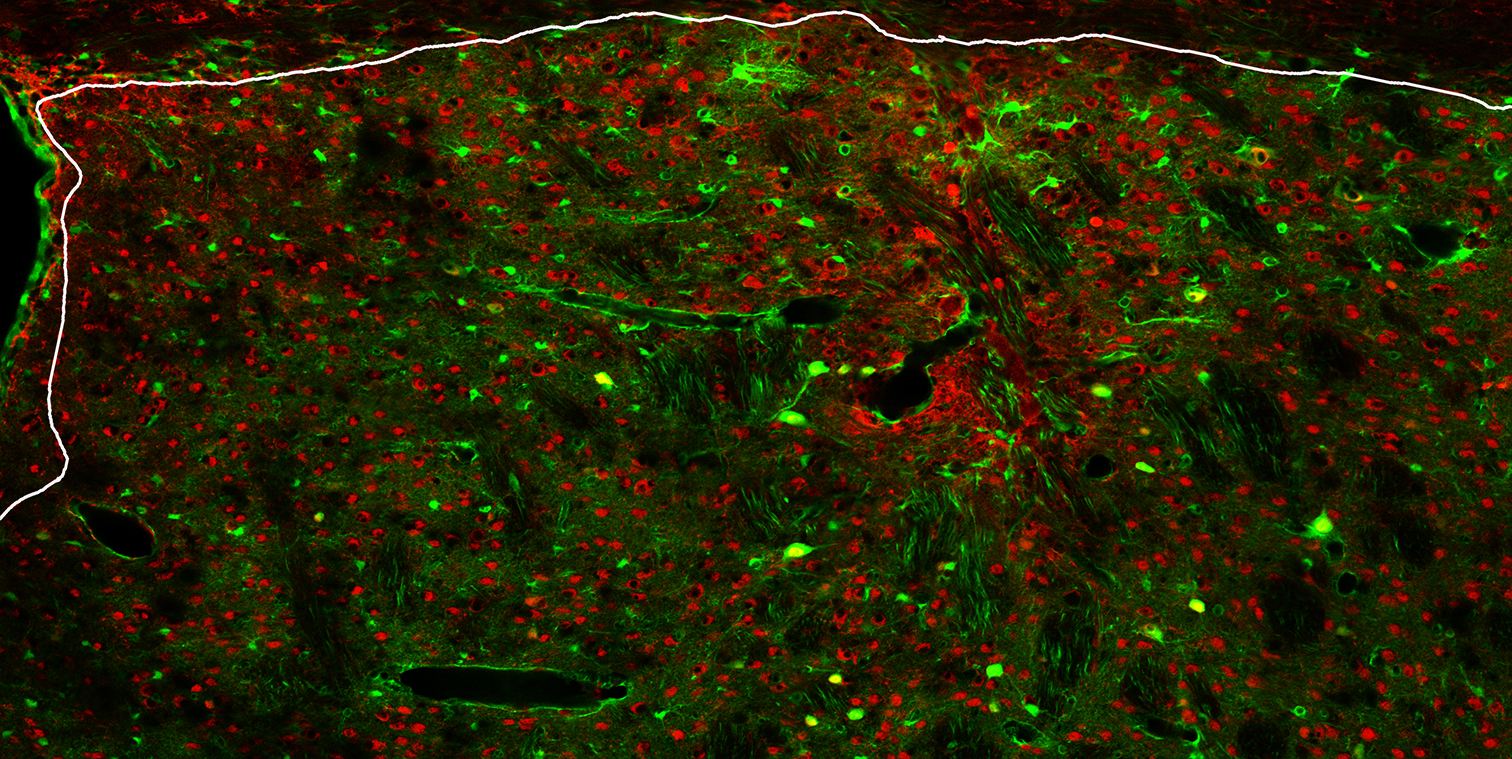

Supplement: Figure 1—source data 3. [file elife-75636-fig1-data3.zip › Fig1 source data 3 for Fig1 F&G/20X/STR shptb 1M MZ2 GFP+neun.jpg]

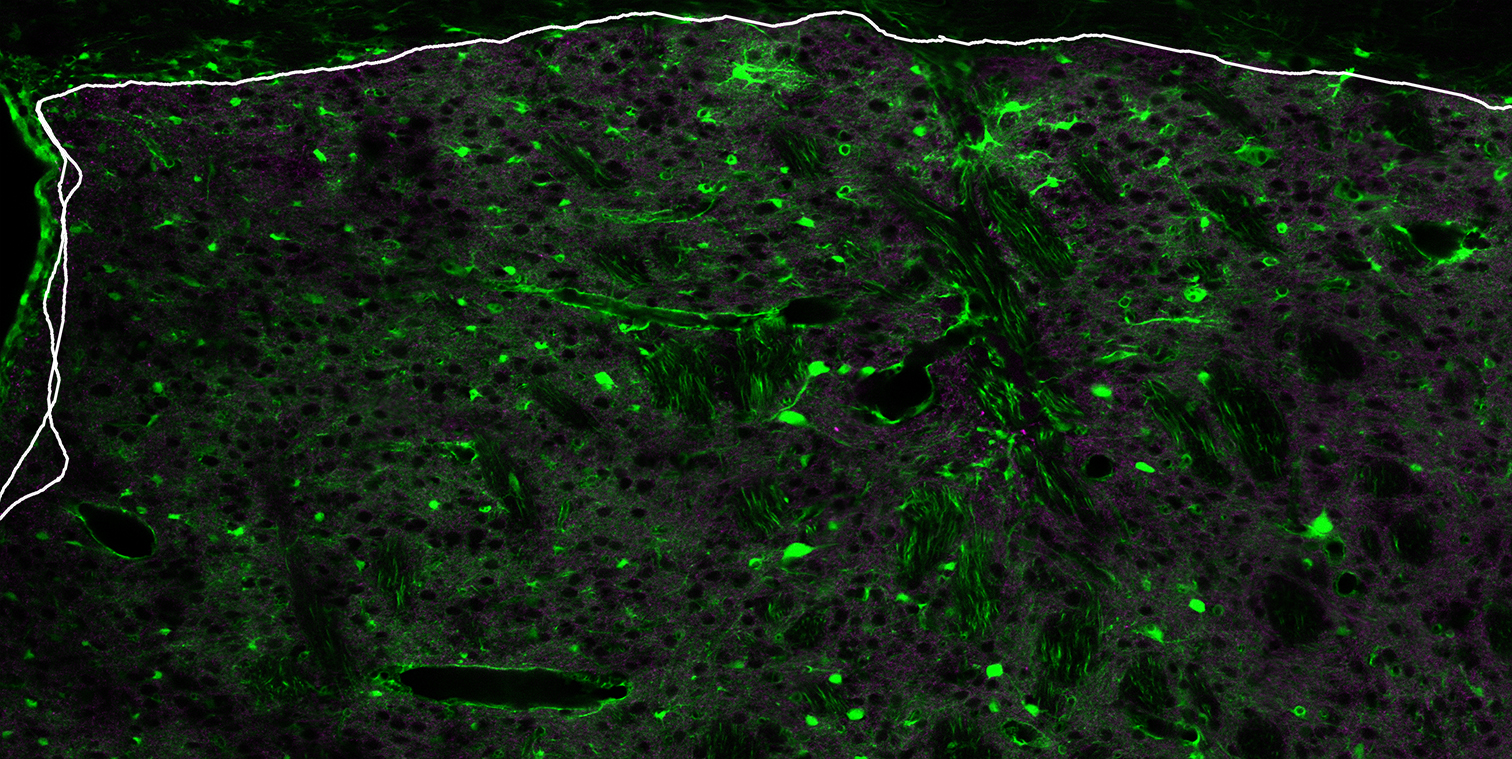

Supplement: Figure 1—source data 3. [file elife-75636-fig1-data3.zip › Fig1 source data 3 for Fig1 F&G/20X/STR shptb 1M MZ2 GFP+TH.jpg]

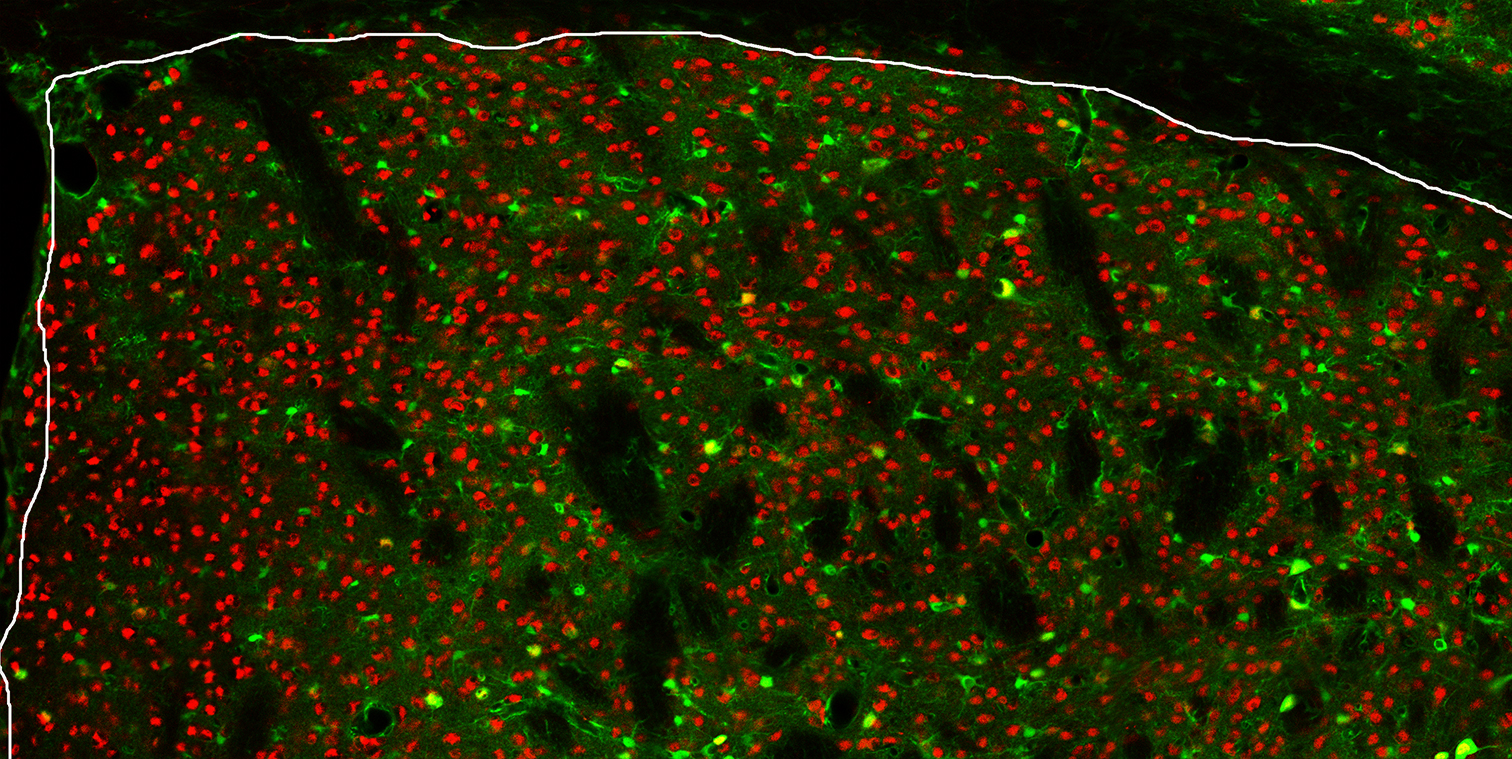

Supplement: Figure 1—source data 3. [file elife-75636-fig1-data3.zip › Fig1 source data 3 for Fig1 F&G/20X/STR shptb 1M MZ3 GFP+neun.jpg]

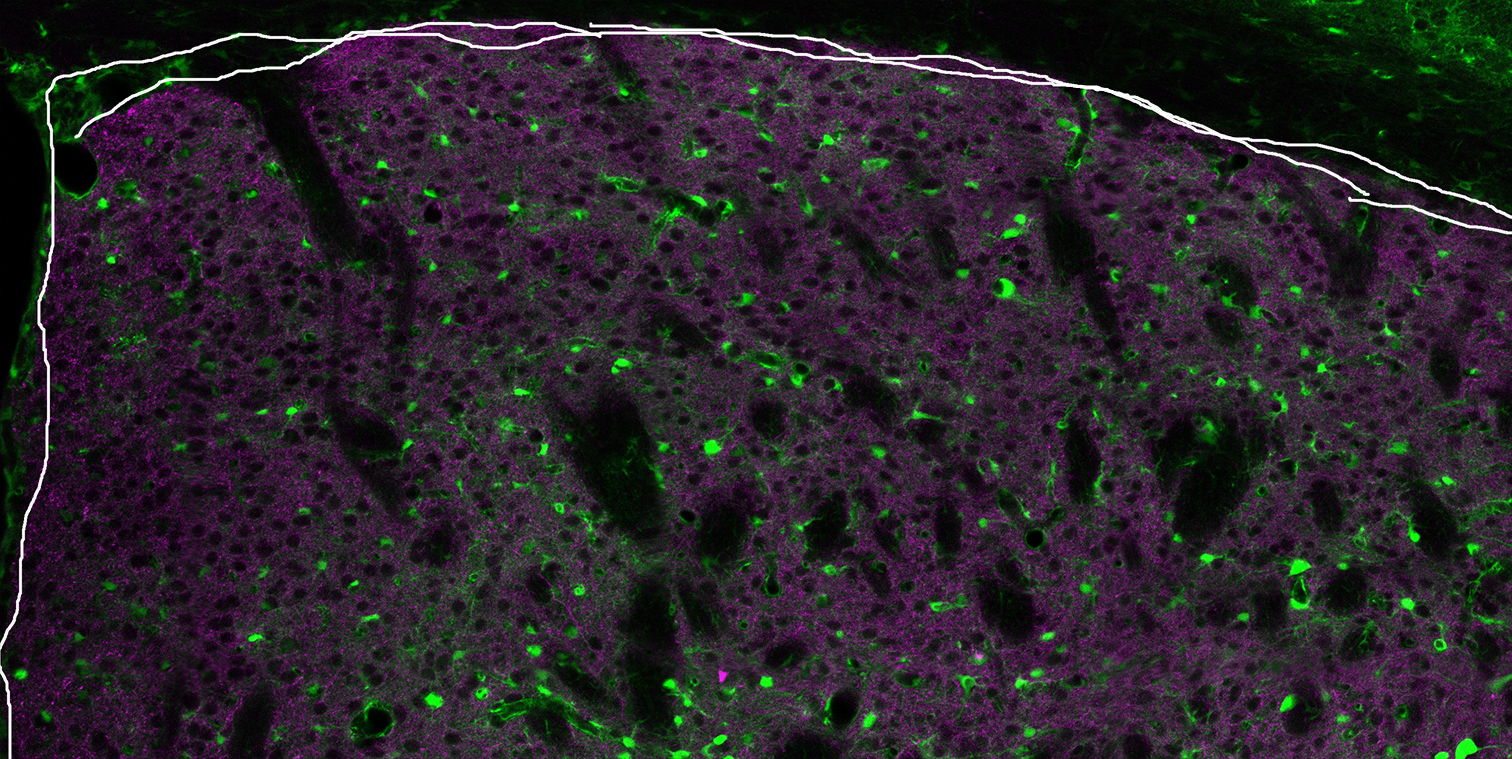

Supplement: Figure 1—source data 3. [file elife-75636-fig1-data3.zip › Fig1 source data 3 for Fig1 F&G/20X/STR shptb 1M MZ3 GFP+TH.jpg]

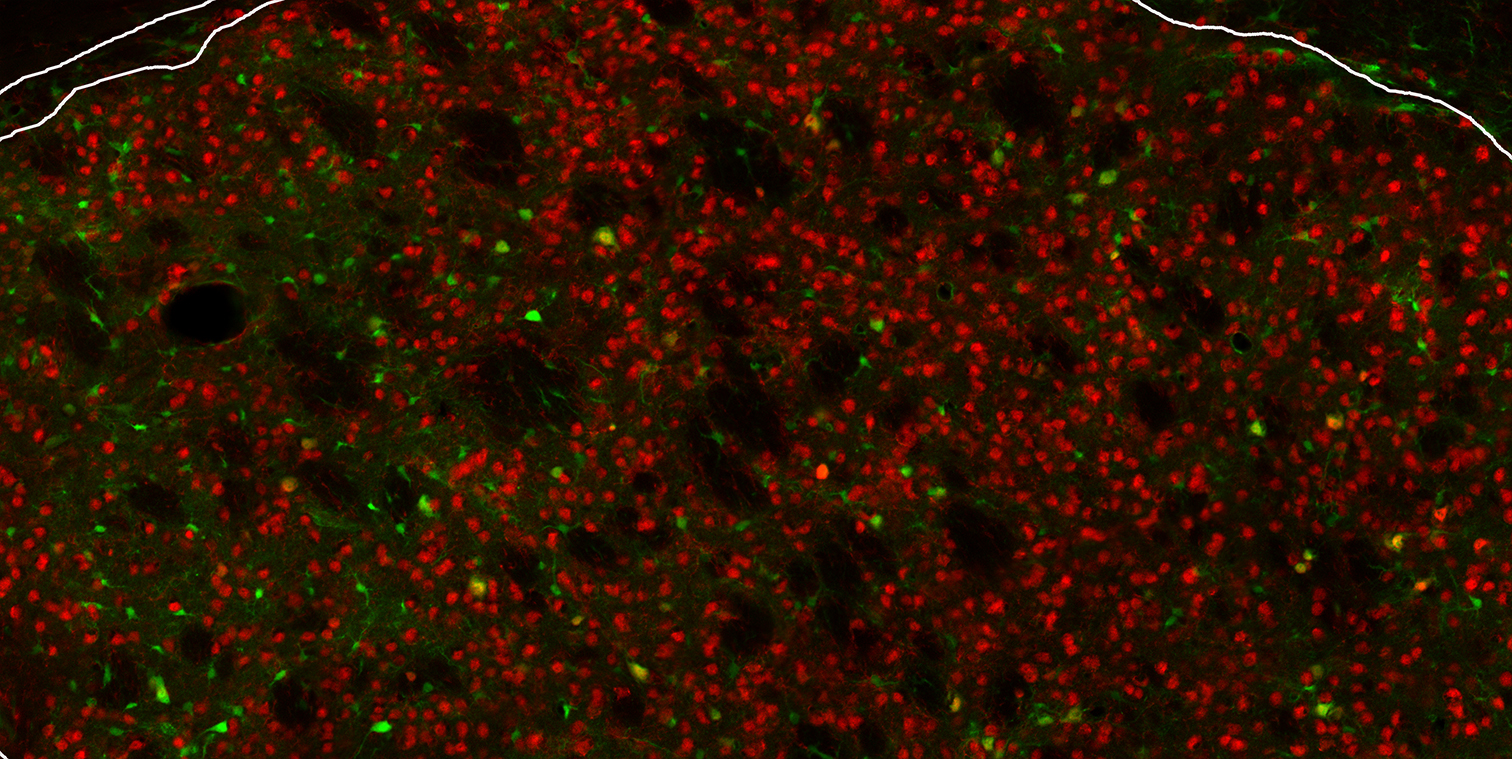

Supplement: Figure 1—source data 3. [file elife-75636-fig1-data3.zip › Fig1 source data 3 for Fig1 F&G/20X/STR shptb 2M MZ1 GFP+neun.jpg]

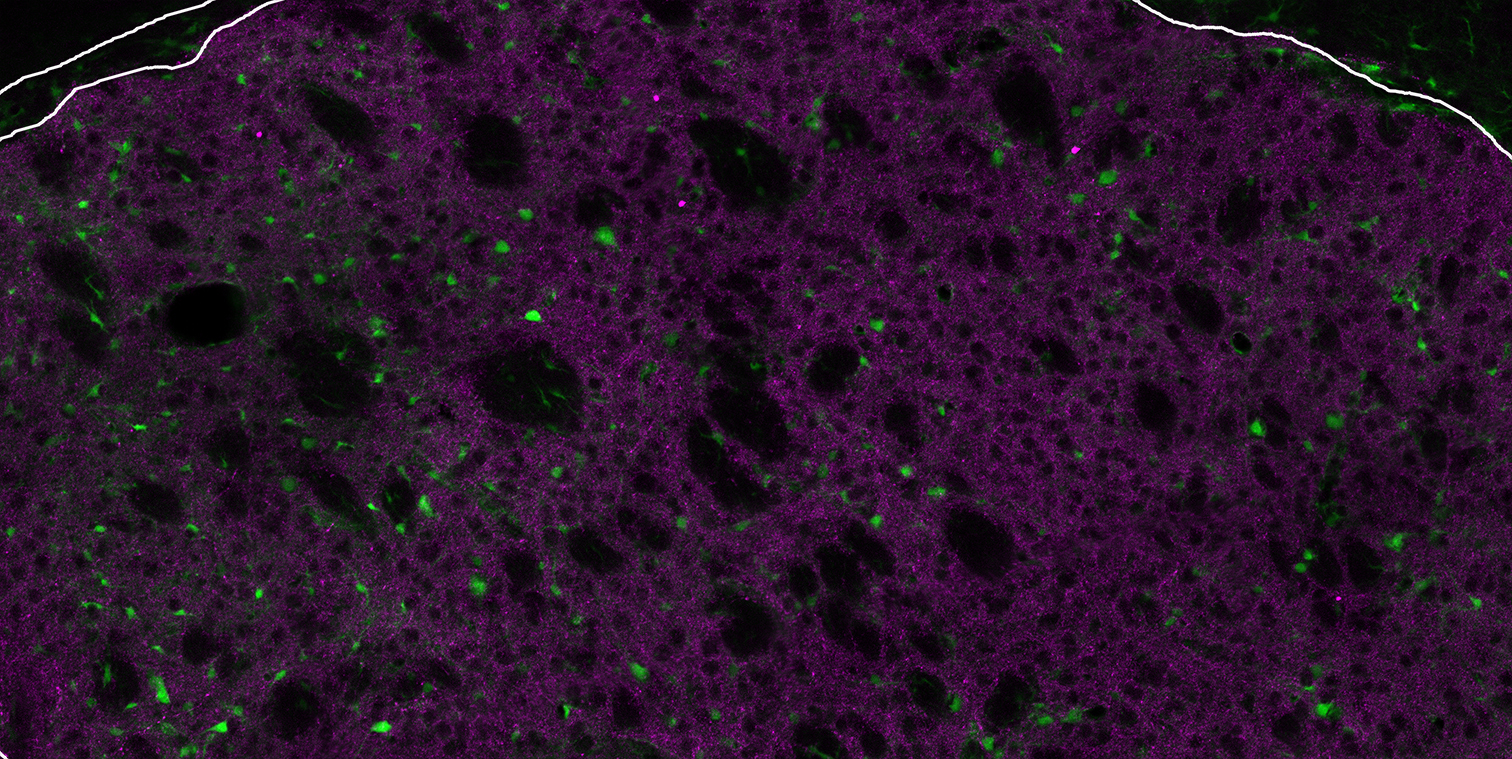

Supplement: Figure 1—source data 3. [file elife-75636-fig1-data3.zip › Fig1 source data 3 for Fig1 F&G/20X/STR shptb 2M MZ1 GFP+TH.jpg]

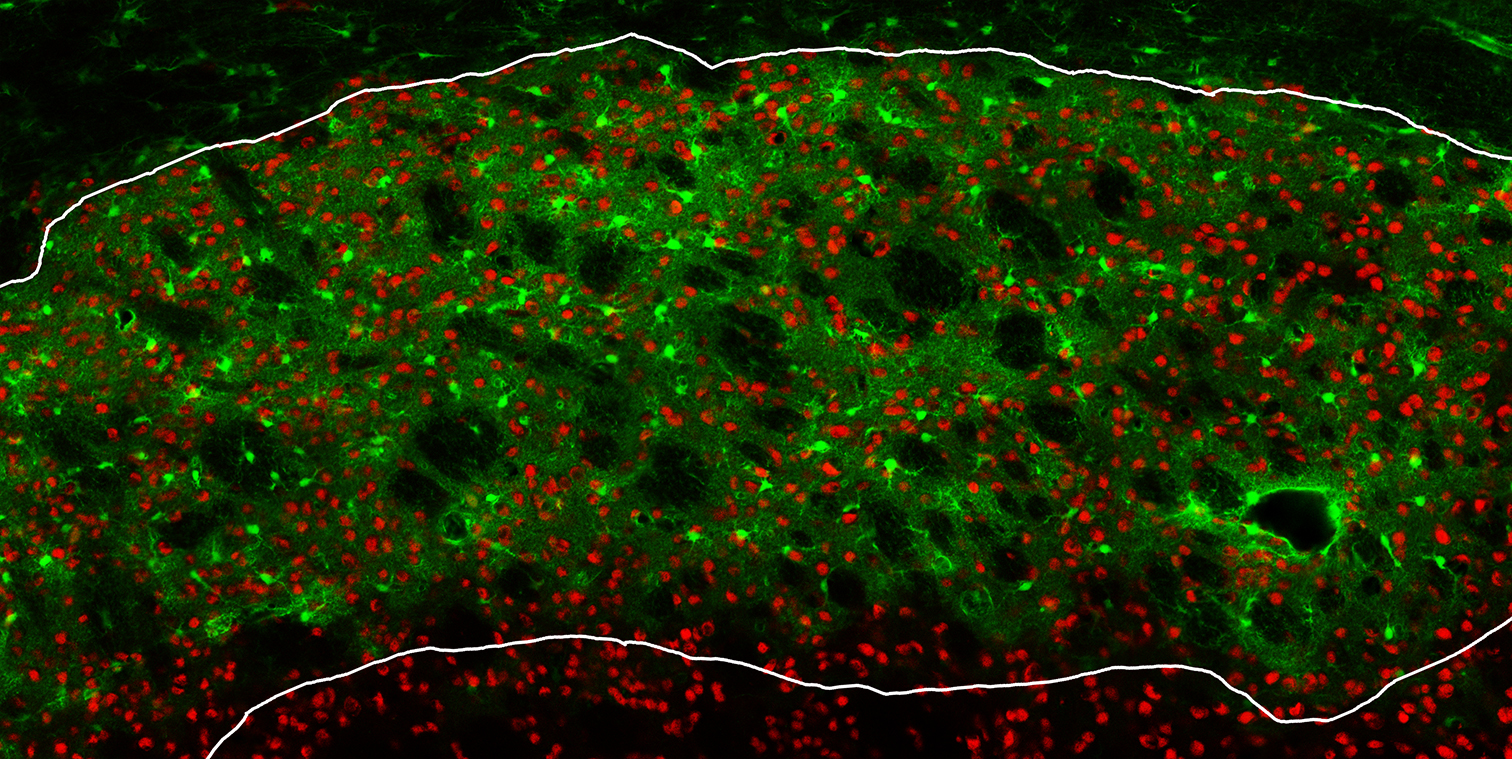

Supplement: Figure 1—source data 3. [file elife-75636-fig1-data3.zip › Fig1 source data 3 for Fig1 F&G/20X/STR shptb 2M MZ2 GFP+neun.jpg]

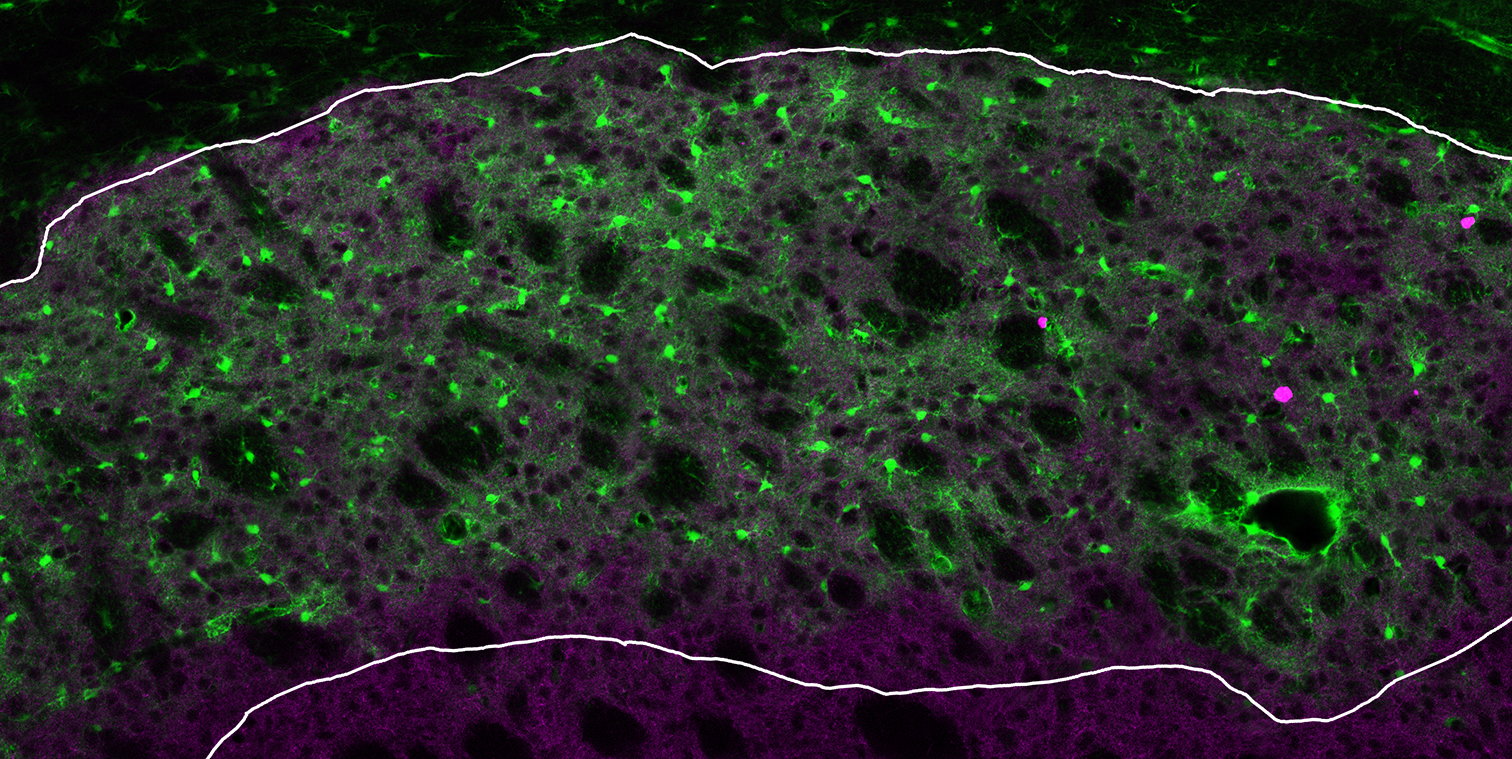

Supplement: Figure 1—source data 3. [file elife-75636-fig1-data3.zip › Fig1 source data 3 for Fig1 F&G/20X/STR shptb 2M MZ2 GFP+TH.jpg]

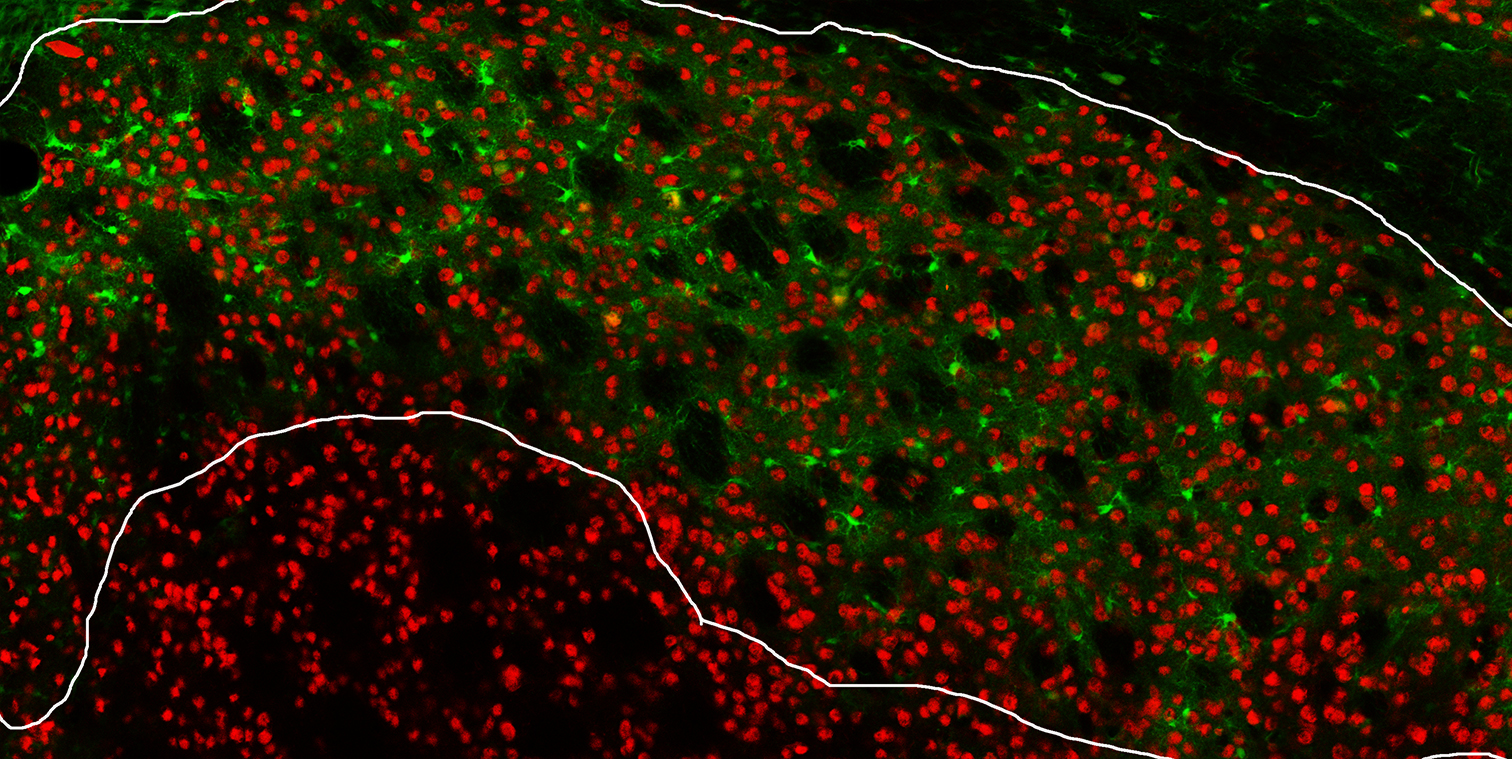

Supplement: Figure 1—source data 3. [file elife-75636-fig1-data3.zip › Fig1 source data 3 for Fig1 F&G/20X/STR shptb 2M MZ3 GFP+neun.jpg]

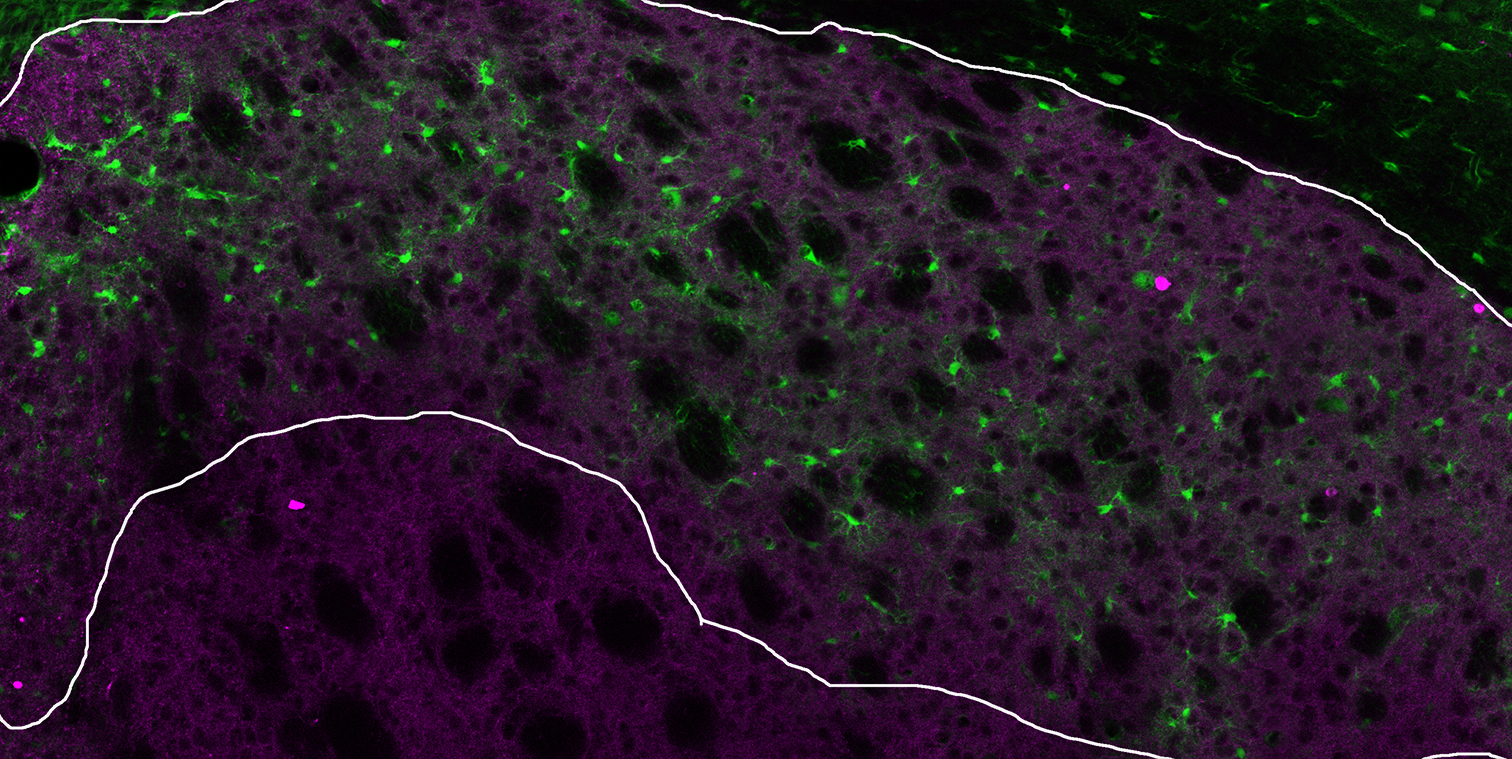

Supplement: Figure 1—source data 3. [file elife-75636-fig1-data3.zip › Fig1 source data 3 for Fig1 F&G/20X/STR shptb 2M MZ3 GFP+TH.jpg]

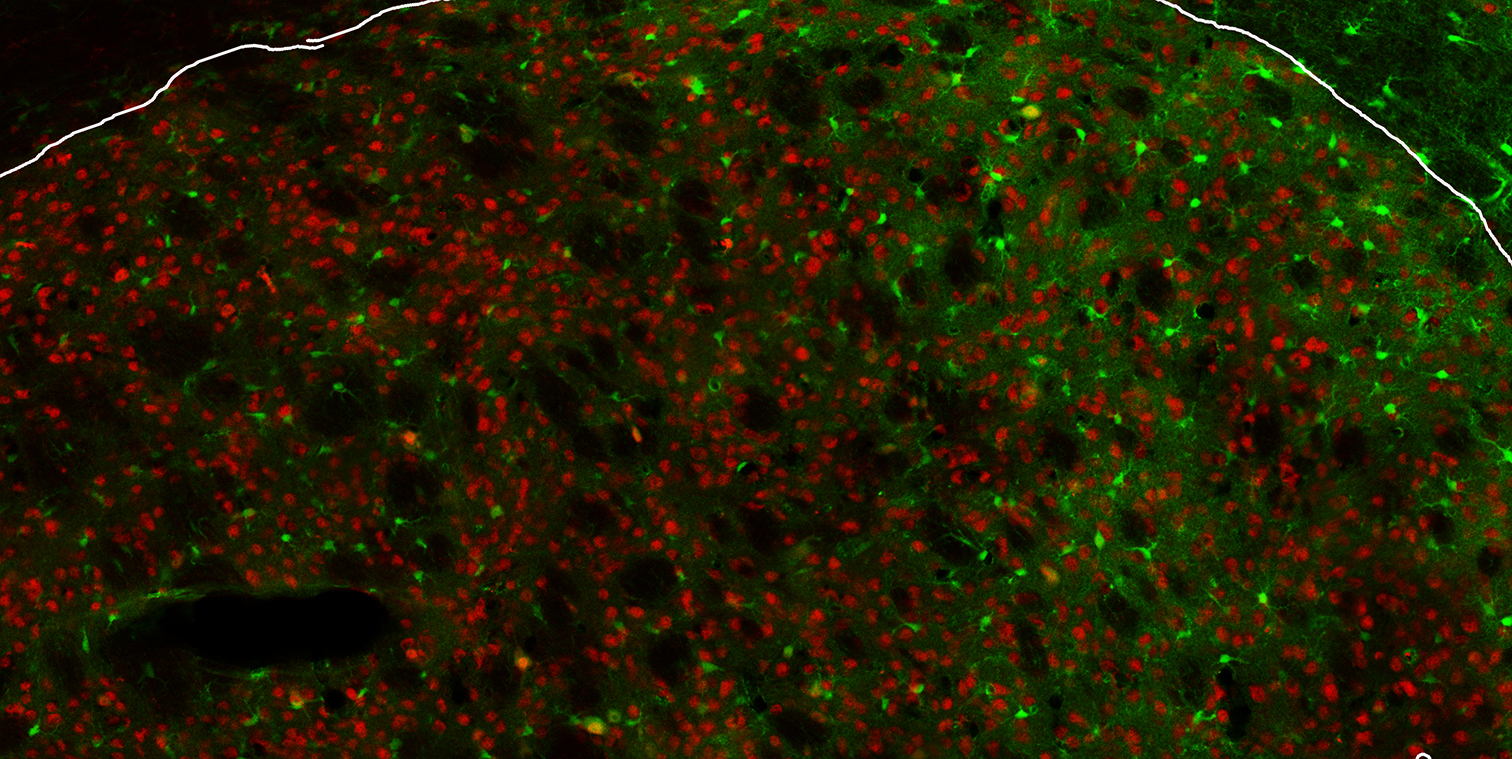

Supplement: Figure 1—source data 3. [file elife-75636-fig1-data3.zip › Fig1 source data 3 for Fig1 F&G/20X/STR shptb 3M MZ1 GFP+neun.jpg]

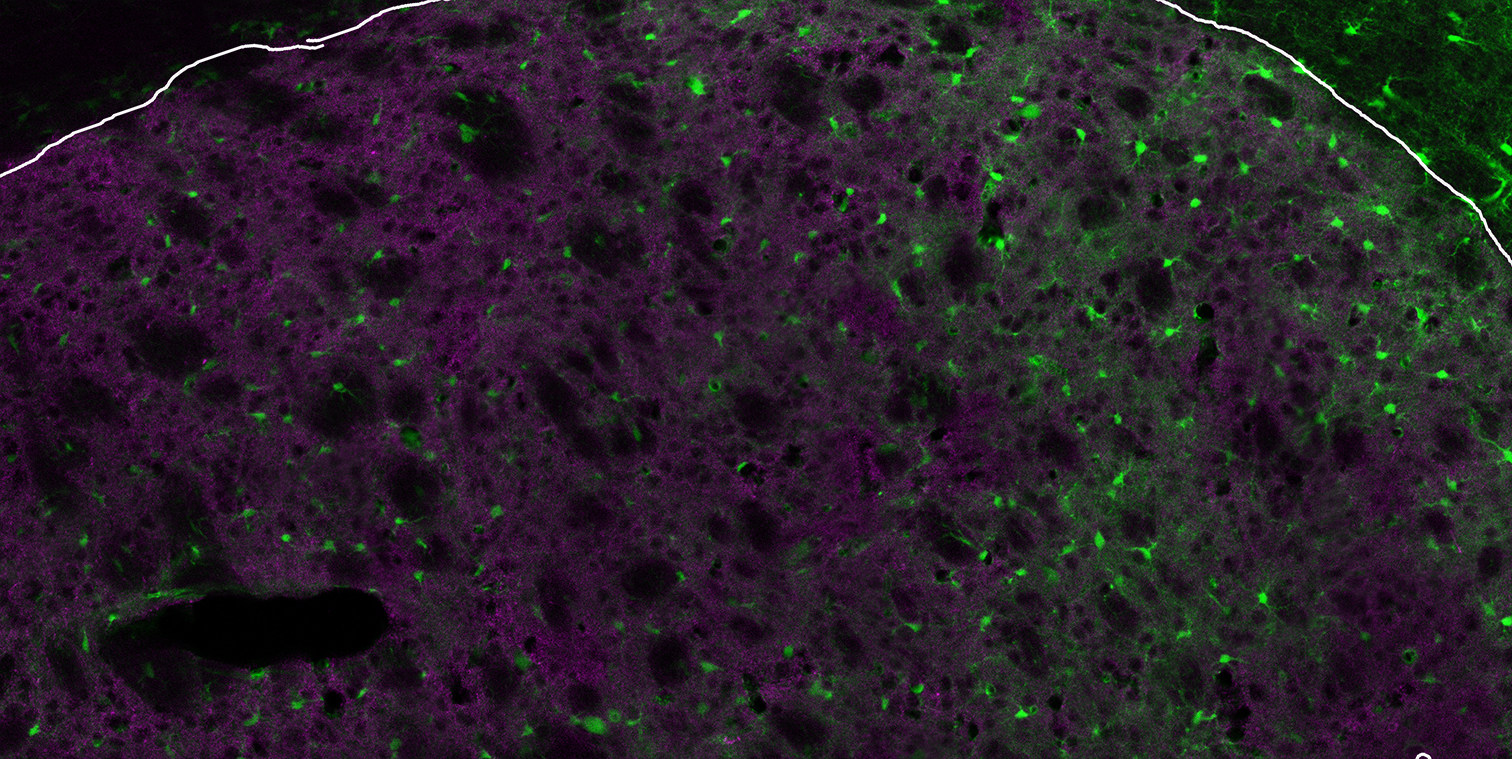

Supplement: Figure 1—source data 3. [file elife-75636-fig1-data3.zip › Fig1 source data 3 for Fig1 F&G/20X/STR shptb 3M MZ1 GFP+TH.jpg]

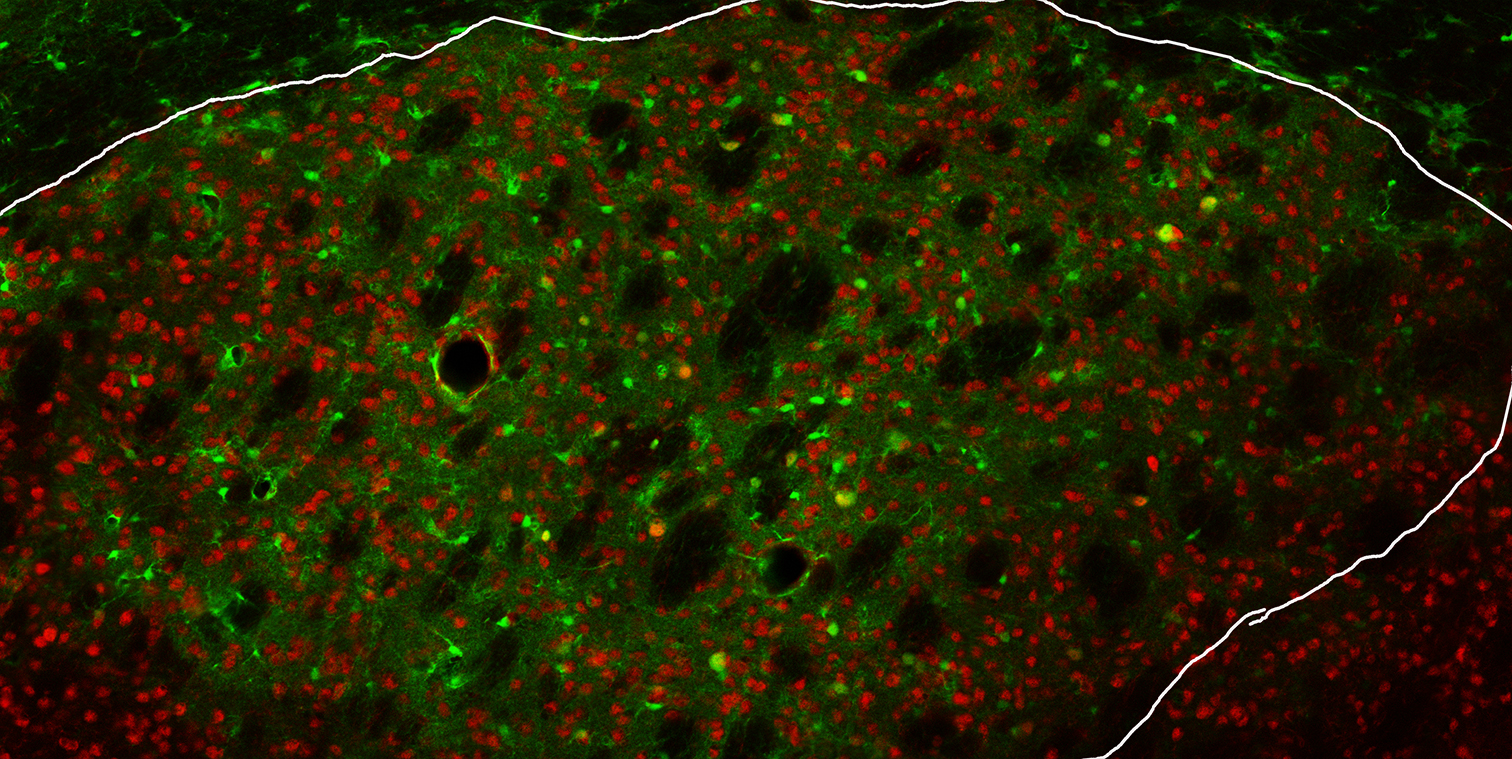

Supplement: Figure 1—source data 3. [file elife-75636-fig1-data3.zip › Fig1 source data 3 for Fig1 F&G/20X/STR shptb 3M MZ2 GFP+neun.jpg]

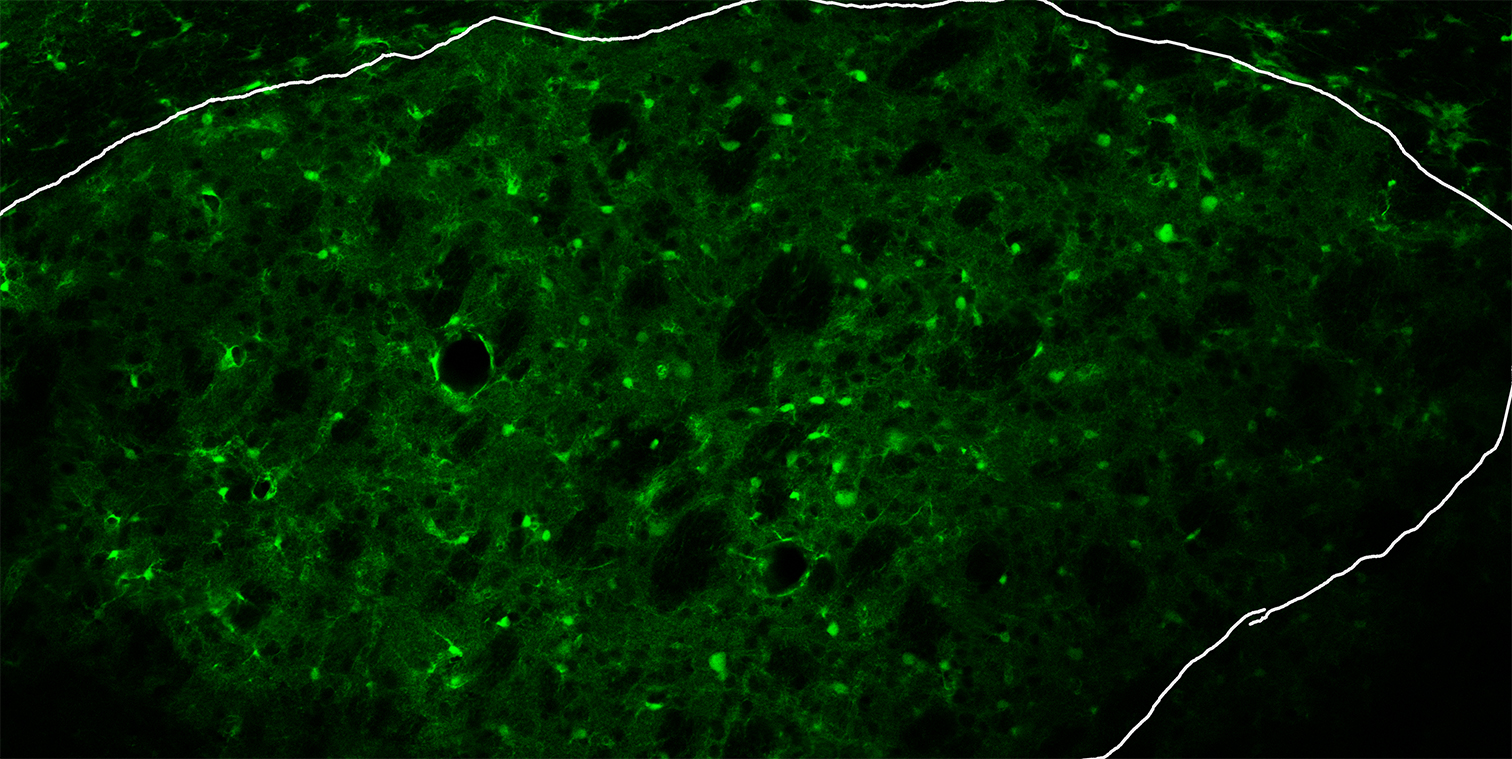

Supplement: Figure 1—source data 3. [file elife-75636-fig1-data3.zip › Fig1 source data 3 for Fig1 F&G/20X/STR shptb 3M MZ2 GFP+TH.jpg]

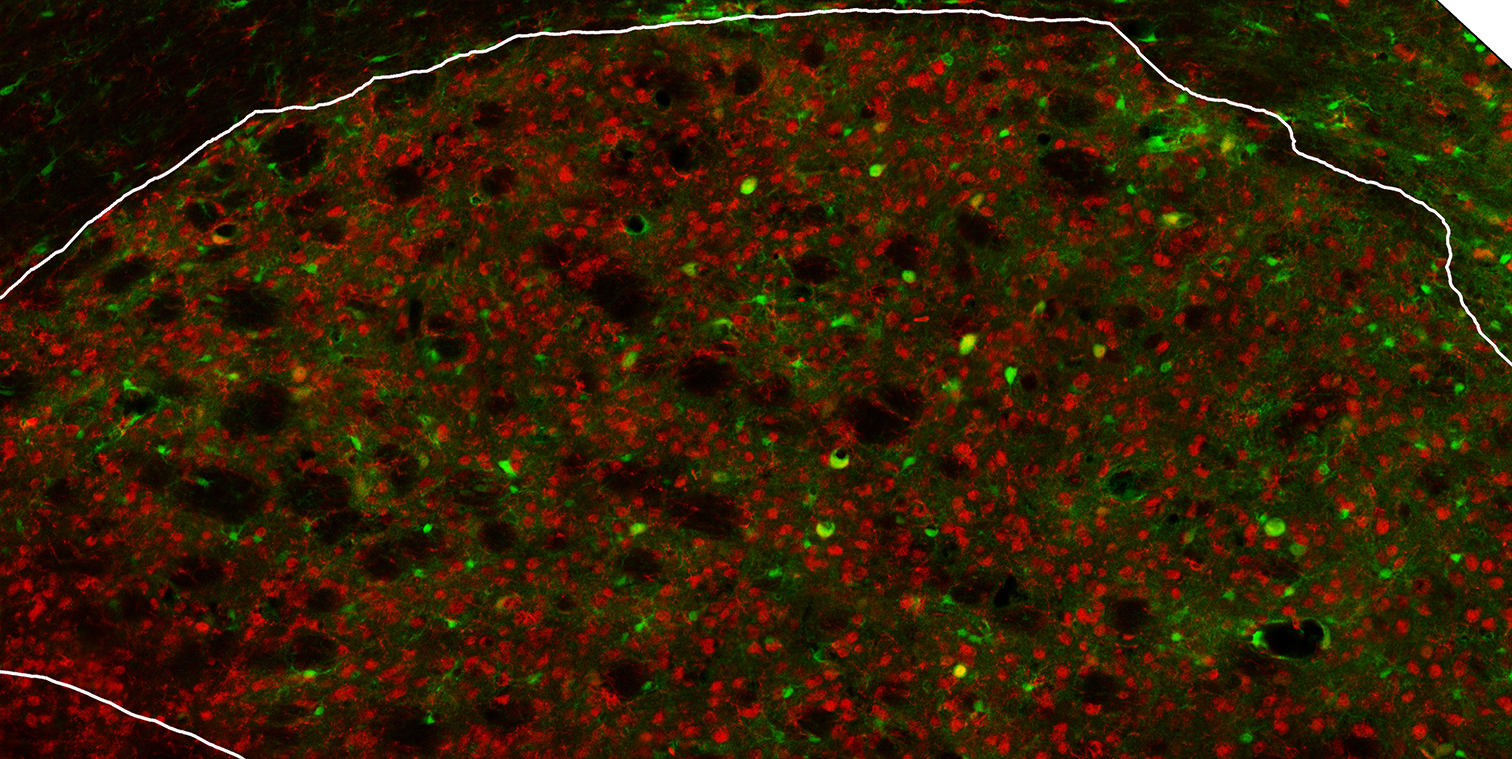

Supplement: Figure 1—source data 3. [file elife-75636-fig1-data3.zip › Fig1 source data 3 for Fig1 F&G/20X/STR shptb 3M MZ3 GFP+neun.jpg]

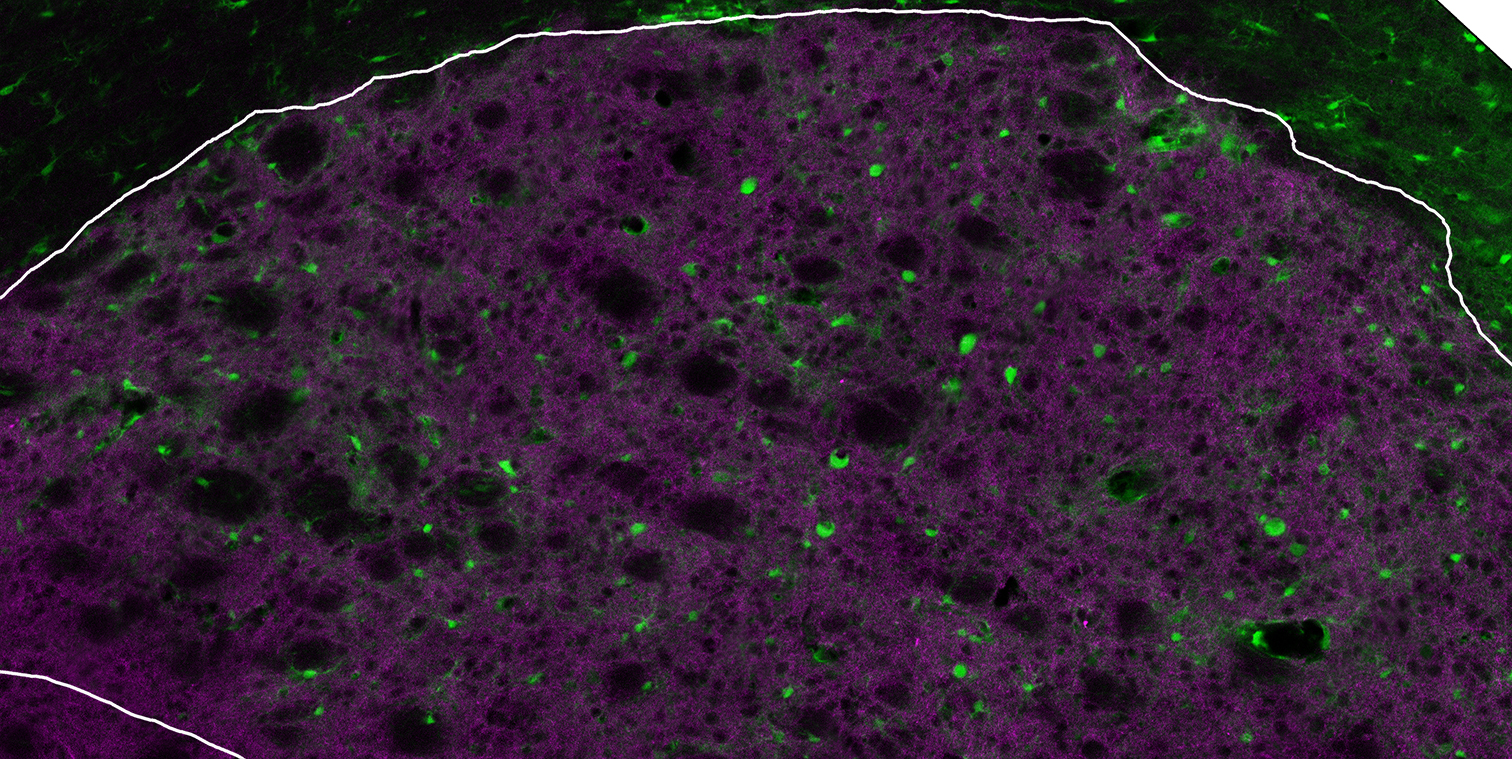

Supplement: Figure 1—source data 3. [file elife-75636-fig1-data3.zip › Fig1 source data 3 for Fig1 F&G/20X/STR shptb 3M MZ3 GFP+TH.jpg]

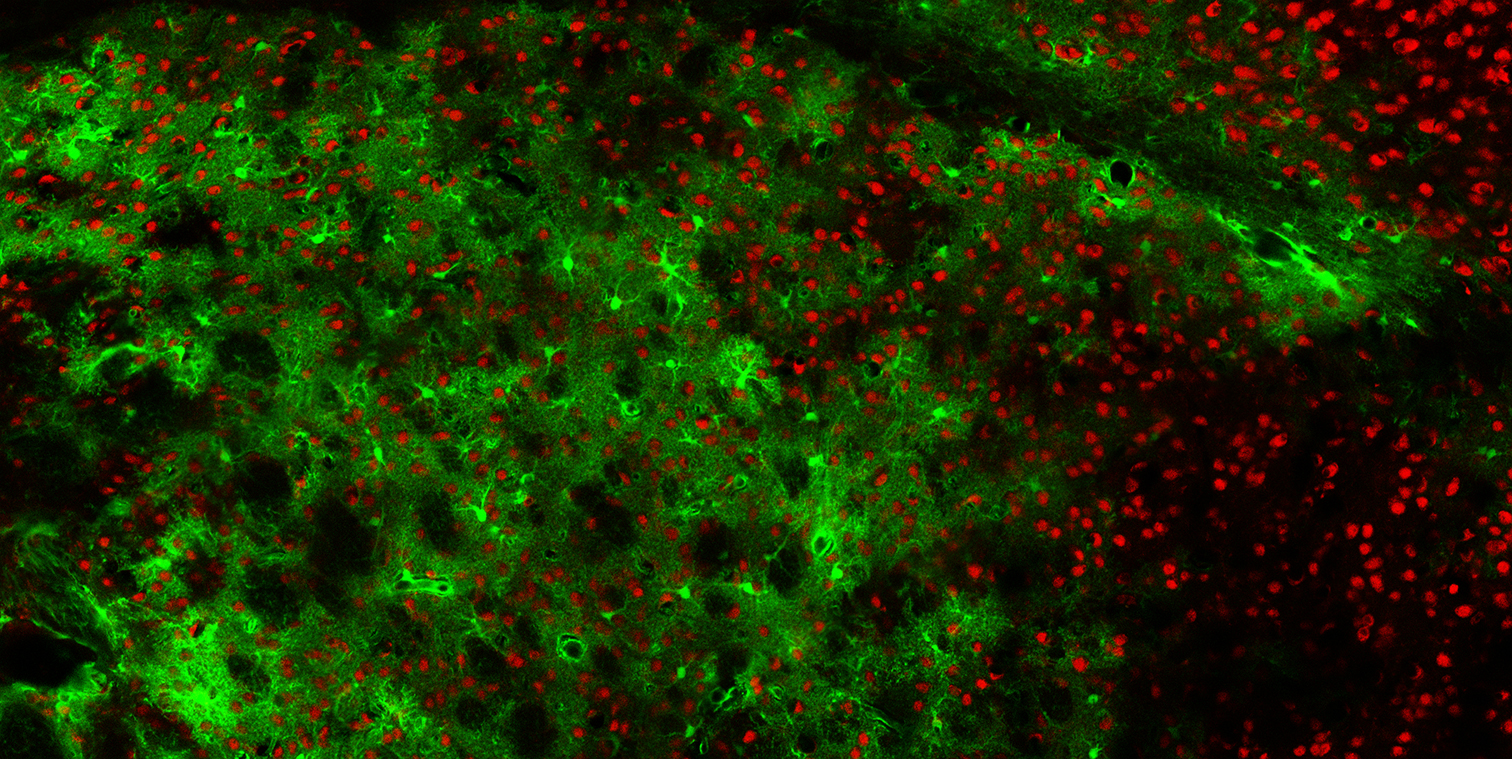

Supplement: Figure 1—source data 3. [file elife-75636-fig1-data3.zip › Fig1 source data 3 for Fig1 F&G/20X/STR shscramble 1M MZ1 GFP+neun.jpg]

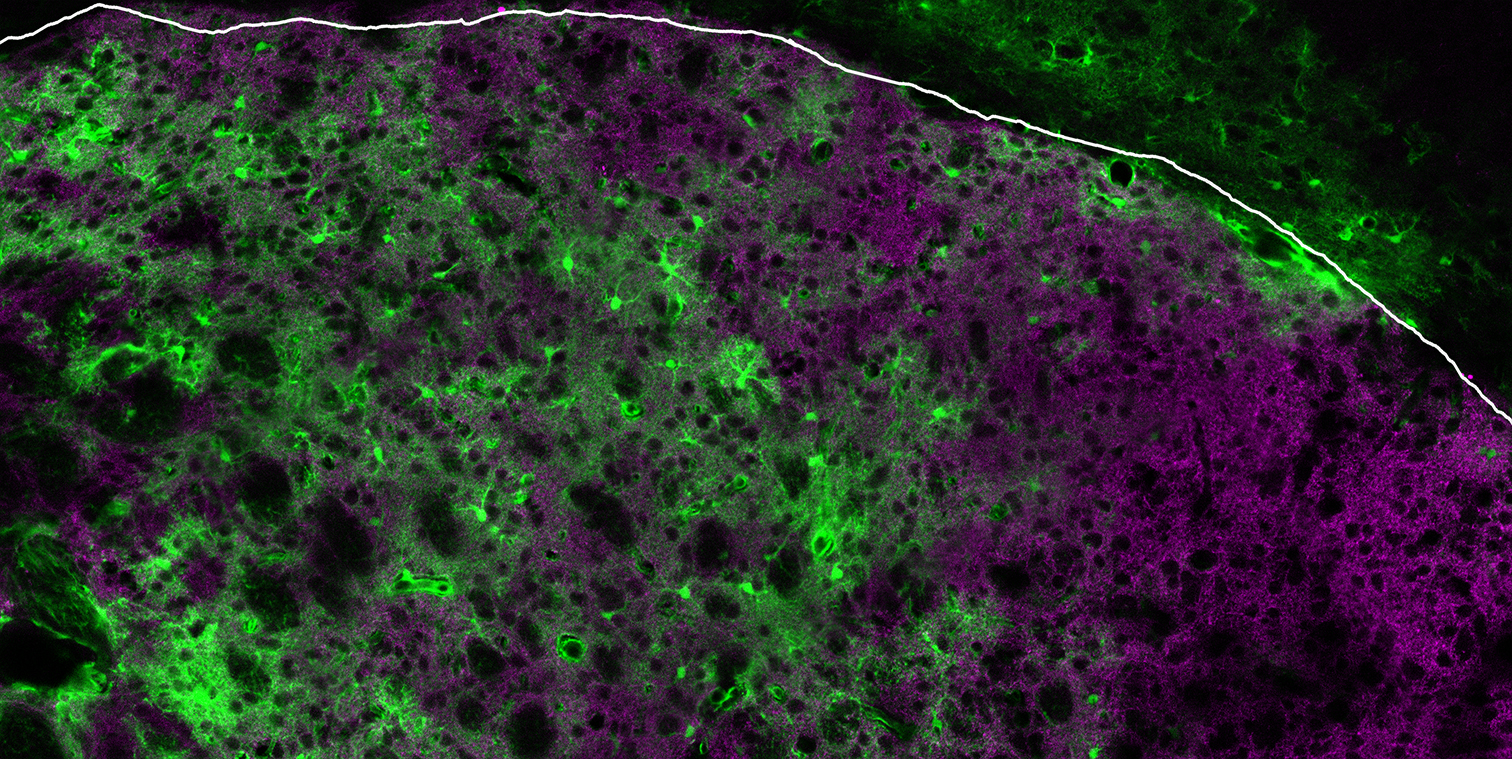

Supplement: Figure 1—source data 3. [file elife-75636-fig1-data3.zip › Fig1 source data 3 for Fig1 F&G/20X/STR shscramble 1M MZ1 GFP+TH.jpg]

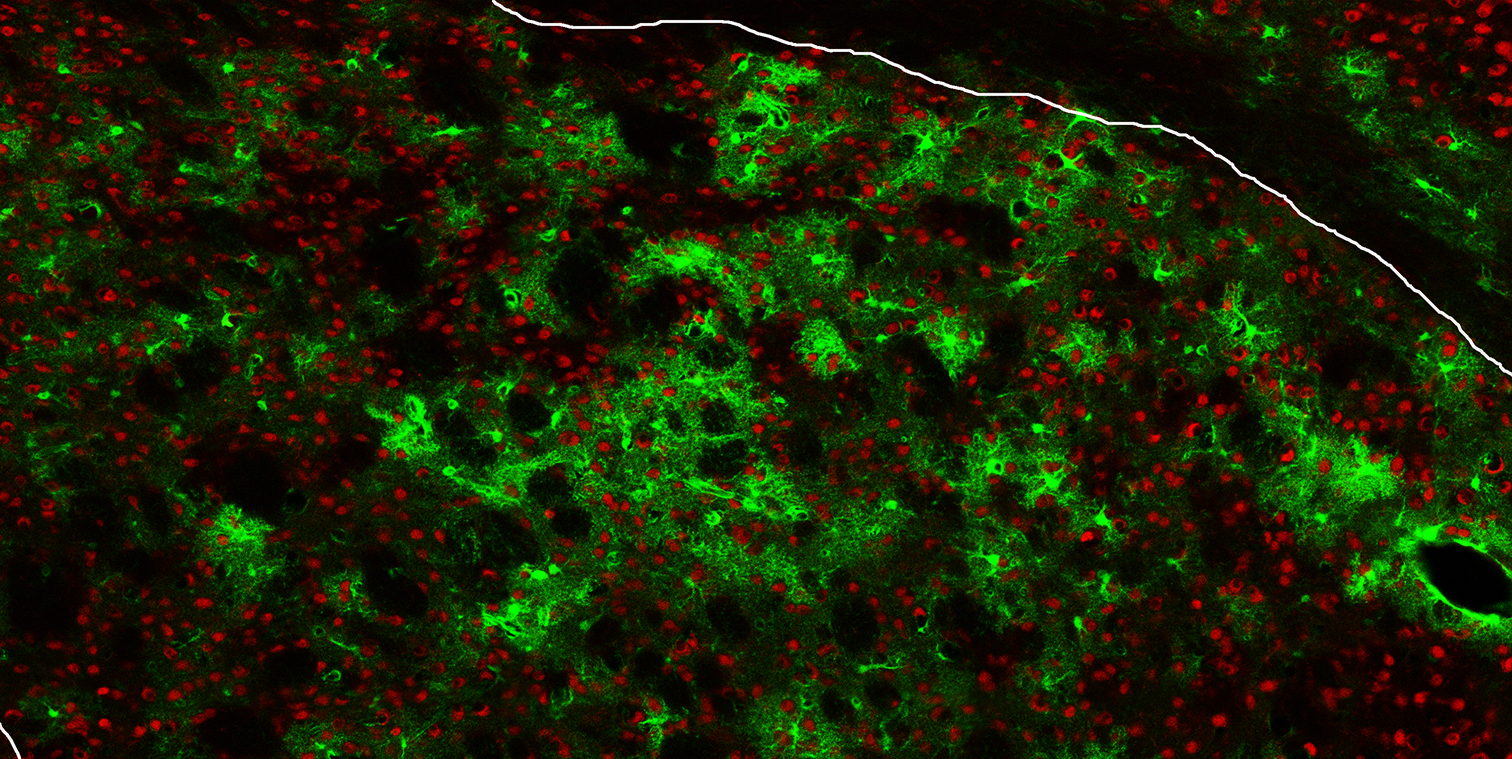

Supplement: Figure 1—source data 3. [file elife-75636-fig1-data3.zip › Fig1 source data 3 for Fig1 F&G/20X/STR shscramble 1M MZ2 GFP+neun.jpg]

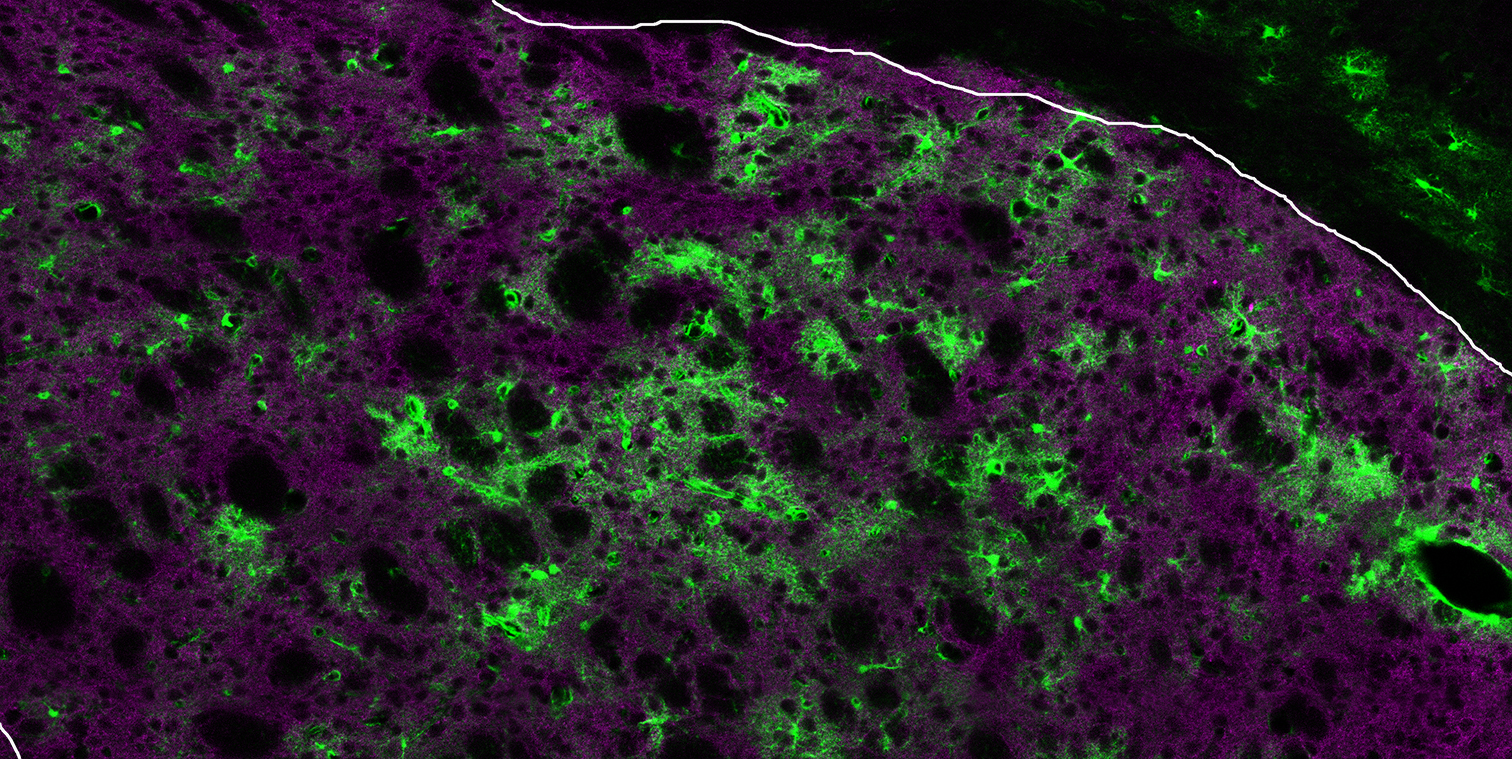

Supplement: Figure 1—source data 3. [file elife-75636-fig1-data3.zip › Fig1 source data 3 for Fig1 F&G/20X/STR shscramble 1M MZ2 GFP+TH.jpg]

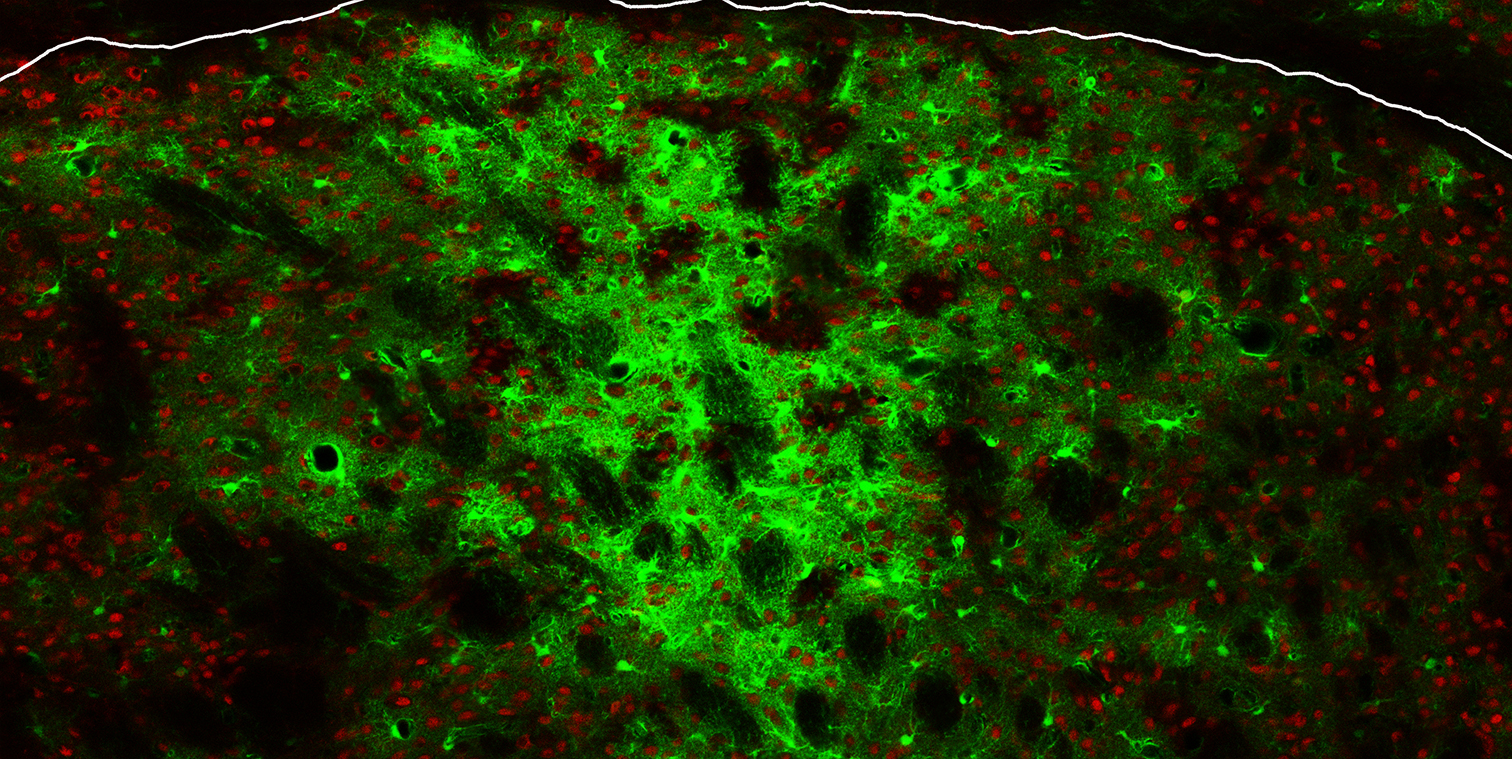

Supplement: Figure 1—source data 3. [file elife-75636-fig1-data3.zip › Fig1 source data 3 for Fig1 F&G/20X/STR shscramble 1M MZ3 GFP+neun.jpg]

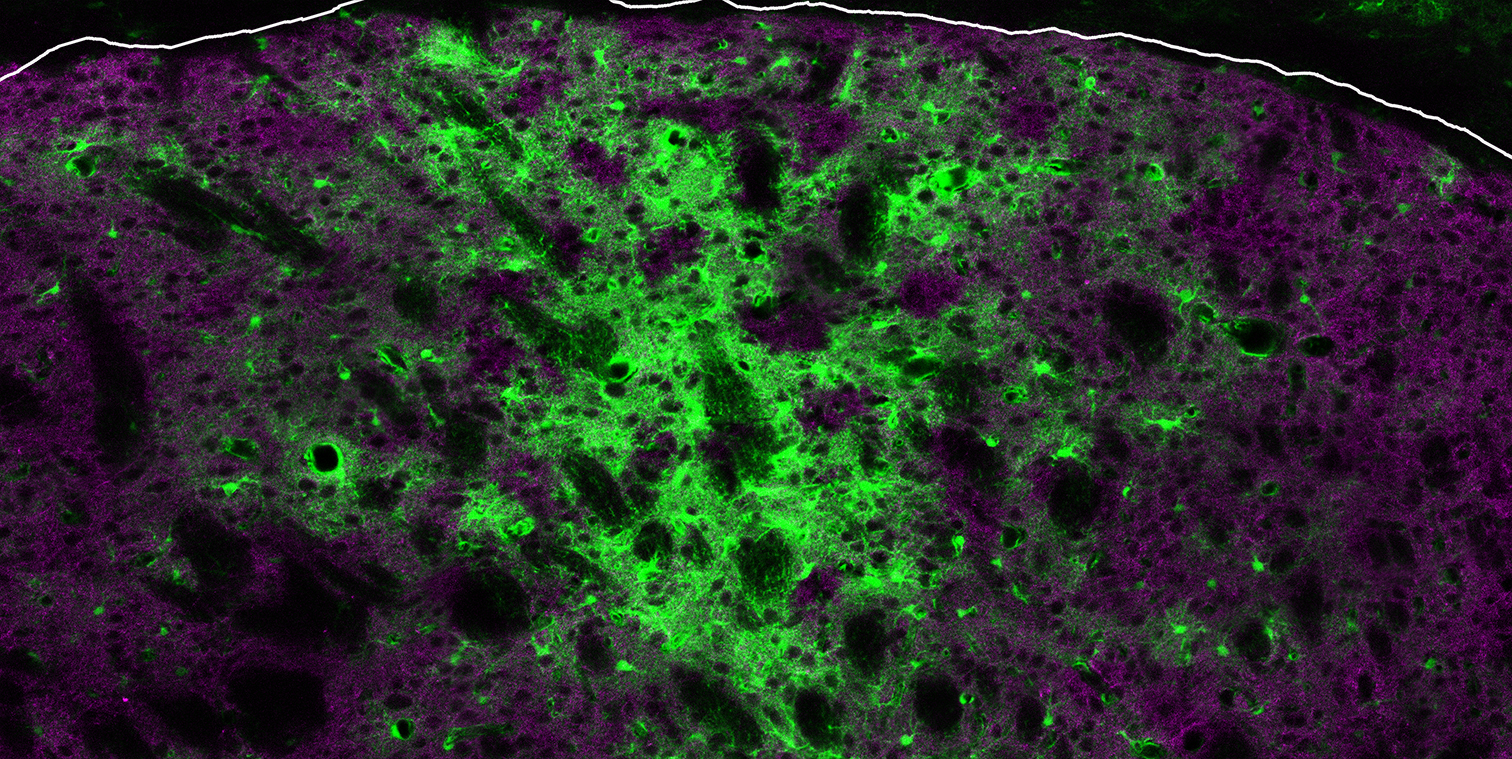

Supplement: Figure 1—source data 3. [file elife-75636-fig1-data3.zip › Fig1 source data 3 for Fig1 F&G/20X/STR shscramble 1M MZ3 GFP+TH.jpg]

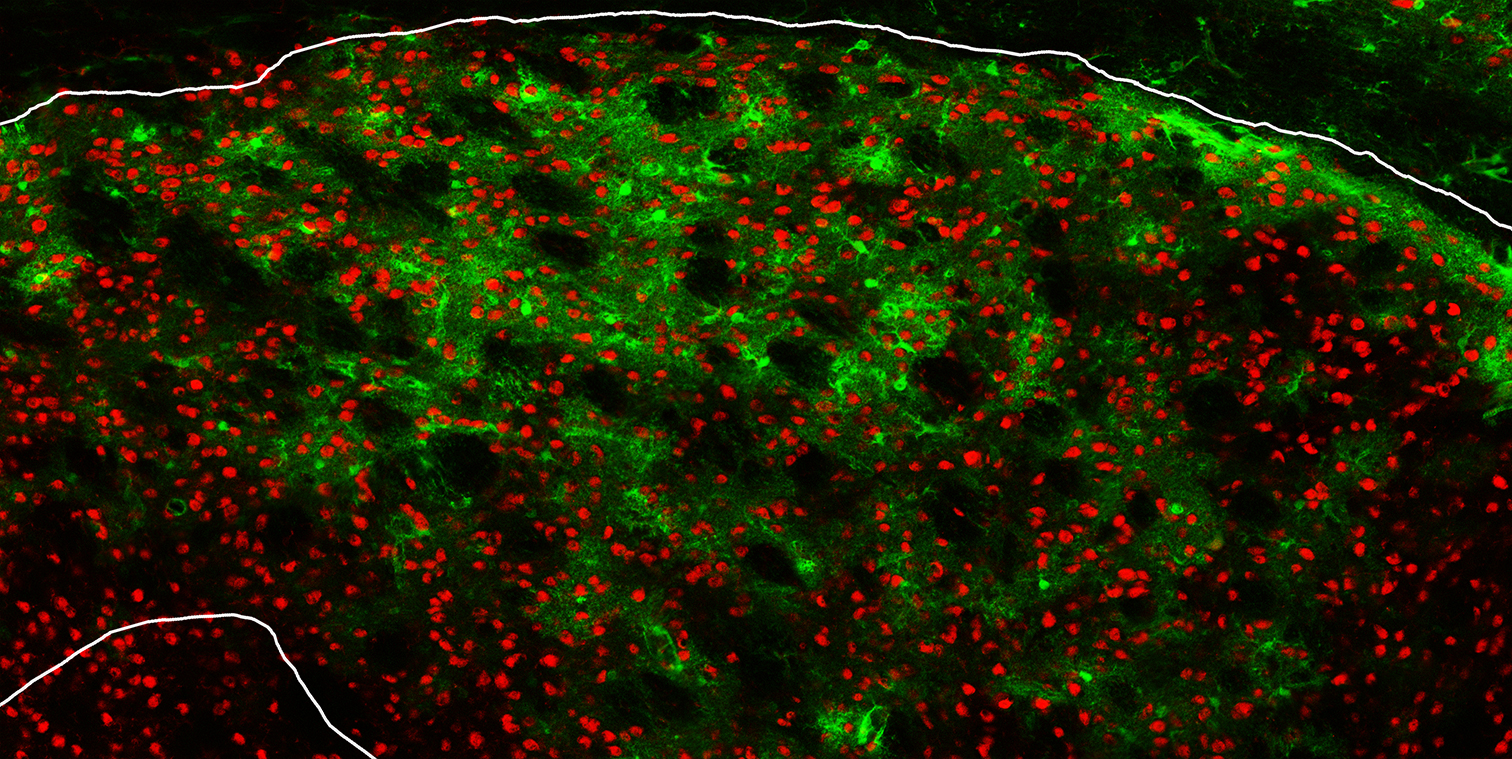

Supplement: Figure 1—source data 3. [file elife-75636-fig1-data3.zip › Fig1 source data 3 for Fig1 F&G/20X/STR shscramble 2M MZ1 GFP+neun.jpg]

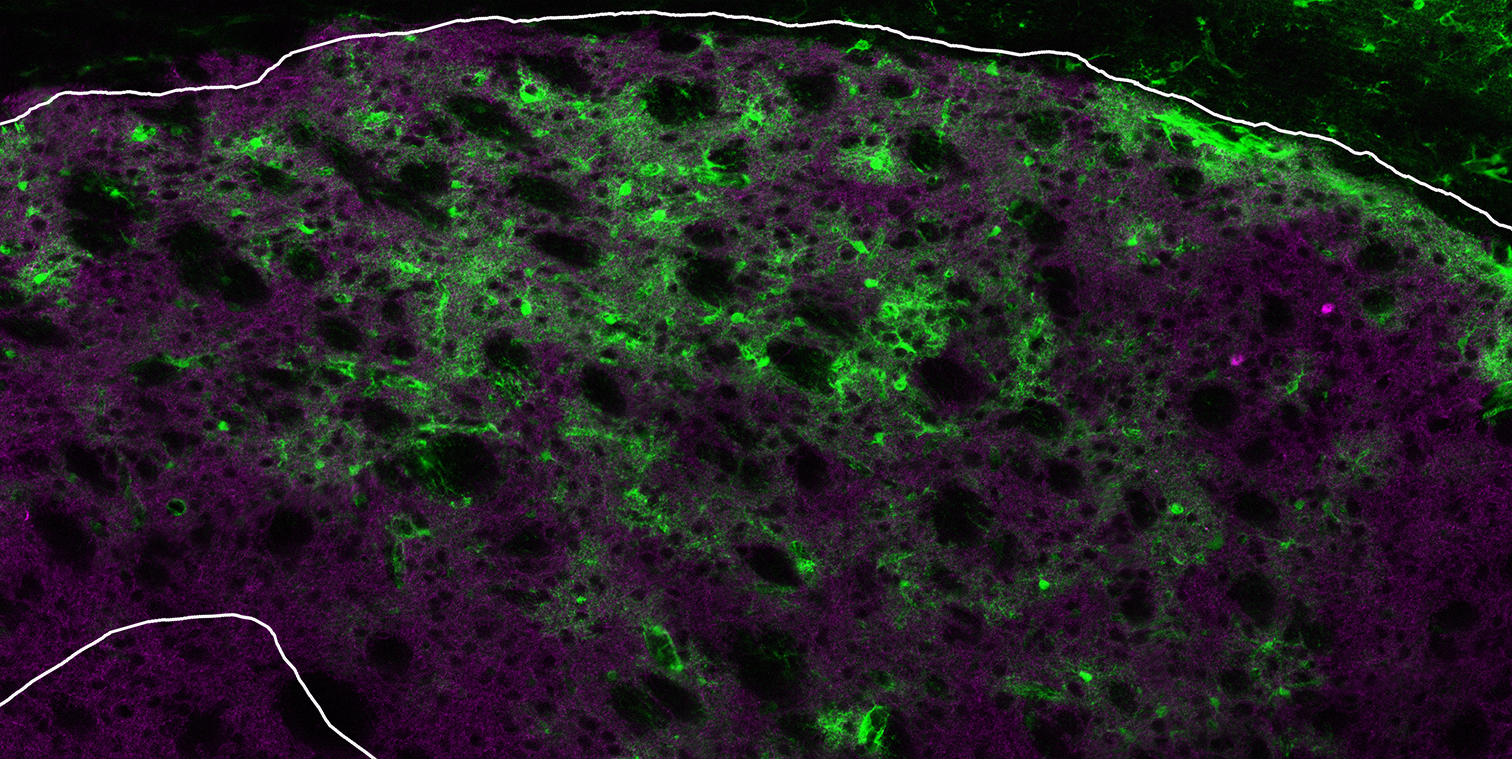

Supplement: Figure 1—source data 3. [file elife-75636-fig1-data3.zip › Fig1 source data 3 for Fig1 F&G/20X/STR shscramble 2M MZ1 GFP+TH.jpg]

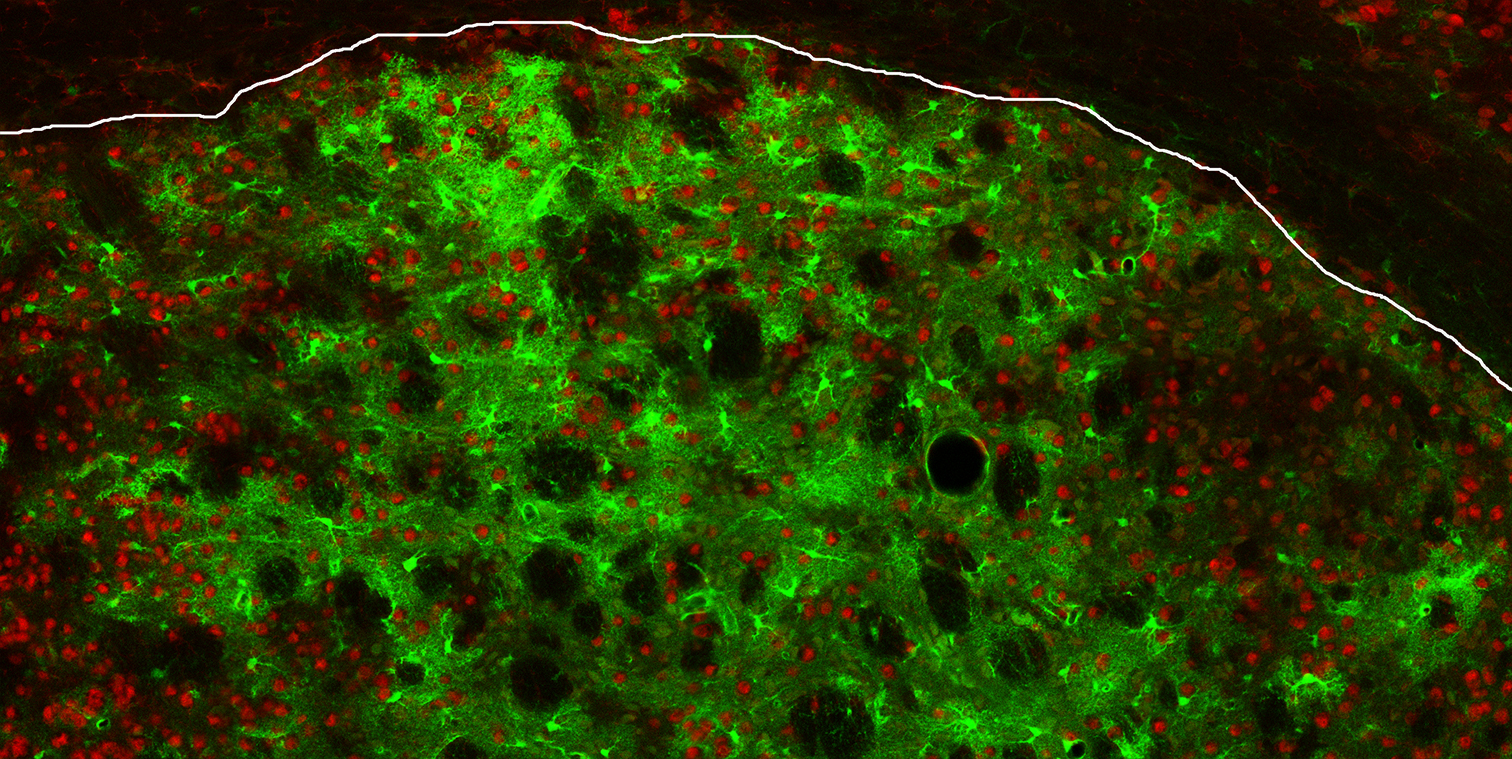

Supplement: Figure 1—source data 3. [file elife-75636-fig1-data3.zip › Fig1 source data 3 for Fig1 F&G/20X/STR shscramble 2M MZ2 GFP+neun.jpg]

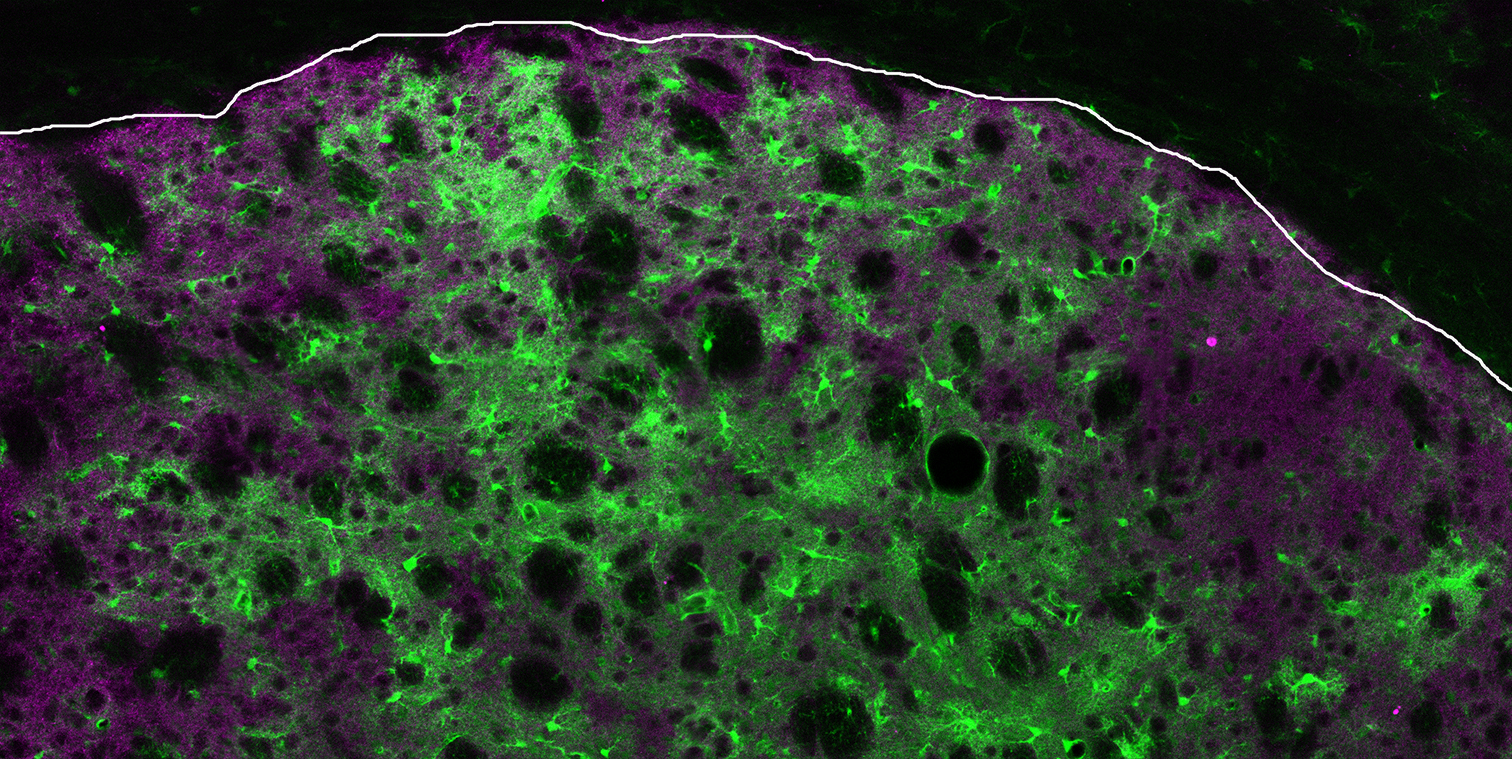

Supplement: Figure 1—source data 3. [file elife-75636-fig1-data3.zip › Fig1 source data 3 for Fig1 F&G/20X/STR shscramble 2M MZ2 GFP+TH.jpg]

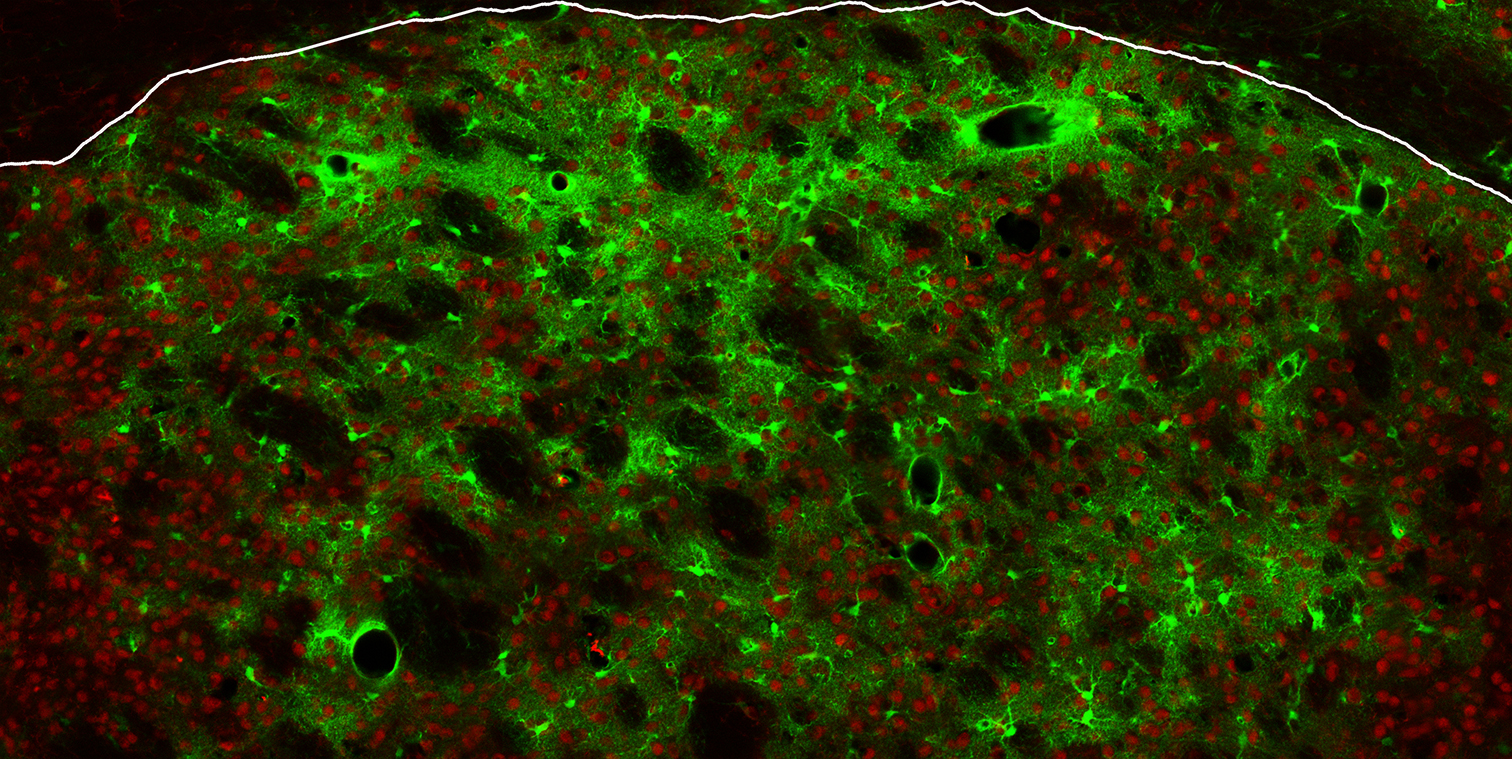

Supplement: Figure 1—source data 3. [file elife-75636-fig1-data3.zip › Fig1 source data 3 for Fig1 F&G/20X/STR shscramble 2M MZ3 GFP+neun.jpg]

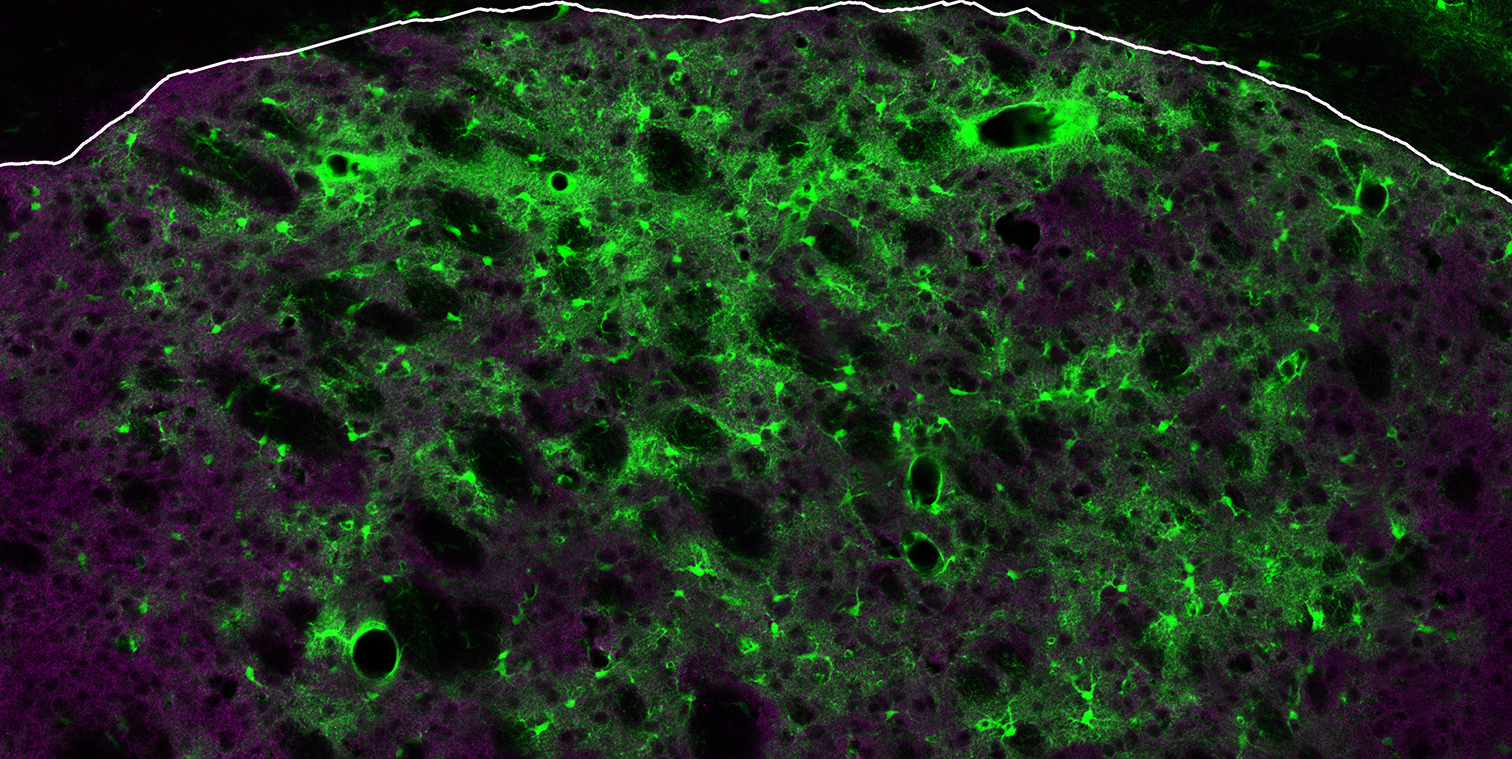

Supplement: Figure 1—source data 3. [file elife-75636-fig1-data3.zip › Fig1 source data 3 for Fig1 F&G/20X/STR shscramble 2M MZ3 GFP+TH.jpg]

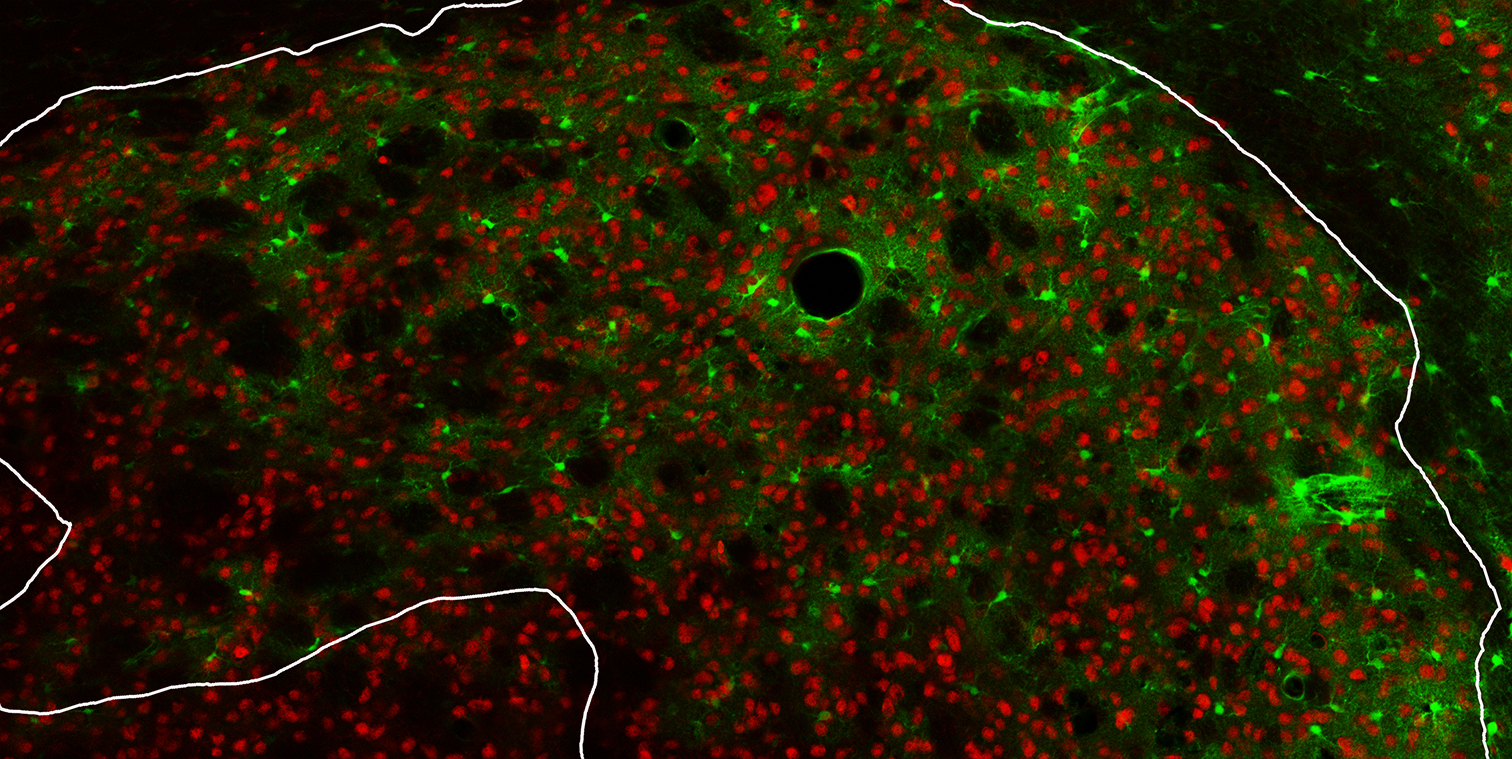

Supplement: Figure 1—source data 3. [file elife-75636-fig1-data3.zip › Fig1 source data 3 for Fig1 F&G/20X/STR shscramble 3M MZ1 GFP+neun.jpg]
