# Supplementary material for: Repressing PTBP1 fails to convert reactive astrocytes to dopaminergic neurons in a 6-hydroxydopamine mouse model of Parkinson’s disease
Source: eLife. 2022 May 10;11:e75636. doi: 10.7554/eLife.75636 (PMC9208759; doi:10.7554/eLife.75636)
Supplement: Figure 1—source data 2. [file elife-75636-fig1-data2.zip › Fig1 source data 2 for Fig1 D&E/description of source data for Fig1D.docx]

Brain slices co-stained GFP (green) with TH (purple) or NeuN (red) at indicated time points after AAV-sh*Ptbp1* or AAV-shscramble delivery in the substantia nigra.
