# Supplementary figures and images for: Repressing PTBP1 fails to convert reactive astrocytes to dopaminergic neurons in a 6-hydroxydopamine mouse model of Parkinson’s disease (part 2 of 4)
Source: eLife. 2022 May 10;11:e75636. doi: 10.7554/eLife.75636 (PMC9208759; doi:10.7554/eLife.75636)

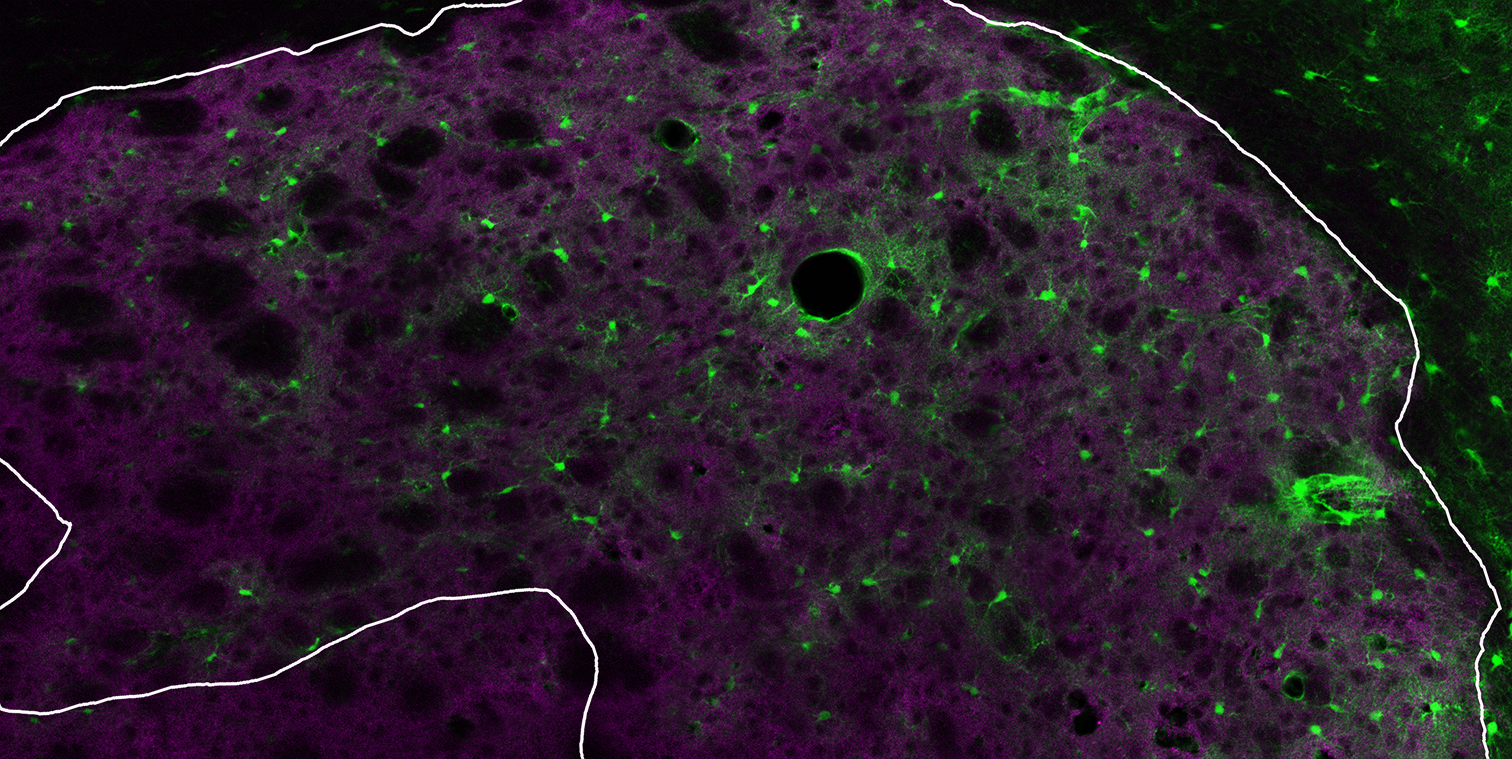

Supplement: Figure 1—source data 3. [file elife-75636-fig1-data3.zip › Fig1 source data 3 for Fig1 F&G/20X/STR shscramble 3M MZ1 GFP+TH.jpg]

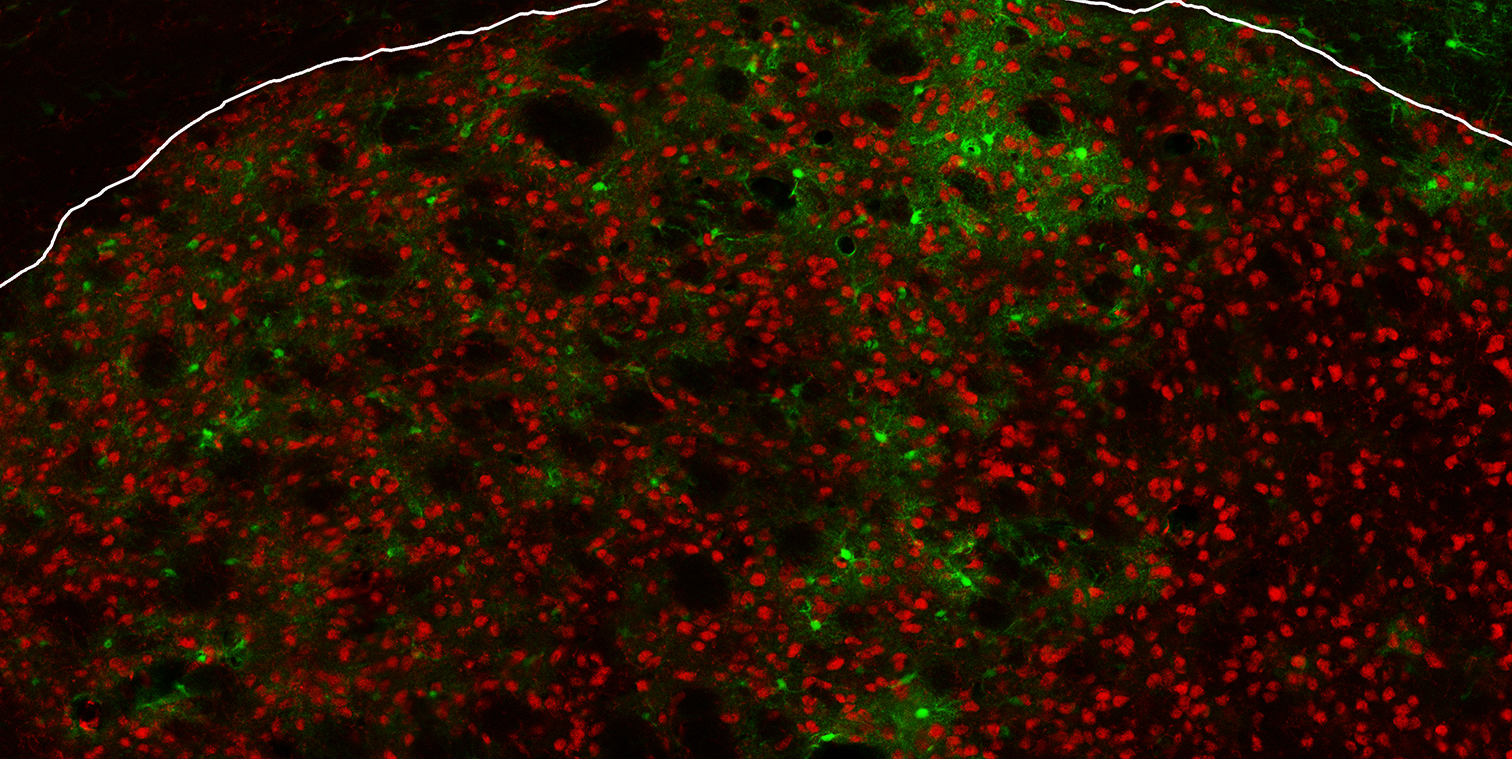

Supplement: Figure 1—source data 3. [file elife-75636-fig1-data3.zip › Fig1 source data 3 for Fig1 F&G/20X/STR shscramble 3M MZ2 GFP+neun.jpg]

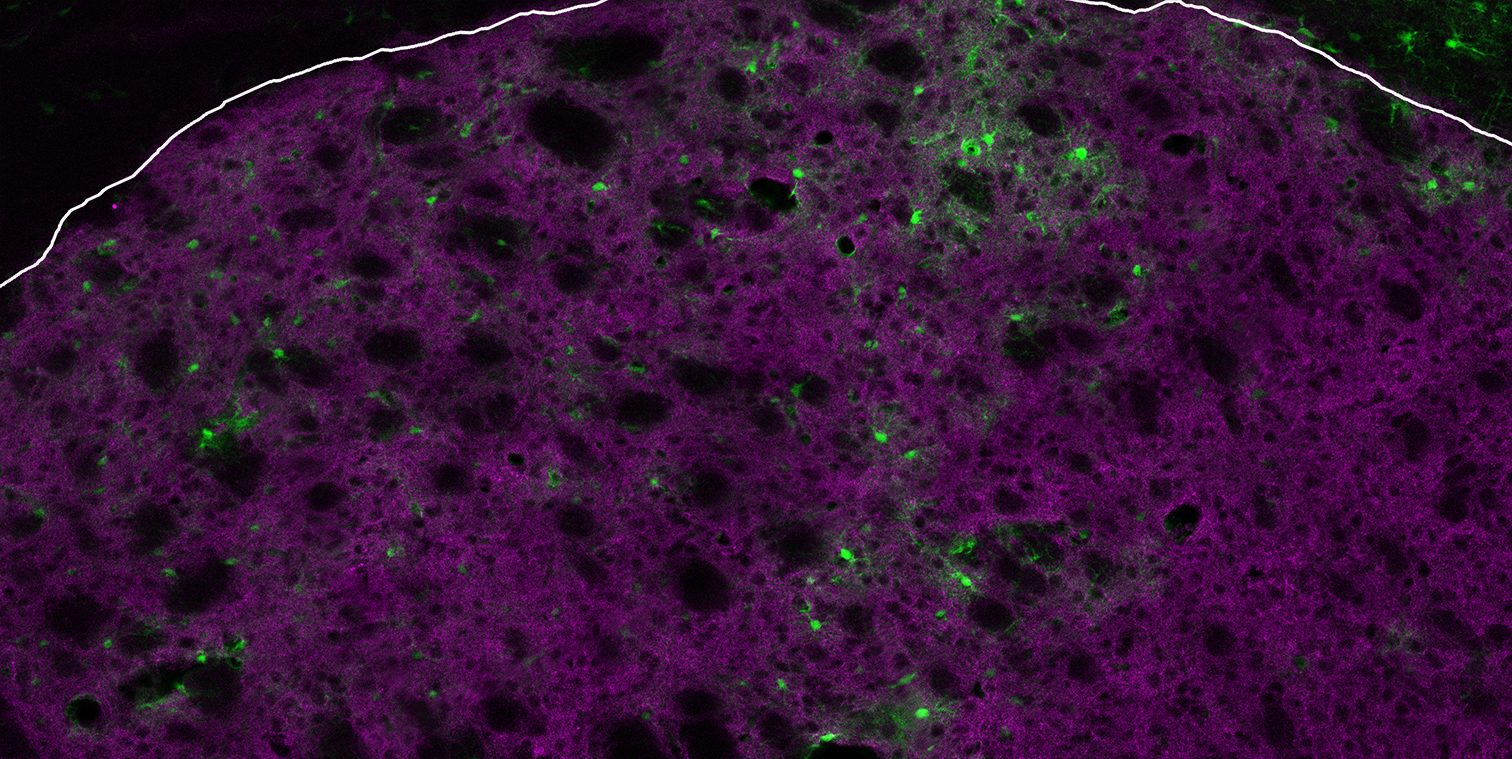

Supplement: Figure 1—source data 3. [file elife-75636-fig1-data3.zip › Fig1 source data 3 for Fig1 F&G/20X/STR shscramble 3M MZ2 GFP+TH.jpg]

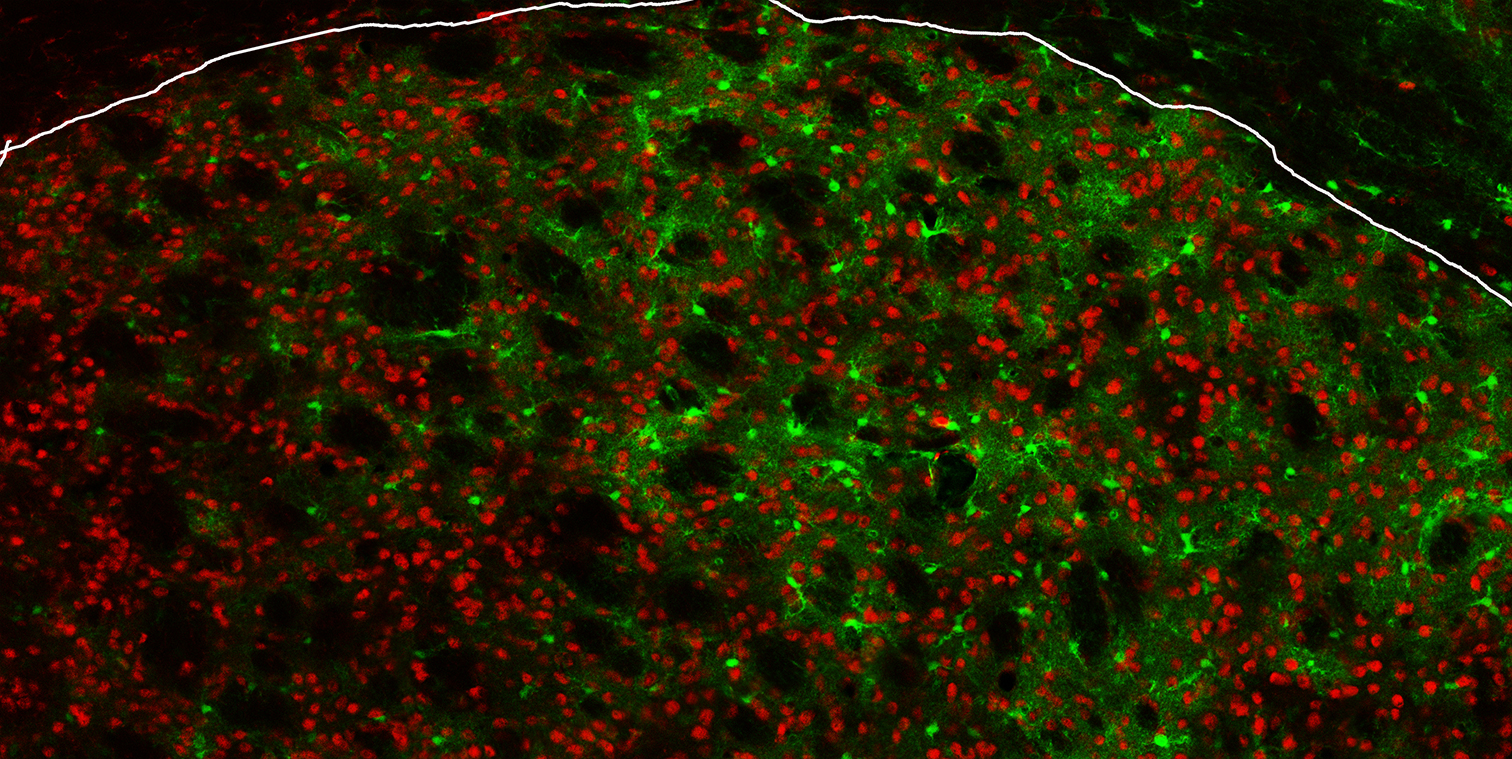

Supplement: Figure 1—source data 3. [file elife-75636-fig1-data3.zip › Fig1 source data 3 for Fig1 F&G/20X/STR shscramble 3M MZ3 GFP+neun.jpg]

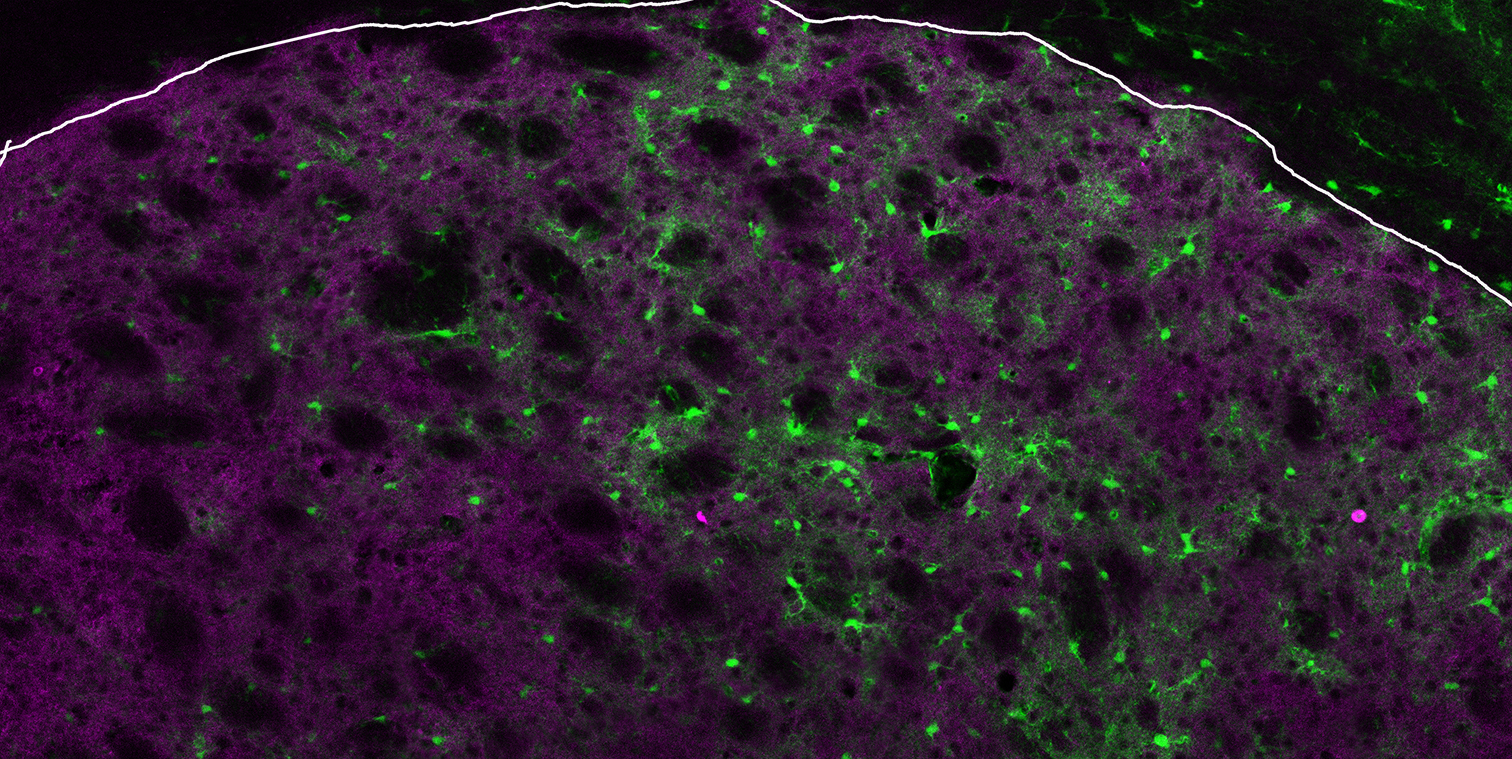

Supplement: Figure 1—source data 3. [file elife-75636-fig1-data3.zip › Fig1 source data 3 for Fig1 F&G/20X/STR shscramble 3M MZ3 GFP+TH.jpg]

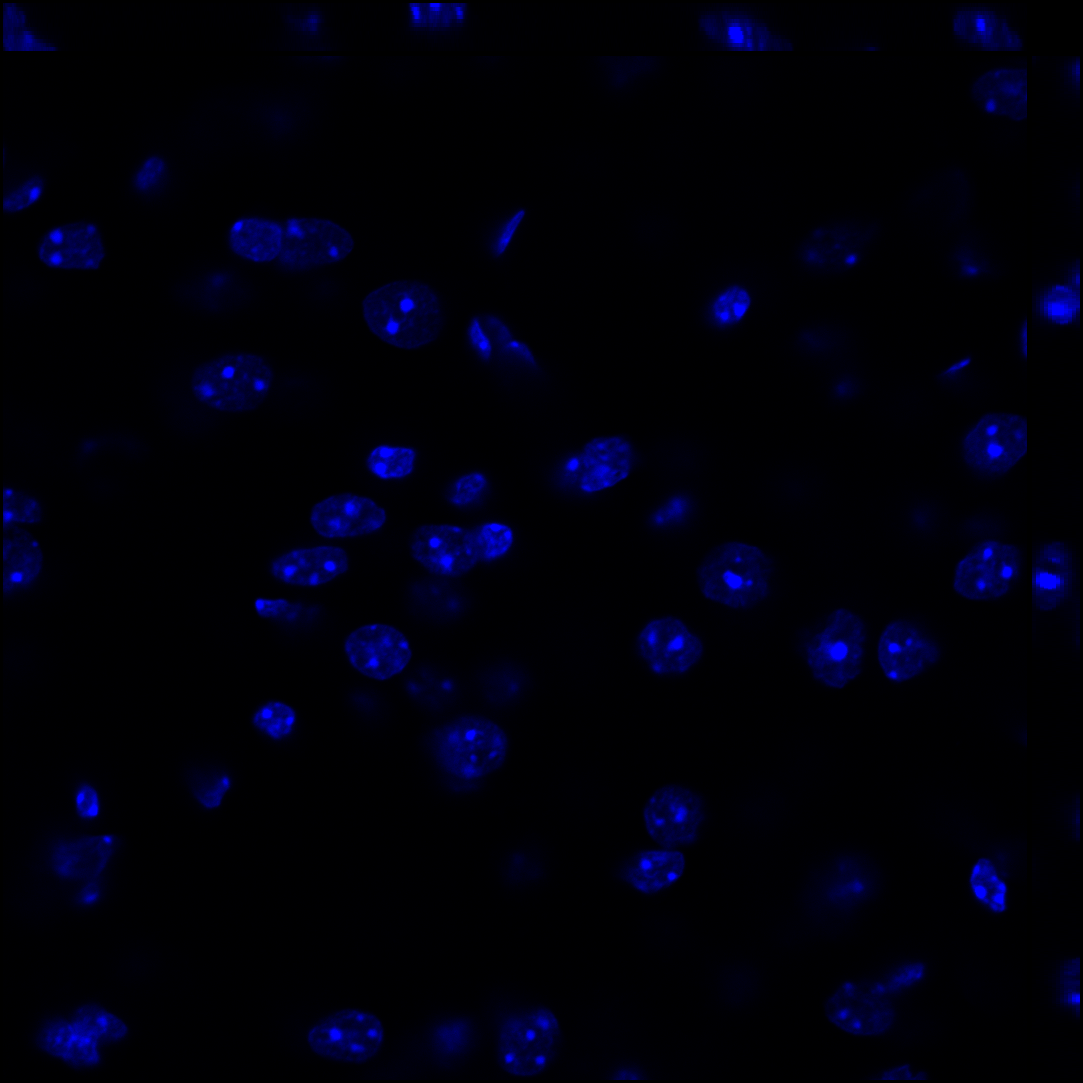

Supplement: Figure 1—source data 3. [file elife-75636-fig1-data3.zip › Fig1 source data 3 for Fig1 F&G/3D/STR 1M MZ1-2/Untitled21_c1.tif]

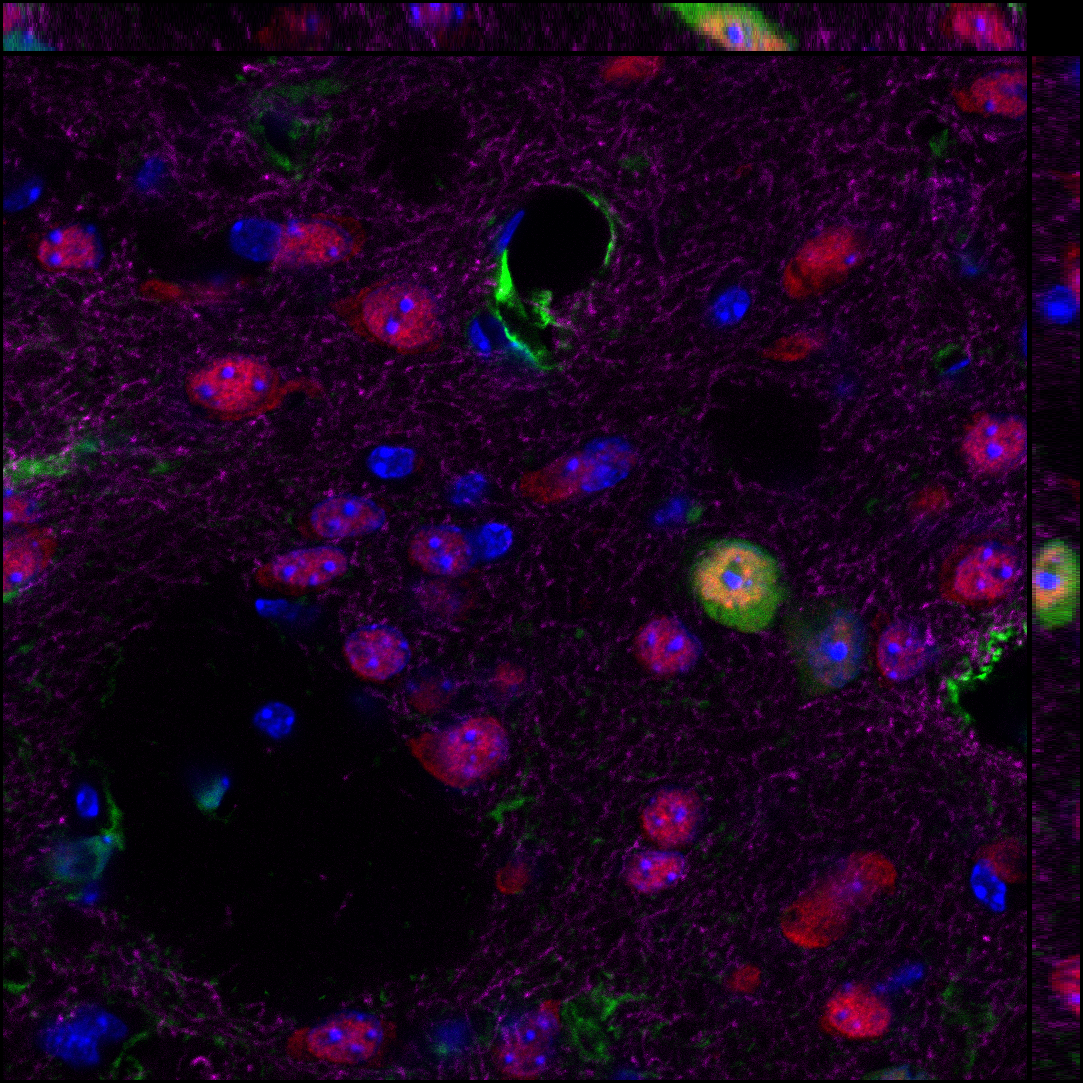

Supplement: Figure 1—source data 3. [file elife-75636-fig1-data3.zip › Fig1 source data 3 for Fig1 F&G/3D/STR 1M MZ1-2/Untitled21_c1+2+3+4.tif]

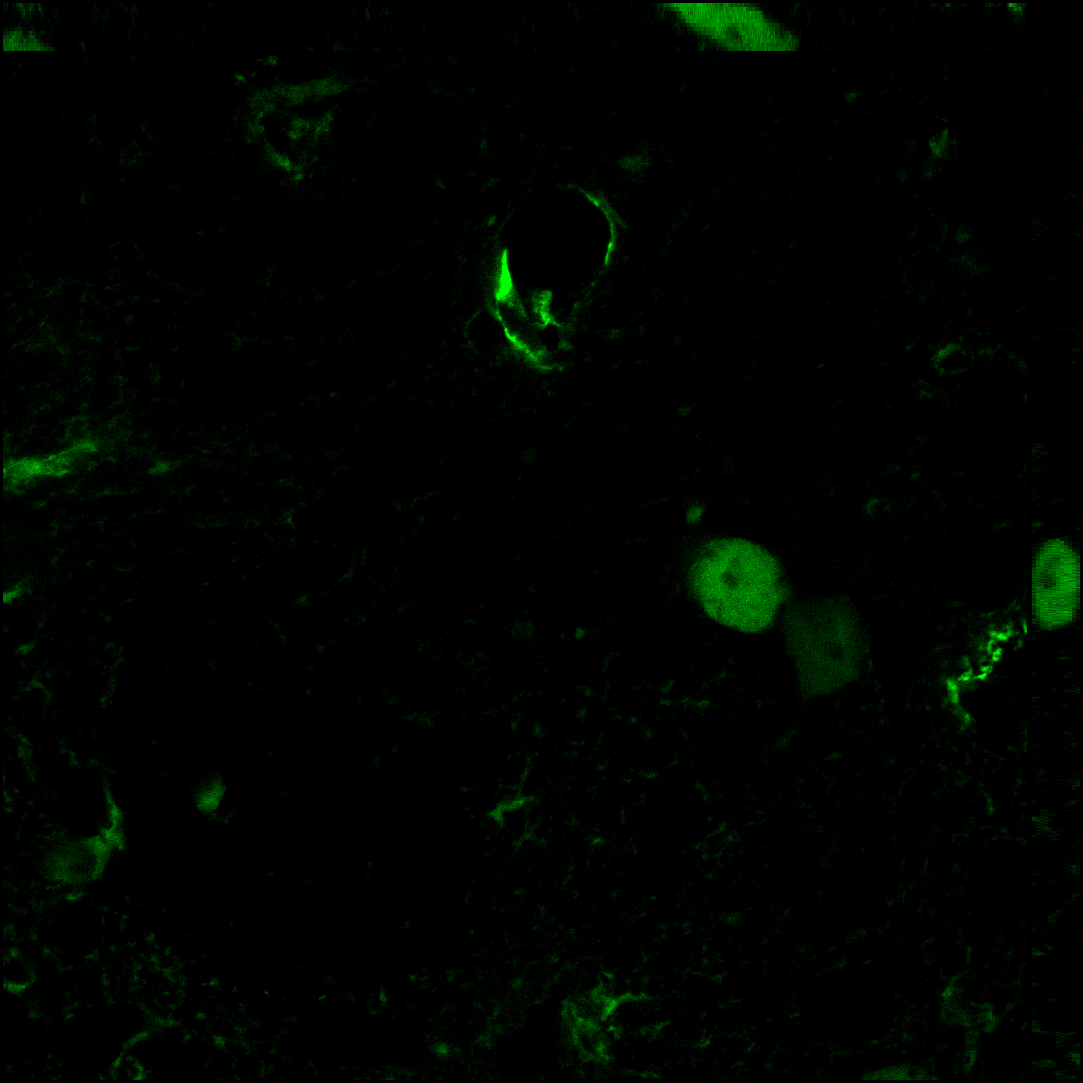

Supplement: Figure 1—source data 3. [file elife-75636-fig1-data3.zip › Fig1 source data 3 for Fig1 F&G/3D/STR 1M MZ1-2/Untitled21_c2.tif]

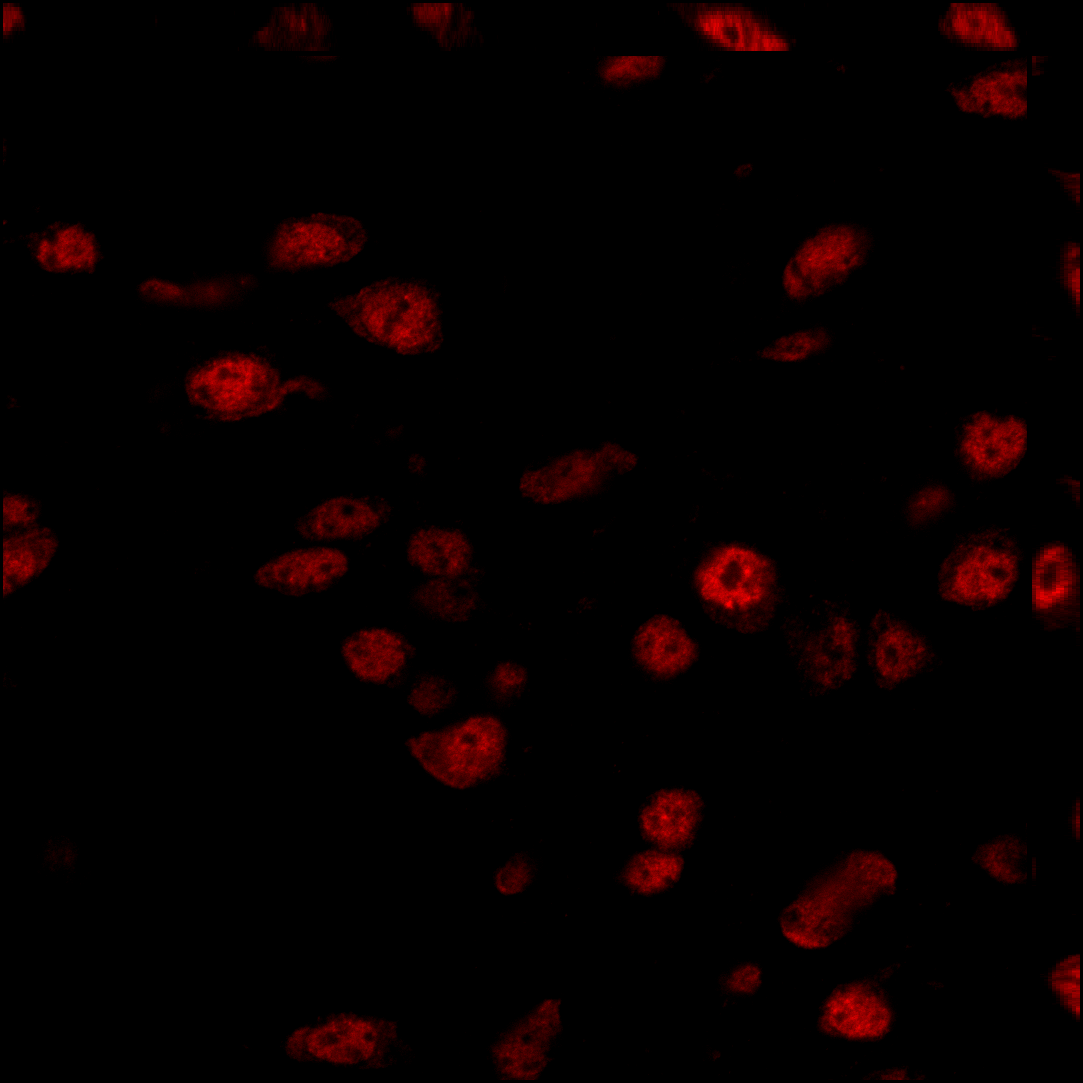

Supplement: Figure 1—source data 3. [file elife-75636-fig1-data3.zip › Fig1 source data 3 for Fig1 F&G/3D/STR 1M MZ1-2/Untitled21_c3.tif]

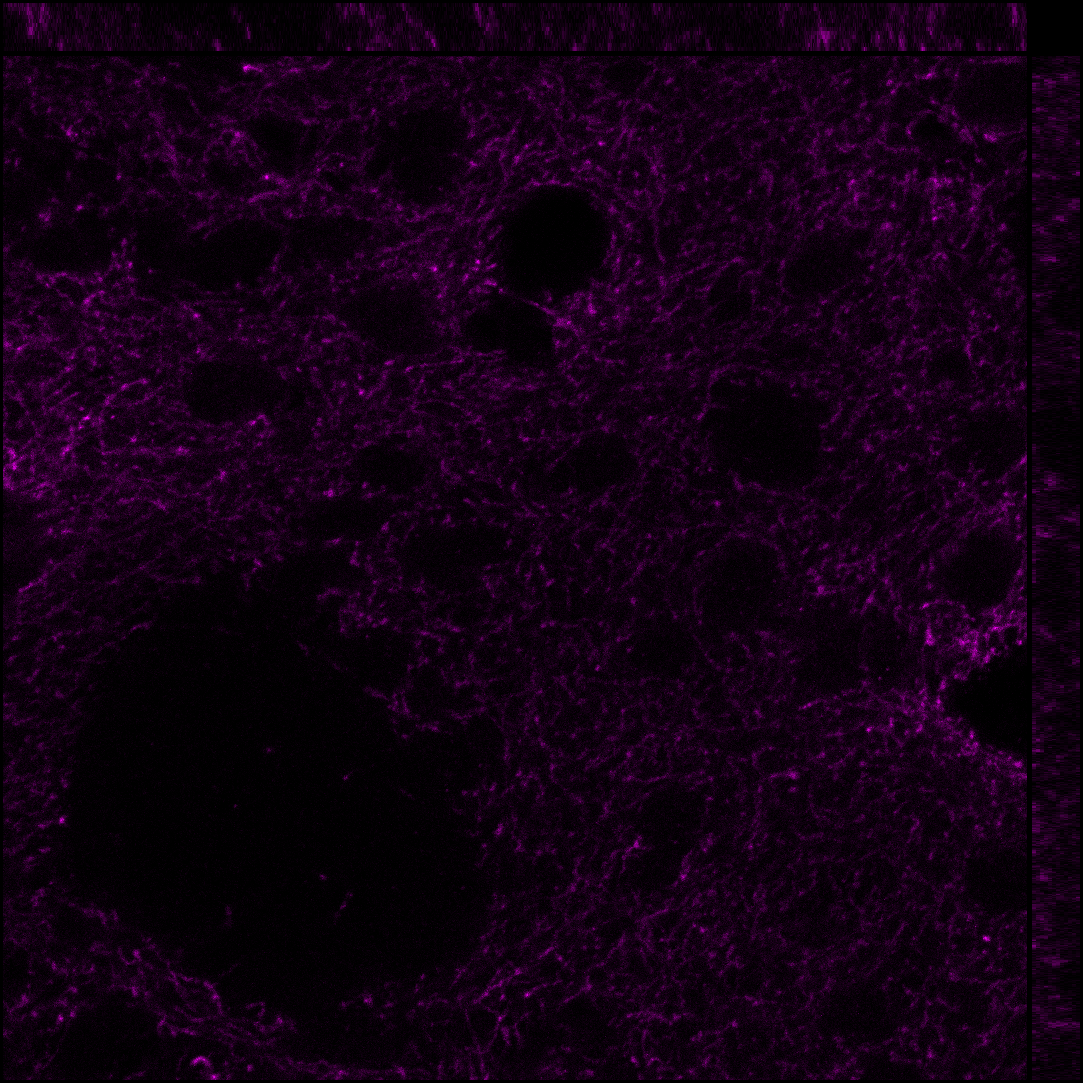

Supplement: Figure 1—source data 3. [file elife-75636-fig1-data3.zip › Fig1 source data 3 for Fig1 F&G/3D/STR 1M MZ1-2/Untitled21_c4.tif]

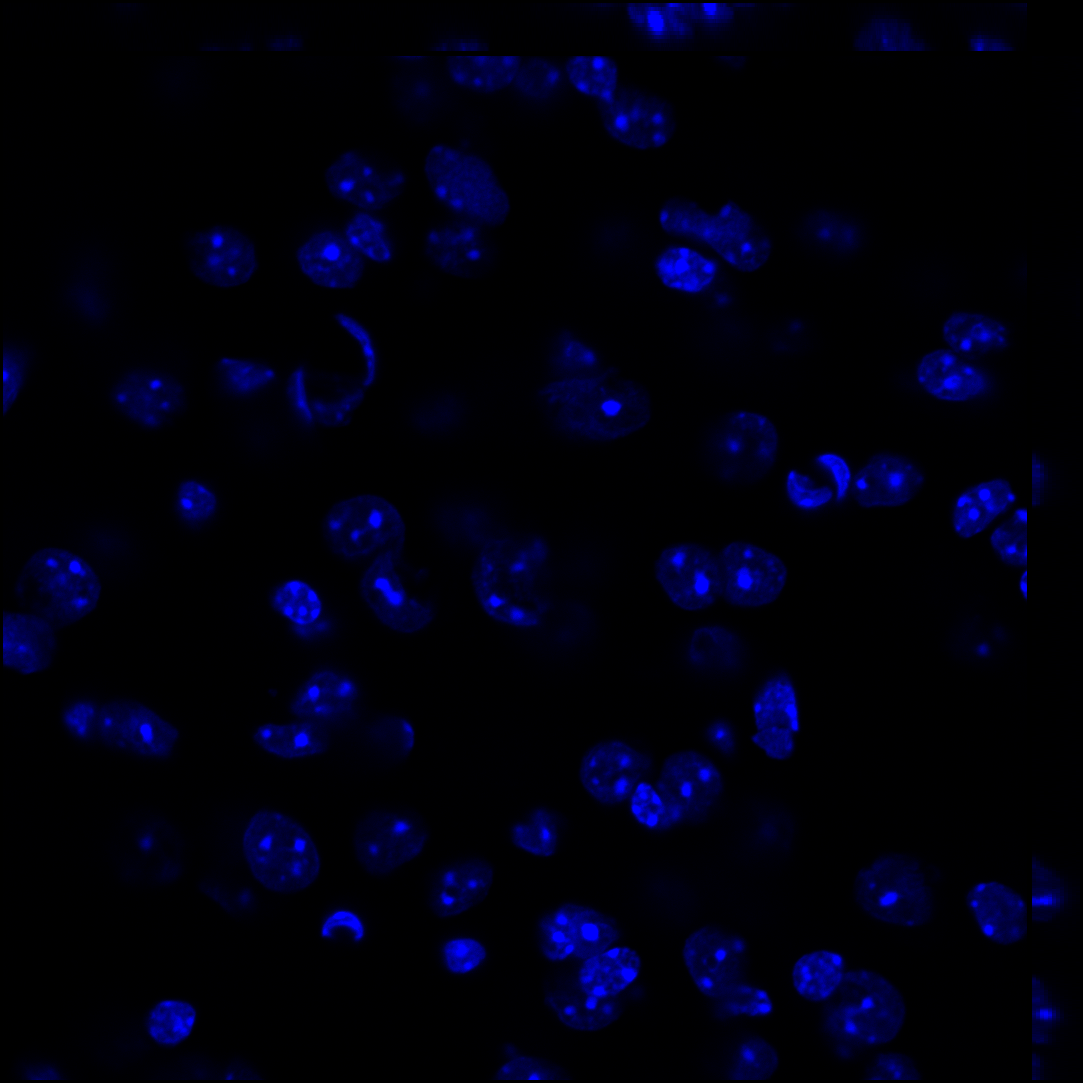

Supplement: Figure 1—source data 3. [file elife-75636-fig1-data3.zip › Fig1 source data 3 for Fig1 F&G/3D/STR 2M MZ3-3/Untitled23_c1.tif]

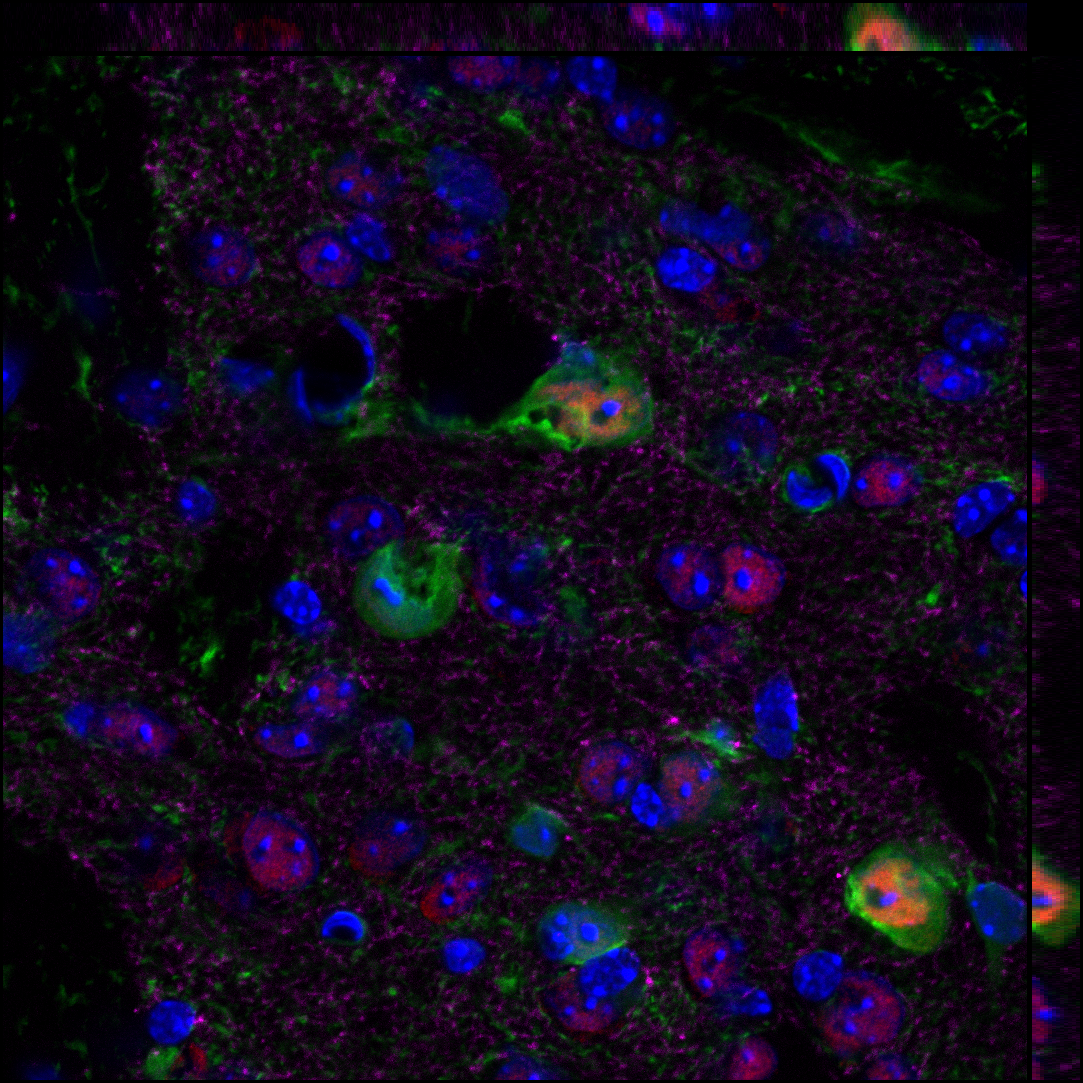

Supplement: Figure 1—source data 3. [file elife-75636-fig1-data3.zip › Fig1 source data 3 for Fig1 F&G/3D/STR 2M MZ3-3/Untitled23_c1+2+3+4.tif]

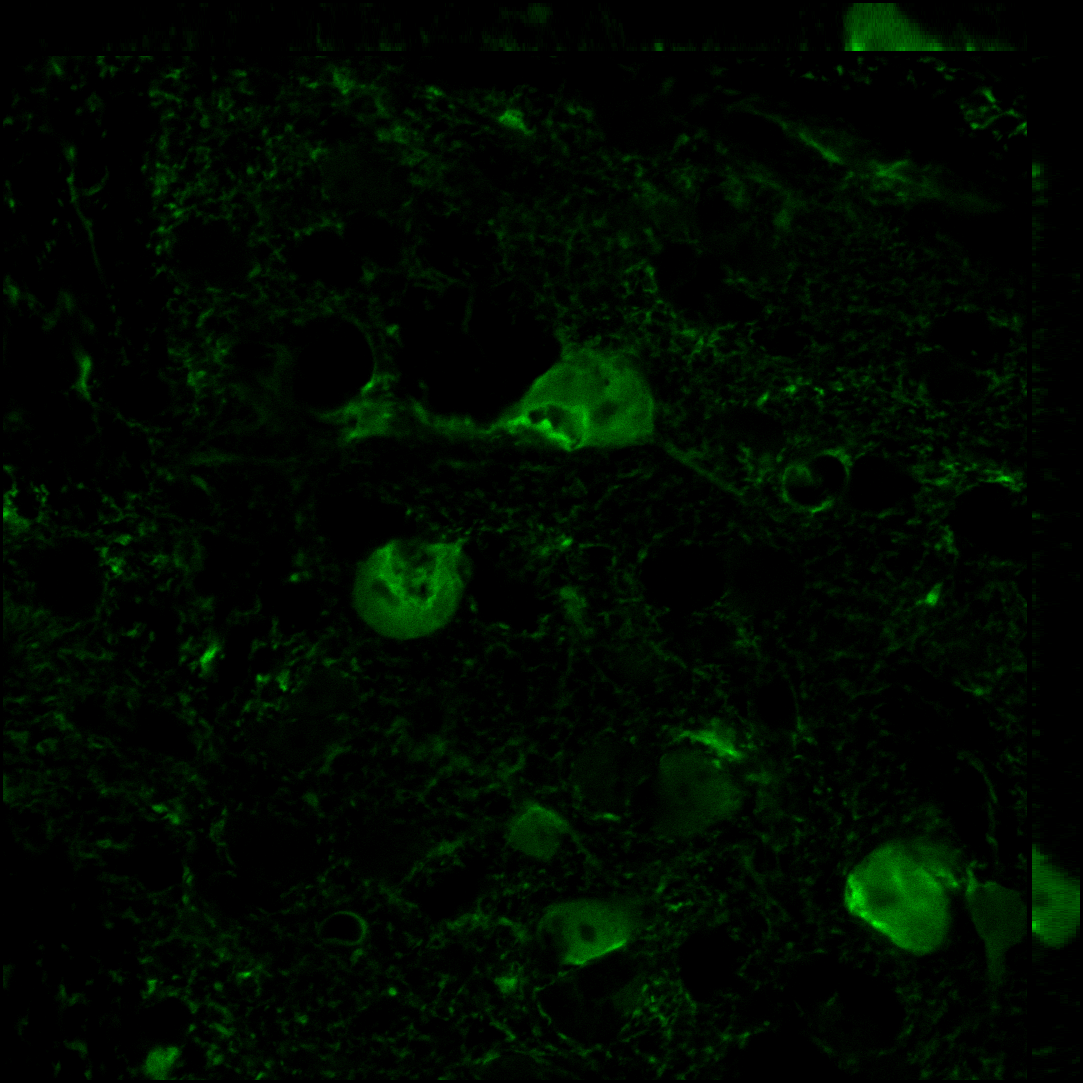

Supplement: Figure 1—source data 3. [file elife-75636-fig1-data3.zip › Fig1 source data 3 for Fig1 F&G/3D/STR 2M MZ3-3/Untitled23_c2.tif]

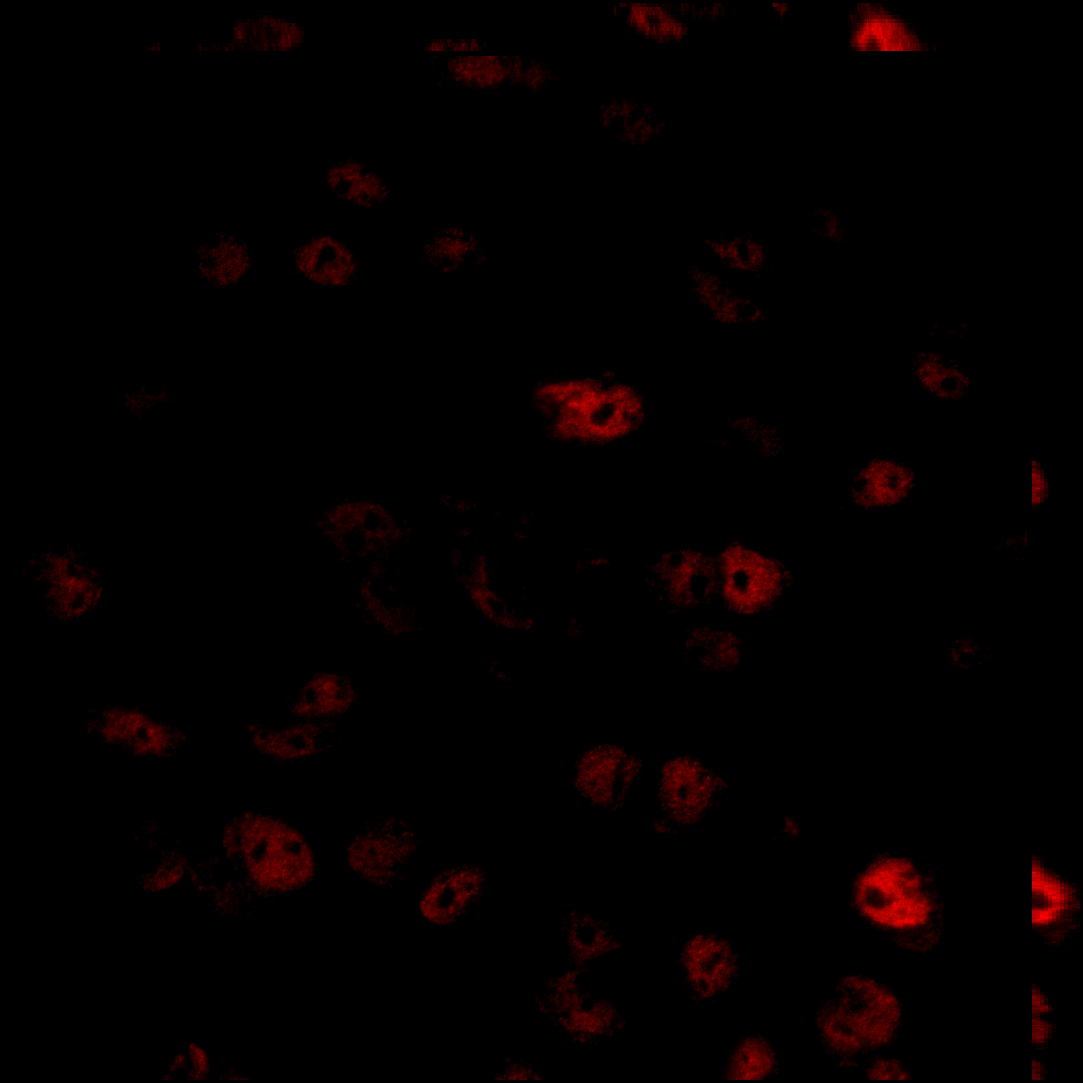

Supplement: Figure 1—source data 3. [file elife-75636-fig1-data3.zip › Fig1 source data 3 for Fig1 F&G/3D/STR 2M MZ3-3/Untitled23_c3.tif]

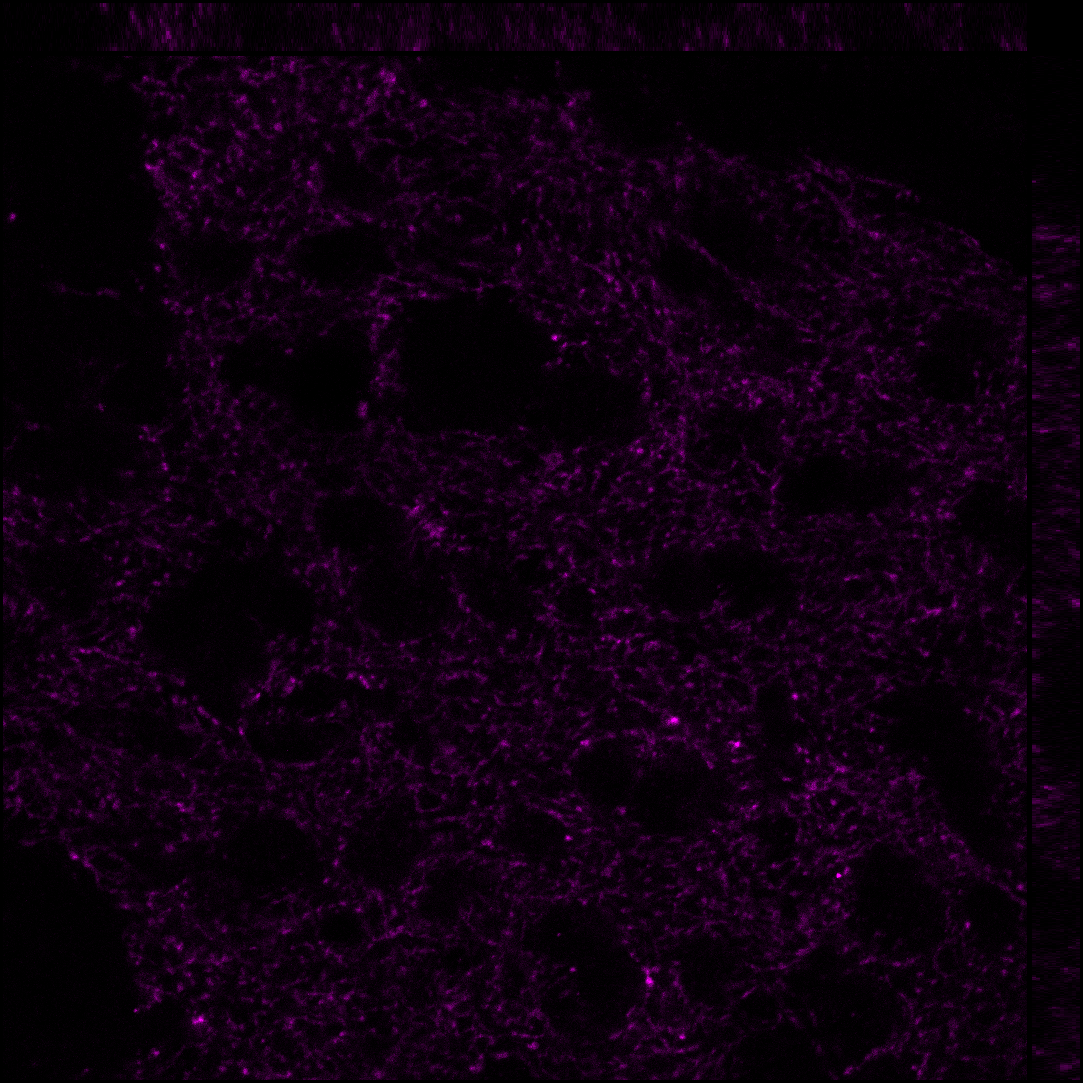

Supplement: Figure 1—source data 3. [file elife-75636-fig1-data3.zip › Fig1 source data 3 for Fig1 F&G/3D/STR 2M MZ3-3/Untitled23_c4.tif]

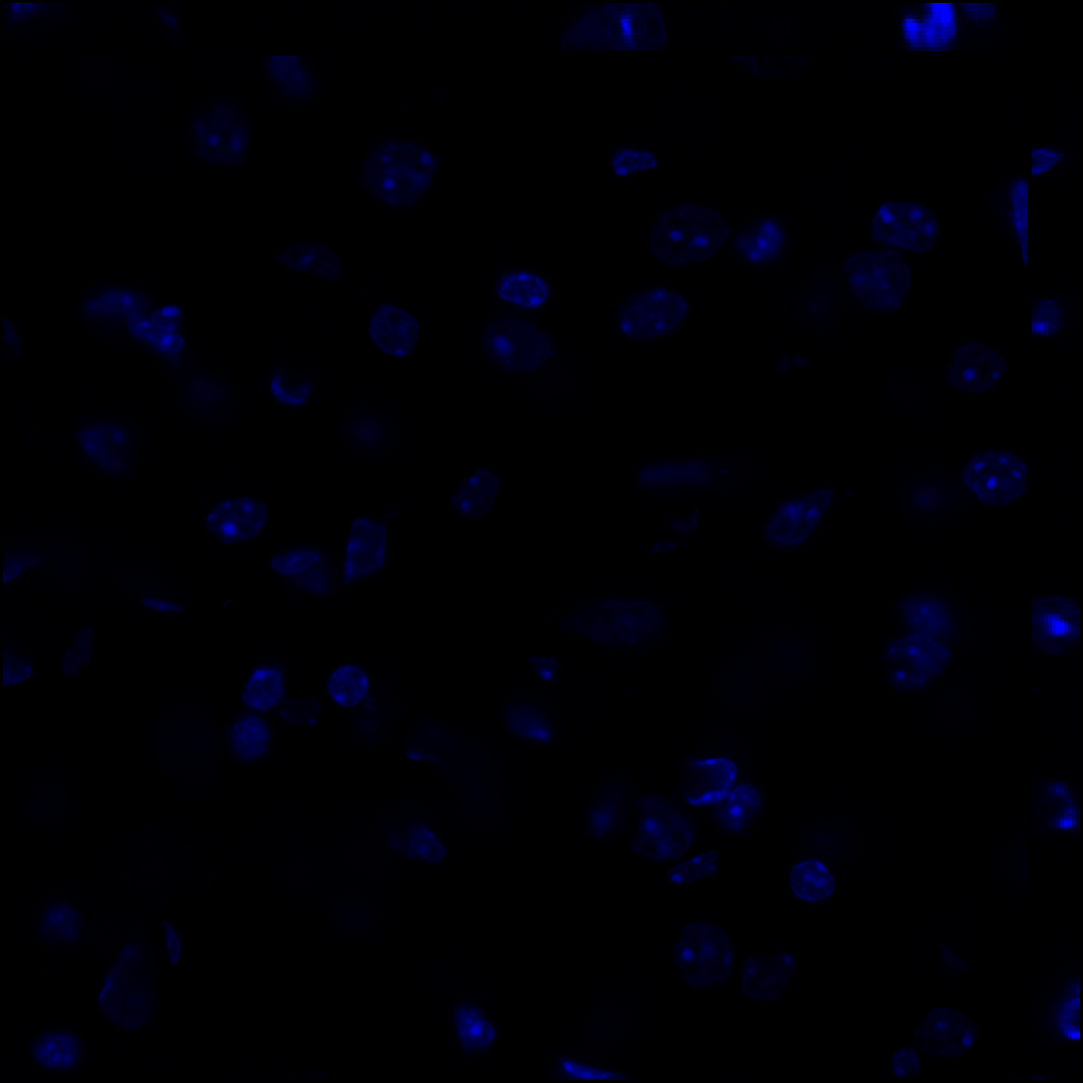

Supplement: Figure 1—source data 3. [file elife-75636-fig1-data3.zip › Fig1 source data 3 for Fig1 F&G/3D/STR 3M MZ3-2/Untitled25_c1.tif]

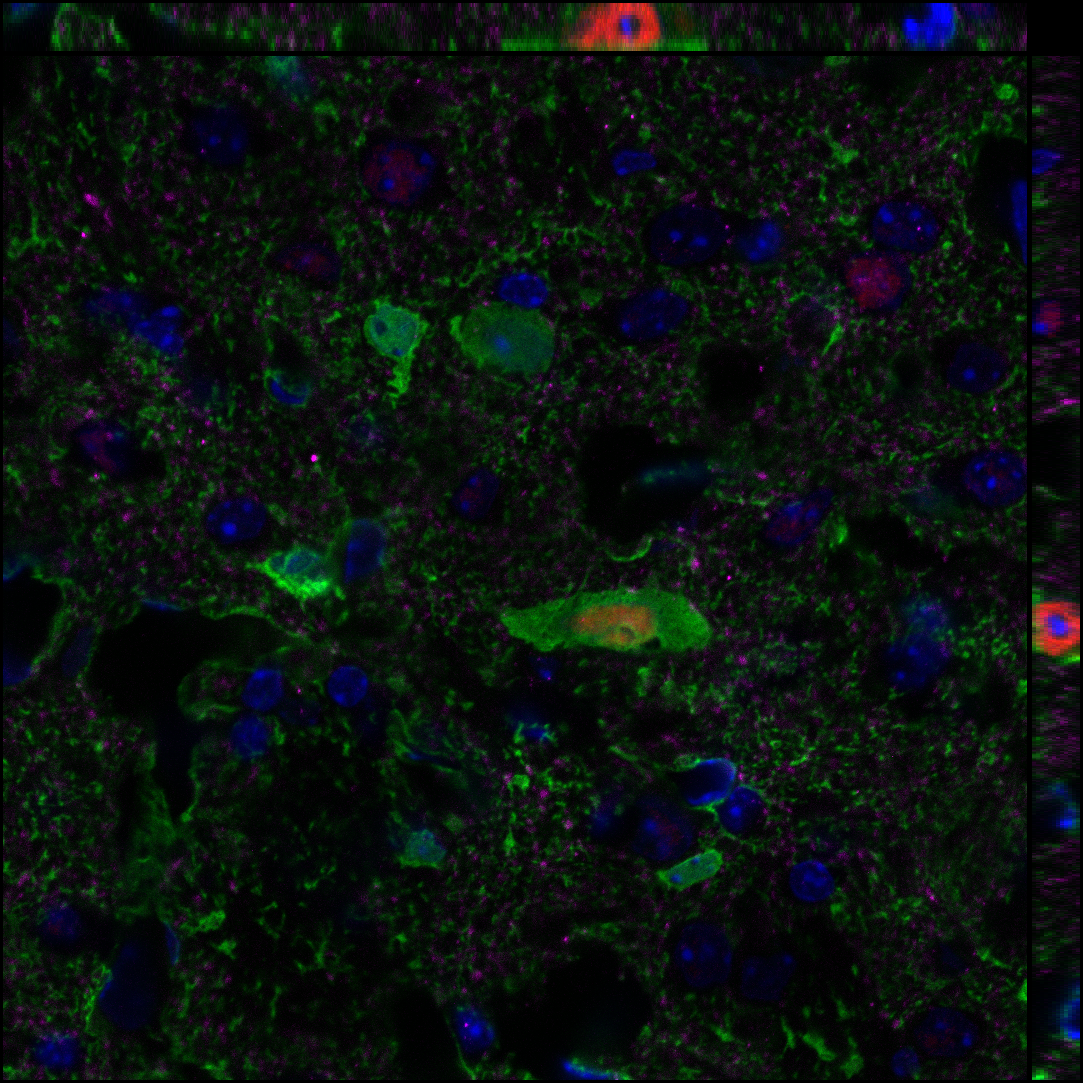

Supplement: Figure 1—source data 3. [file elife-75636-fig1-data3.zip › Fig1 source data 3 for Fig1 F&G/3D/STR 3M MZ3-2/Untitled25_c1+2+3+4.tif]

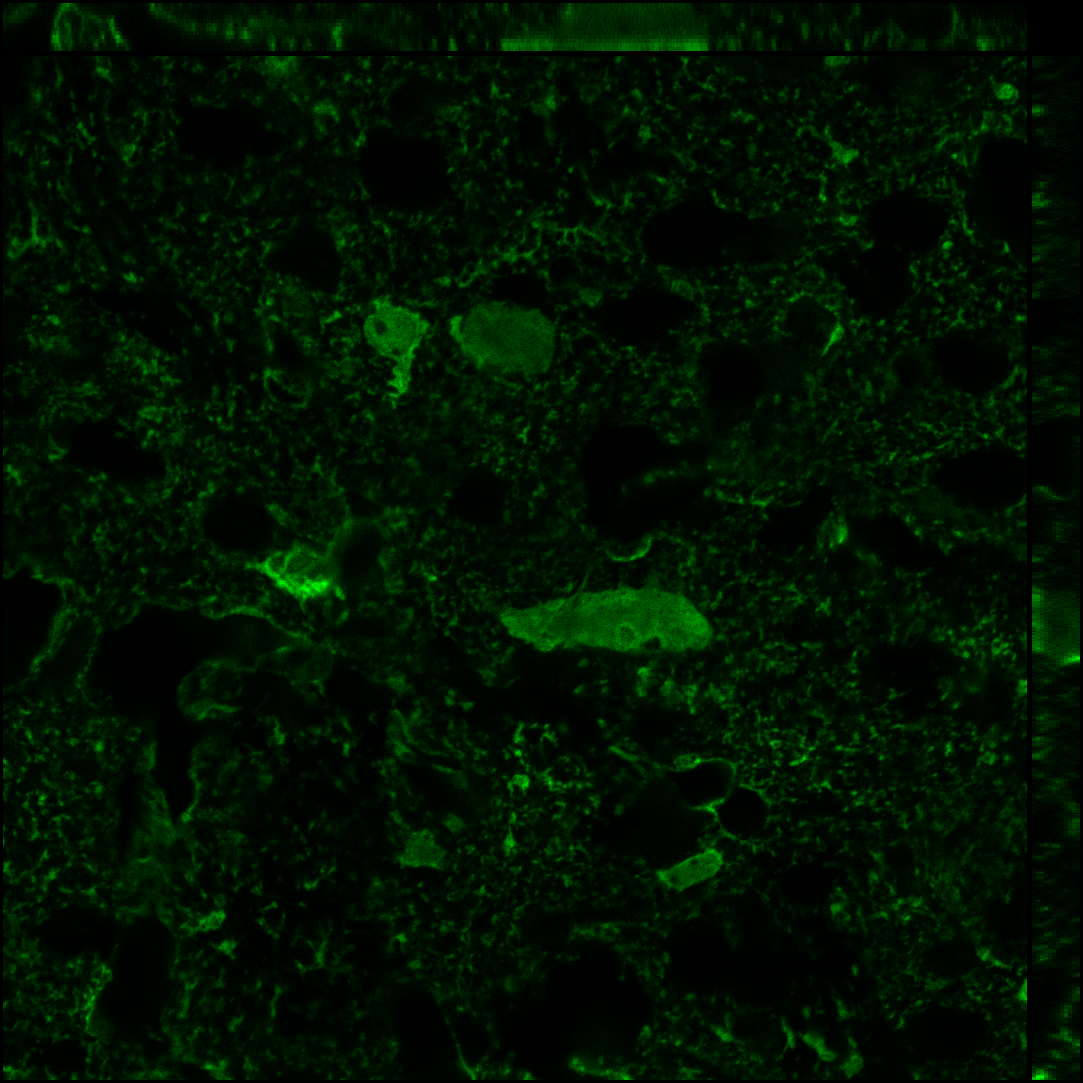

Supplement: Figure 1—source data 3. [file elife-75636-fig1-data3.zip › Fig1 source data 3 for Fig1 F&G/3D/STR 3M MZ3-2/Untitled25_c2.tif]

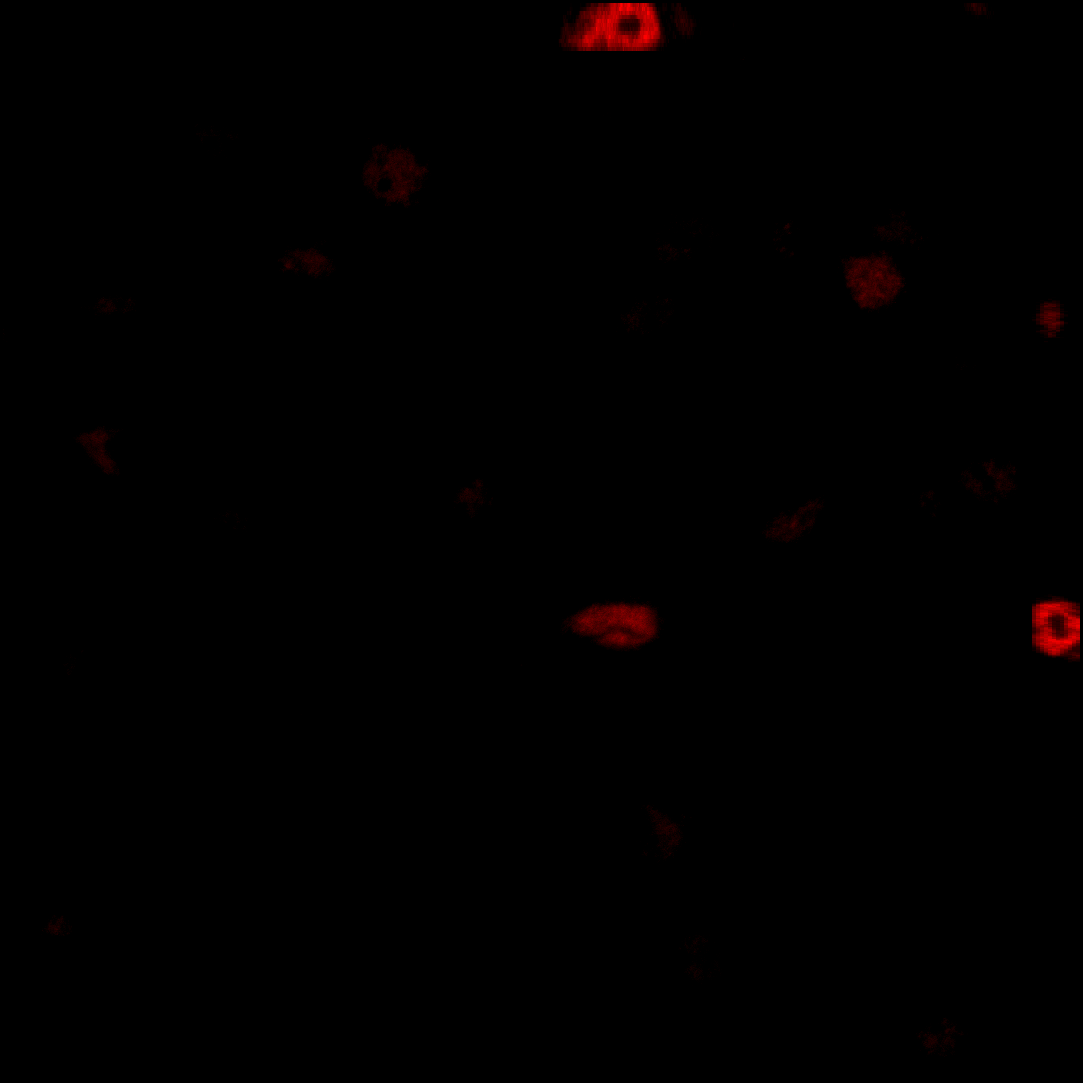

Supplement: Figure 1—source data 3. [file elife-75636-fig1-data3.zip › Fig1 source data 3 for Fig1 F&G/3D/STR 3M MZ3-2/Untitled25_c3.tif]

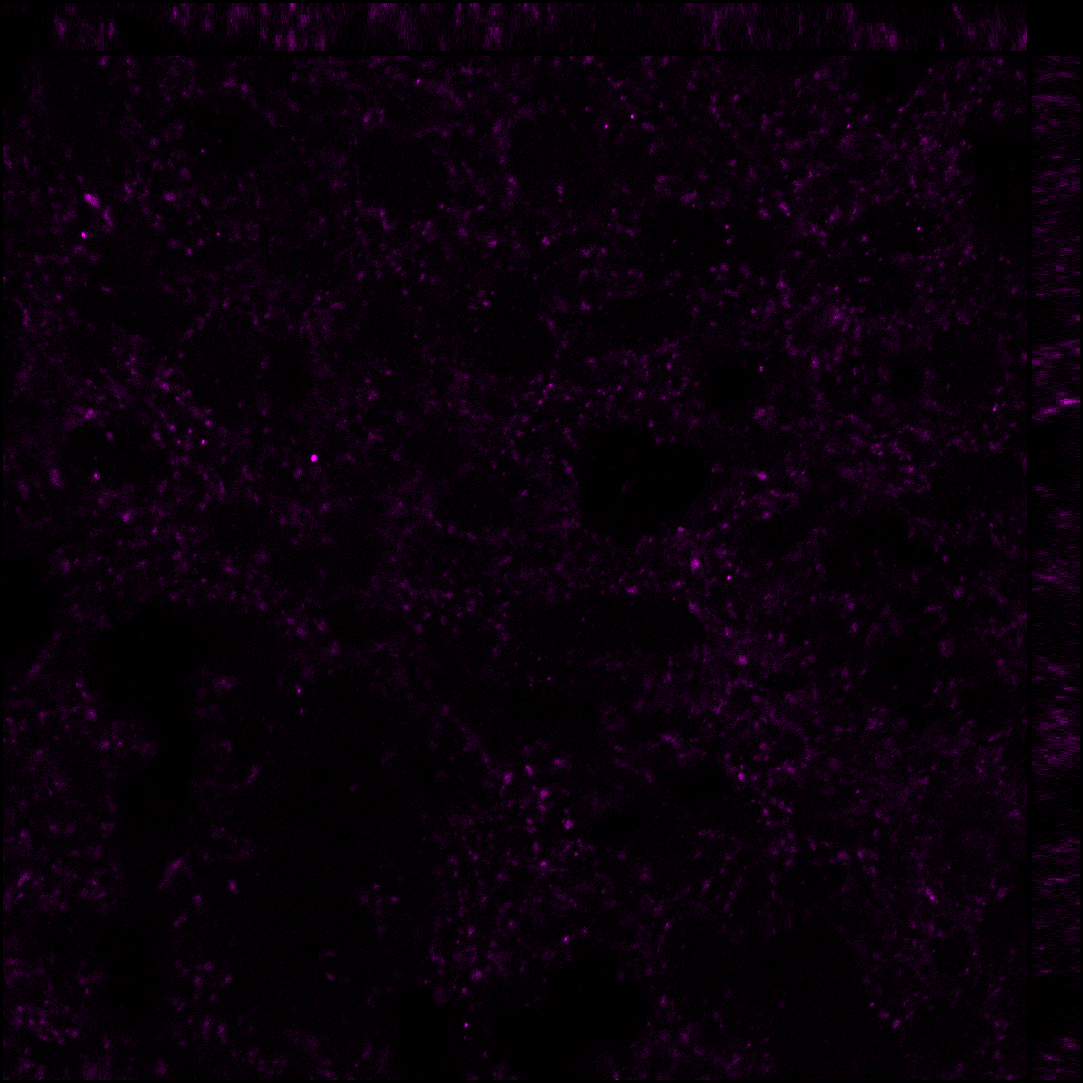

Supplement: Figure 1—source data 3. [file elife-75636-fig1-data3.zip › Fig1 source data 3 for Fig1 F&G/3D/STR 3M MZ3-2/Untitled25_c4.tif]

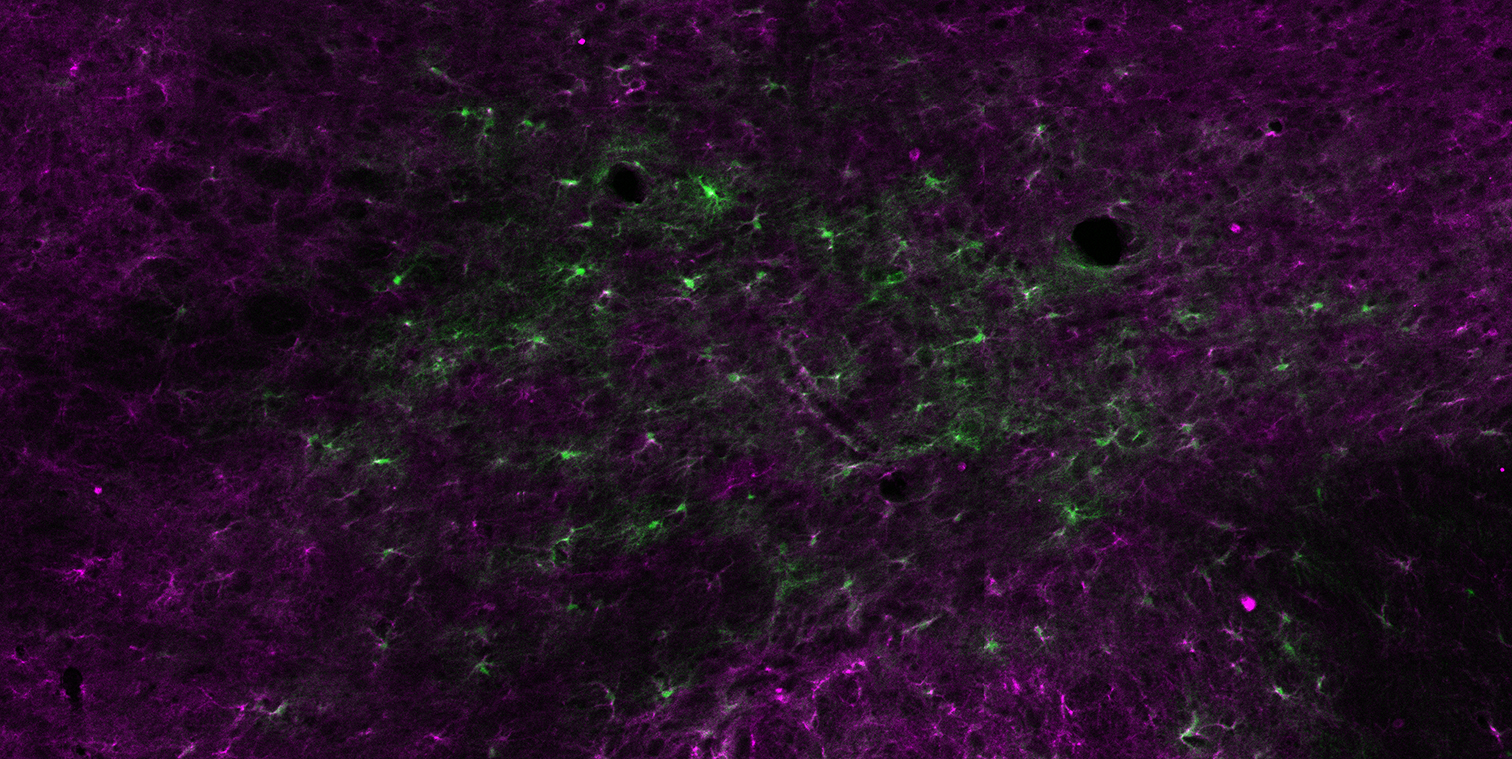

Supplement: Figure 1—figure supplement 1—source data 1. [file elife-75636-fig1-figsupp1-data1.zip › Fig1 source data 4 for Fig1 supplement 1/GFP+ALDOC OR GFP+NeuN/AAV-scramble SN #10 GFP+AldoC.jpg]

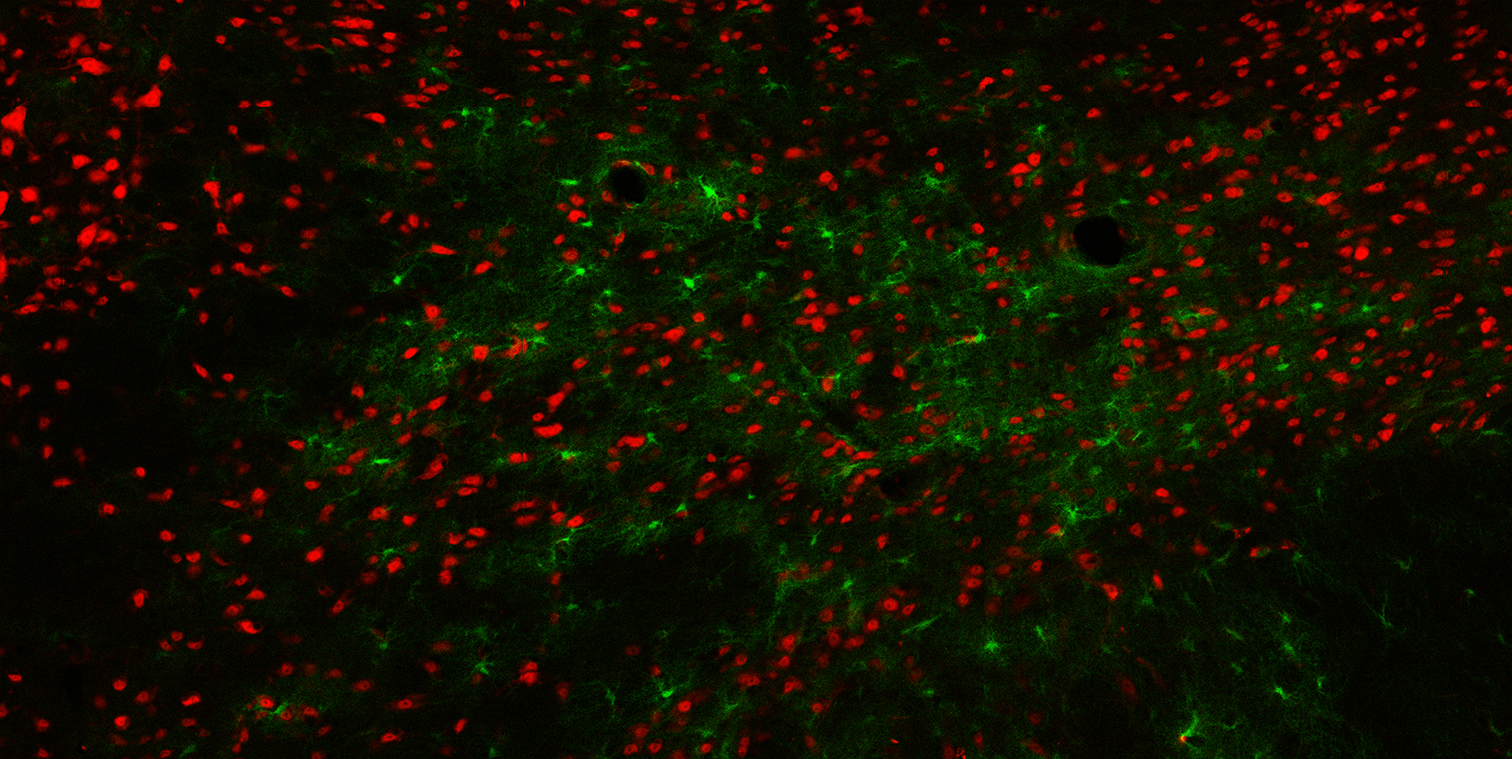

Supplement: Figure 1—figure supplement 1—source data 1. [file elife-75636-fig1-figsupp1-data1.zip › Fig1 source data 4 for Fig1 supplement 1/GFP+ALDOC OR GFP+NeuN/AAV-scramble SN #10 GFP+NeuN.jpg]

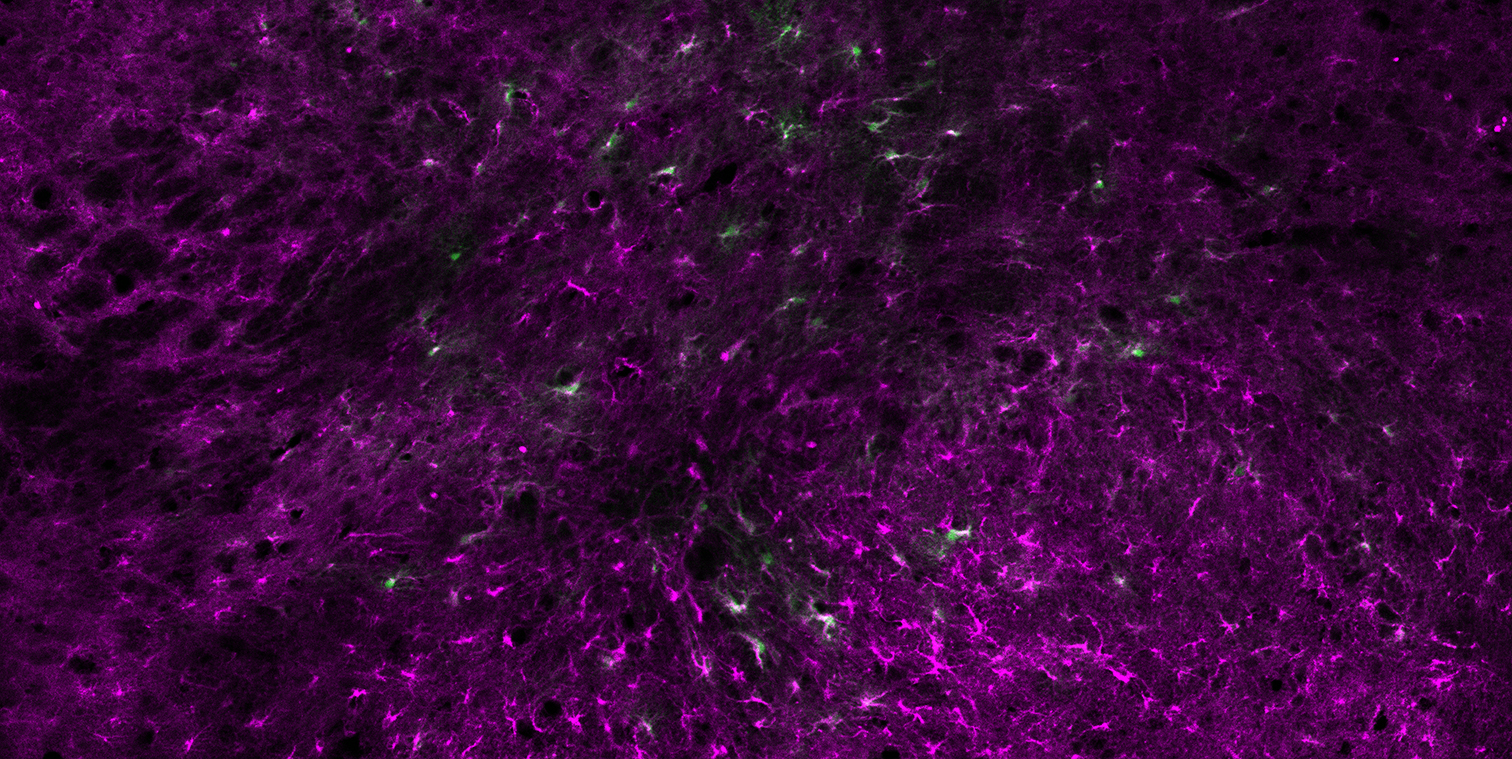

Supplement: Figure 1—figure supplement 1—source data 1. [file elife-75636-fig1-figsupp1-data1.zip › Fig1 source data 4 for Fig1 supplement 1/GFP+ALDOC OR GFP+NeuN/AAV-scramble SN #13 GFP+AldoC.jpg]

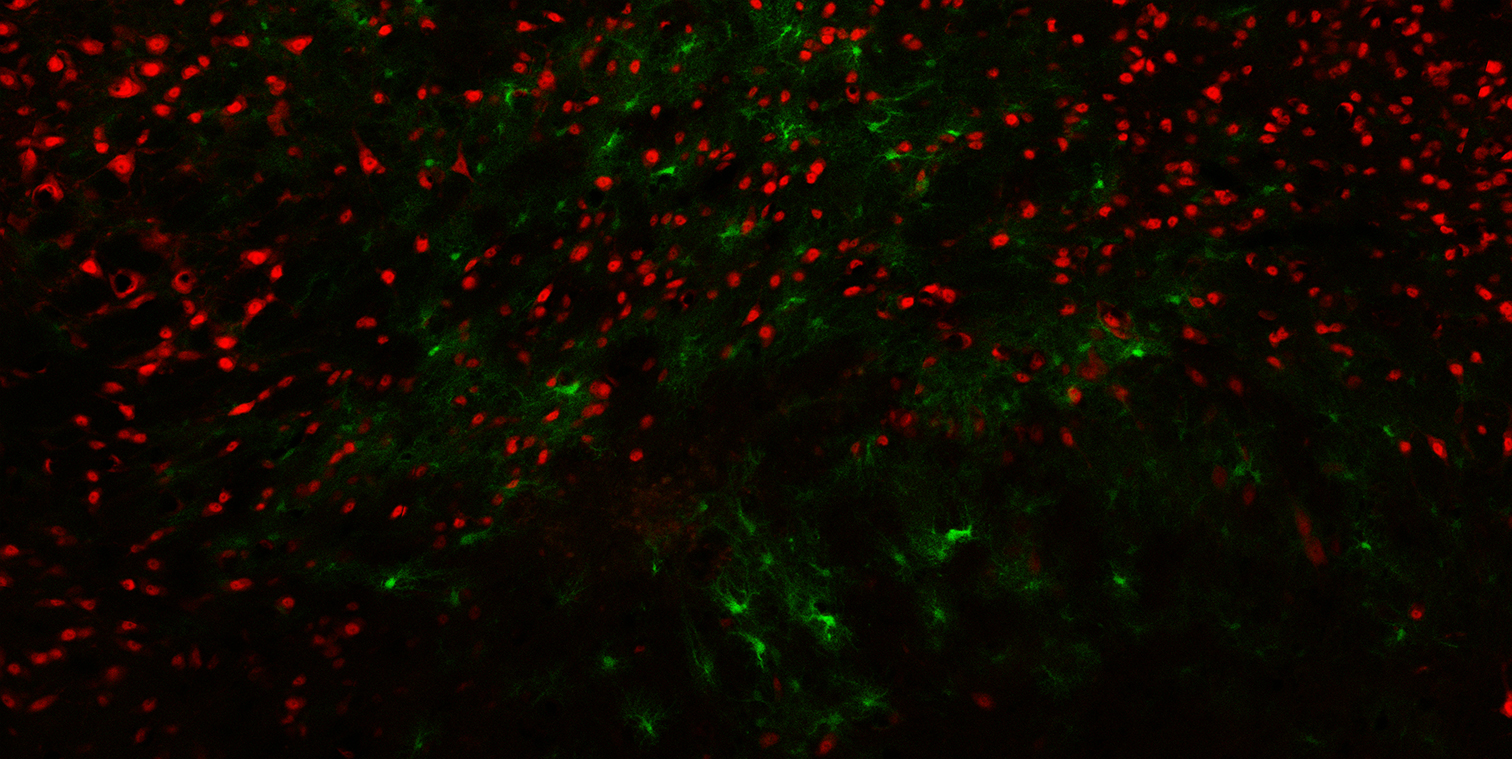

Supplement: Figure 1—figure supplement 1—source data 1. [file elife-75636-fig1-figsupp1-data1.zip › Fig1 source data 4 for Fig1 supplement 1/GFP+ALDOC OR GFP+NeuN/AAV-scramble SN #13 GFP+NeuN.jpg]

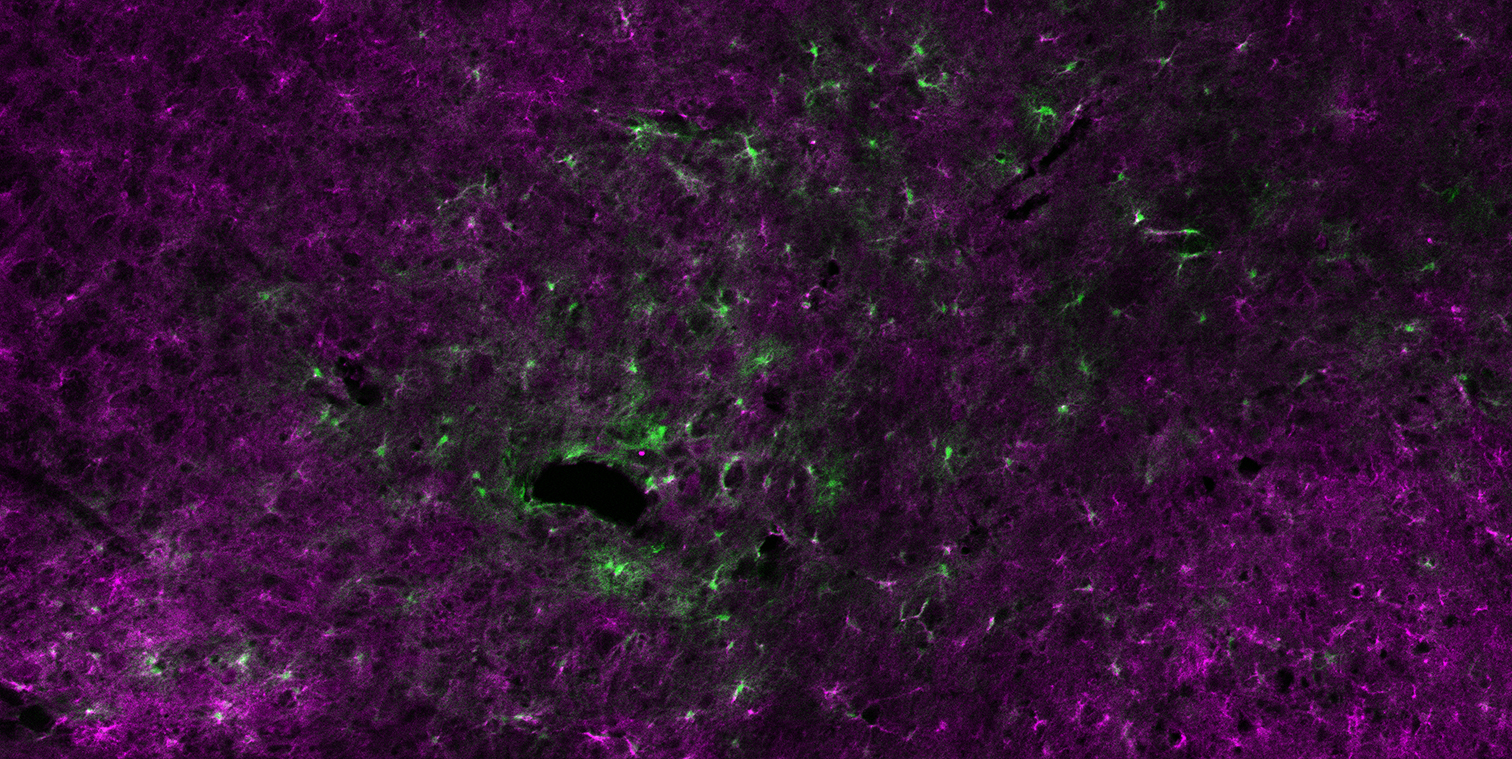

Supplement: Figure 1—figure supplement 1—source data 1. [file elife-75636-fig1-figsupp1-data1.zip › Fig1 source data 4 for Fig1 supplement 1/GFP+ALDOC OR GFP+NeuN/AAV-scramble SN #9 GFP+AldoC.jpg]

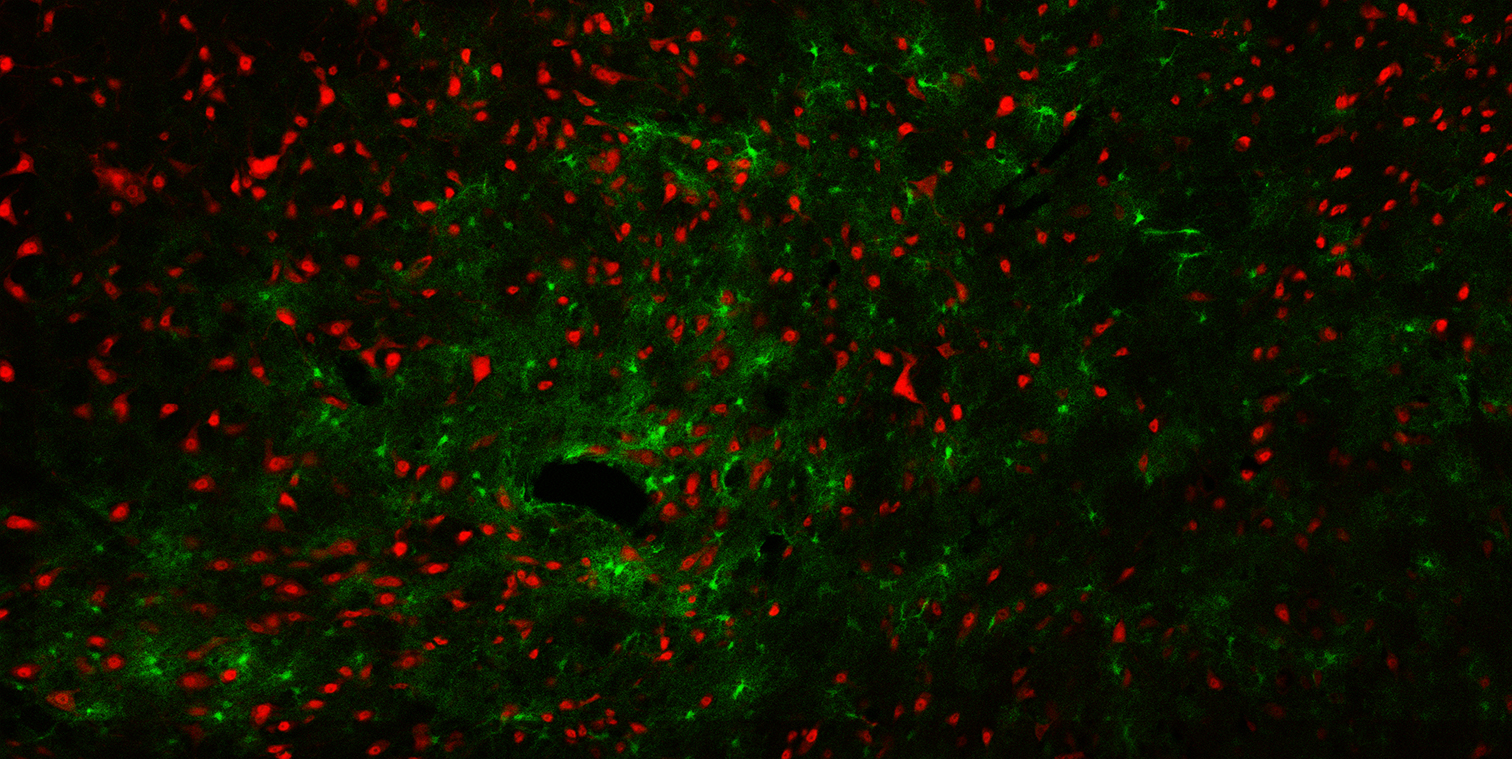

Supplement: Figure 1—figure supplement 1—source data 1. [file elife-75636-fig1-figsupp1-data1.zip › Fig1 source data 4 for Fig1 supplement 1/GFP+ALDOC OR GFP+NeuN/AAV-scramble SN #9 GFP+NeuN.jpg]

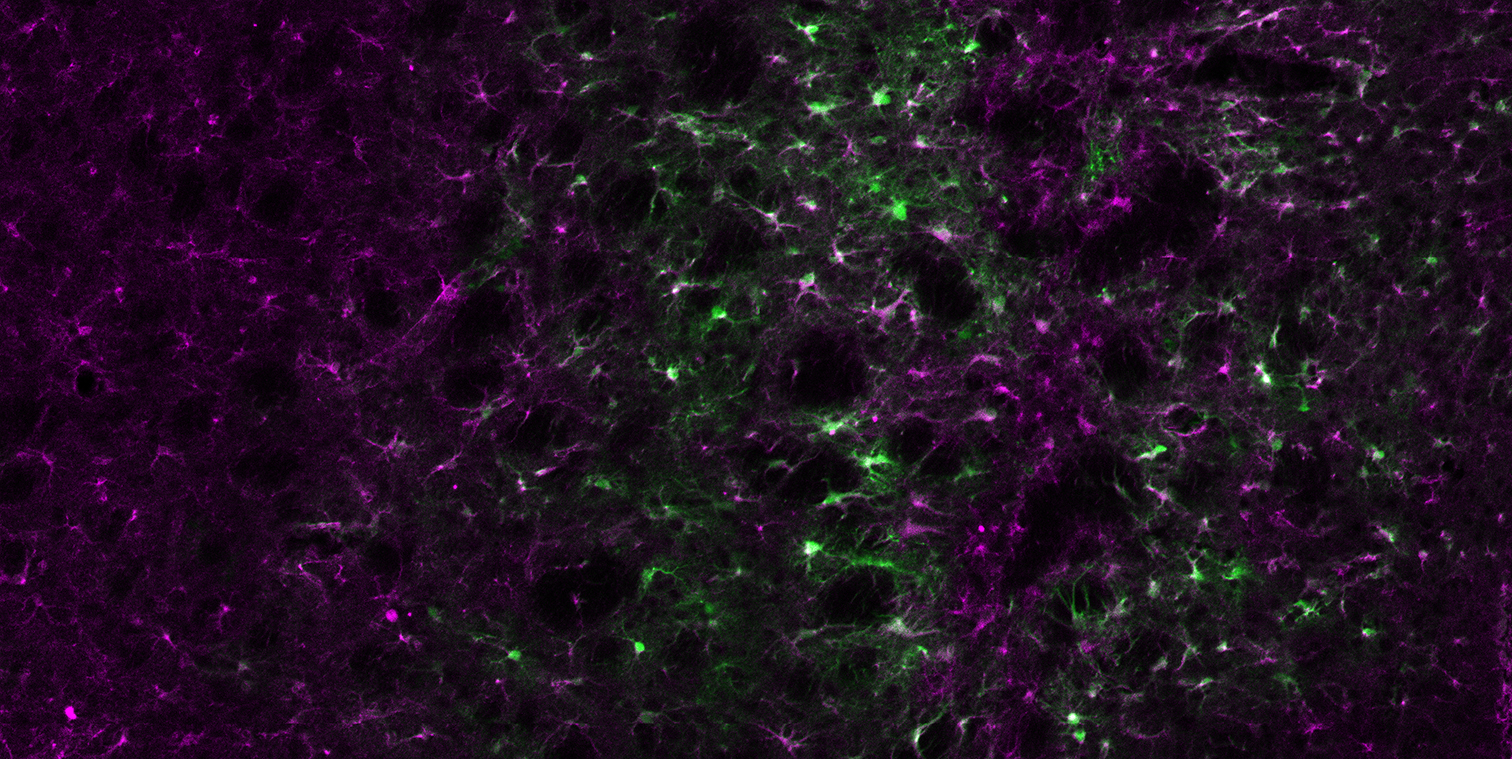

Supplement: Figure 1—figure supplement 1—source data 1. [file elife-75636-fig1-figsupp1-data1.zip › Fig1 source data 4 for Fig1 supplement 1/GFP+ALDOC OR GFP+NeuN/AAV-scramble STR #10 GFP+AldoC.jpg]

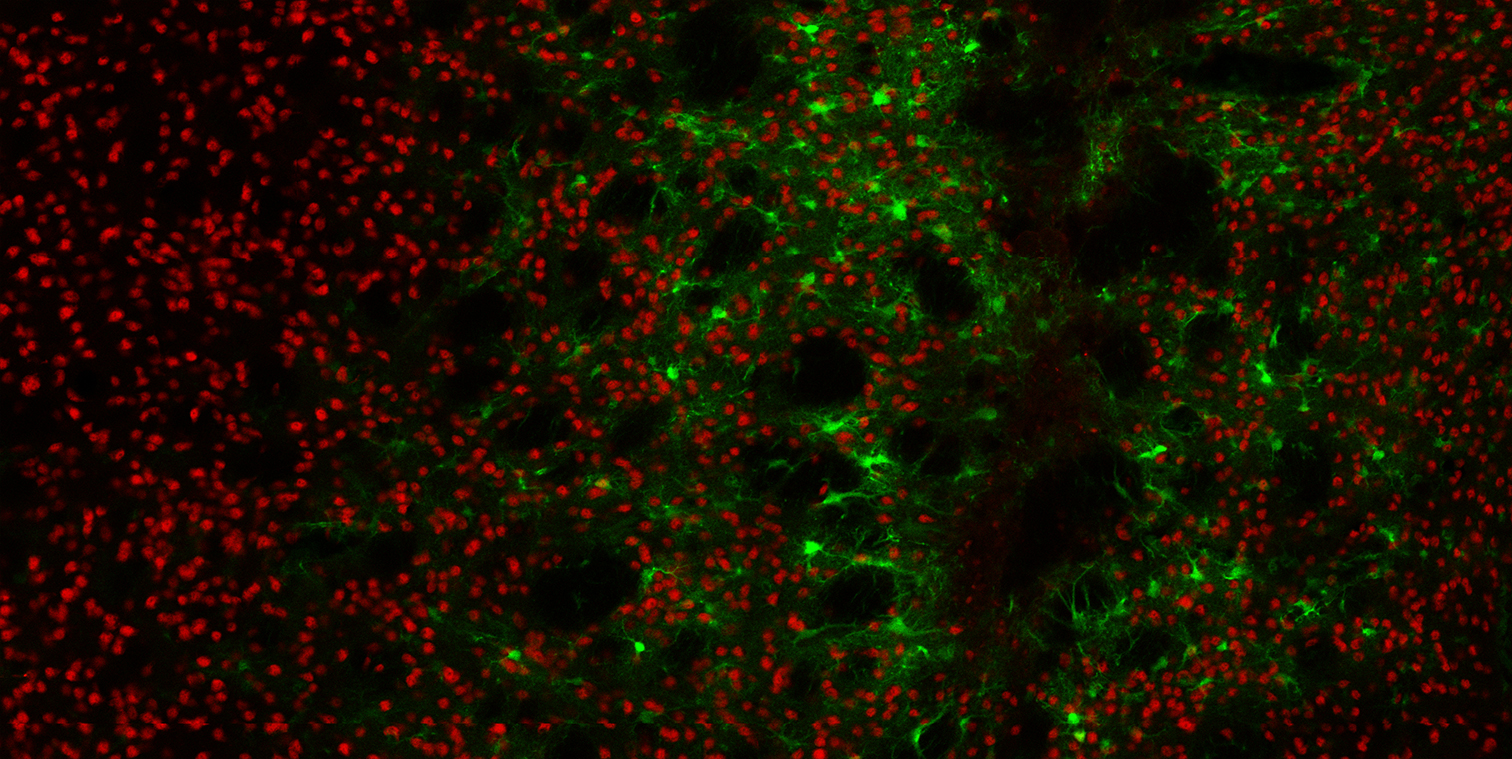

Supplement: Figure 1—figure supplement 1—source data 1. [file elife-75636-fig1-figsupp1-data1.zip › Fig1 source data 4 for Fig1 supplement 1/GFP+ALDOC OR GFP+NeuN/AAV-scramble STR #10 GFP+NeuN.jpg]

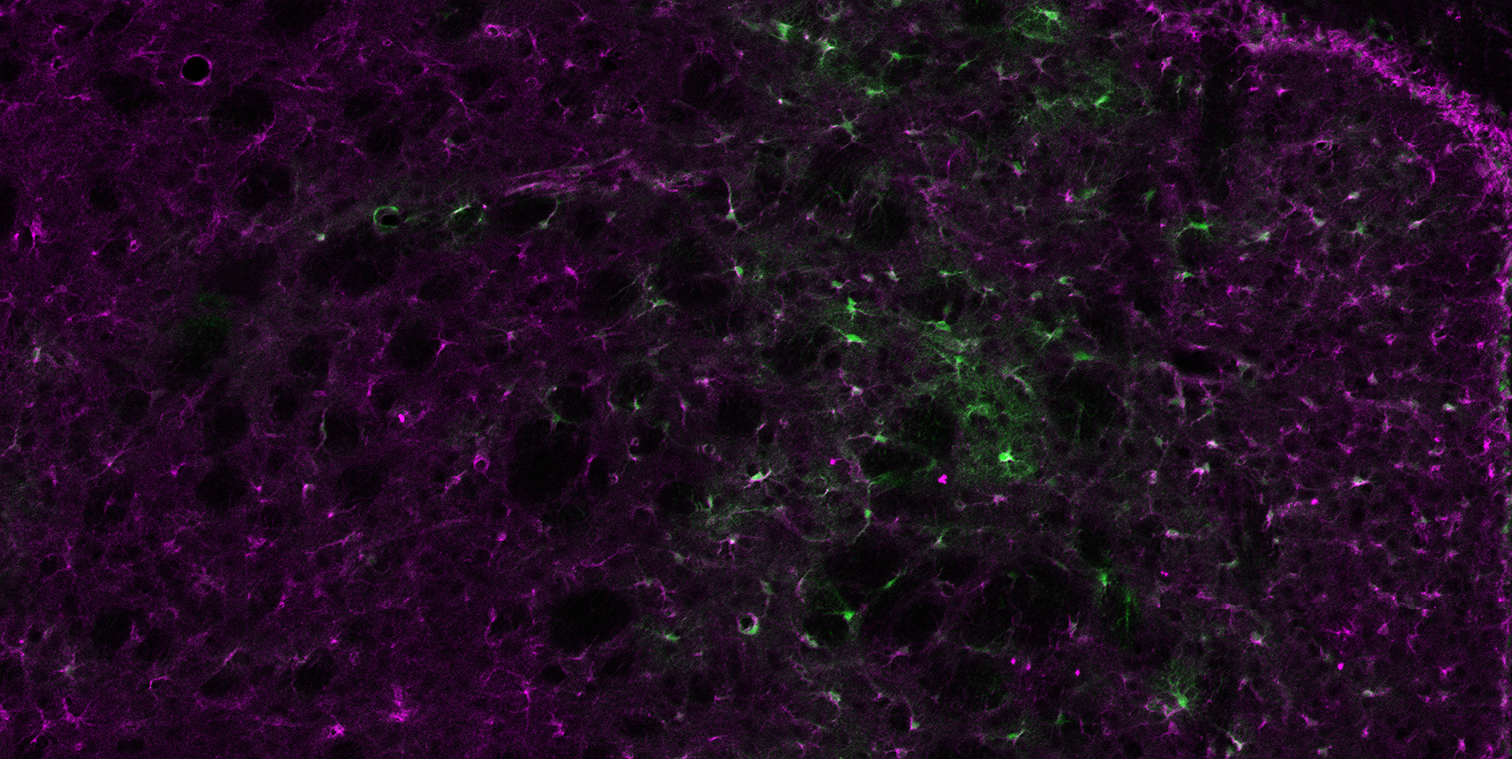

Supplement: Figure 1—figure supplement 1—source data 1. [file elife-75636-fig1-figsupp1-data1.zip › Fig1 source data 4 for Fig1 supplement 1/GFP+ALDOC OR GFP+NeuN/AAV-scramble STR #13 GFP+AldoC.jpg]

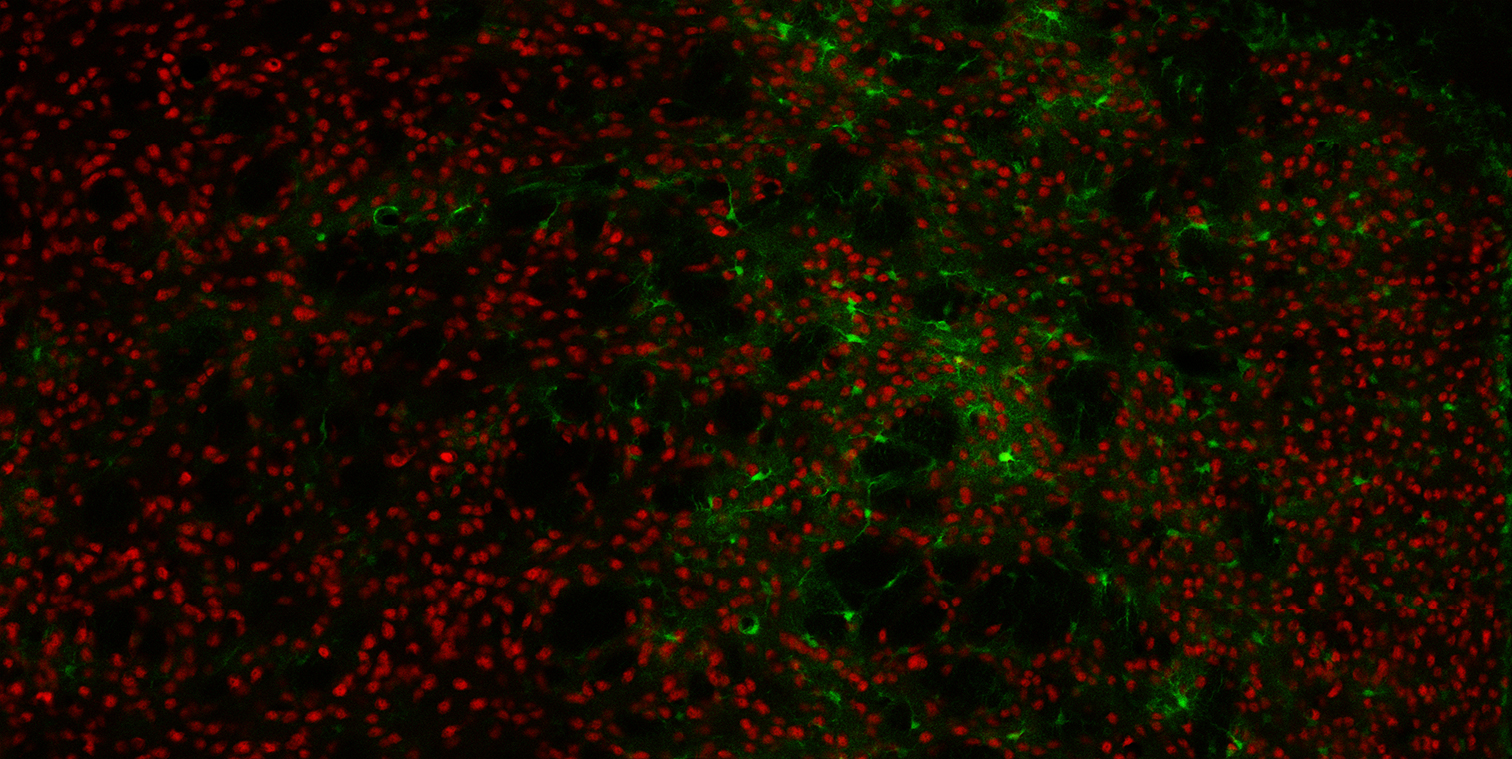

Supplement: Figure 1—figure supplement 1—source data 1. [file elife-75636-fig1-figsupp1-data1.zip › Fig1 source data 4 for Fig1 supplement 1/GFP+ALDOC OR GFP+NeuN/AAV-scramble STR #13 GFP+NeuN.jpg]

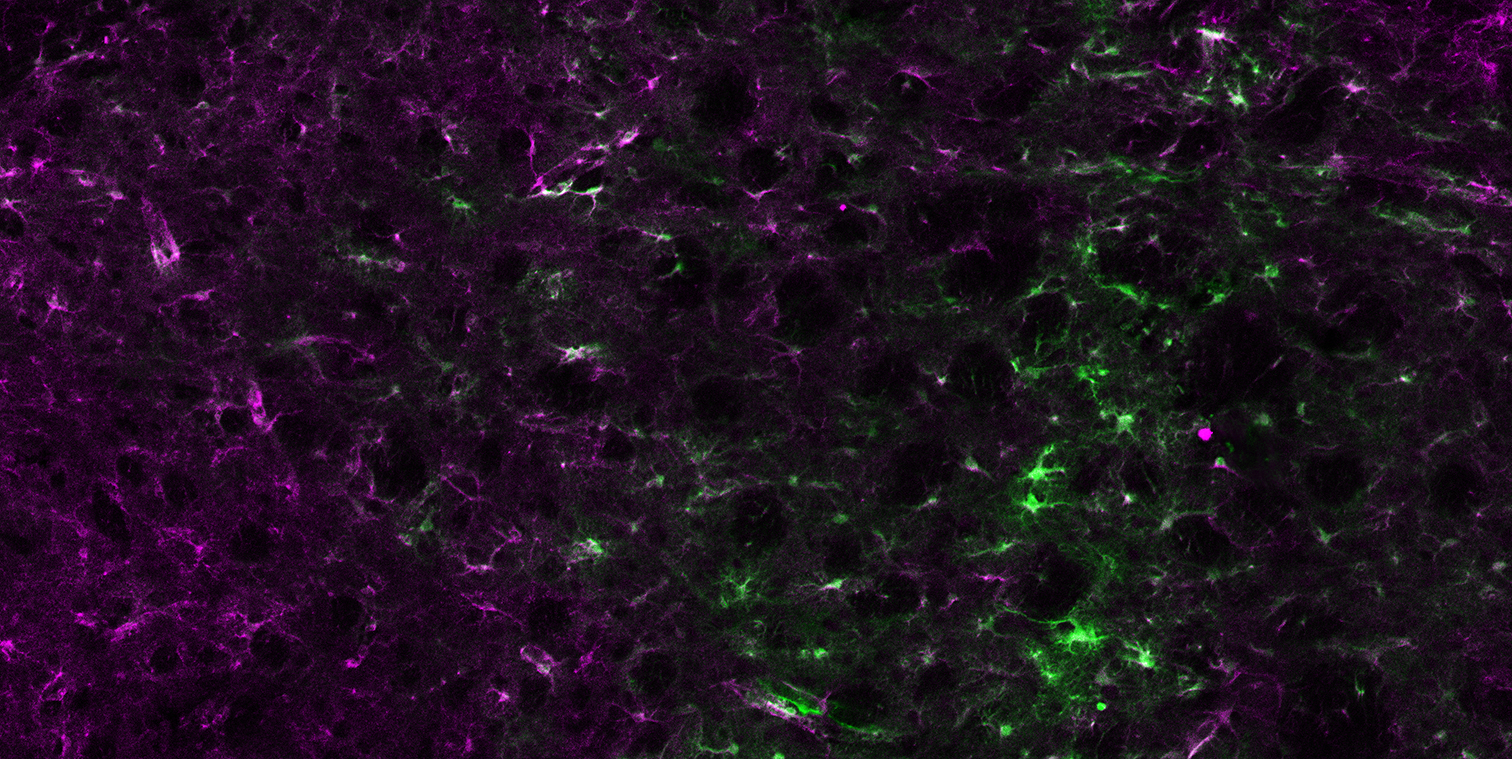

Supplement: Figure 1—figure supplement 1—source data 1. [file elife-75636-fig1-figsupp1-data1.zip › Fig1 source data 4 for Fig1 supplement 1/GFP+ALDOC OR GFP+NeuN/AAV-scramble STR #9 GFP+AldoC.jpg]

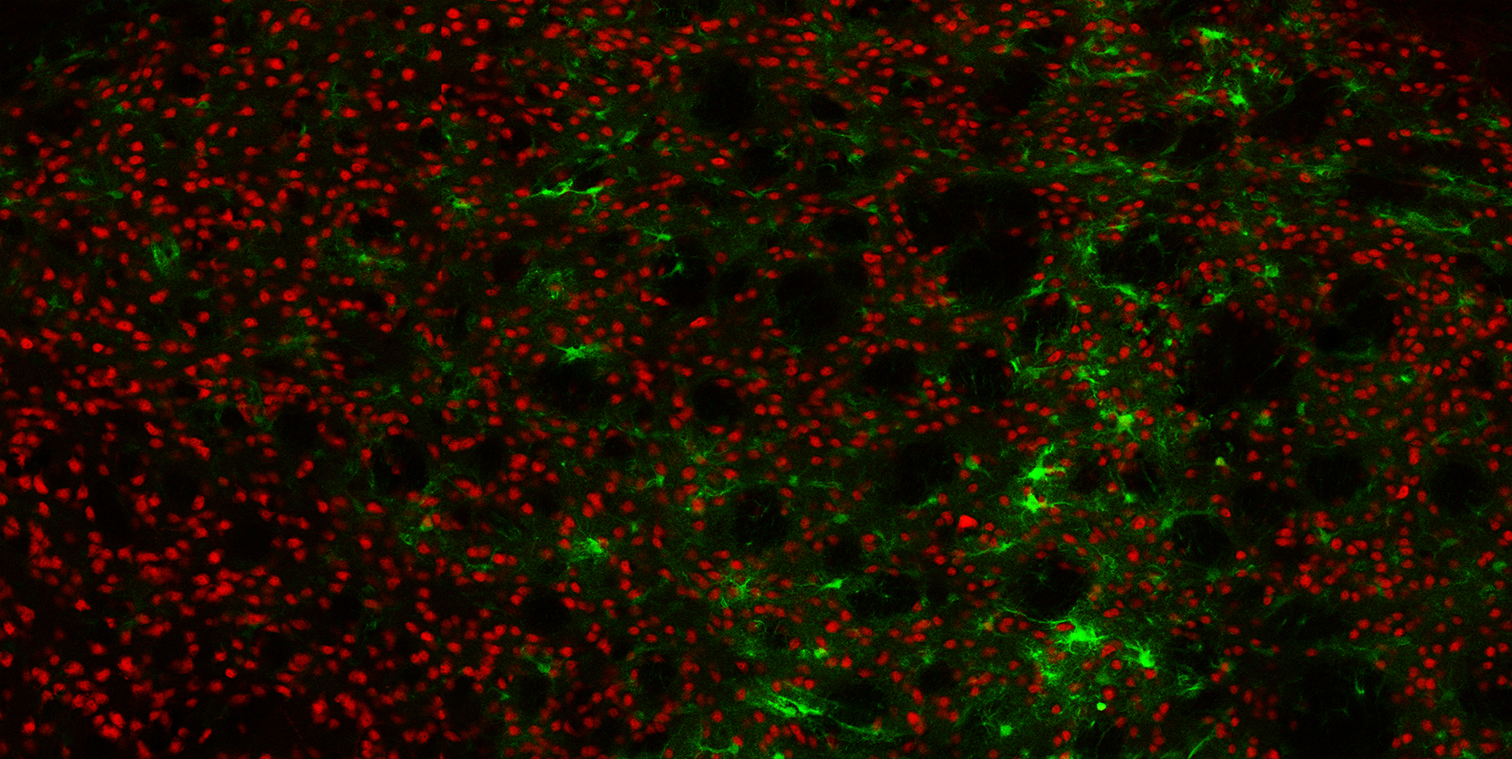

Supplement: Figure 1—figure supplement 1—source data 1. [file elife-75636-fig1-figsupp1-data1.zip › Fig1 source data 4 for Fig1 supplement 1/GFP+ALDOC OR GFP+NeuN/AAV-scramble STR #9 GFP+NeuN.jpg]

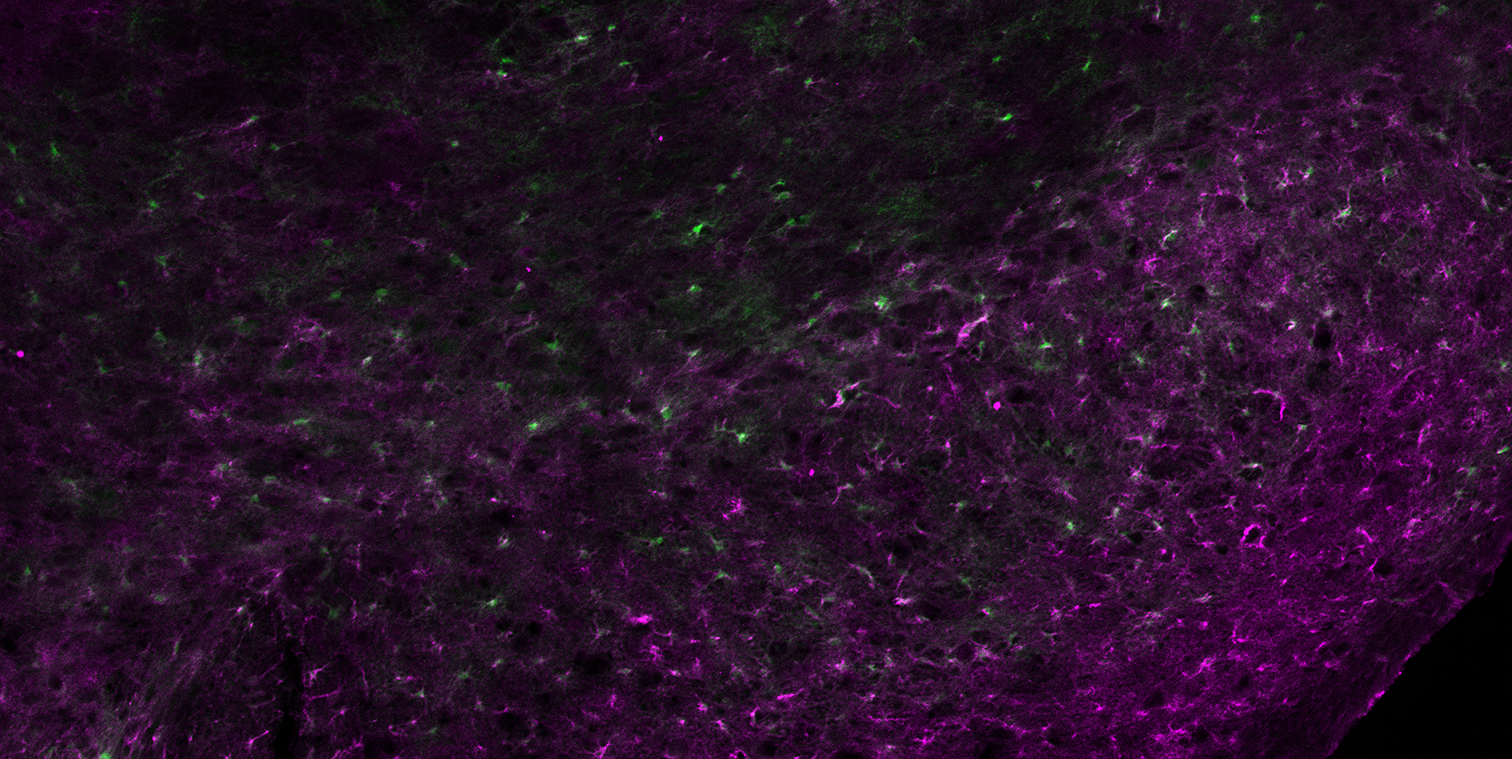

Supplement: Figure 1—figure supplement 1—source data 1. [file elife-75636-fig1-figsupp1-data1.zip › Fig1 source data 4 for Fig1 supplement 1/GFP+ALDOC OR GFP+NeuN/AAV-shPTB SN #12 GFP+AldoC.jpg]

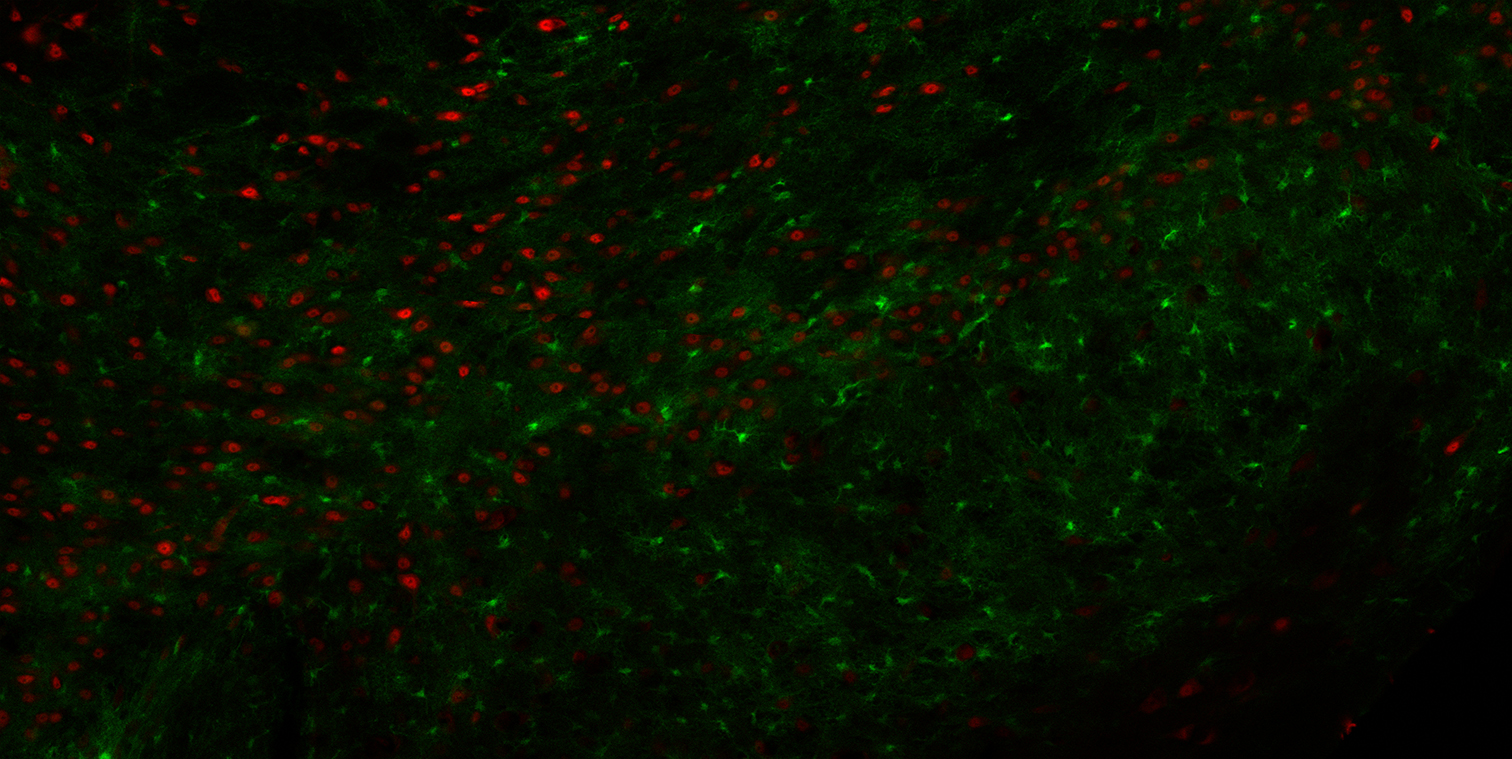

Supplement: Figure 1—figure supplement 1—source data 1. [file elife-75636-fig1-figsupp1-data1.zip › Fig1 source data 4 for Fig1 supplement 1/GFP+ALDOC OR GFP+NeuN/AAV-shPTB SN #12 GFP+NeuN.jpg]

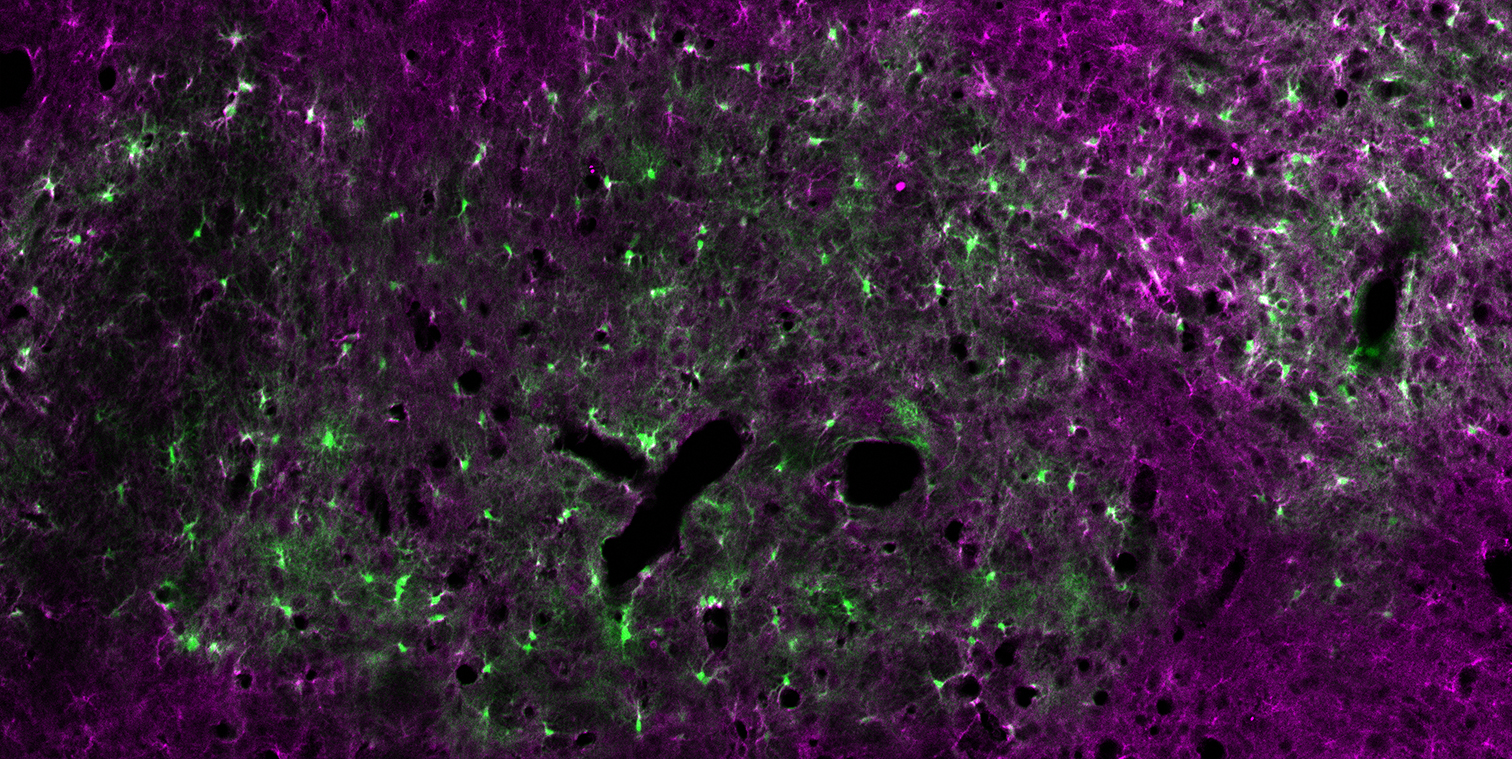

Supplement: Figure 1—figure supplement 1—source data 1. [file elife-75636-fig1-figsupp1-data1.zip › Fig1 source data 4 for Fig1 supplement 1/GFP+ALDOC OR GFP+NeuN/AAV-shPTB SN #53 GFP+AldoC.jpg]

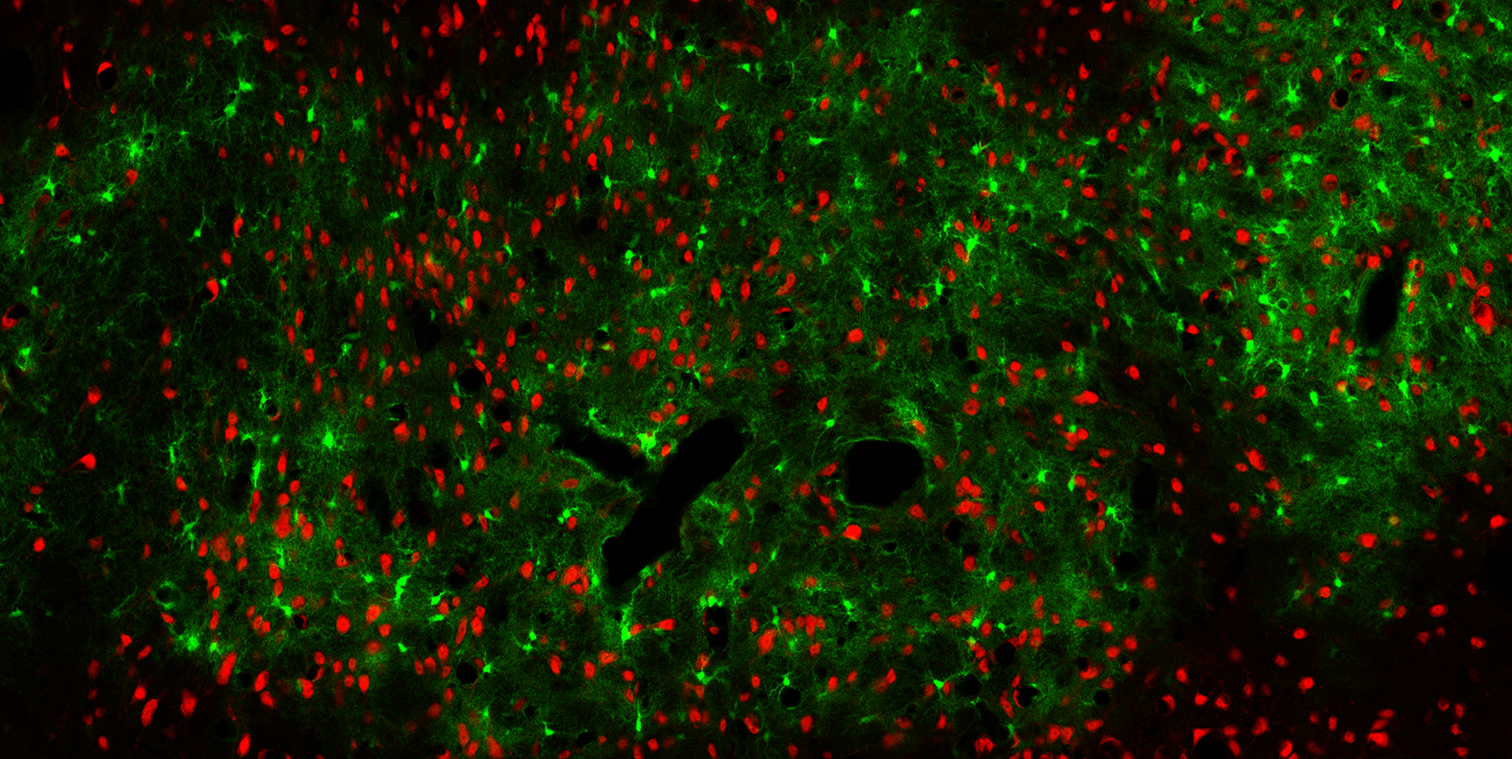

Supplement: Figure 1—figure supplement 1—source data 1. [file elife-75636-fig1-figsupp1-data1.zip › Fig1 source data 4 for Fig1 supplement 1/GFP+ALDOC OR GFP+NeuN/AAV-shPTB SN #53 GFP+NeuN.jpg]

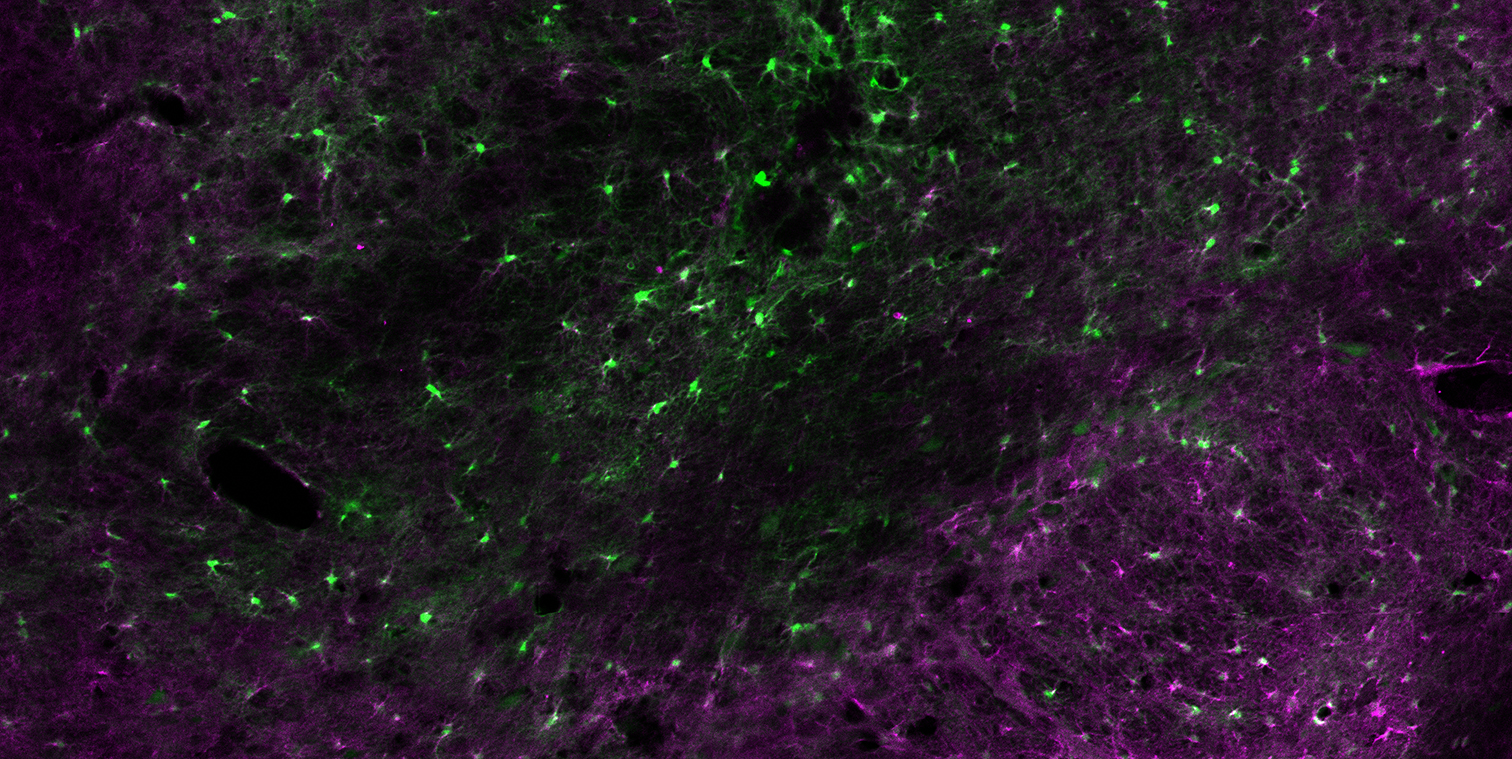

Supplement: Figure 1—figure supplement 1—source data 1. [file elife-75636-fig1-figsupp1-data1.zip › Fig1 source data 4 for Fig1 supplement 1/GFP+ALDOC OR GFP+NeuN/AAV-shPTB SN #98 GFP+AldoC.jpg]

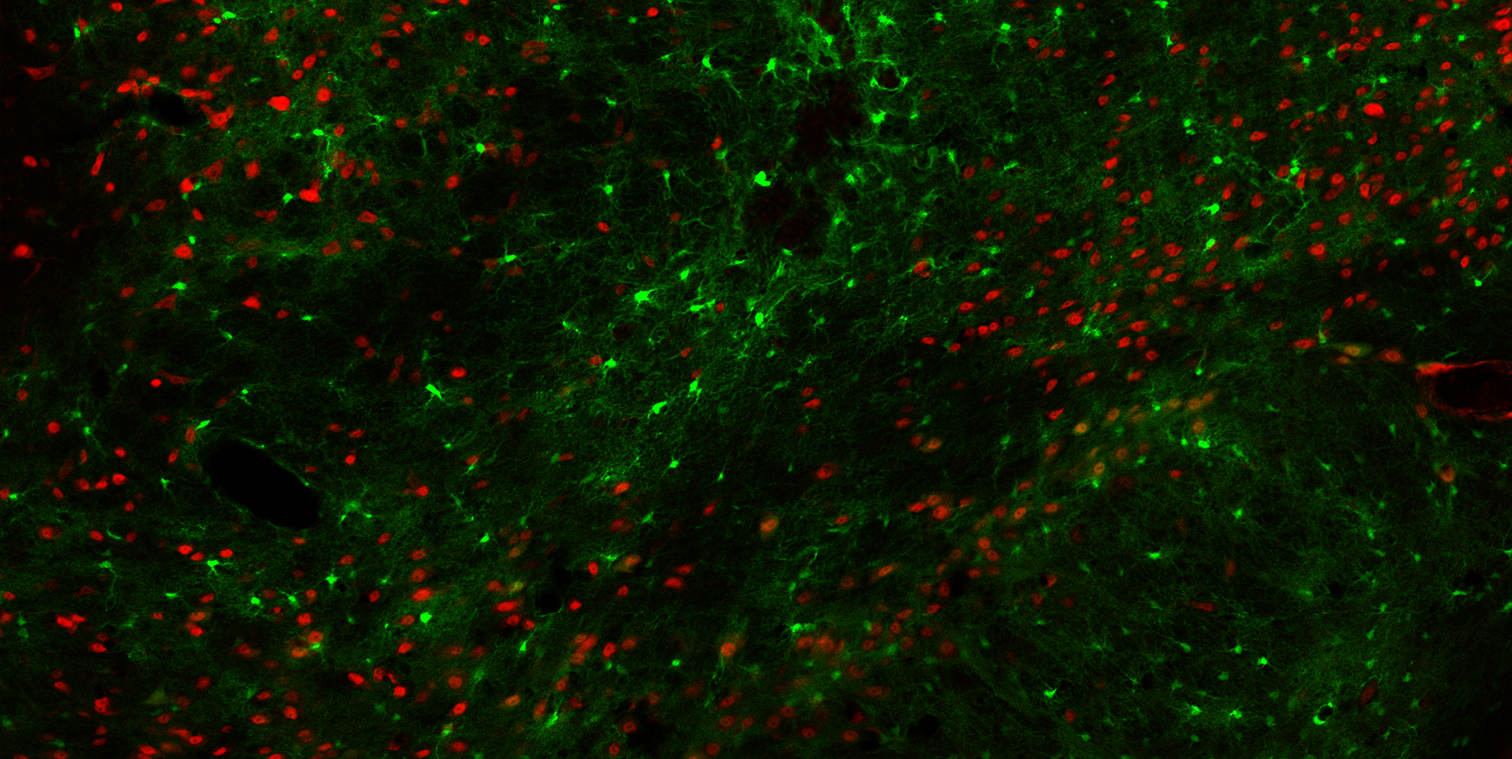

Supplement: Figure 1—figure supplement 1—source data 1. [file elife-75636-fig1-figsupp1-data1.zip › Fig1 source data 4 for Fig1 supplement 1/GFP+ALDOC OR GFP+NeuN/AAV-shPTB SN #98 GFP+NeuN.jpg]

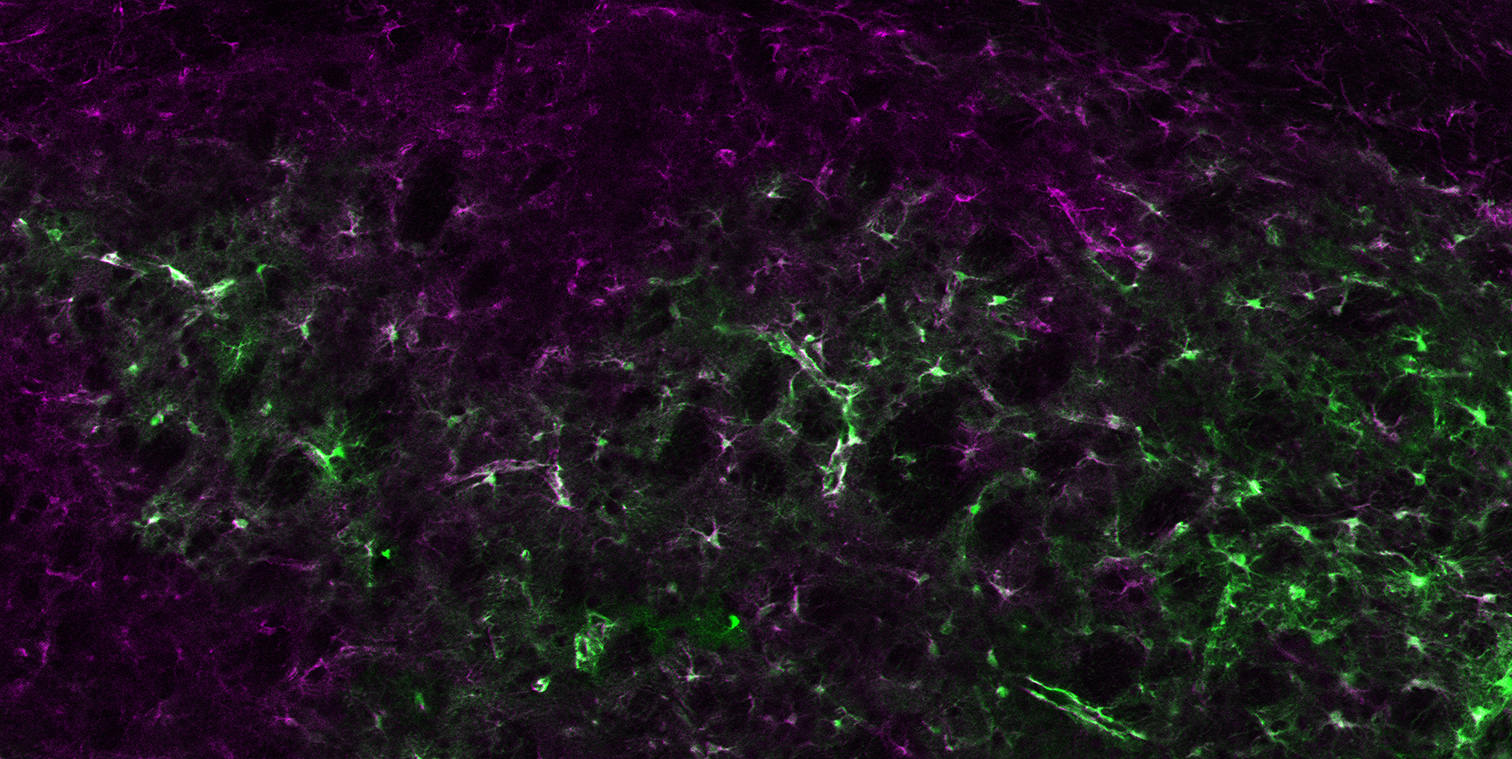

Supplement: Figure 1—figure supplement 1—source data 1. [file elife-75636-fig1-figsupp1-data1.zip › Fig1 source data 4 for Fig1 supplement 1/GFP+ALDOC OR GFP+NeuN/AAV-shPTB STR #12 GFP+AldoC.jpg]

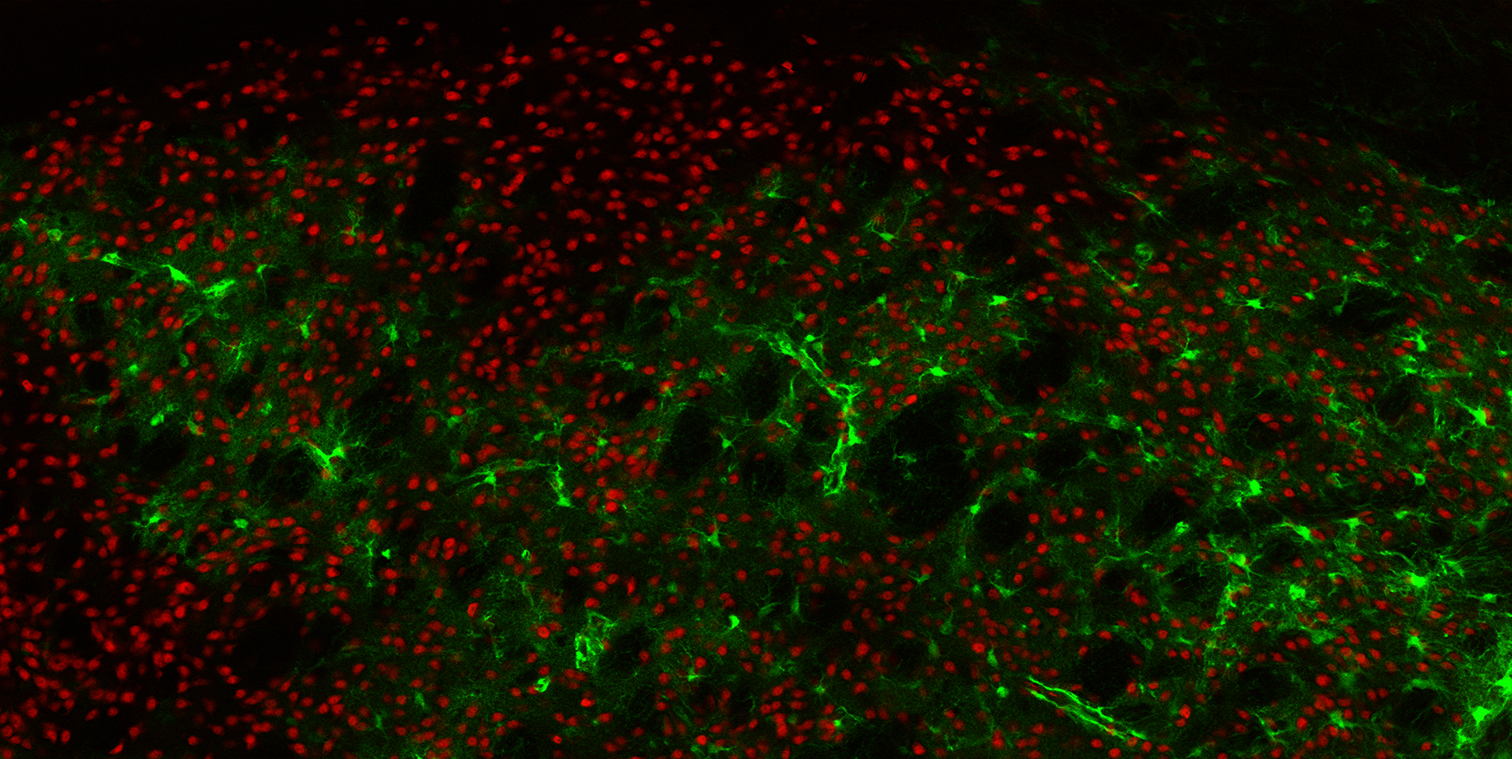

Supplement: Figure 1—figure supplement 1—source data 1. [file elife-75636-fig1-figsupp1-data1.zip › Fig1 source data 4 for Fig1 supplement 1/GFP+ALDOC OR GFP+NeuN/AAV-shPTB STR #12 GFP+NeuN.jpg]

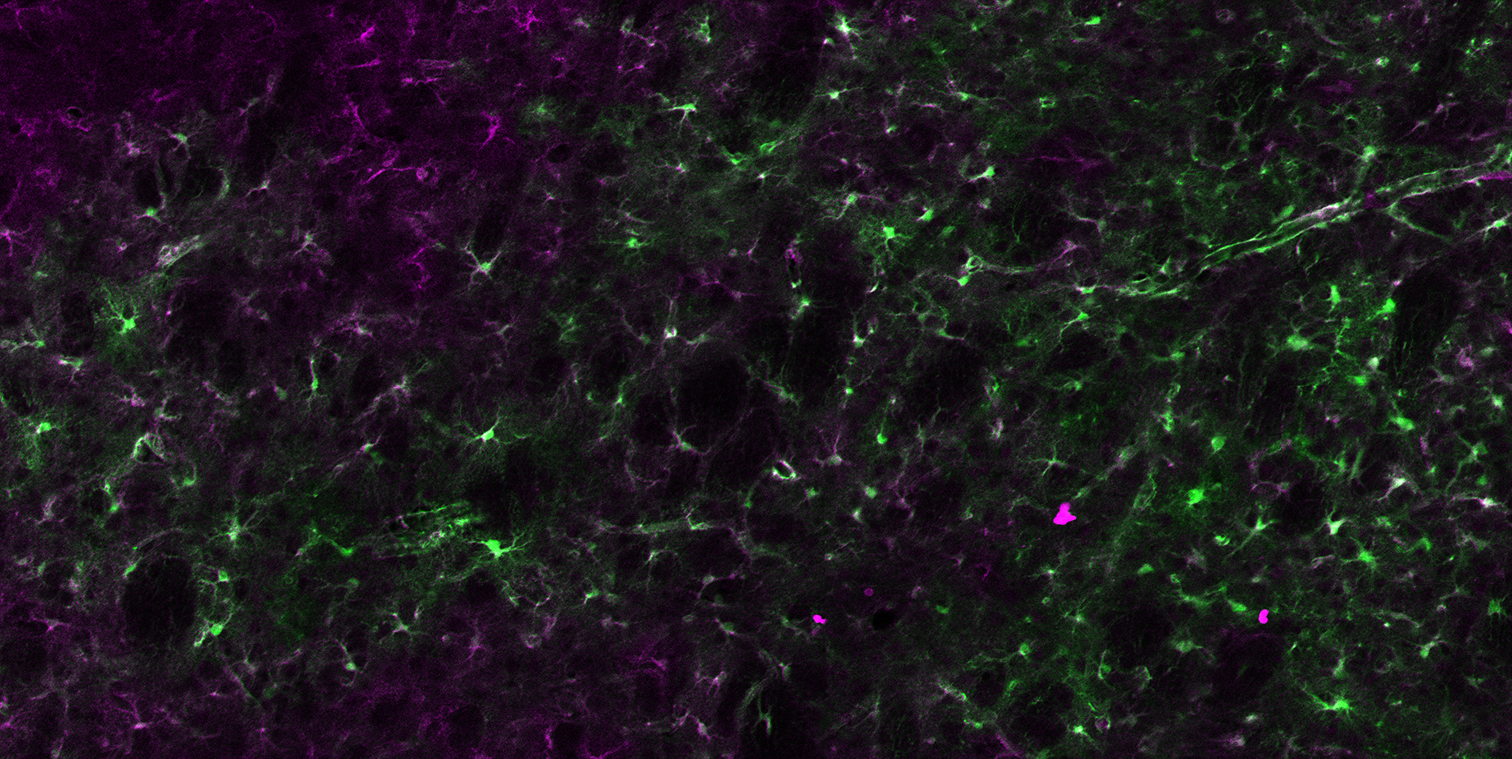

Supplement: Figure 1—figure supplement 1—source data 1. [file elife-75636-fig1-figsupp1-data1.zip › Fig1 source data 4 for Fig1 supplement 1/GFP+ALDOC OR GFP+NeuN/AAV-shPTB STR #53 GFP+AldoC.jpg]

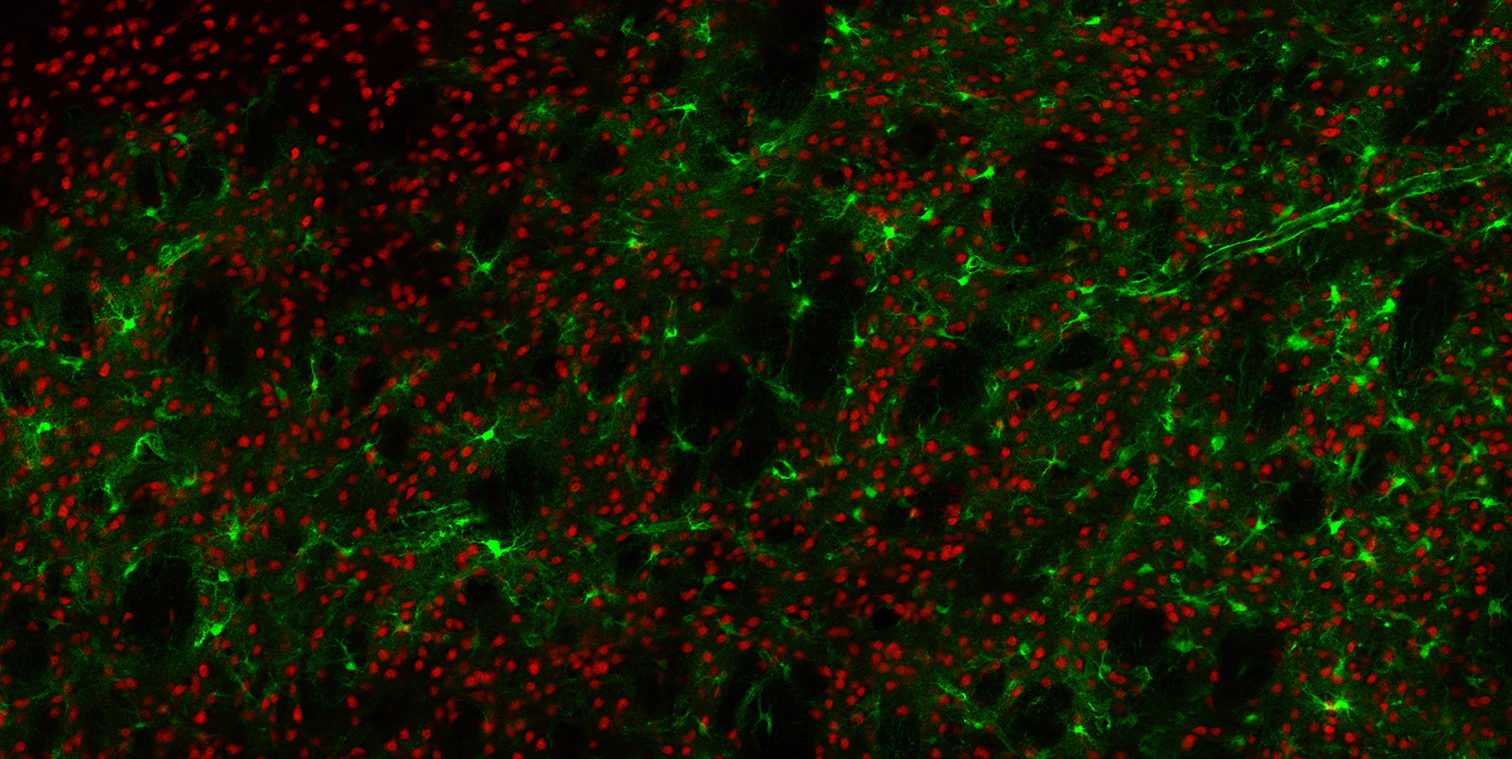

Supplement: Figure 1—figure supplement 1—source data 1. [file elife-75636-fig1-figsupp1-data1.zip › Fig1 source data 4 for Fig1 supplement 1/GFP+ALDOC OR GFP+NeuN/AAV-shPTB STR #53 GFP+NeuN.jpg]

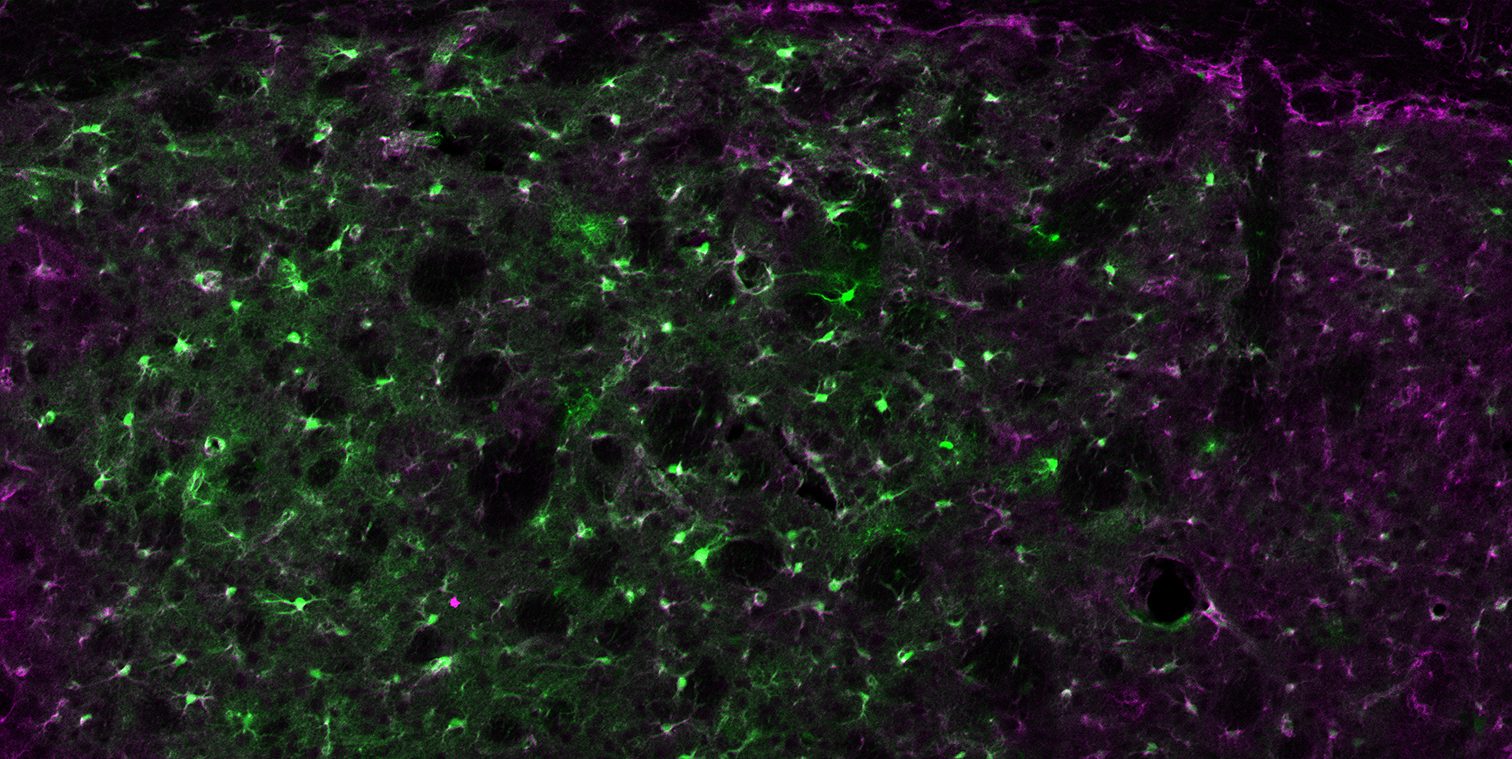

Supplement: Figure 1—figure supplement 1—source data 1. [file elife-75636-fig1-figsupp1-data1.zip › Fig1 source data 4 for Fig1 supplement 1/GFP+ALDOC OR GFP+NeuN/AAV-shPTB STR #98 GFP+AldoC.jpg]

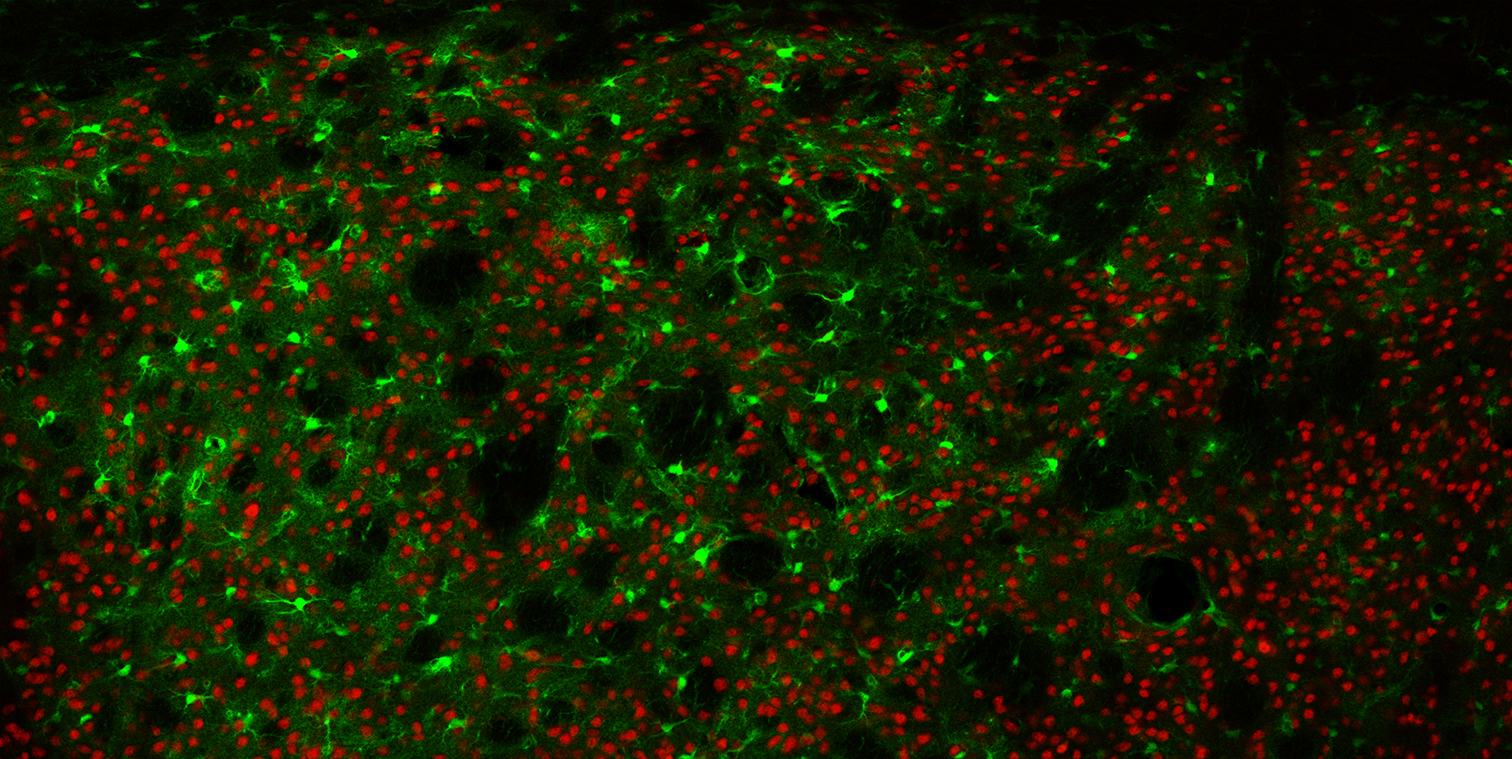

Supplement: Figure 1—figure supplement 1—source data 1. [file elife-75636-fig1-figsupp1-data1.zip › Fig1 source data 4 for Fig1 supplement 1/GFP+ALDOC OR GFP+NeuN/AAV-shPTB STR #98 GFP+NeuN.jpg]

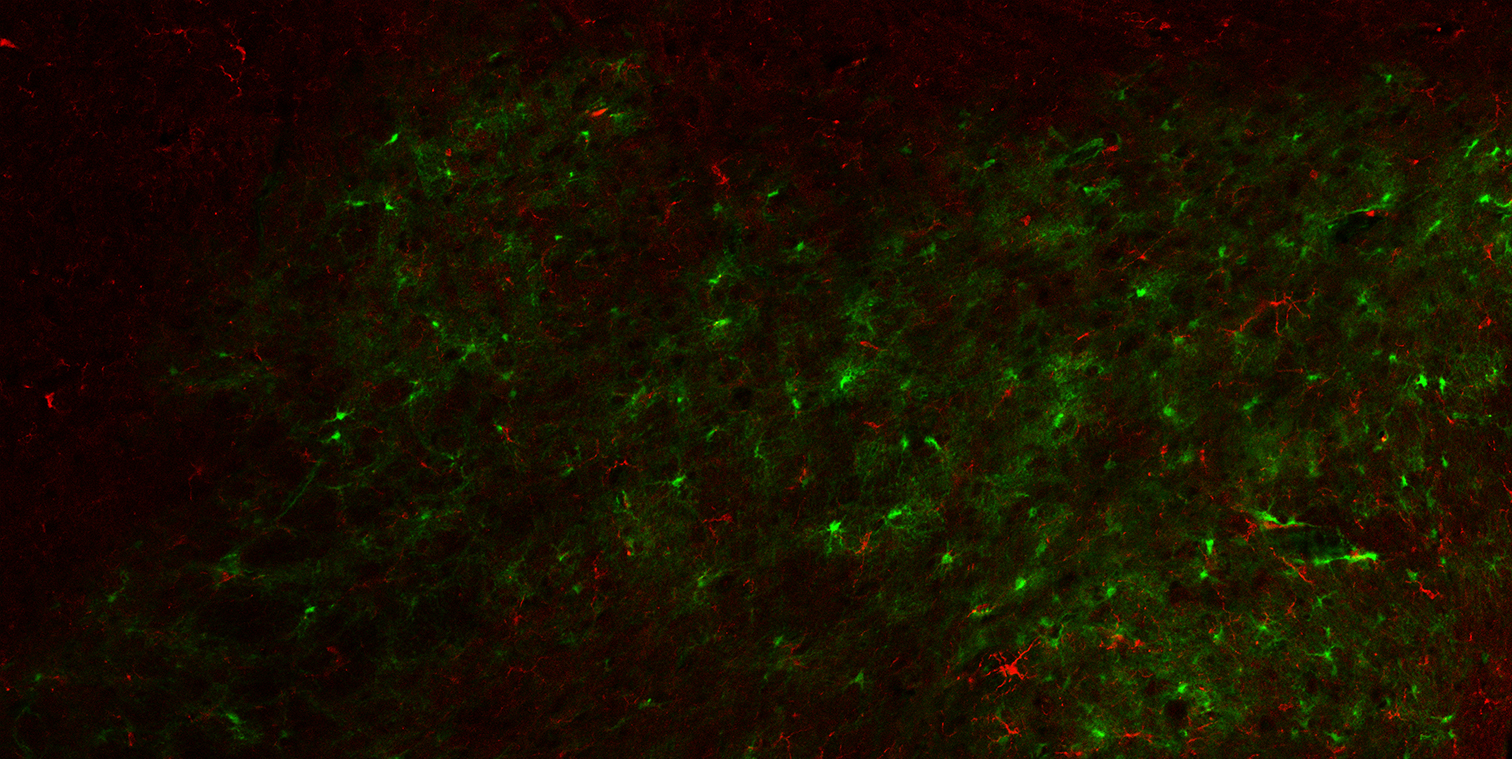

Supplement: Figure 1—figure supplement 1—source data 1. [file elife-75636-fig1-figsupp1-data1.zip › Fig1 source data 4 for Fig1 supplement 1/GFP+NG2 OR GFP+Iba-1/AAV-shPTB SN #12 GFP+Iba-1.jpg]

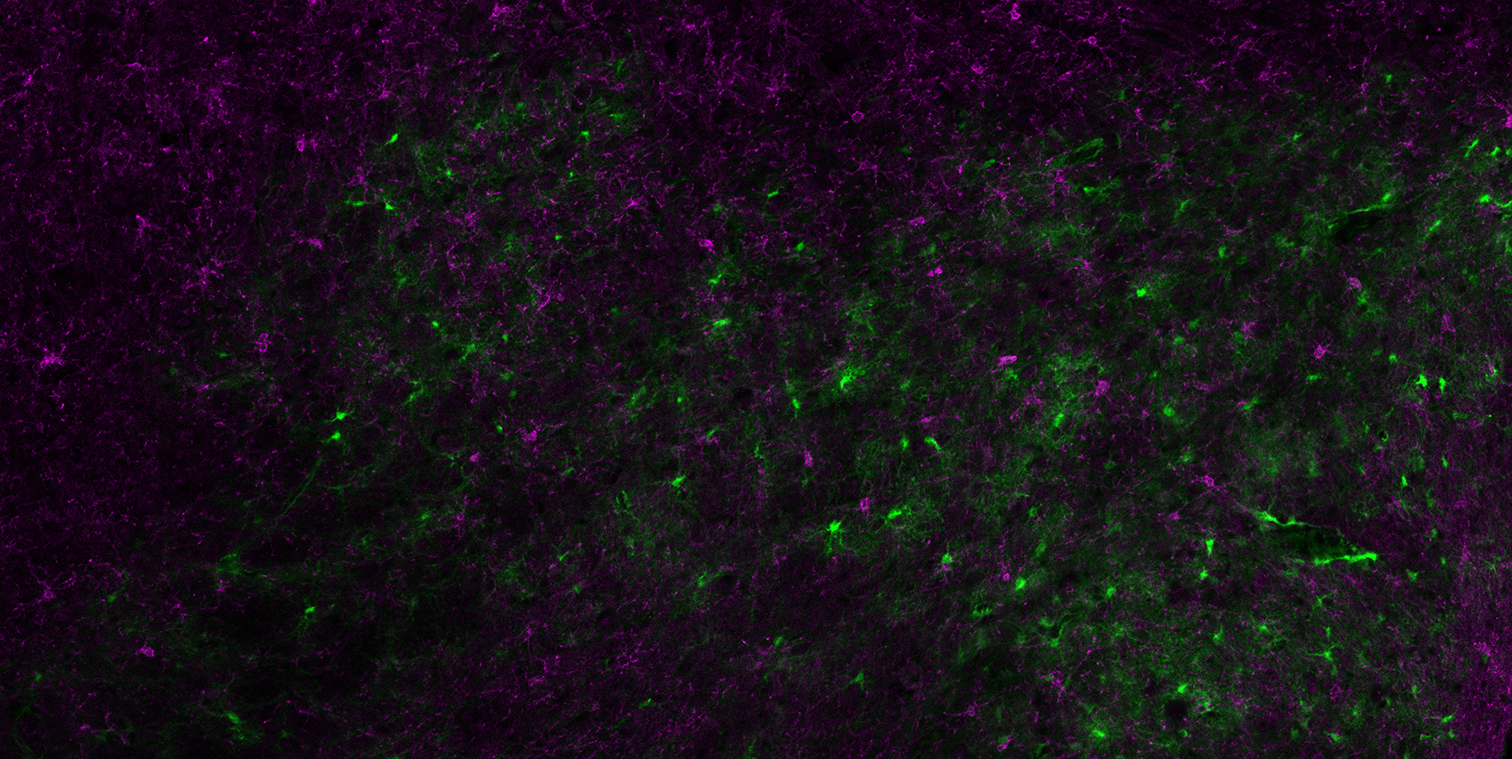

Supplement: Figure 1—figure supplement 1—source data 1. [file elife-75636-fig1-figsupp1-data1.zip › Fig1 source data 4 for Fig1 supplement 1/GFP+NG2 OR GFP+Iba-1/AAV-shPTB SN #12 GFP+NG2.jpg]

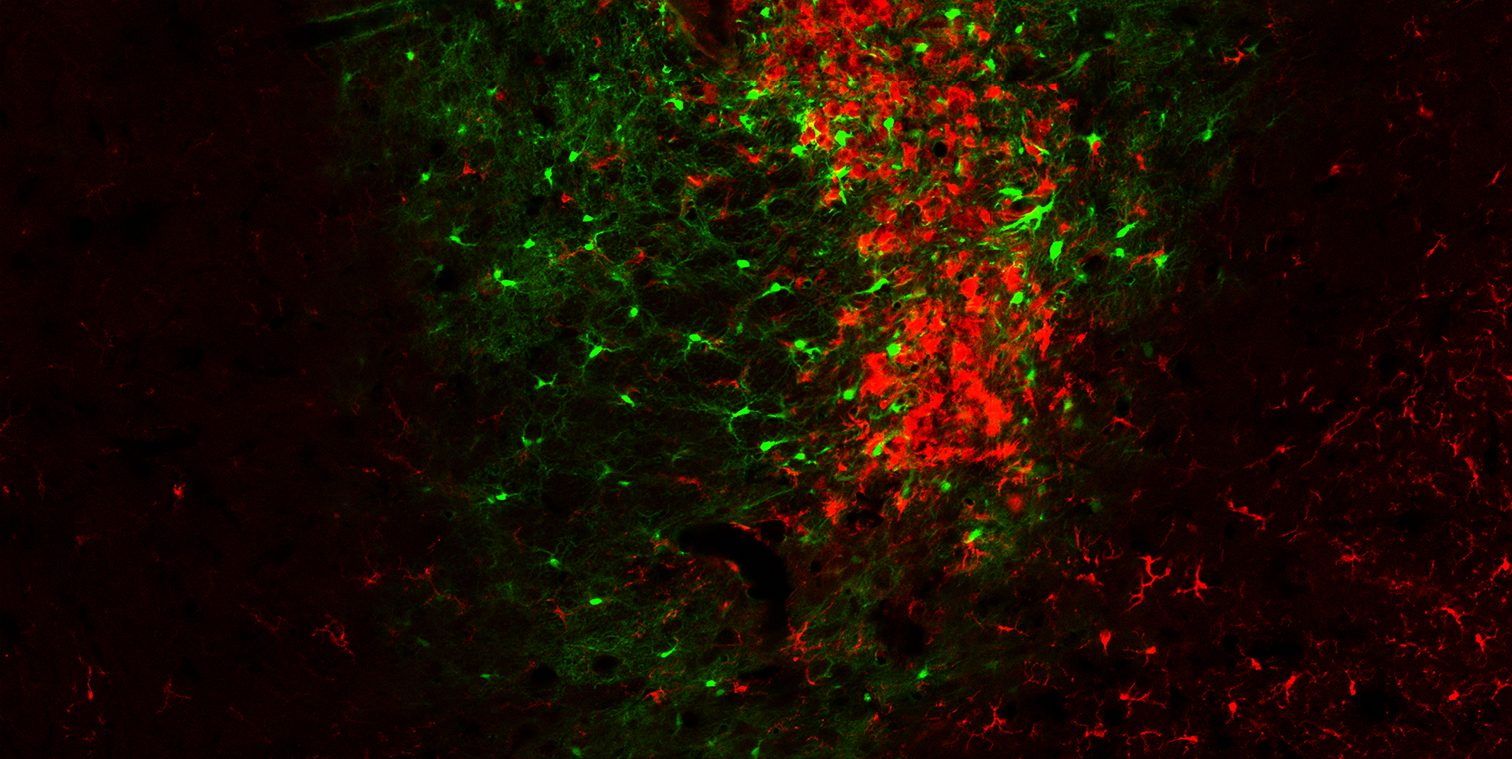

Supplement: Figure 1—figure supplement 1—source data 1. [file elife-75636-fig1-figsupp1-data1.zip › Fig1 source data 4 for Fig1 supplement 1/GFP+NG2 OR GFP+Iba-1/AAV-shPTB SN #53 GFP+Iba-1.jpg]

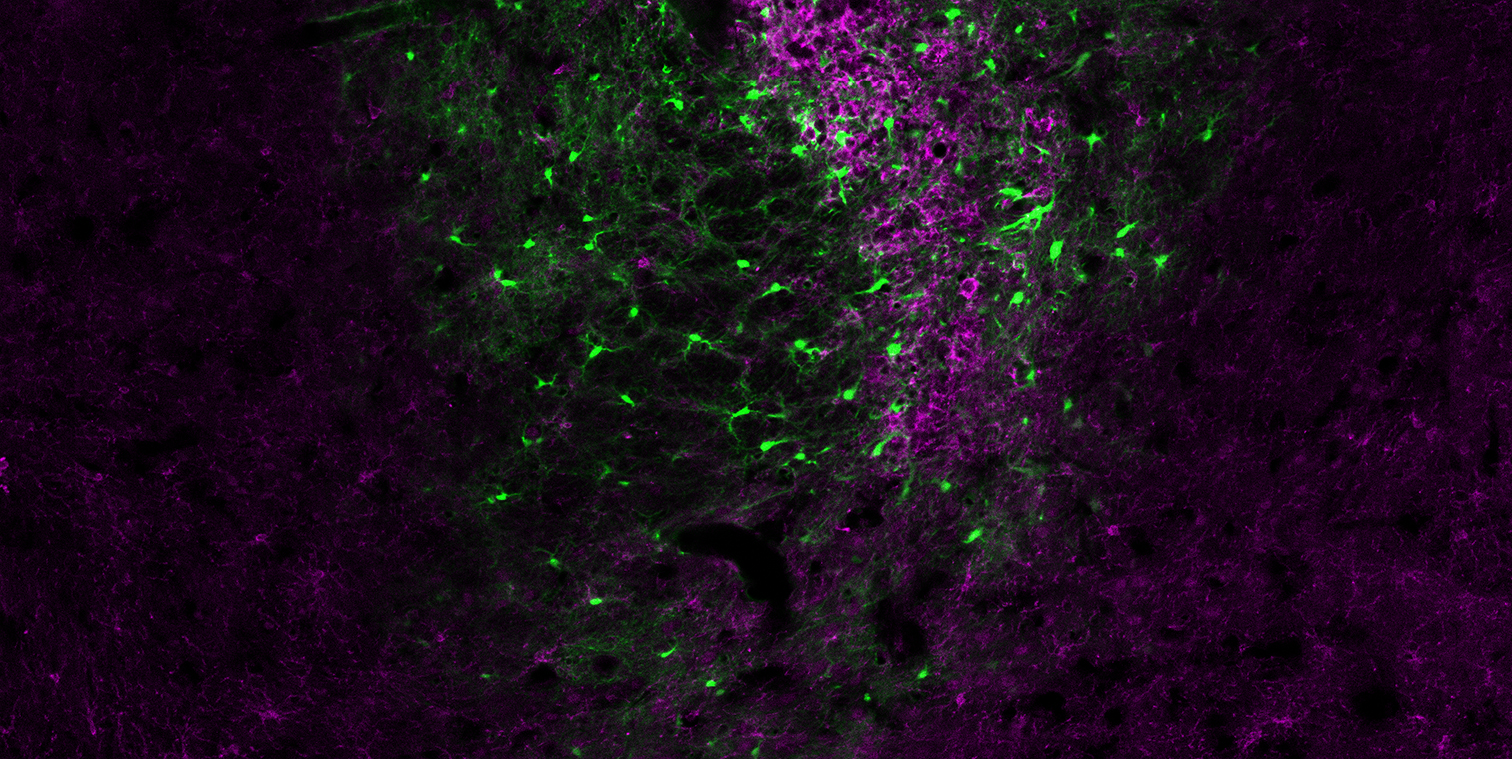

Supplement: Figure 1—figure supplement 1—source data 1. [file elife-75636-fig1-figsupp1-data1.zip › Fig1 source data 4 for Fig1 supplement 1/GFP+NG2 OR GFP+Iba-1/AAV-shPTB SN #53 GFP+NG2.jpg]

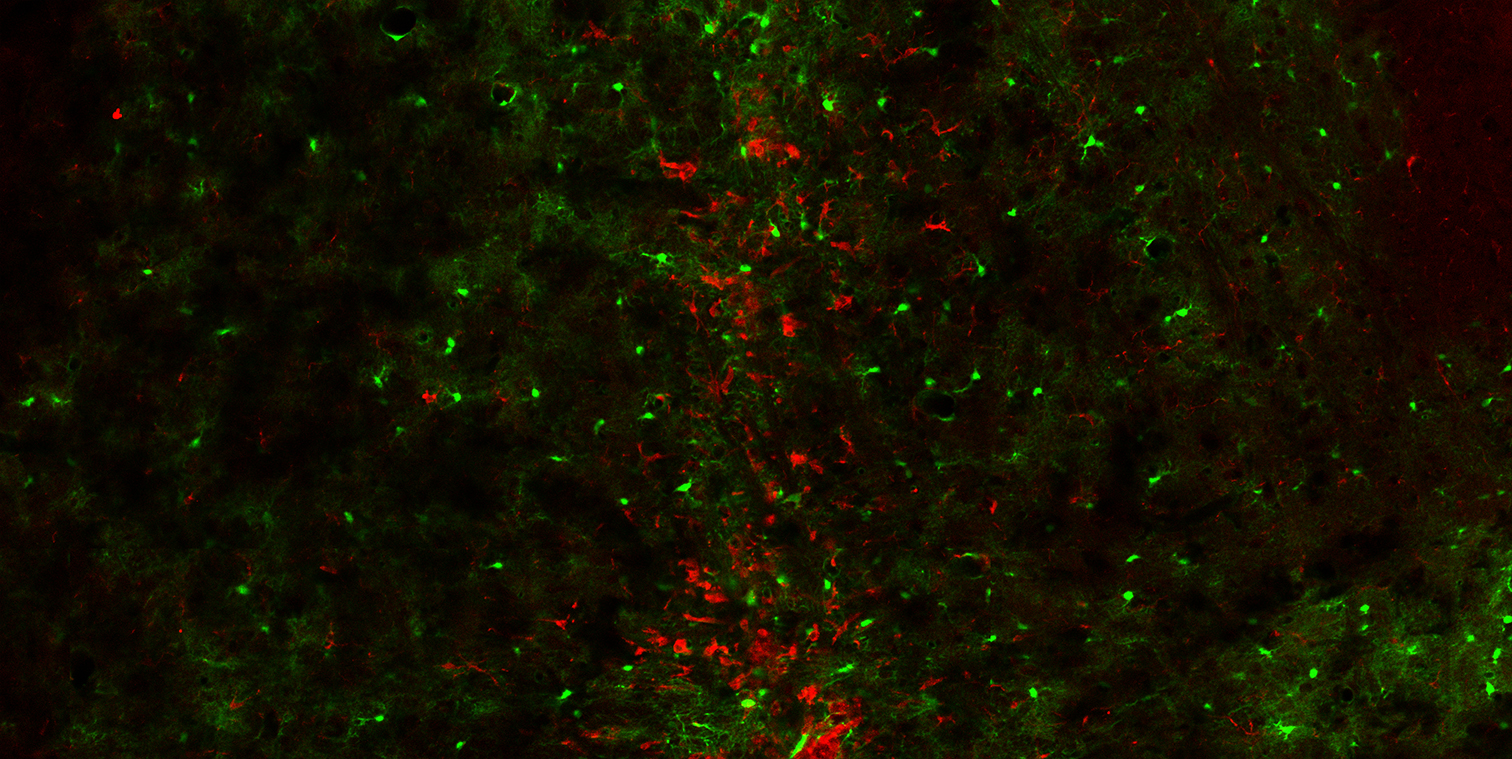

Supplement: Figure 1—figure supplement 1—source data 1. [file elife-75636-fig1-figsupp1-data1.zip › Fig1 source data 4 for Fig1 supplement 1/GFP+NG2 OR GFP+Iba-1/AAV-shPTB SN #98 GFP+Iba-1.jpg]

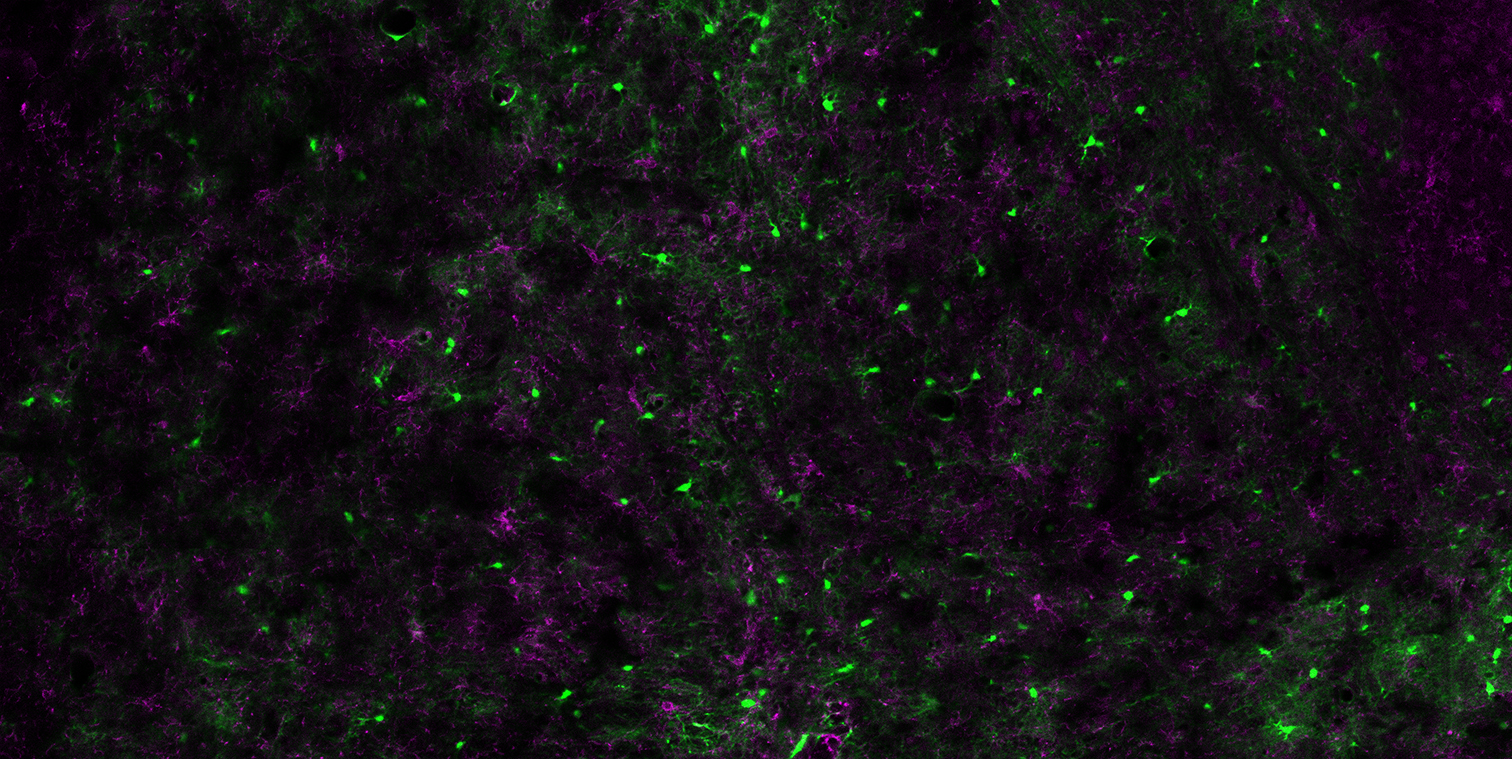

Supplement: Figure 1—figure supplement 1—source data 1. [file elife-75636-fig1-figsupp1-data1.zip › Fig1 source data 4 for Fig1 supplement 1/GFP+NG2 OR GFP+Iba-1/AAV-shPTB SN #98 GFP+NG2.jpg]

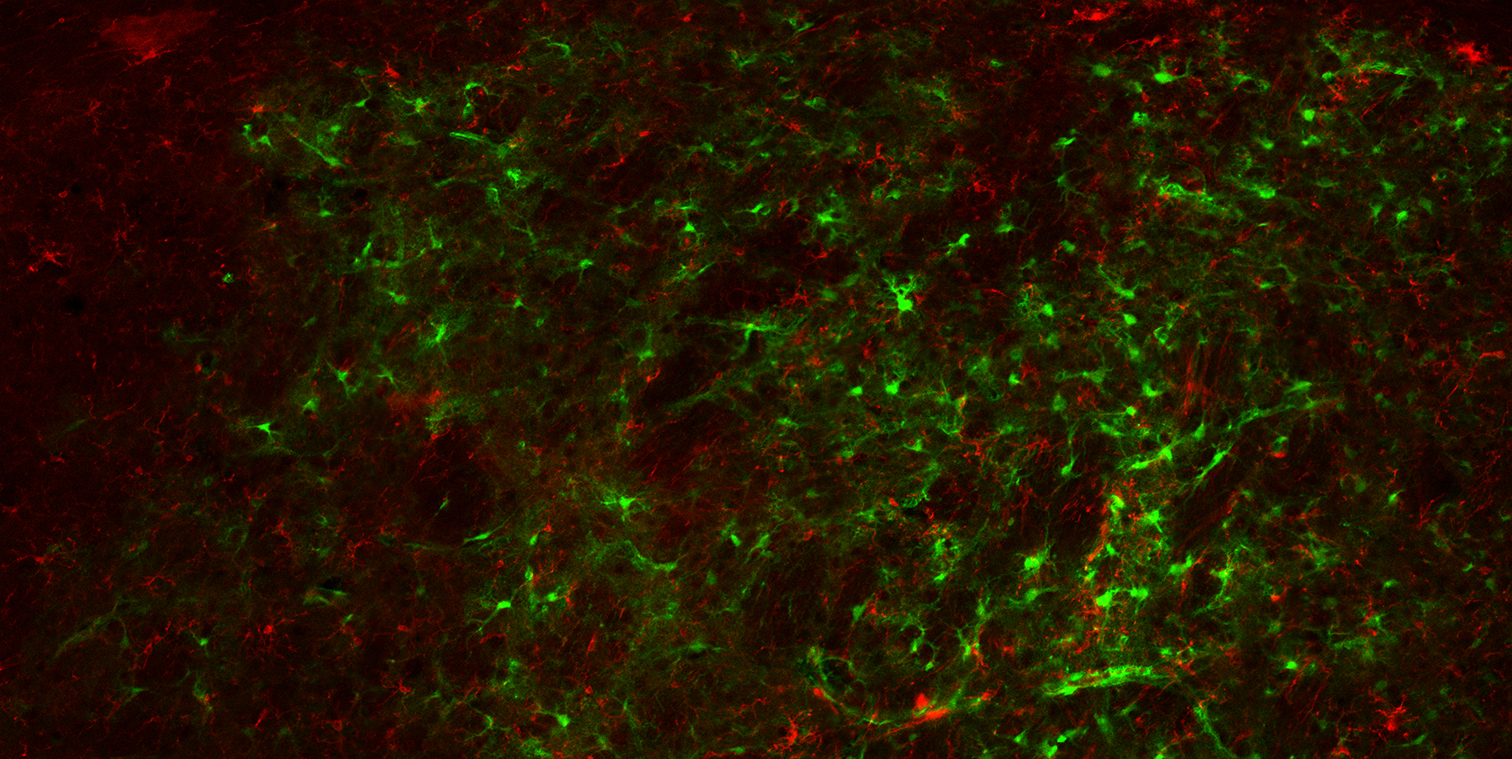

Supplement: Figure 1—figure supplement 1—source data 1. [file elife-75636-fig1-figsupp1-data1.zip › Fig1 source data 4 for Fig1 supplement 1/GFP+NG2 OR GFP+Iba-1/AAV-shPTB STR #12 GFP+Iba-1.jpg]

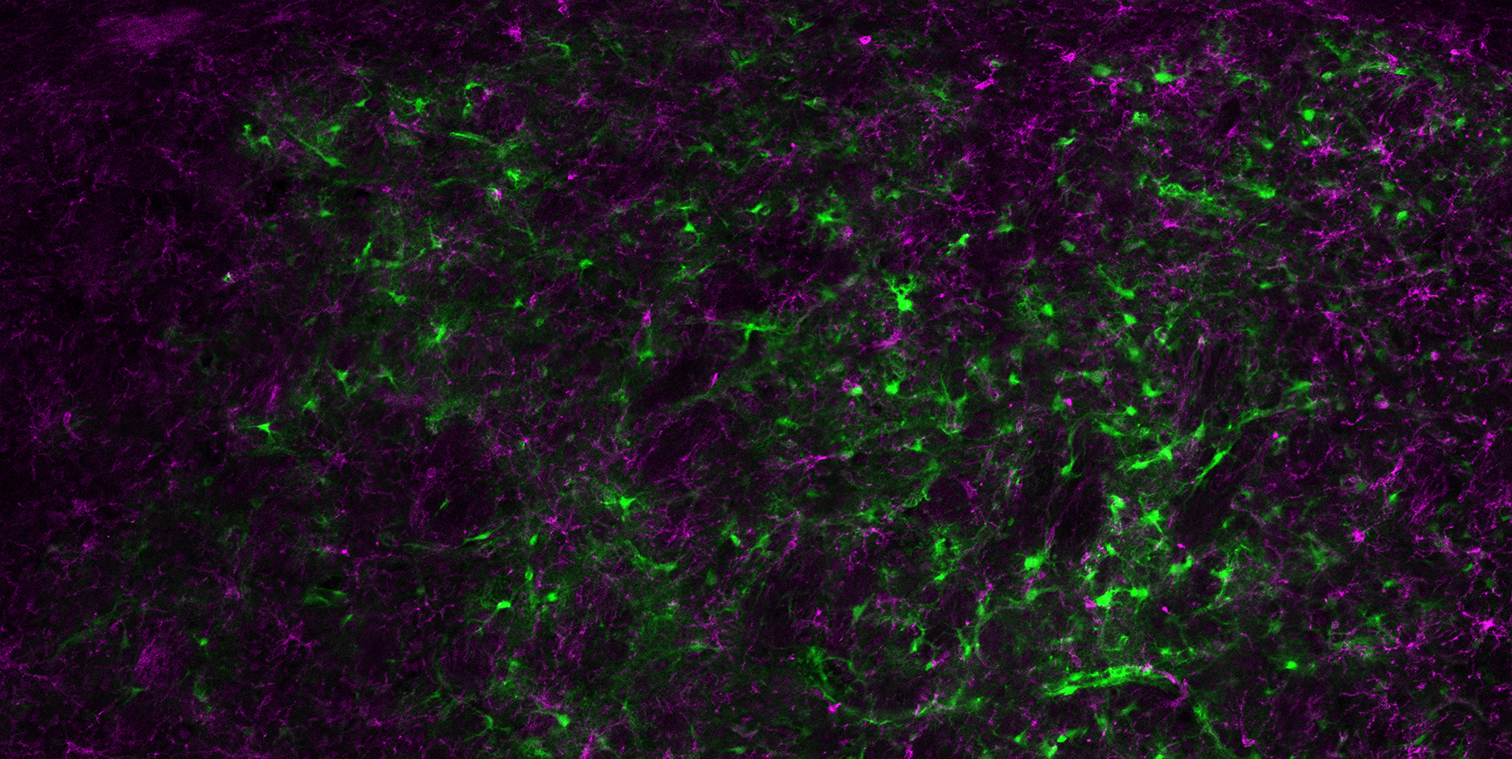

Supplement: Figure 1—figure supplement 1—source data 1. [file elife-75636-fig1-figsupp1-data1.zip › Fig1 source data 4 for Fig1 supplement 1/GFP+NG2 OR GFP+Iba-1/AAV-shPTB STR #12 GFP+NG2.jpg]

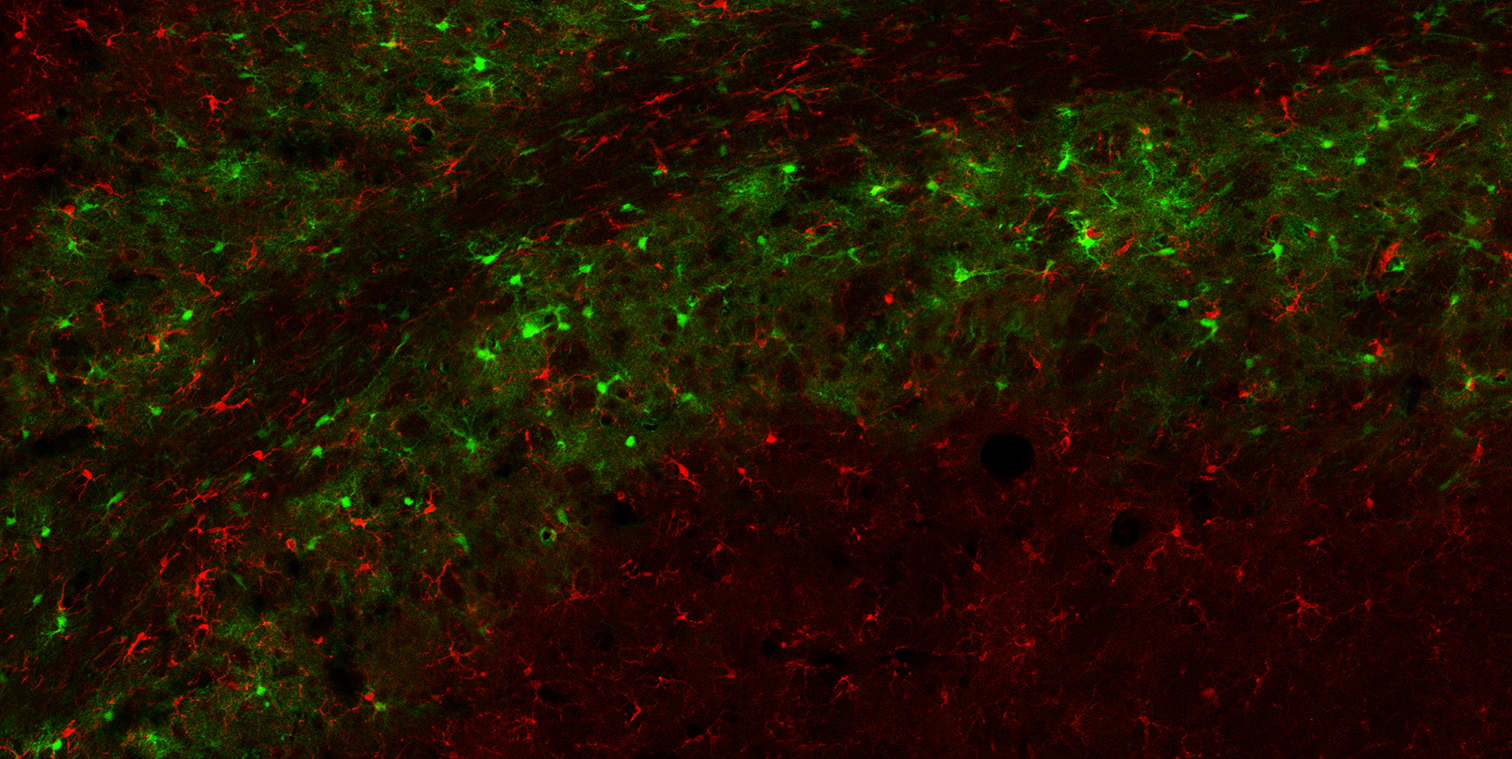

Supplement: Figure 1—figure supplement 1—source data 1. [file elife-75636-fig1-figsupp1-data1.zip › Fig1 source data 4 for Fig1 supplement 1/GFP+NG2 OR GFP+Iba-1/AAV-shPTB STR #53 GFP+Iba-1.jpg]

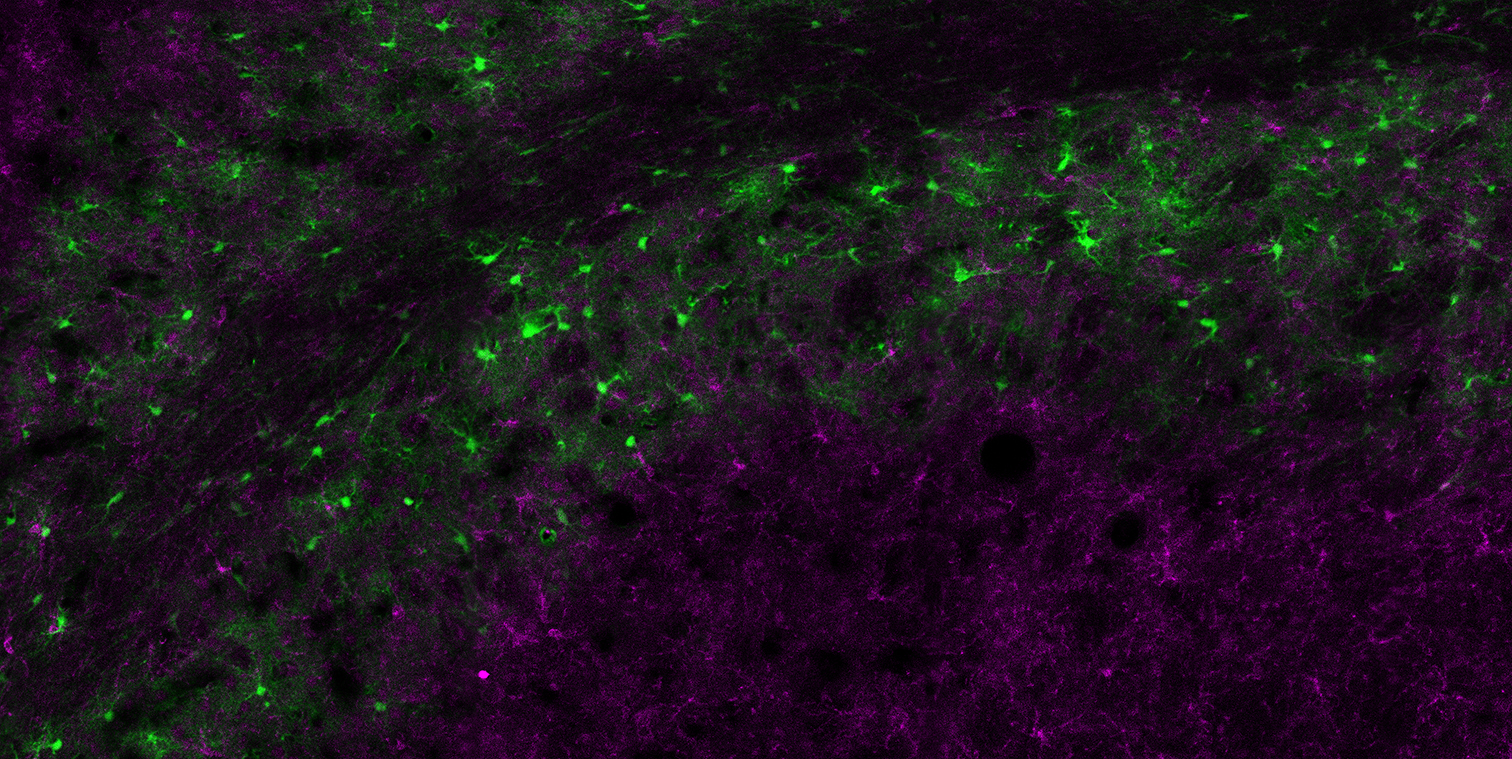

Supplement: Figure 1—figure supplement 1—source data 1. [file elife-75636-fig1-figsupp1-data1.zip › Fig1 source data 4 for Fig1 supplement 1/GFP+NG2 OR GFP+Iba-1/AAV-shPTB STR #53 GFP+NG2.jpg]

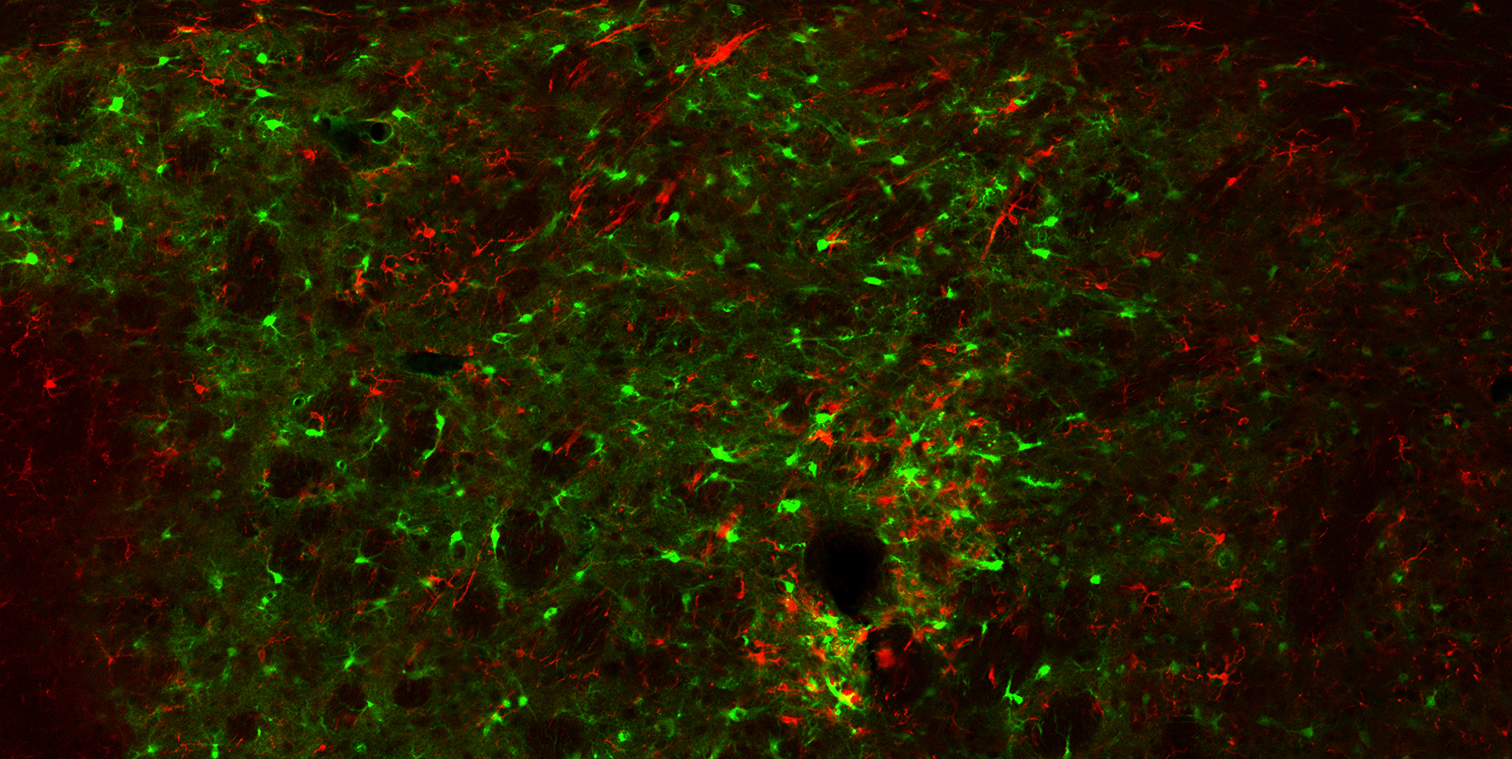

Supplement: Figure 1—figure supplement 1—source data 1. [file elife-75636-fig1-figsupp1-data1.zip › Fig1 source data 4 for Fig1 supplement 1/GFP+NG2 OR GFP+Iba-1/AAV-shPTB STR #98 GFP+Iba-1.jpg]

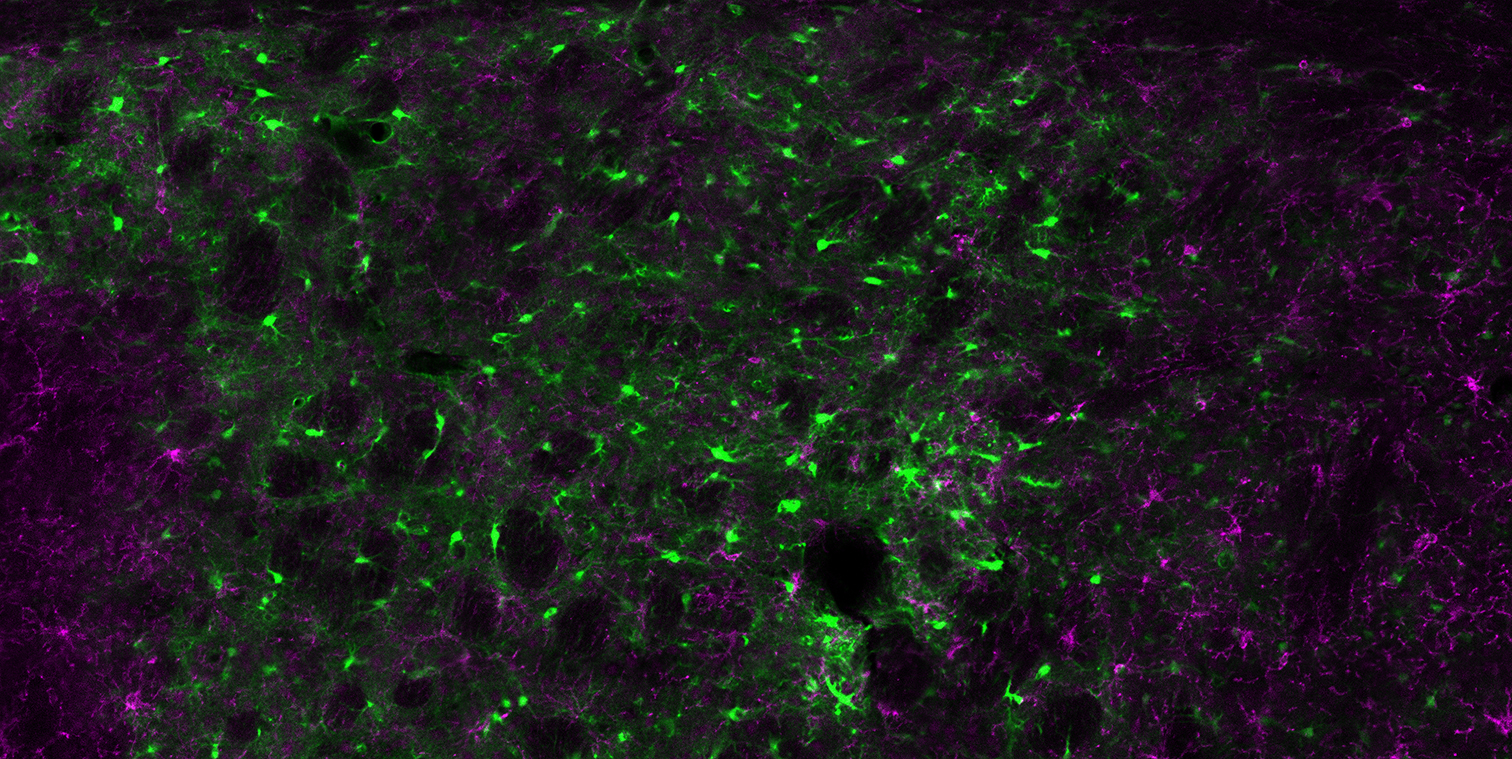

Supplement: Figure 1—figure supplement 1—source data 1. [file elife-75636-fig1-figsupp1-data1.zip › Fig1 source data 4 for Fig1 supplement 1/GFP+NG2 OR GFP+Iba-1/AAV-shPTB STR #98 GFP+NG2.jpg]

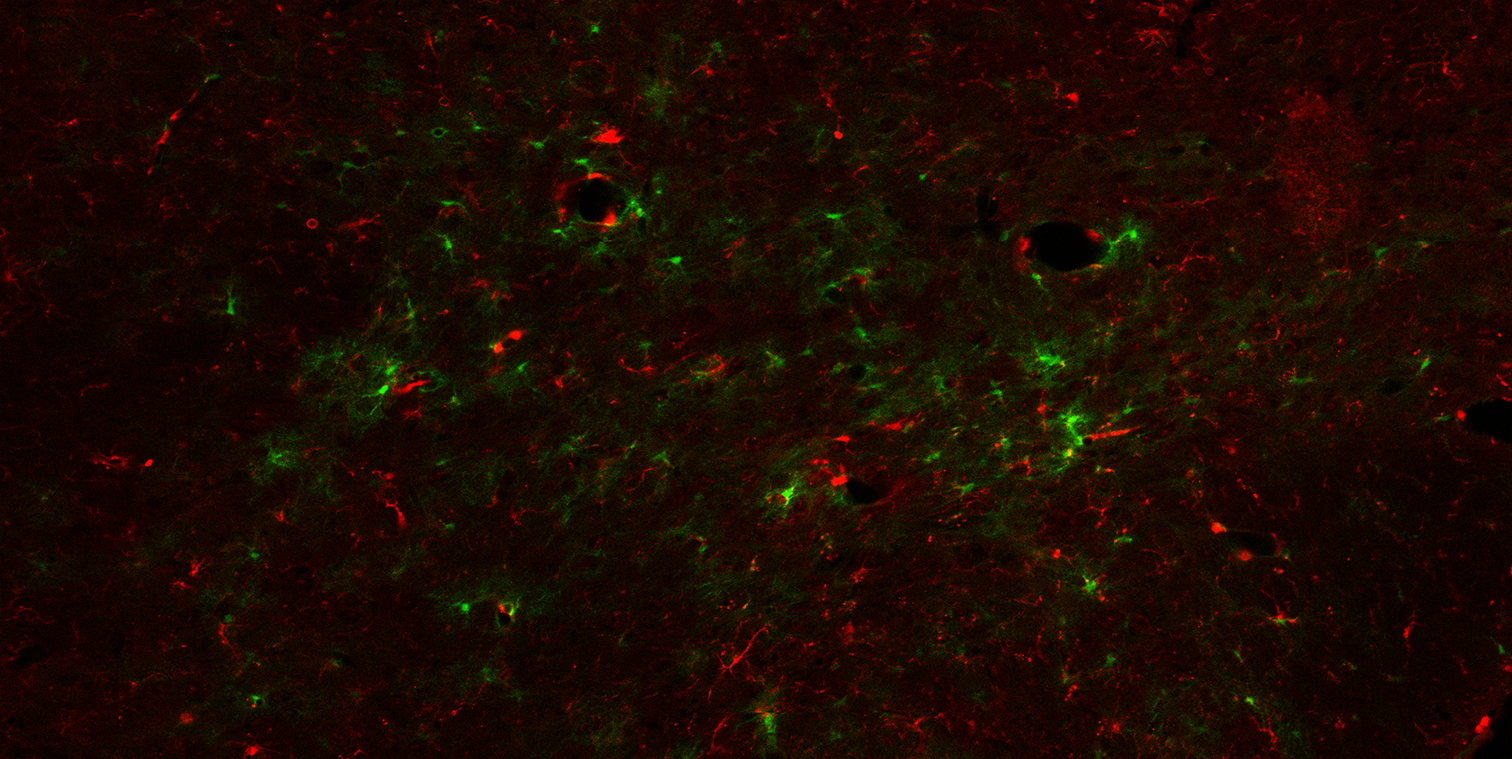

Supplement: Figure 1—figure supplement 1—source data 1. [file elife-75636-fig1-figsupp1-data1.zip › Fig1 source data 4 for Fig1 supplement 1/GFP+NG2 OR GFP+Iba-1/AAV-shscramble SN #10 GFP+Iba-1.jpg]

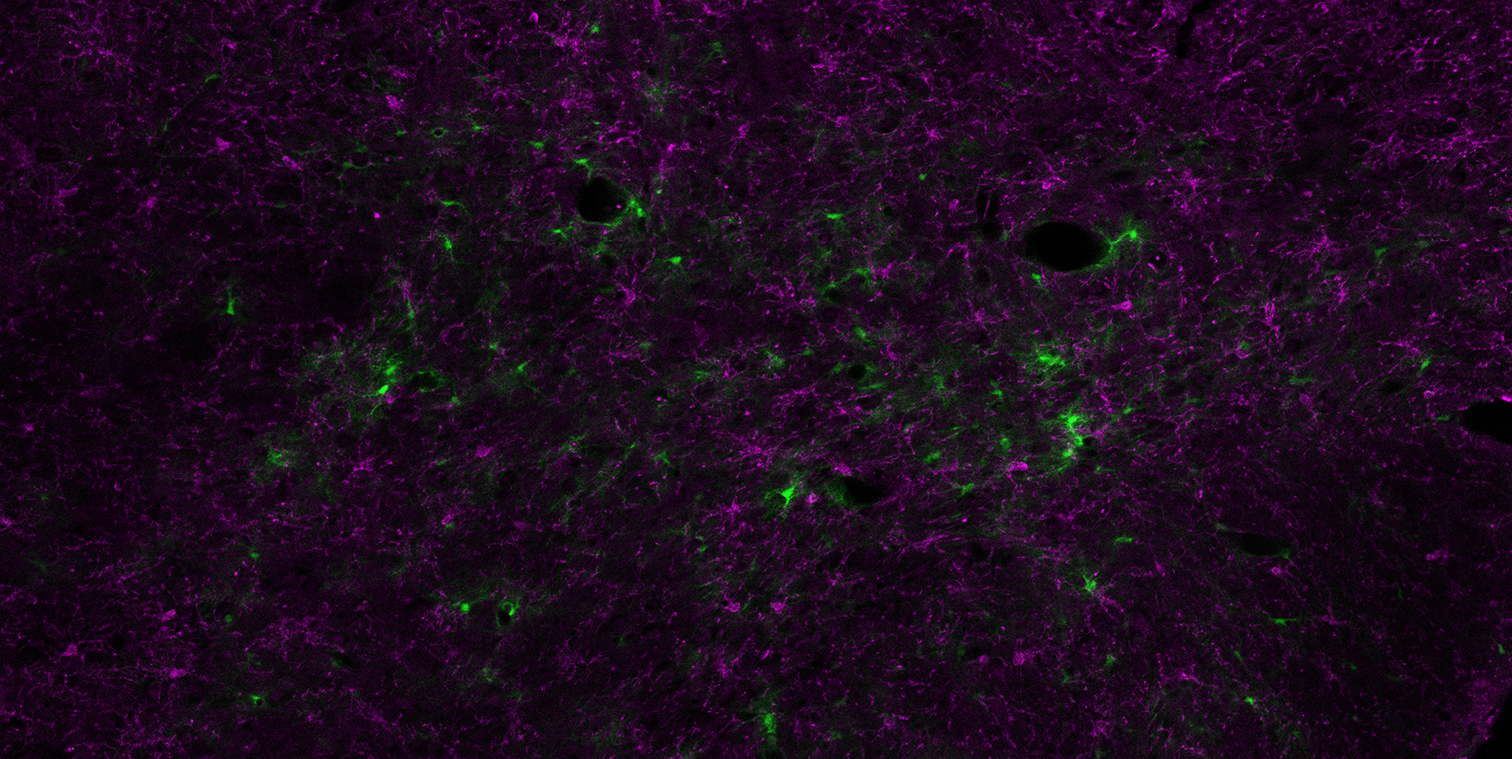

Supplement: Figure 1—figure supplement 1—source data 1. [file elife-75636-fig1-figsupp1-data1.zip › Fig1 source data 4 for Fig1 supplement 1/GFP+NG2 OR GFP+Iba-1/AAV-shscramble SN #10 GFP+NG2.jpg]

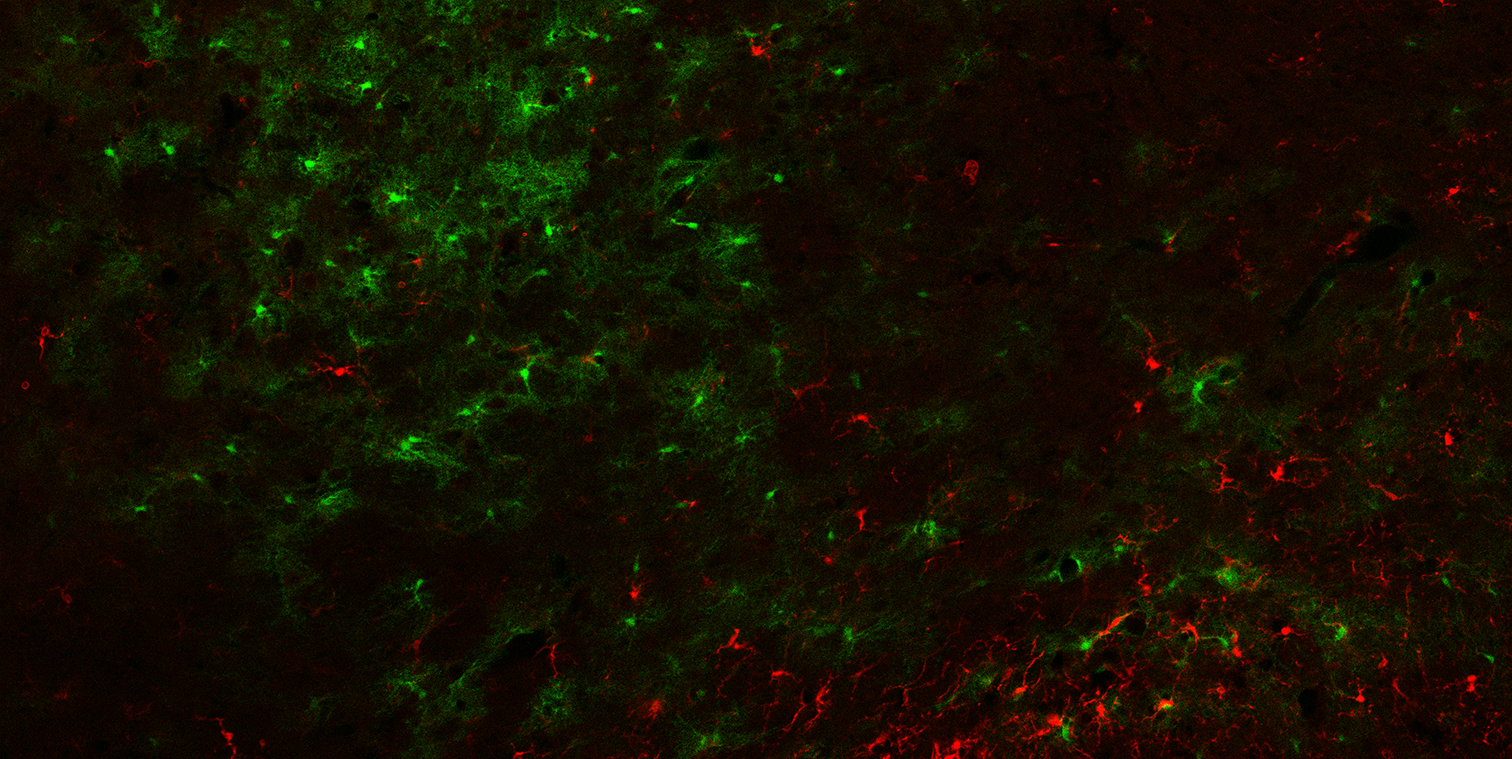

Supplement: Figure 1—figure supplement 1—source data 1. [file elife-75636-fig1-figsupp1-data1.zip › Fig1 source data 4 for Fig1 supplement 1/GFP+NG2 OR GFP+Iba-1/AAV-shscramble SN #13 GFP+Iba-1.jpg]

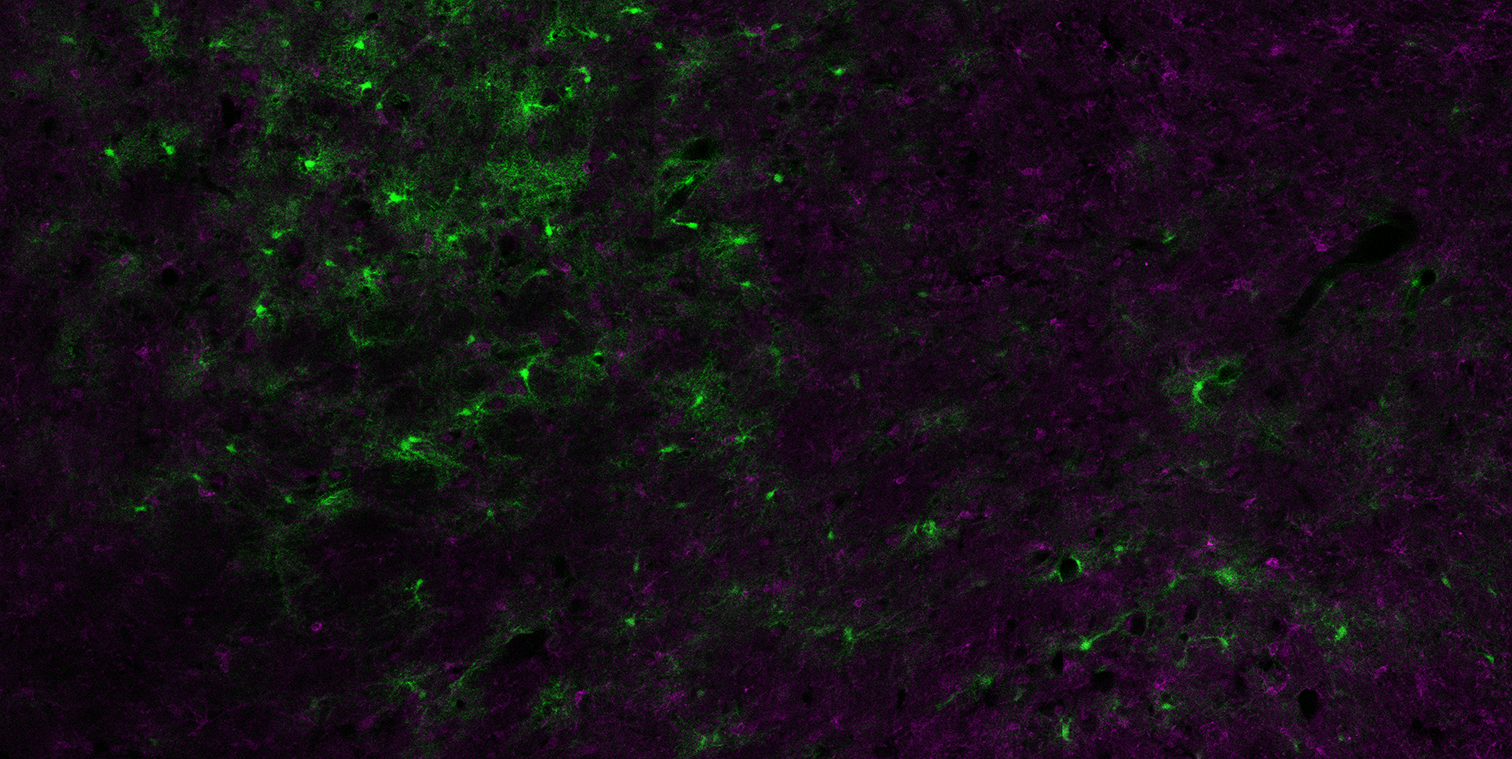

Supplement: Figure 1—figure supplement 1—source data 1. [file elife-75636-fig1-figsupp1-data1.zip › Fig1 source data 4 for Fig1 supplement 1/GFP+NG2 OR GFP+Iba-1/AAV-shscramble SN #13 GFP+NG2.jpg]

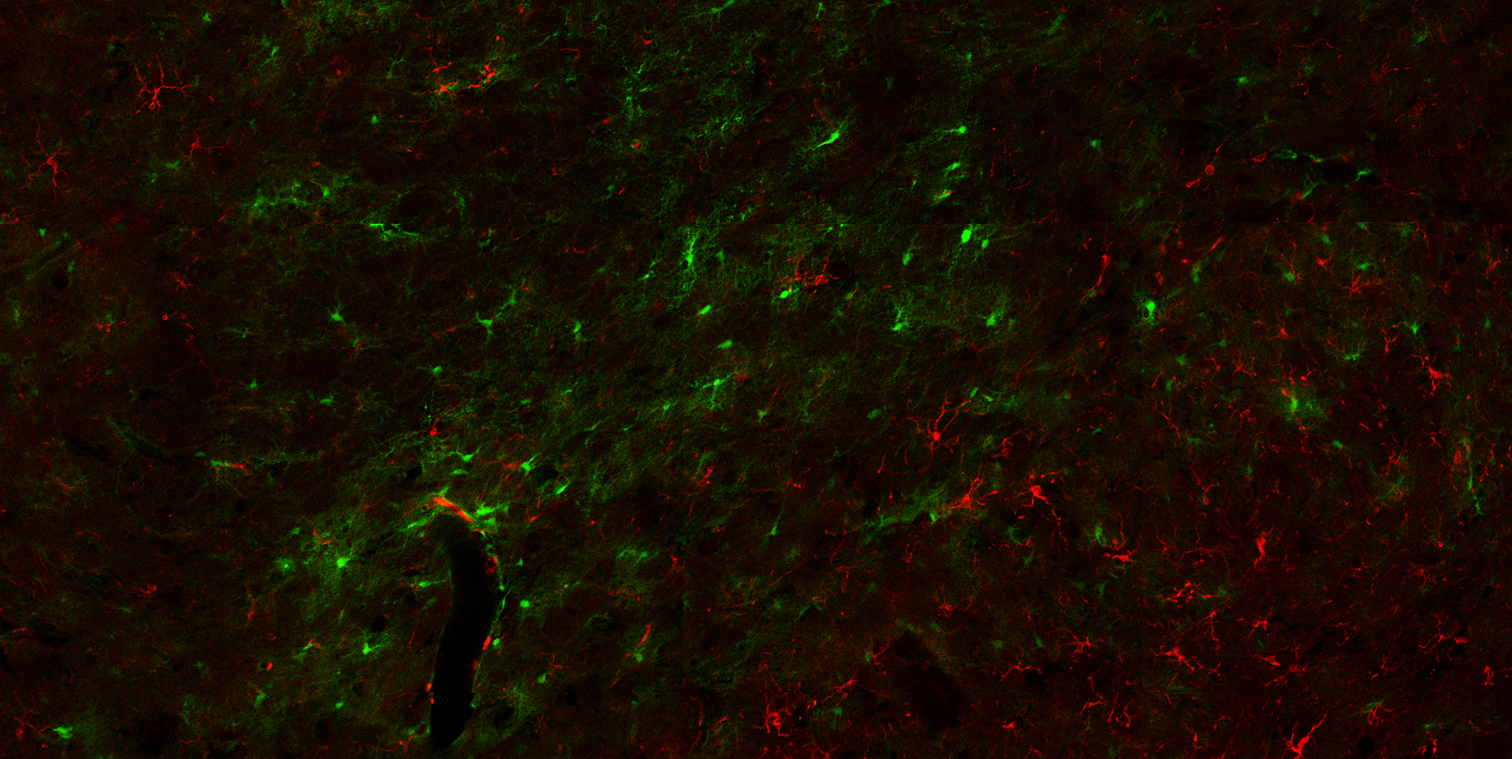

Supplement: Figure 1—figure supplement 1—source data 1. [file elife-75636-fig1-figsupp1-data1.zip › Fig1 source data 4 for Fig1 supplement 1/GFP+NG2 OR GFP+Iba-1/AAV-shscramble SN #9 GFP+Iba-1.jpg]

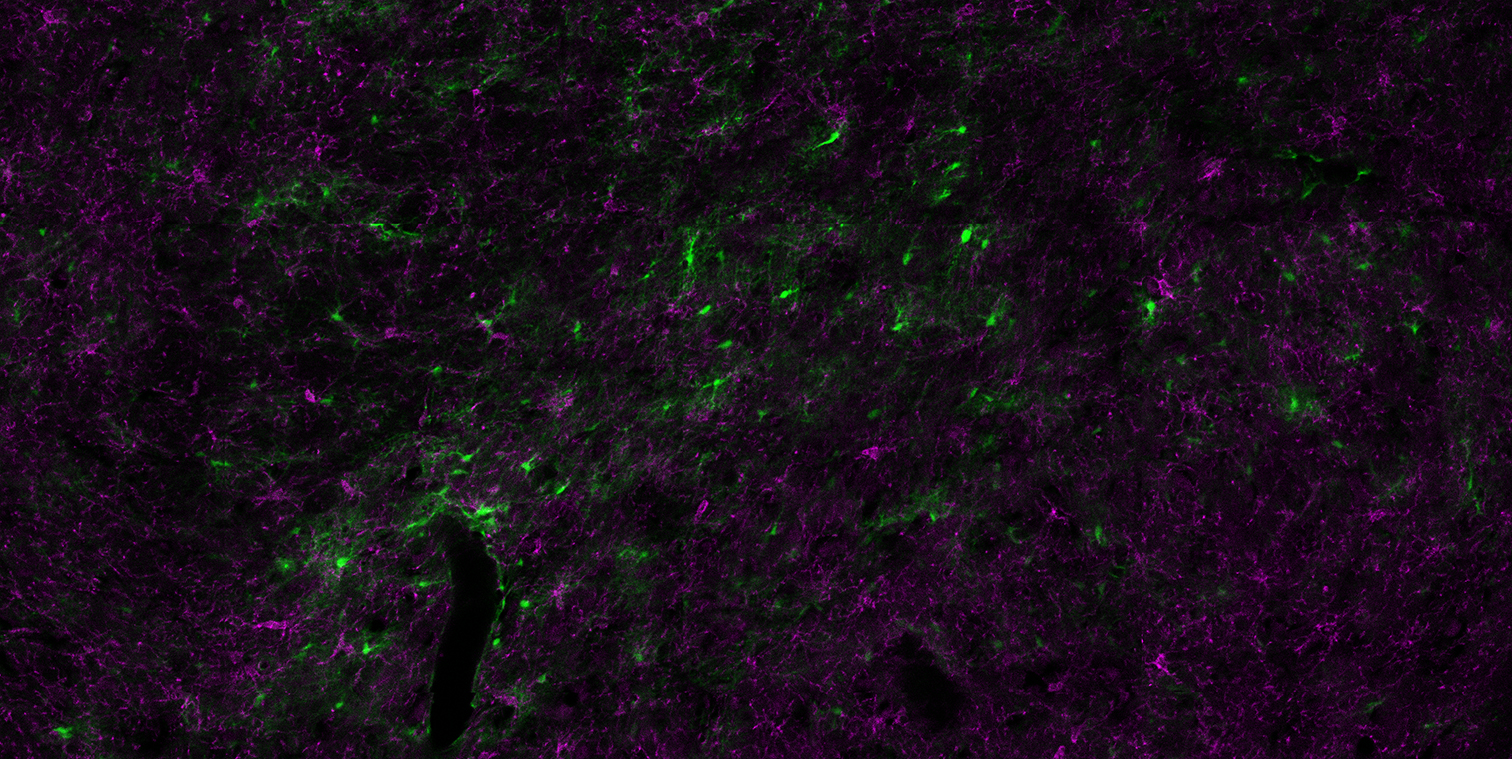

Supplement: Figure 1—figure supplement 1—source data 1. [file elife-75636-fig1-figsupp1-data1.zip › Fig1 source data 4 for Fig1 supplement 1/GFP+NG2 OR GFP+Iba-1/AAV-shscramble SN #9 GFP+NG2.jpg]

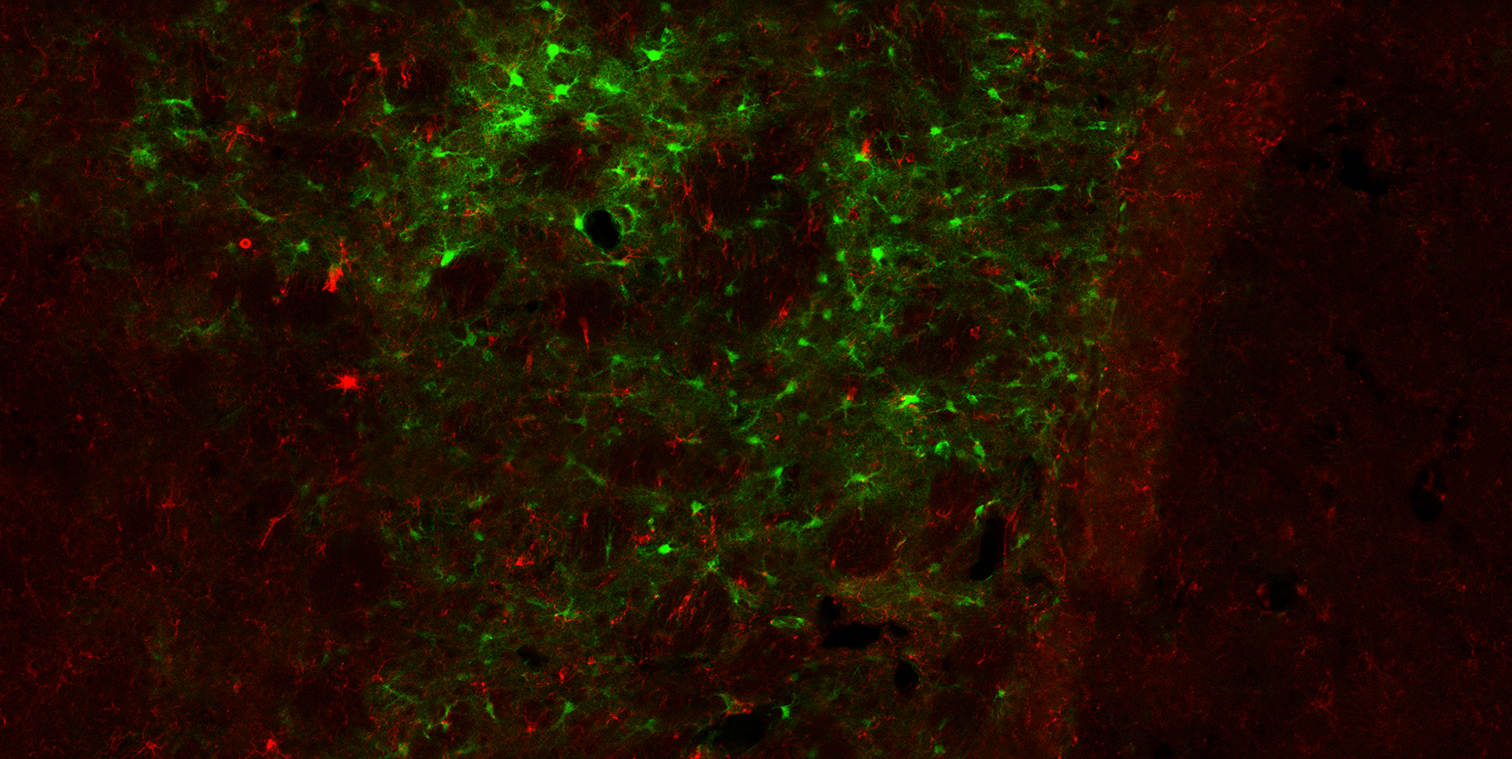

Supplement: Figure 1—figure supplement 1—source data 1. [file elife-75636-fig1-figsupp1-data1.zip › Fig1 source data 4 for Fig1 supplement 1/GFP+NG2 OR GFP+Iba-1/AAV-shscramble STR #10 GFP+Iba-1.jpg]

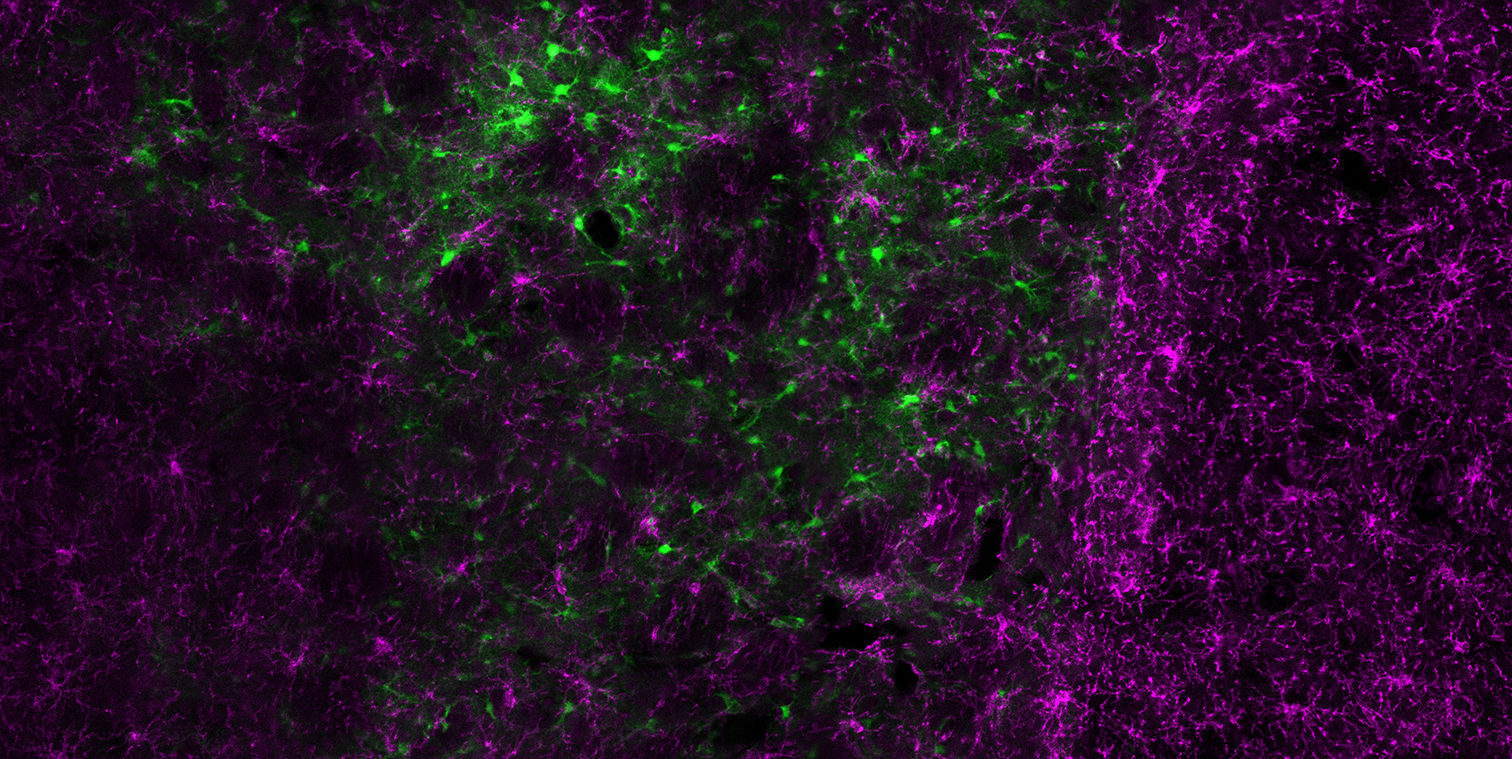

Supplement: Figure 1—figure supplement 1—source data 1. [file elife-75636-fig1-figsupp1-data1.zip › Fig1 source data 4 for Fig1 supplement 1/GFP+NG2 OR GFP+Iba-1/AAV-shscramble STR #10 GFP+NG2.jpg]

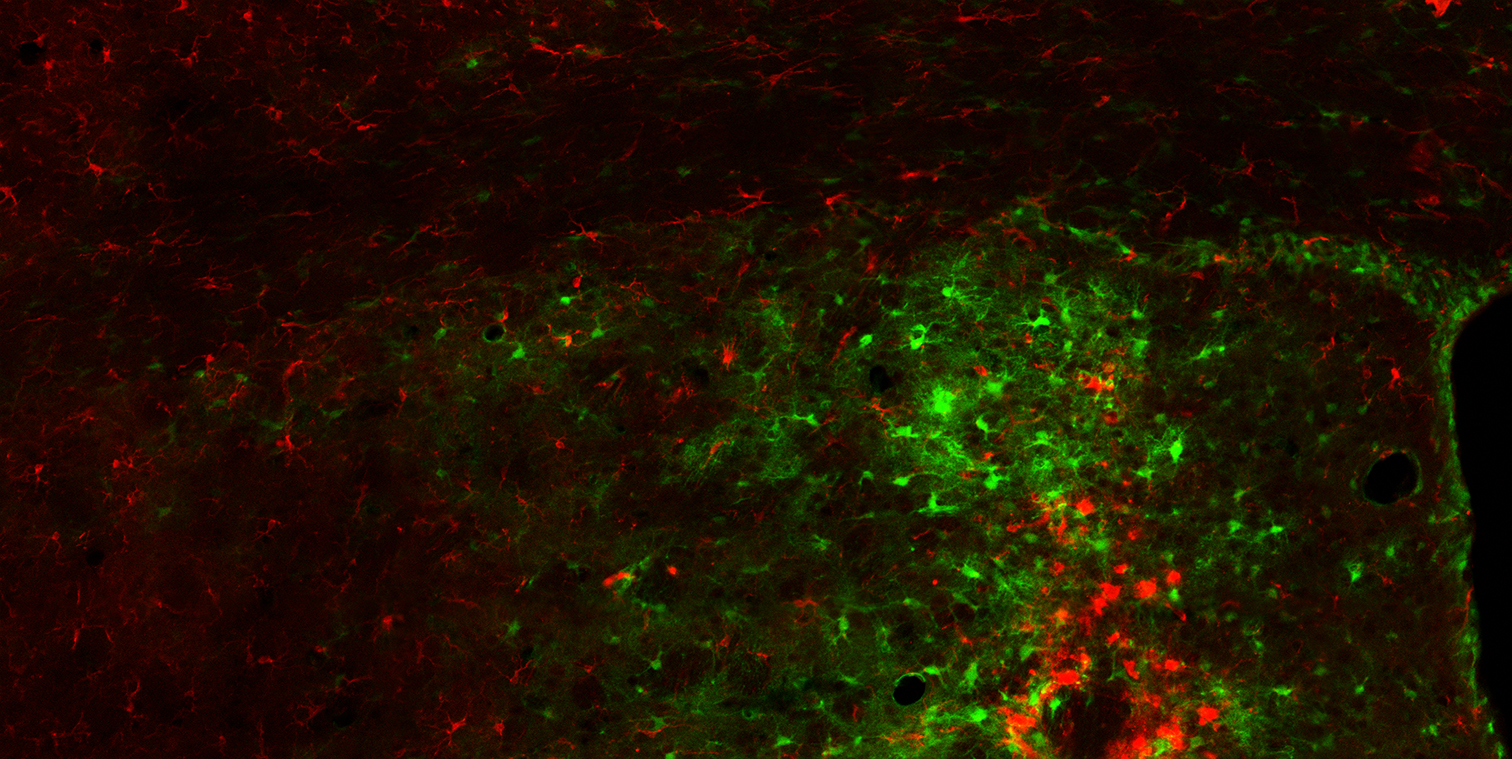

Supplement: Figure 1—figure supplement 1—source data 1. [file elife-75636-fig1-figsupp1-data1.zip › Fig1 source data 4 for Fig1 supplement 1/GFP+NG2 OR GFP+Iba-1/AAV-shscramble STR #13 GFP+Iba-1.jpg]

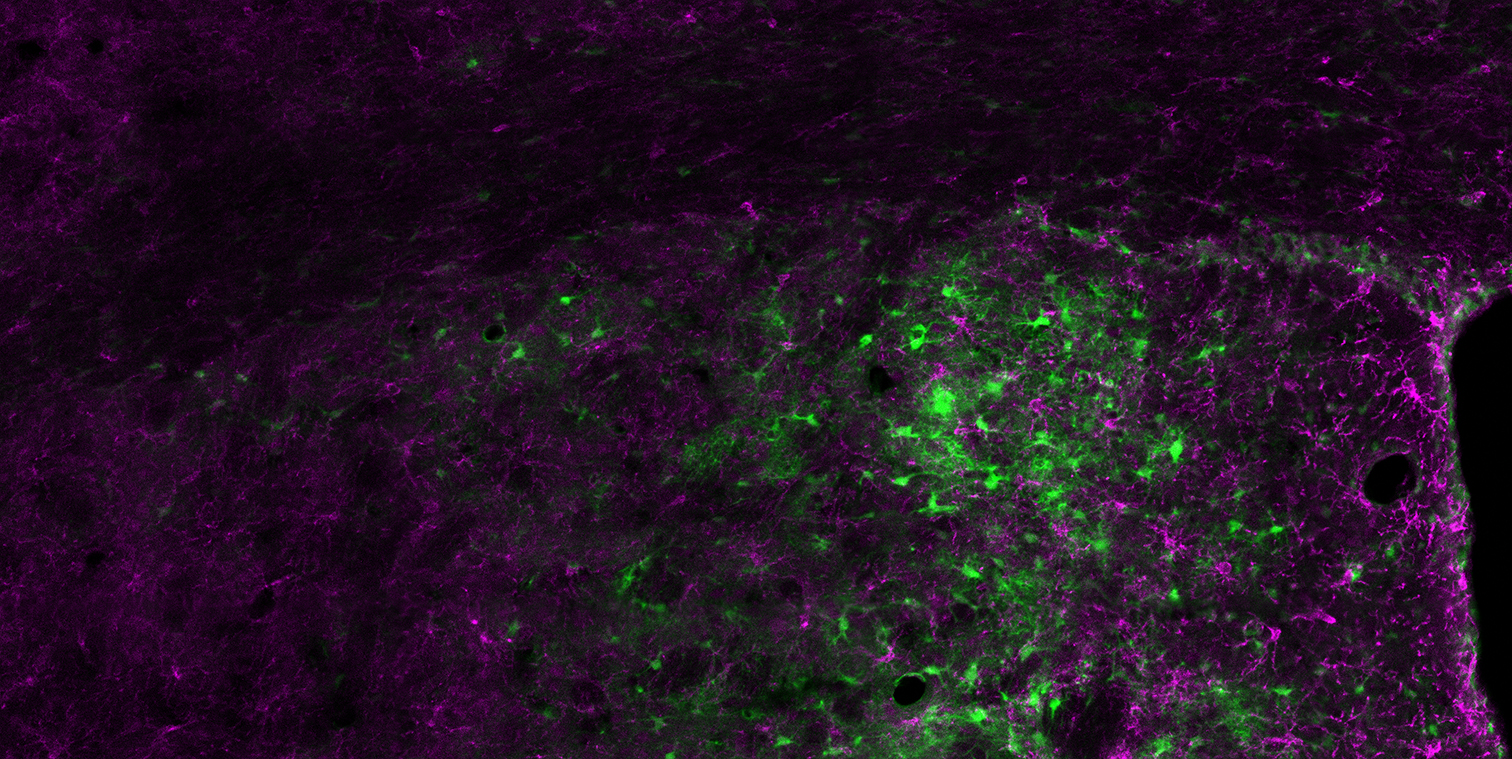

Supplement: Figure 1—figure supplement 1—source data 1. [file elife-75636-fig1-figsupp1-data1.zip › Fig1 source data 4 for Fig1 supplement 1/GFP+NG2 OR GFP+Iba-1/AAV-shscramble STR #13 GFP+NG2.jpg]

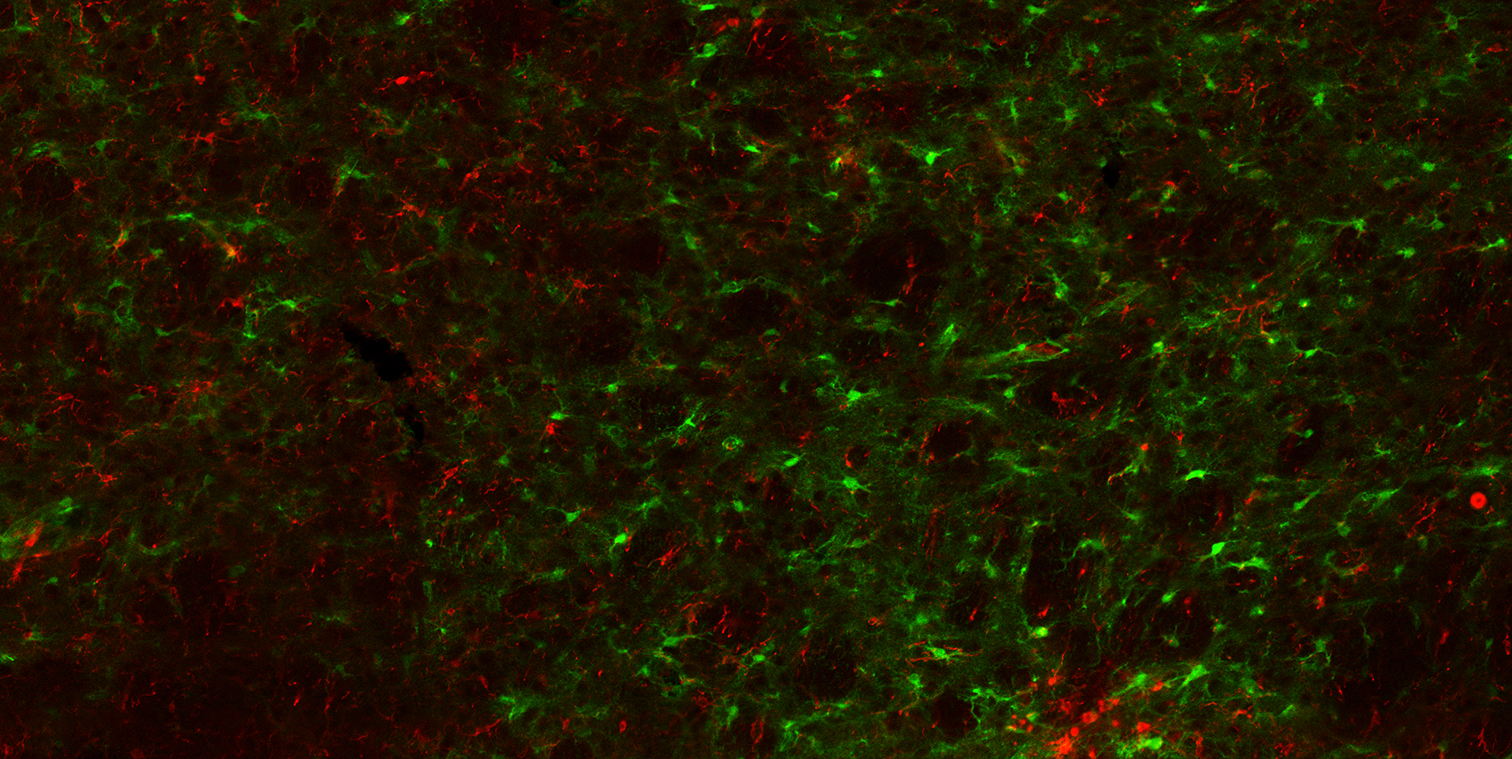

Supplement: Figure 1—figure supplement 1—source data 1. [file elife-75636-fig1-figsupp1-data1.zip › Fig1 source data 4 for Fig1 supplement 1/GFP+NG2 OR GFP+Iba-1/AAV-shscramble STR #9 GFP+Iba-1.jpg]

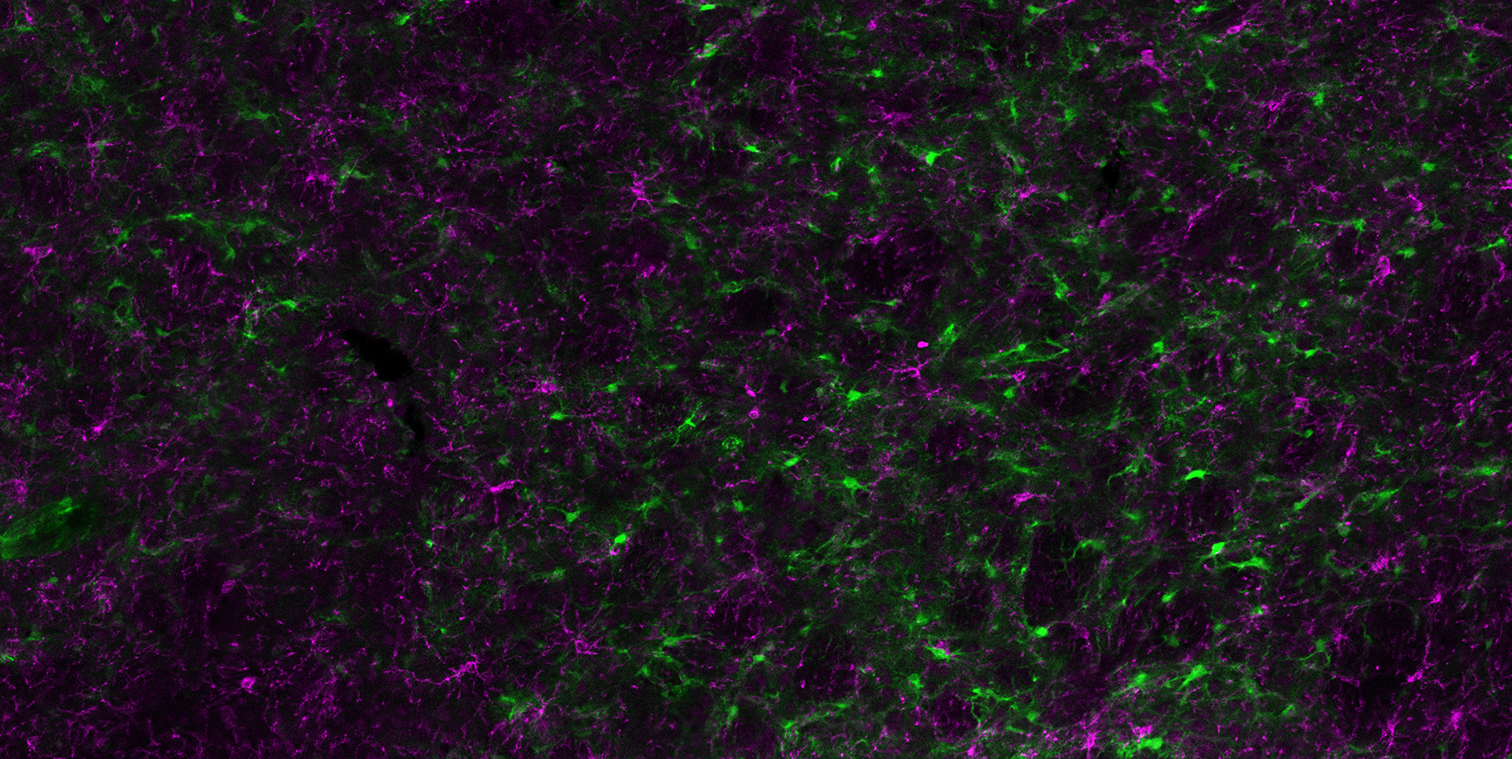

Supplement: Figure 1—figure supplement 1—source data 1. [file elife-75636-fig1-figsupp1-data1.zip › Fig1 source data 4 for Fig1 supplement 1/GFP+NG2 OR GFP+Iba-1/AAV-shscramble STR #9 GFP+NG2.jpg]

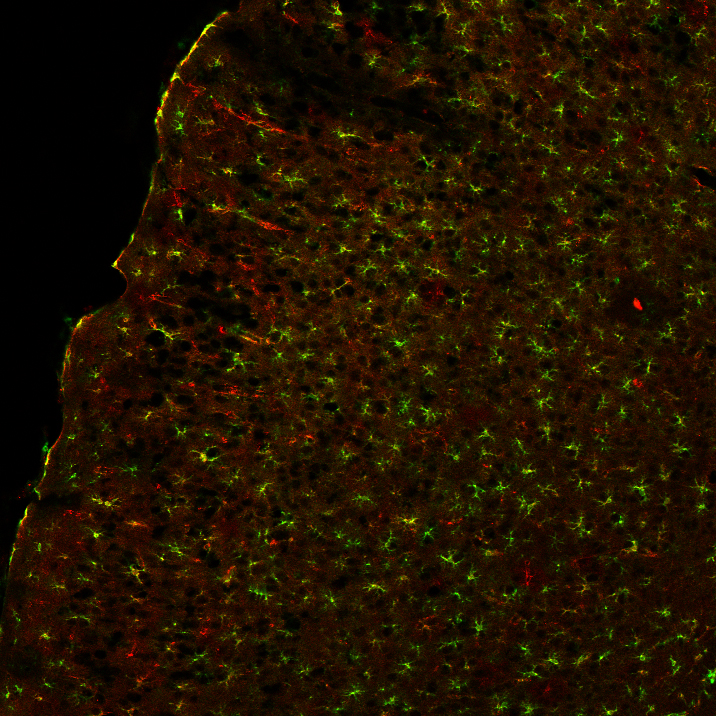

Supplement: Figure 2—source data 1. [file elife-75636-fig2-data1.zip › Fig2 source data 1 for Fig2 B/CORTEX HA+AldoC.jpg]

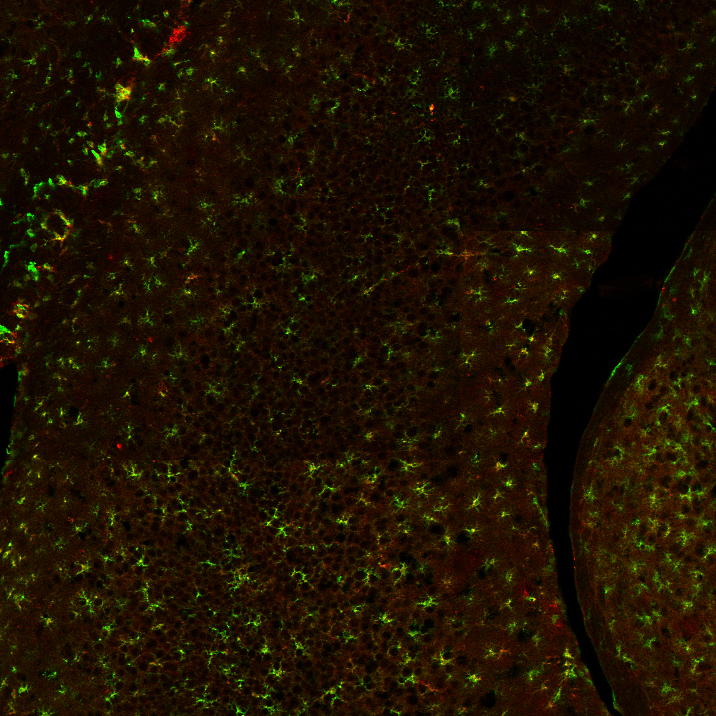

Supplement: Figure 2—source data 1. [file elife-75636-fig2-data1.zip › Fig2 source data 1 for Fig2 B/HIPPO HA+AldoC.jpg]

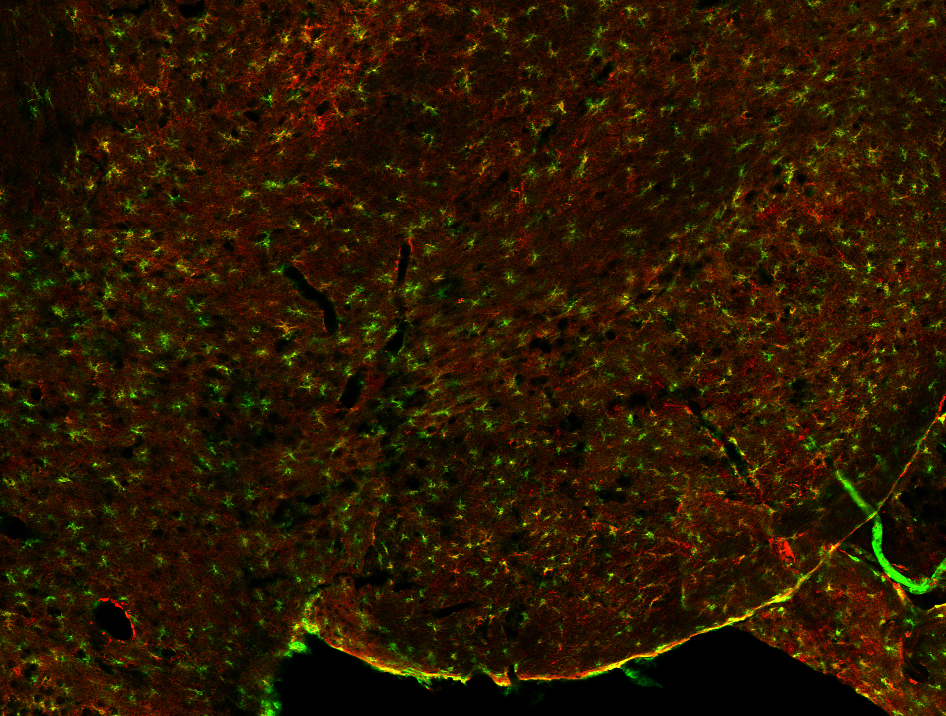

Supplement: Figure 2—source data 1. [file elife-75636-fig2-data1.zip › Fig2 source data 1 for Fig2 B/SN HA+AldoC.jpg]

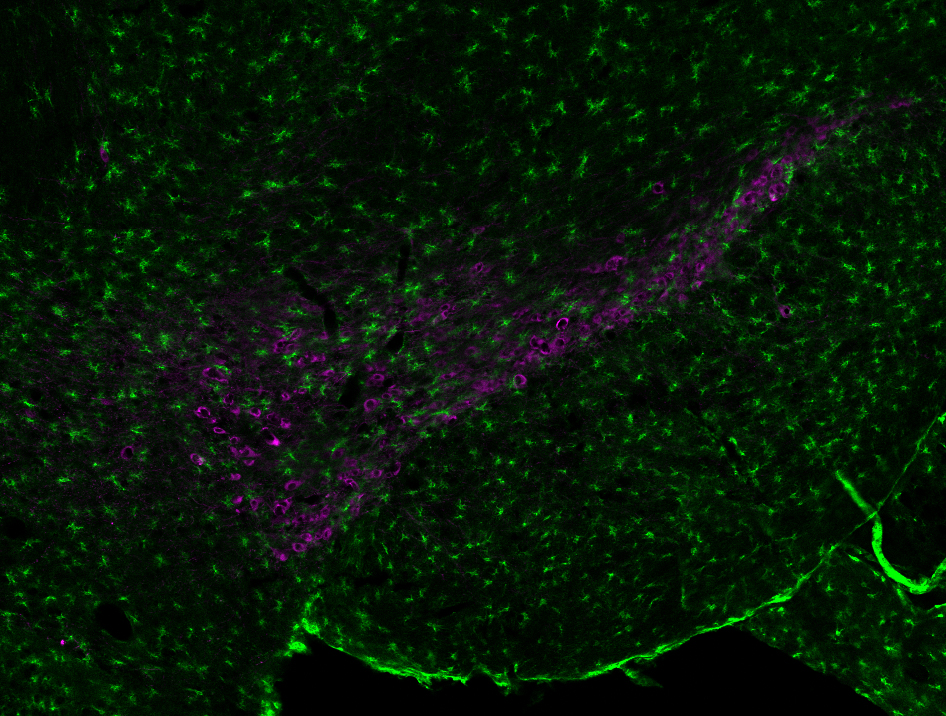

Supplement: Figure 2—source data 1. [file elife-75636-fig2-data1.zip › Fig2 source data 1 for Fig2 B/SN HA+TH.jpg]

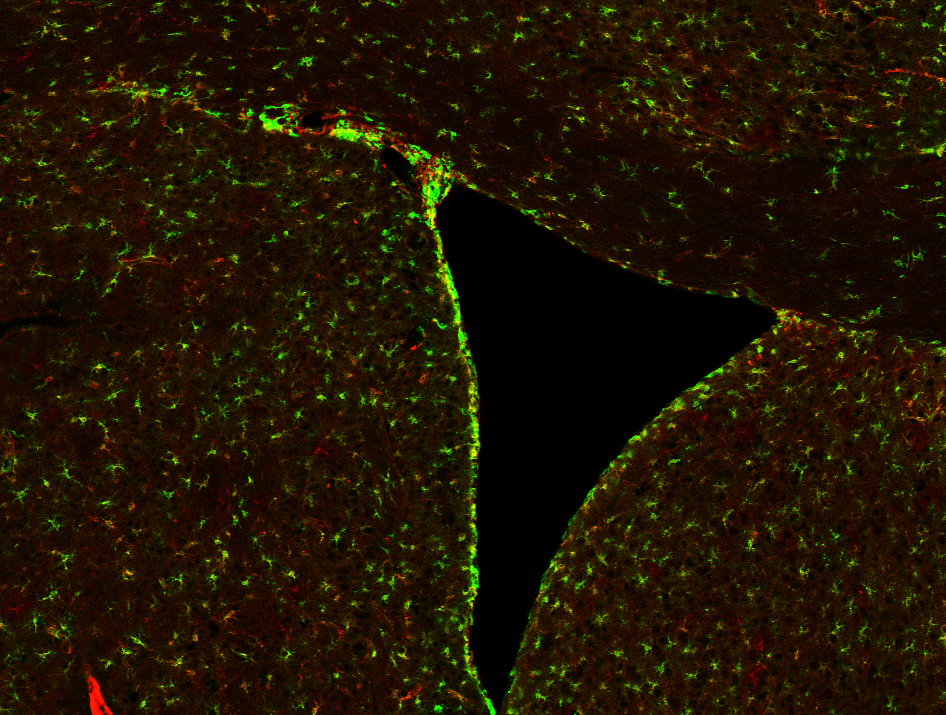

Supplement: Figure 2—source data 1. [file elife-75636-fig2-data1.zip › Fig2 source data 1 for Fig2 B/STR HA+AldoC.jpg]

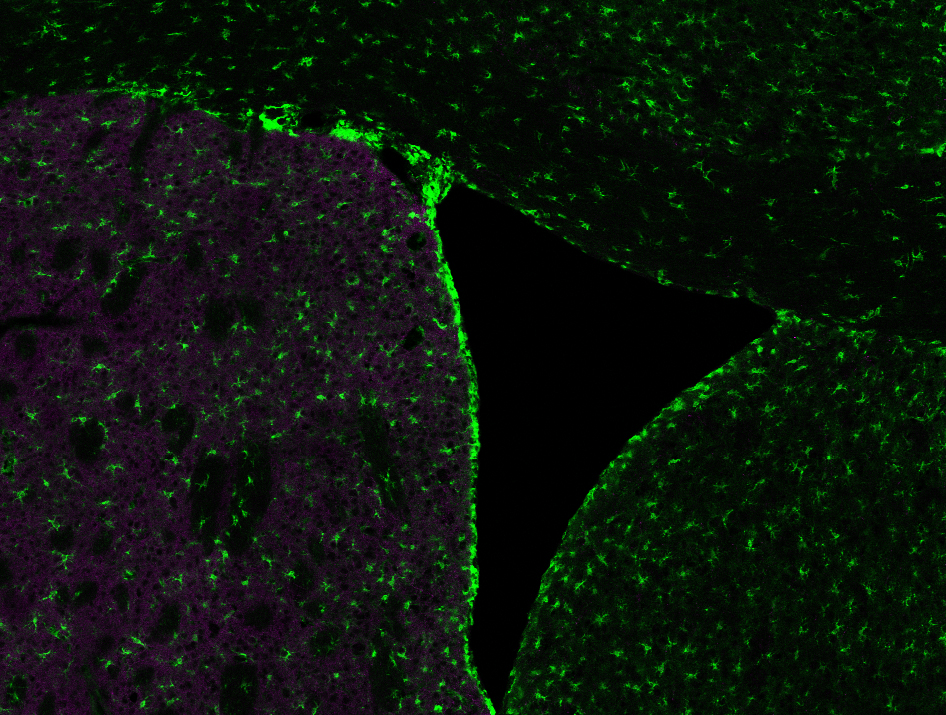

Supplement: Figure 2—source data 1. [file elife-75636-fig2-data1.zip › Fig2 source data 1 for Fig2 B/STR HA+TH.jpg]

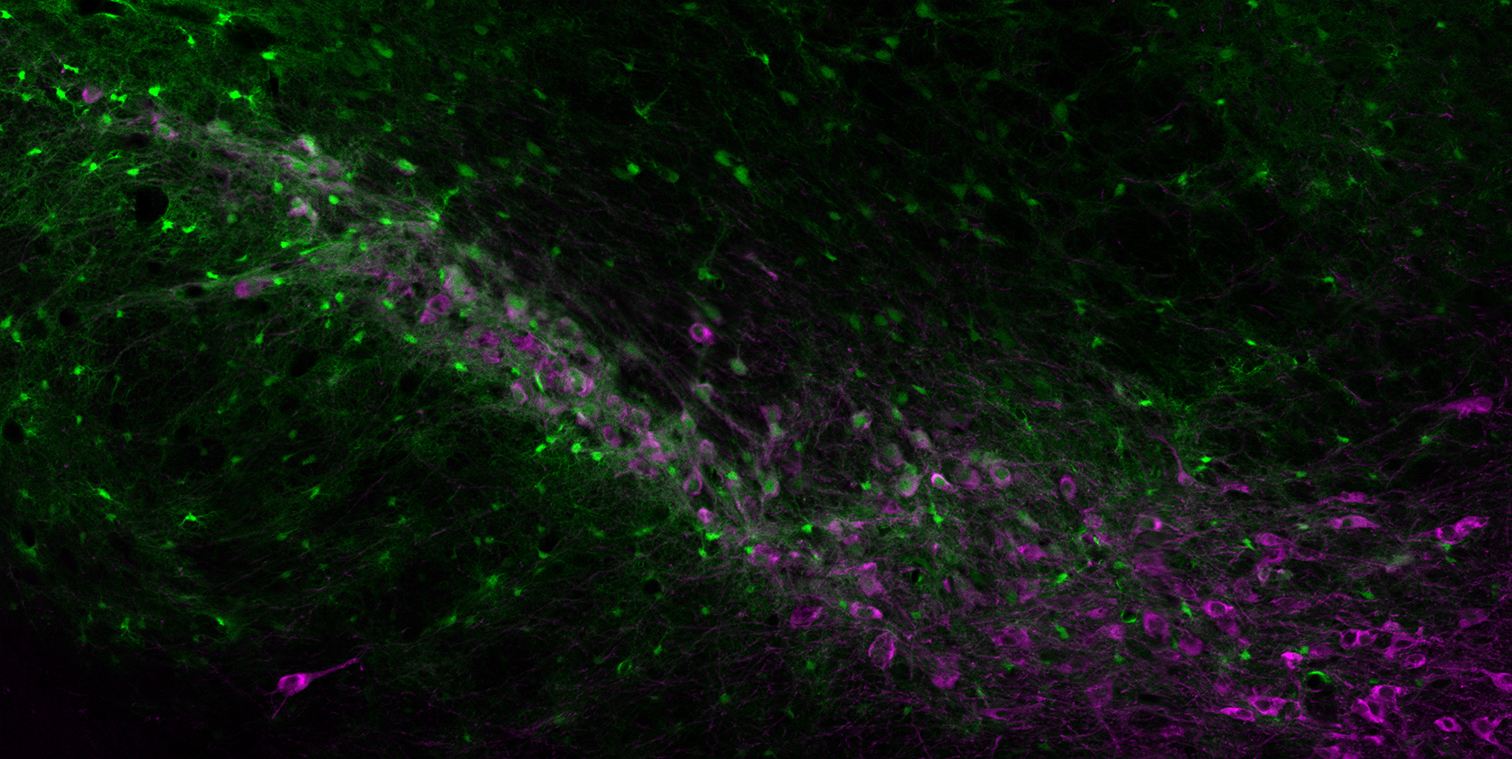

Supplement: Figure 2—source data 2. [file elife-75636-fig2-data2.zip › Fig2 source data 2 for Fig2 D/AAV-shPTB SN MZ1 GFP+TH.jpg]

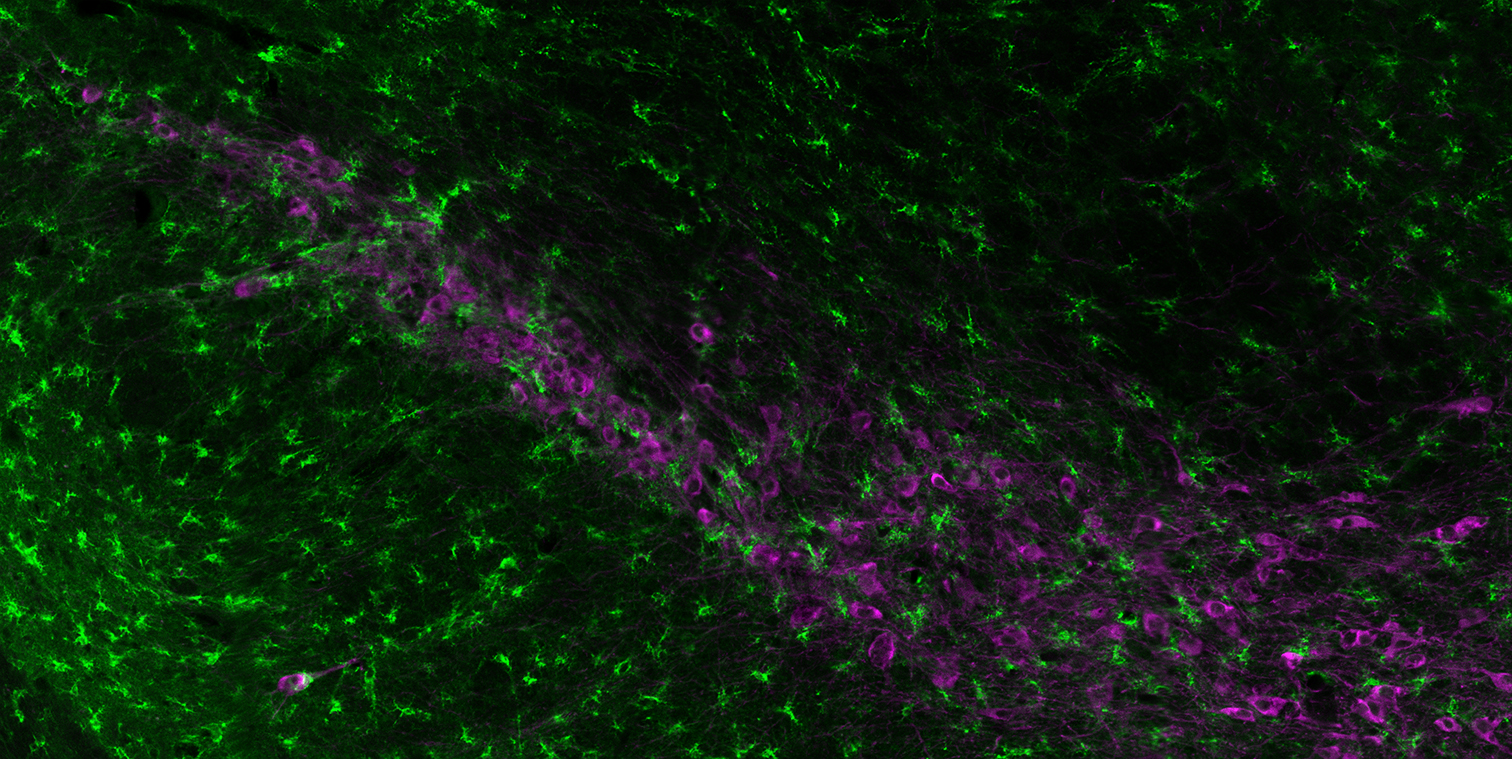

Supplement: Figure 2—source data 2. [file elife-75636-fig2-data2.zip › Fig2 source data 2 for Fig2 D/AAV-shPTB SN MZ1 HA+TH.jpg]

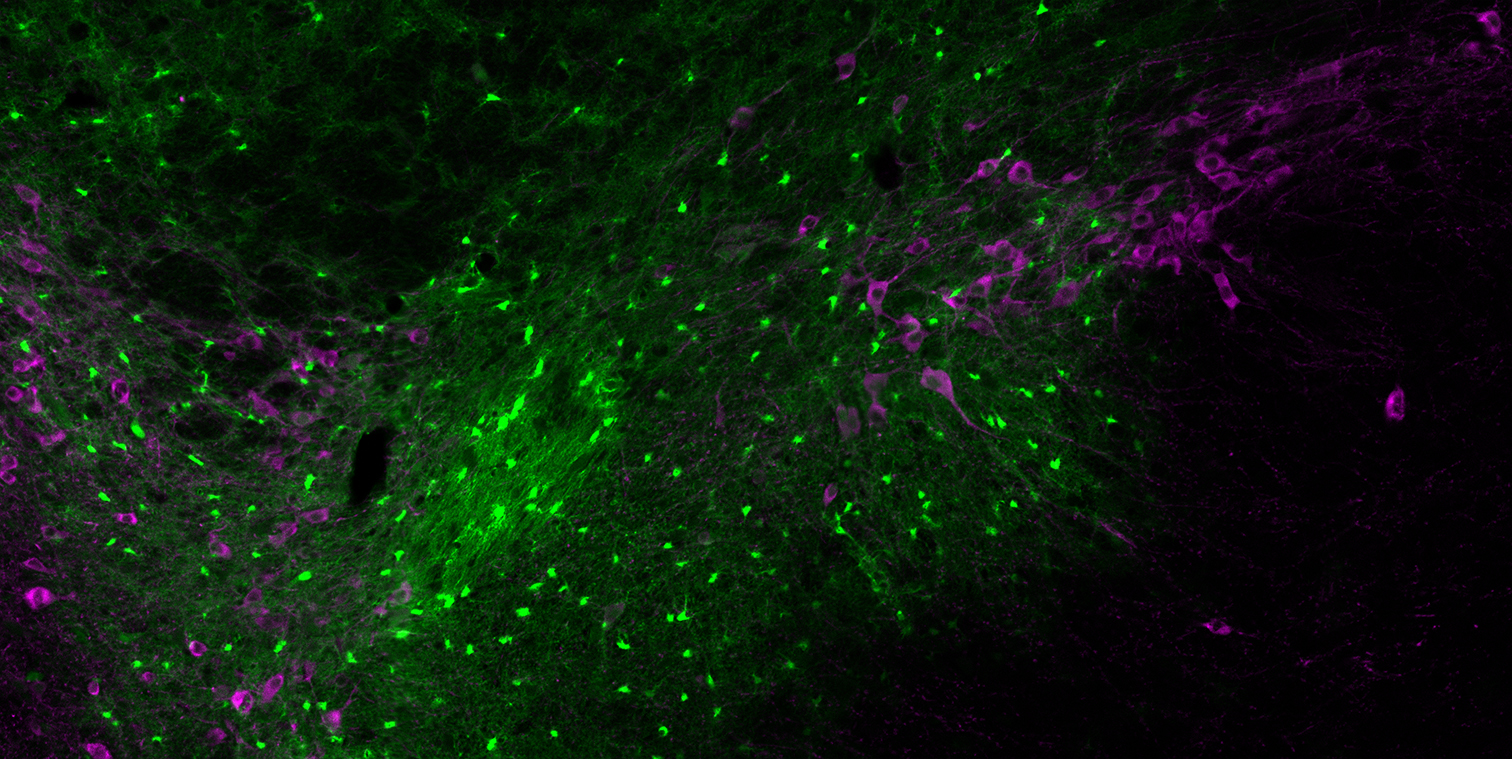

Supplement: Figure 2—source data 2. [file elife-75636-fig2-data2.zip › Fig2 source data 2 for Fig2 D/AAV-shPTB SN MZ2 GFP+TH.jpg]

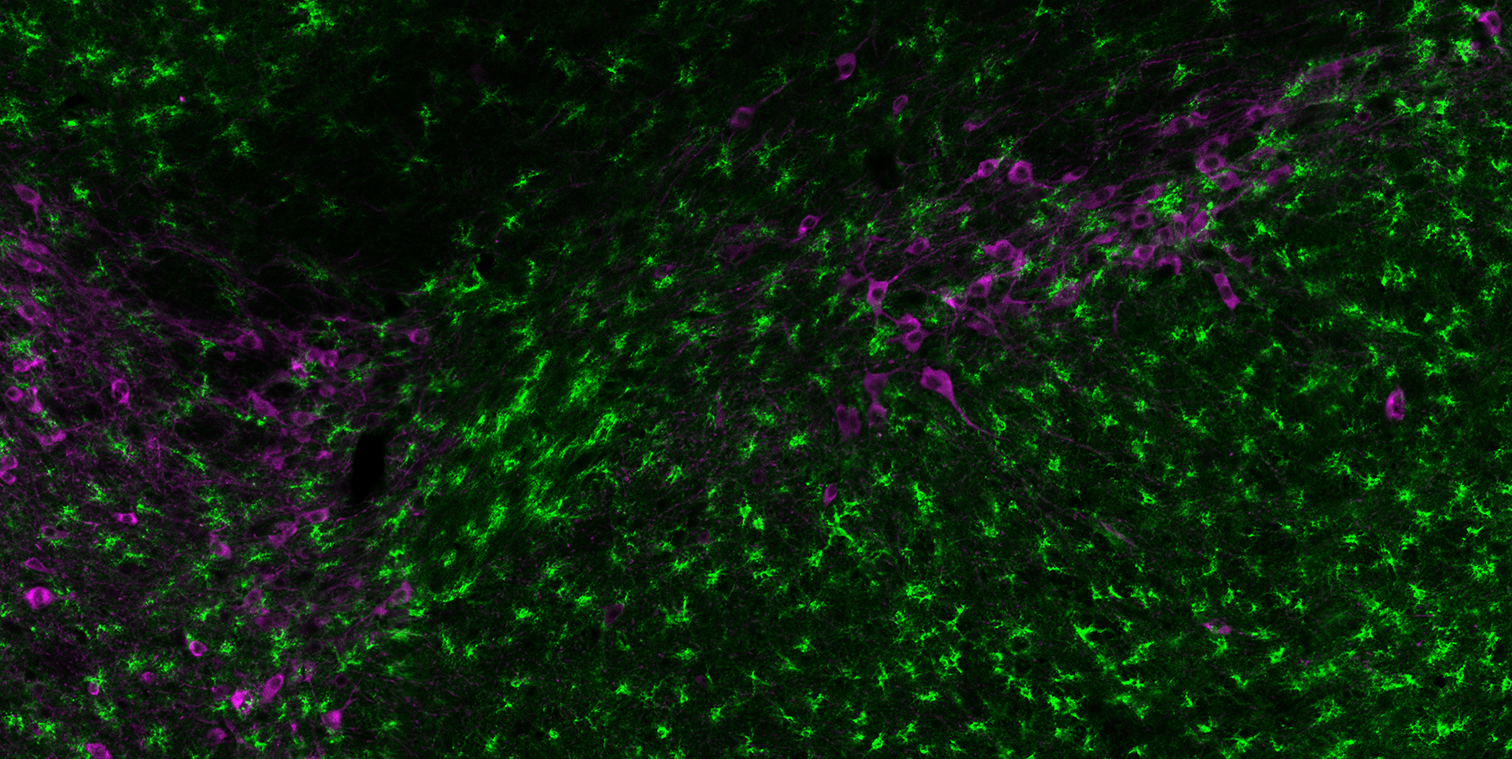

Supplement: Figure 2—source data 2. [file elife-75636-fig2-data2.zip › Fig2 source data 2 for Fig2 D/AAV-shPTB SN MZ2 HA+TH.jpg]

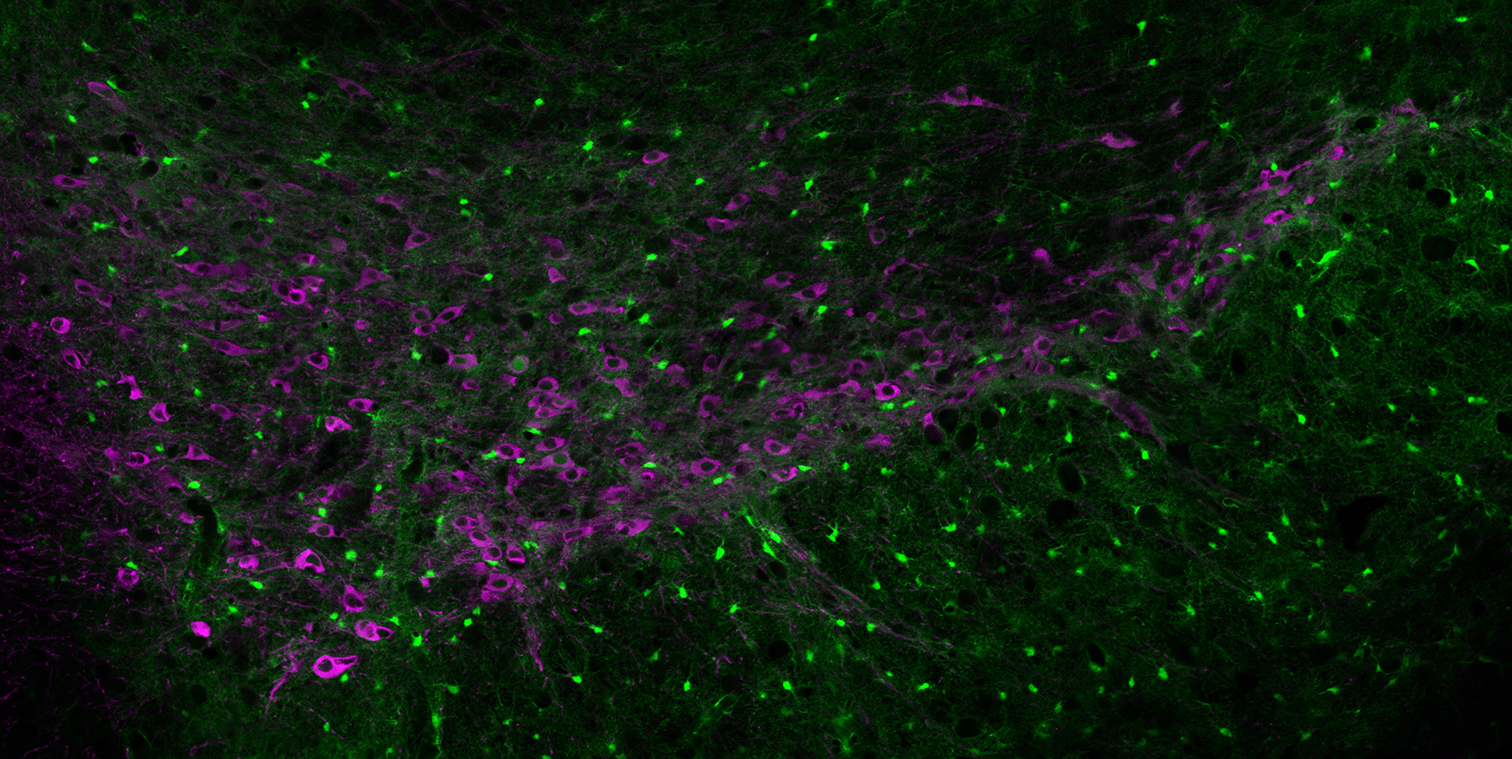

Supplement: Figure 2—source data 2. [file elife-75636-fig2-data2.zip › Fig2 source data 2 for Fig2 D/AAV-shPTB SN MZ3 GFP+TH.jpg]

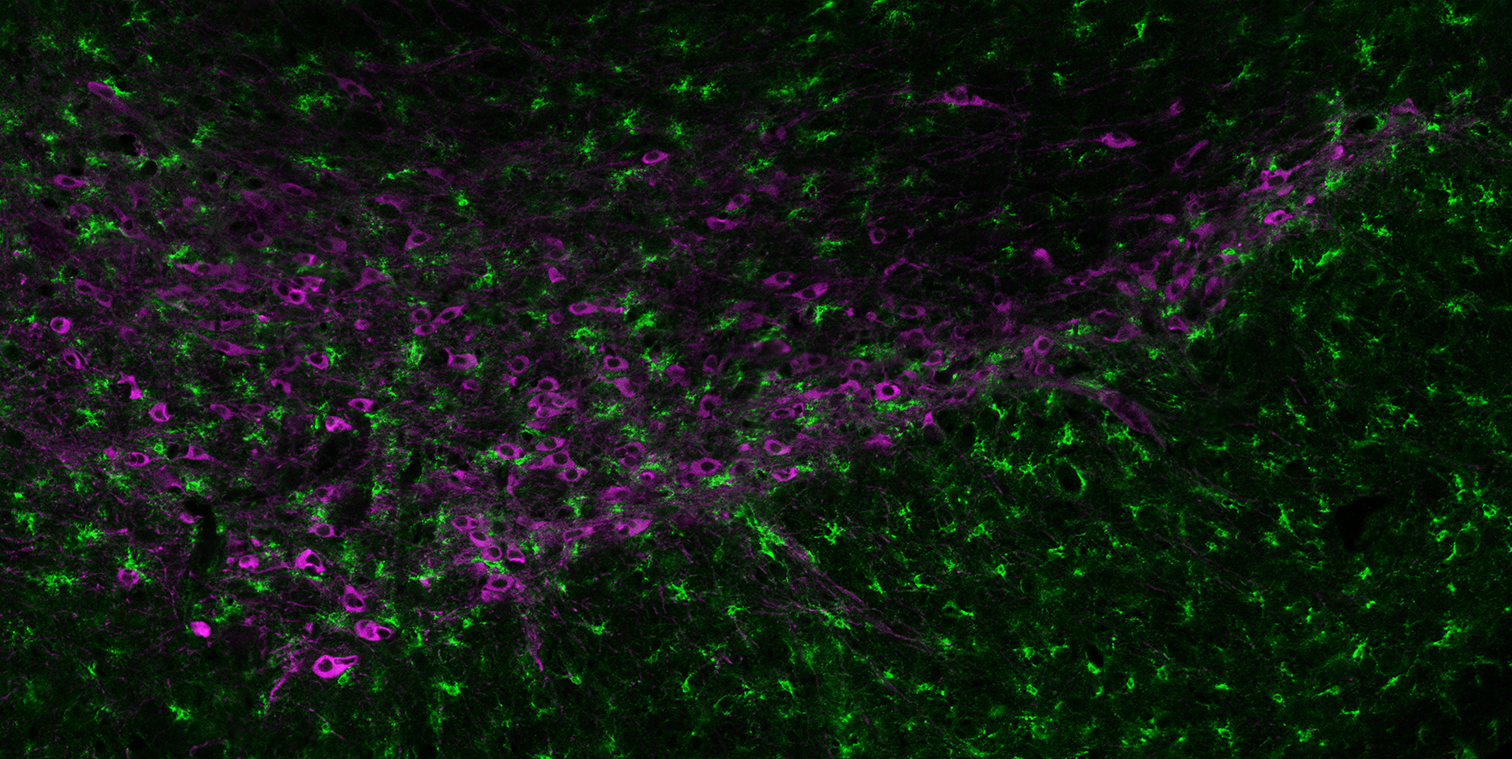

Supplement: Figure 2—source data 2. [file elife-75636-fig2-data2.zip › Fig2 source data 2 for Fig2 D/AAV-shPTB SN MZ3 HA+TH.jpg]

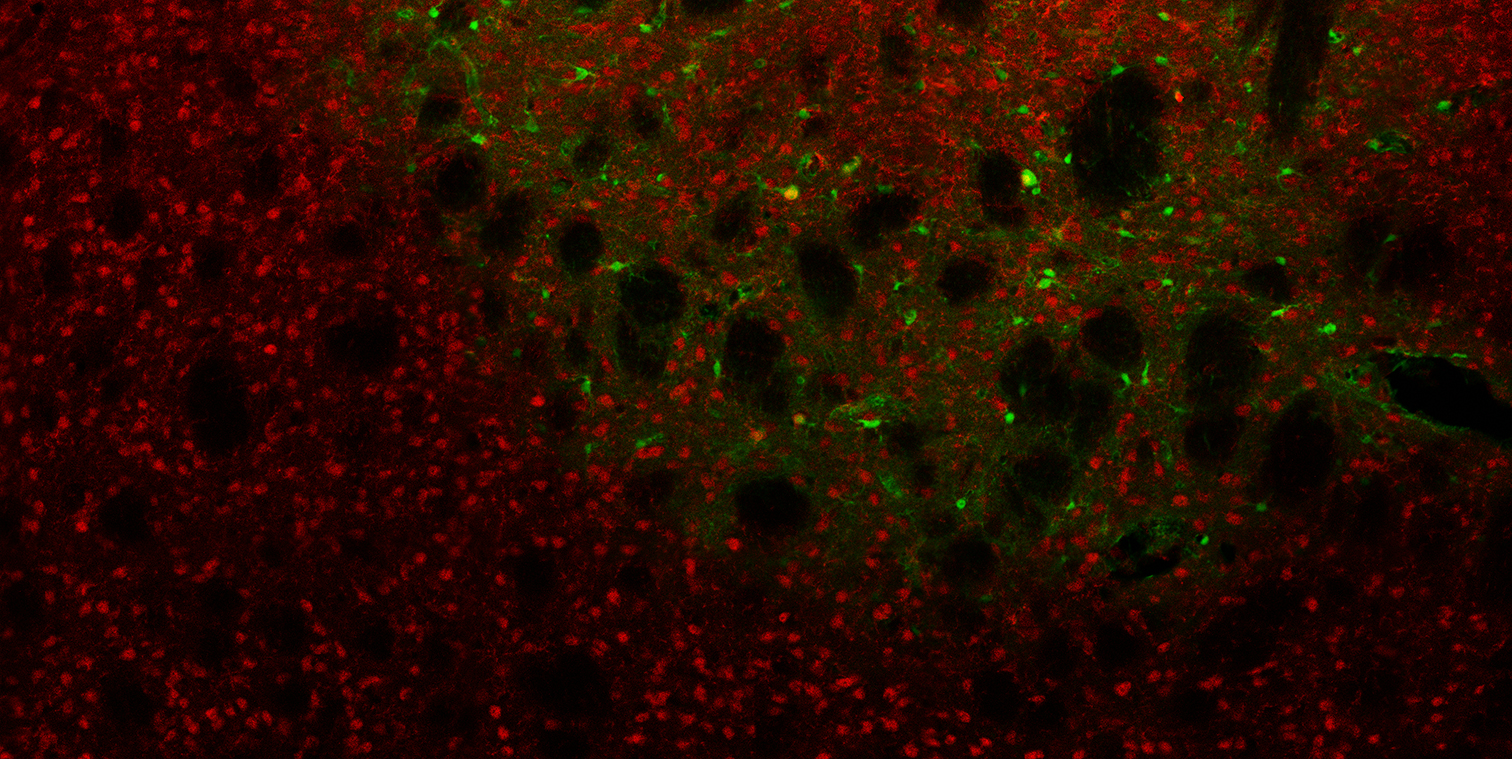

Supplement: Figure 2—source data 3. [file elife-75636-fig2-data3.zip › Fig2 source data 3 for Fig2 E/AAV-shPTB STR MZ1 #20 GFP+NeuN.jpg]

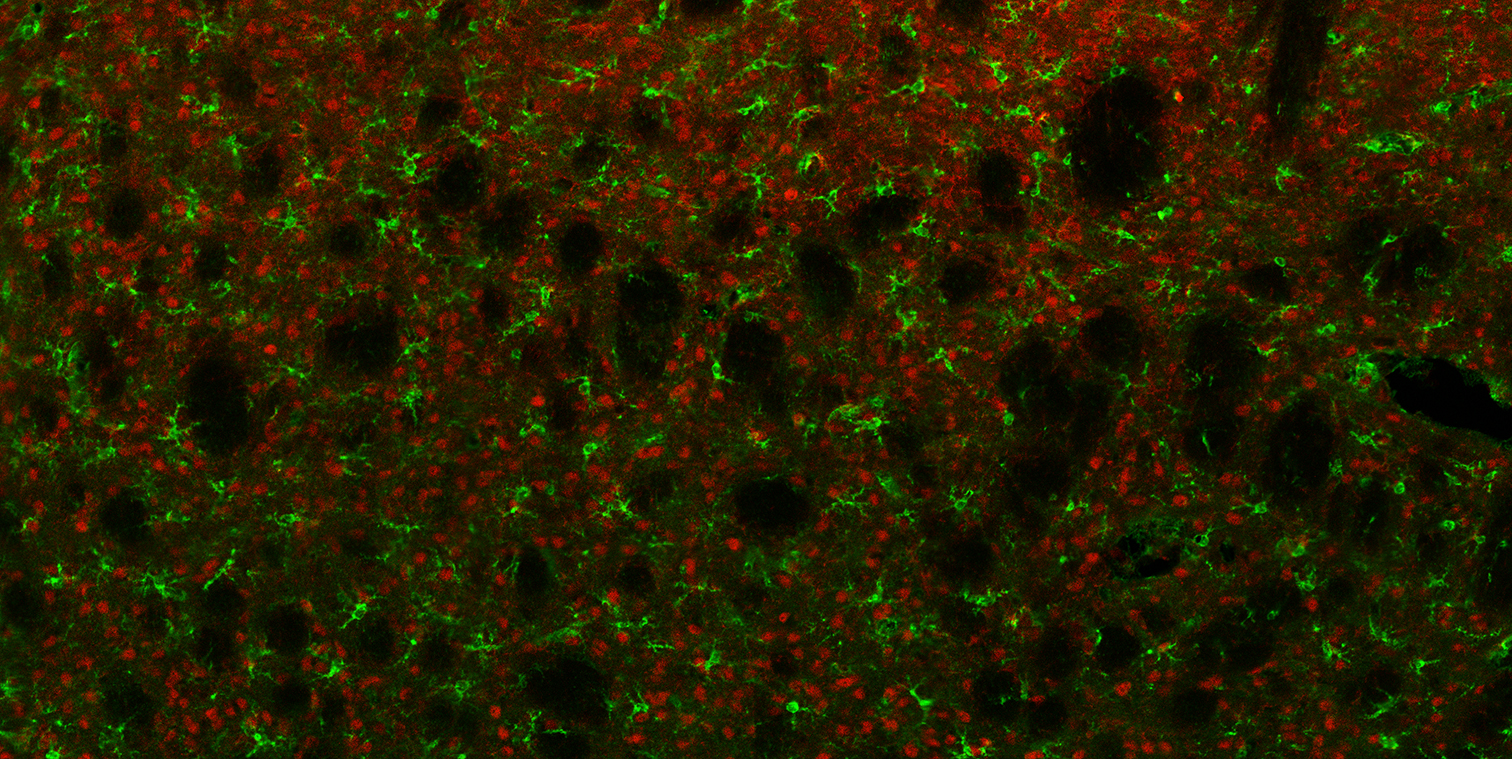

Supplement: Figure 2—source data 3. [file elife-75636-fig2-data3.zip › Fig2 source data 3 for Fig2 E/AAV-shPTB STR MZ1 #20 HA+NeuN.jpg]

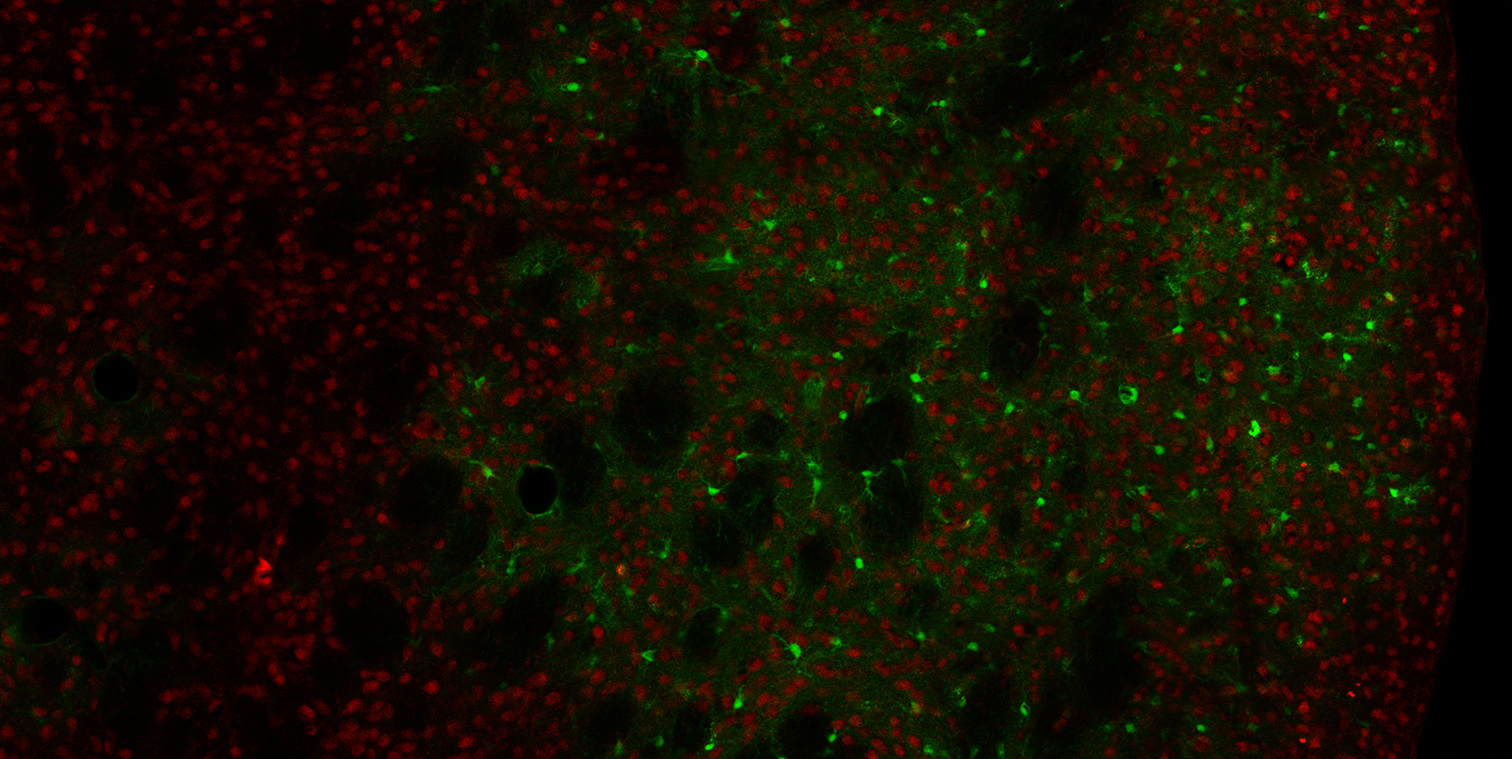

Supplement: Figure 2—source data 3. [file elife-75636-fig2-data3.zip › Fig2 source data 3 for Fig2 E/AAV-shPTB STR MZ2 #21 GFP+NeuN.jpg]

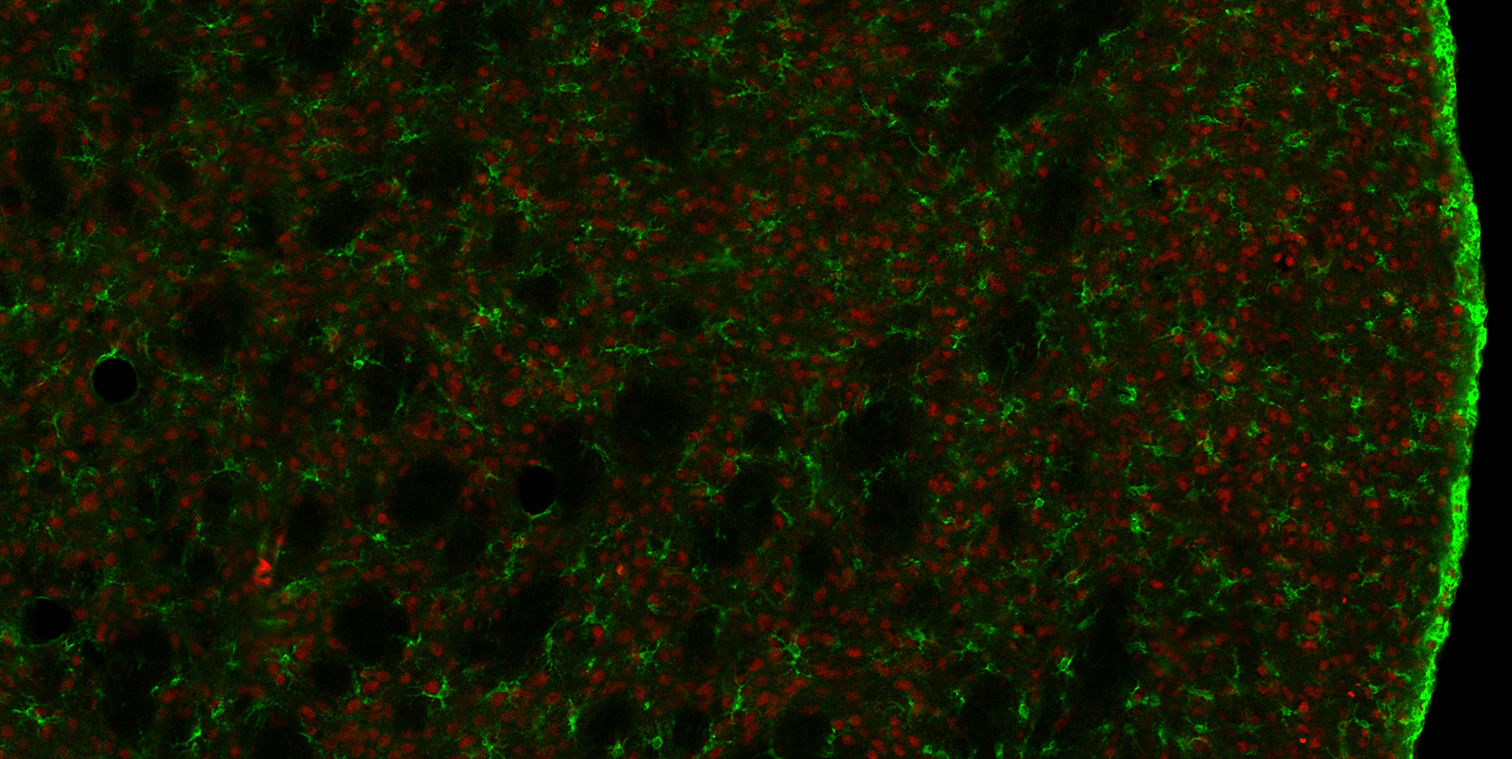

Supplement: Figure 2—source data 3. [file elife-75636-fig2-data3.zip › Fig2 source data 3 for Fig2 E/AAV-shPTB STR MZ2 #21 HA+NeuN.jpg]

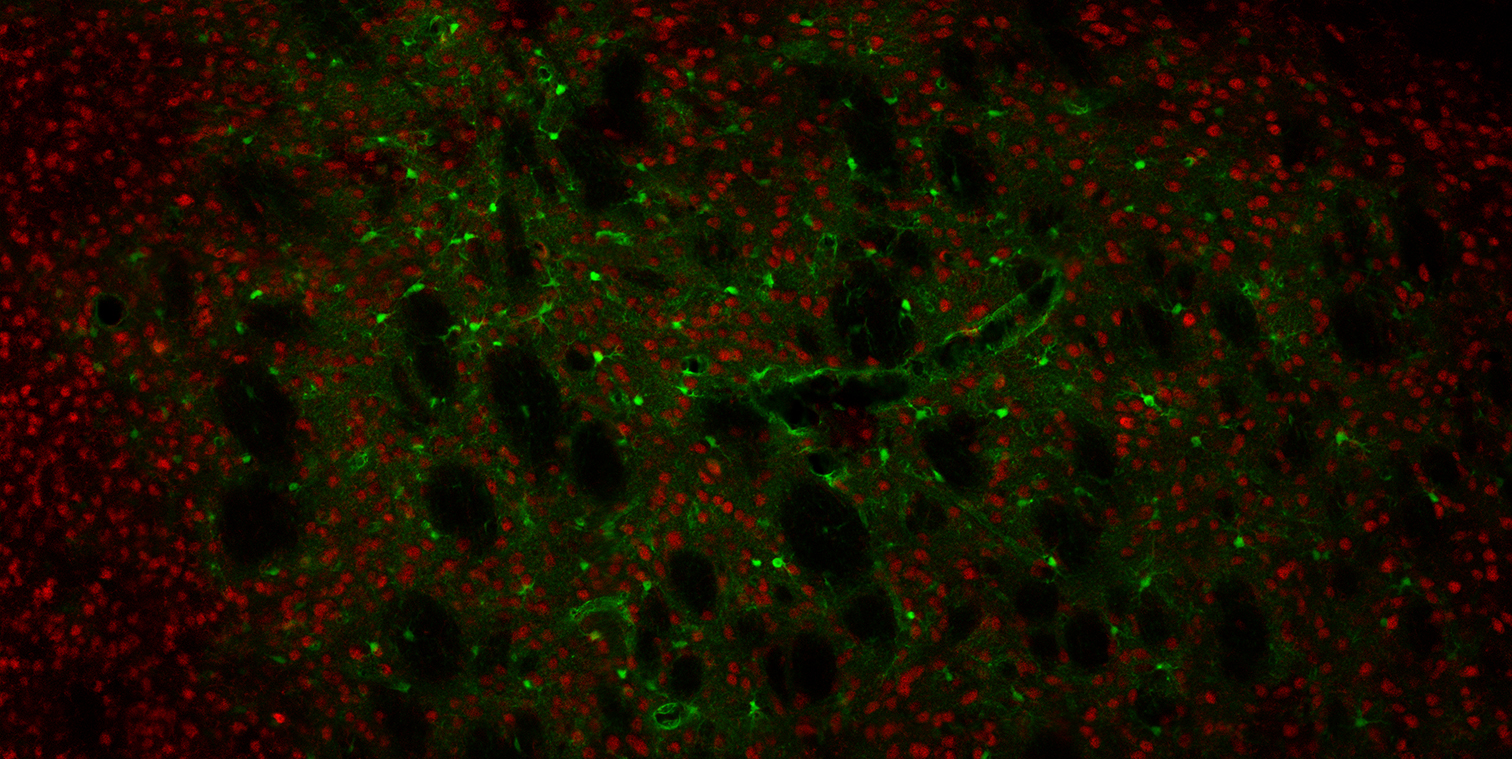

Supplement: Figure 2—source data 3. [file elife-75636-fig2-data3.zip › Fig2 source data 3 for Fig2 E/AAV-shPTB STR MZ3 #24 GFP+NeuN.jpg]

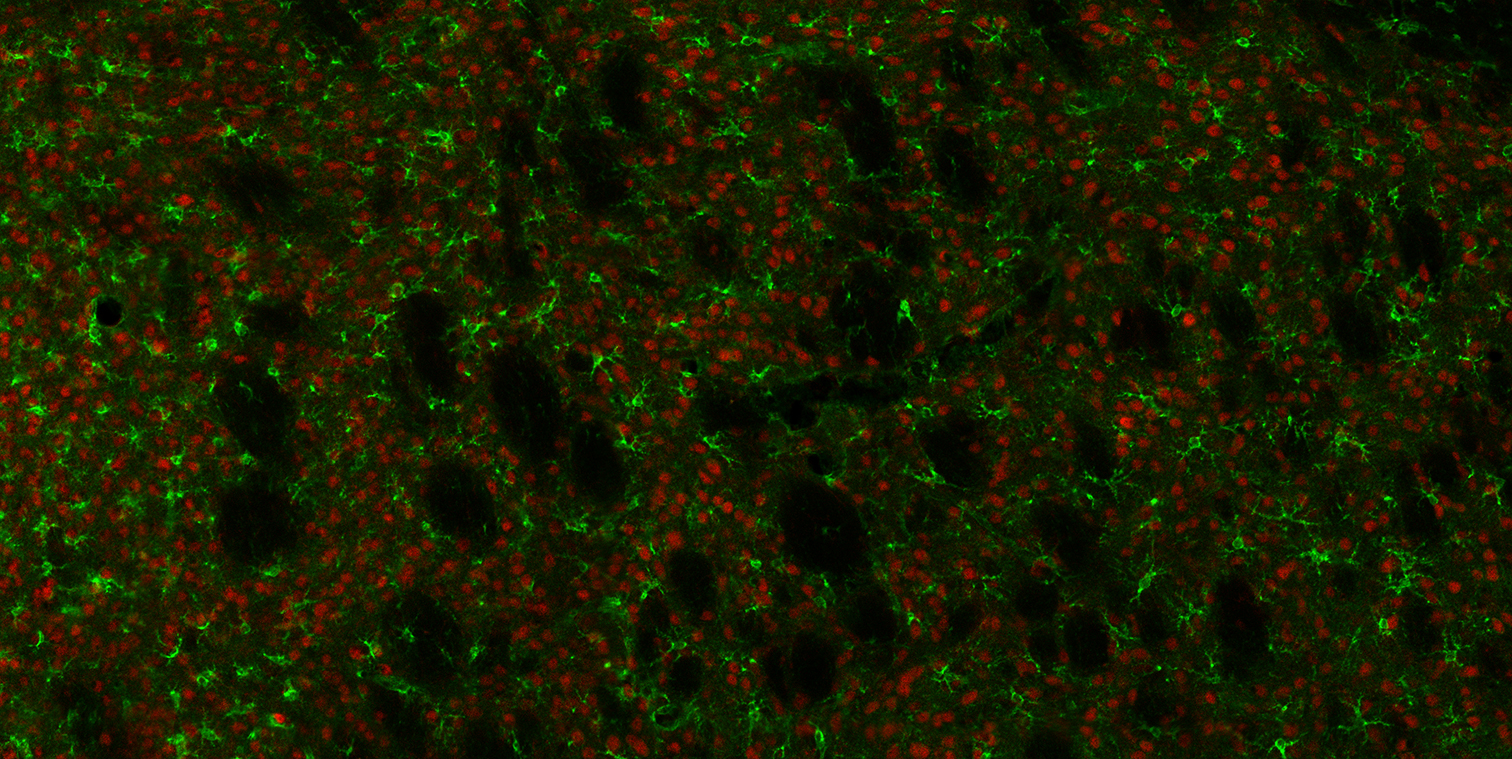

Supplement: Figure 2—source data 3. [file elife-75636-fig2-data3.zip › Fig2 source data 3 for Fig2 E/AAV-shPTB STR MZ3 #24 HA+NeuN.jpg]

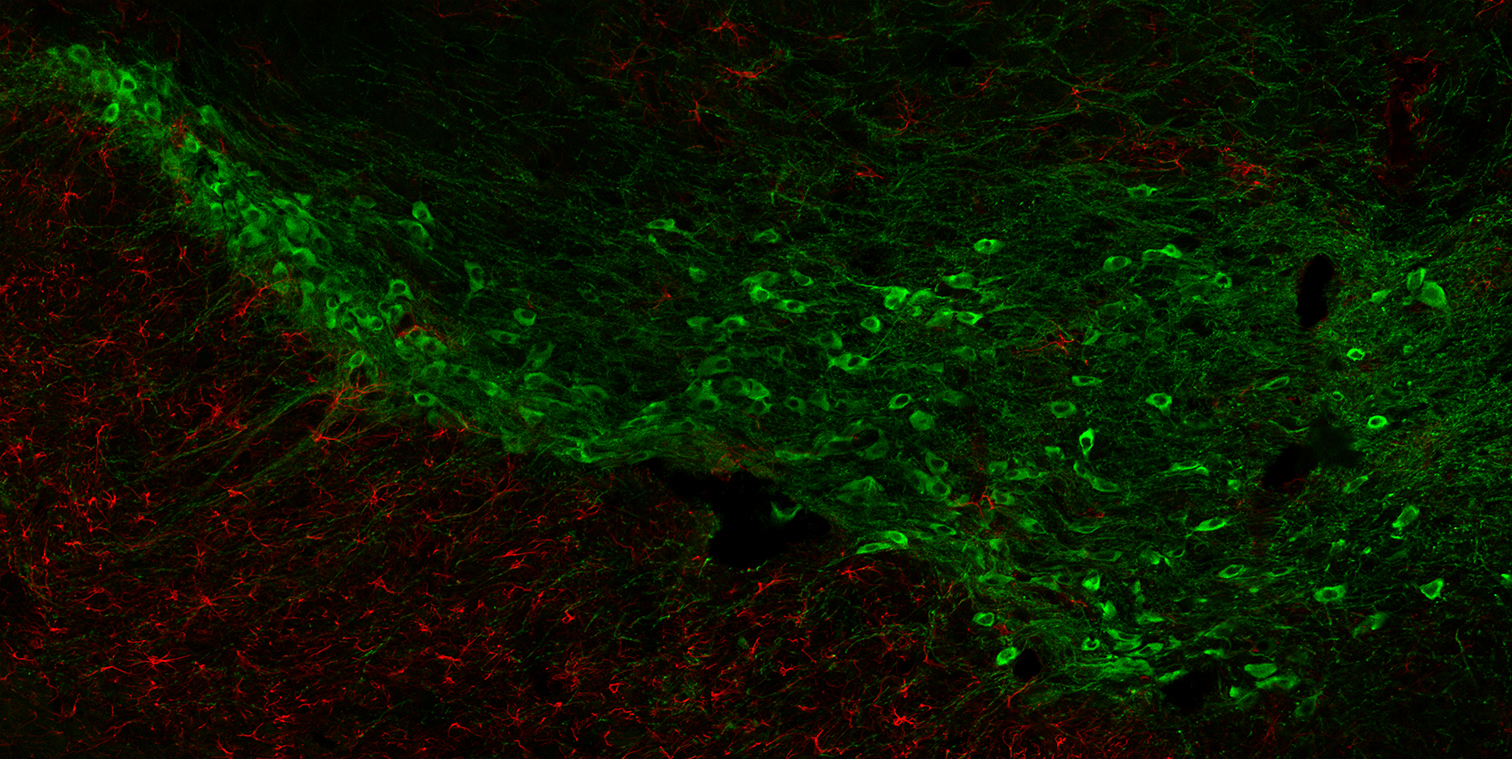

Supplement: Figure 3—source data 1. [file elife-75636-fig3-data1.zip › Fig3 source data 1 for Fig3 B/#56 6-OHDA SN CONTRALATERAL GFAP+TH.jpg]

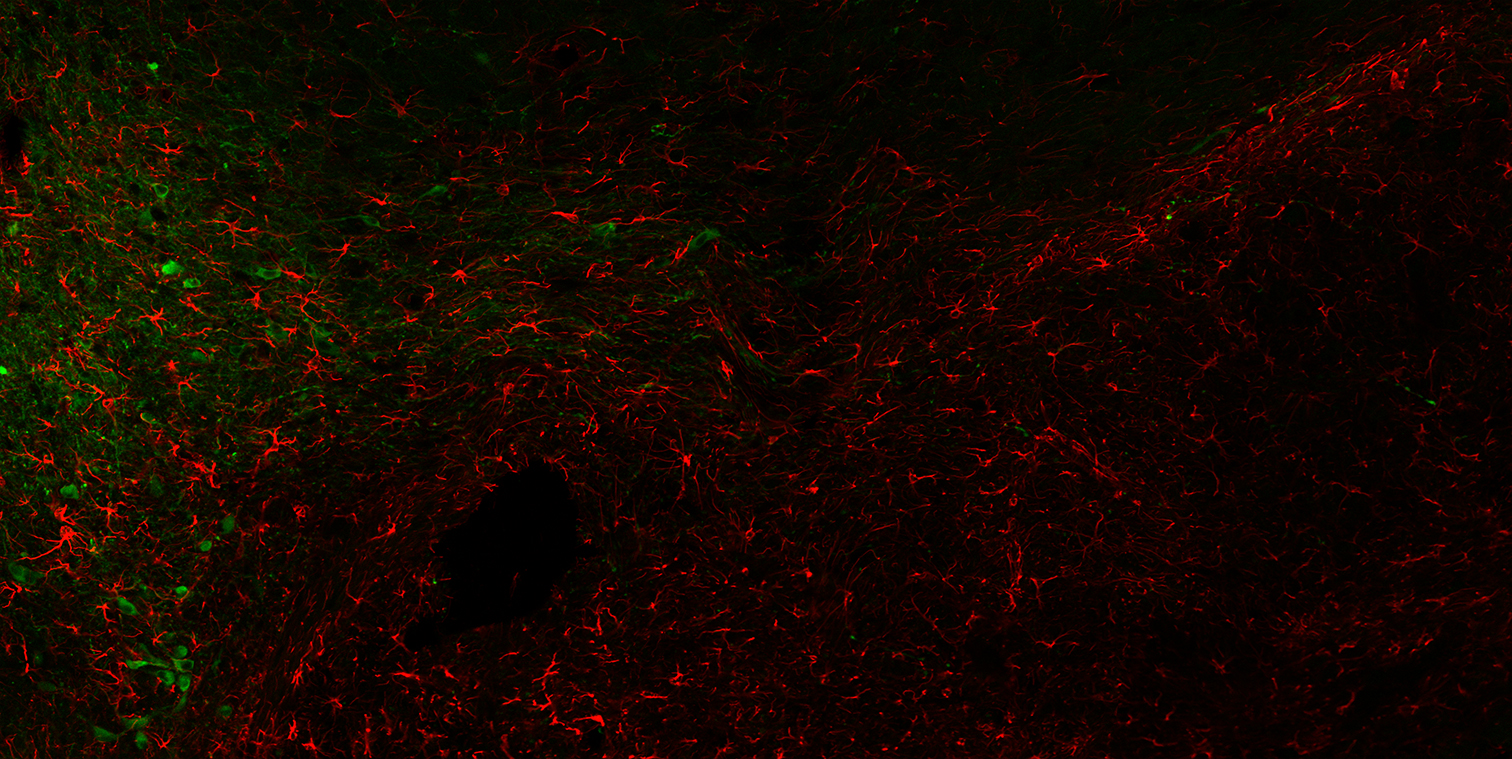

Supplement: Figure 3—source data 1. [file elife-75636-fig3-data1.zip › Fig3 source data 1 for Fig3 B/#56 6-OHDA SN IPSILATERAL GFAP+TH.jpg]

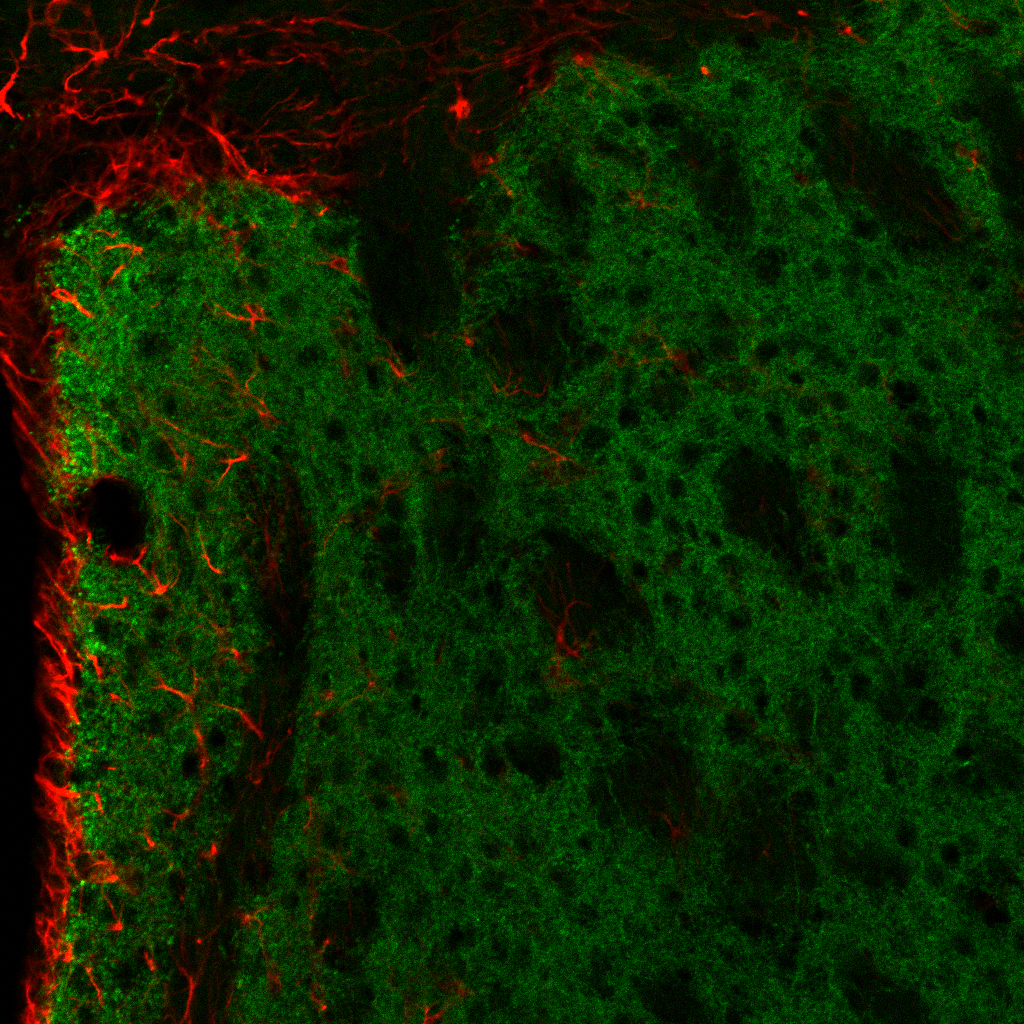

Supplement: Figure 3—source data 1. [file elife-75636-fig3-data1.zip › Fig3 source data 1 for Fig3 B/#56 6-OHDA STR CONTRALATERAL GFAP+TH.jpg]

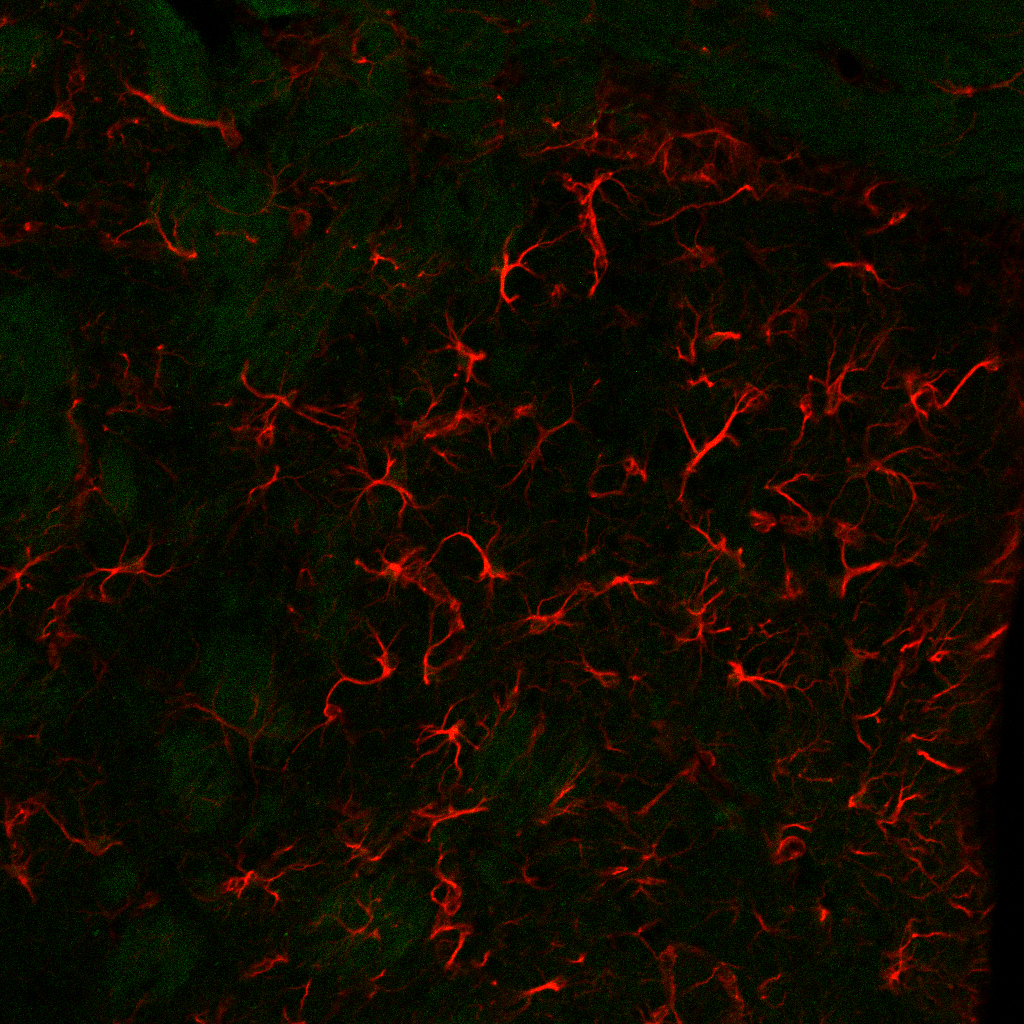

Supplement: Figure 3—source data 1. [file elife-75636-fig3-data1.zip › Fig3 source data 1 for Fig3 B/#56 6-OHDA STR IPSILATERAL GFAP+TH.jpg]

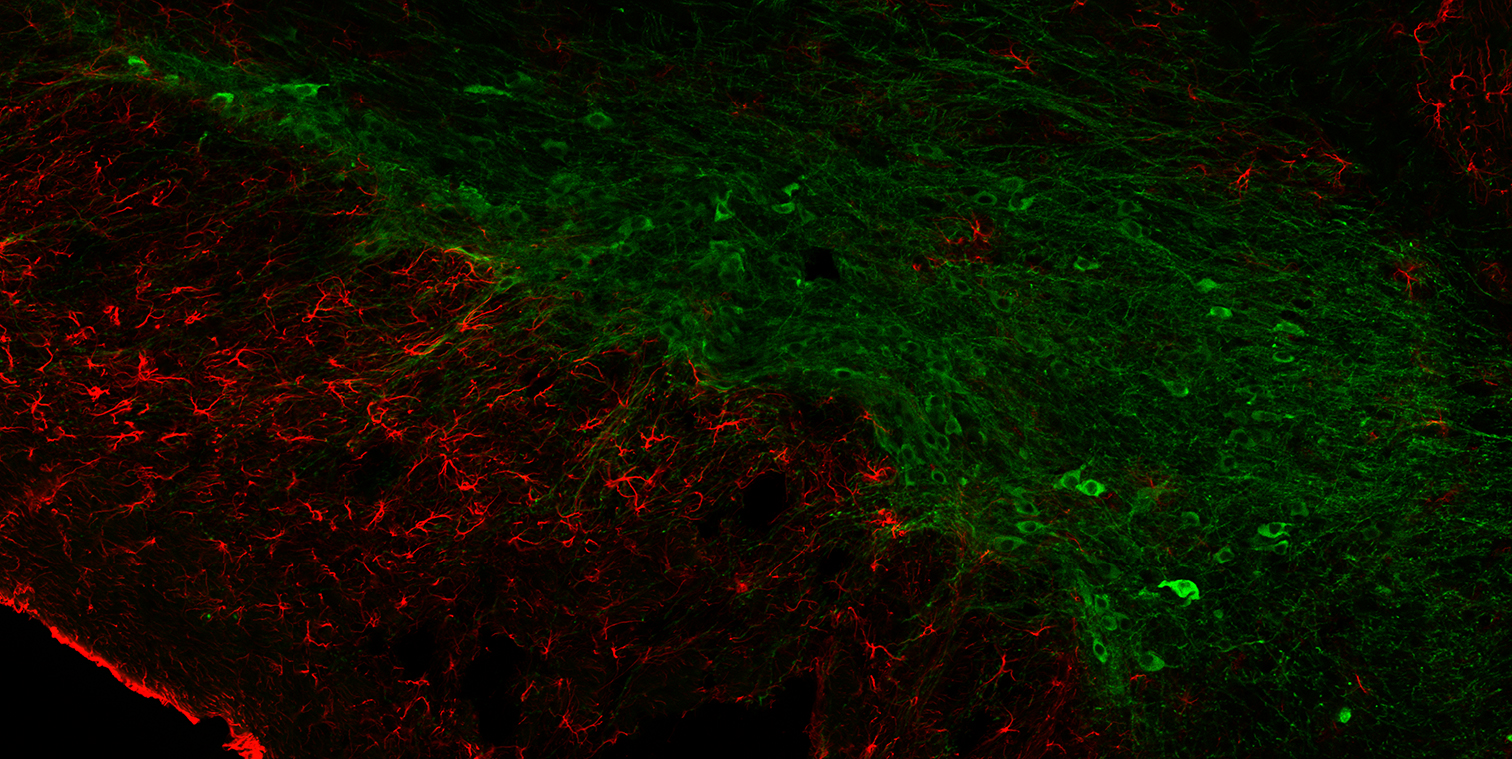

Supplement: Figure 3—source data 1. [file elife-75636-fig3-data1.zip › Fig3 source data 1 for Fig3 B/#57 6-OHDA SN CONTRALATERAL GFAP+TH.jpg]

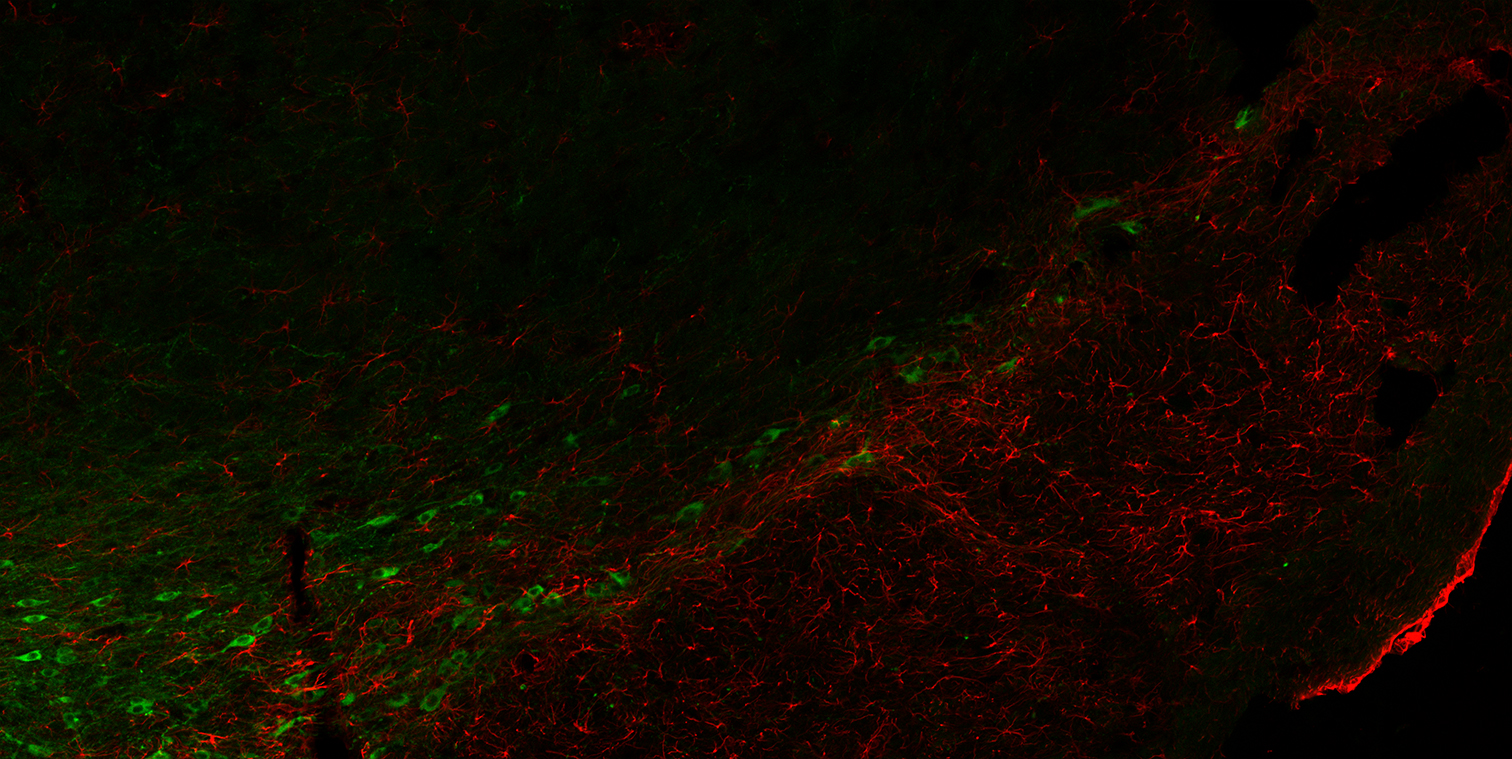

Supplement: Figure 3—source data 1. [file elife-75636-fig3-data1.zip › Fig3 source data 1 for Fig3 B/#57 6-OHDA SN IPSILATERAL GFAP+TH.jpg]

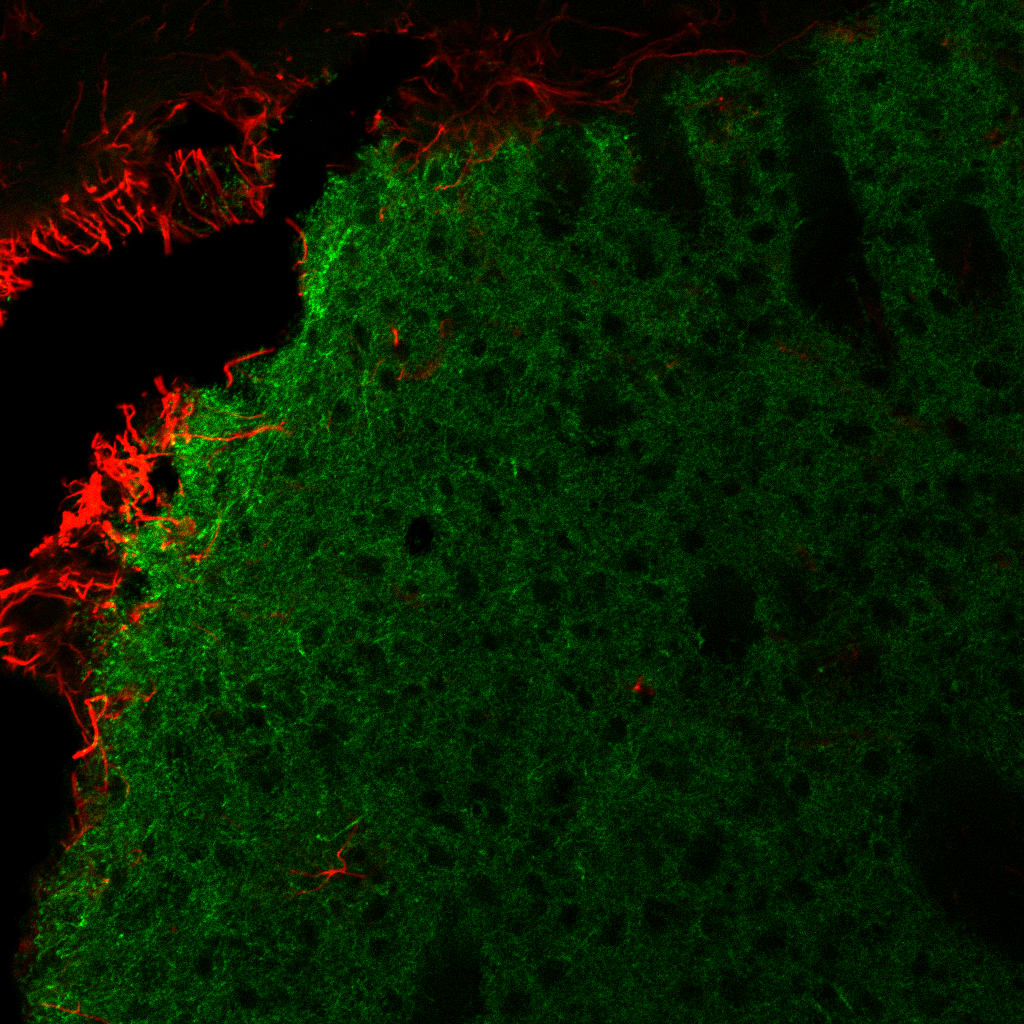

Supplement: Figure 3—source data 1. [file elife-75636-fig3-data1.zip › Fig3 source data 1 for Fig3 B/#57 6-OHDA STR CONTRALATERAL GFAP+TH.jpg]

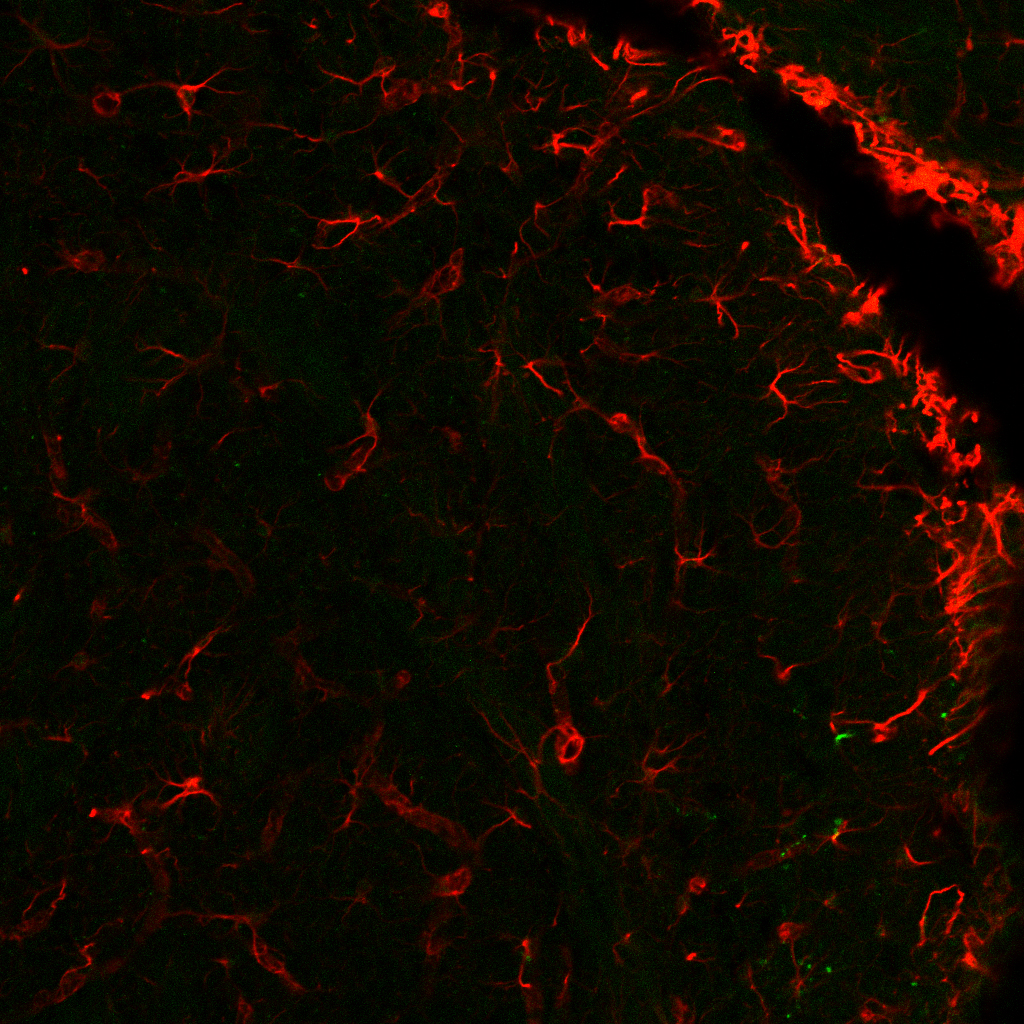

Supplement: Figure 3—source data 1. [file elife-75636-fig3-data1.zip › Fig3 source data 1 for Fig3 B/#57 6-OHDA STR IPSILATERAL GFAP+TH.jpg]

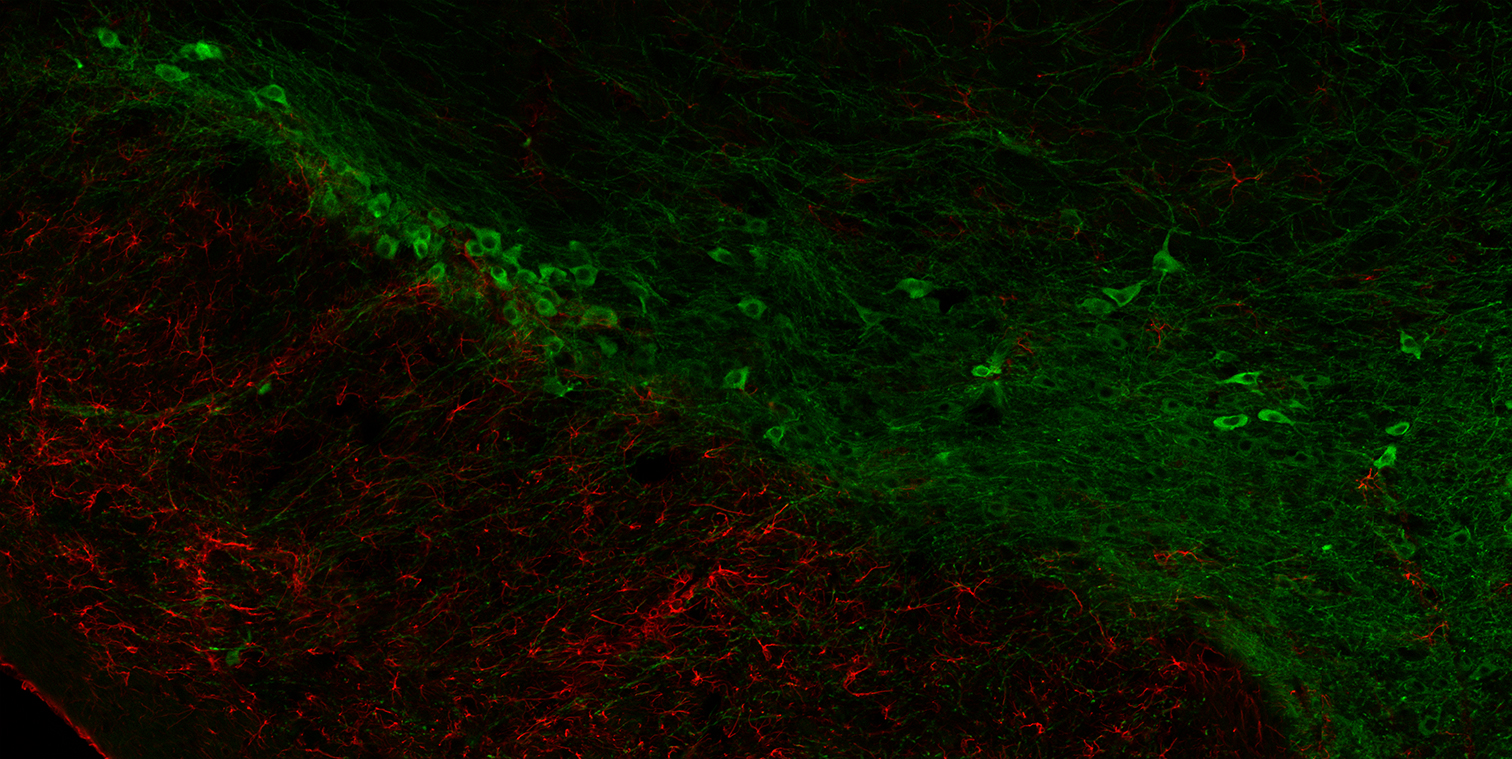

Supplement: Figure 3—source data 1. [file elife-75636-fig3-data1.zip › Fig3 source data 1 for Fig3 B/#60 6-OHDA SN CONTRALATERAL GFAP+TH.jpg]

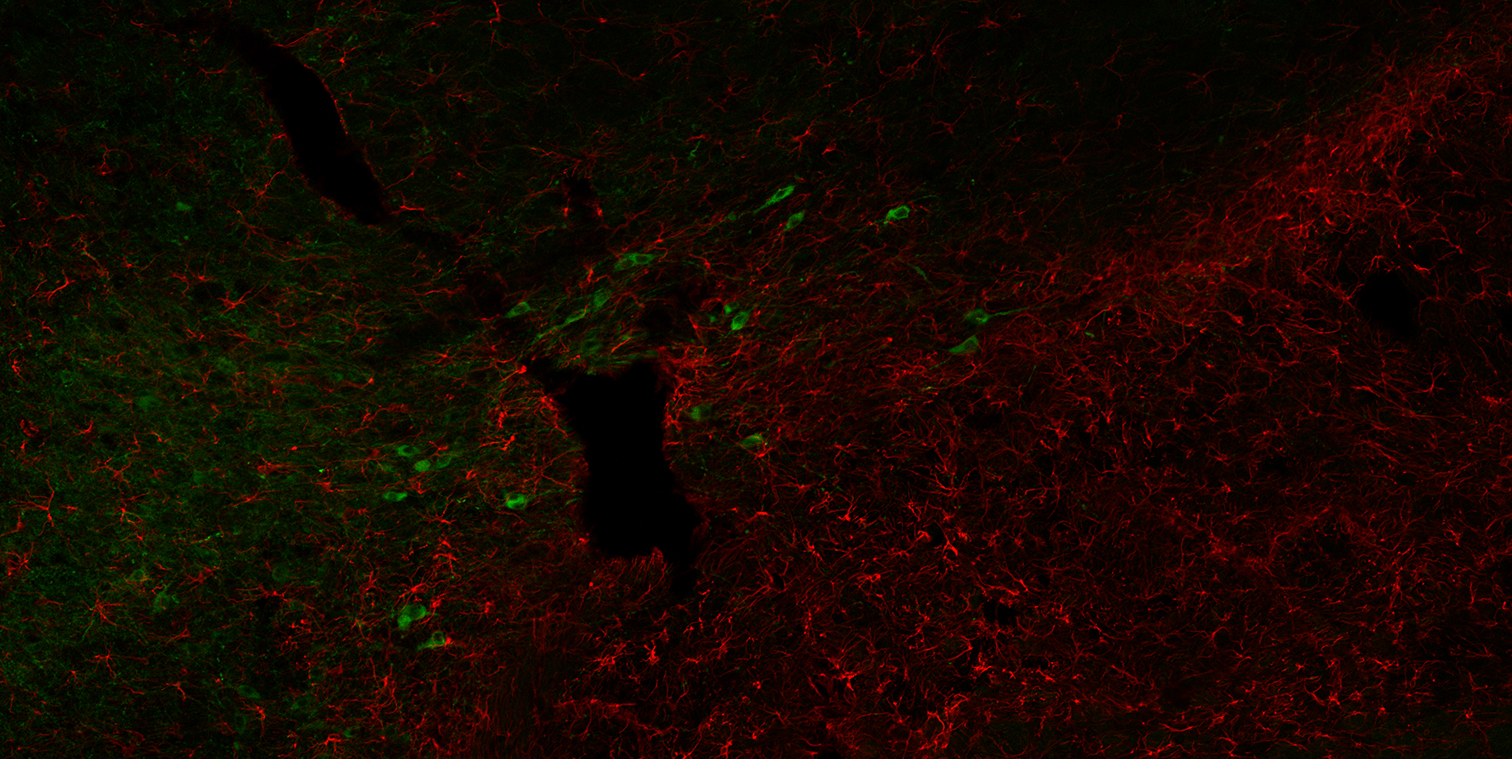

Supplement: Figure 3—source data 1. [file elife-75636-fig3-data1.zip › Fig3 source data 1 for Fig3 B/#60 6-OHDA SN IPSILATERAL GFAP+TH.jpg]

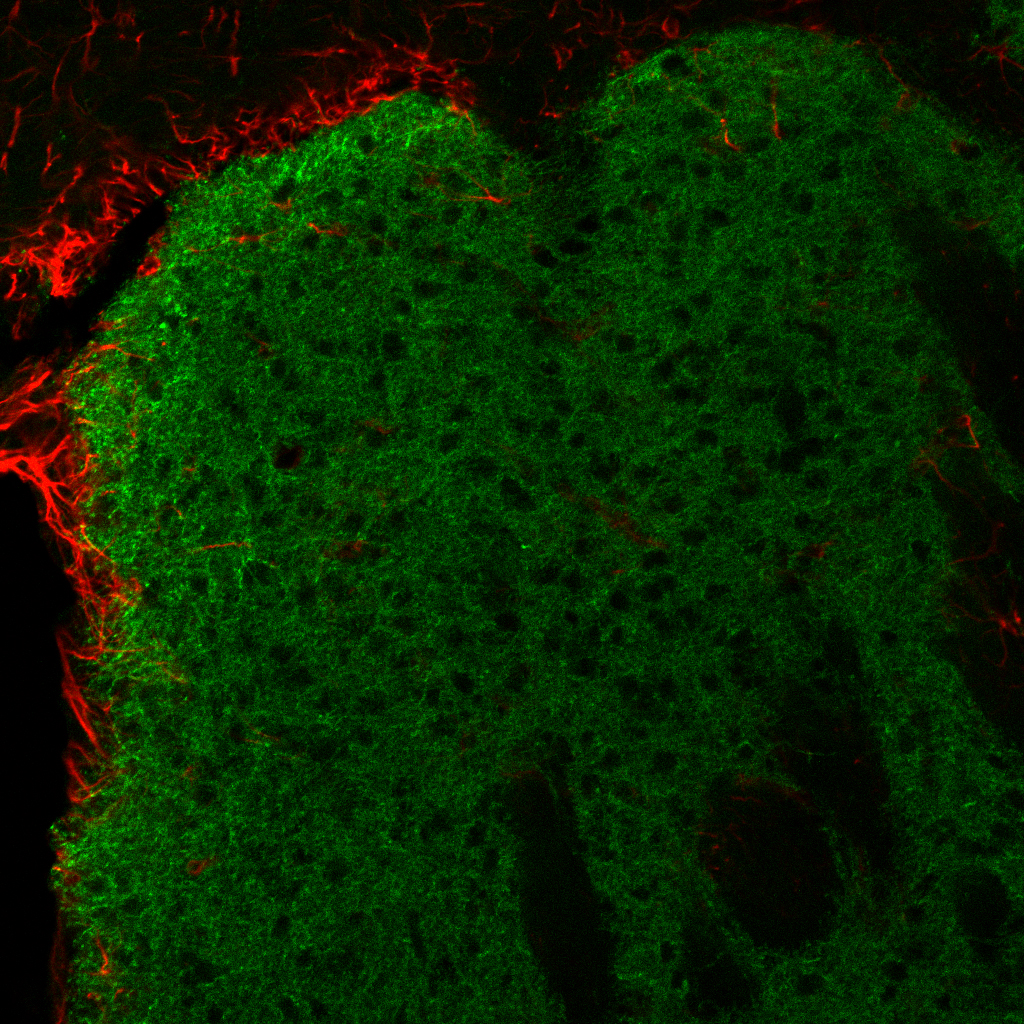

Supplement: Figure 3—source data 1. [file elife-75636-fig3-data1.zip › Fig3 source data 1 for Fig3 B/#60 6-OHDA STR CONTRALATERAL GFAP+TH.jpg]

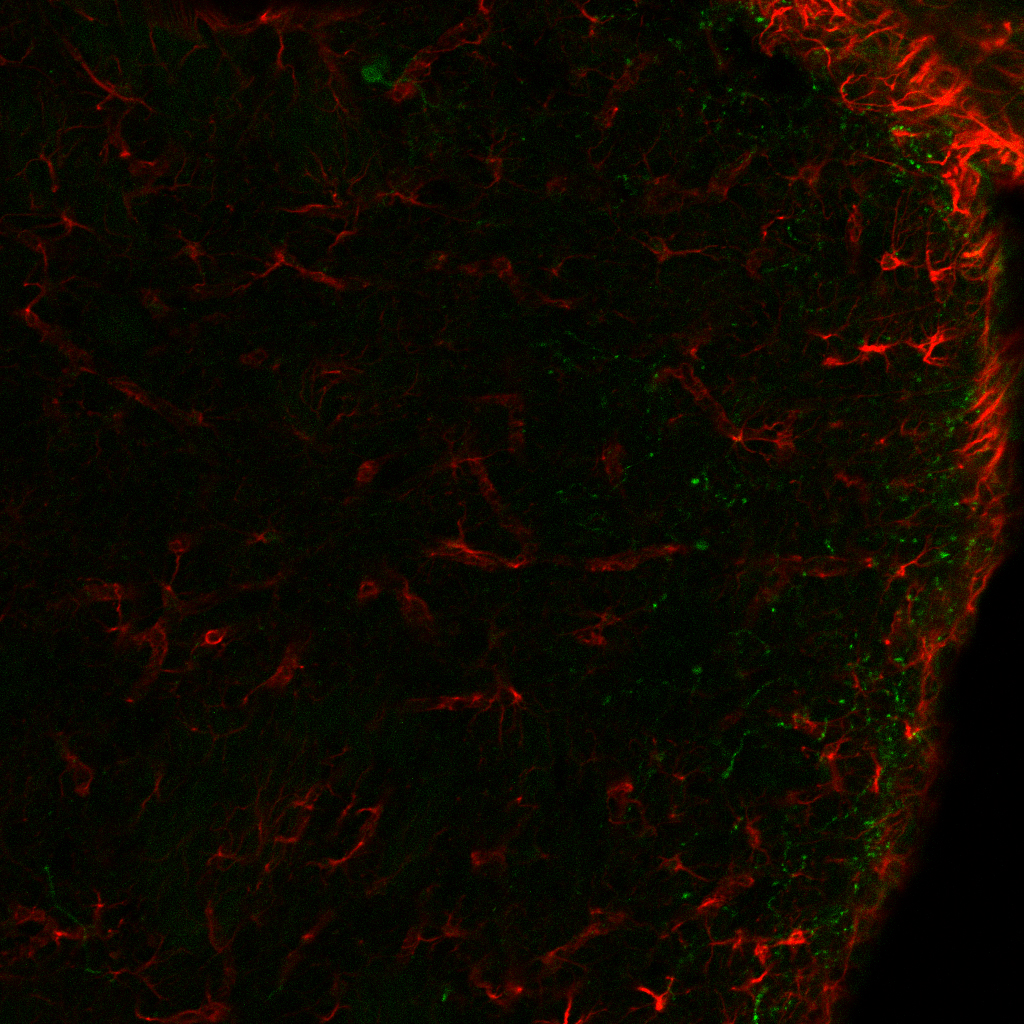

Supplement: Figure 3—source data 1. [file elife-75636-fig3-data1.zip › Fig3 source data 1 for Fig3 B/#60 6-OHDA STR IPSILATERAL GFAP+TH.jpg]

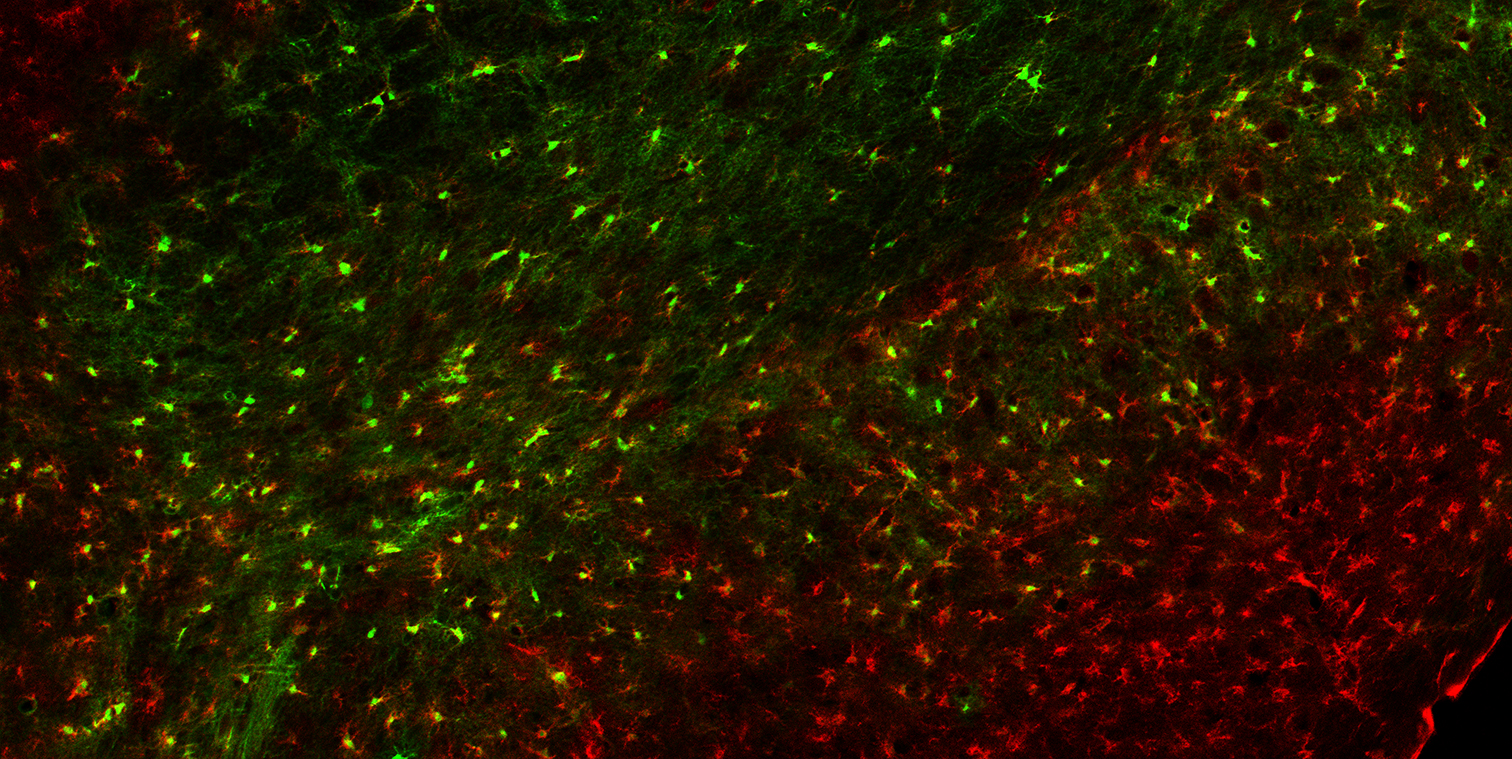

Supplement: Figure 3—source data 2. [file elife-75636-fig3-data2.zip › Fig3 source data 2 for Fig3 C/AAV-shptb SN #13 GFP+HA-1.jpg]

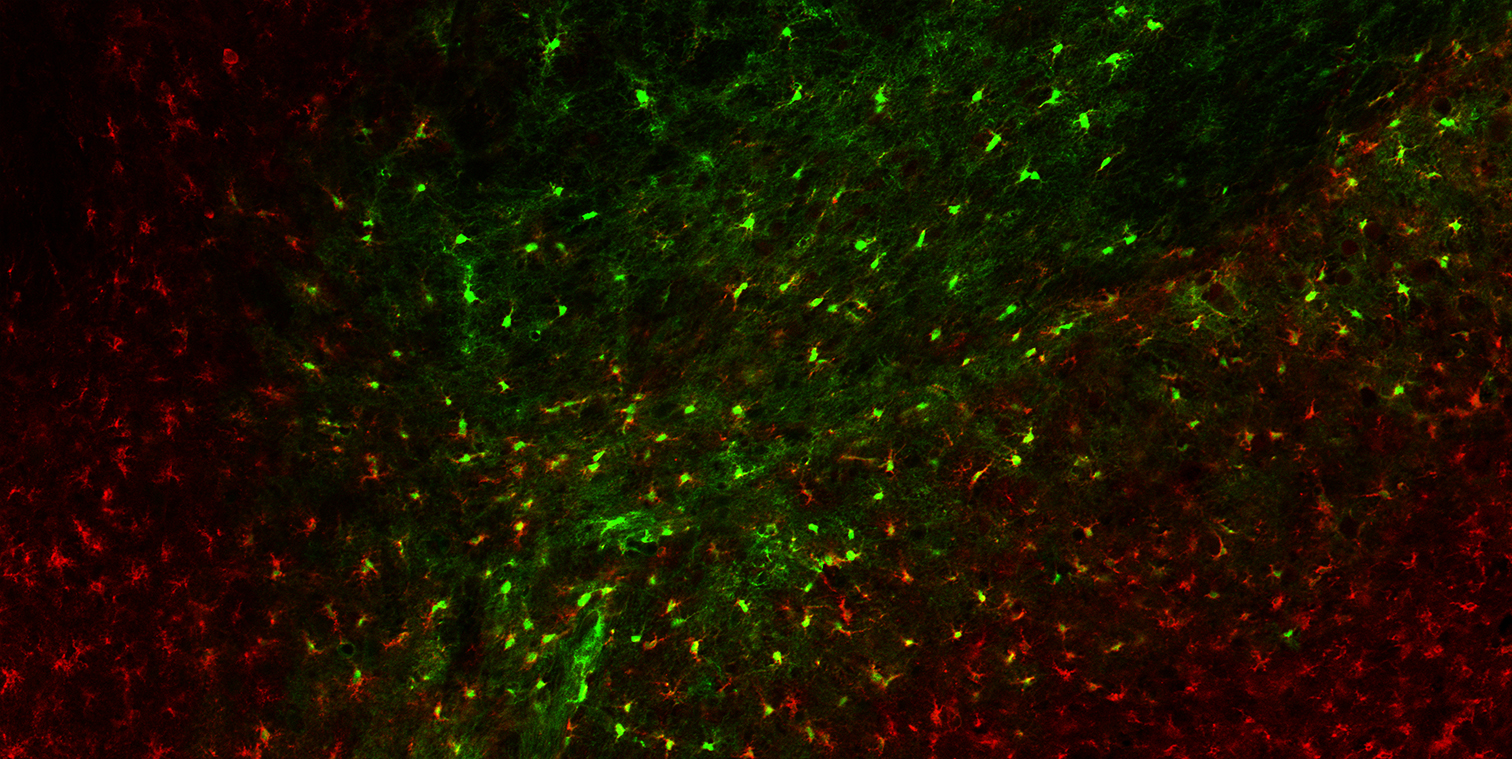

Supplement: Figure 3—source data 2. [file elife-75636-fig3-data2.zip › Fig3 source data 2 for Fig3 C/AAV-shptb SN #13 GFP+HA-2.jpg]
