# Supplementary material for: Repressing PTBP1 fails to convert reactive astrocytes to dopaminergic neurons in a 6-hydroxydopamine mouse model of Parkinson’s disease
Source: eLife. 2022 May 10;11:e75636. doi: 10.7554/eLife.75636 (PMC9208759; doi:10.7554/eLife.75636)
Supplement: Figure 1—figure supplement 1—source data 1. [file elife-75636-fig1-figsupp1-data1.zip › Fig1 source data 4 for Fig1 supplement 1/description of source data for Fig1 supplement 1.docx]

Brain slices co-stained with GFP (green) and AldoC (purple), NeuN (red), NG2 (purple), or Iba-1 (red) 7 days after AAV-sh*Ptbp1* or AAV-shscramble delivery in the substantia nigra (SN) or striatum (STR).
