# Supplementary material for: Repressing PTBP1 fails to convert reactive astrocytes to dopaminergic neurons in a 6-hydroxydopamine mouse model of Parkinson’s disease
Source: eLife. 2022 May 10;11:e75636. doi: 10.7554/eLife.75636 (PMC9208759; doi:10.7554/eLife.75636)
Supplement: Figure 2—source data 1. [file elife-75636-fig2-data1.zip › Fig2 source data 1 for Fig2 B/description of source data for Fig2B.docx]

Indicated brain regions of Aldh1l1-CreERT2:Rpl22HA/HA mice co-stained HA (red) with pan-astrocyte marker AldoC (green) and TH (purple) 2 weeks after TAM administration.
