# Supplementary material for: Repressing PTBP1 fails to convert reactive astrocytes to dopaminergic neurons in a 6-hydroxydopamine mouse model of Parkinson’s disease
Source: eLife. 2022 May 10;11:e75636. doi: 10.7554/eLife.75636 (PMC9208759; doi:10.7554/eLife.75636)
Supplement: Figure 2—source data 3. [file elife-75636-fig2-data3.zip › Fig2 source data 3 for Fig2 E/description of source data for Fig2E.docx]

Brain slices co-stained GFP (green), HA (red) with NeuN (purple) in the striatum 3 months after AAV-sh*Ptbp1* delivery.
