# Supplementary material for: Repressing PTBP1 fails to convert reactive astrocytes to dopaminergic neurons in a 6-hydroxydopamine mouse model of Parkinson’s disease
Source: eLife. 2022 May 10;11:e75636. doi: 10.7554/eLife.75636 (PMC9208759; doi:10.7554/eLife.75636)
Supplement: Figure 3—source data 1. [file elife-75636-fig3-data1.zip › Fig3 source data 1 for Fig3 B/description of source data for Fig3B.docx]

Brain slices of the substantia nigra or striatum after 6-OHDA lesion, co-stained with TH (green) and GFAP (red).
