# Supplementary material for: Repressing PTBP1 fails to convert reactive astrocytes to dopaminergic neurons in a 6-hydroxydopamine mouse model of Parkinson’s disease
Source: eLife. 2022 May 10;11:e75636. doi: 10.7554/eLife.75636 (PMC9208759; doi:10.7554/eLife.75636)
Supplement: Figure 3—source data 2. [file elife-75636-fig3-data2.zip › Fig3 source data 2 for Fig3 C/description of source data for Fig3C.docx]

Brain slices of *Aldh1l1-CreER^T2^*:*Rpl22^HA/HA^* mice subjected to 6-OHDA lesion and AAV-sh*Ptbp1* or AAV-shscramble injection in substantia or striatum, co-stained with GFP (green) and HA (red), or NeuN (green) and HA (red), or TH(green) and HA (red).
