# Supplementary figures and images for: Repressing PTBP1 fails to convert reactive astrocytes to dopaminergic neurons in a 6-hydroxydopamine mouse model of Parkinson’s disease (part 3 of 4)
Source: eLife. 2022 May 10;11:e75636. doi: 10.7554/eLife.75636 (PMC9208759; doi:10.7554/eLife.75636)

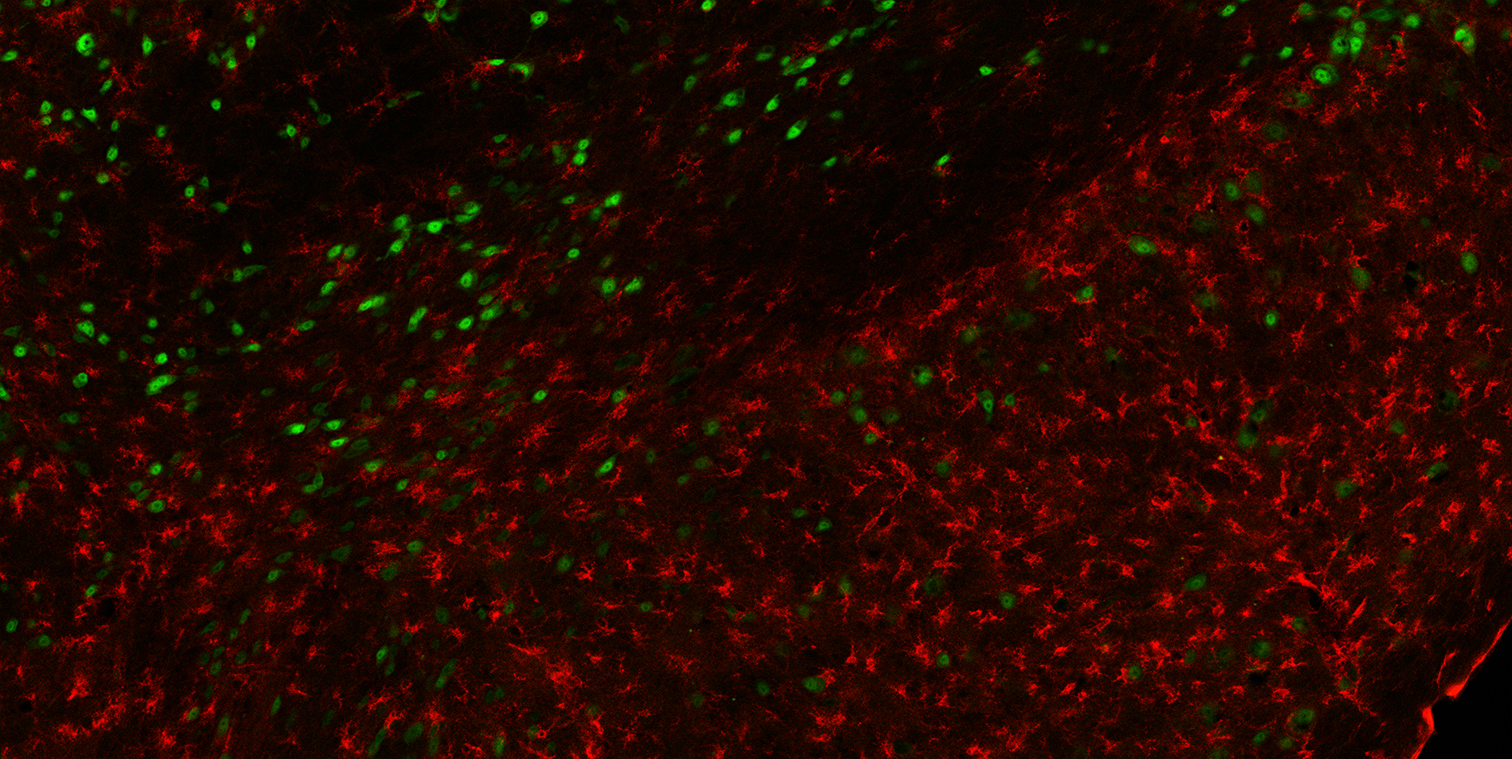

Supplement: Figure 3—source data 2. [file elife-75636-fig3-data2.zip › Fig3 source data 2 for Fig3 C/AAV-shptb SN #13 NeuN+HA.jpg]

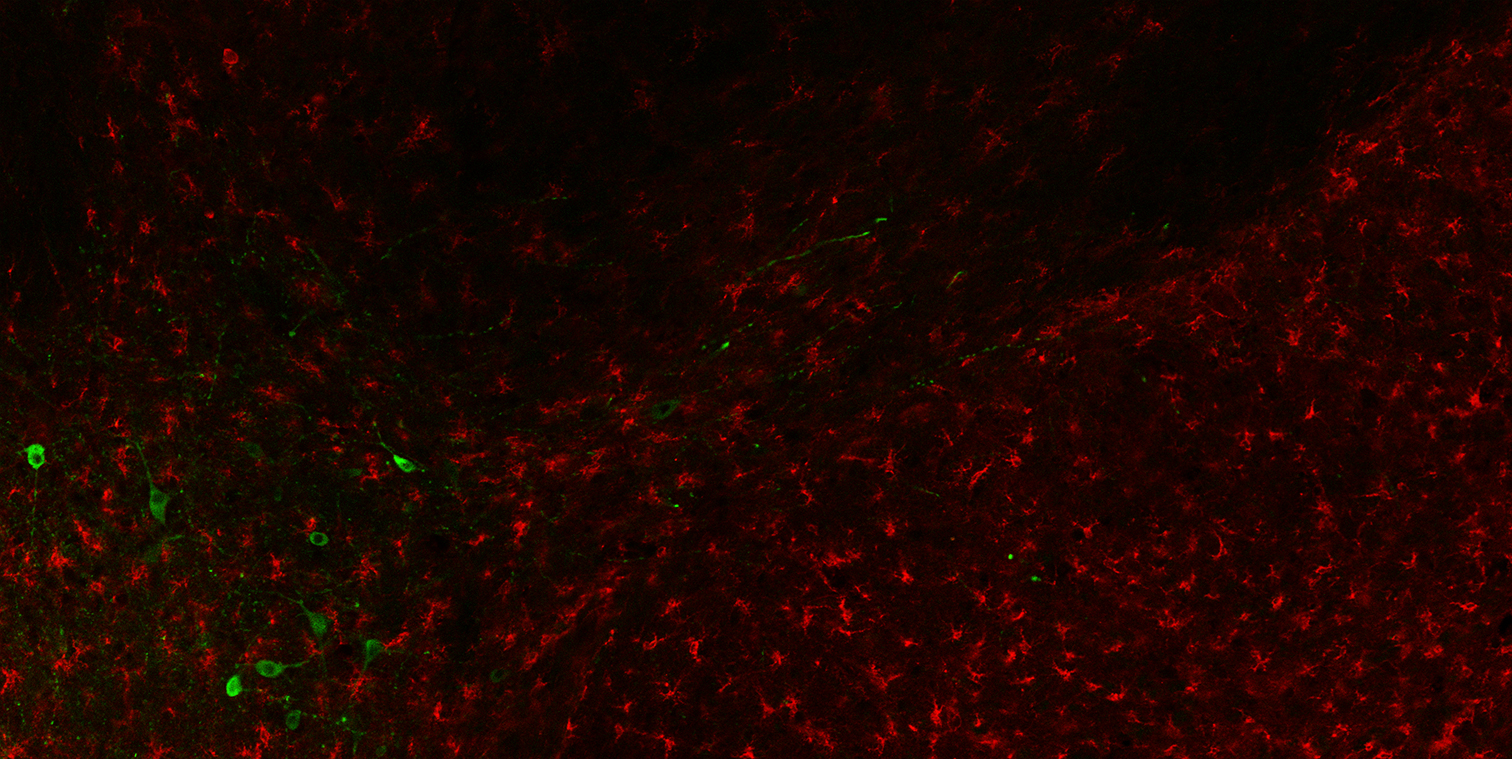

Supplement: Figure 3—source data 2. [file elife-75636-fig3-data2.zip › Fig3 source data 2 for Fig3 C/AAV-shptb SN #13 TH+HA.jpg]

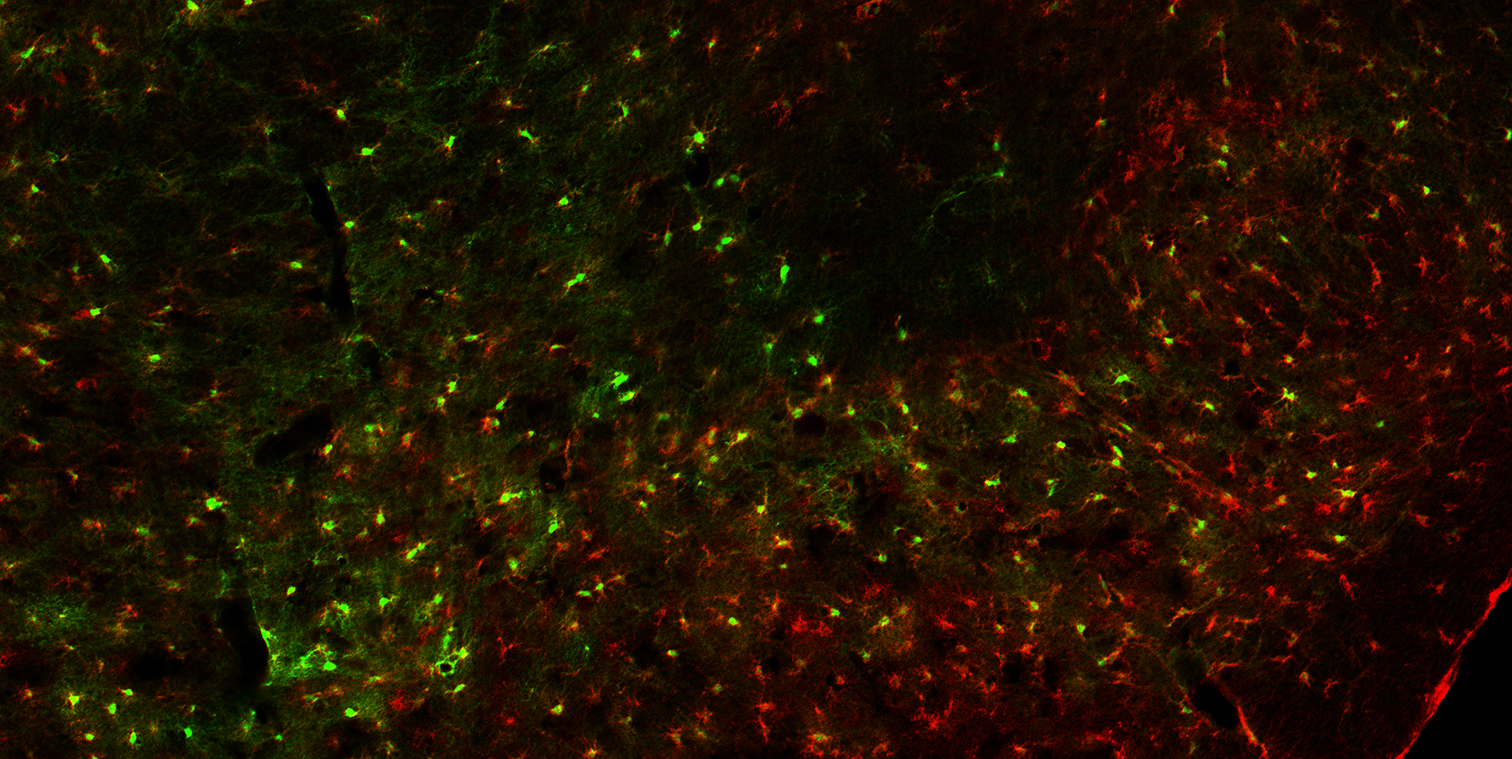

Supplement: Figure 3—source data 2. [file elife-75636-fig3-data2.zip › Fig3 source data 2 for Fig3 C/AAV-shptb SN #19 GFP+HA-1.jpg]

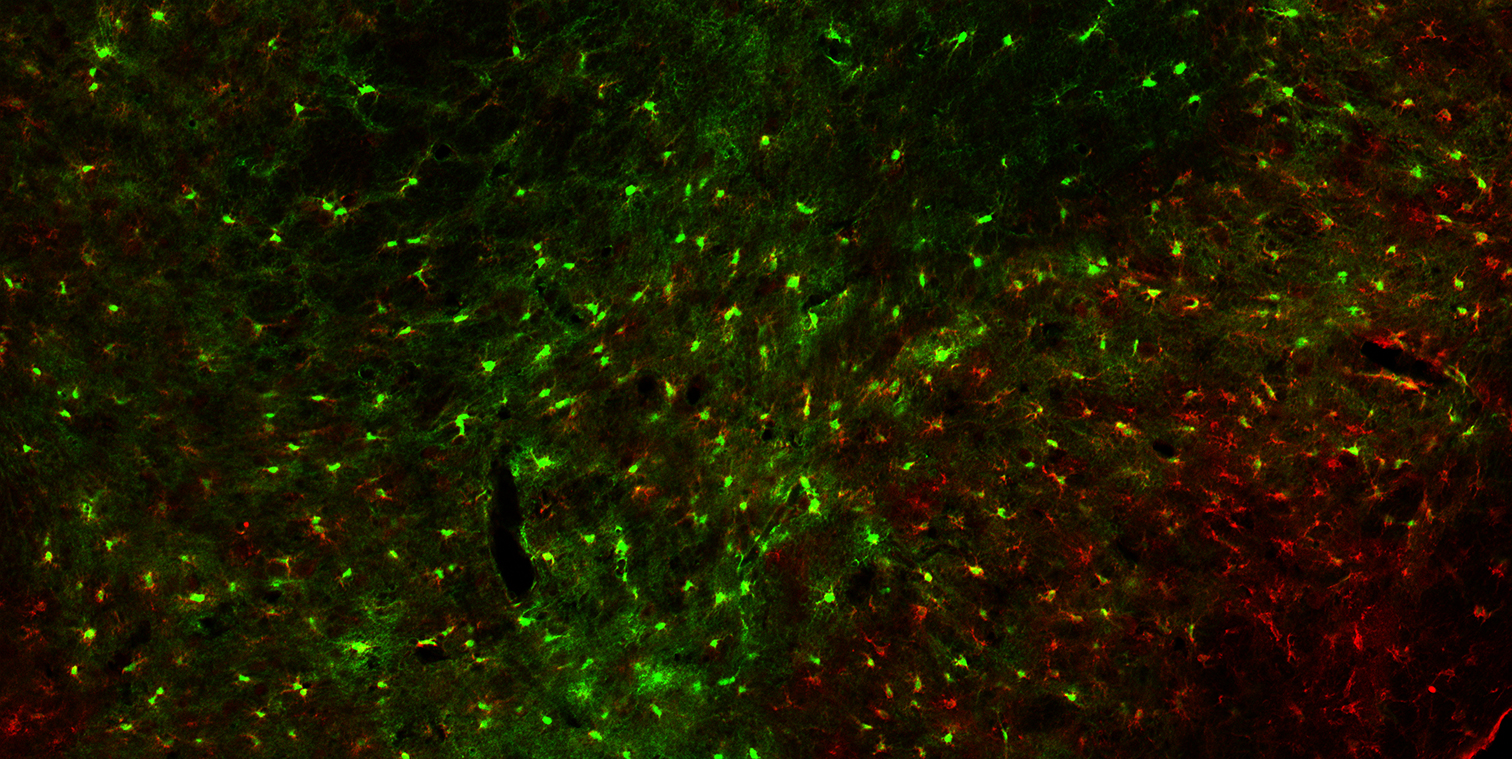

Supplement: Figure 3—source data 2. [file elife-75636-fig3-data2.zip › Fig3 source data 2 for Fig3 C/AAV-shptb SN #19 GFP+HA-2.jpg]

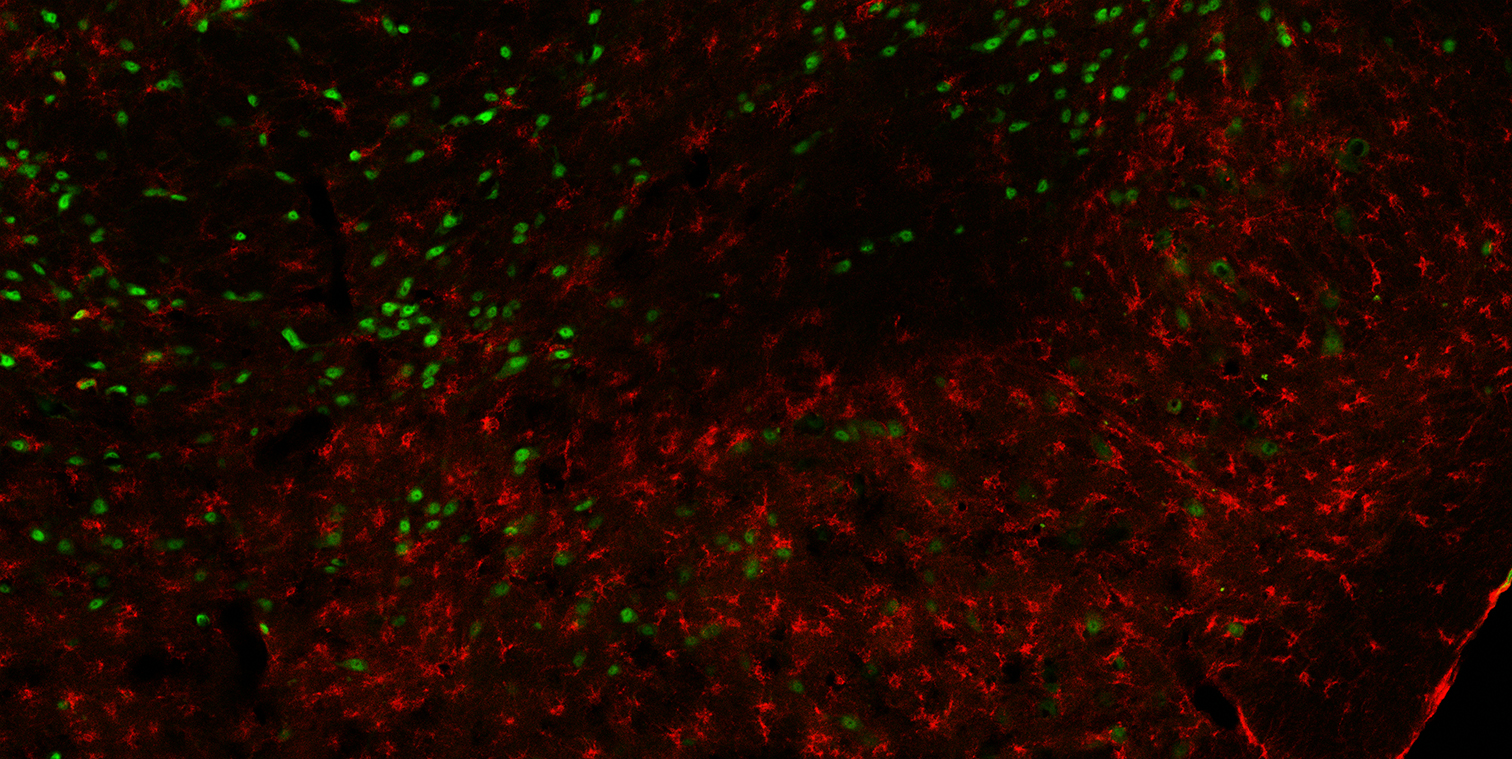

Supplement: Figure 3—source data 2. [file elife-75636-fig3-data2.zip › Fig3 source data 2 for Fig3 C/AAV-shptb SN #19 NeuN+HA.jpg]

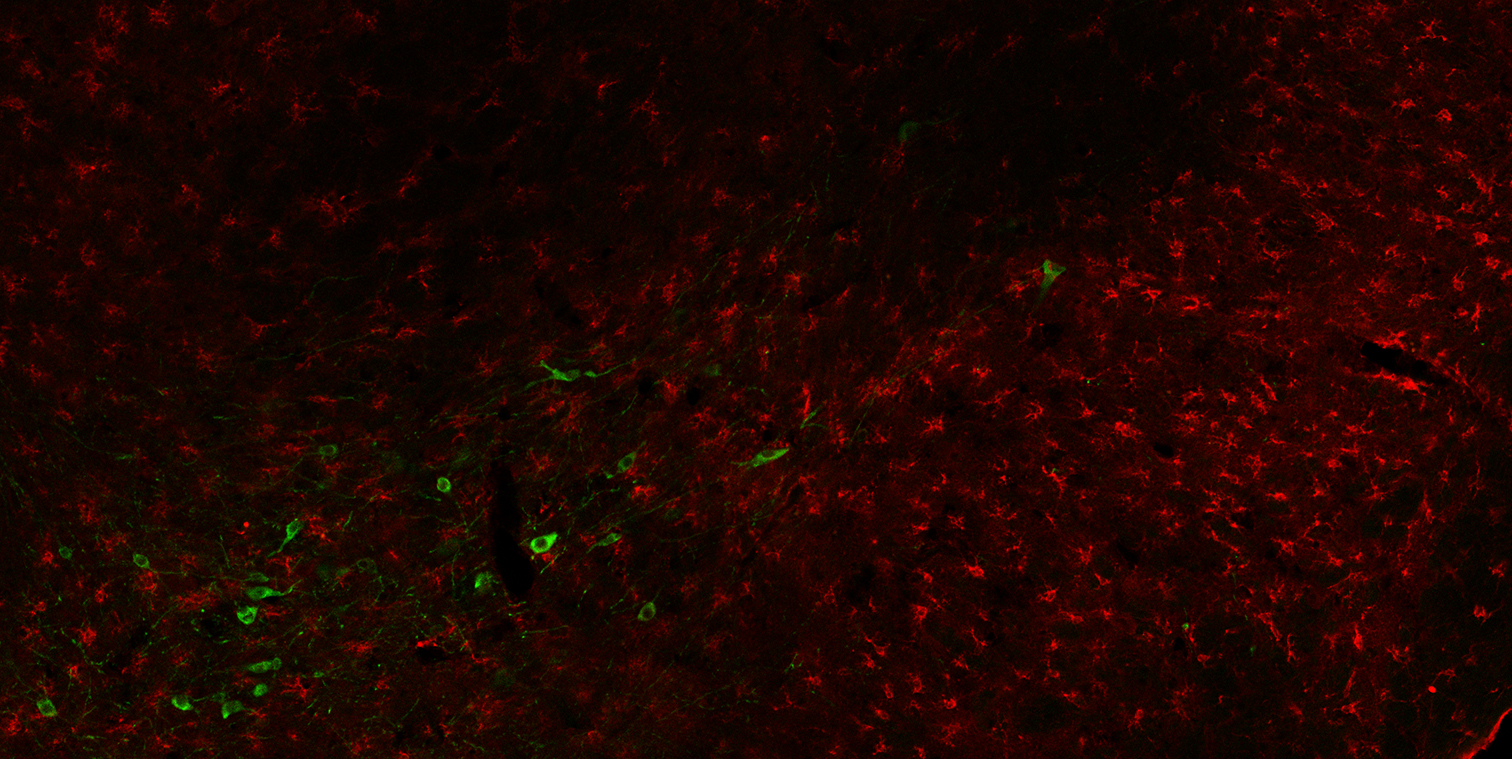

Supplement: Figure 3—source data 2. [file elife-75636-fig3-data2.zip › Fig3 source data 2 for Fig3 C/AAV-shptb SN #19 TH+HA.jpg]

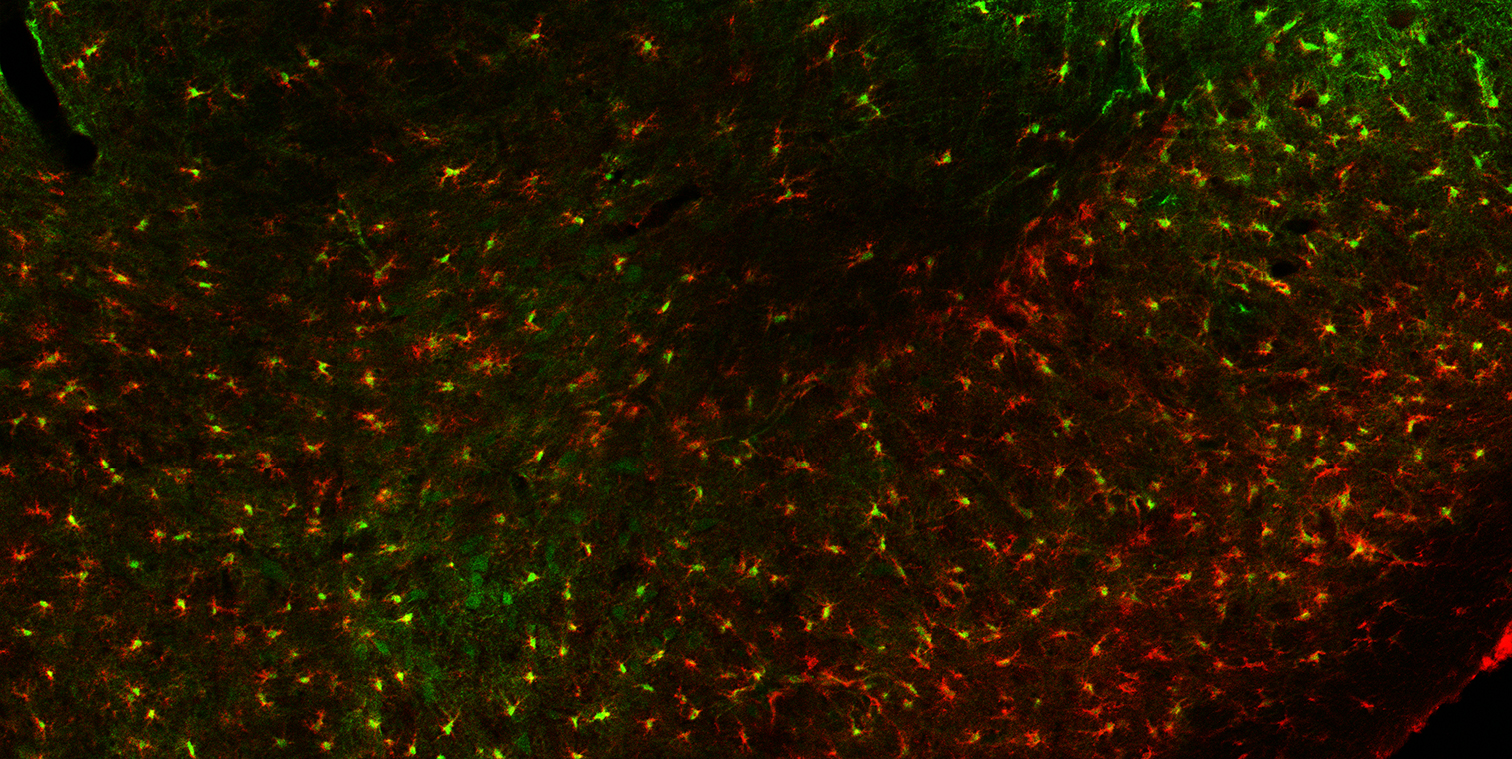

Supplement: Figure 3—source data 2. [file elife-75636-fig3-data2.zip › Fig3 source data 2 for Fig3 C/AAV-shptb SN #21 GFP+HA-1.jpg]

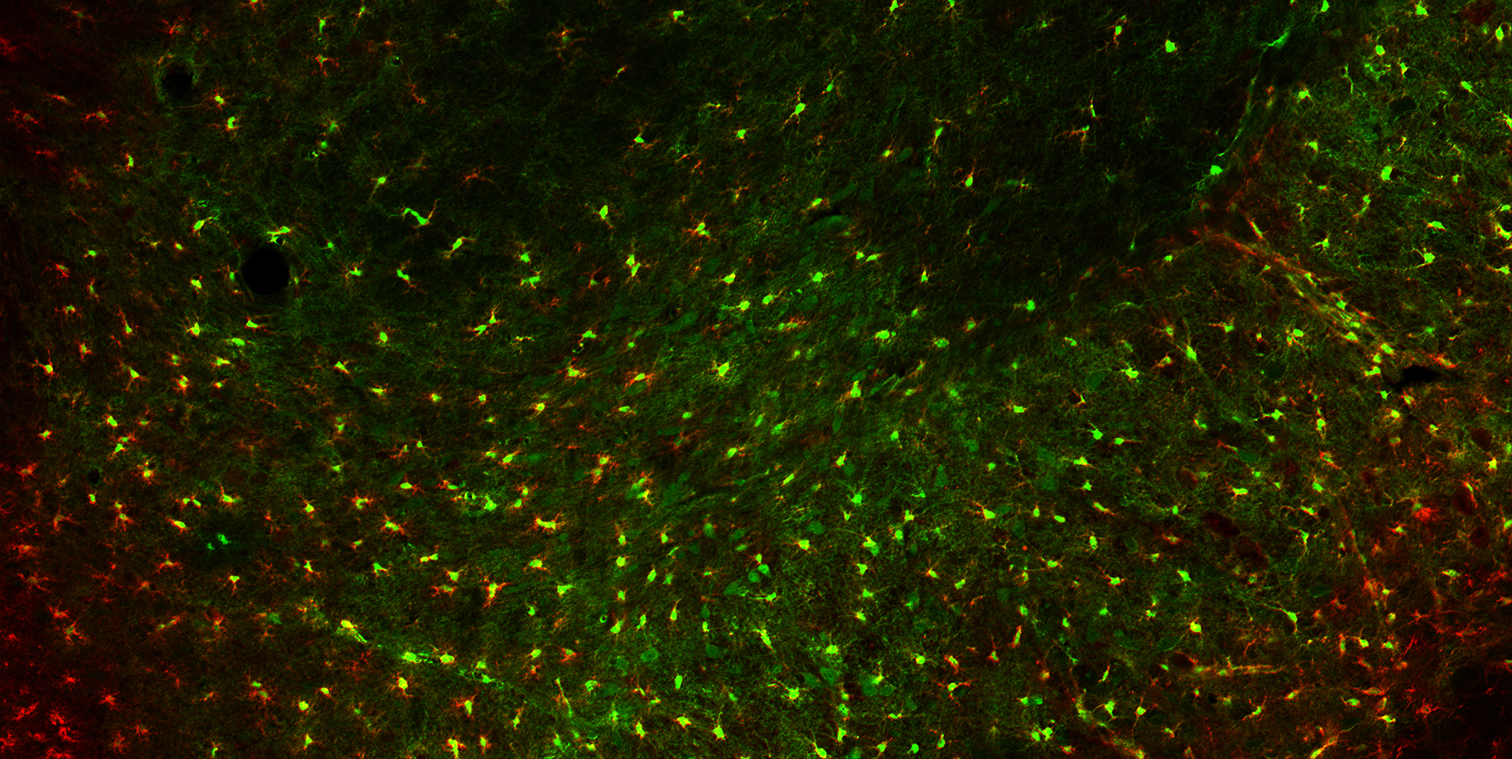

Supplement: Figure 3—source data 2. [file elife-75636-fig3-data2.zip › Fig3 source data 2 for Fig3 C/AAV-shptb SN #21 GFP+HA-2.jpg]

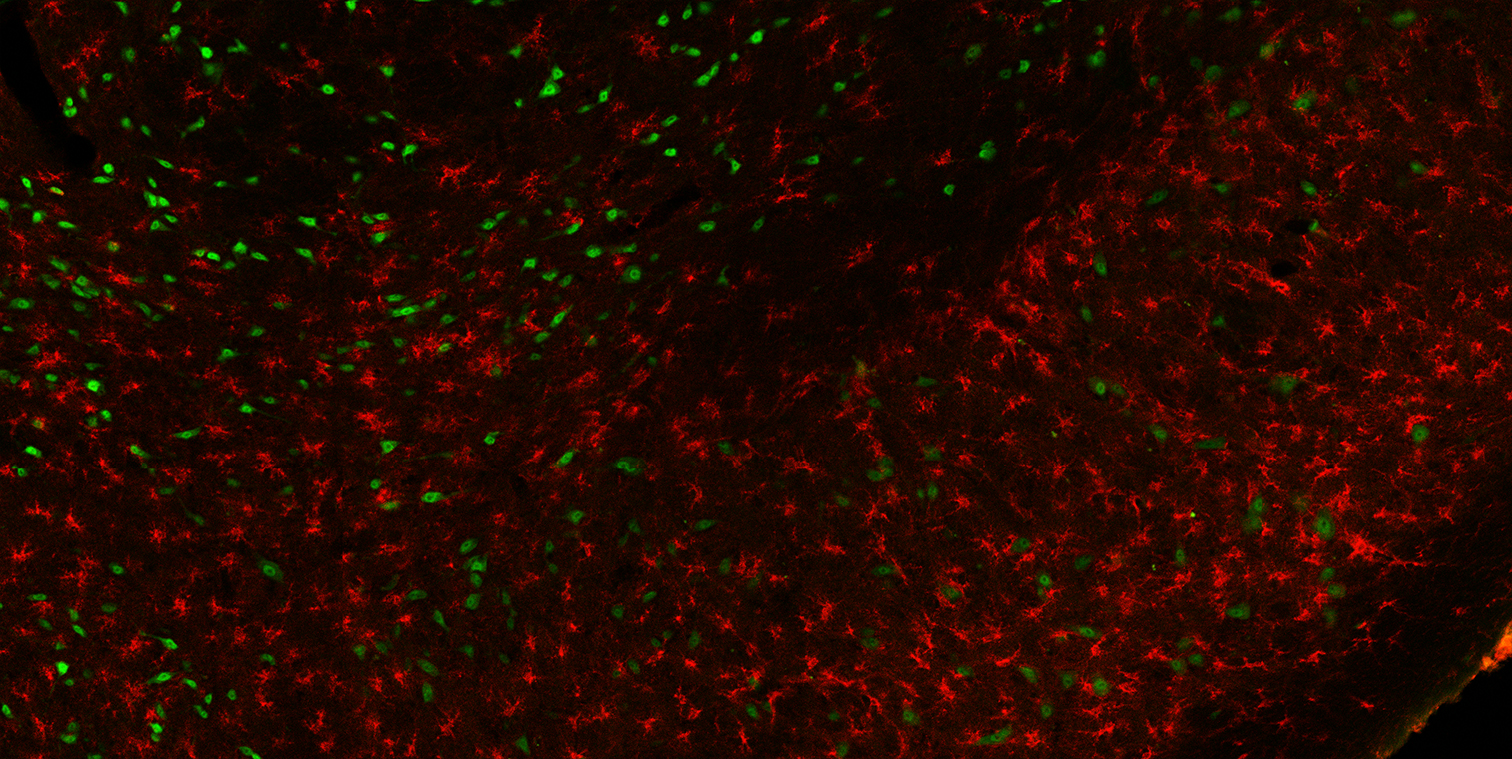

Supplement: Figure 3—source data 2. [file elife-75636-fig3-data2.zip › Fig3 source data 2 for Fig3 C/AAV-shptb SN #21 NeuN+HA.jpg]

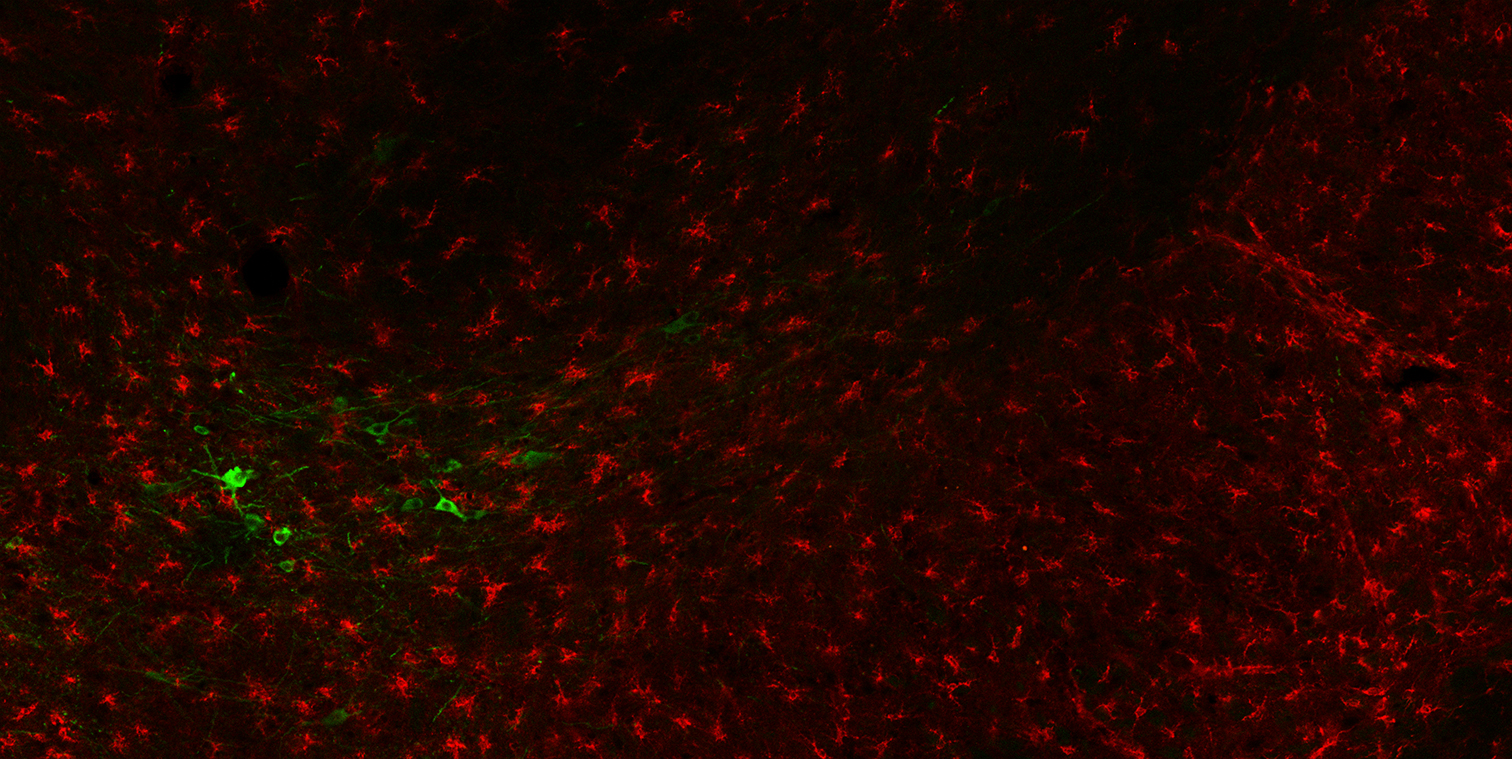

Supplement: Figure 3—source data 2. [file elife-75636-fig3-data2.zip › Fig3 source data 2 for Fig3 C/AAV-shptb SN #21 TH+HA.jpg]

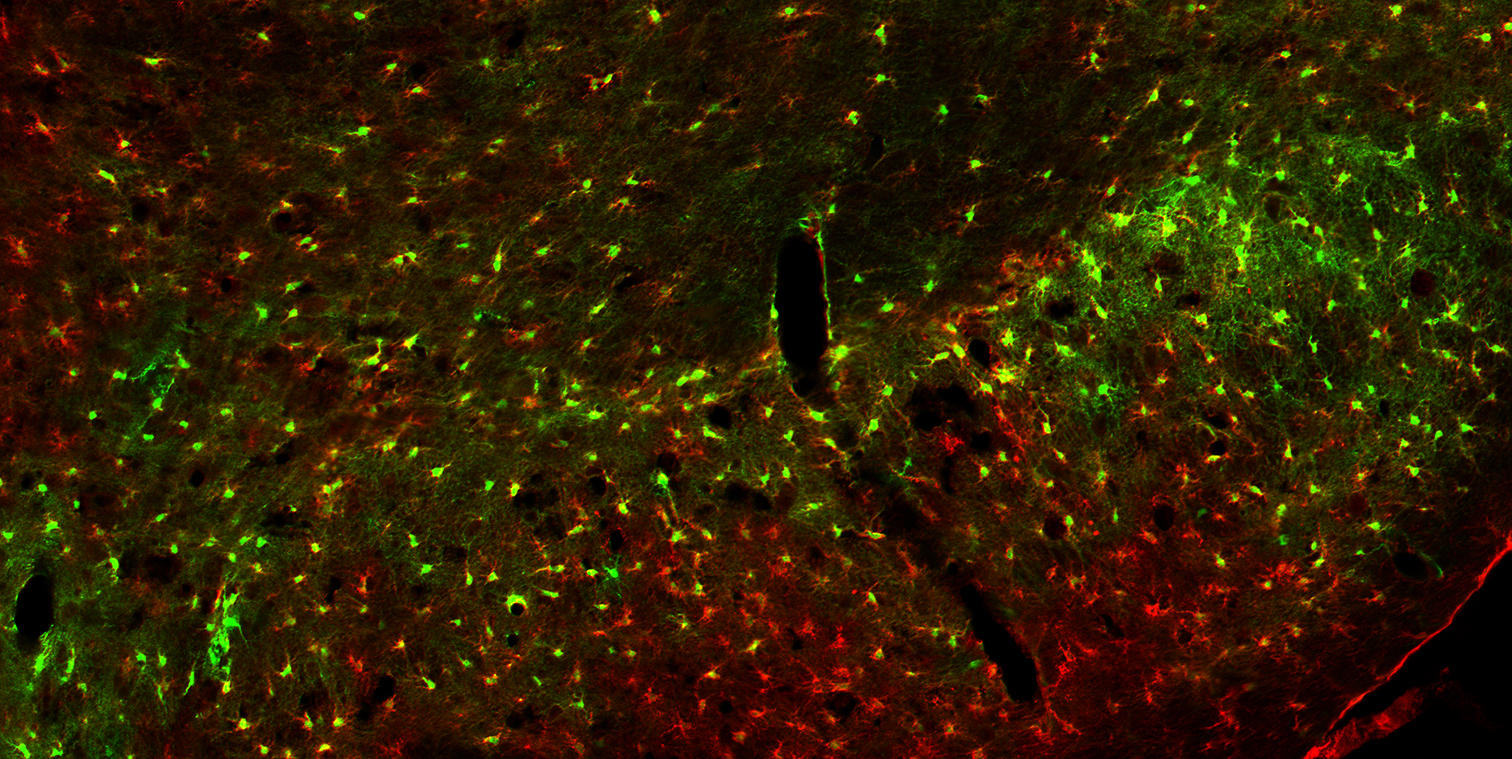

Supplement: Figure 3—source data 2. [file elife-75636-fig3-data2.zip › Fig3 source data 2 for Fig3 C/AAV-shptb SN #24 GFP+HA-1.jpg]

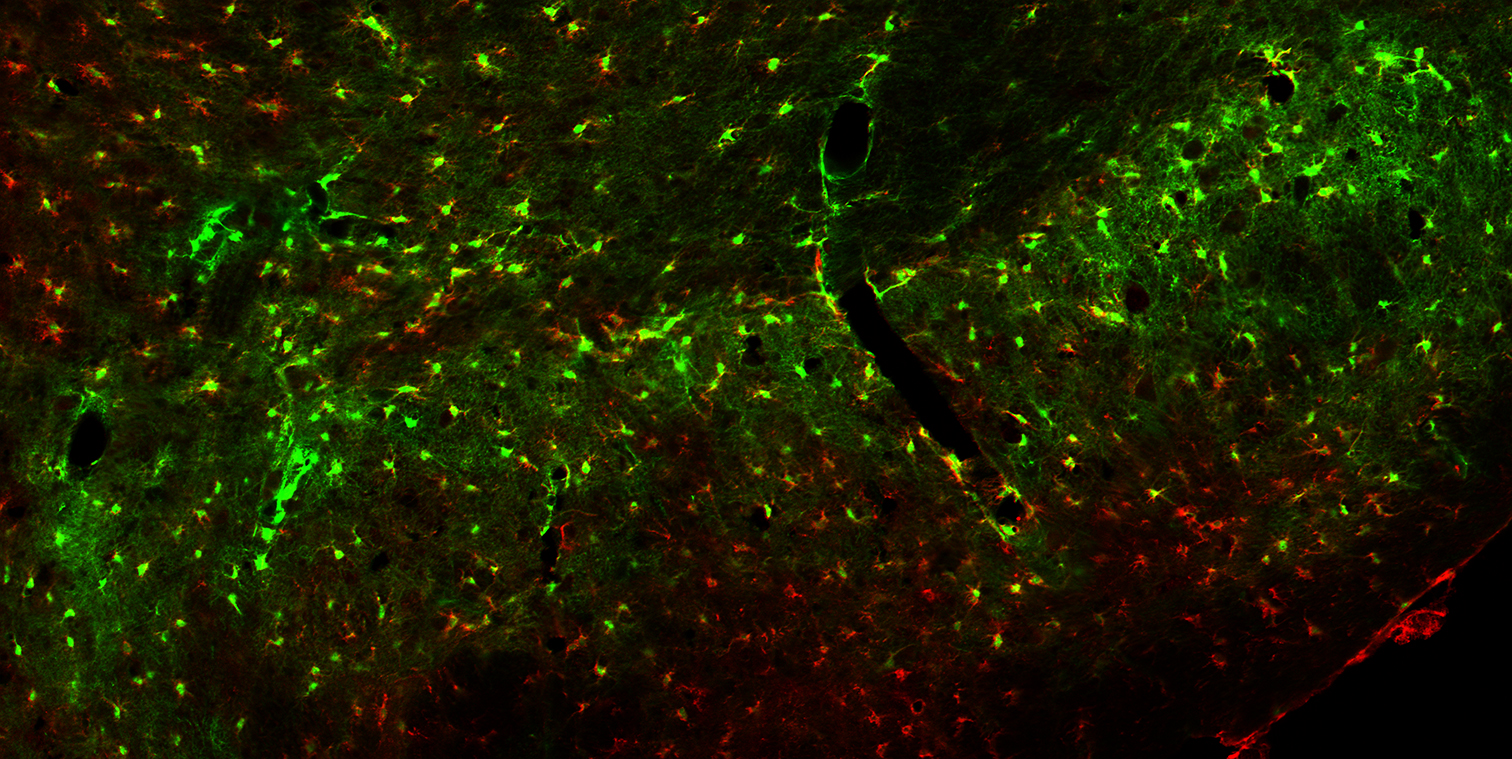

Supplement: Figure 3—source data 2. [file elife-75636-fig3-data2.zip › Fig3 source data 2 for Fig3 C/AAV-shptb SN #24 GFP+HA-2.jpg]

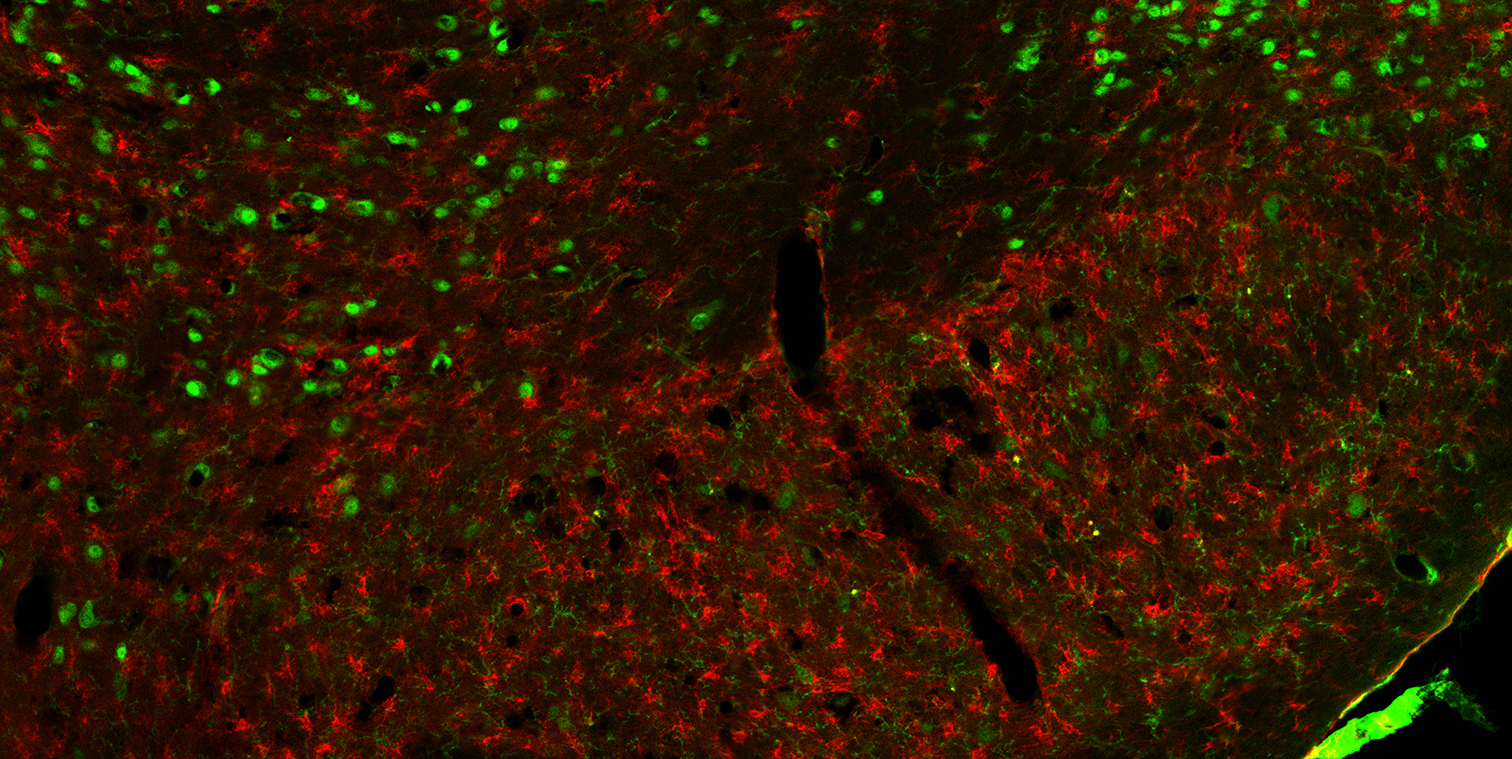

Supplement: Figure 3—source data 2. [file elife-75636-fig3-data2.zip › Fig3 source data 2 for Fig3 C/AAV-shptb SN #24 NeuN+HA.jpg]

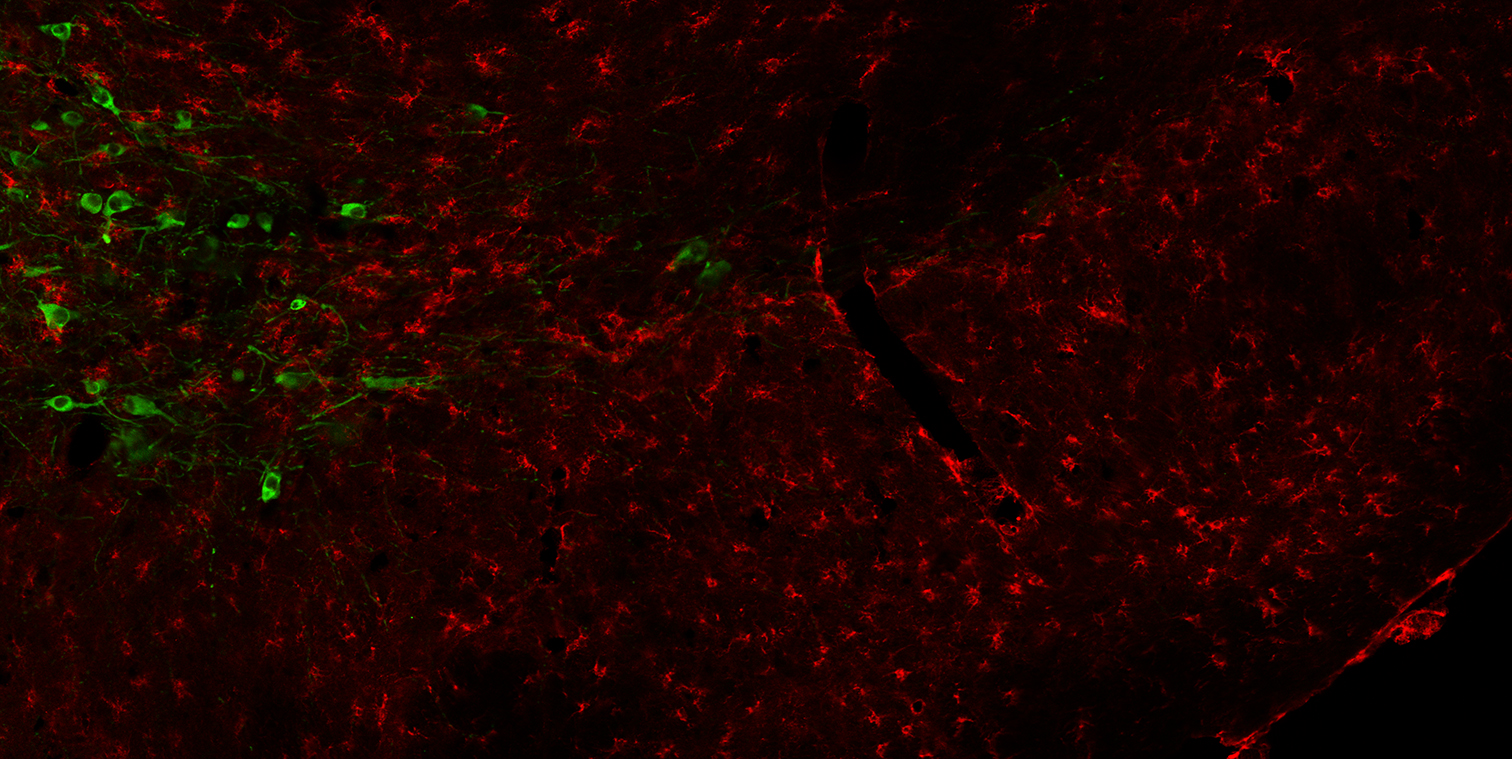

Supplement: Figure 3—source data 2. [file elife-75636-fig3-data2.zip › Fig3 source data 2 for Fig3 C/AAV-shptb SN #24 TH+HA.jpg]

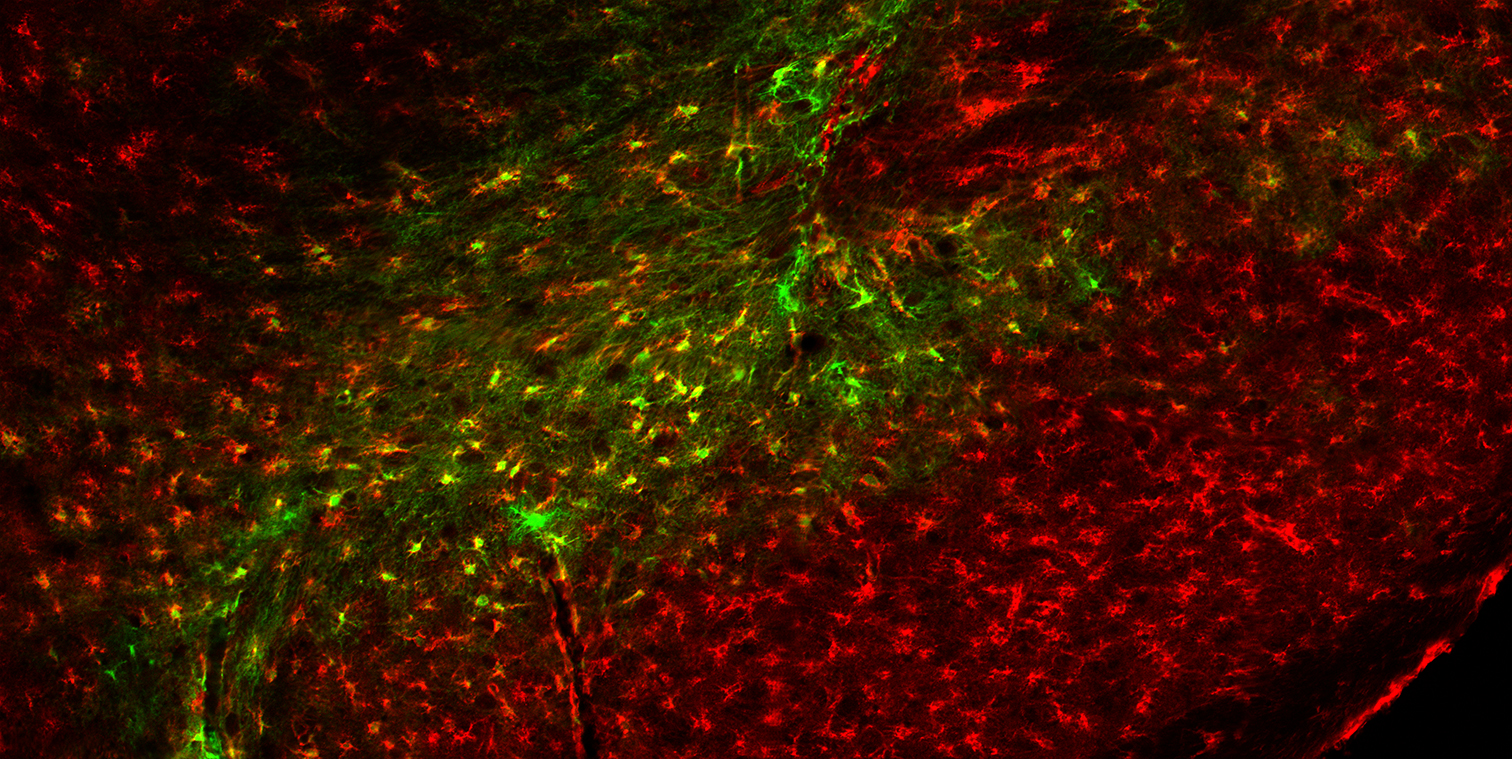

Supplement: Figure 3—source data 2. [file elife-75636-fig3-data2.zip › Fig3 source data 2 for Fig3 C/AAV-shptb SN #66 GFP+HA-1.jpg]

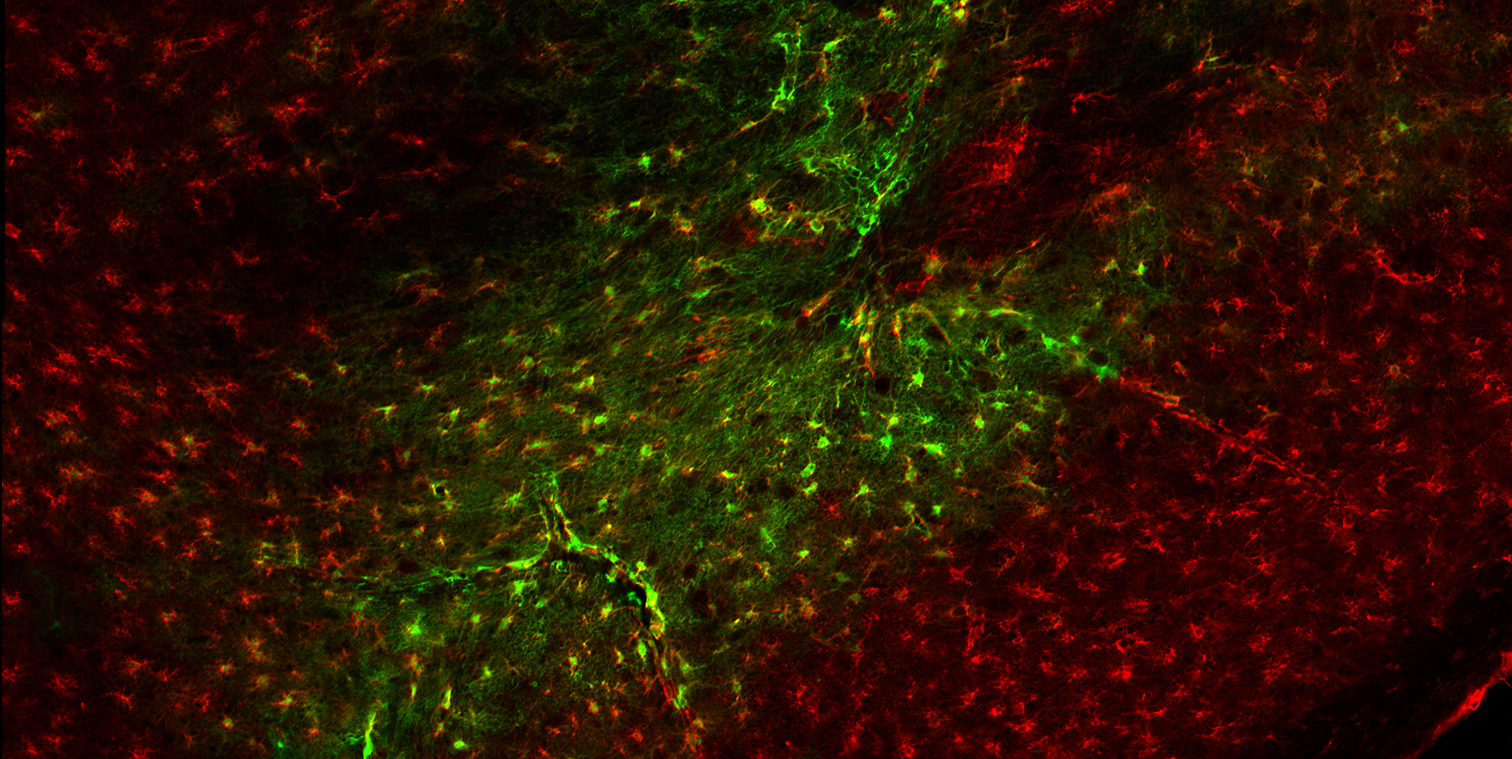

Supplement: Figure 3—source data 2. [file elife-75636-fig3-data2.zip › Fig3 source data 2 for Fig3 C/AAV-shptb SN #66 GFP+HA-2.jpg]

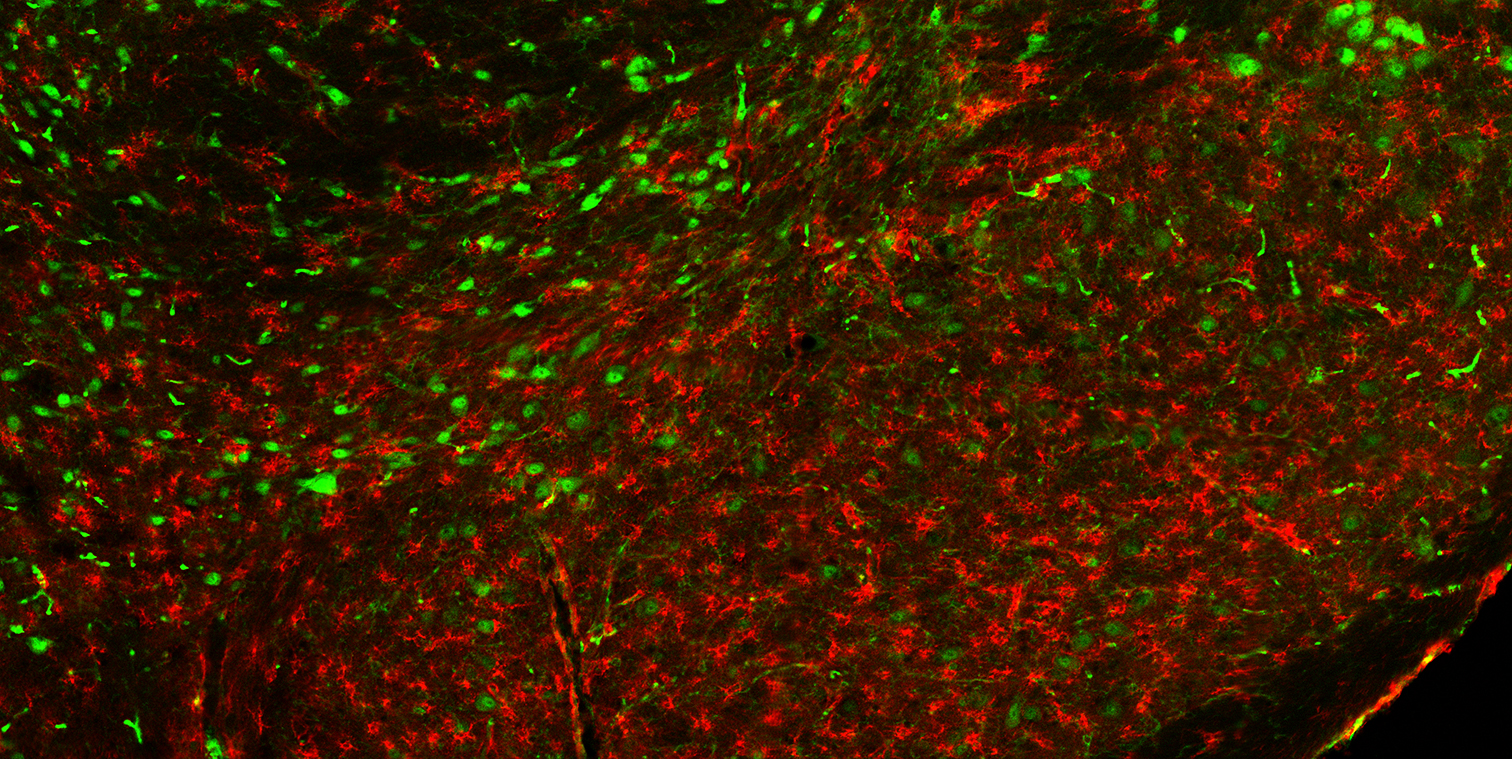

Supplement: Figure 3—source data 2. [file elife-75636-fig3-data2.zip › Fig3 source data 2 for Fig3 C/AAV-shptb SN #66 NeuN+HA.jpg]

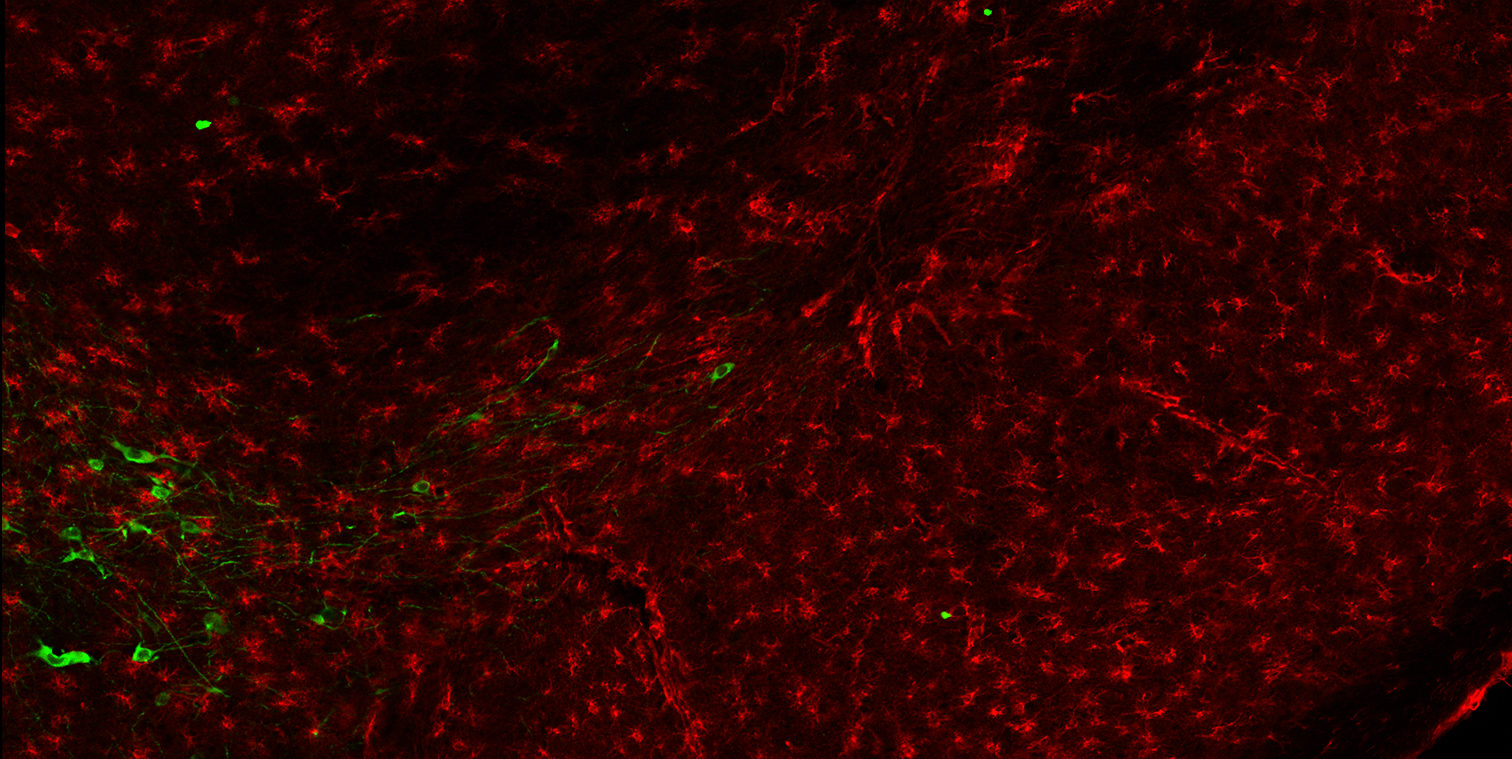

Supplement: Figure 3—source data 2. [file elife-75636-fig3-data2.zip › Fig3 source data 2 for Fig3 C/AAV-shptb SN #66 TH+HA.jpg]

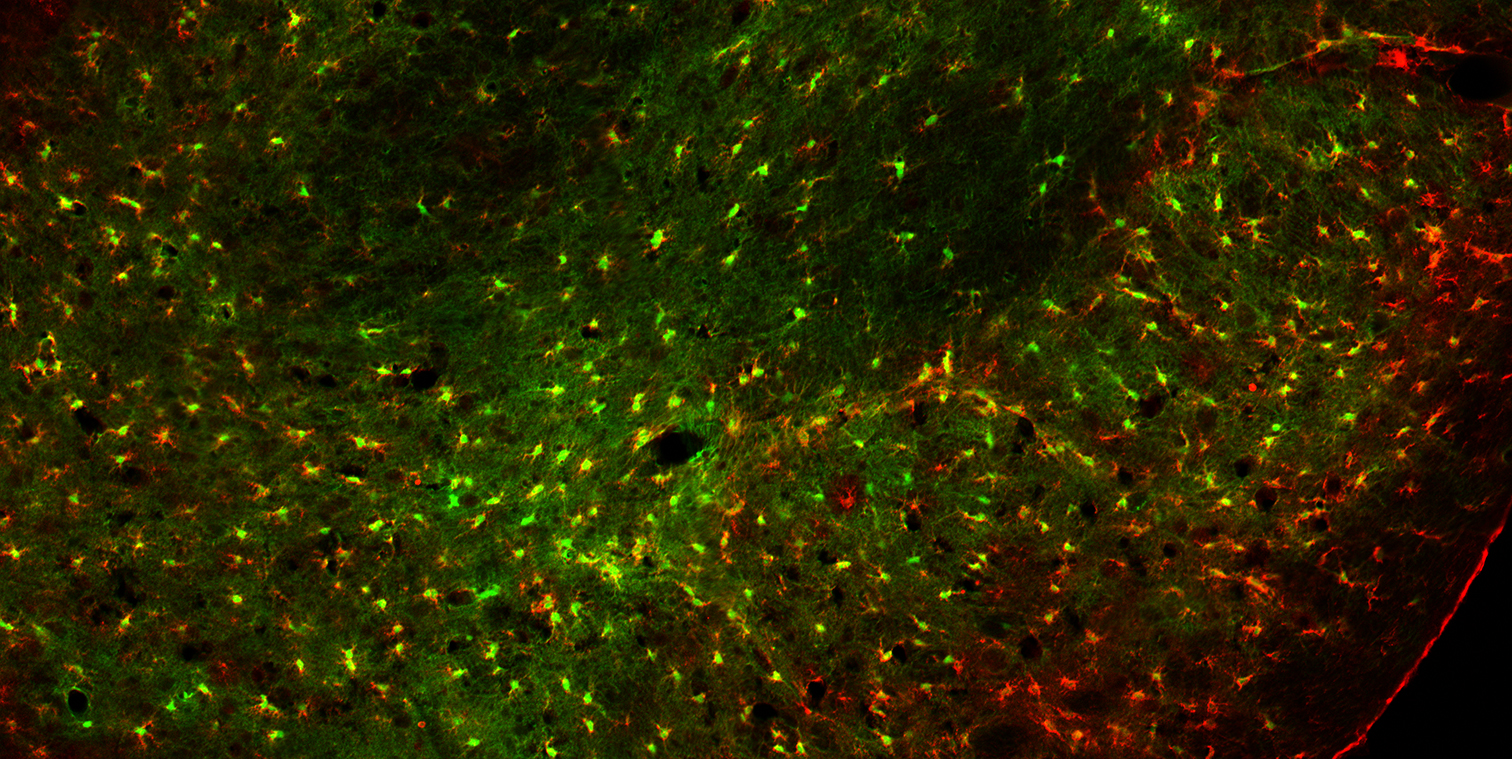

Supplement: Figure 3—source data 2. [file elife-75636-fig3-data2.zip › Fig3 source data 2 for Fig3 C/AAV-shptb SN #74 GFP+HA-1.jpg]

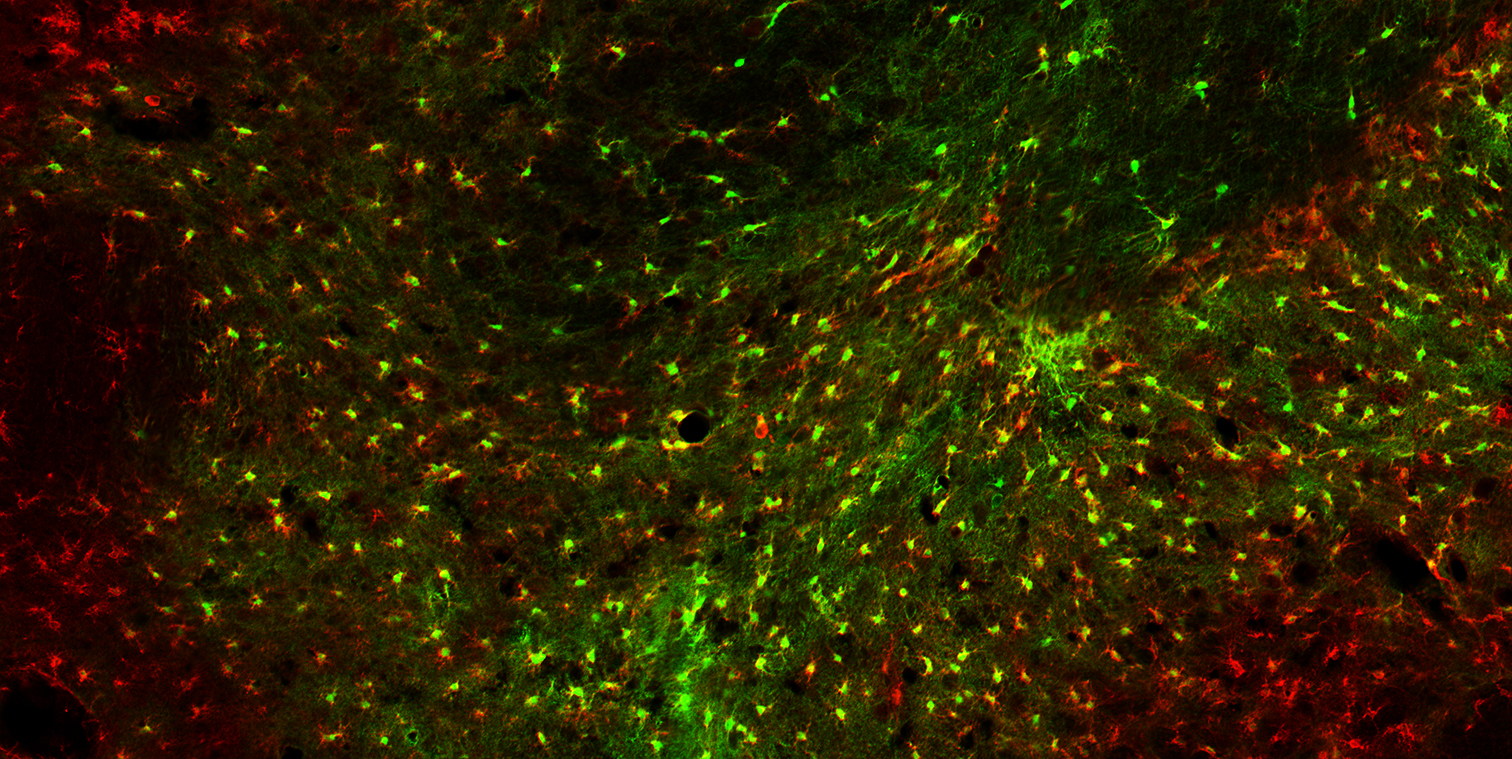

Supplement: Figure 3—source data 2. [file elife-75636-fig3-data2.zip › Fig3 source data 2 for Fig3 C/AAV-shptb SN #74 GFP+HA-2.jpg]

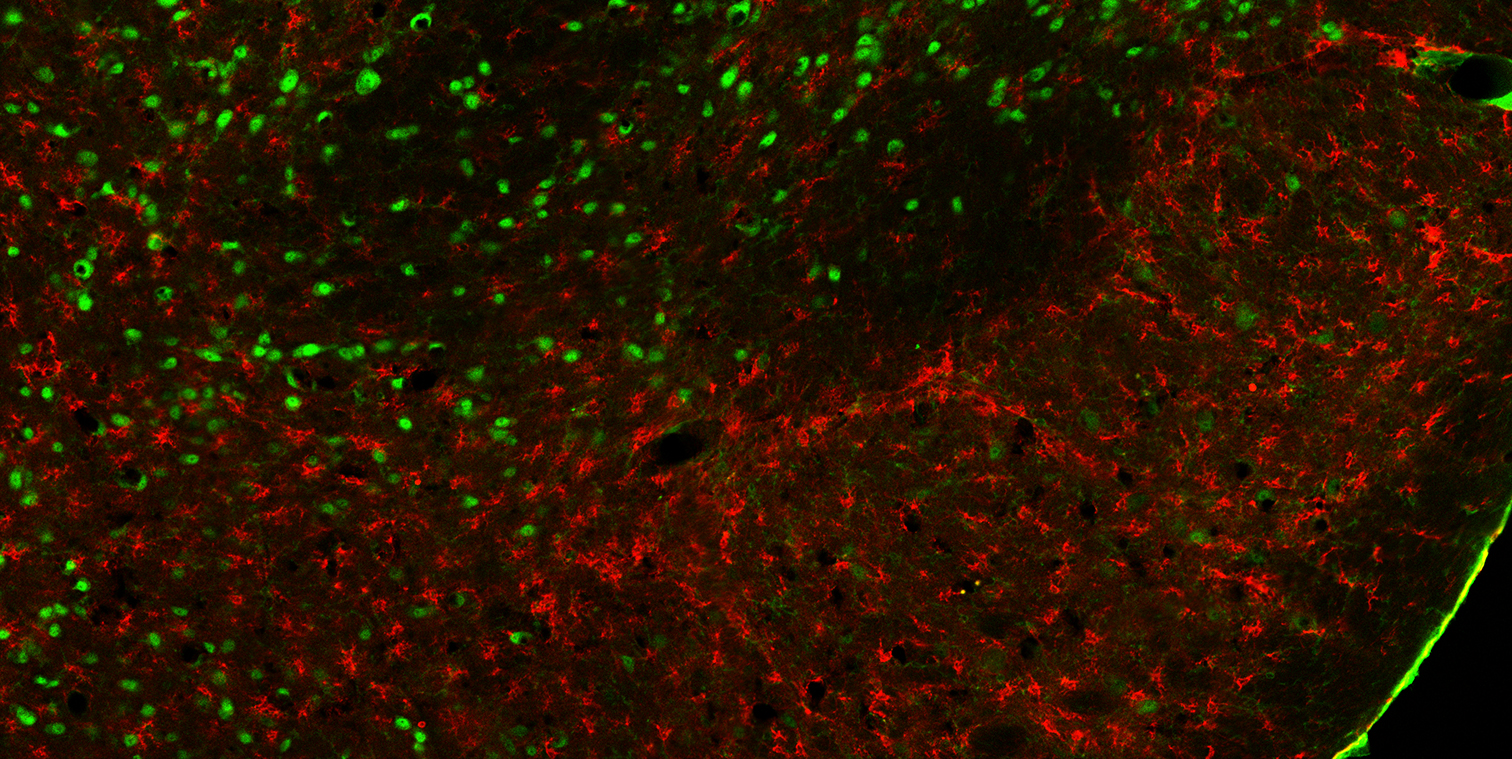

Supplement: Figure 3—source data 2. [file elife-75636-fig3-data2.zip › Fig3 source data 2 for Fig3 C/AAV-shptb SN #74 NeuN+HA.jpg]

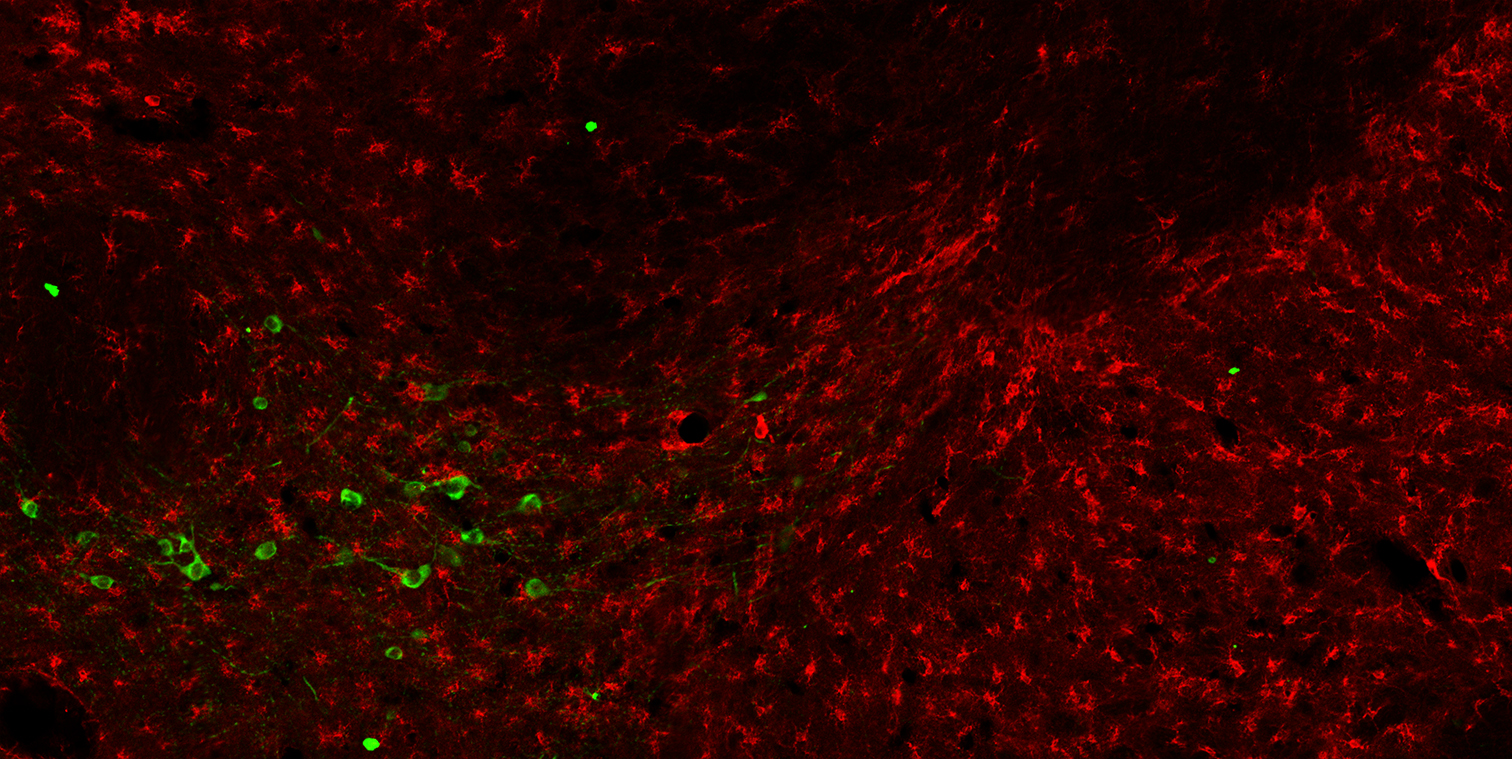

Supplement: Figure 3—source data 2. [file elife-75636-fig3-data2.zip › Fig3 source data 2 for Fig3 C/AAV-shptb SN #74 TH+HA.jpg]

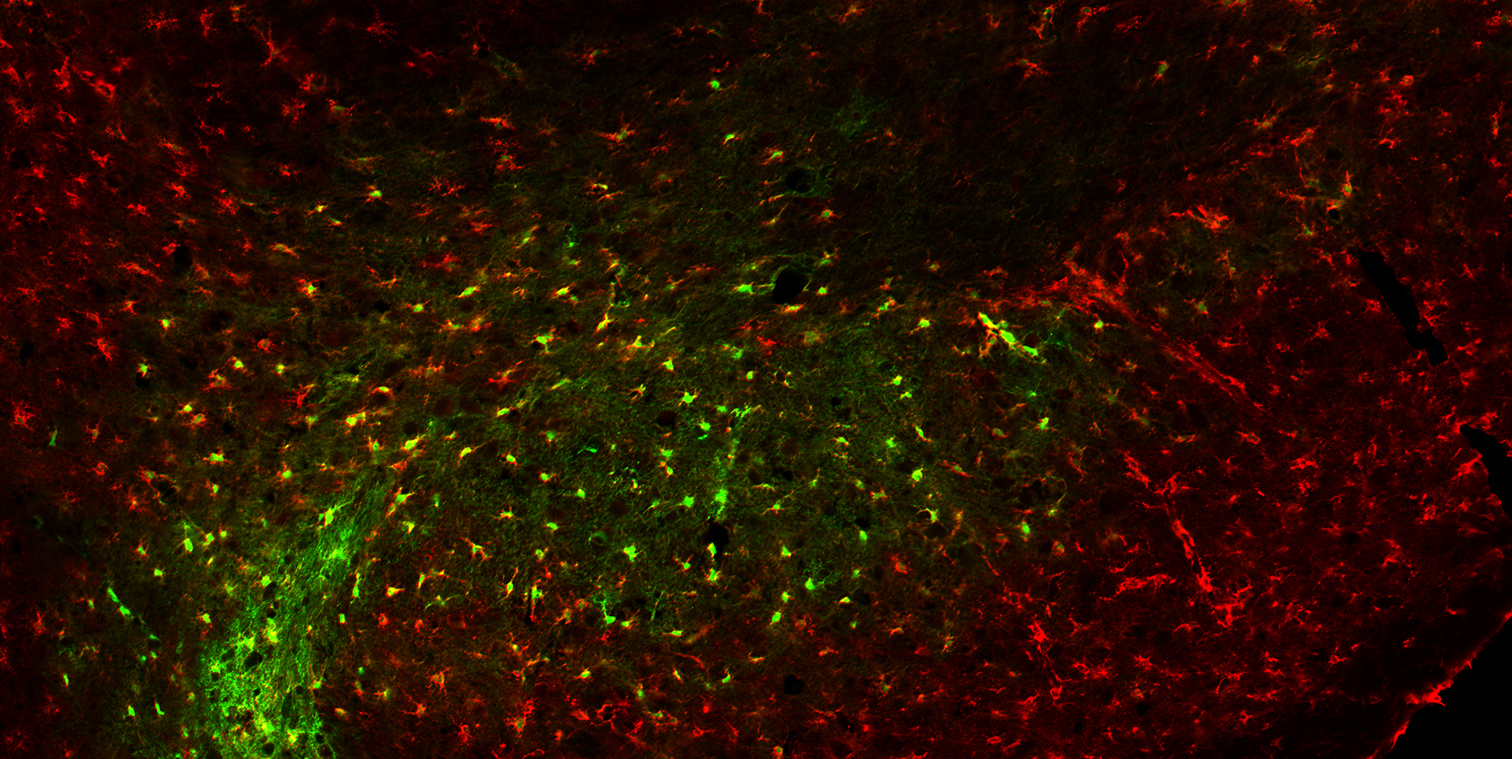

Supplement: Figure 3—source data 2. [file elife-75636-fig3-data2.zip › Fig3 source data 2 for Fig3 C/AAV-shptb SN #76 GFP+HA-1.jpg]

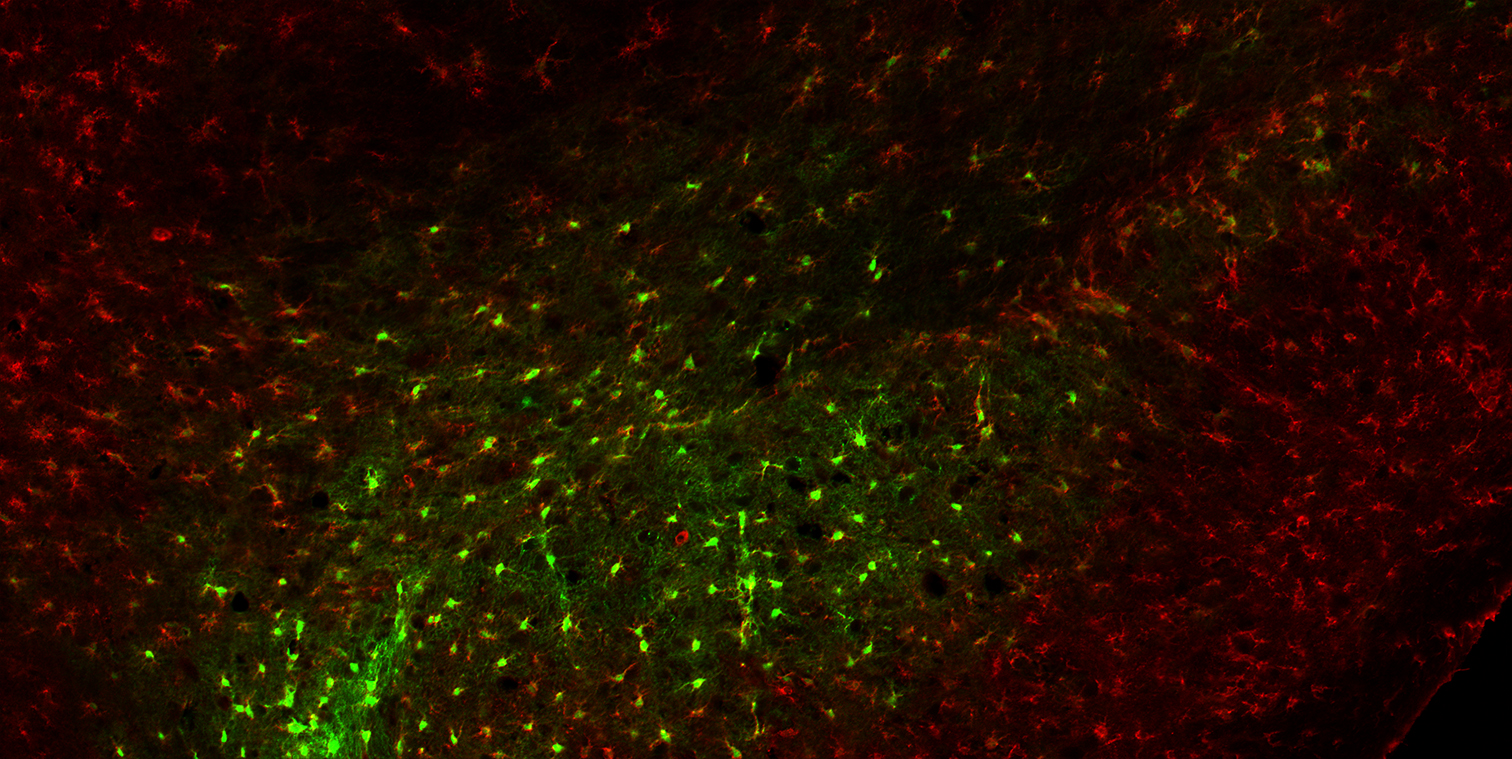

Supplement: Figure 3—source data 2. [file elife-75636-fig3-data2.zip › Fig3 source data 2 for Fig3 C/AAV-shptb SN #76 GFP+HA-2.jpg]

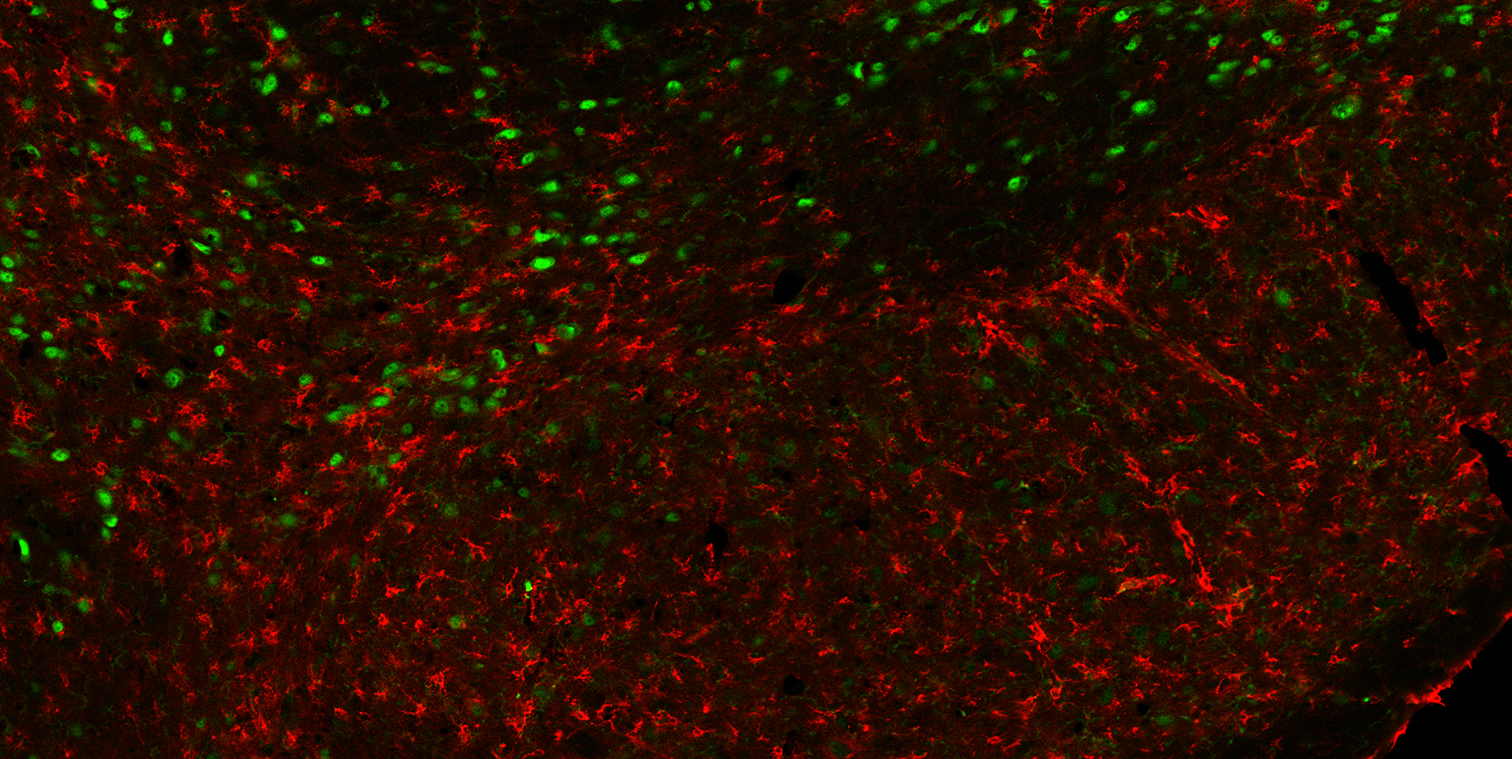

Supplement: Figure 3—source data 2. [file elife-75636-fig3-data2.zip › Fig3 source data 2 for Fig3 C/AAV-shptb SN #76 NeuN+HA.jpg]

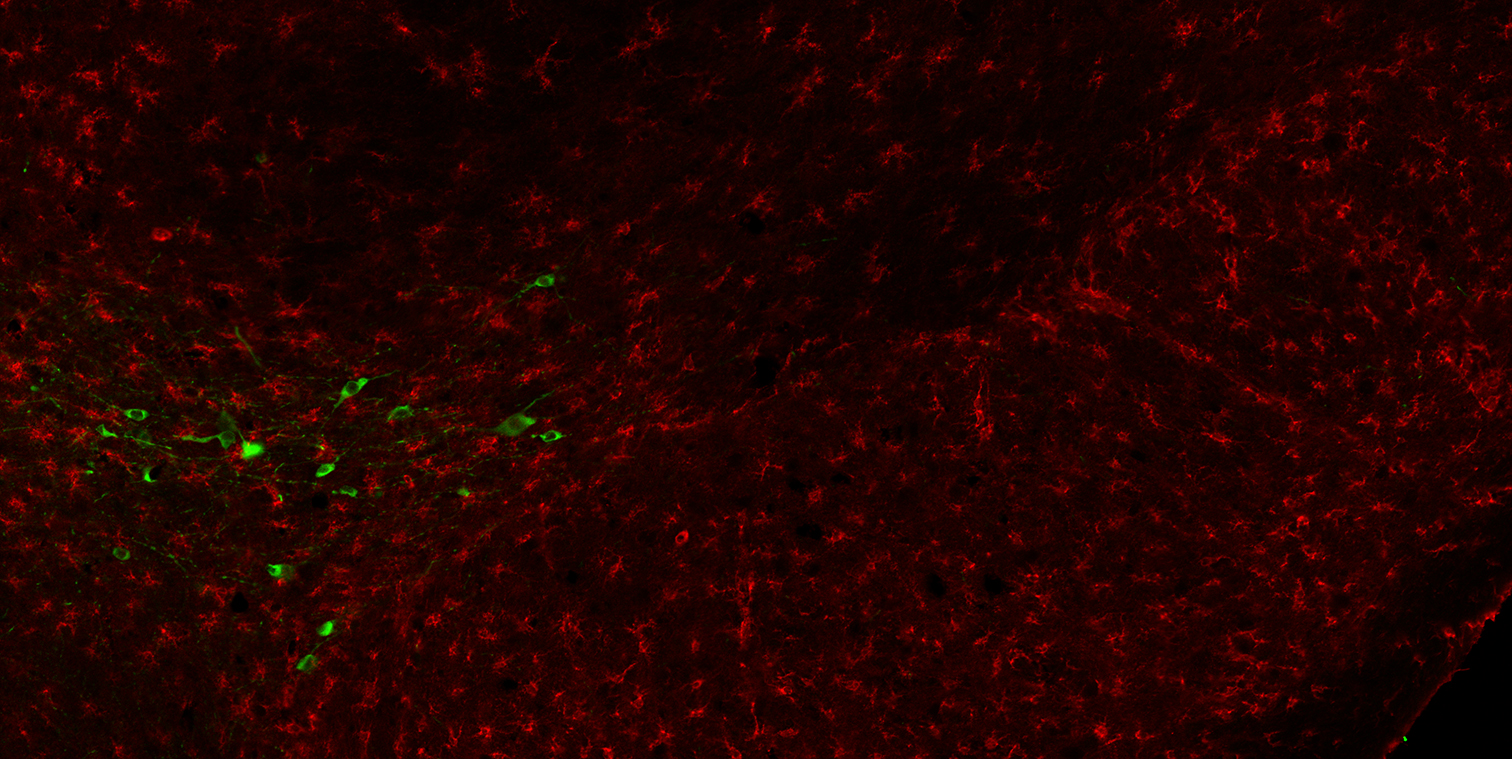

Supplement: Figure 3—source data 2. [file elife-75636-fig3-data2.zip › Fig3 source data 2 for Fig3 C/AAV-shptb SN #76 TH+HA.jpg]

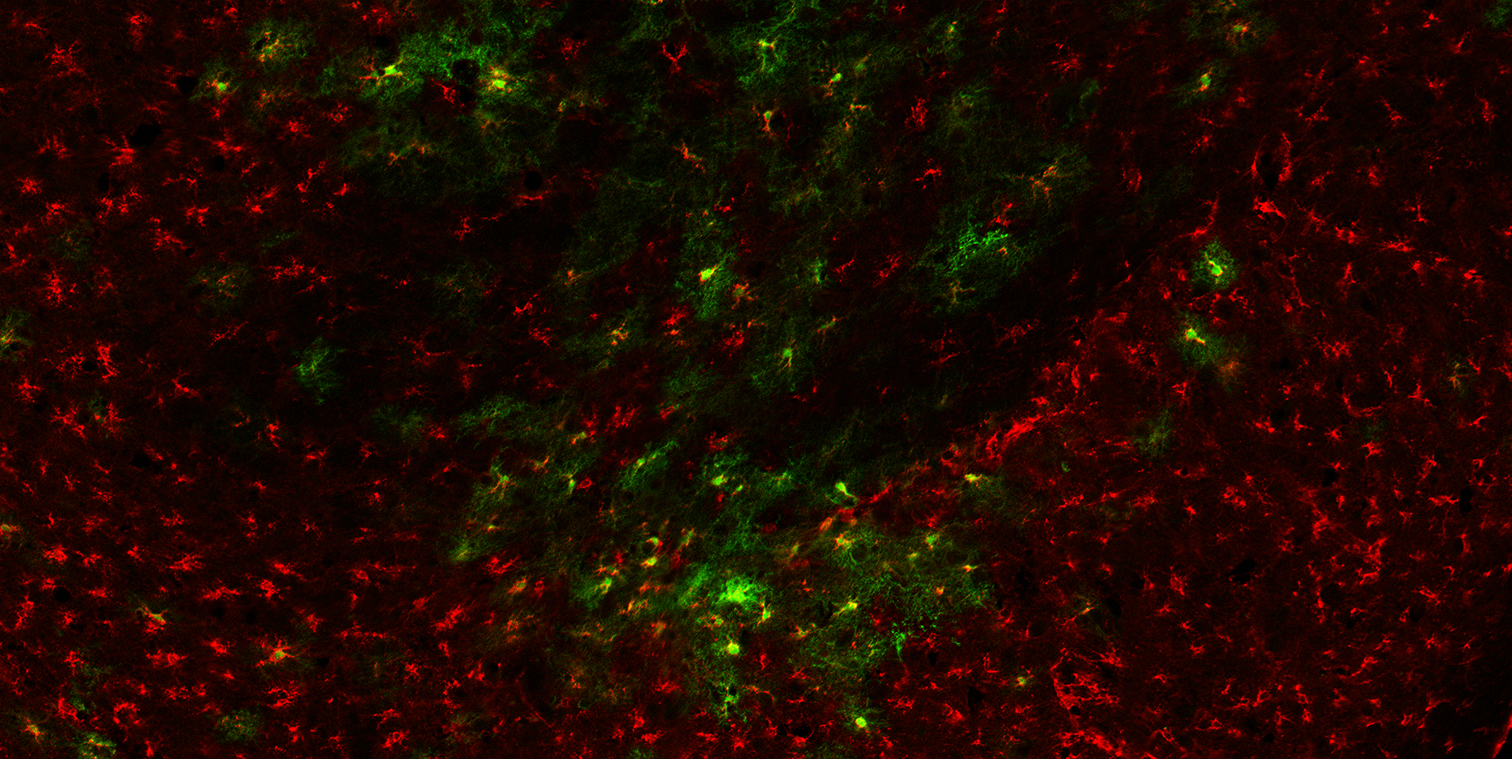

Supplement: Figure 3—source data 2. [file elife-75636-fig3-data2.zip › Fig3 source data 2 for Fig3 C/AAV-shptb SN #84 GFP+HA-1.jpg]

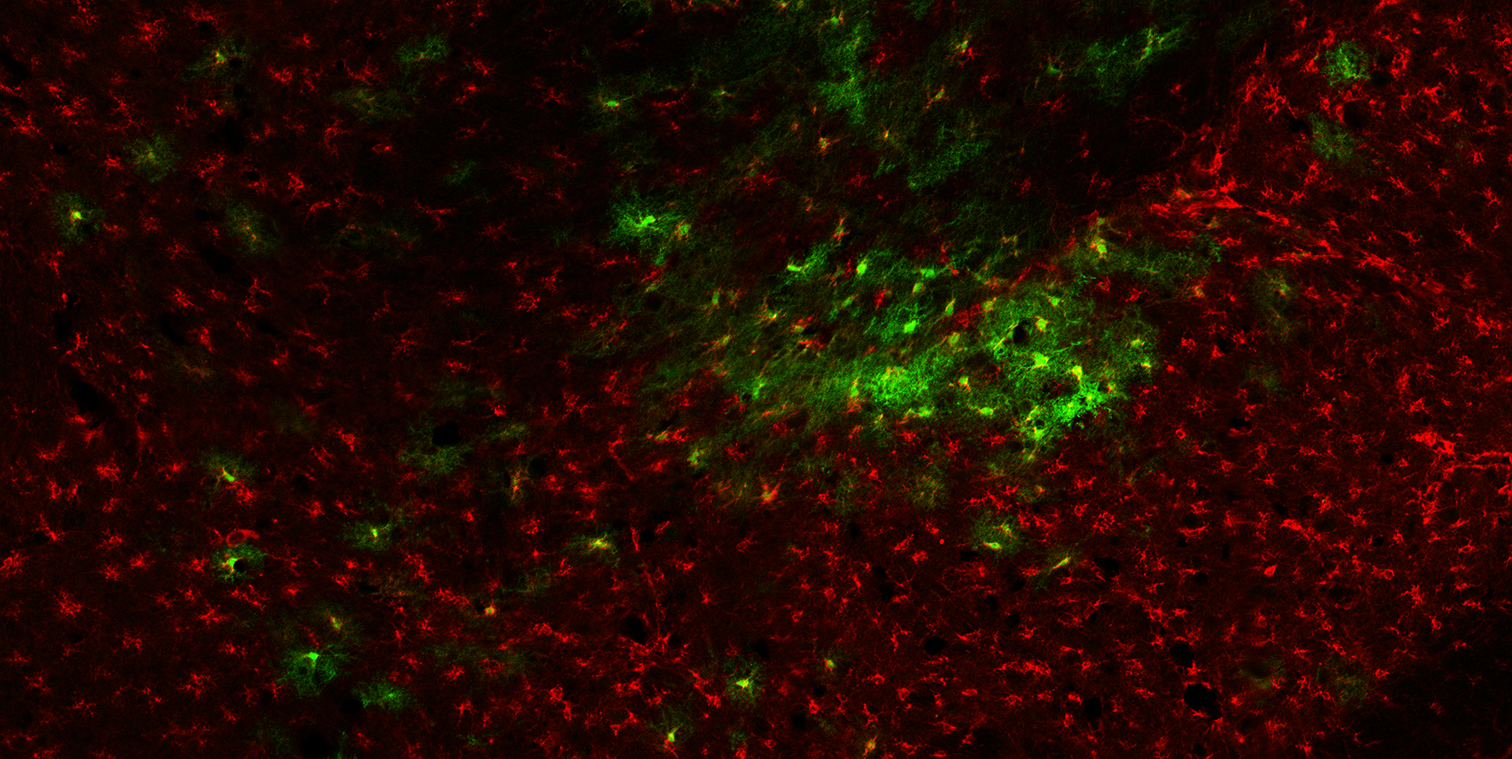

Supplement: Figure 3—source data 2. [file elife-75636-fig3-data2.zip › Fig3 source data 2 for Fig3 C/AAV-shptb SN #84 GFP+HA-2.jpg]

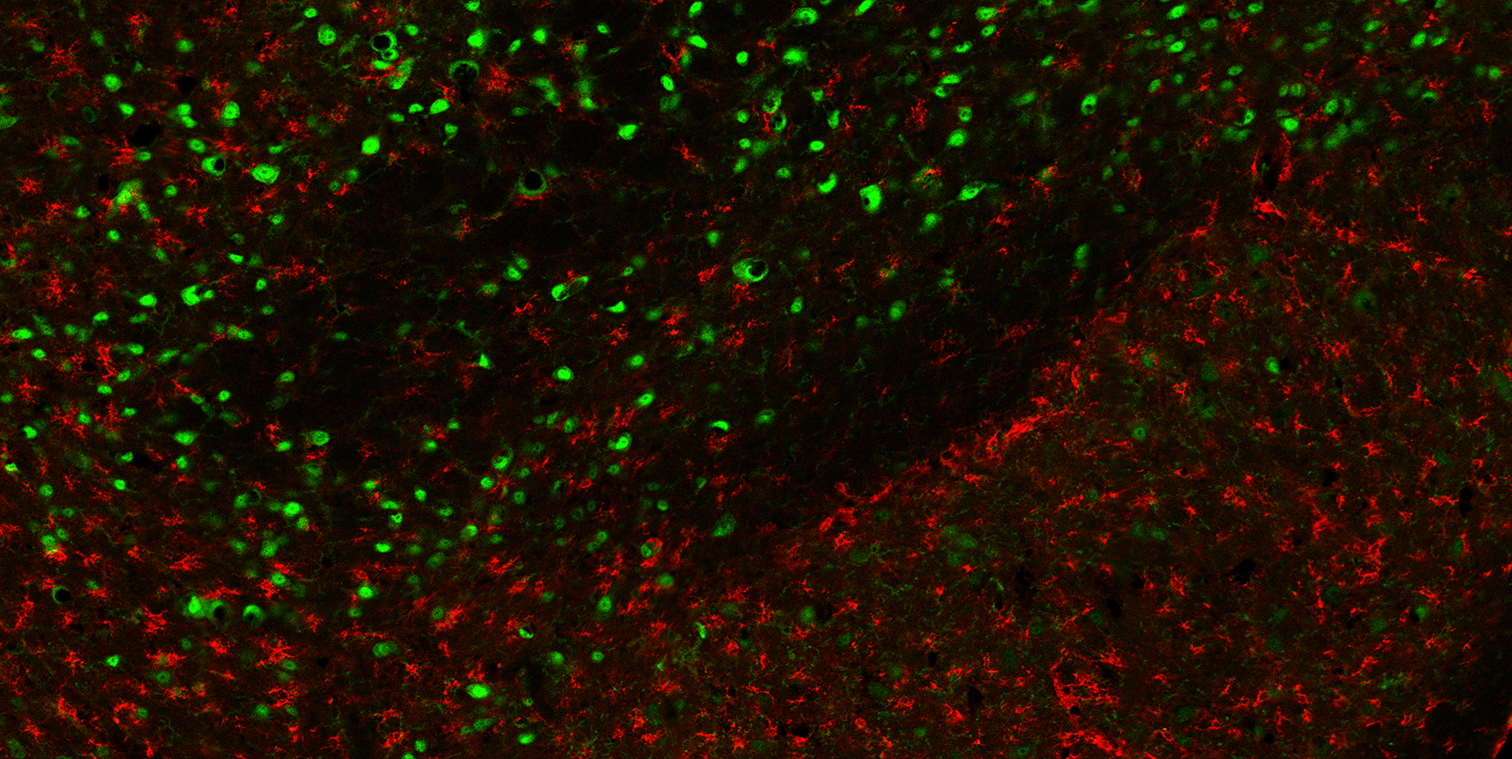

Supplement: Figure 3—source data 2. [file elife-75636-fig3-data2.zip › Fig3 source data 2 for Fig3 C/AAV-shptb SN #84 NeuN+HA.jpg]

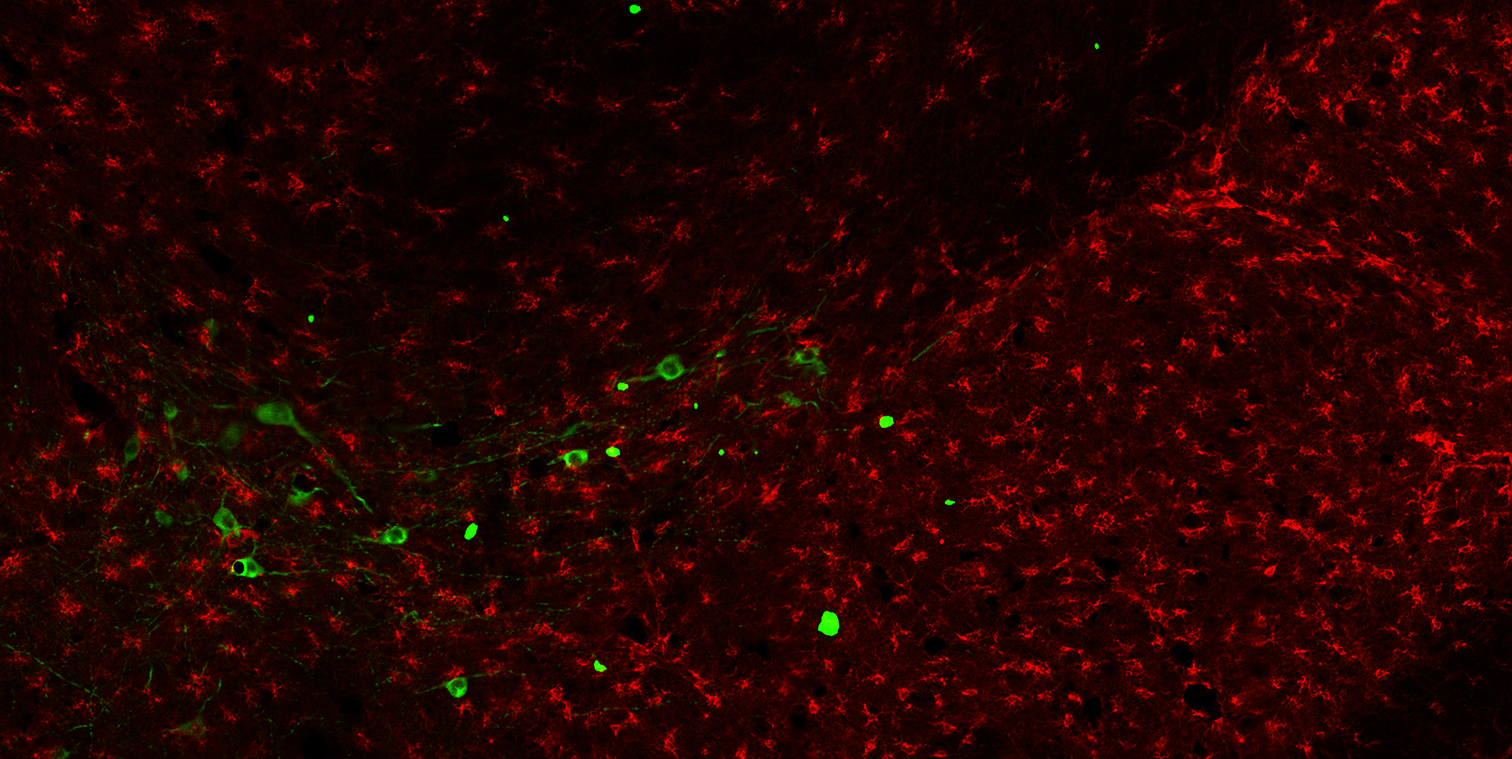

Supplement: Figure 3—source data 2. [file elife-75636-fig3-data2.zip › Fig3 source data 2 for Fig3 C/AAV-shptb SN #84 TH+HA.jpg]

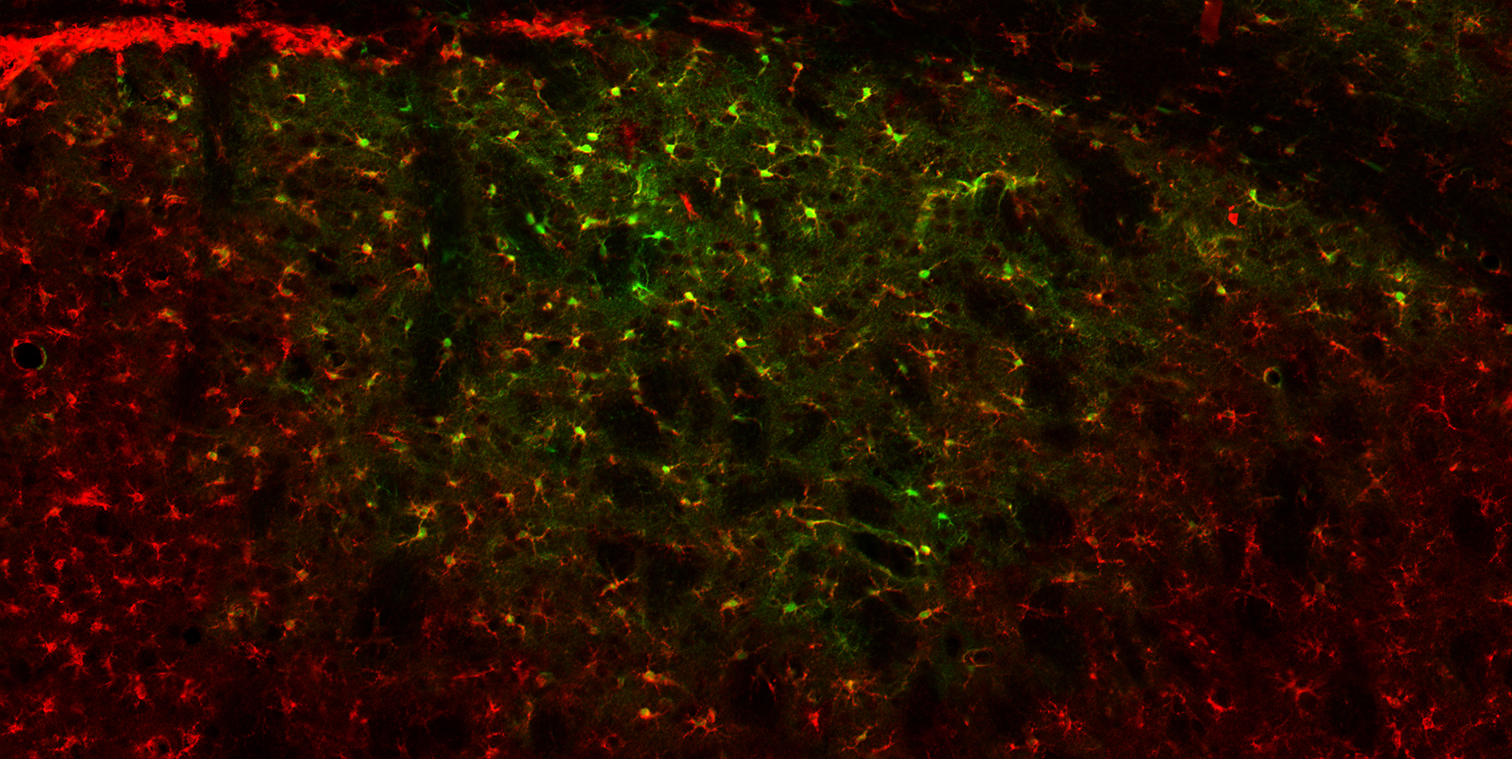

Supplement: Figure 3—source data 2. [file elife-75636-fig3-data2.zip › Fig3 source data 2 for Fig3 C/AAV-shptb STR #20 GFP+HA-1.jpg]

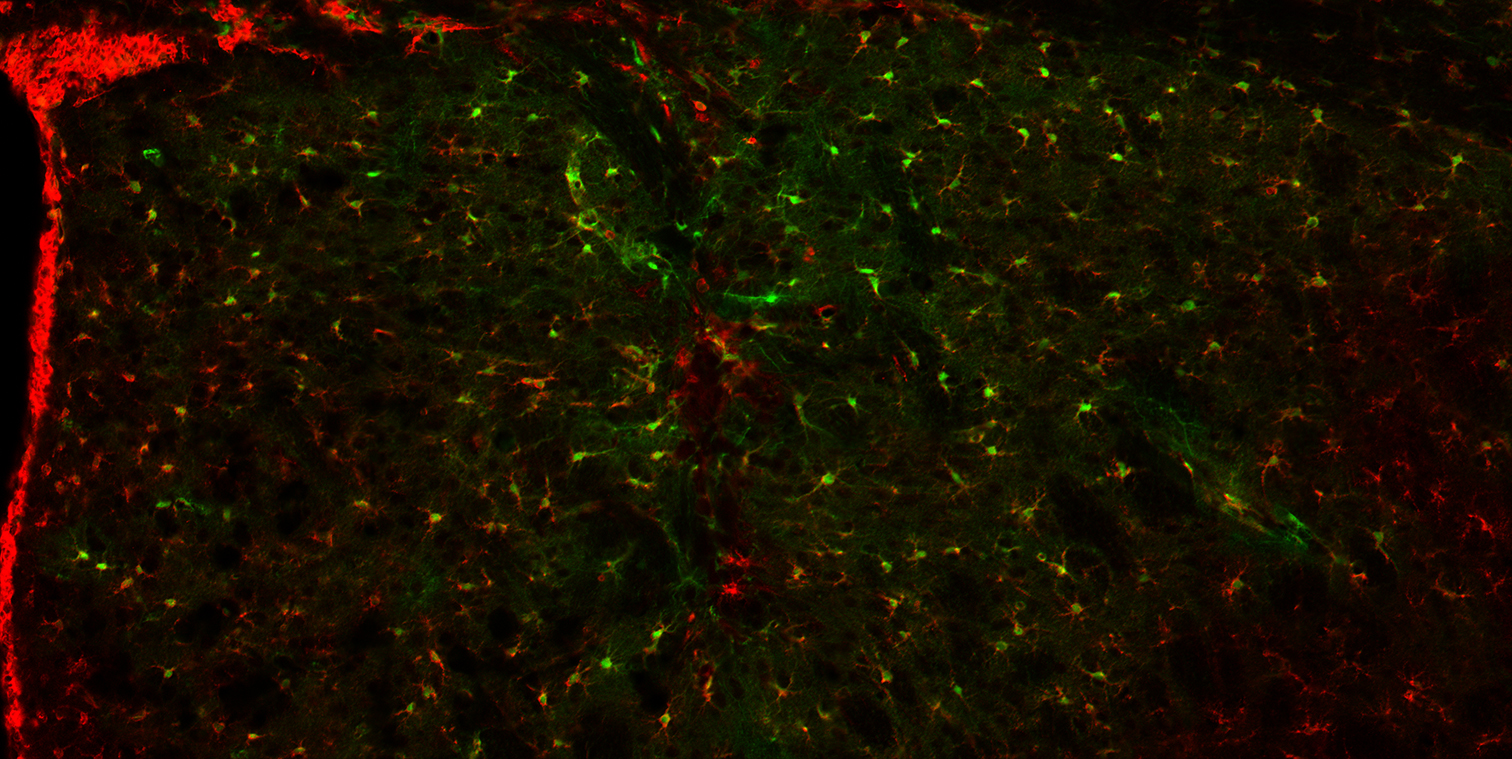

Supplement: Figure 3—source data 2. [file elife-75636-fig3-data2.zip › Fig3 source data 2 for Fig3 C/AAV-shptb STR #20 GFP+HA-2.jpg]

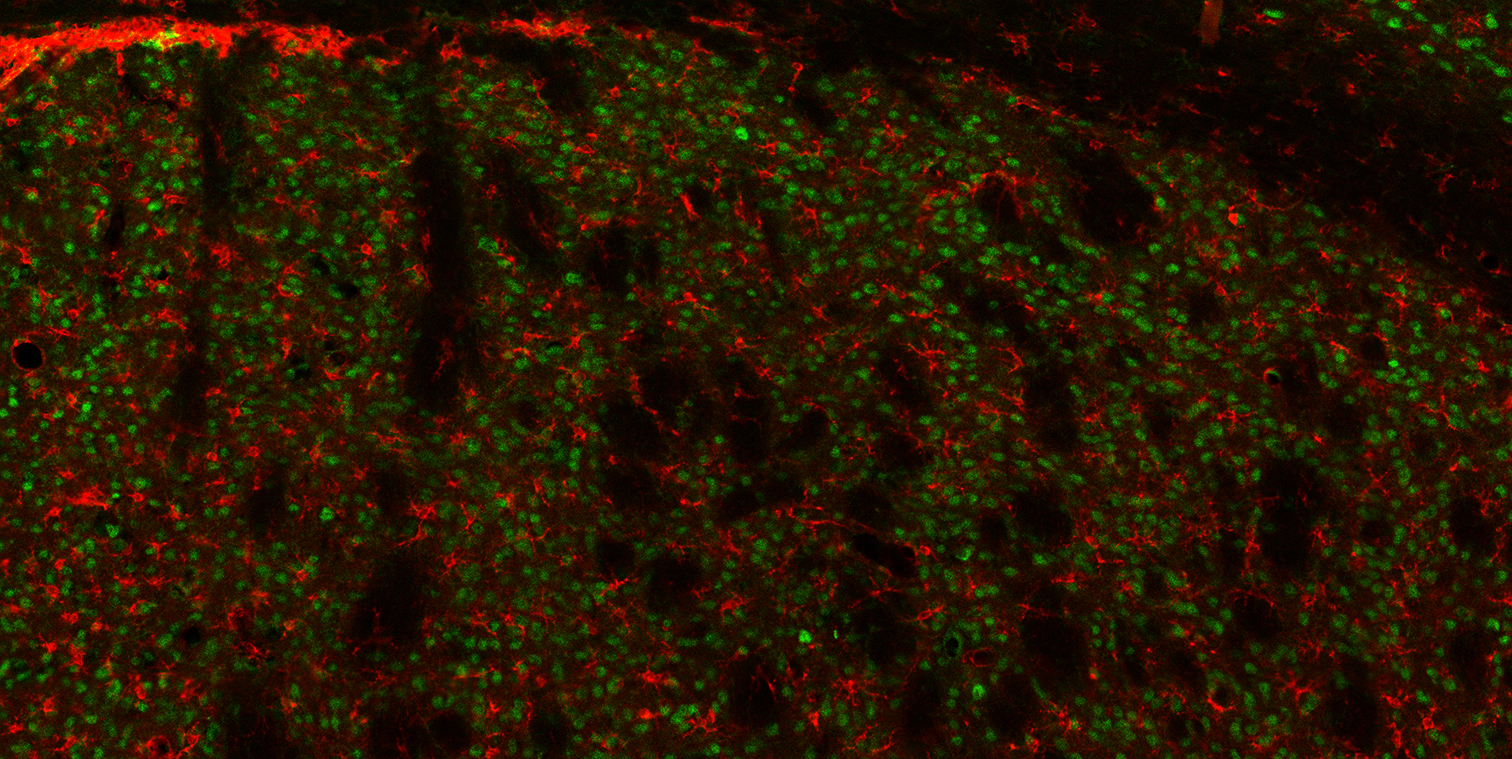

Supplement: Figure 3—source data 2. [file elife-75636-fig3-data2.zip › Fig3 source data 2 for Fig3 C/AAV-shptb STR #20 NeuN+HA.jpg]

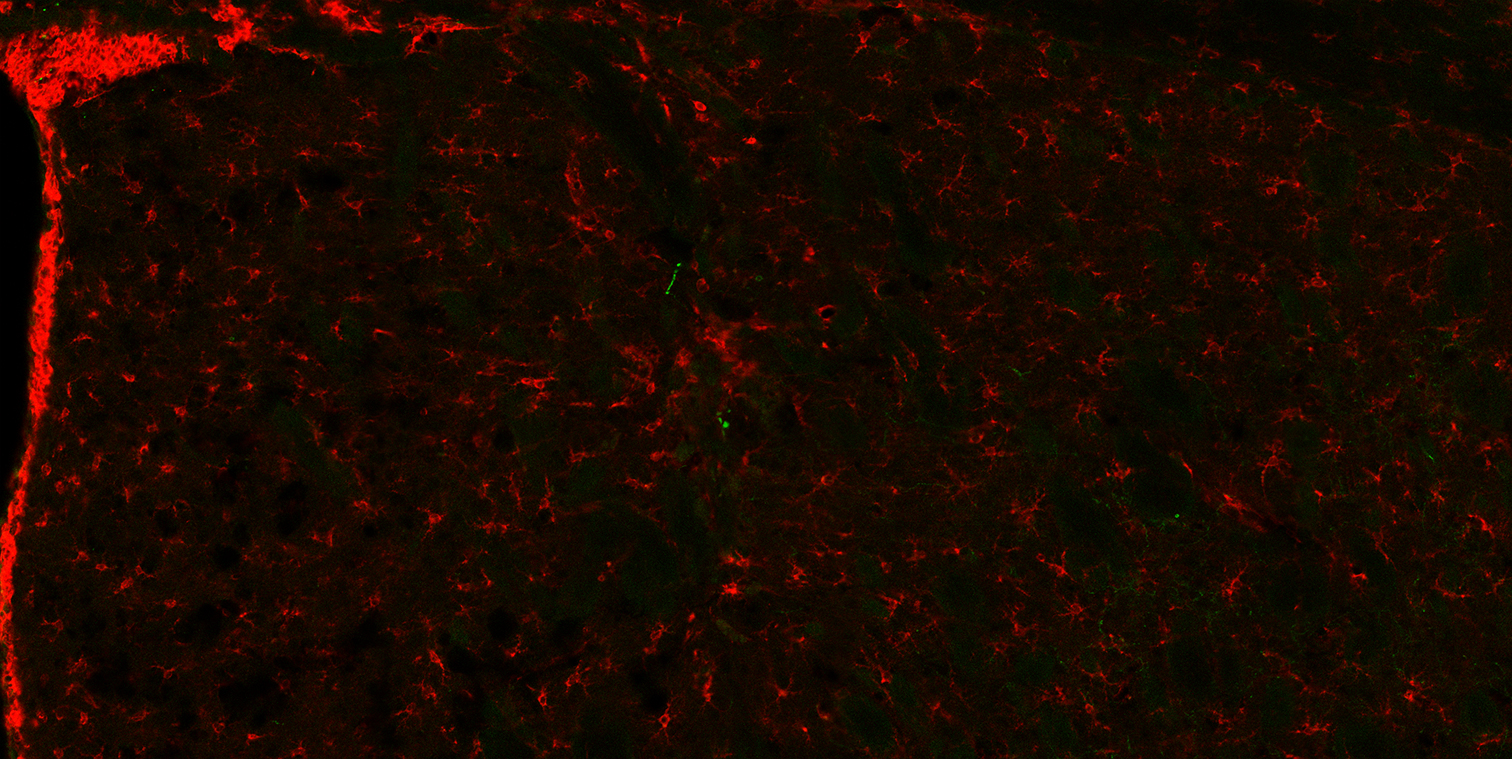

Supplement: Figure 3—source data 2. [file elife-75636-fig3-data2.zip › Fig3 source data 2 for Fig3 C/AAV-shptb STR #20 TH+HA.jpg]

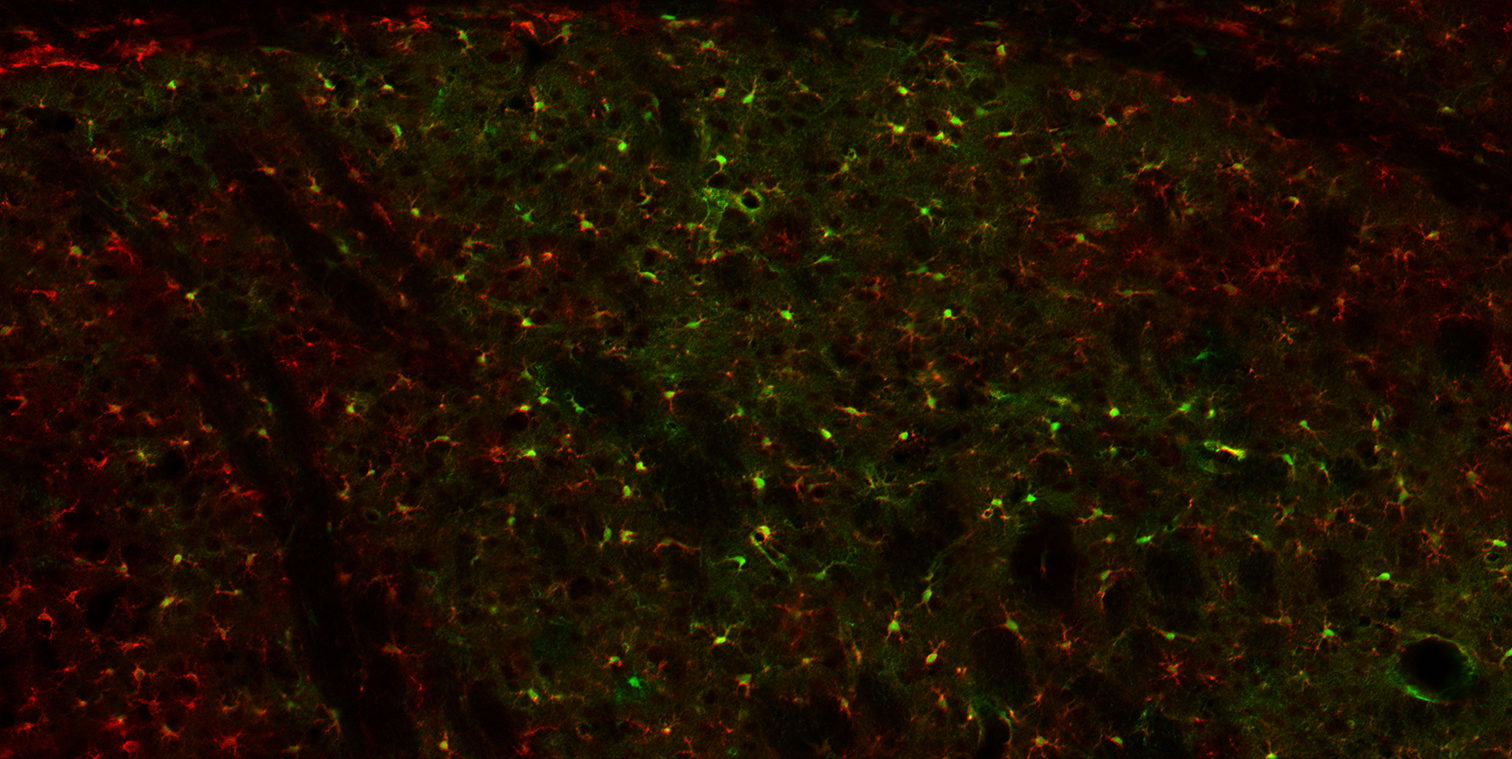

Supplement: Figure 3—source data 2. [file elife-75636-fig3-data2.zip › Fig3 source data 2 for Fig3 C/AAV-shptb STR #21 GFP+HA-1.jpg]

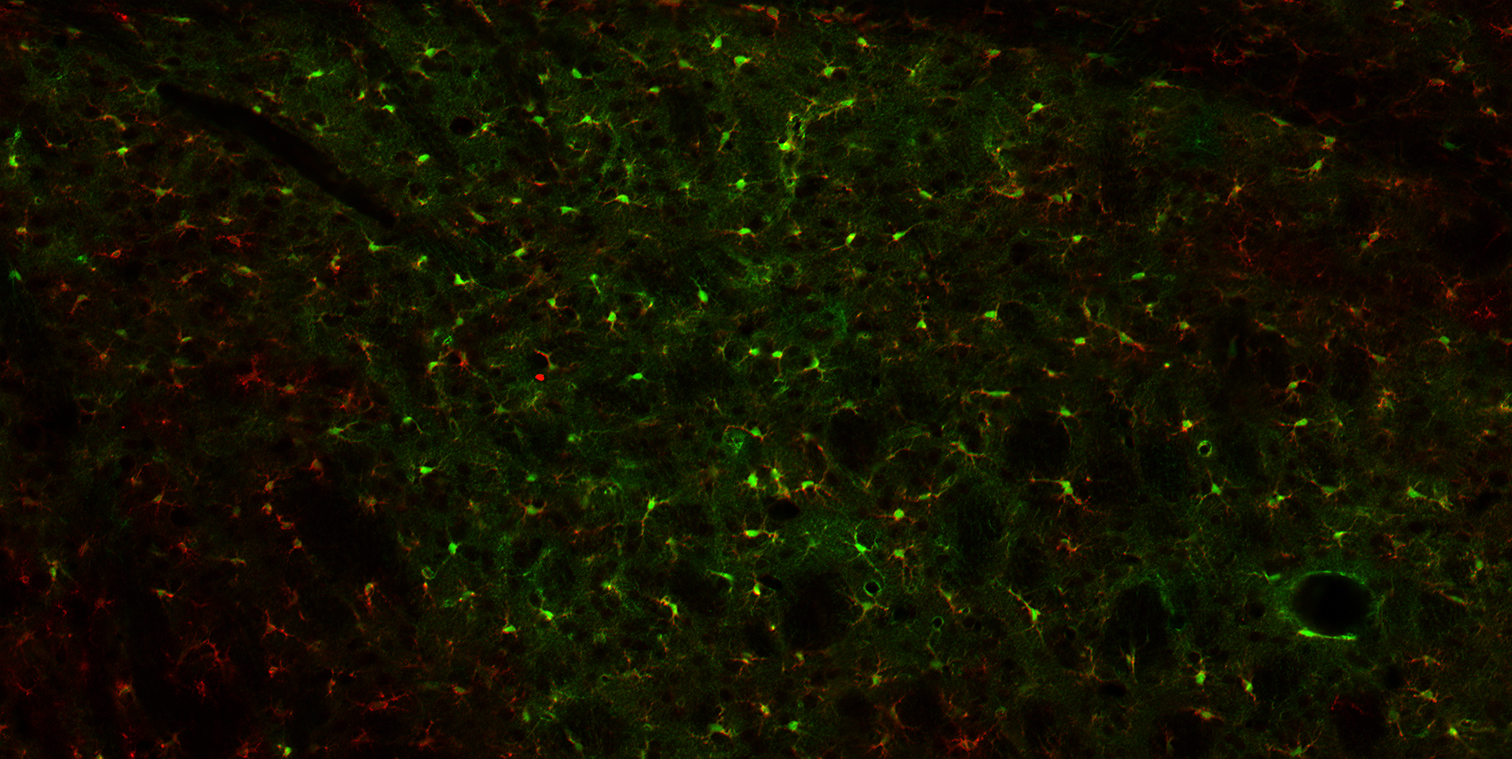

Supplement: Figure 3—source data 2. [file elife-75636-fig3-data2.zip › Fig3 source data 2 for Fig3 C/AAV-shptb STR #21 GFP+HA-2.jpg]

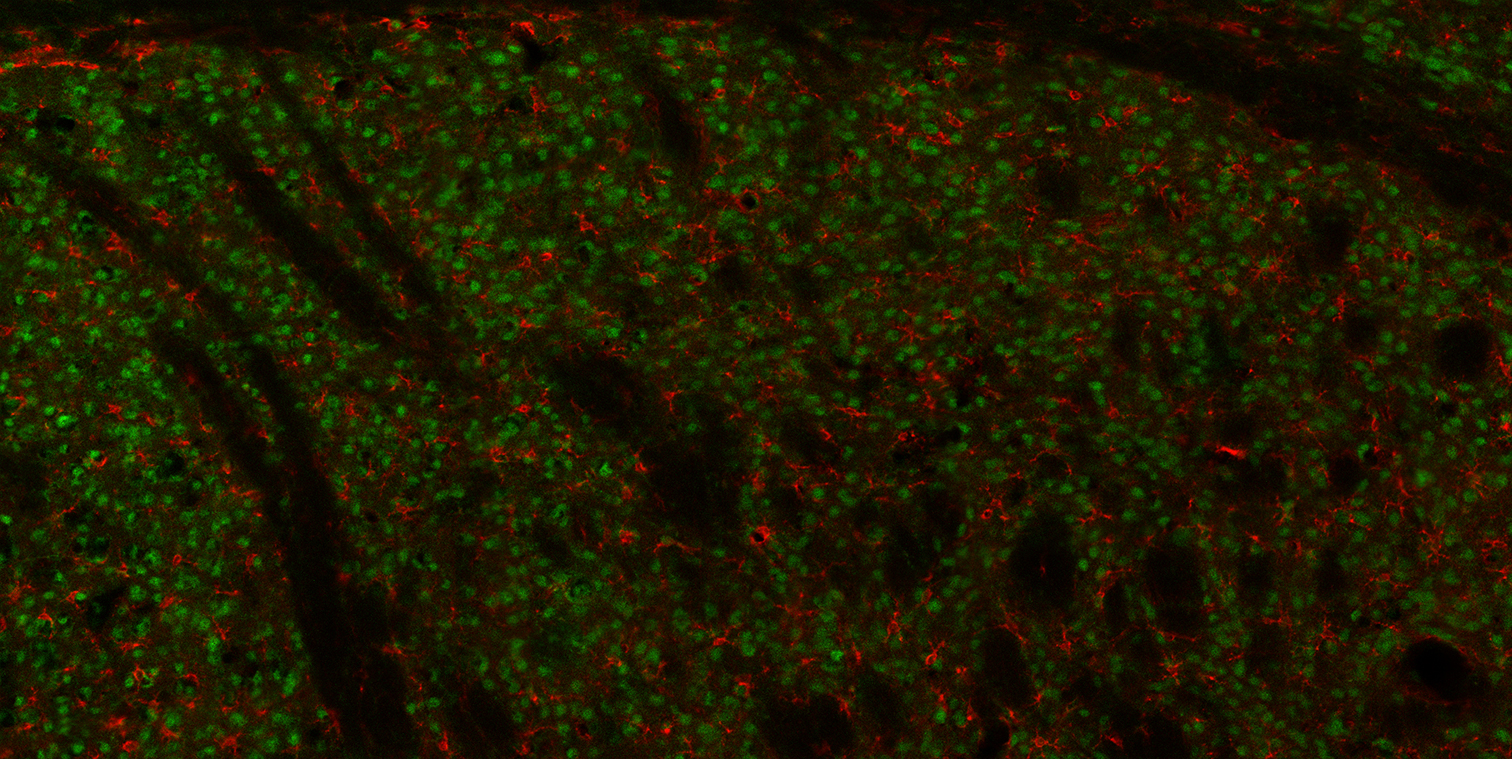

Supplement: Figure 3—source data 2. [file elife-75636-fig3-data2.zip › Fig3 source data 2 for Fig3 C/AAV-shptb STR #21 NeuN+HA.jpg]

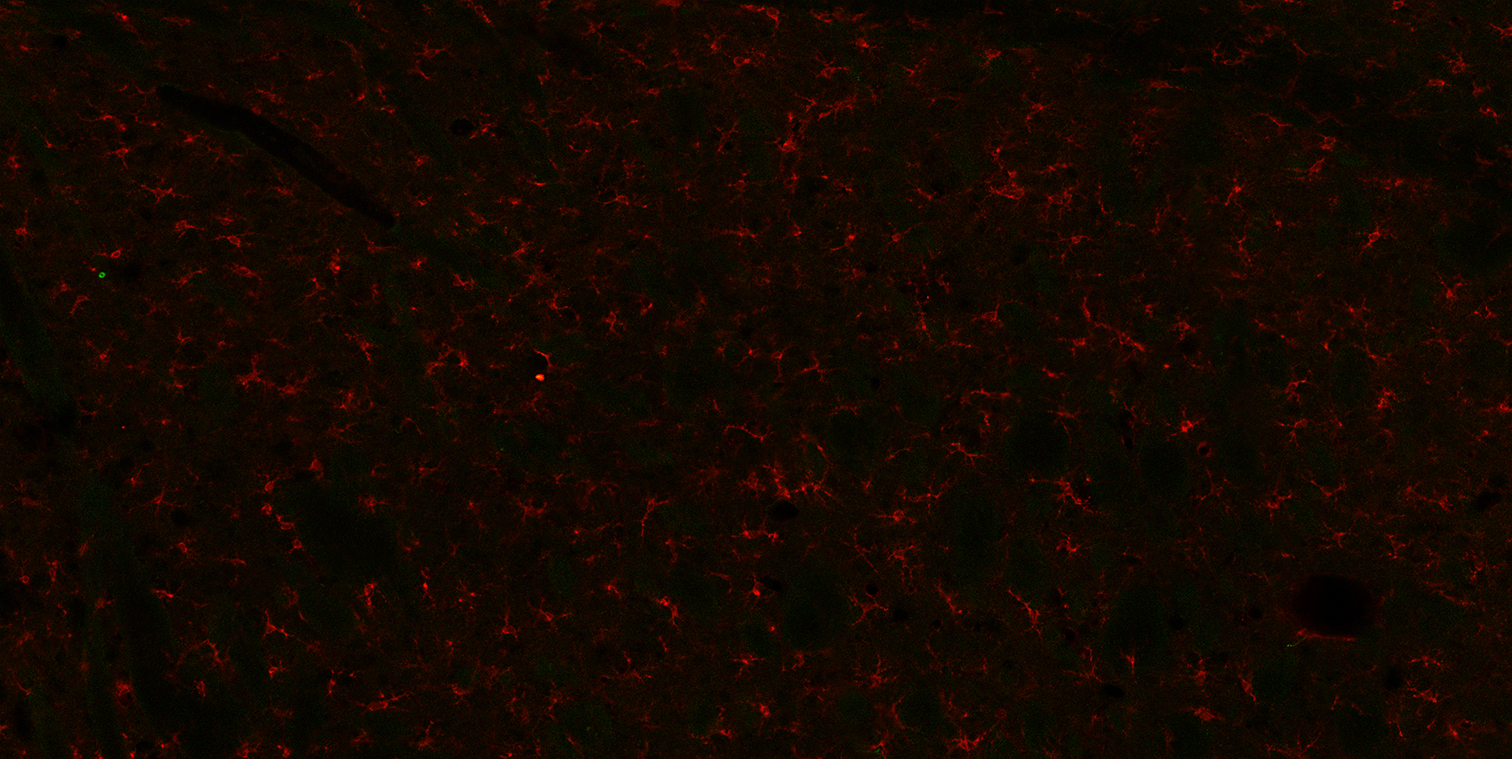

Supplement: Figure 3—source data 2. [file elife-75636-fig3-data2.zip › Fig3 source data 2 for Fig3 C/AAV-shptb STR #21 TH+HA.jpg]

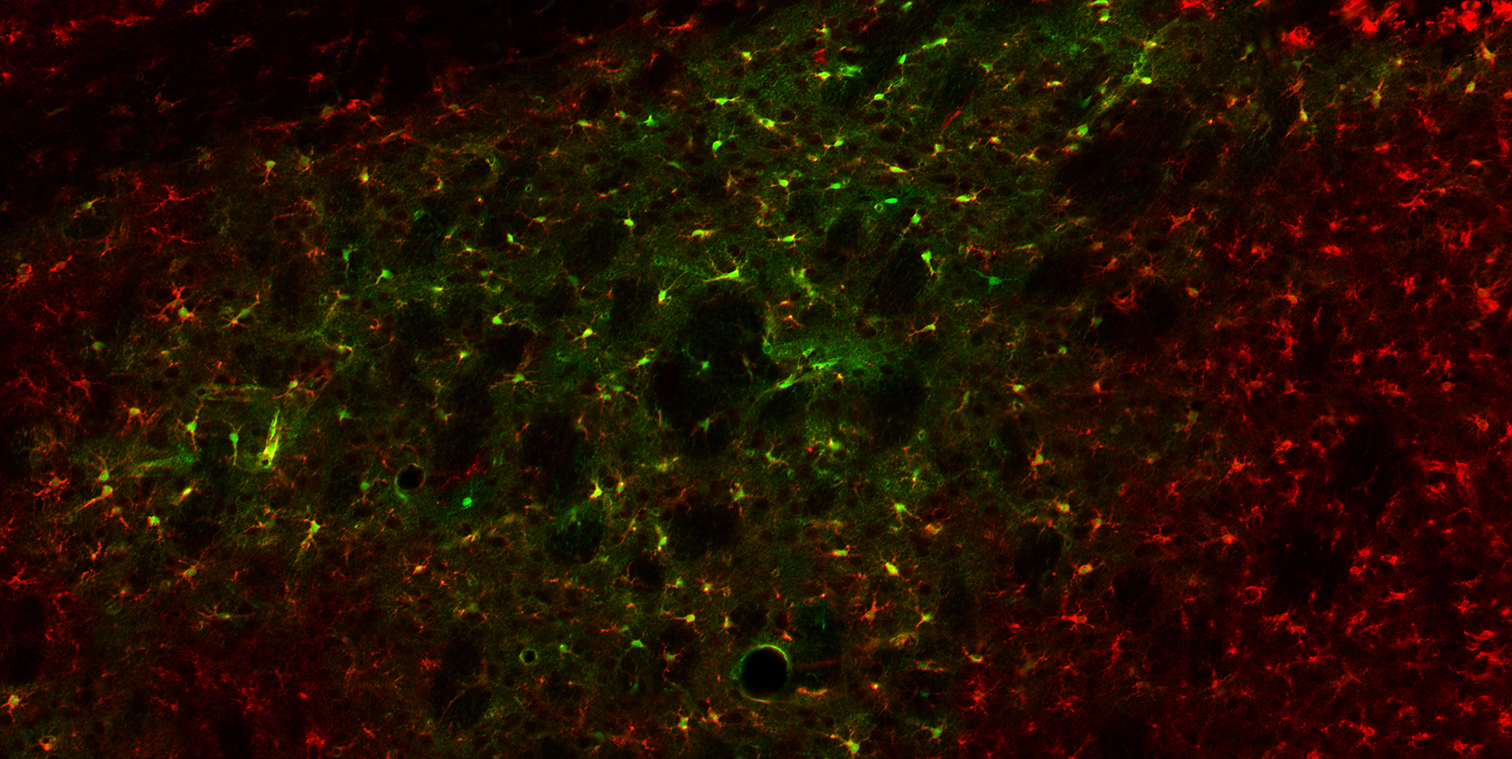

Supplement: Figure 3—source data 2. [file elife-75636-fig3-data2.zip › Fig3 source data 2 for Fig3 C/AAV-shptb STR #24 GFP+HA-1.jpg]

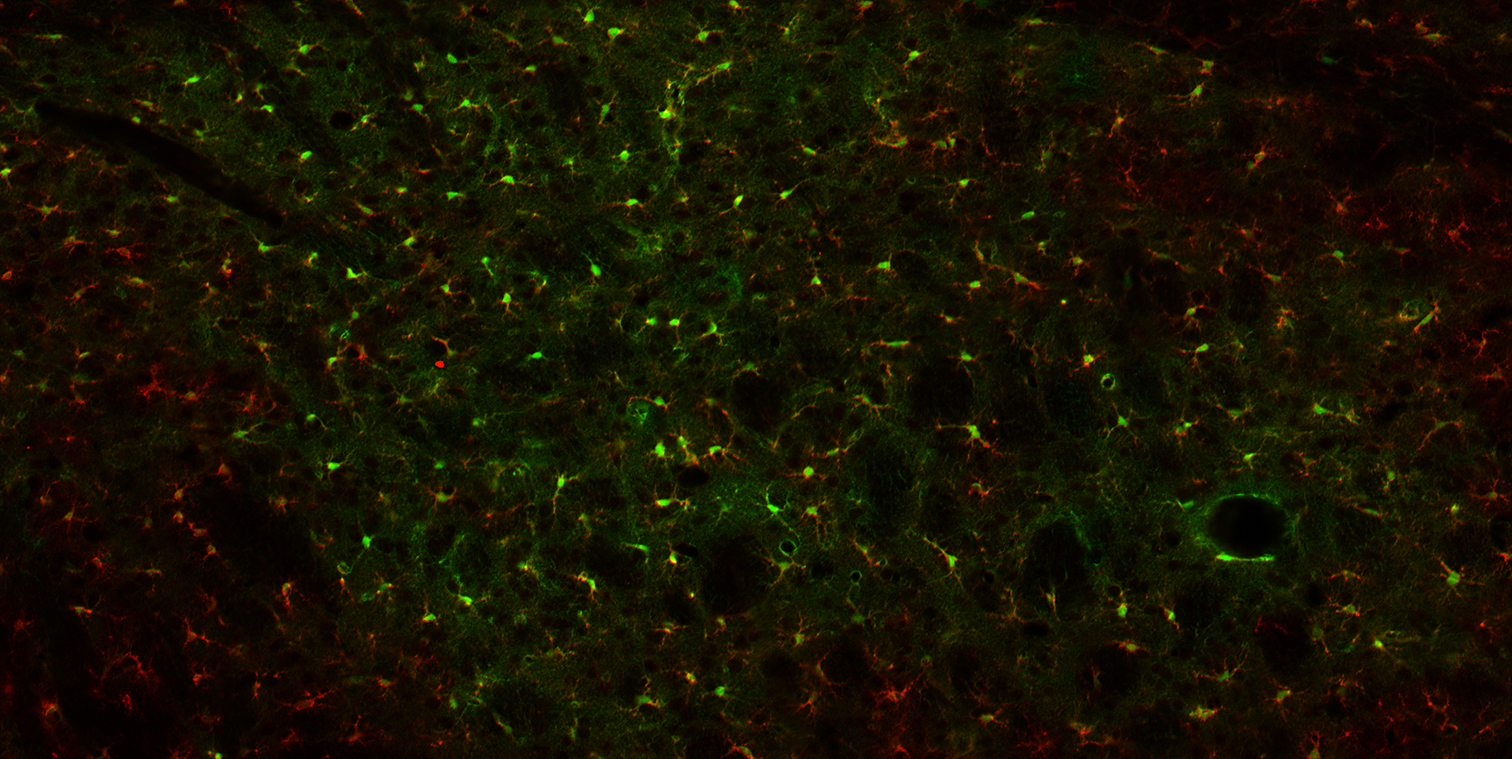

Supplement: Figure 3—source data 2. [file elife-75636-fig3-data2.zip › Fig3 source data 2 for Fig3 C/AAV-shptb STR #24 GFP+HA-2.jpg]

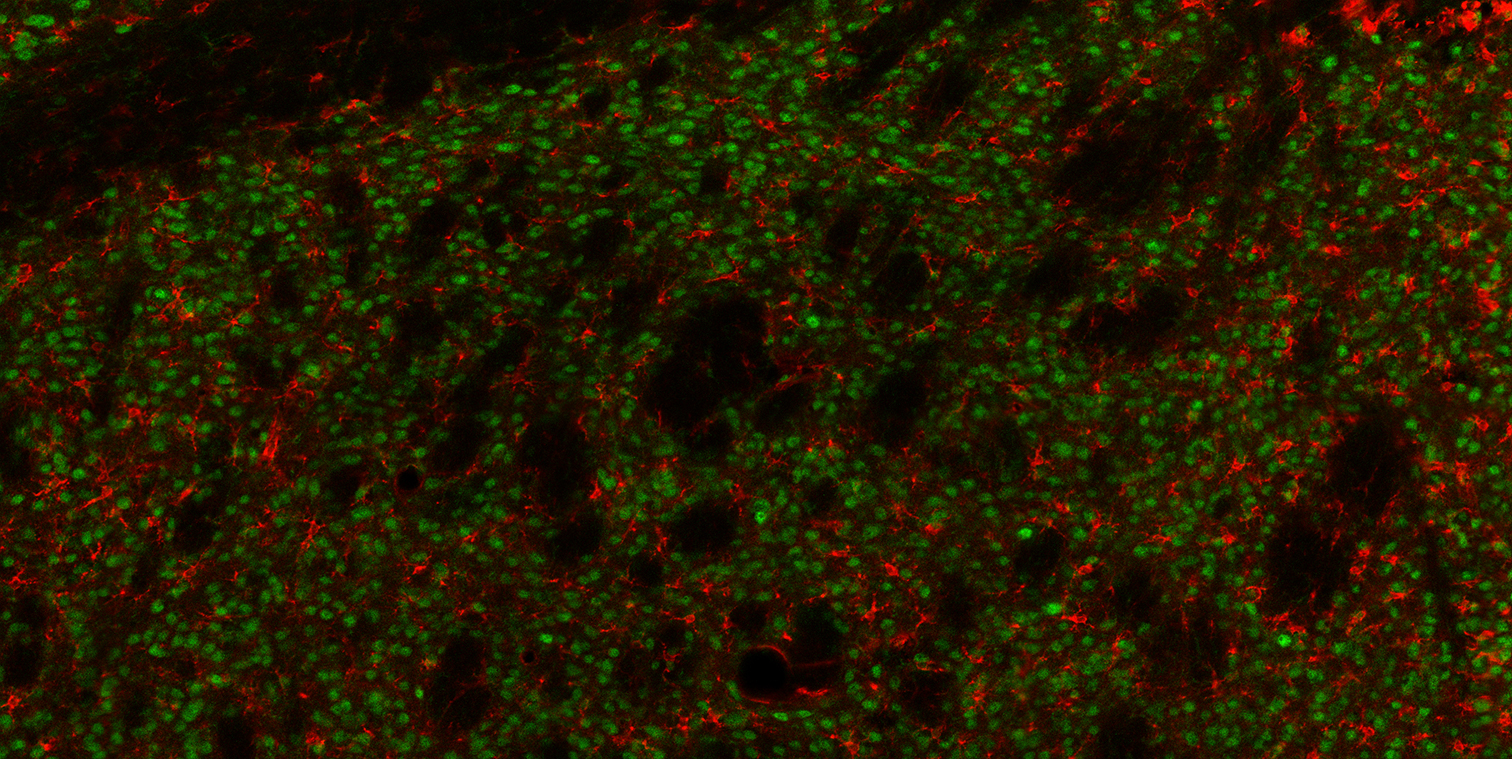

Supplement: Figure 3—source data 2. [file elife-75636-fig3-data2.zip › Fig3 source data 2 for Fig3 C/AAV-shptb STR #24 NeuN+HA.jpg]

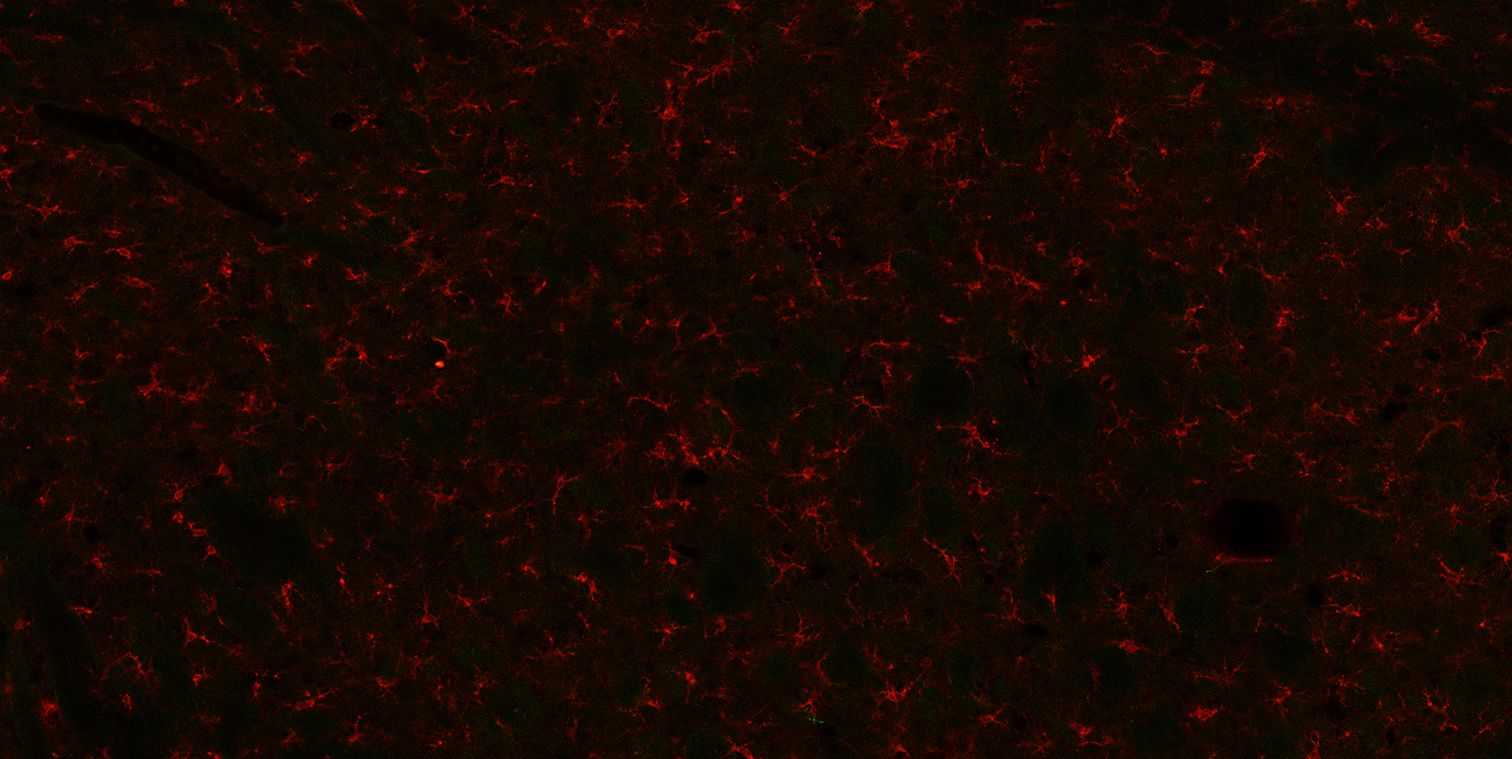

Supplement: Figure 3—source data 2. [file elife-75636-fig3-data2.zip › Fig3 source data 2 for Fig3 C/AAV-shptb STR #24 TH+HA.jpg]

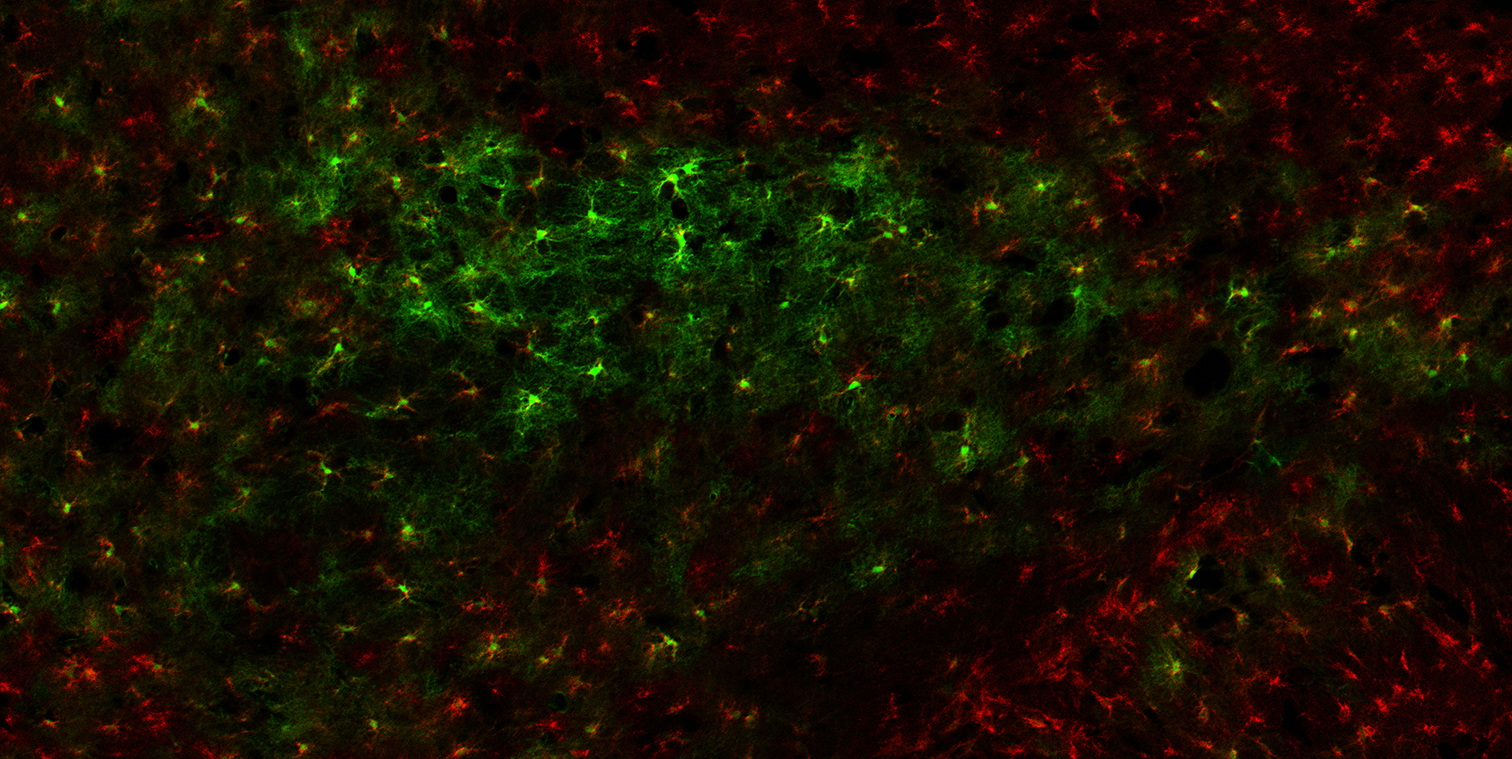

Supplement: Figure 3—source data 2. [file elife-75636-fig3-data2.zip › Fig3 source data 2 for Fig3 C/AAV-shscramble SN #10 GFP+HA-1.jpg]

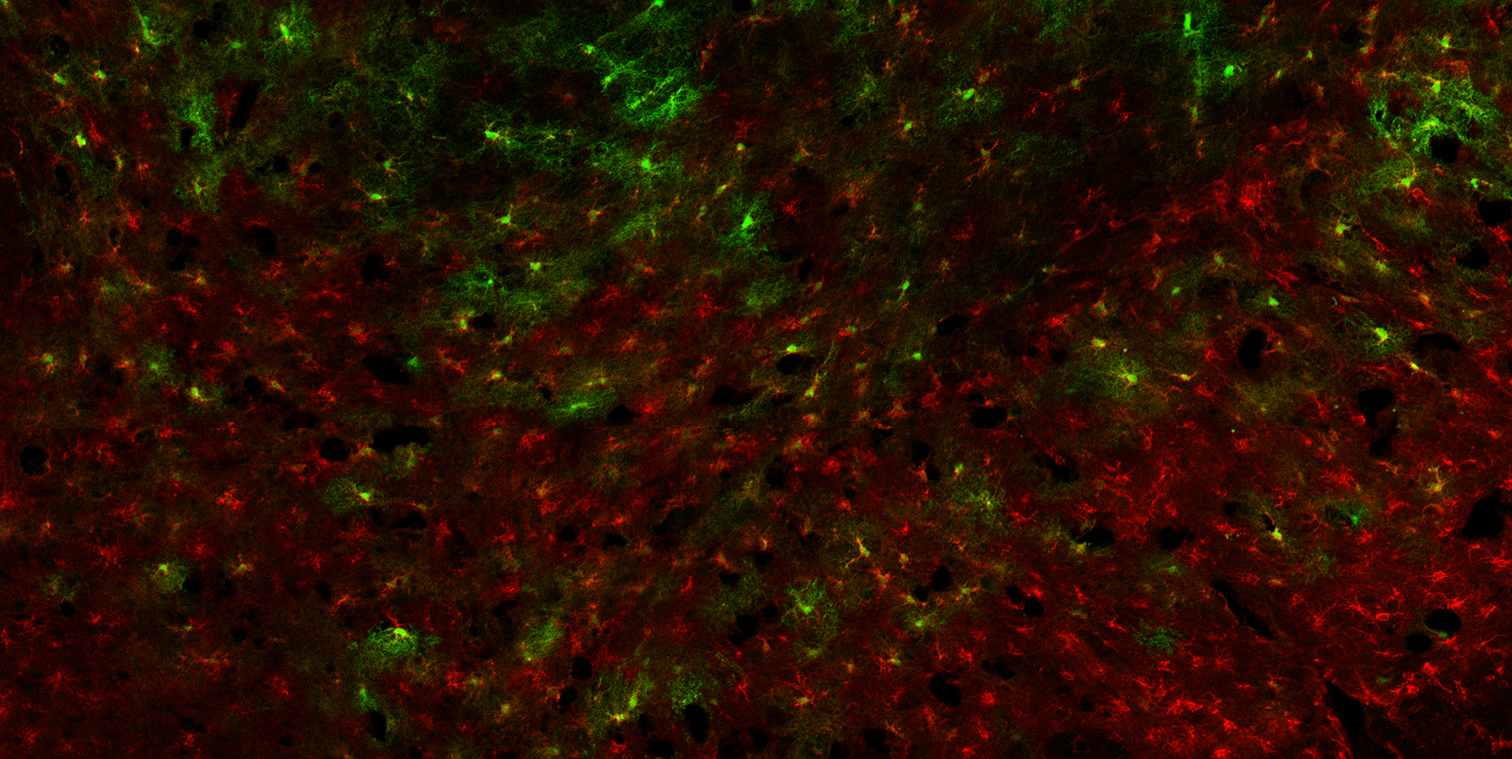

Supplement: Figure 3—source data 2. [file elife-75636-fig3-data2.zip › Fig3 source data 2 for Fig3 C/AAV-shscramble SN #10 GFP+HA-2.jpg]

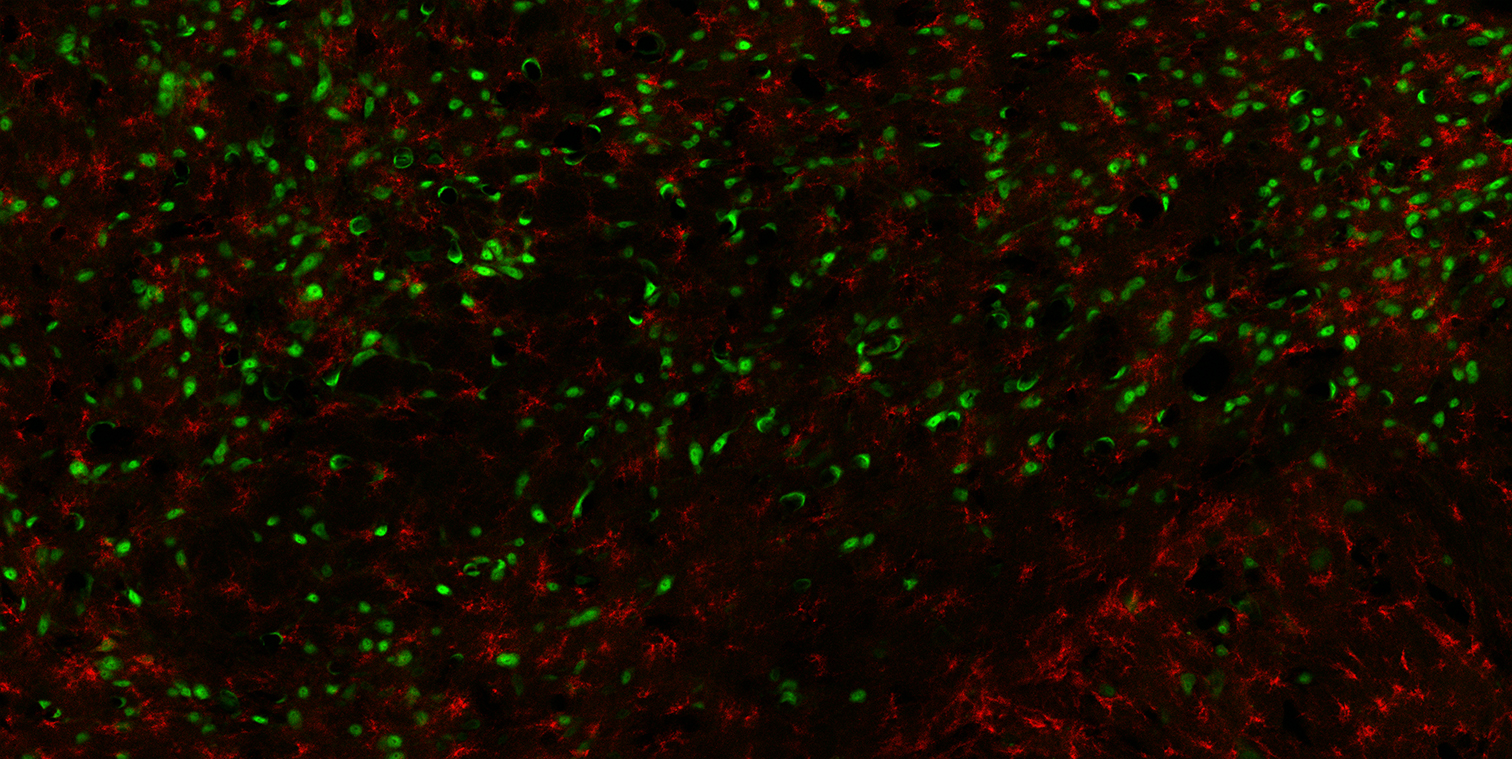

Supplement: Figure 3—source data 2. [file elife-75636-fig3-data2.zip › Fig3 source data 2 for Fig3 C/AAV-shscramble SN #10 NeuN+HA.jpg]

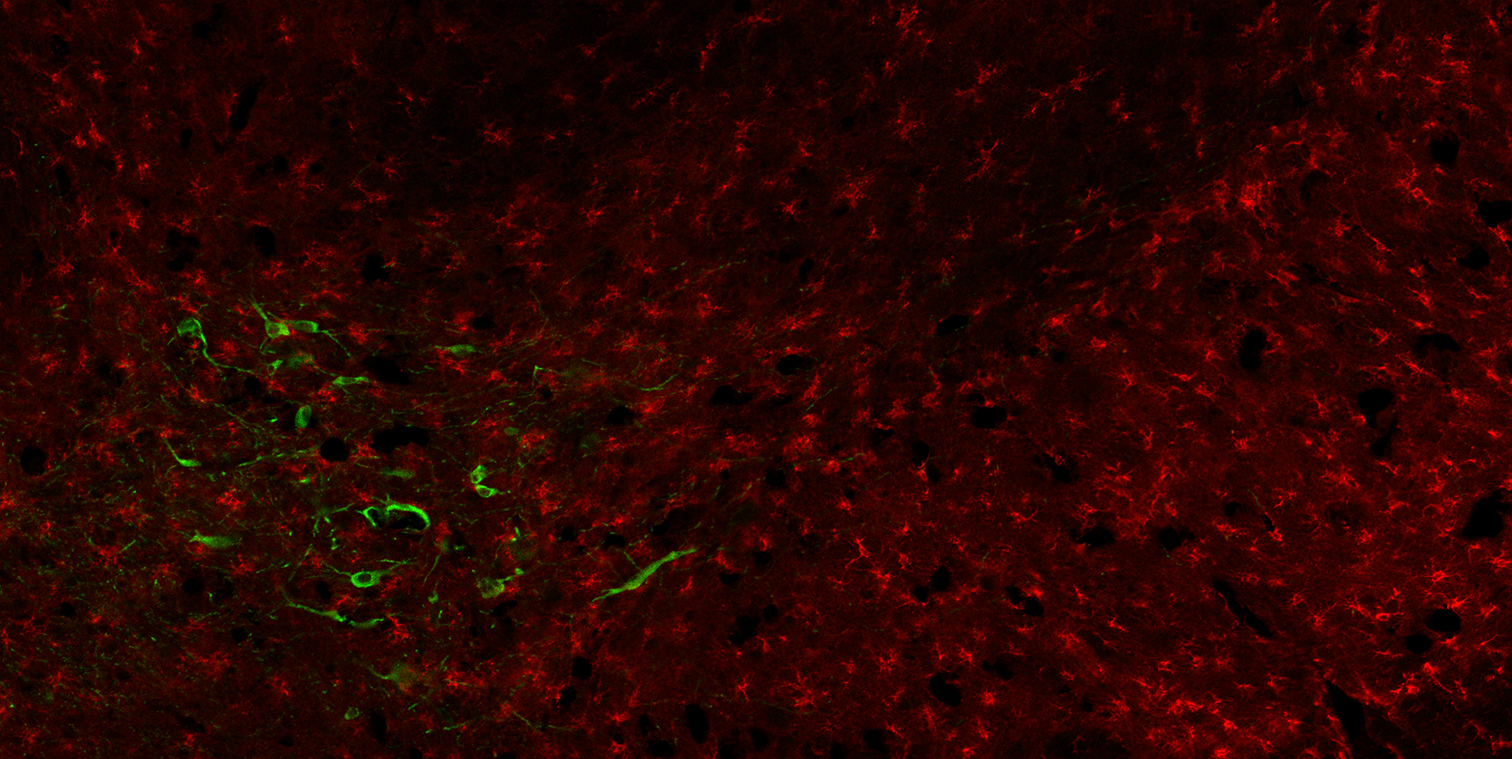

Supplement: Figure 3—source data 2. [file elife-75636-fig3-data2.zip › Fig3 source data 2 for Fig3 C/AAV-shscramble SN #10 TH+HA.jpg]

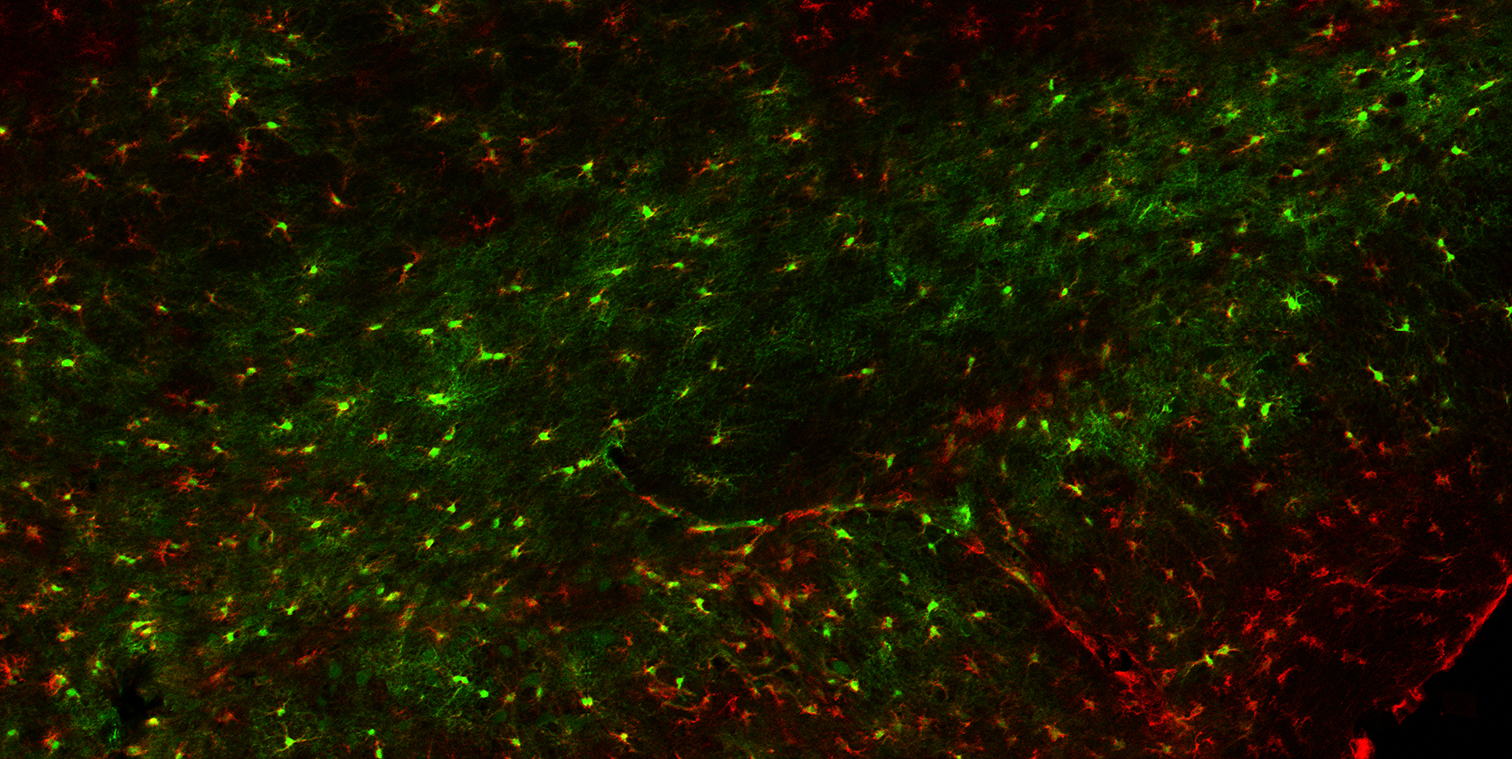

Supplement: Figure 3—source data 2. [file elife-75636-fig3-data2.zip › Fig3 source data 2 for Fig3 C/AAV-shscramble SN #24 GFP+HA-1.jpg]

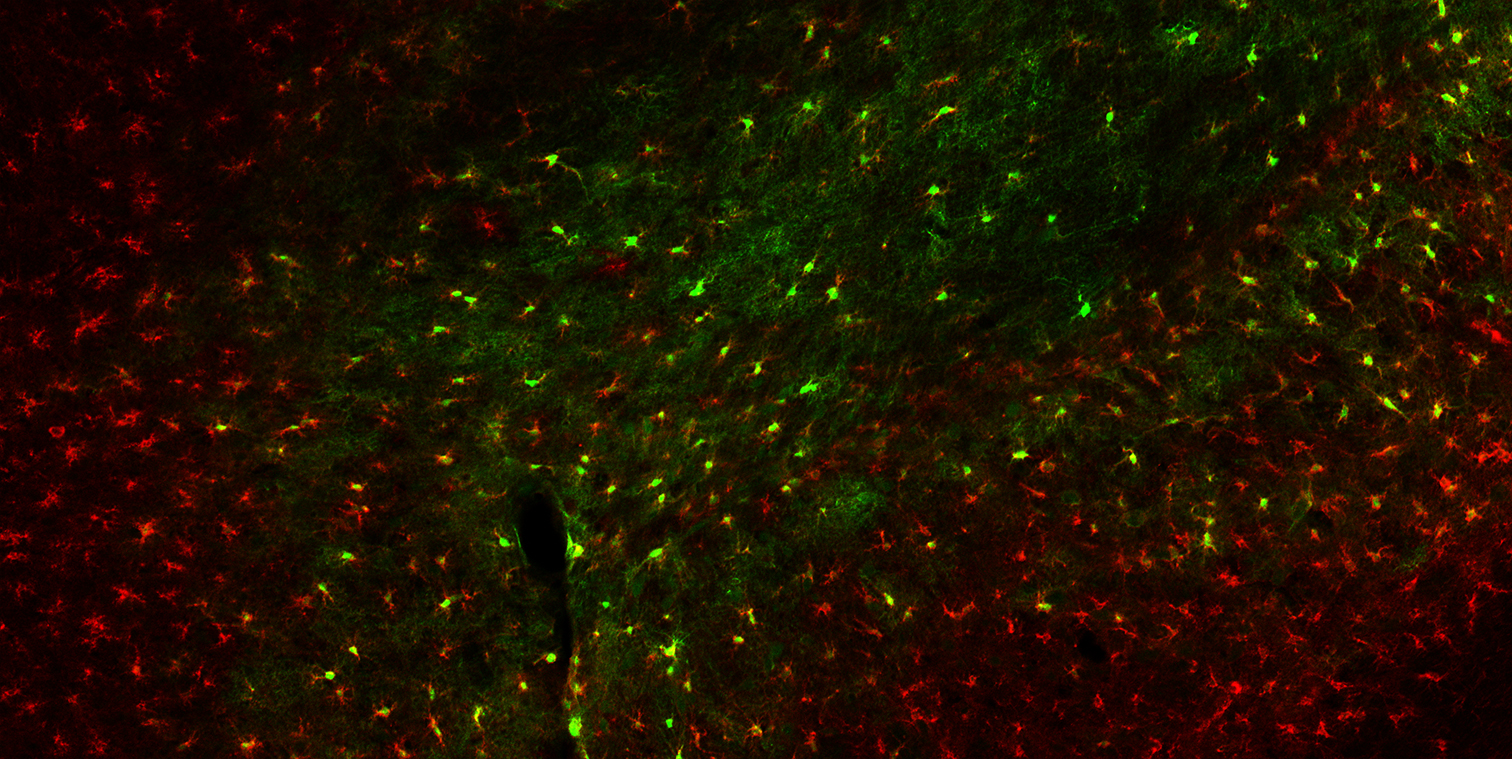

Supplement: Figure 3—source data 2. [file elife-75636-fig3-data2.zip › Fig3 source data 2 for Fig3 C/AAV-shscramble SN #24 GFP+HA-2.jpg]

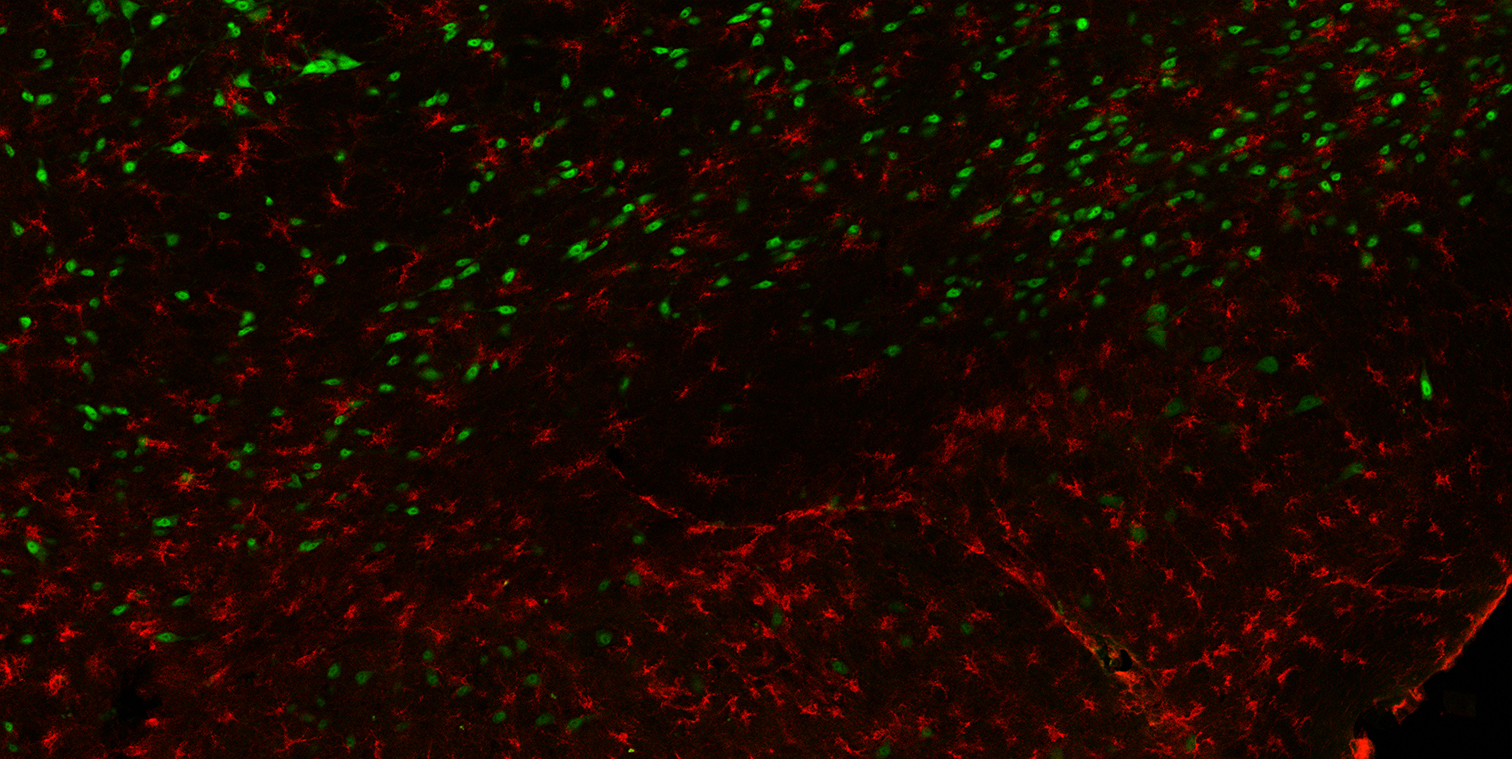

Supplement: Figure 3—source data 2. [file elife-75636-fig3-data2.zip › Fig3 source data 2 for Fig3 C/AAV-shscramble SN #24 NeuN+HA.jpg]

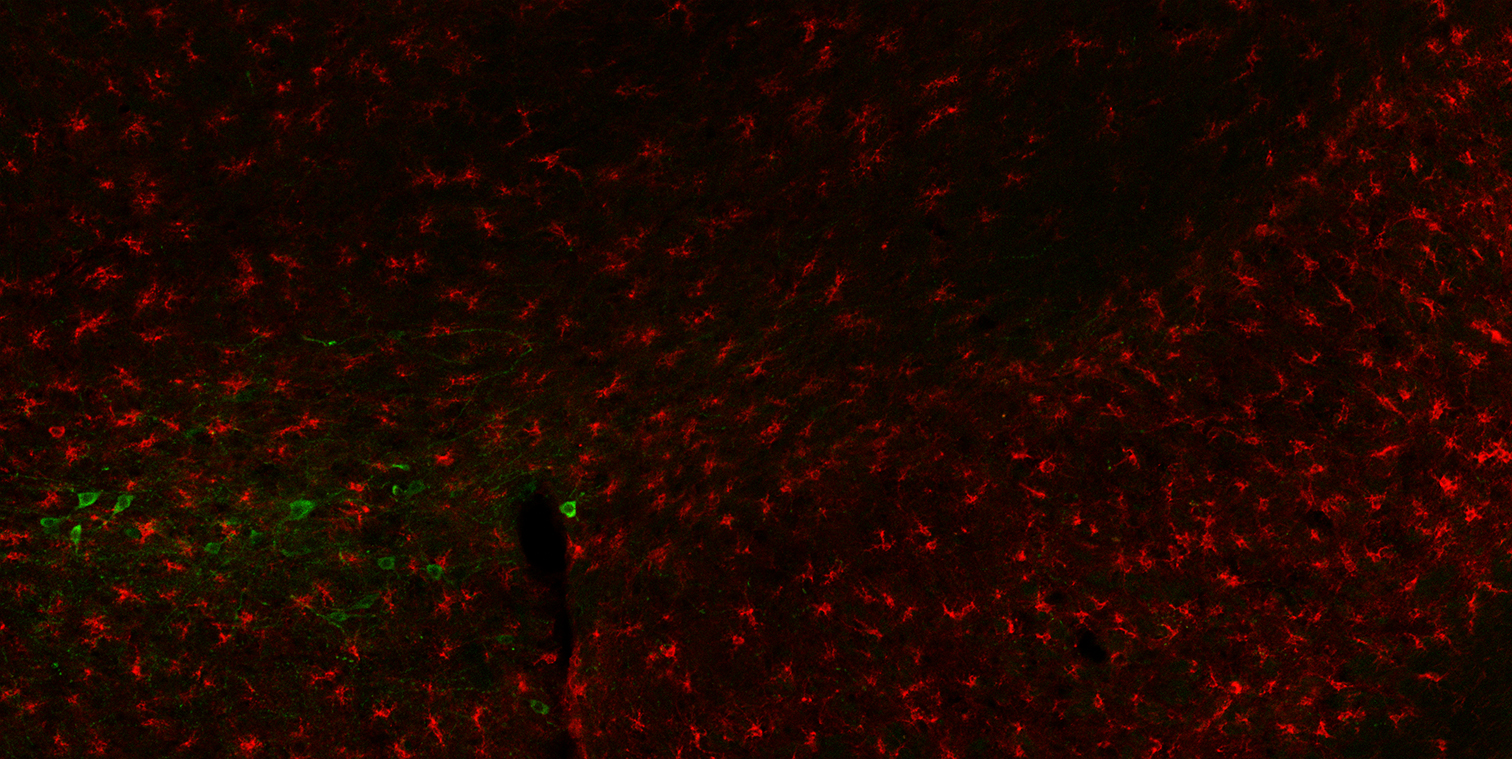

Supplement: Figure 3—source data 2. [file elife-75636-fig3-data2.zip › Fig3 source data 2 for Fig3 C/AAV-shscramble SN #24 TH+HA.jpg]

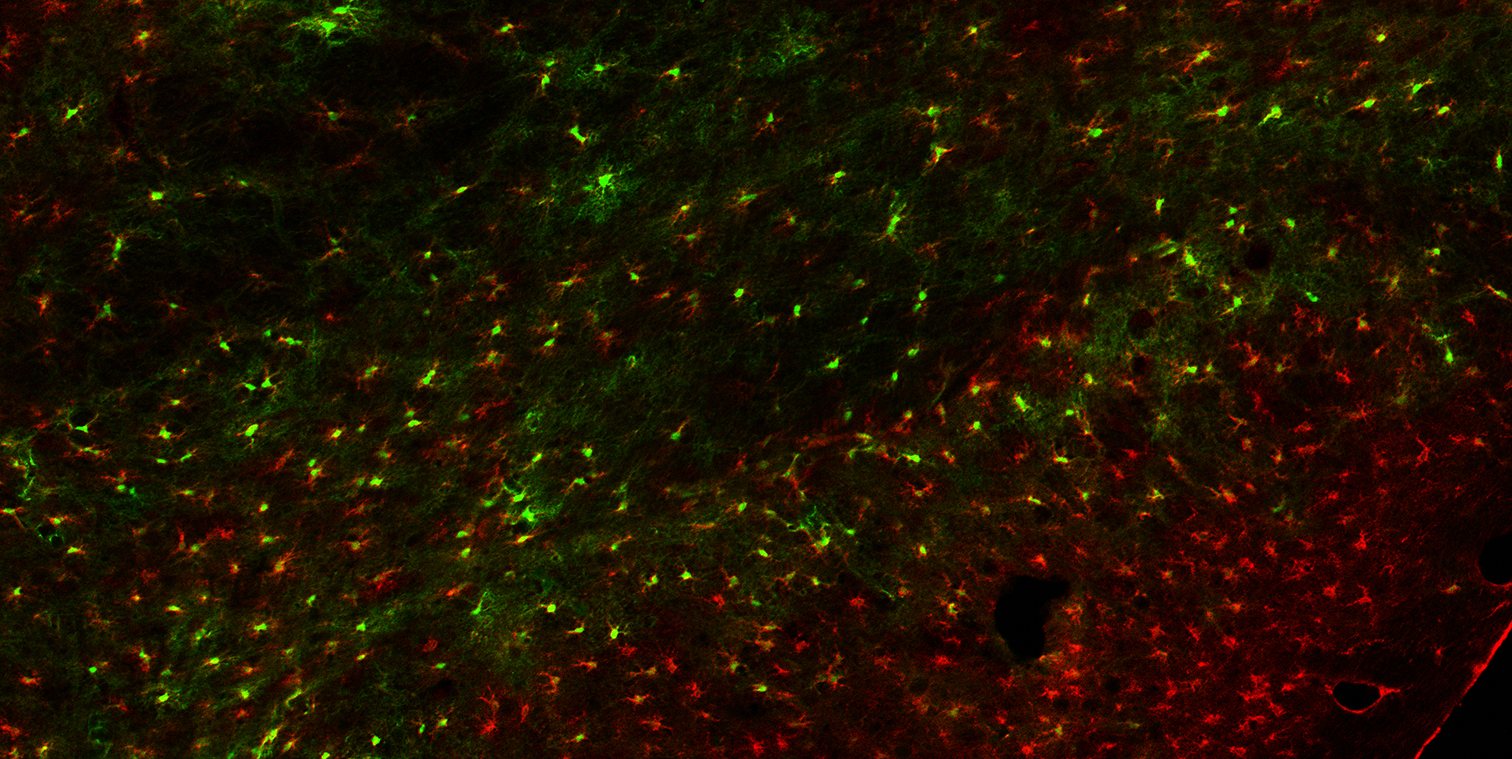

Supplement: Figure 3—source data 2. [file elife-75636-fig3-data2.zip › Fig3 source data 2 for Fig3 C/AAV-shscramble SN #26 GFP+HA-1.jpg]

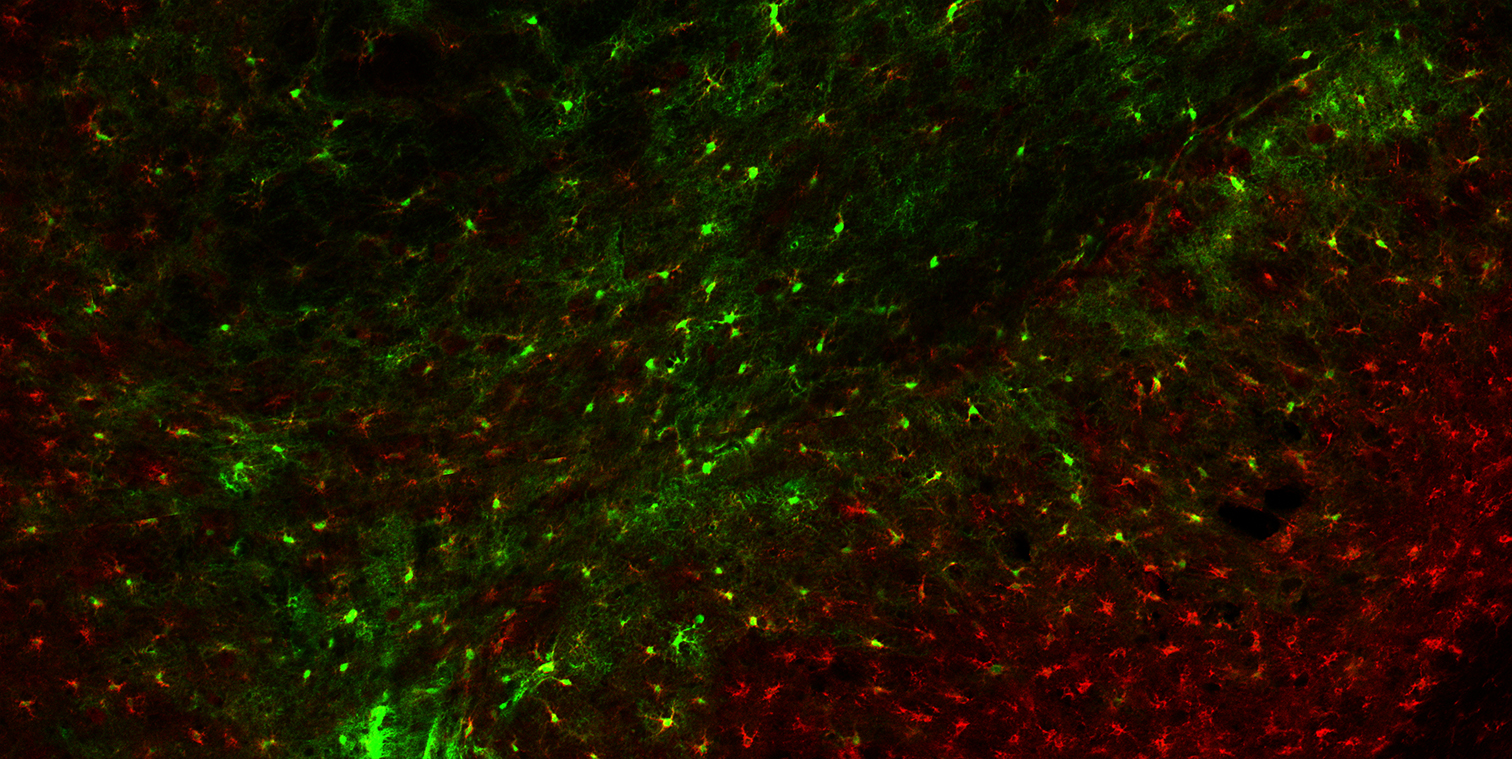

Supplement: Figure 3—source data 2. [file elife-75636-fig3-data2.zip › Fig3 source data 2 for Fig3 C/AAV-shscramble SN #26 GFP+HA-2.jpg]

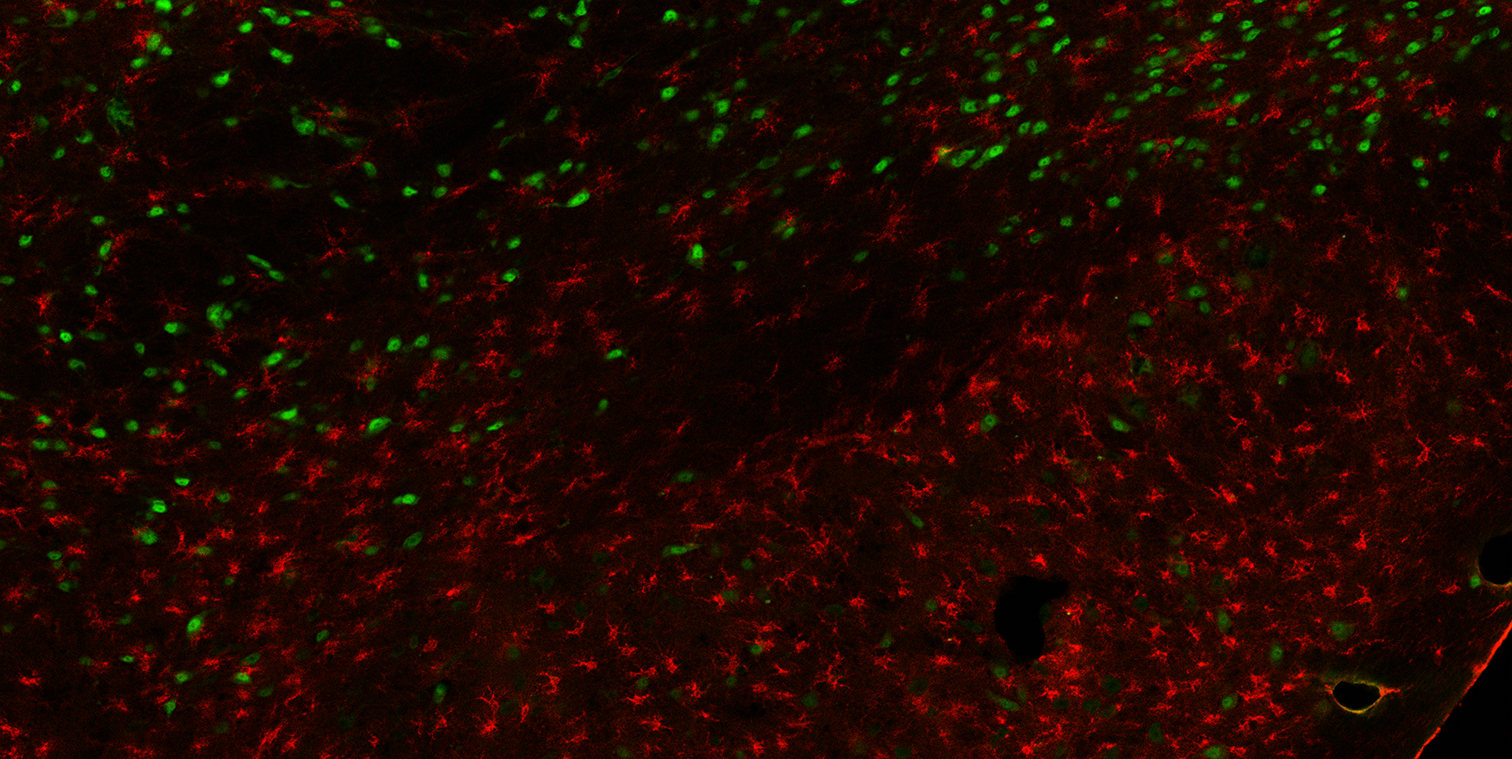

Supplement: Figure 3—source data 2. [file elife-75636-fig3-data2.zip › Fig3 source data 2 for Fig3 C/AAV-shscramble SN #26 NeuN+HA.jpg]

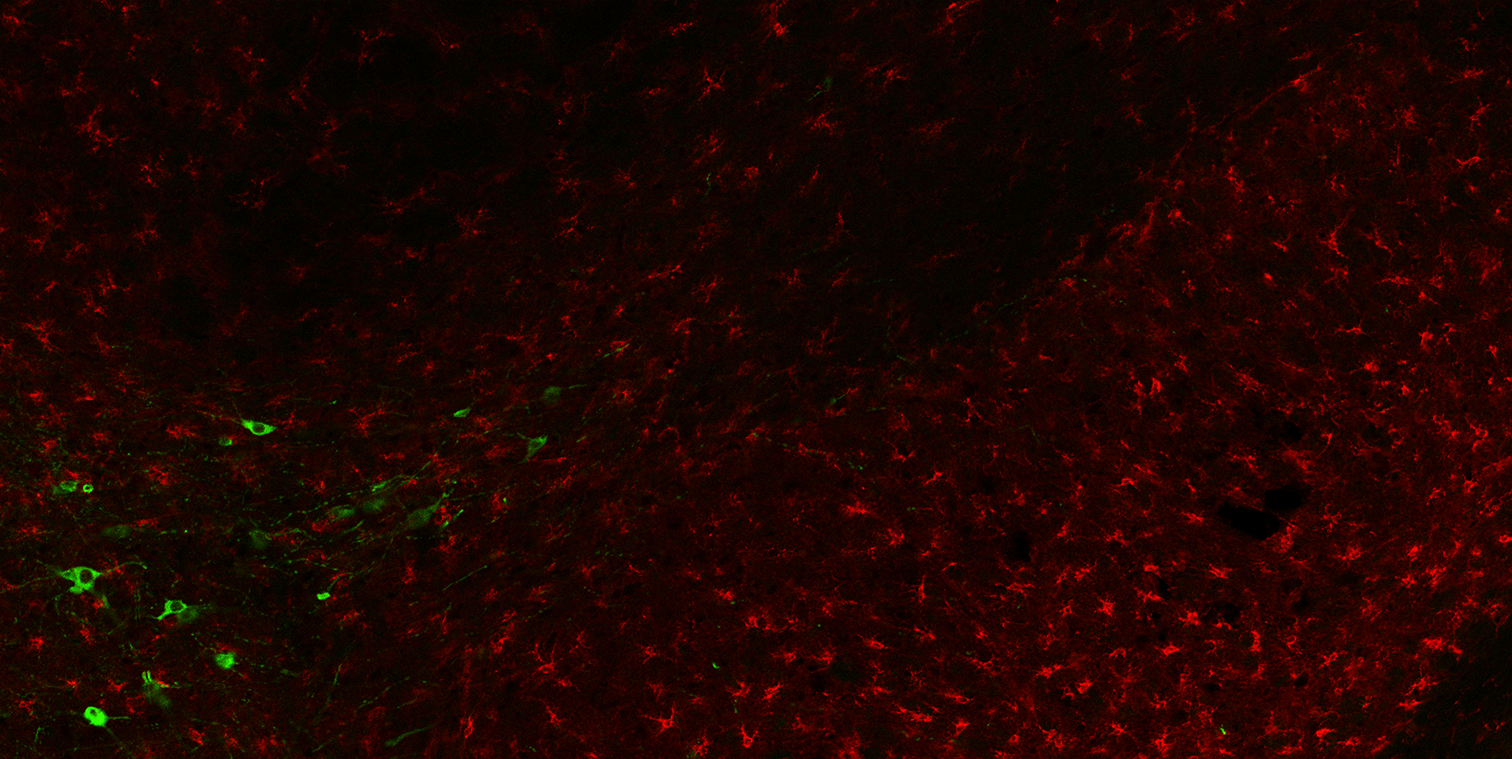

Supplement: Figure 3—source data 2. [file elife-75636-fig3-data2.zip › Fig3 source data 2 for Fig3 C/AAV-shscramble SN #26 TH+HA.jpg]

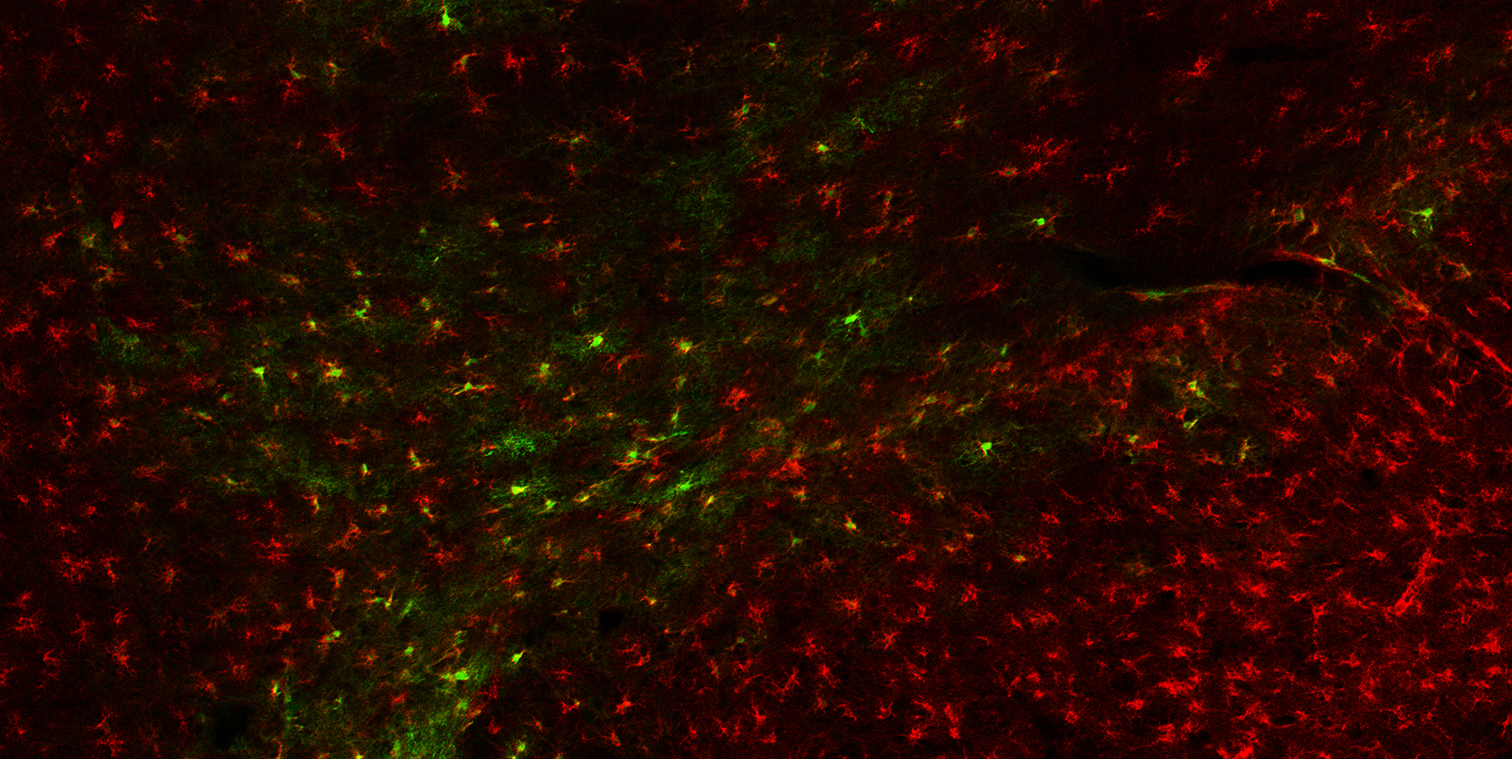

Supplement: Figure 3—source data 2. [file elife-75636-fig3-data2.zip › Fig3 source data 2 for Fig3 C/AAV-shscramble SN #28 GFP+HA-1.jpg]

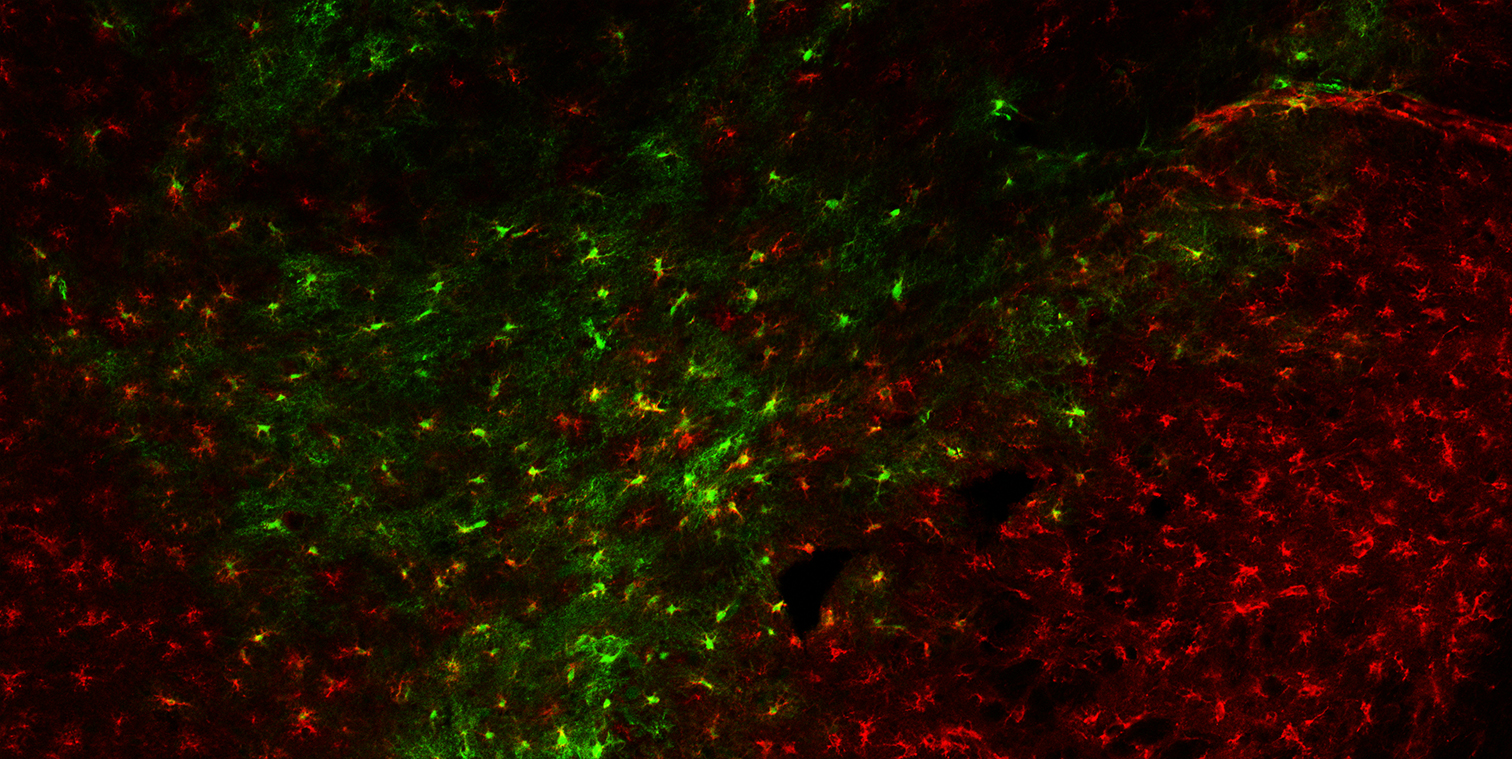

Supplement: Figure 3—source data 2. [file elife-75636-fig3-data2.zip › Fig3 source data 2 for Fig3 C/AAV-shscramble SN #28 GFP+HA-2.jpg]

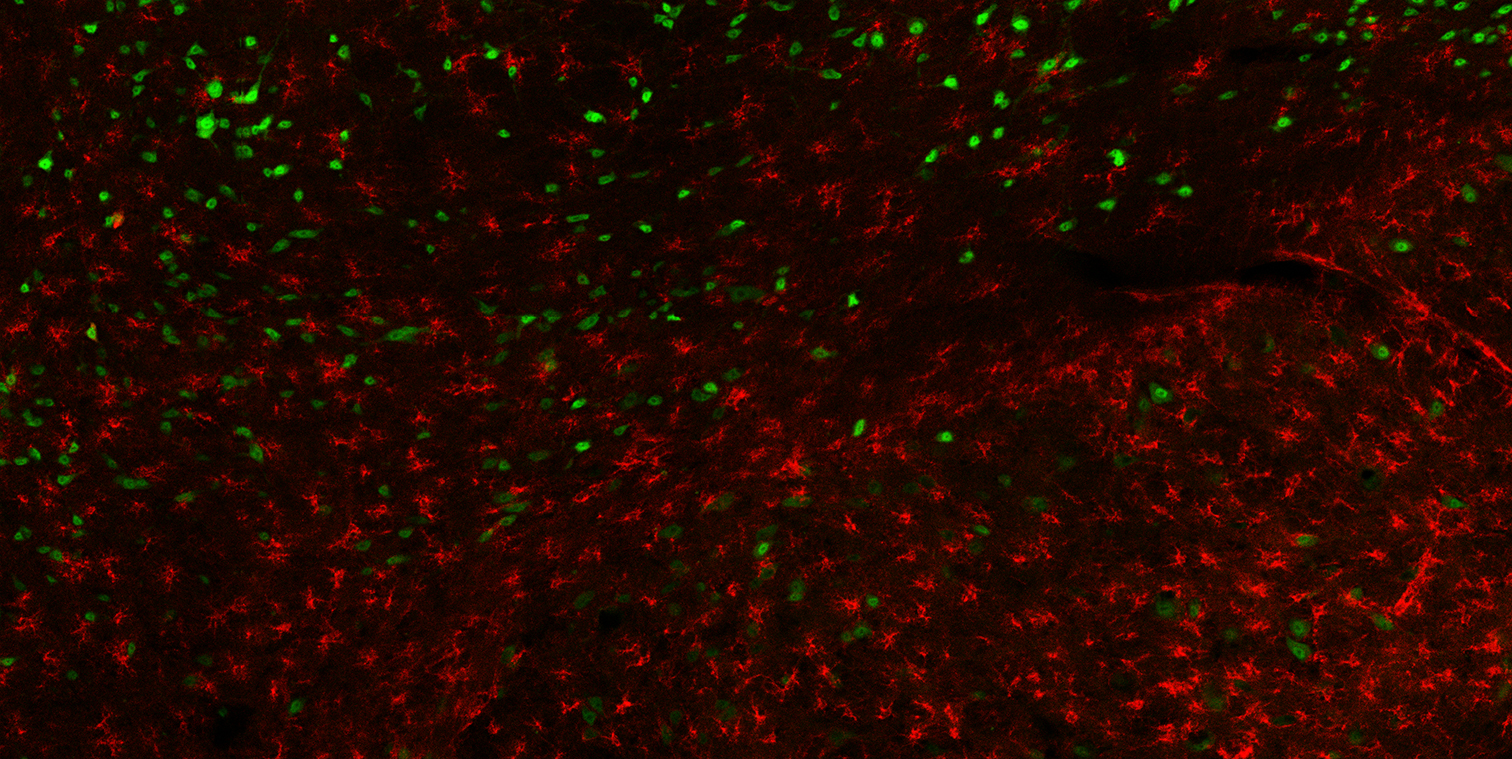

Supplement: Figure 3—source data 2. [file elife-75636-fig3-data2.zip › Fig3 source data 2 for Fig3 C/AAV-shscramble SN #28 NeuN+HA.jpg]

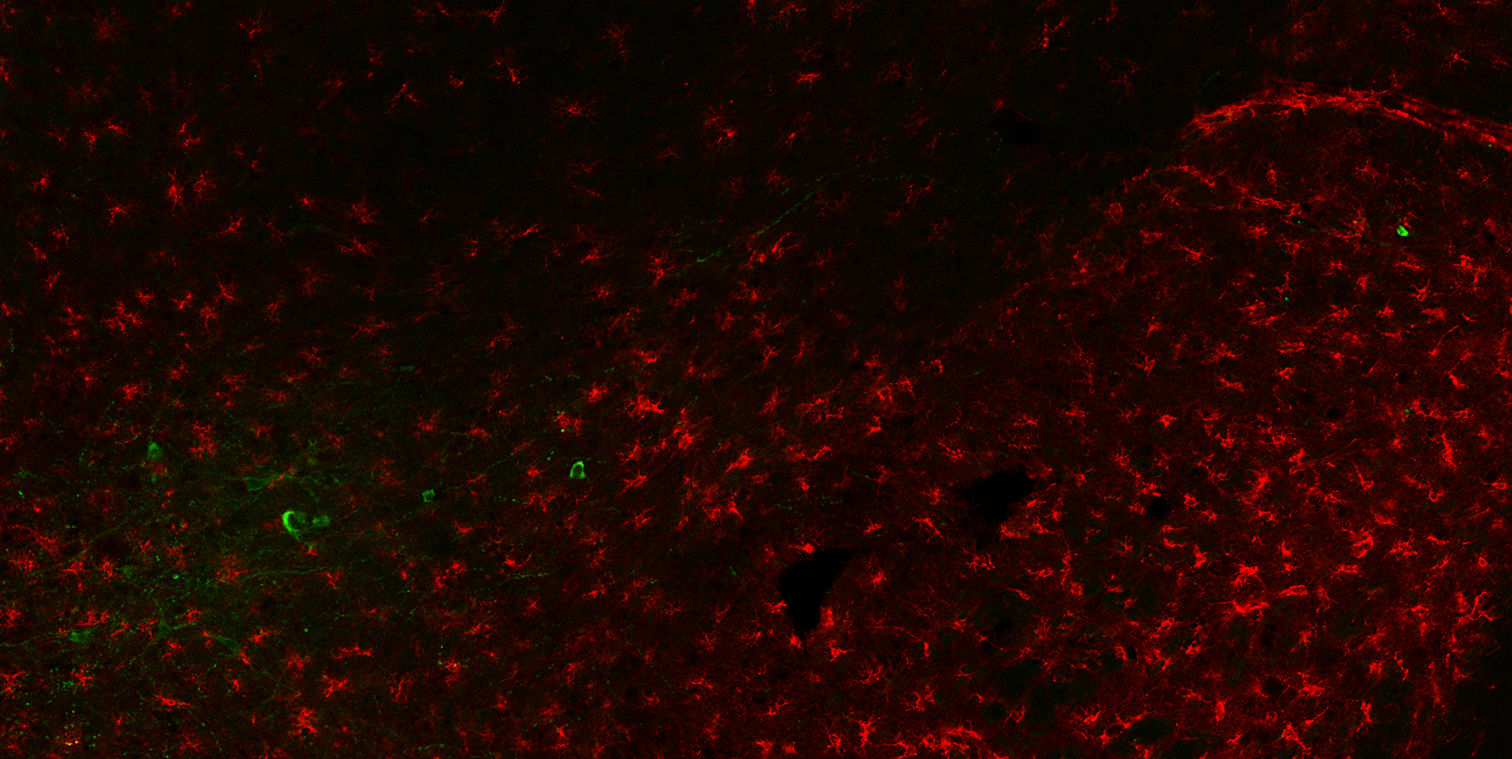

Supplement: Figure 3—source data 2. [file elife-75636-fig3-data2.zip › Fig3 source data 2 for Fig3 C/AAV-shscramble SN #28 TH+HA.jpg]

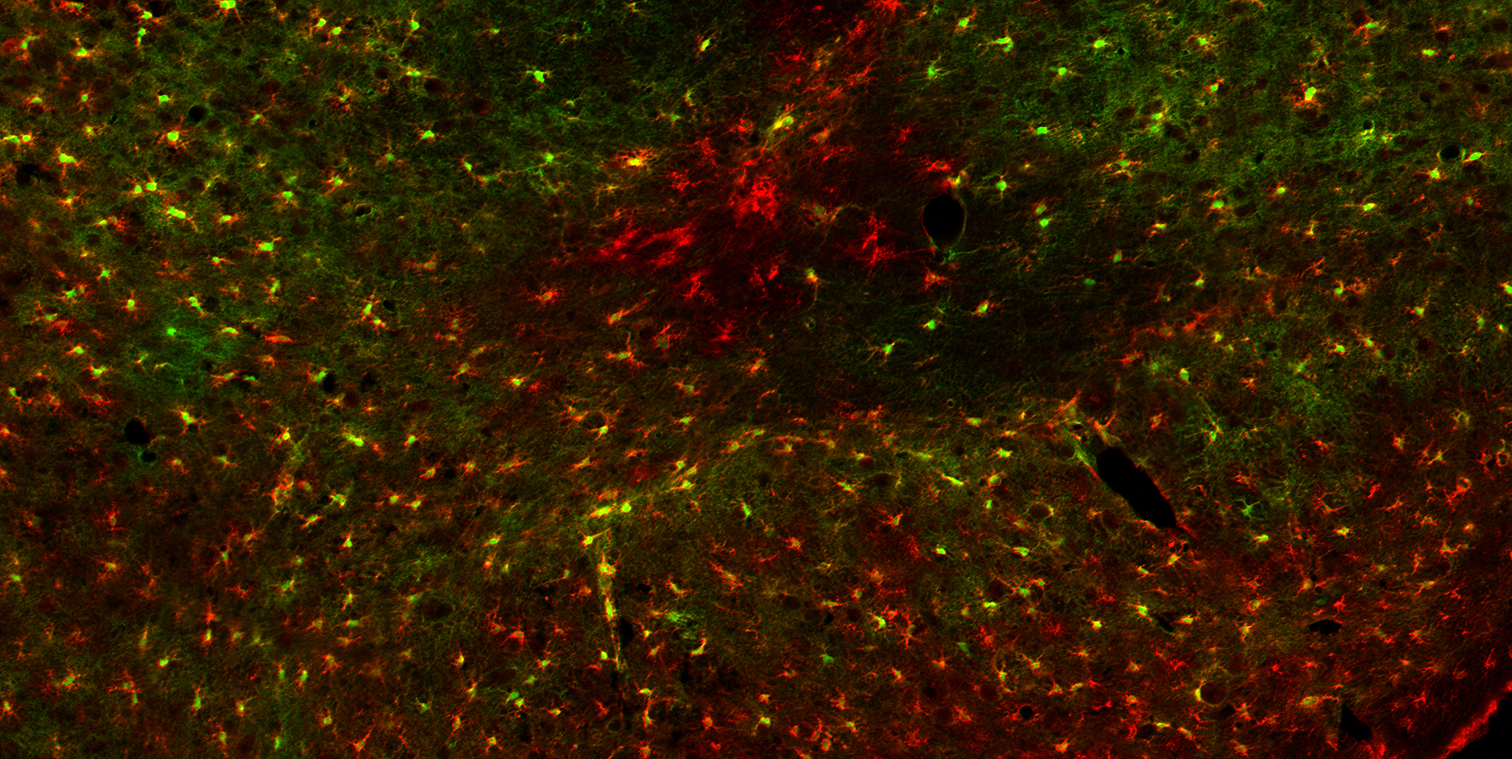

Supplement: Figure 3—source data 2. [file elife-75636-fig3-data2.zip › Fig3 source data 2 for Fig3 C/AAV-shscramble SN #81 GFP+HA-1.jpg]

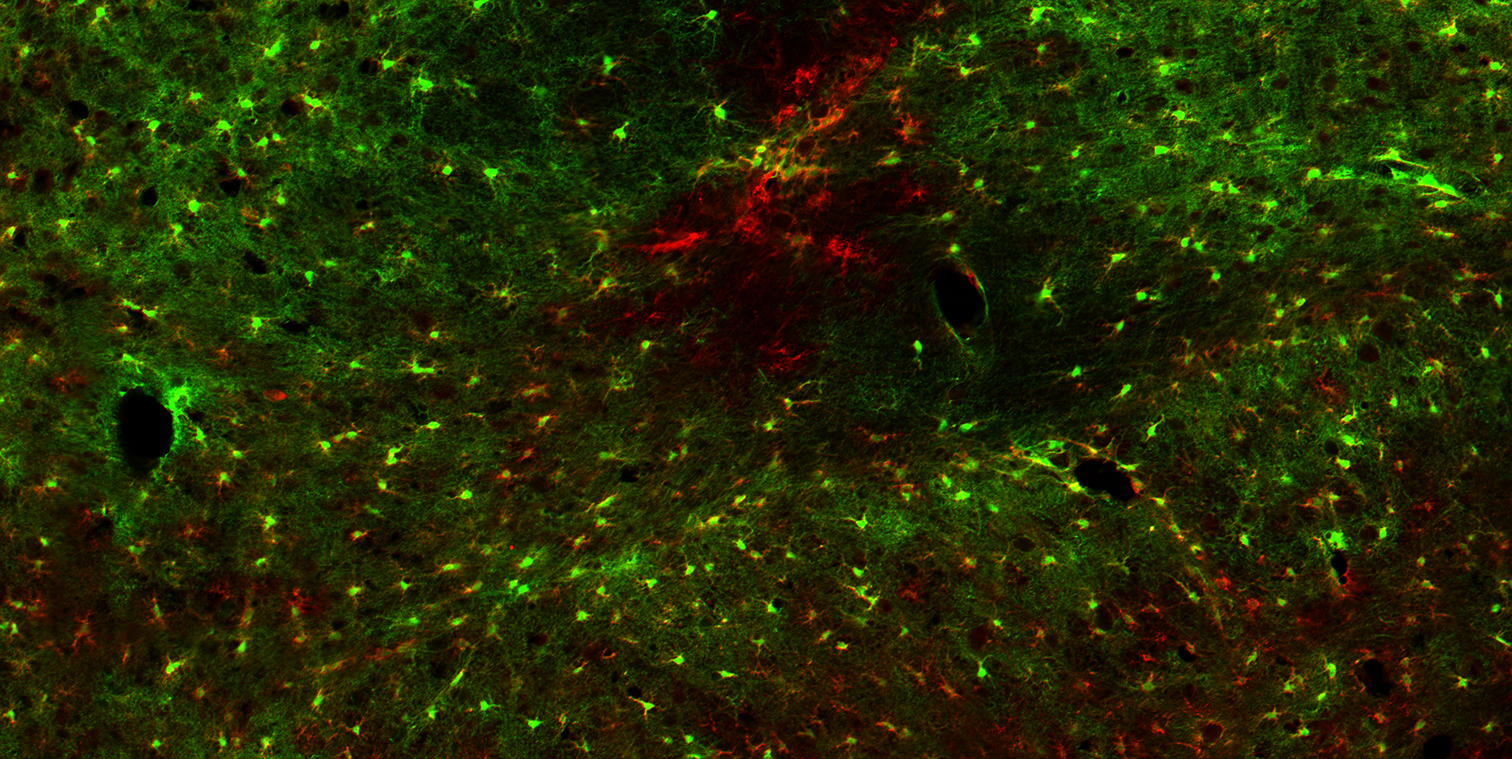

Supplement: Figure 3—source data 2. [file elife-75636-fig3-data2.zip › Fig3 source data 2 for Fig3 C/AAV-shscramble SN #81 GFP+HA-2.jpg]

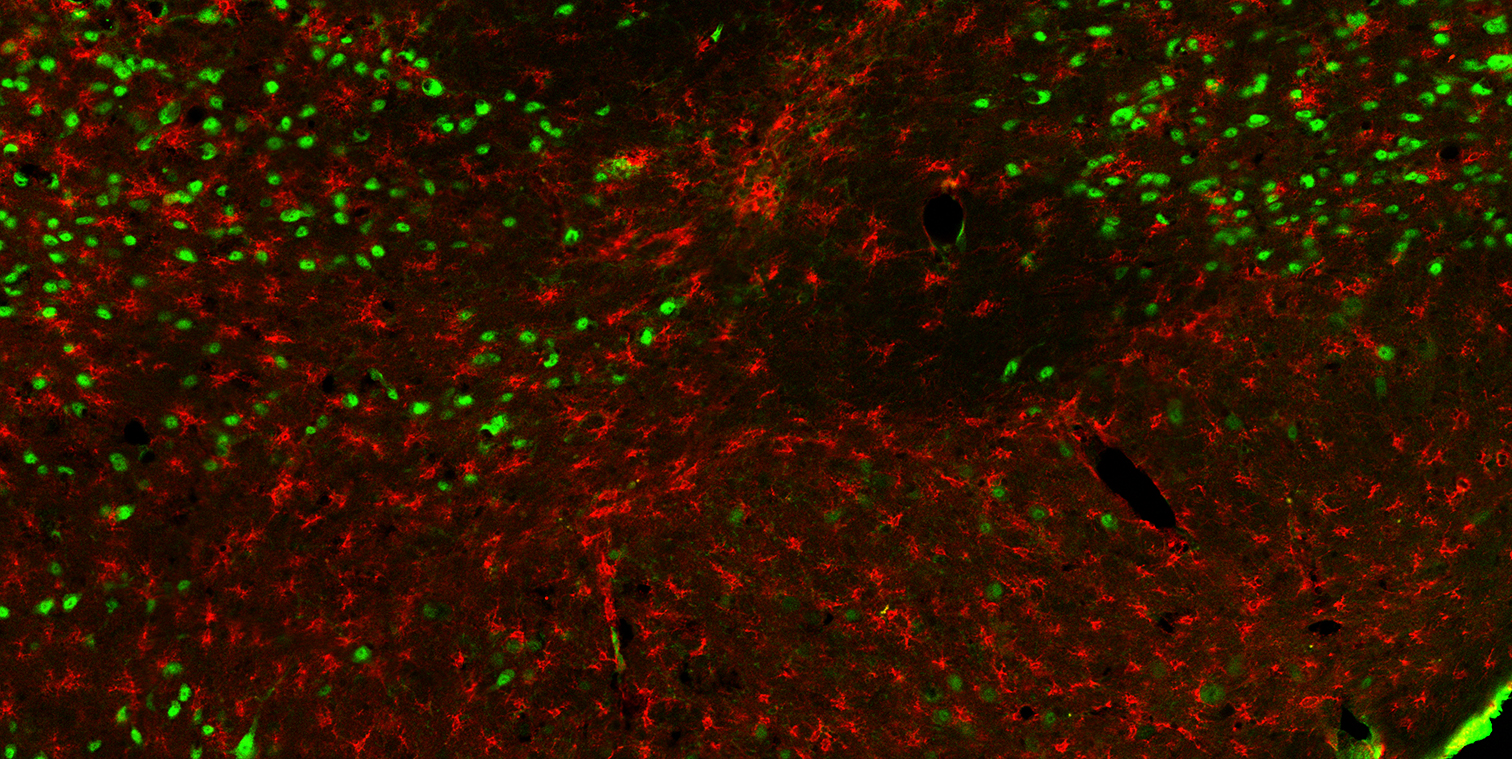

Supplement: Figure 3—source data 2. [file elife-75636-fig3-data2.zip › Fig3 source data 2 for Fig3 C/AAV-shscramble SN #81 NeuN+HA.jpg]

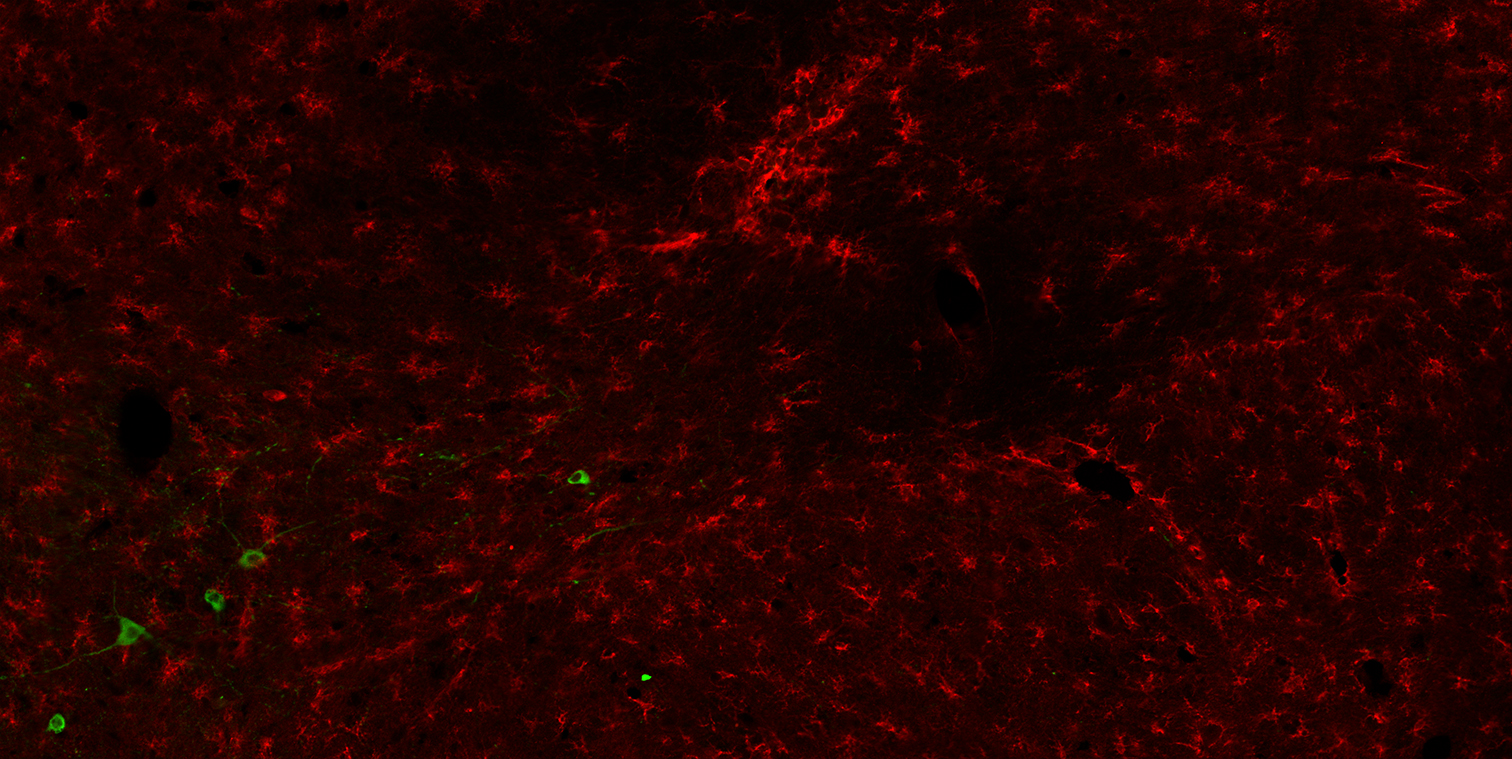

Supplement: Figure 3—source data 2. [file elife-75636-fig3-data2.zip › Fig3 source data 2 for Fig3 C/AAV-shscramble SN #81 TH+HA.jpg]

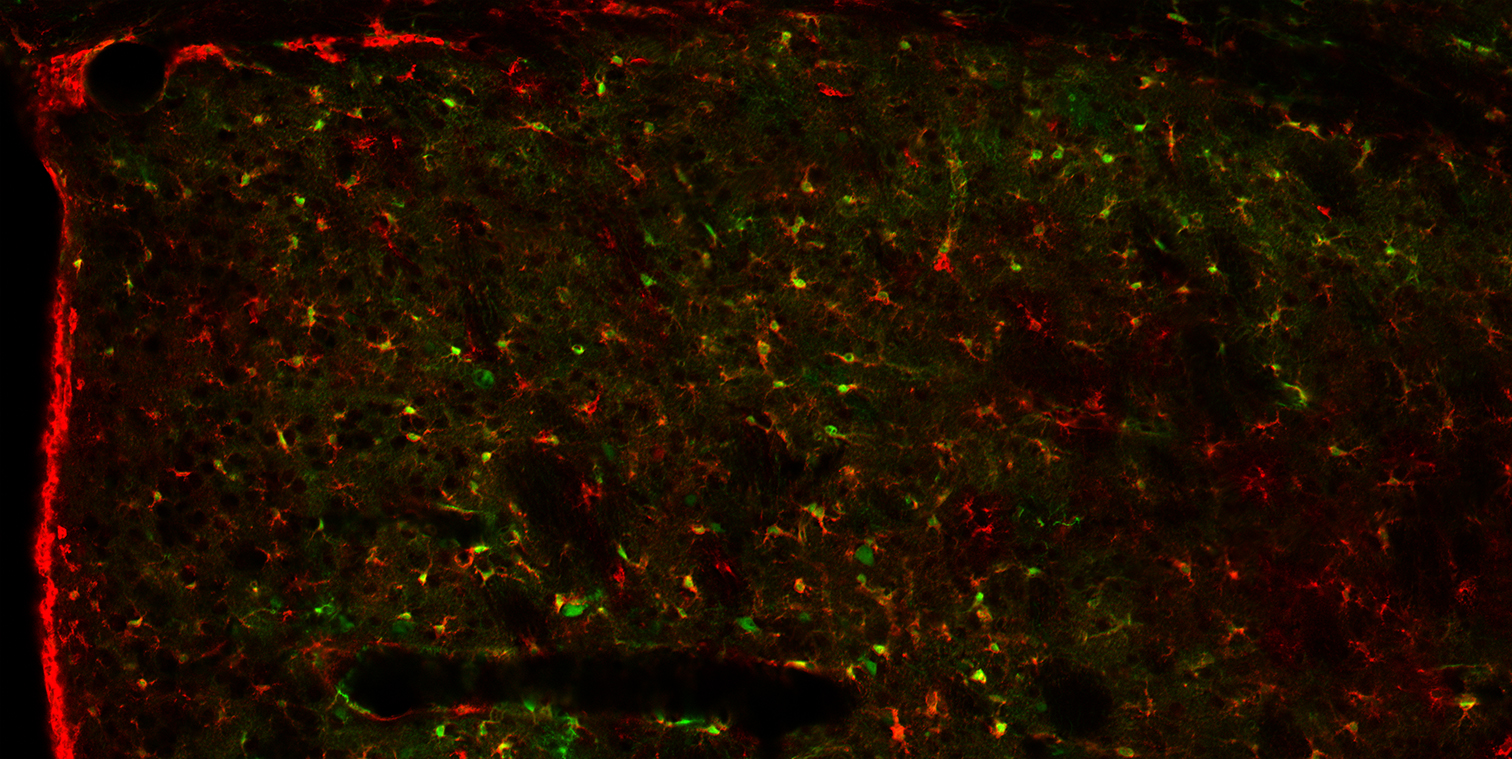

Supplement: Figure 3—source data 2. [file elife-75636-fig3-data2.zip › Fig3 source data 2 for Fig3 C/AAV-shscramble STR #18 GFP+HA-1.jpg]

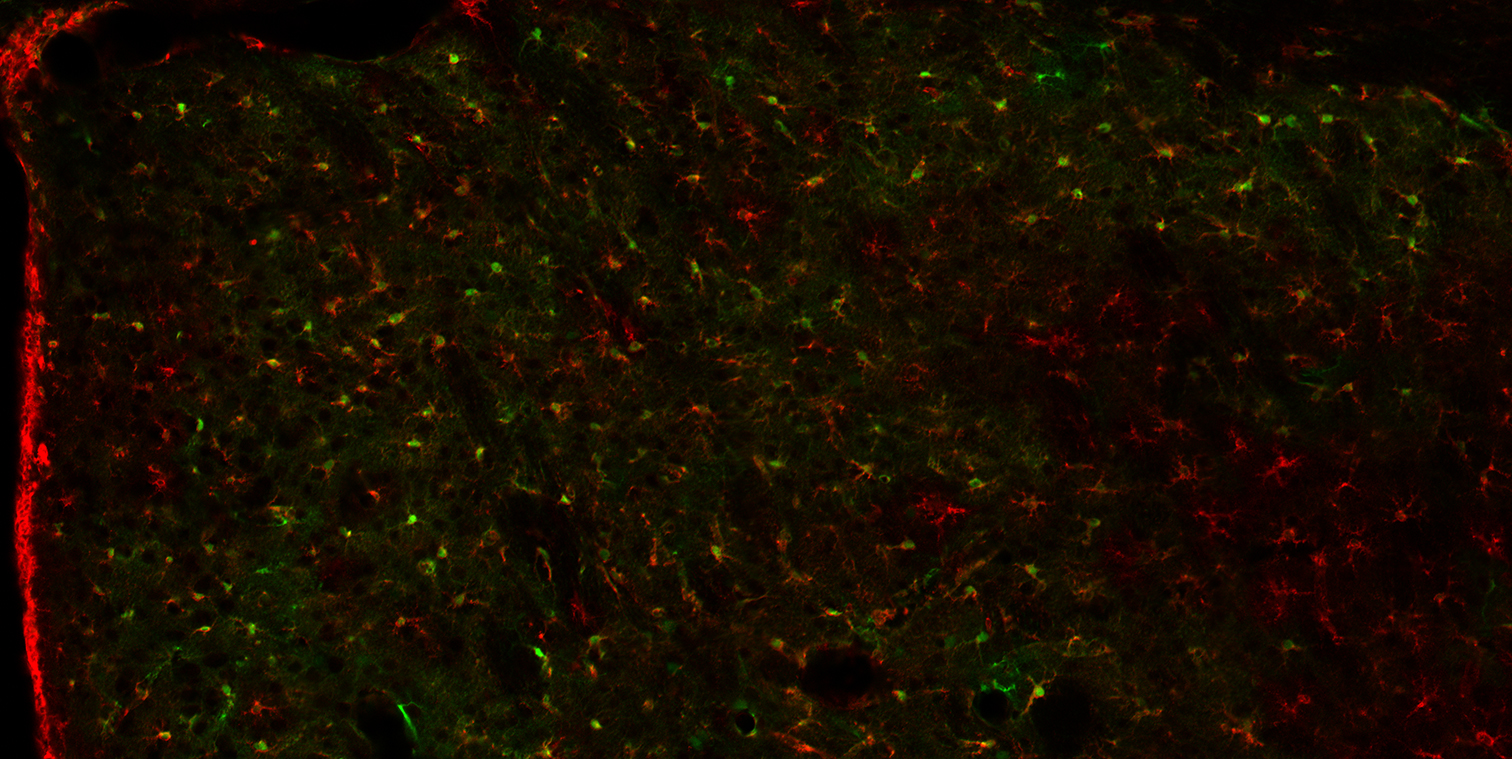

Supplement: Figure 3—source data 2. [file elife-75636-fig3-data2.zip › Fig3 source data 2 for Fig3 C/AAV-shscramble STR #18 GFP+HA-2.jpg]

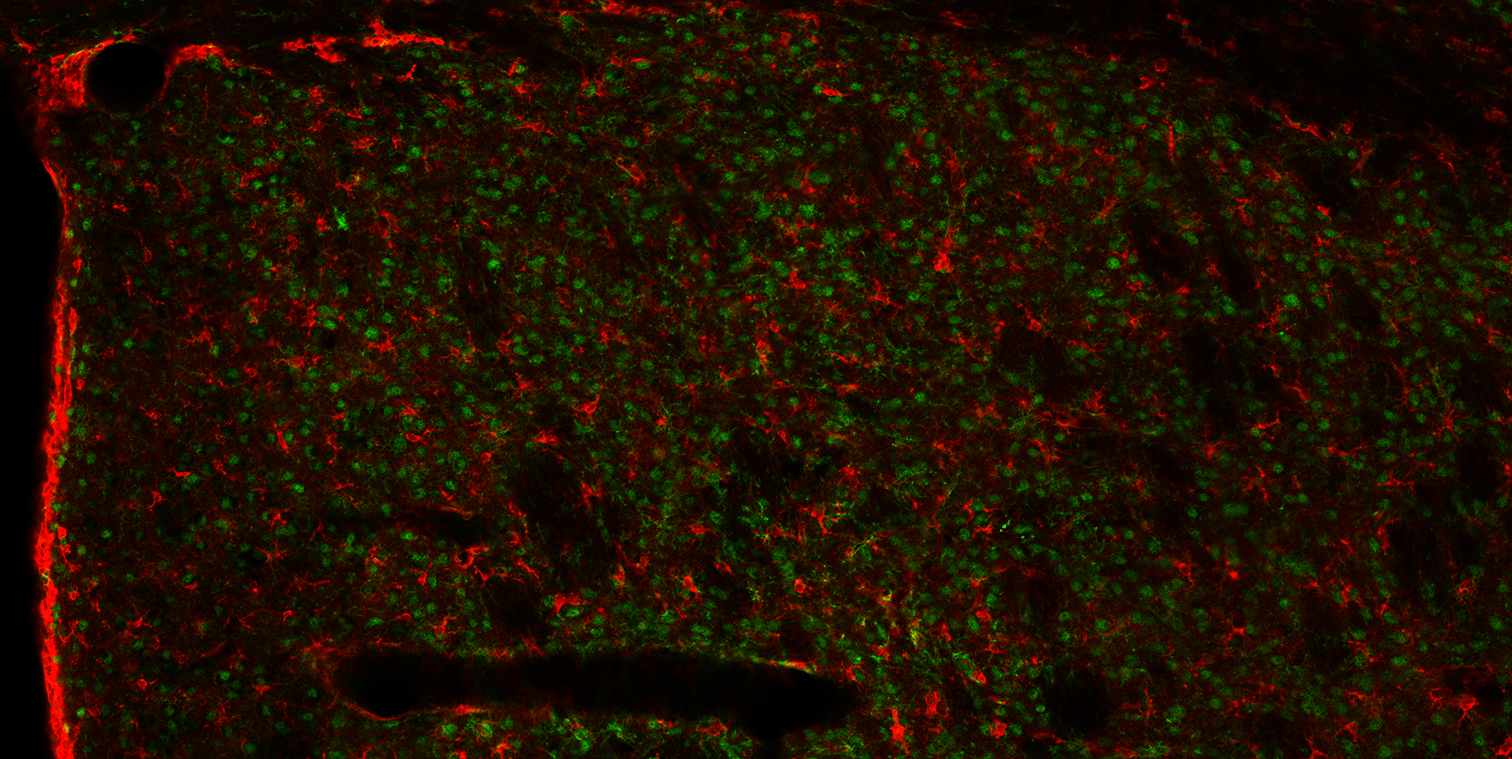

Supplement: Figure 3—source data 2. [file elife-75636-fig3-data2.zip › Fig3 source data 2 for Fig3 C/AAV-shscramble STR #18 NeuN+HA.jpg]

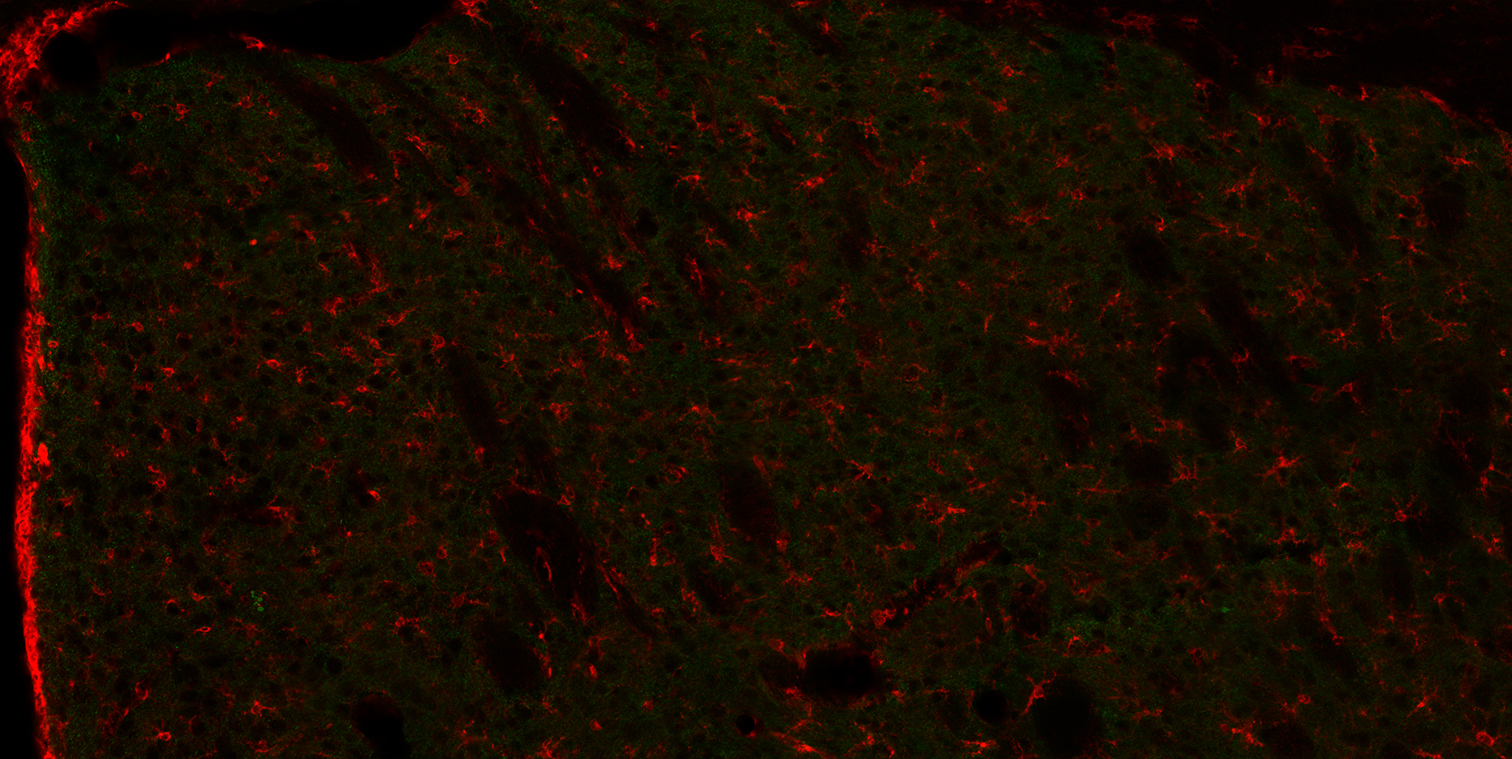

Supplement: Figure 3—source data 2. [file elife-75636-fig3-data2.zip › Fig3 source data 2 for Fig3 C/AAV-shscramble STR #18 TH+HA.jpg]

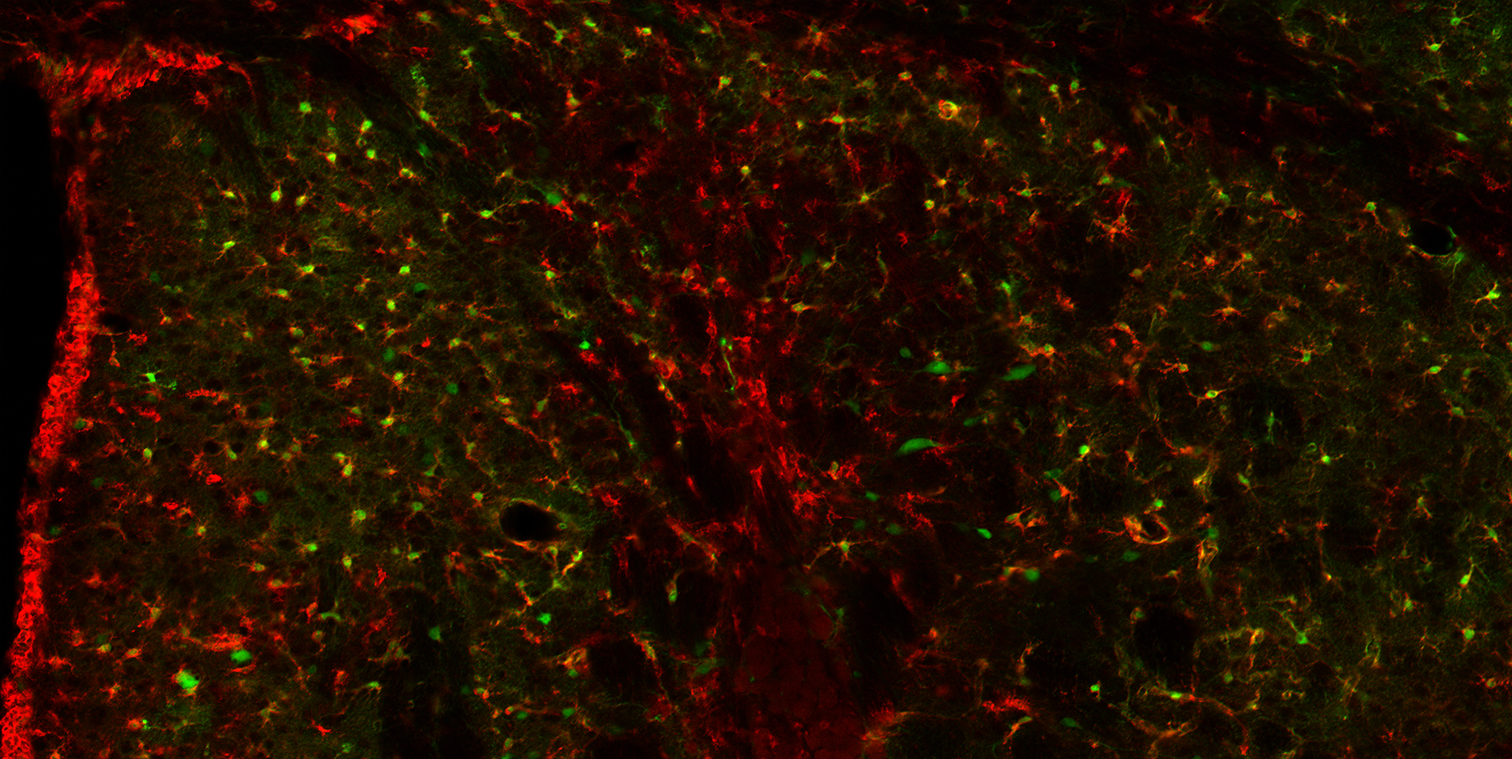

Supplement: Figure 3—source data 2. [file elife-75636-fig3-data2.zip › Fig3 source data 2 for Fig3 C/AAV-shscramble STR #56 GFP+HA-1.jpg]

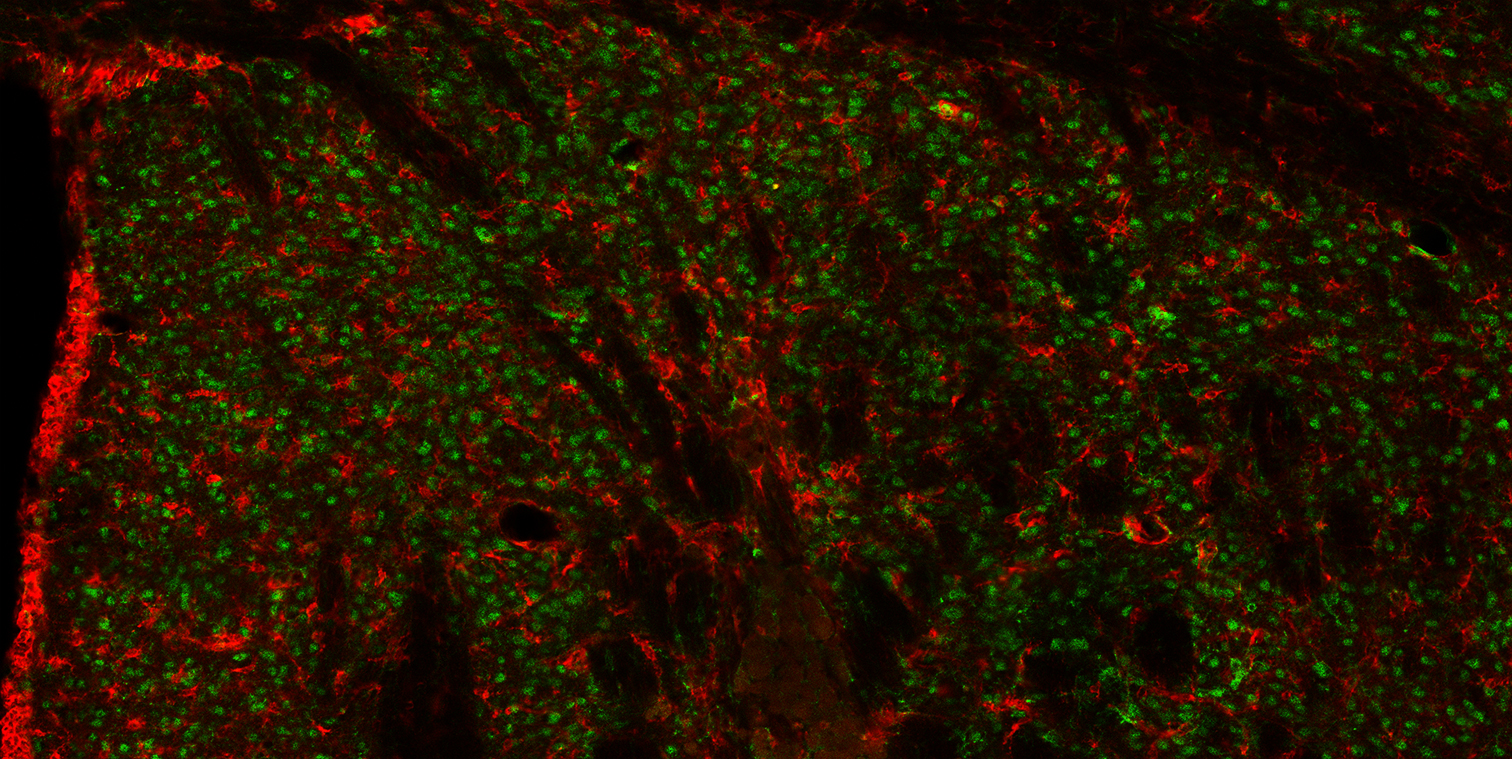

Supplement: Figure 3—source data 2. [file elife-75636-fig3-data2.zip › Fig3 source data 2 for Fig3 C/AAV-shscramble STR #56 NeuN+HA.jpg]

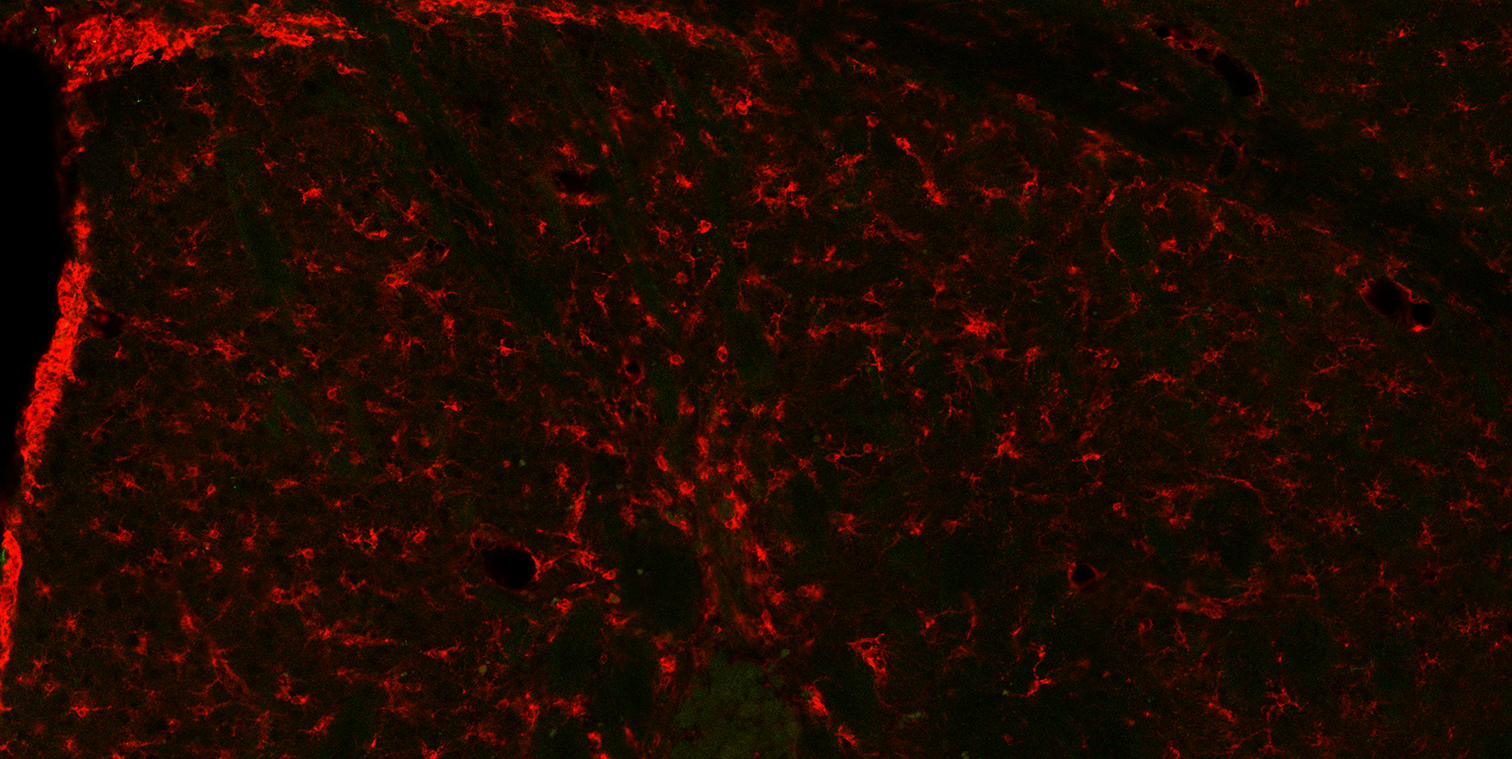

Supplement: Figure 3—source data 2. [file elife-75636-fig3-data2.zip › Fig3 source data 2 for Fig3 C/AAV-shscramble STR #56 TH+HA.jpg]

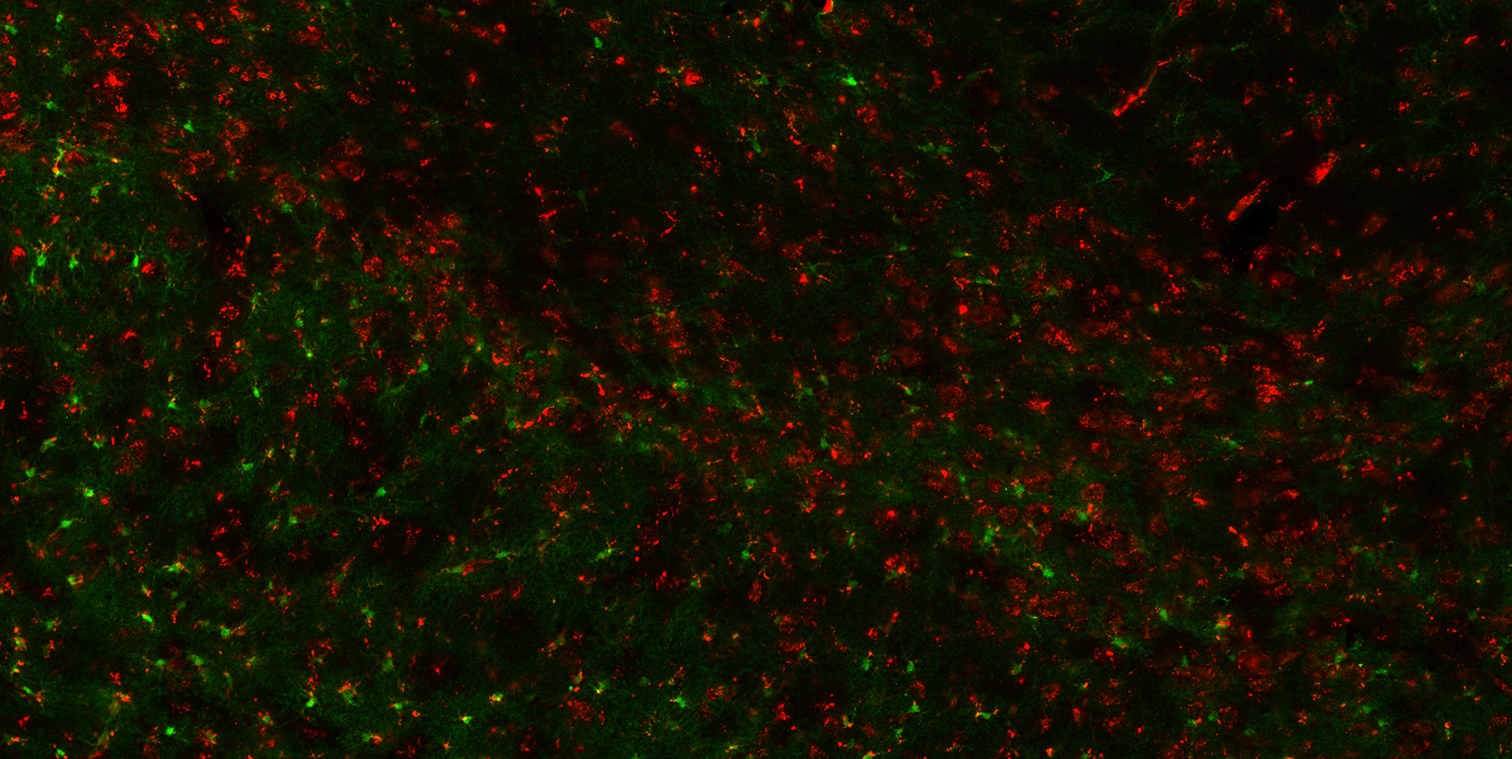

Supplement: Figure 4—source data 1. [file elife-75636-fig4-data1.zip › Fig4 source data 1 for Fig4 B/ASO CTRL #22 YFP+CY3-1.jpg]

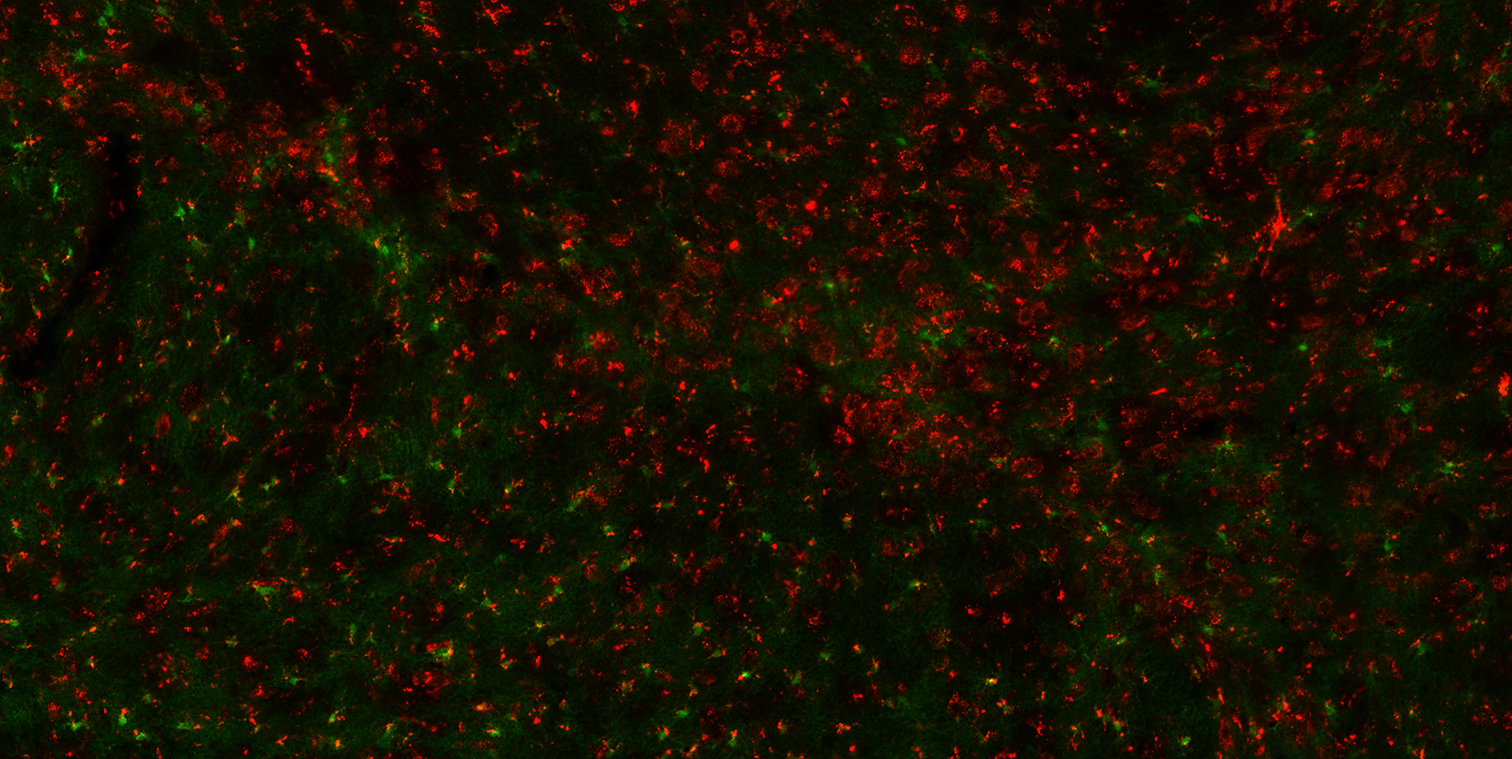

Supplement: Figure 4—source data 1. [file elife-75636-fig4-data1.zip › Fig4 source data 1 for Fig4 B/ASO CTRL #22 YFP+CY3-2.jpg]

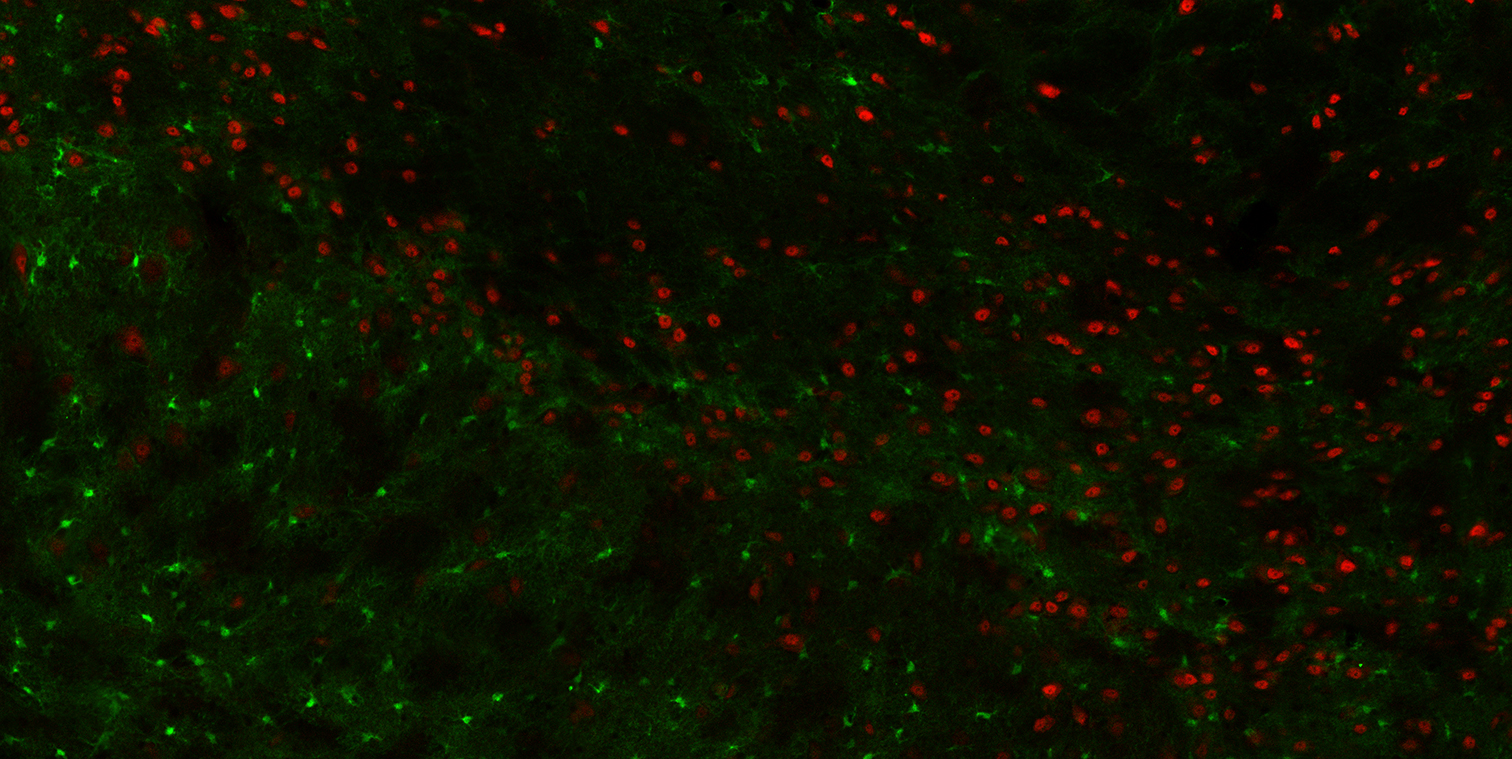

Supplement: Figure 4—source data 1. [file elife-75636-fig4-data1.zip › Fig4 source data 1 for Fig4 B/ASO CTRL #22 YFP+NeuN.jpg]

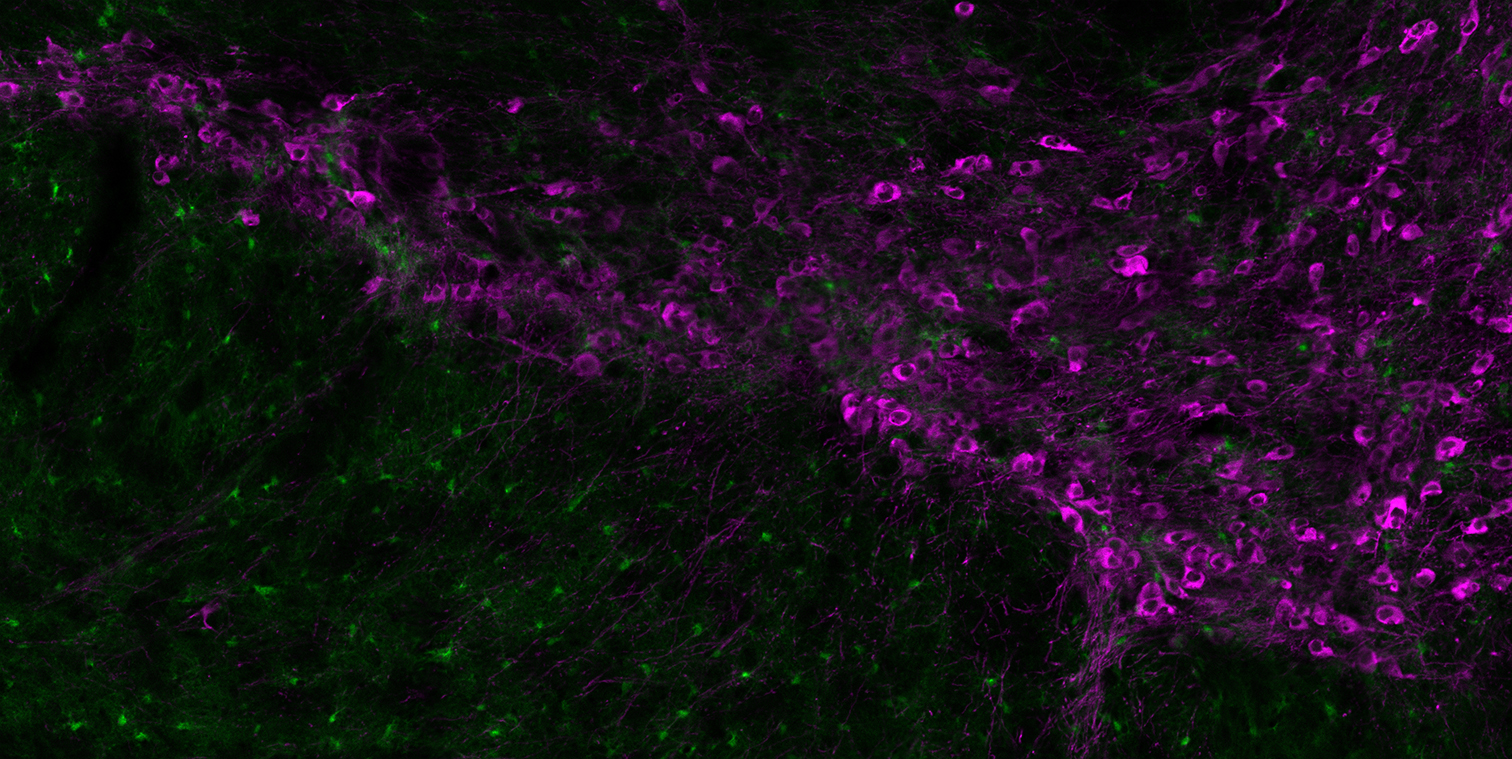

Supplement: Figure 4—source data 1. [file elife-75636-fig4-data1.zip › Fig4 source data 1 for Fig4 B/ASO CTRL #22 YFP+TH.jpg]

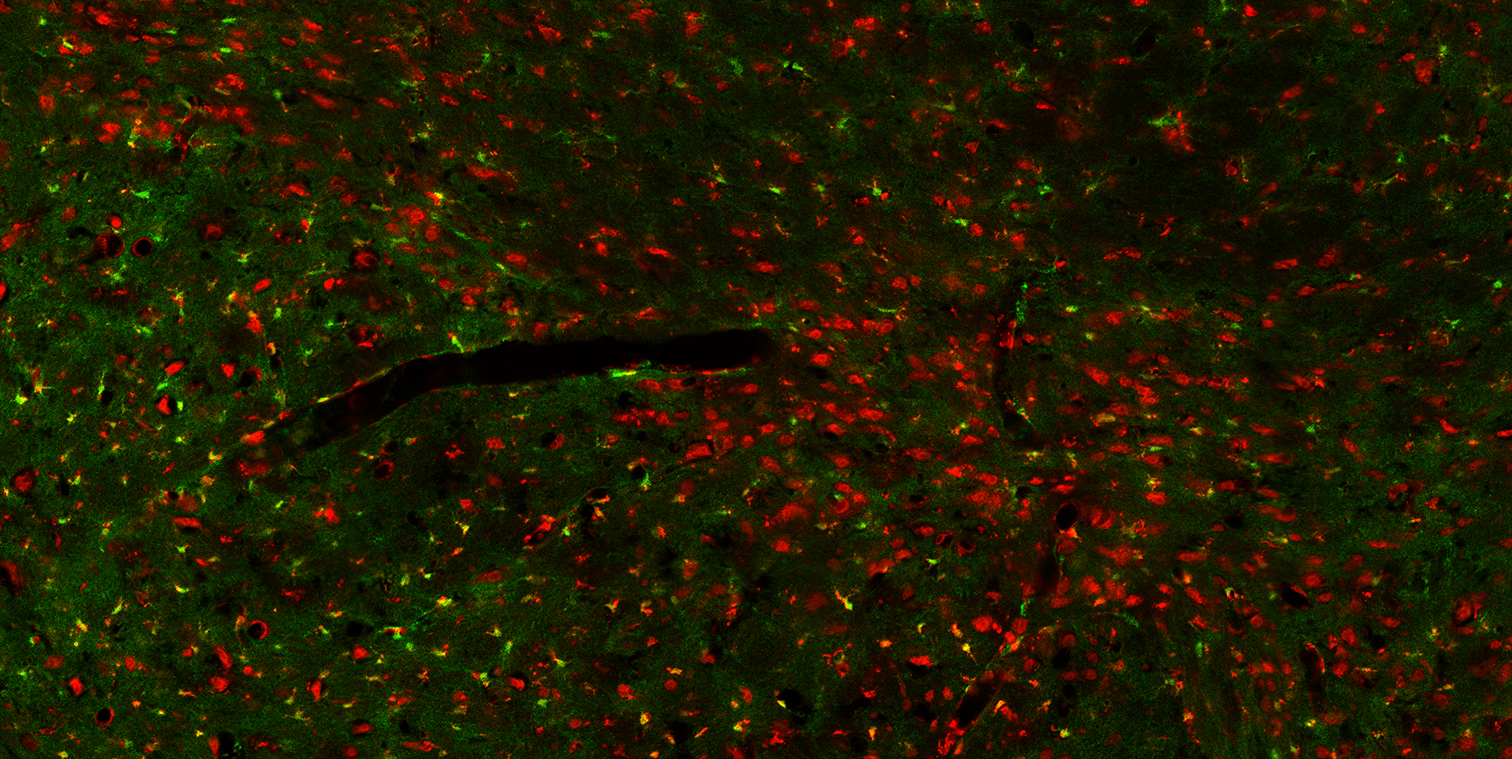

Supplement: Figure 4—source data 1. [file elife-75636-fig4-data1.zip › Fig4 source data 1 for Fig4 B/ASO CTRL #23 YFP+CY3-1.jpg]

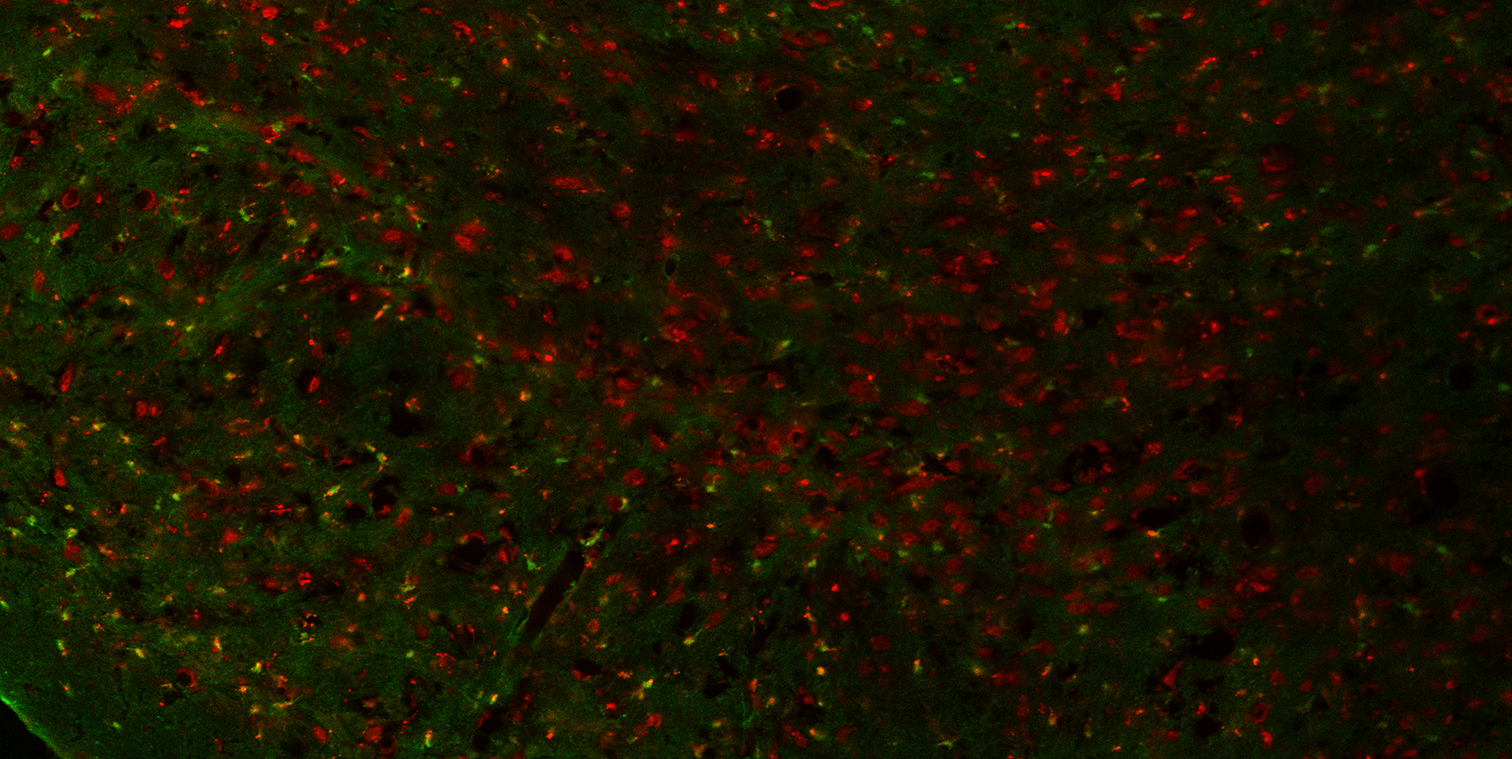

Supplement: Figure 4—source data 1. [file elife-75636-fig4-data1.zip › Fig4 source data 1 for Fig4 B/ASO CTRL #23 YFP+CY3-2.jpg]

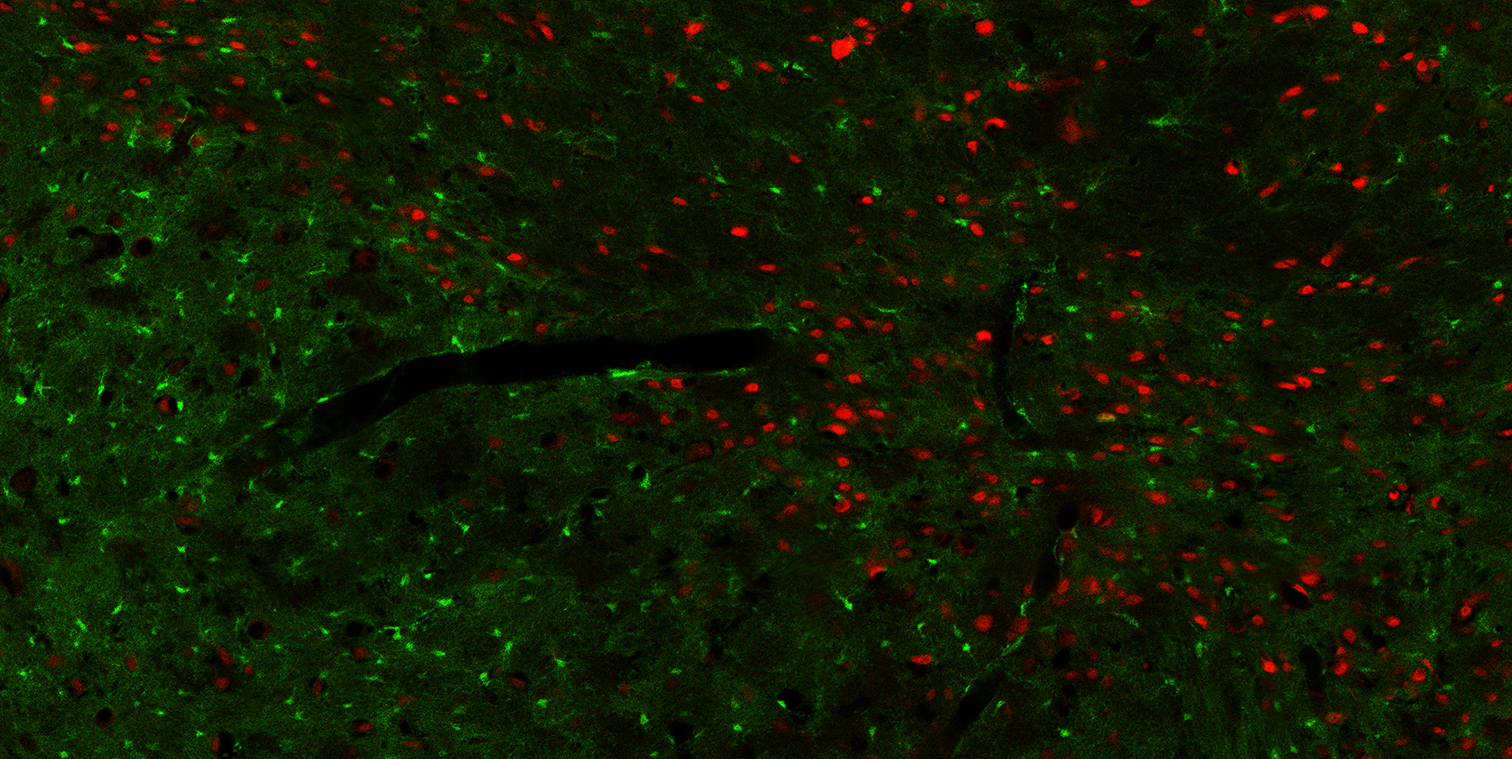

Supplement: Figure 4—source data 1. [file elife-75636-fig4-data1.zip › Fig4 source data 1 for Fig4 B/ASO CTRL #23 YFP+NeuN.jpg]

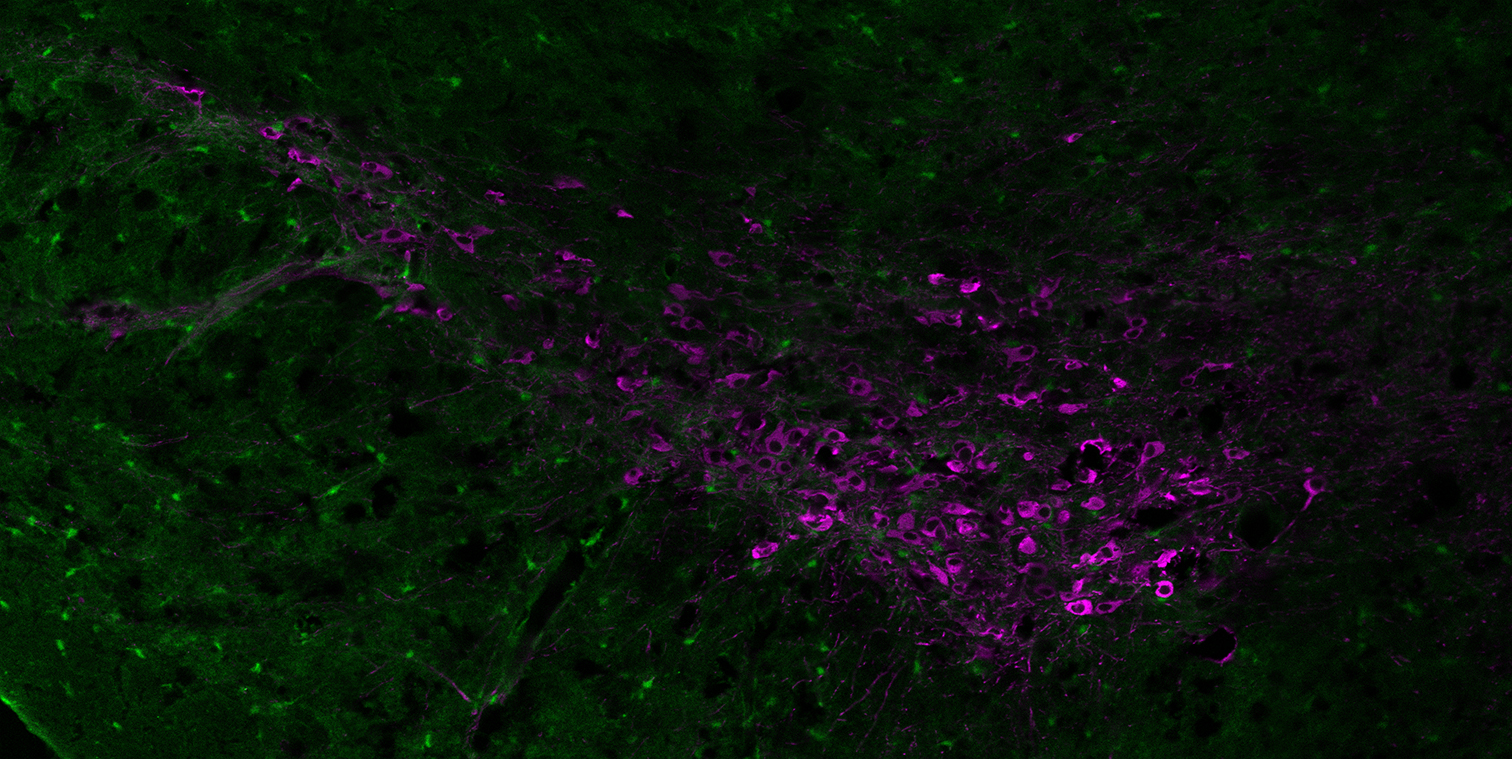

Supplement: Figure 4—source data 1. [file elife-75636-fig4-data1.zip › Fig4 source data 1 for Fig4 B/ASO CTRL #23 YFP+TH.jpg]

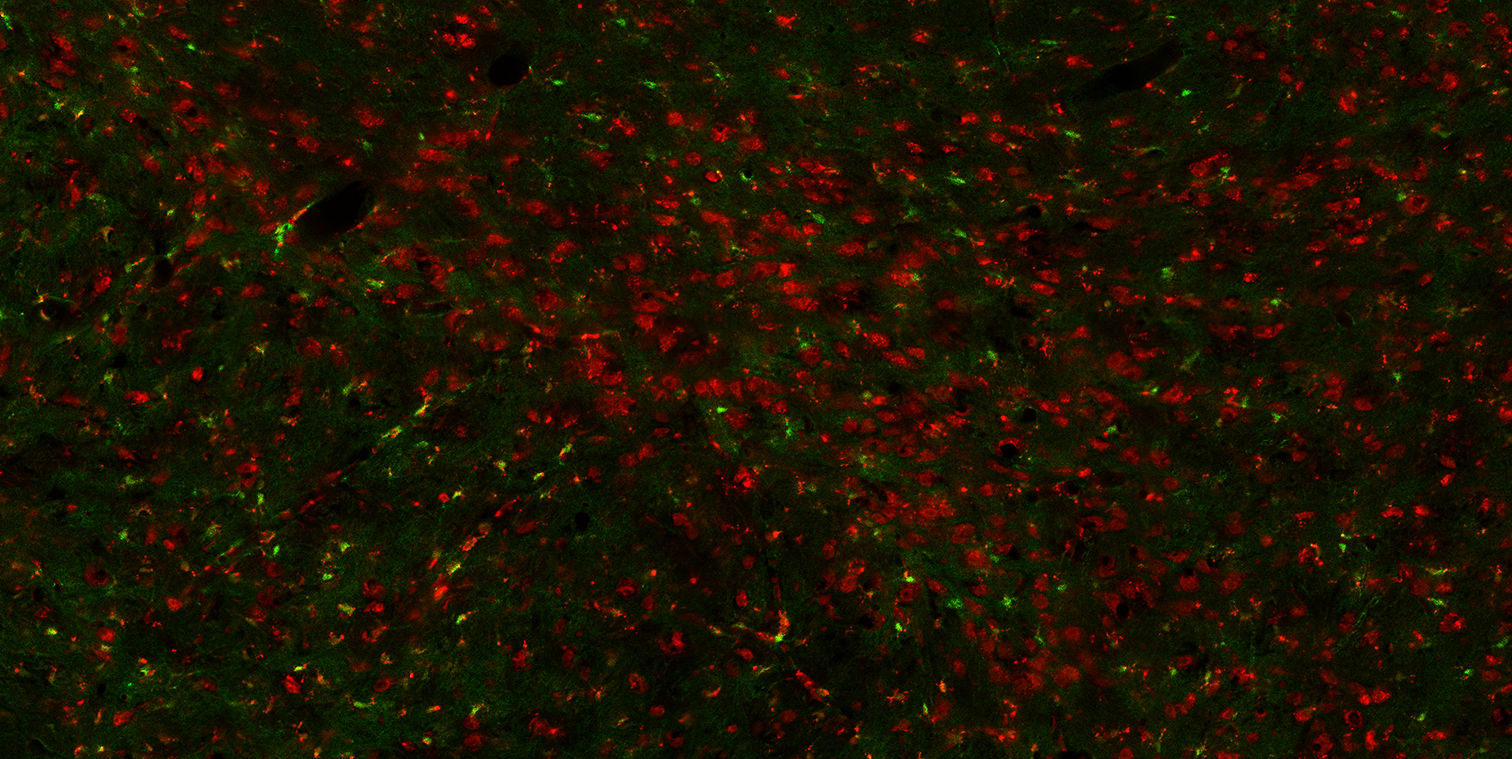

Supplement: Figure 4—source data 1. [file elife-75636-fig4-data1.zip › Fig4 source data 1 for Fig4 B/ASO CTRL #24 YFP+CY3-1.jpg]

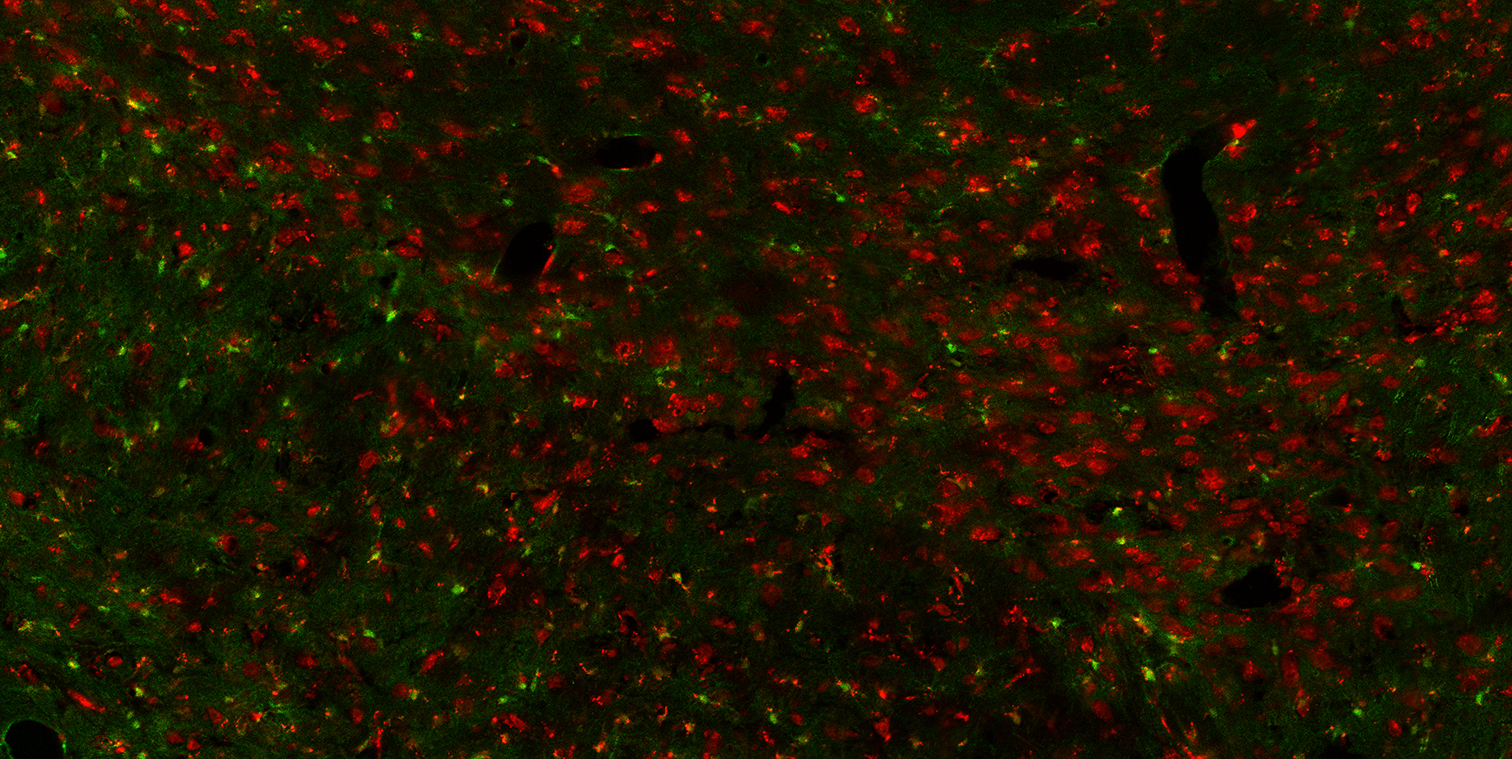

Supplement: Figure 4—source data 1. [file elife-75636-fig4-data1.zip › Fig4 source data 1 for Fig4 B/ASO CTRL #24 YFP+CY3-2.jpg]

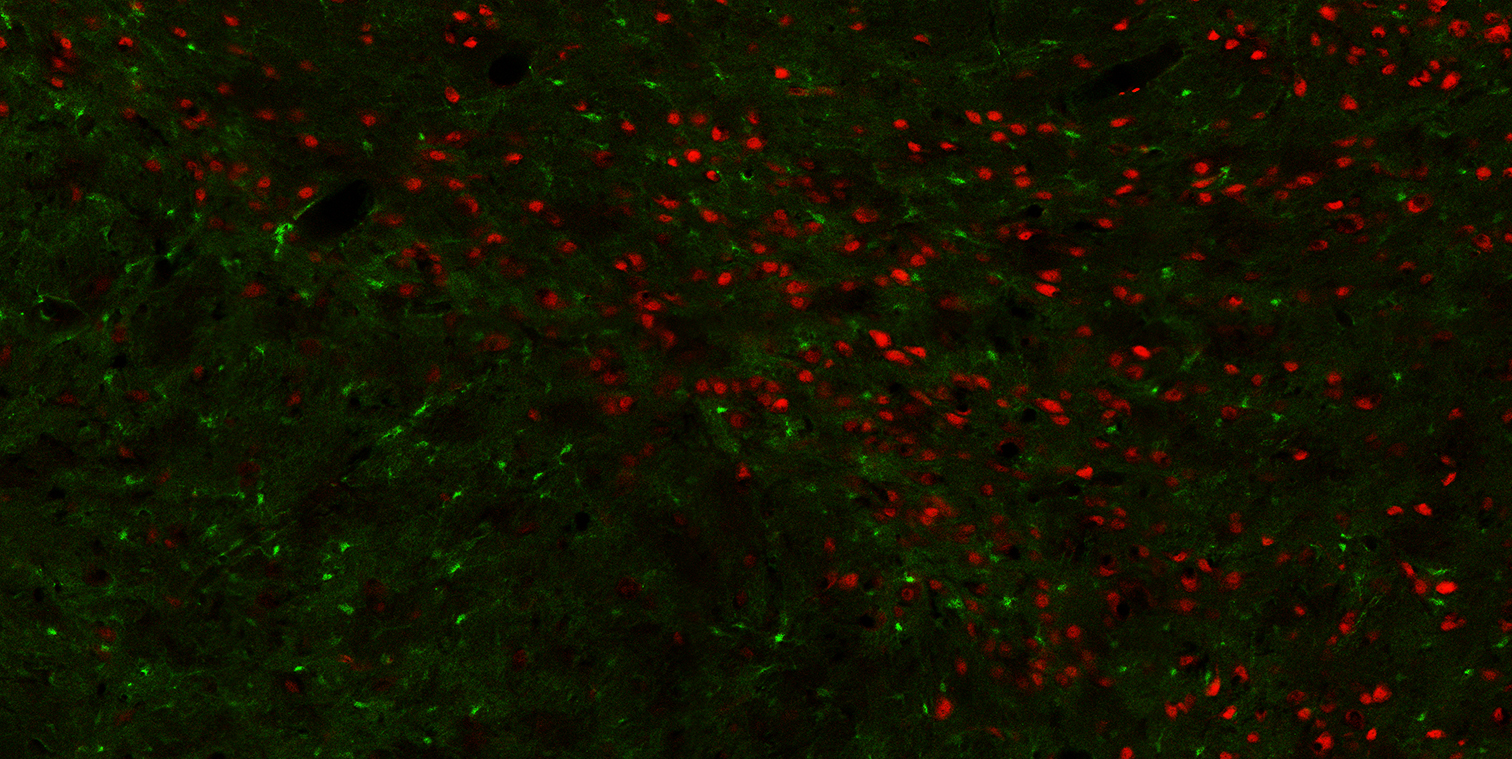

Supplement: Figure 4—source data 1. [file elife-75636-fig4-data1.zip › Fig4 source data 1 for Fig4 B/ASO CTRL #24 YFP+NeuN.jpg]

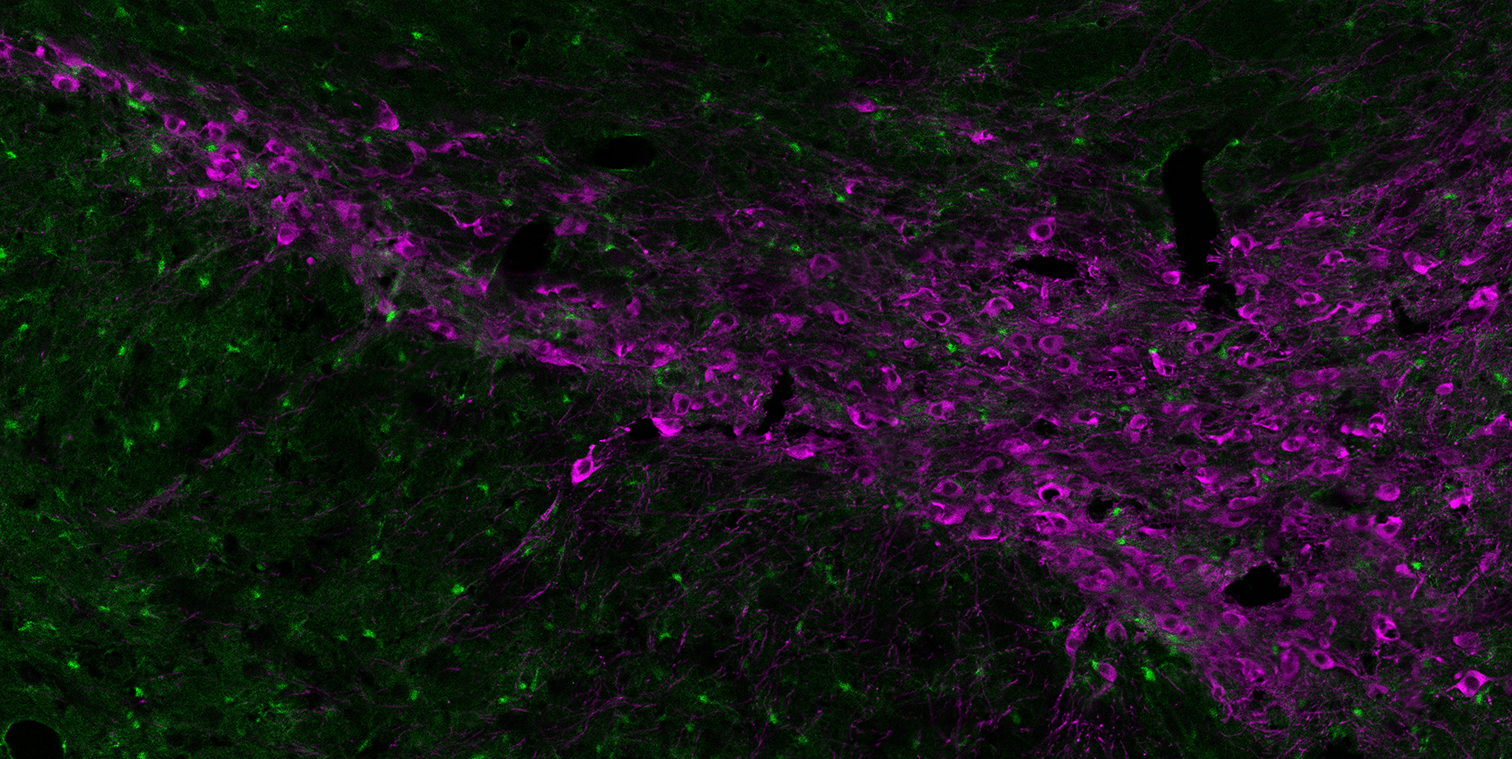

Supplement: Figure 4—source data 1. [file elife-75636-fig4-data1.zip › Fig4 source data 1 for Fig4 B/ASO CTRL #24 YFP+TH.jpg]

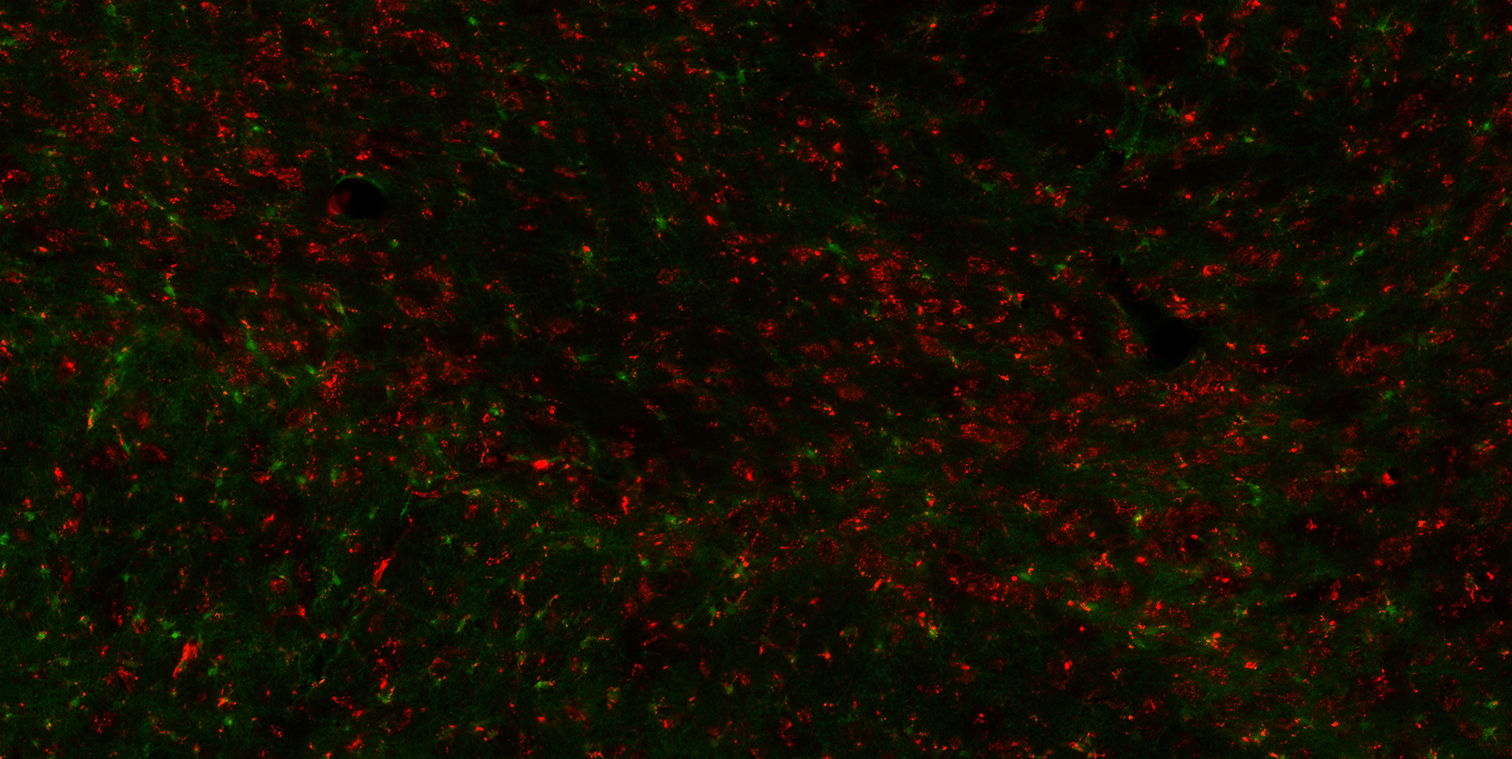

Supplement: Figure 4—source data 1. [file elife-75636-fig4-data1.zip › Fig4 source data 1 for Fig4 B/ASO CTRL #26 YFP+CY3-1.jpg]

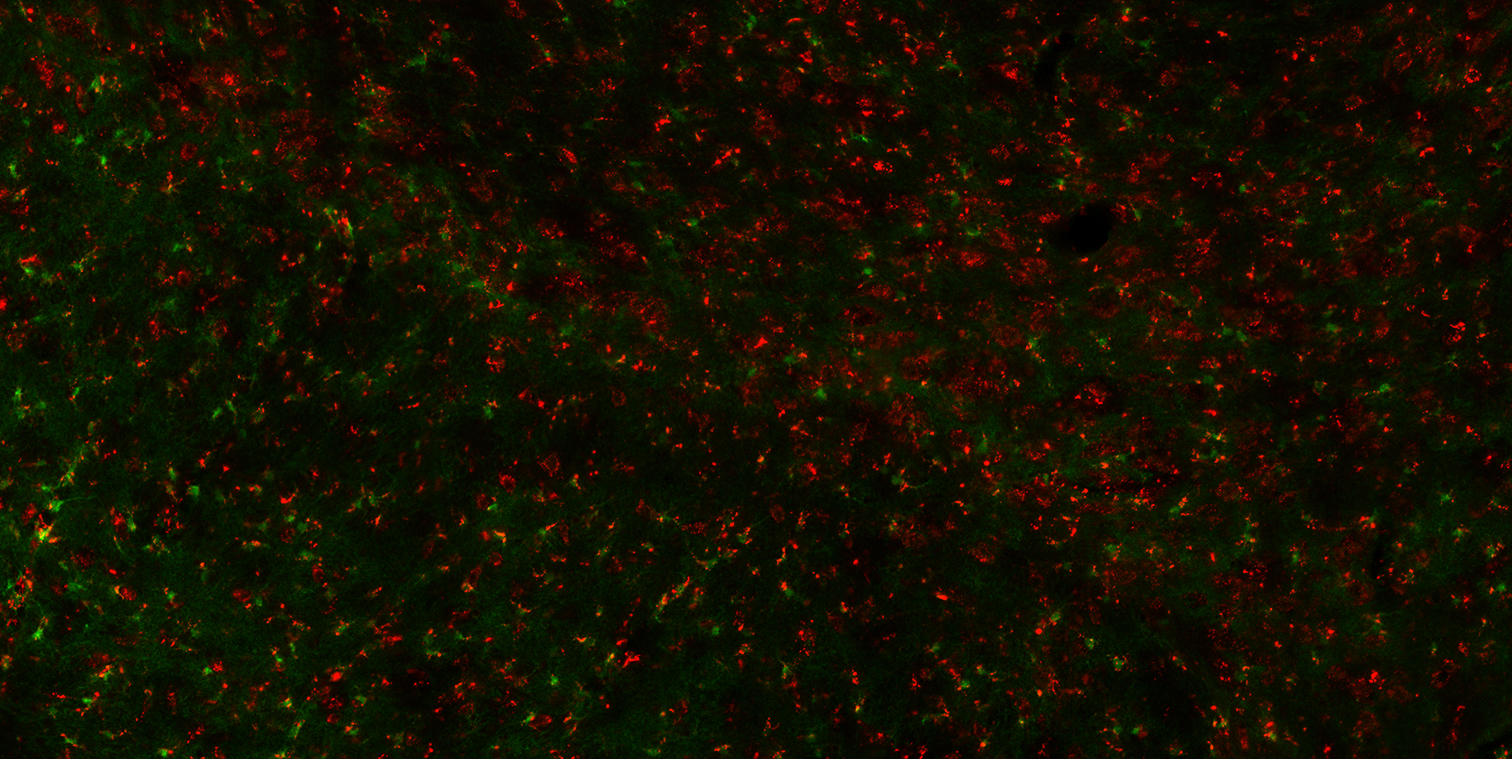

Supplement: Figure 4—source data 1. [file elife-75636-fig4-data1.zip › Fig4 source data 1 for Fig4 B/ASO CTRL #26 YFP+CY3-2.jpg]

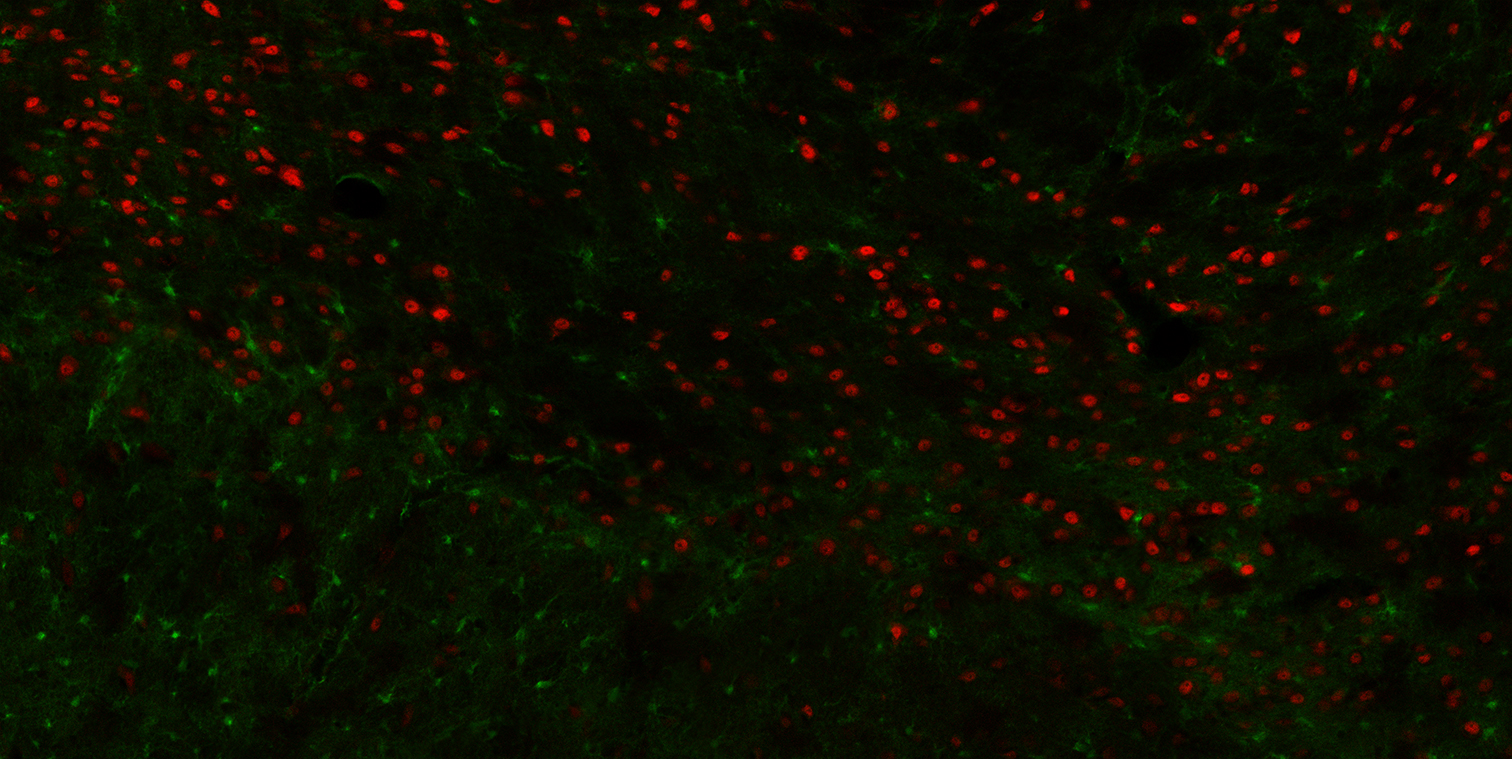

Supplement: Figure 4—source data 1. [file elife-75636-fig4-data1.zip › Fig4 source data 1 for Fig4 B/ASO CTRL #26 YFP+NeuN.jpg]

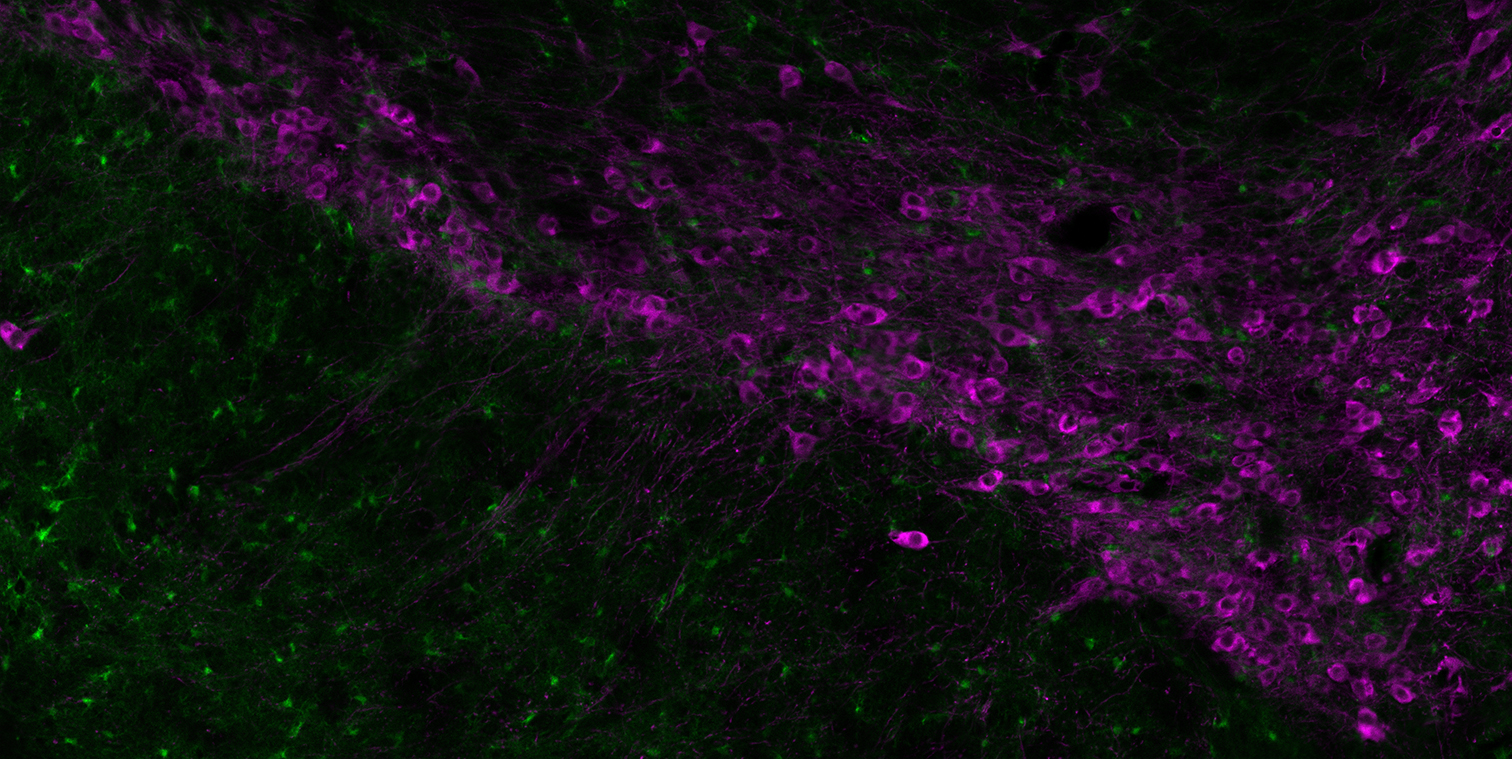

Supplement: Figure 4—source data 1. [file elife-75636-fig4-data1.zip › Fig4 source data 1 for Fig4 B/ASO CTRL #26 YFP+TH.jpg]

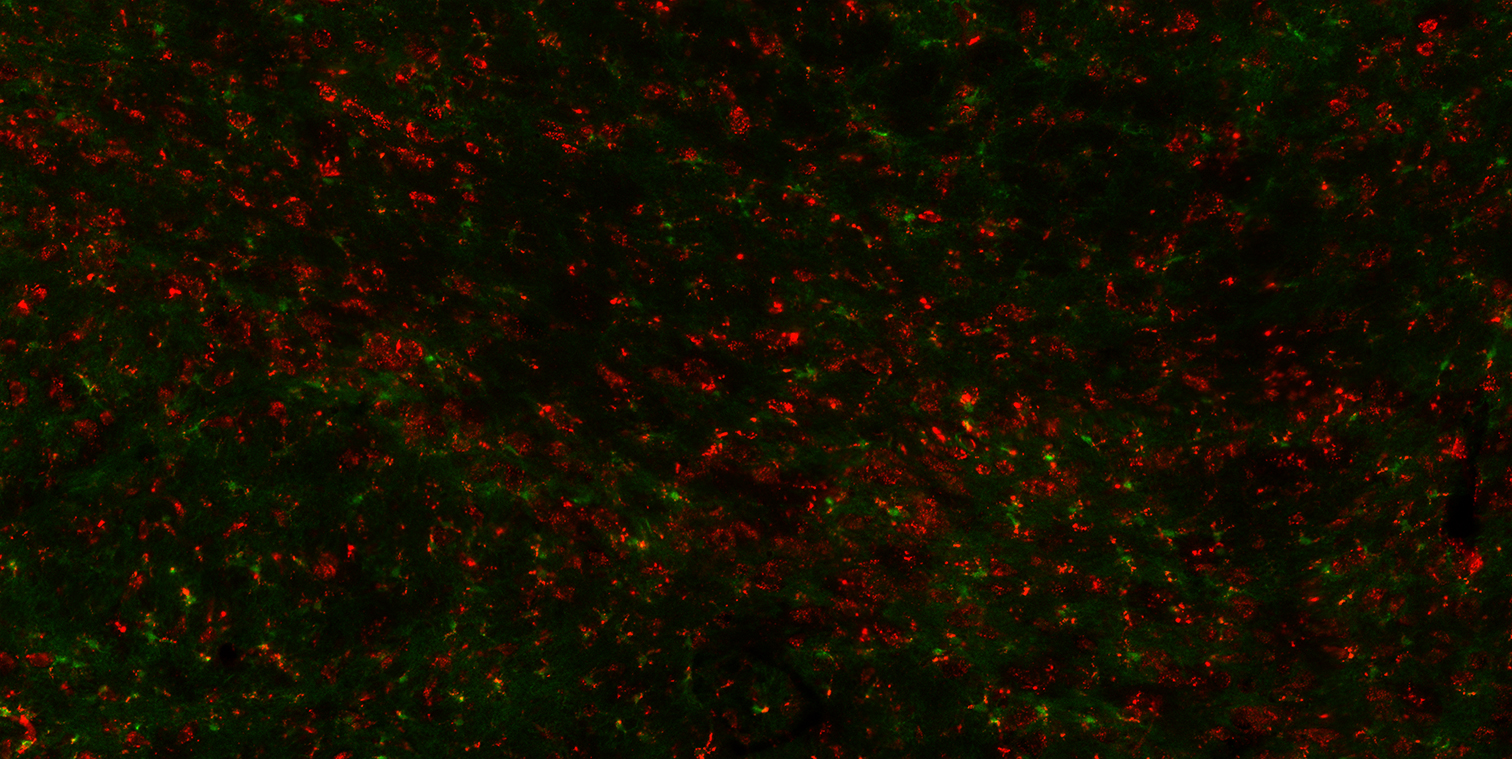

Supplement: Figure 4—source data 1. [file elife-75636-fig4-data1.zip › Fig4 source data 1 for Fig4 B/ASO CTRL #27 YFP+CY3-1.jpg]

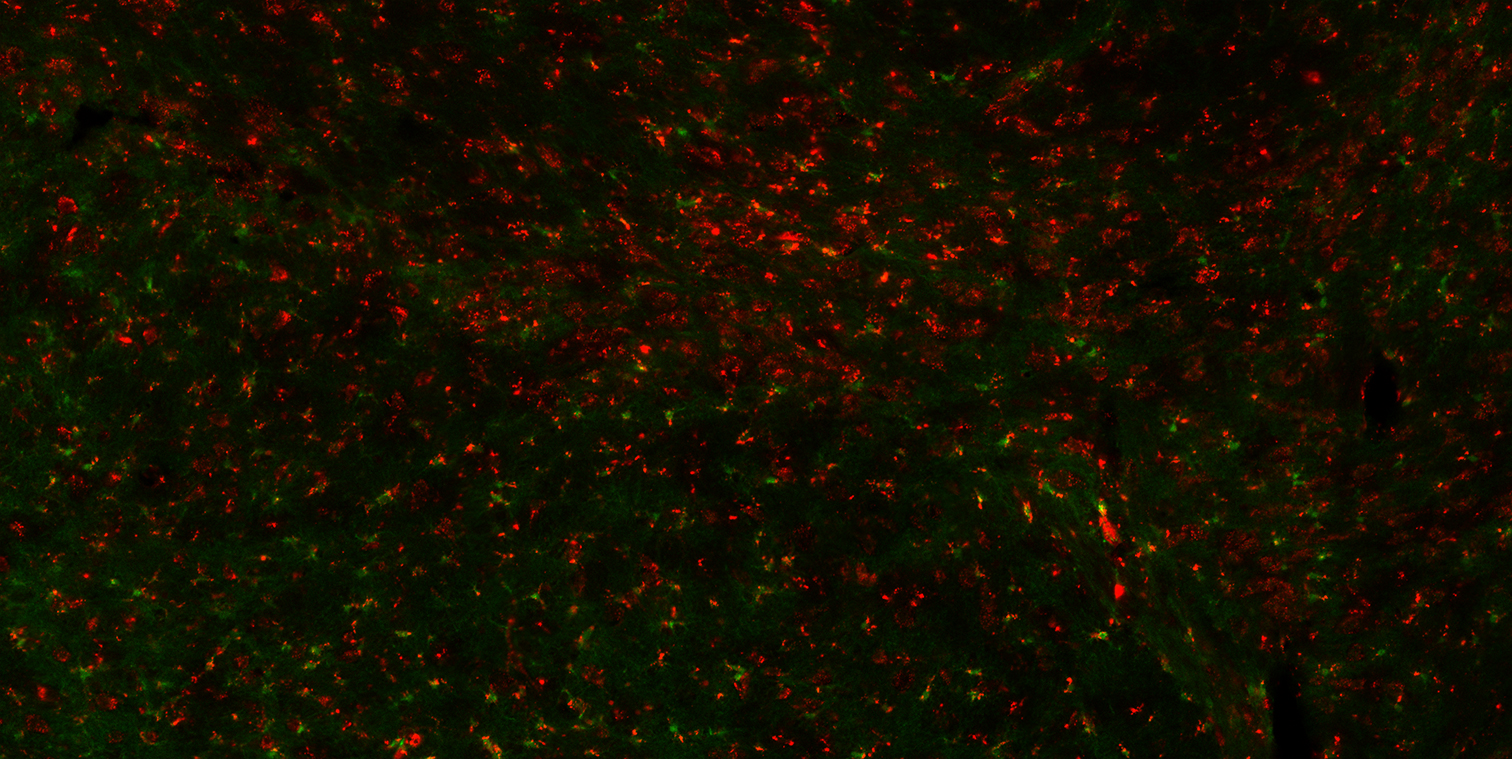

Supplement: Figure 4—source data 1. [file elife-75636-fig4-data1.zip › Fig4 source data 1 for Fig4 B/ASO CTRL #27 YFP+CY3-2.jpg]

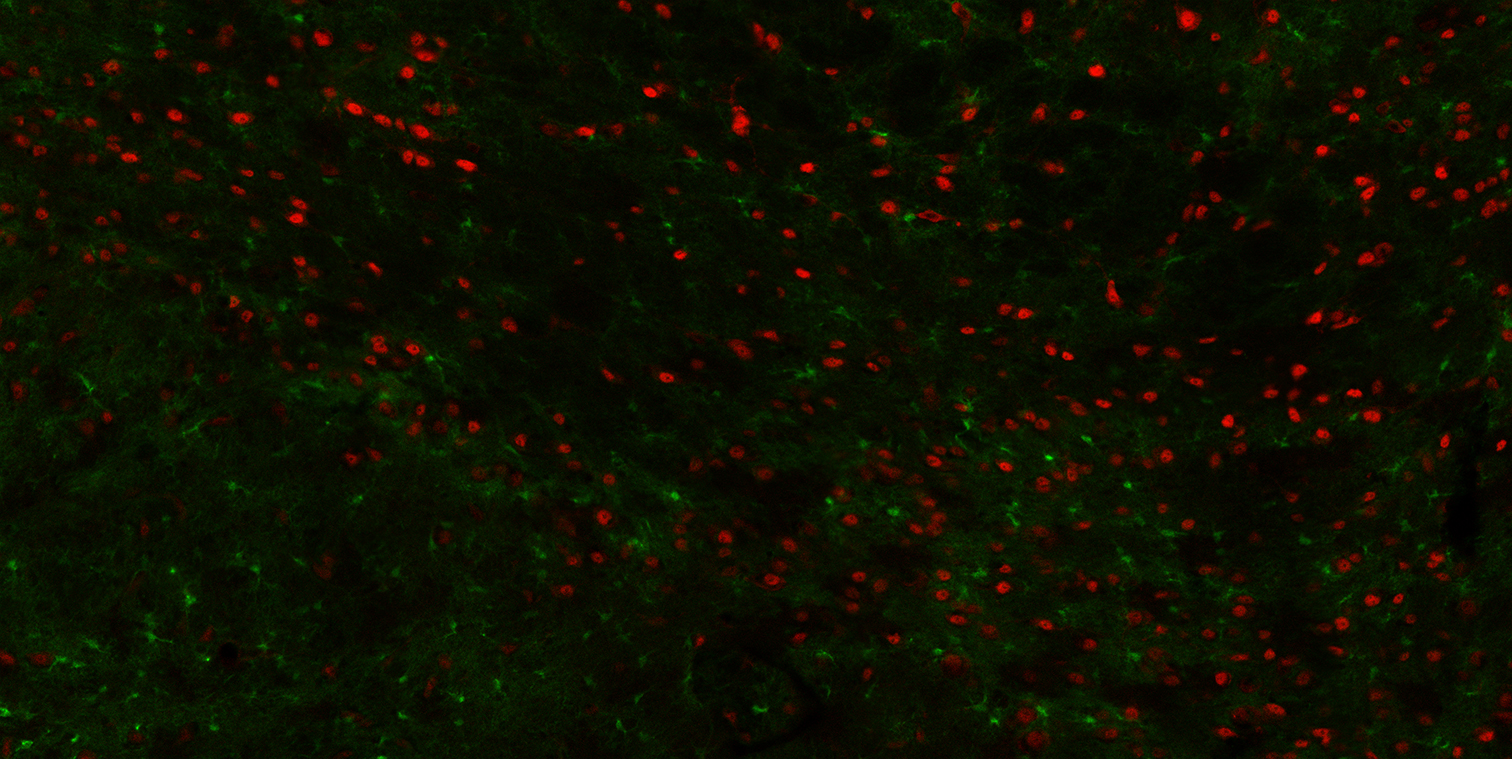

Supplement: Figure 4—source data 1. [file elife-75636-fig4-data1.zip › Fig4 source data 1 for Fig4 B/ASO CTRL #27 YFP+NeuN.jpg]

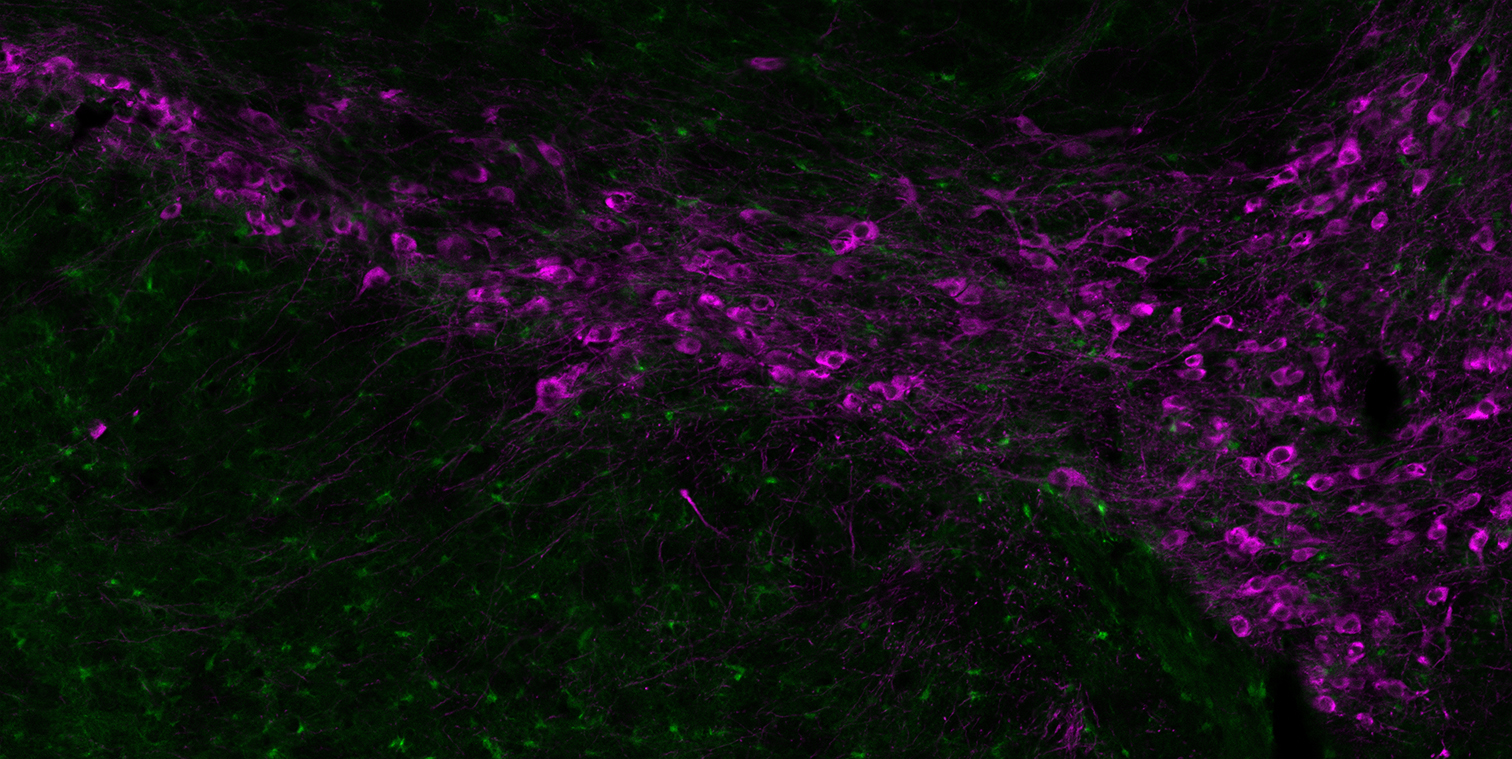

Supplement: Figure 4—source data 1. [file elife-75636-fig4-data1.zip › Fig4 source data 1 for Fig4 B/ASO CTRL #27 YFP+TH.jpg]

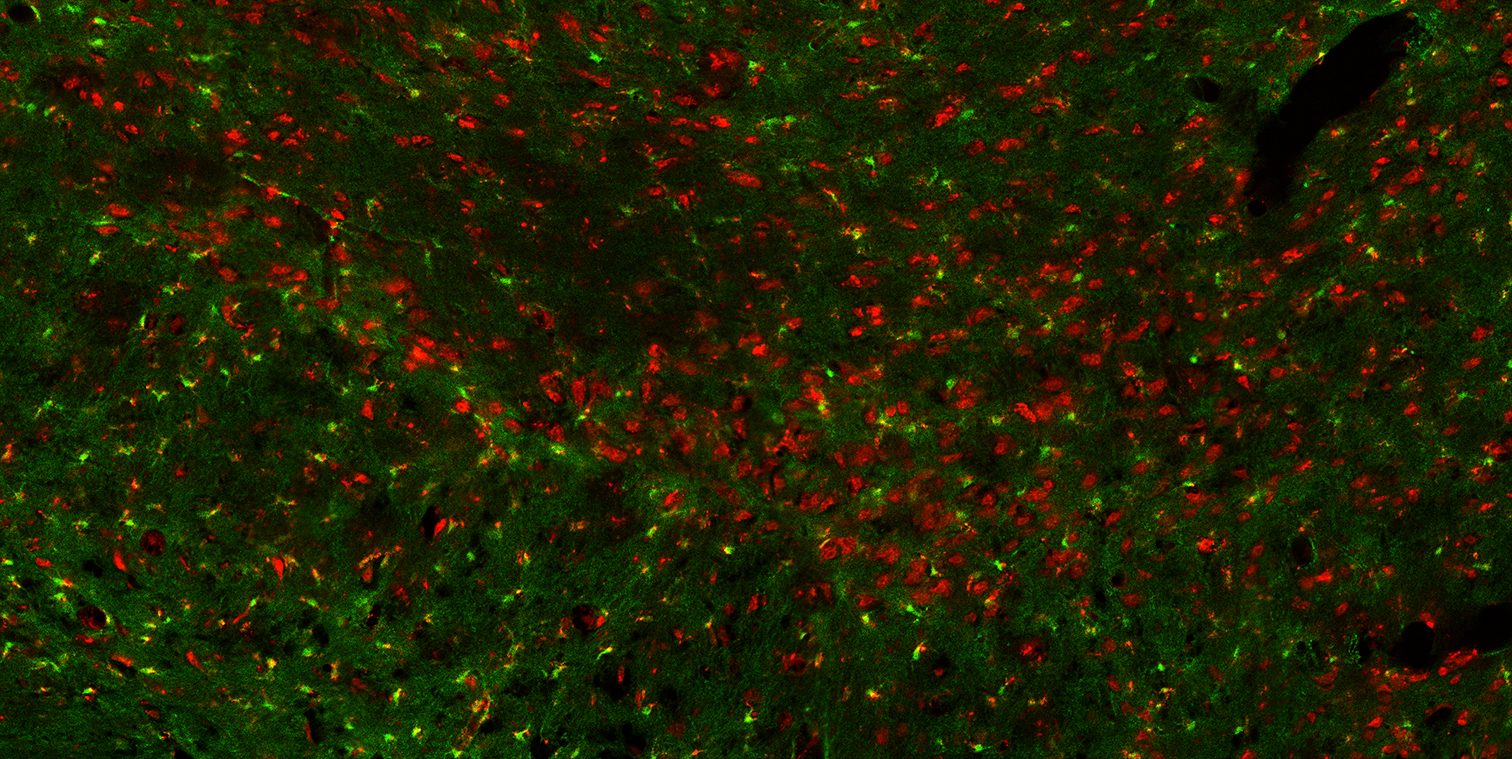

Supplement: Figure 4—source data 1. [file elife-75636-fig4-data1.zip › Fig4 source data 1 for Fig4 B/ASO CTRL #66 YFP+CY3-1.jpg]

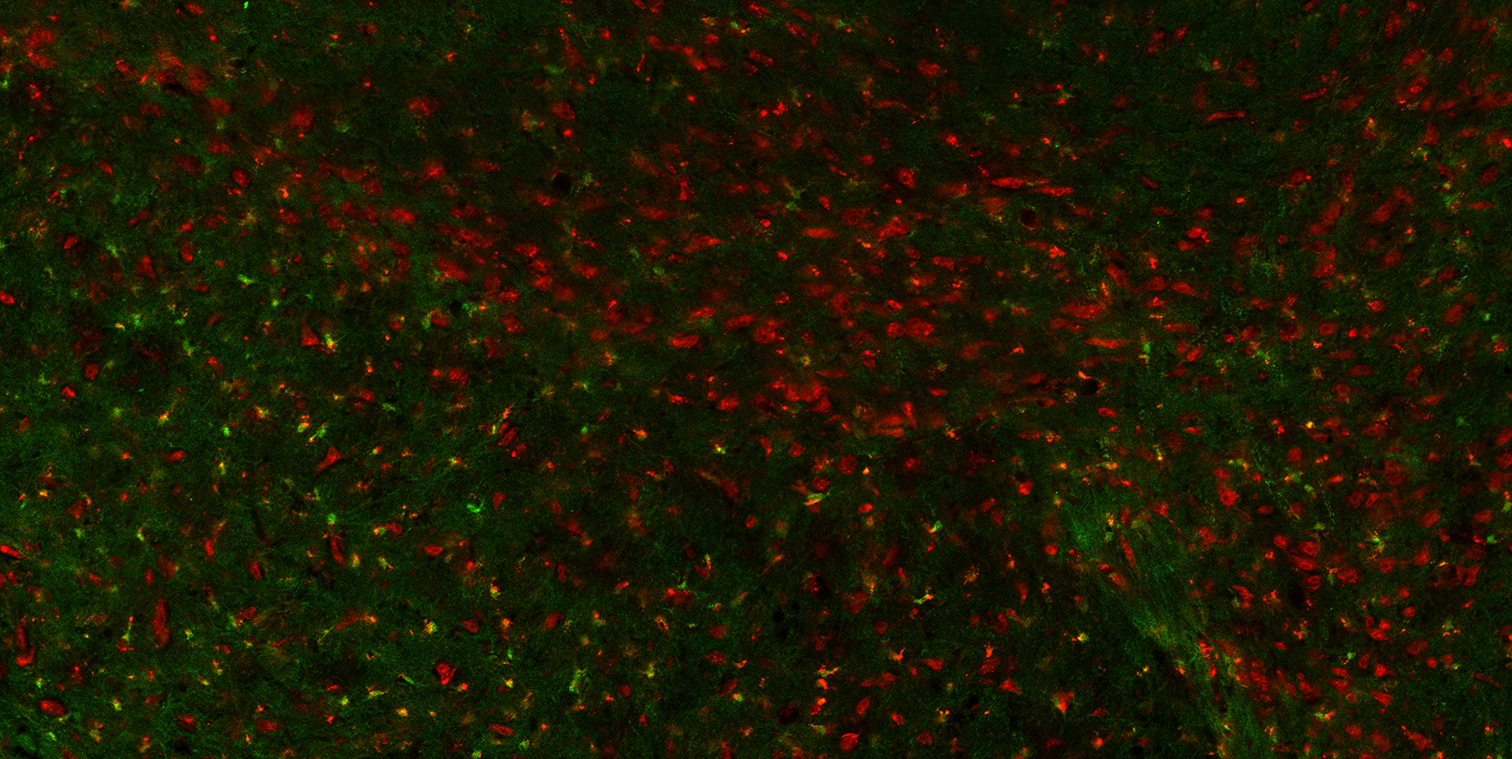

Supplement: Figure 4—source data 1. [file elife-75636-fig4-data1.zip › Fig4 source data 1 for Fig4 B/ASO CTRL #66 YFP+CY3-2.jpg]

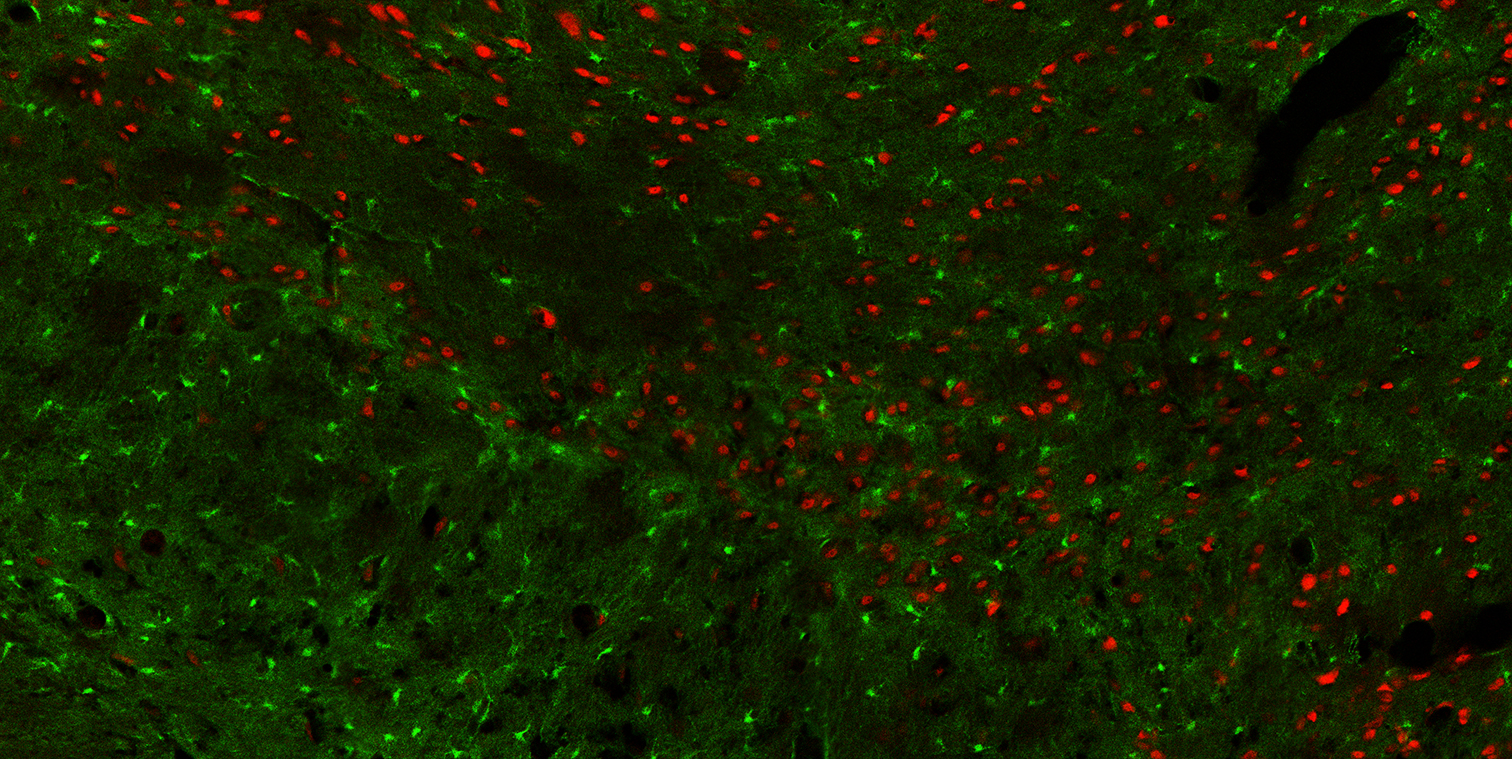

Supplement: Figure 4—source data 1. [file elife-75636-fig4-data1.zip › Fig4 source data 1 for Fig4 B/ASO CTRL #66 YFP+NeuN.jpg]

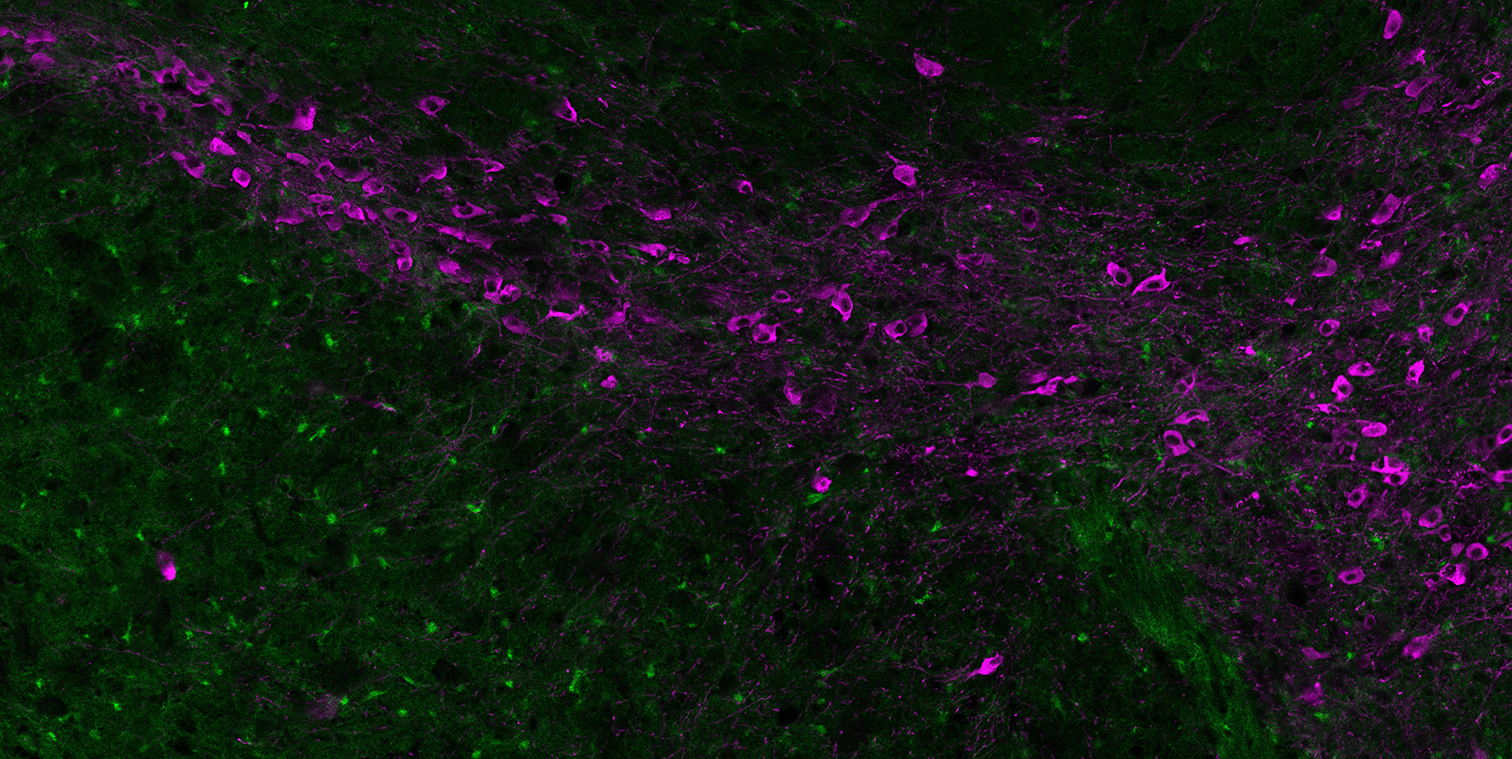

Supplement: Figure 4—source data 1. [file elife-75636-fig4-data1.zip › Fig4 source data 1 for Fig4 B/ASO CTRL #66 YFP+TH.jpg]

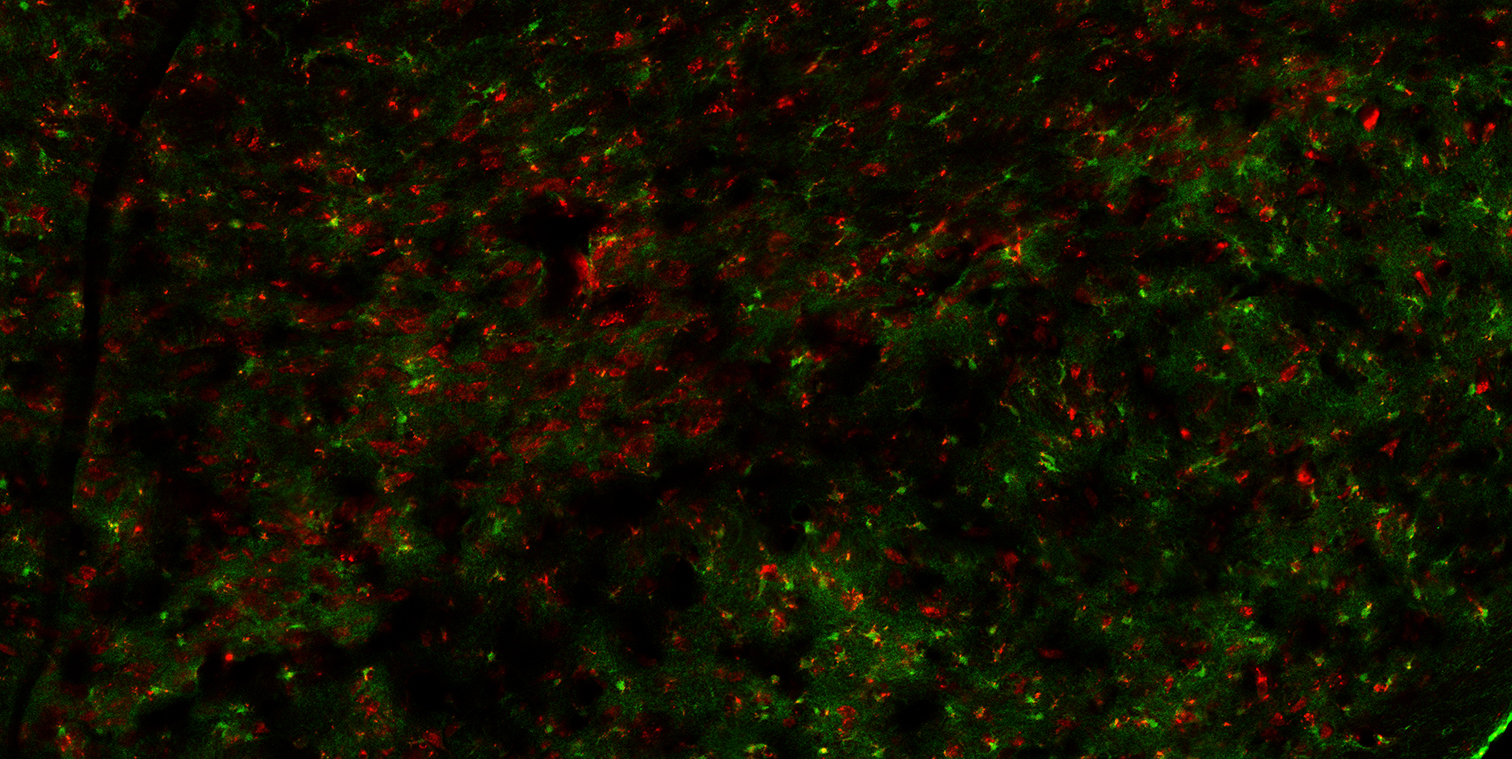

Supplement: Figure 4—source data 1. [file elife-75636-fig4-data1.zip › Fig4 source data 1 for Fig4 B/ASO PTB #68 YFP+CY3-1.jpg]

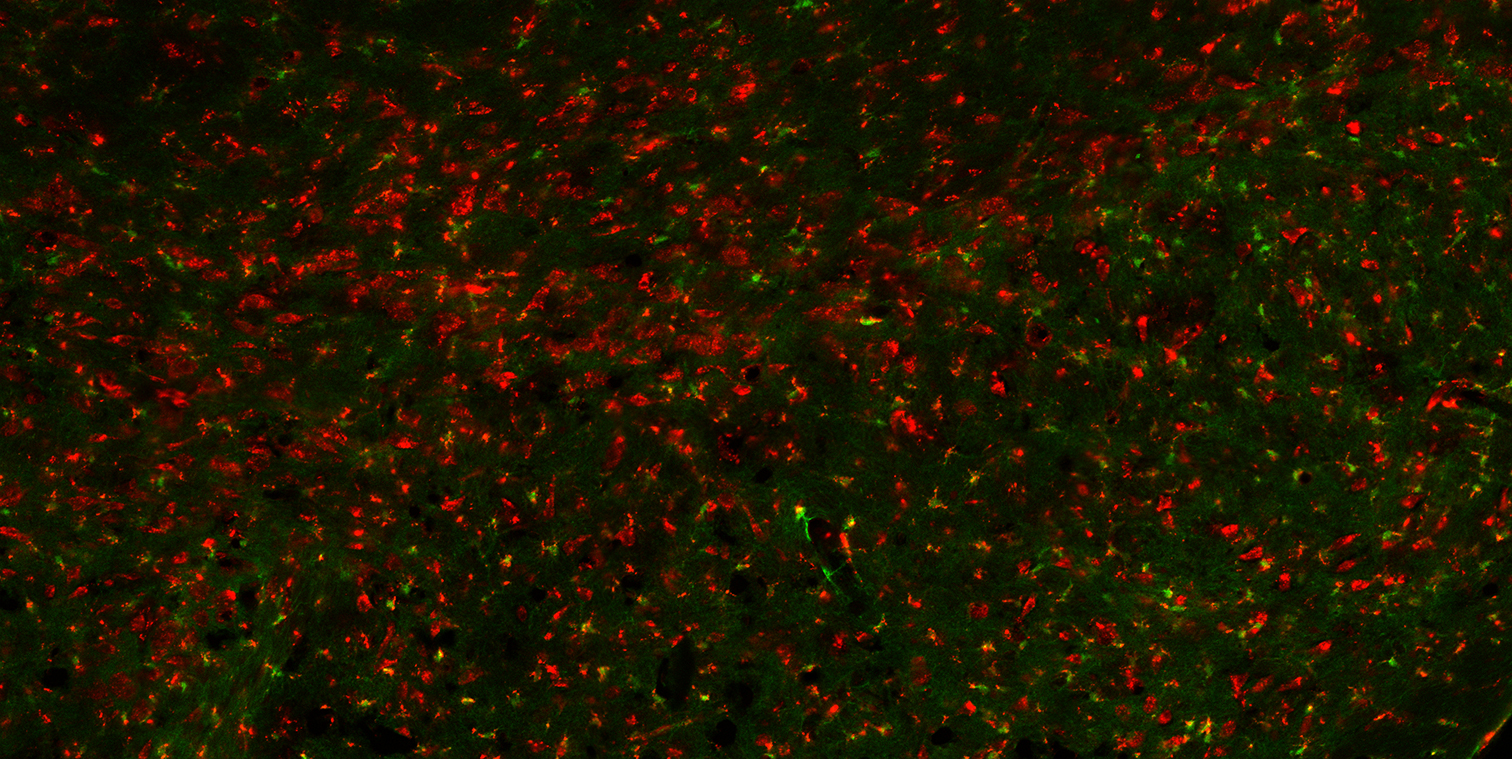

Supplement: Figure 4—source data 1. [file elife-75636-fig4-data1.zip › Fig4 source data 1 for Fig4 B/ASO PTB #68 YFP+CY3-2.jpg]

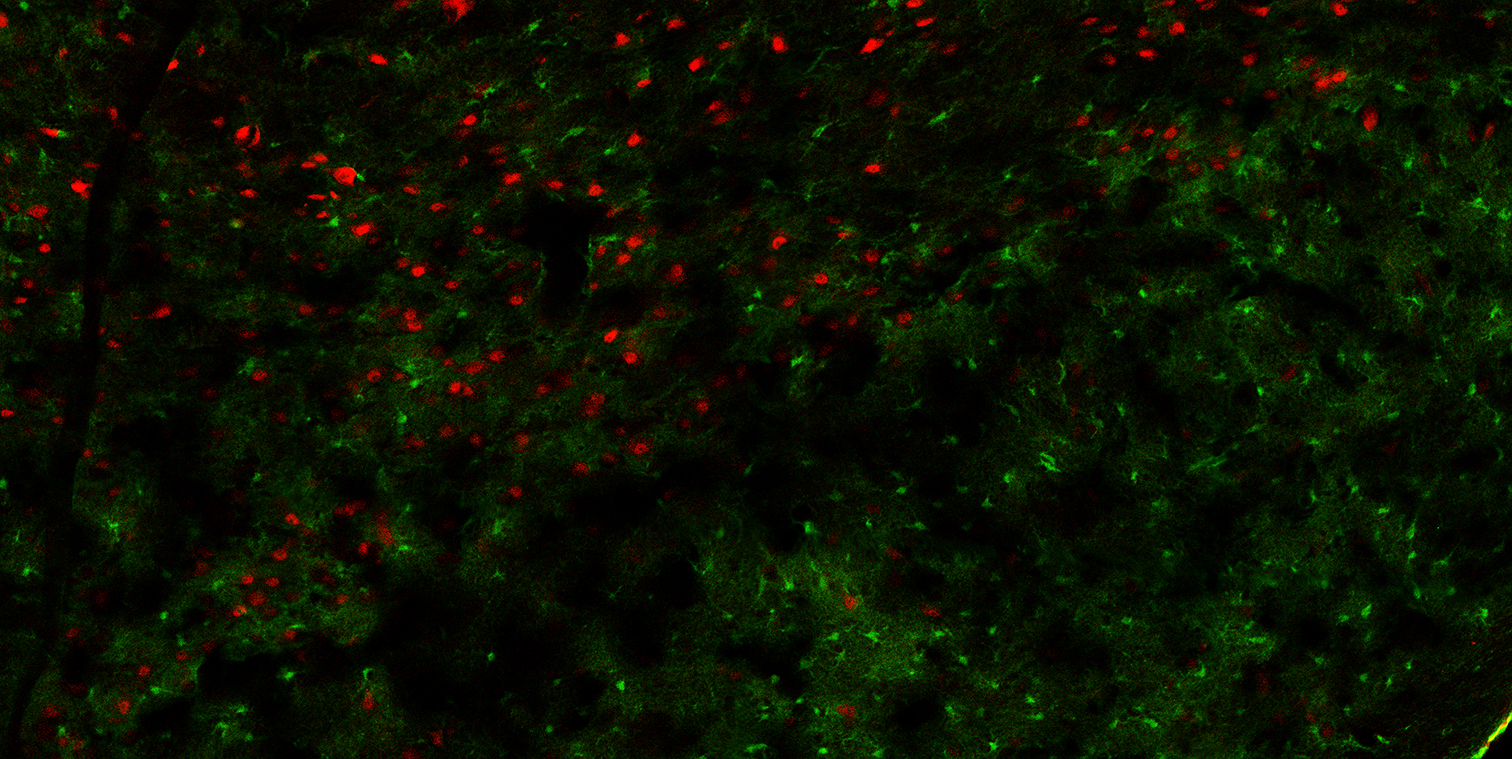

Supplement: Figure 4—source data 1. [file elife-75636-fig4-data1.zip › Fig4 source data 1 for Fig4 B/ASO PTB #68 YFP+NeuN.jpg]

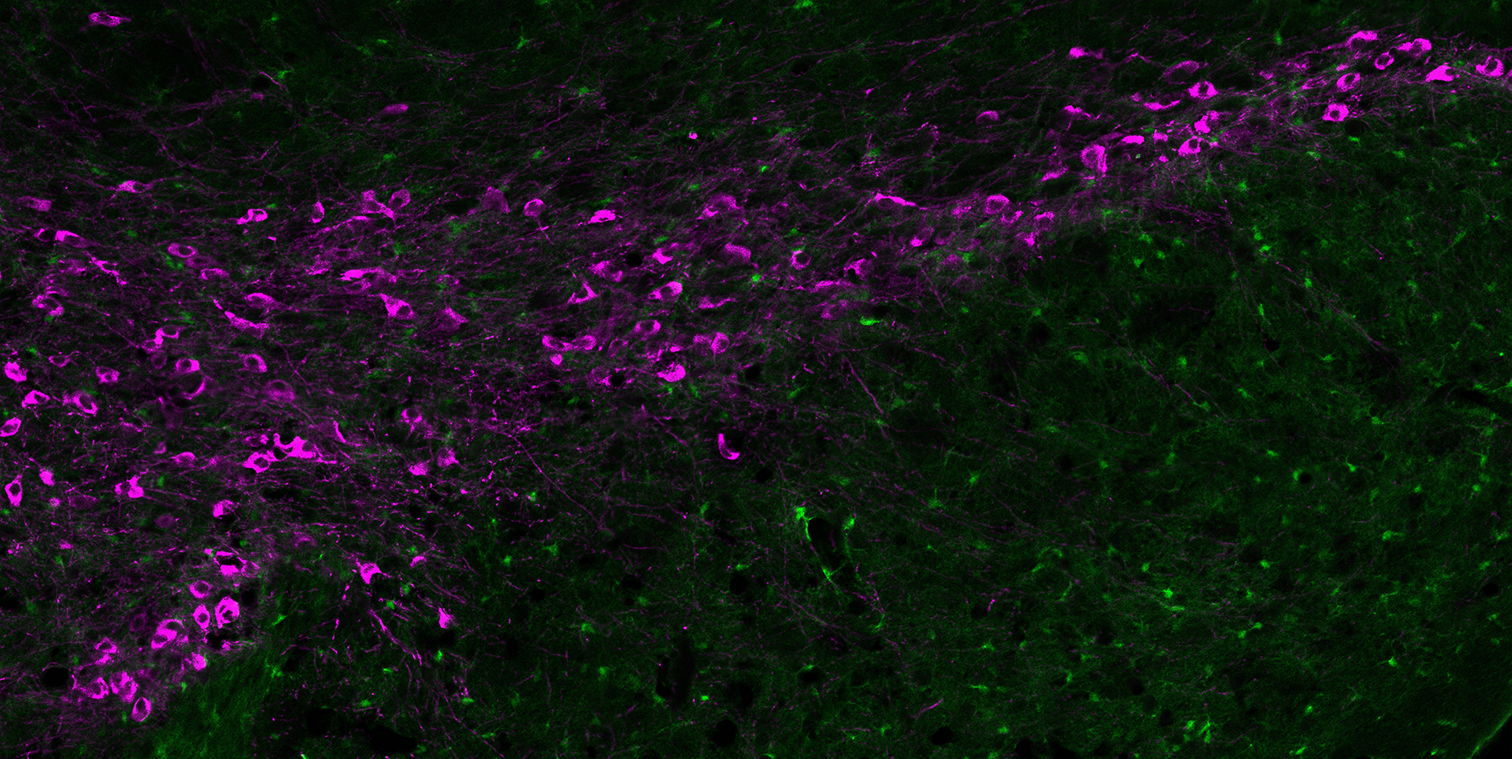

Supplement: Figure 4—source data 1. [file elife-75636-fig4-data1.zip › Fig4 source data 1 for Fig4 B/ASO PTB #68 YFP+TH.jpg]

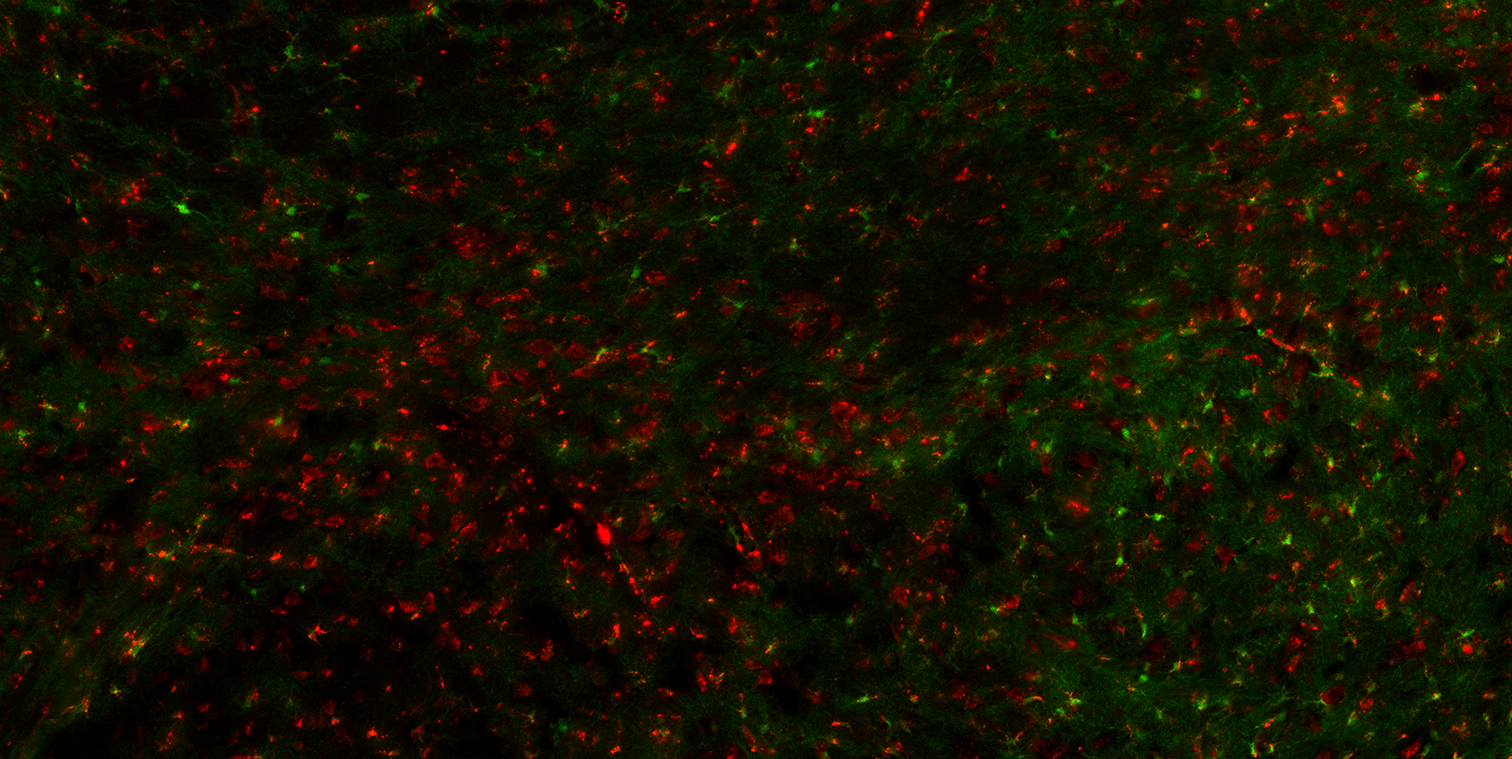

Supplement: Figure 4—source data 1. [file elife-75636-fig4-data1.zip › Fig4 source data 1 for Fig4 B/ASO PTB #69 YFP+CY3-1.jpg]

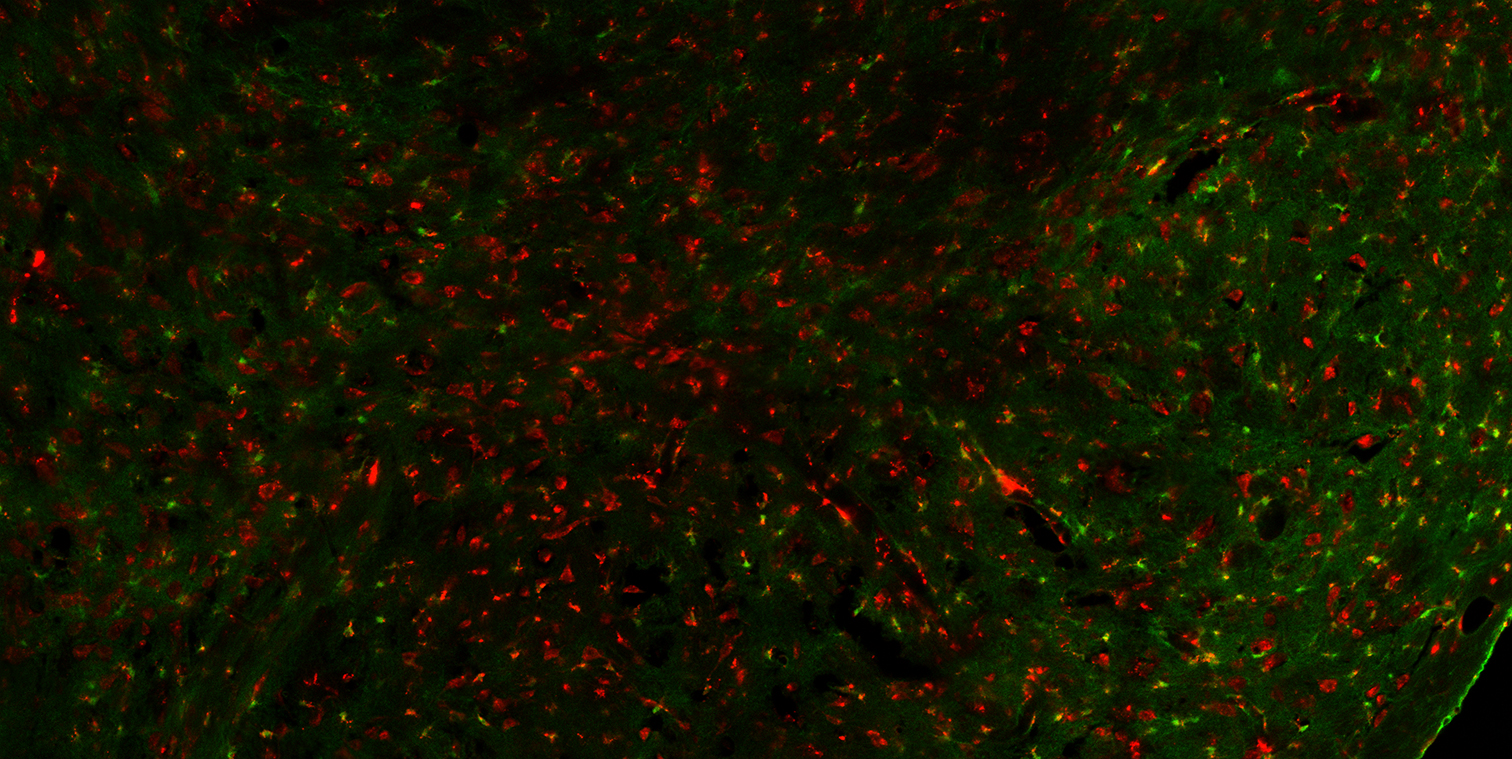

Supplement: Figure 4—source data 1. [file elife-75636-fig4-data1.zip › Fig4 source data 1 for Fig4 B/ASO PTB #69 YFP+CY3-2.jpg]

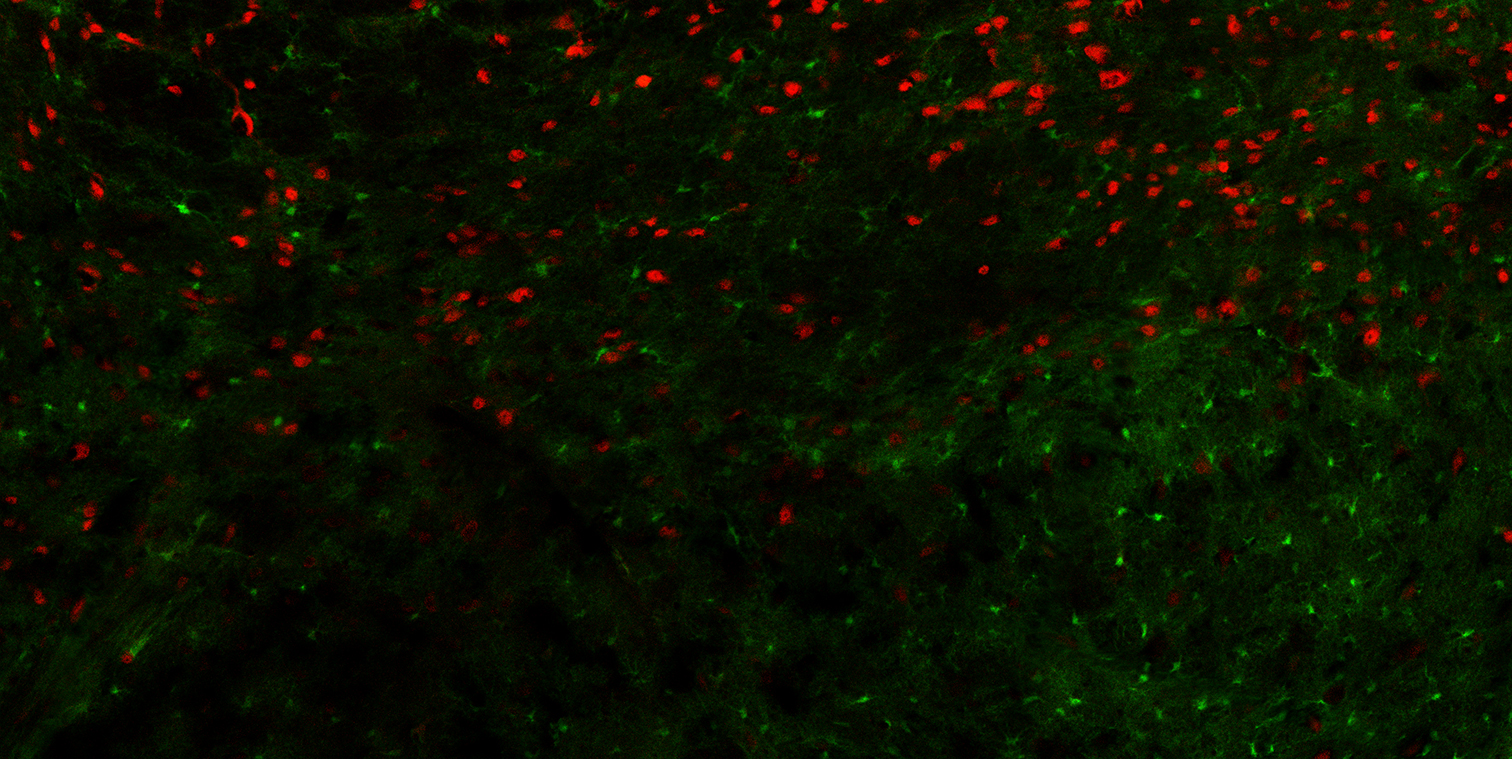

Supplement: Figure 4—source data 1. [file elife-75636-fig4-data1.zip › Fig4 source data 1 for Fig4 B/ASO PTB #69 YFP+NeuN.jpg]
