# Supplementary figures and images for: Repressing PTBP1 fails to convert reactive astrocytes to dopaminergic neurons in a 6-hydroxydopamine mouse model of Parkinson’s disease (part 4 of 4)
Source: eLife. 2022 May 10;11:e75636. doi: 10.7554/eLife.75636 (PMC9208759; doi:10.7554/eLife.75636)

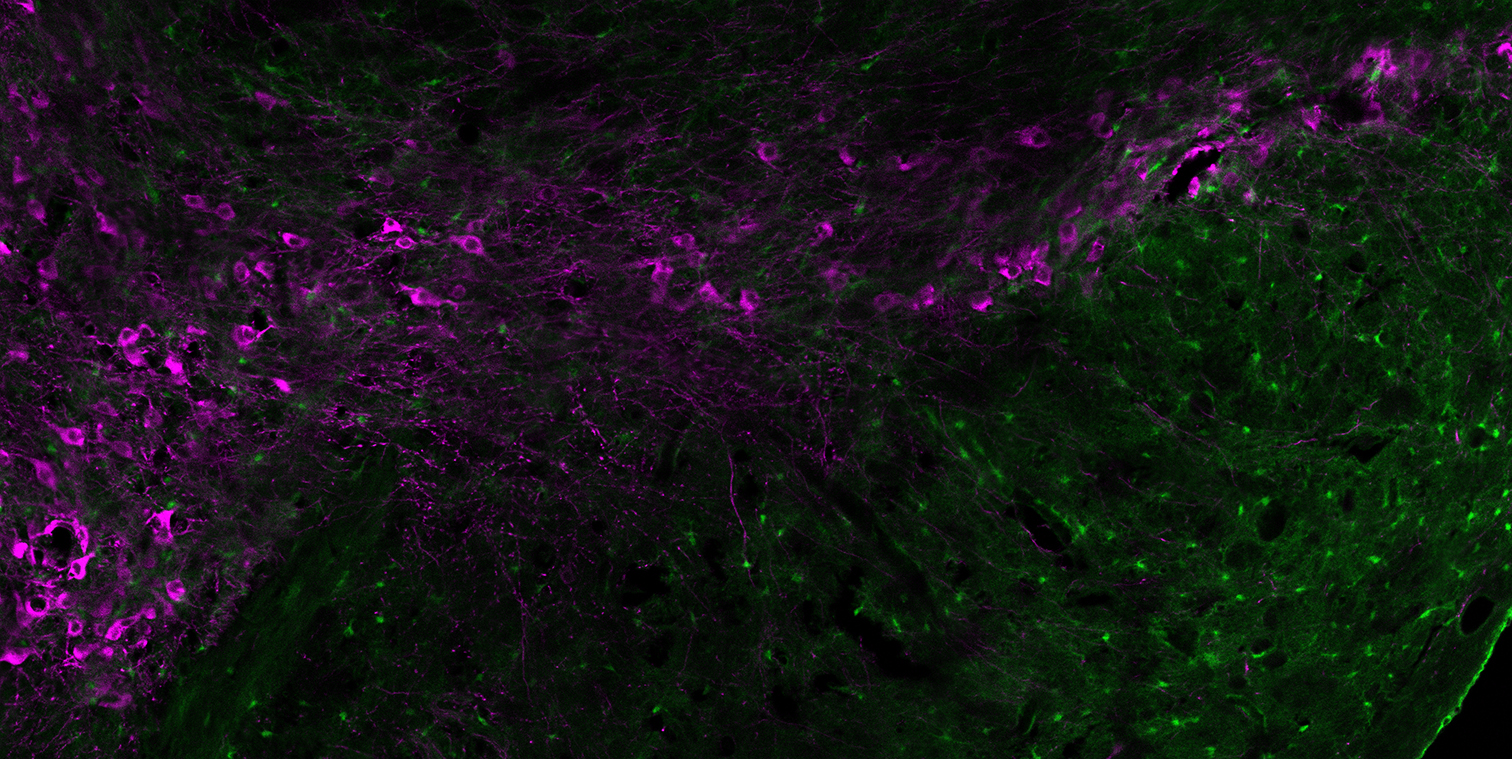

Supplement: Figure 4—source data 1. [file elife-75636-fig4-data1.zip › Fig4 source data 1 for Fig4 B/ASO PTB #69 YFP+TH.jpg]

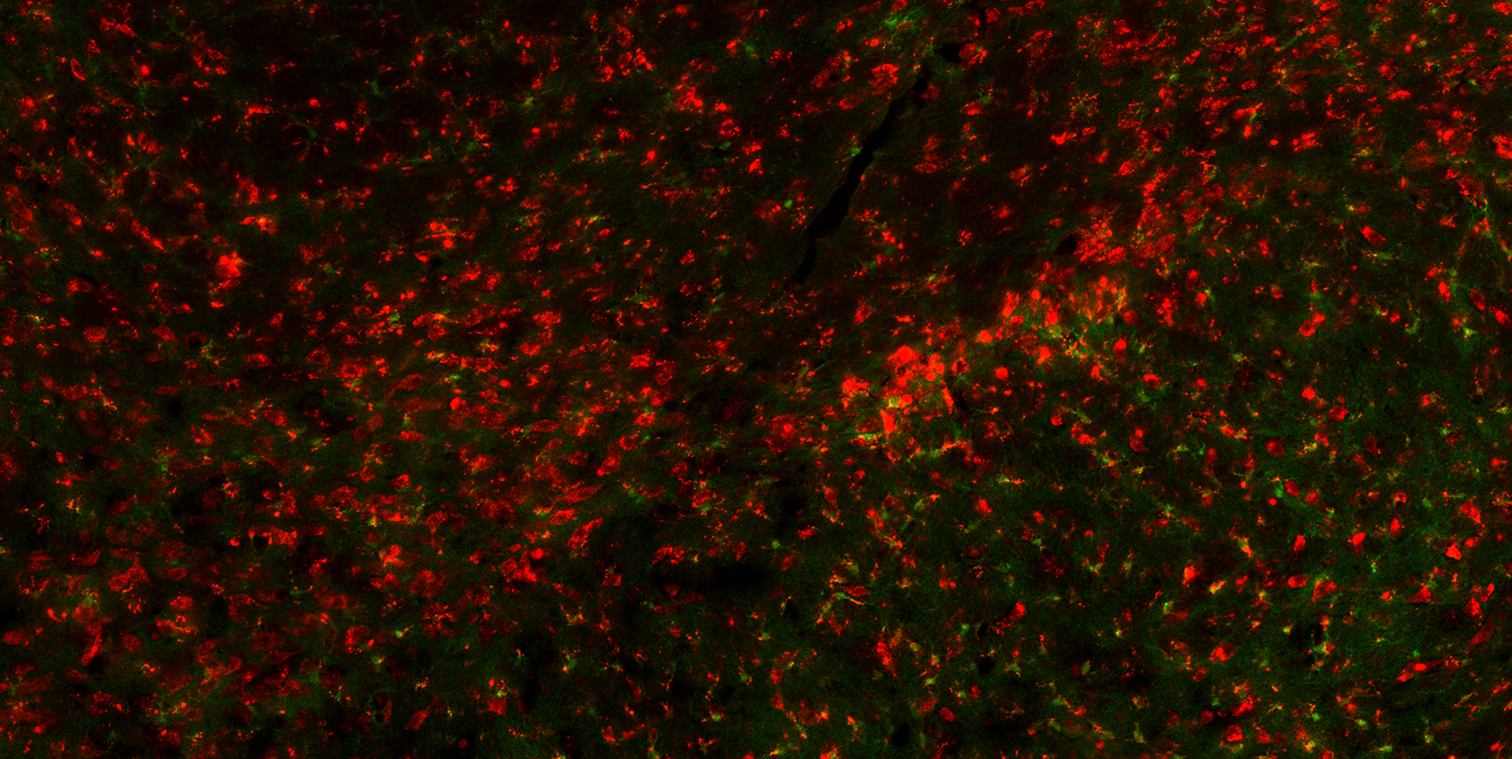

Supplement: Figure 4—source data 1. [file elife-75636-fig4-data1.zip › Fig4 source data 1 for Fig4 B/ASO PTB #70 YFP+CY3-1.jpg]

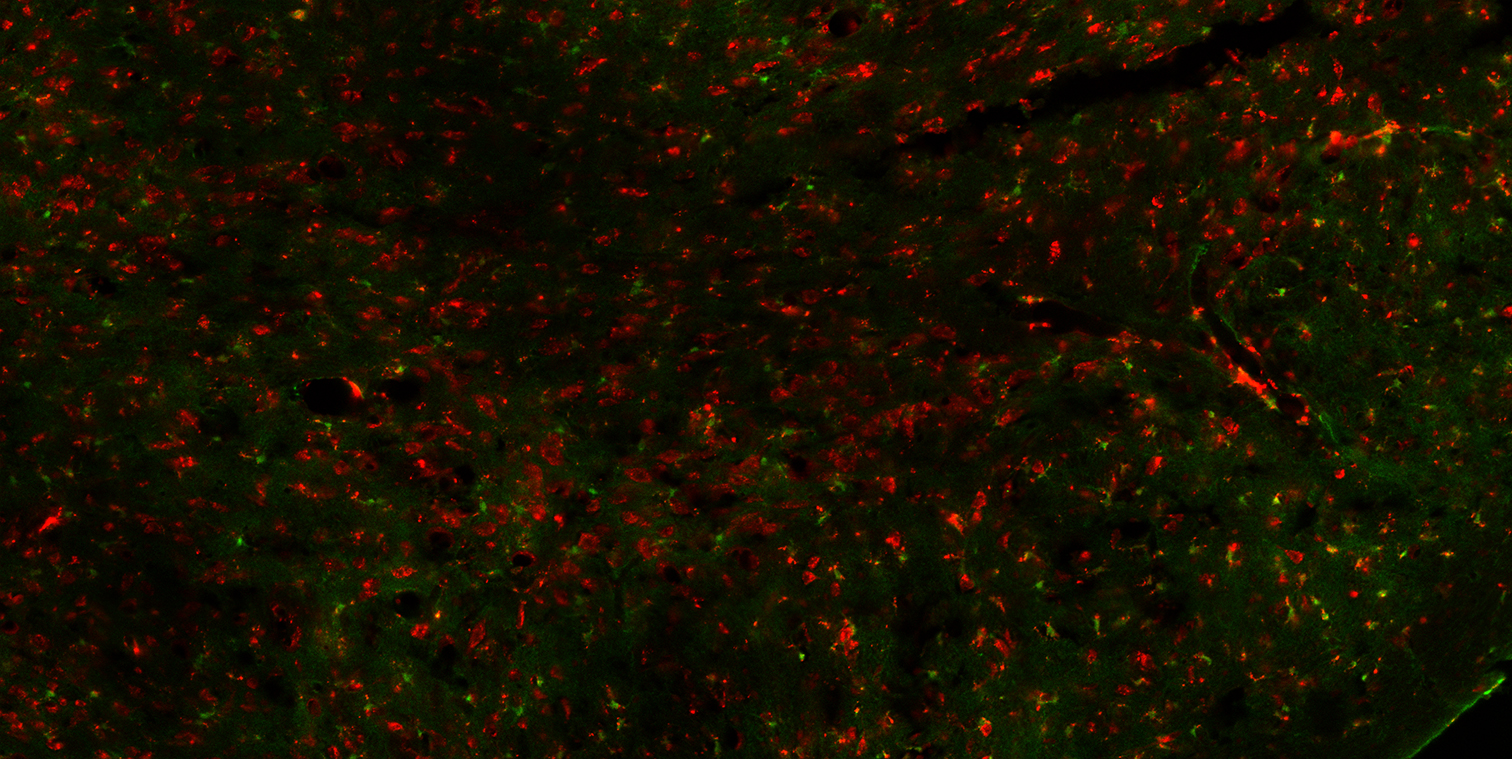

Supplement: Figure 4—source data 1. [file elife-75636-fig4-data1.zip › Fig4 source data 1 for Fig4 B/ASO PTB #70 YFP+CY3-2.jpg]

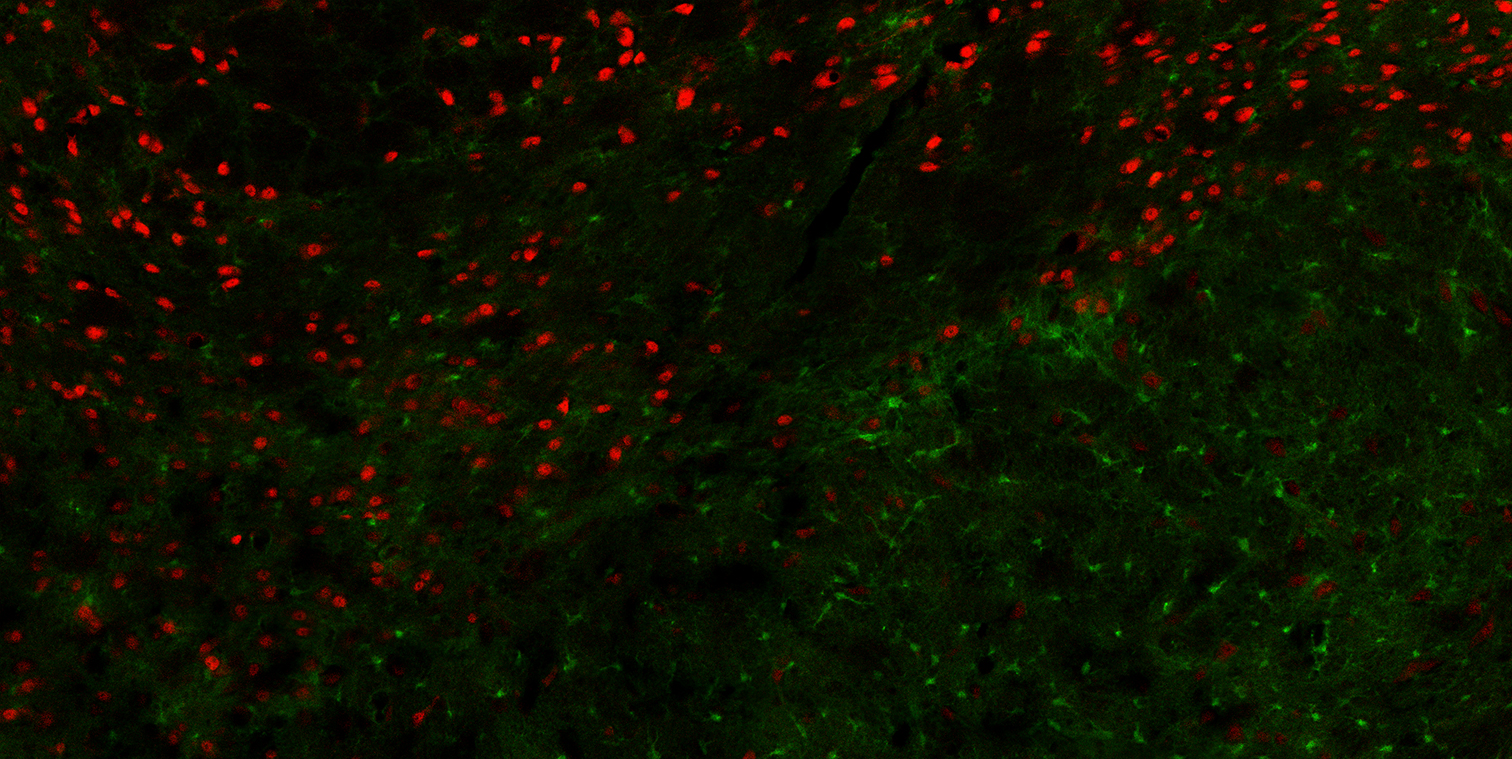

Supplement: Figure 4—source data 1. [file elife-75636-fig4-data1.zip › Fig4 source data 1 for Fig4 B/ASO PTB #70 YFP+NeuN.jpg]

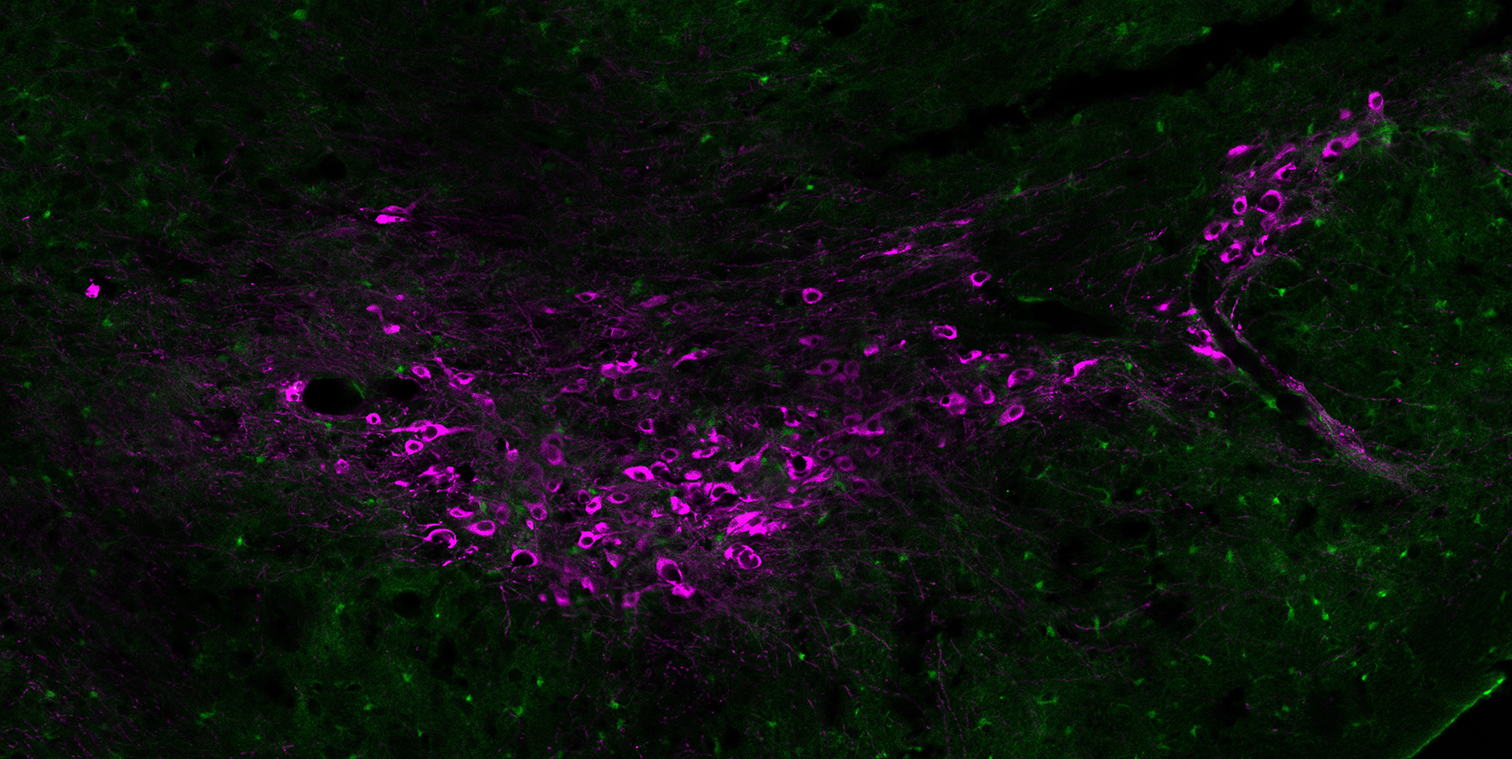

Supplement: Figure 4—source data 1. [file elife-75636-fig4-data1.zip › Fig4 source data 1 for Fig4 B/ASO PTB #70 YFP+TH.jpg]

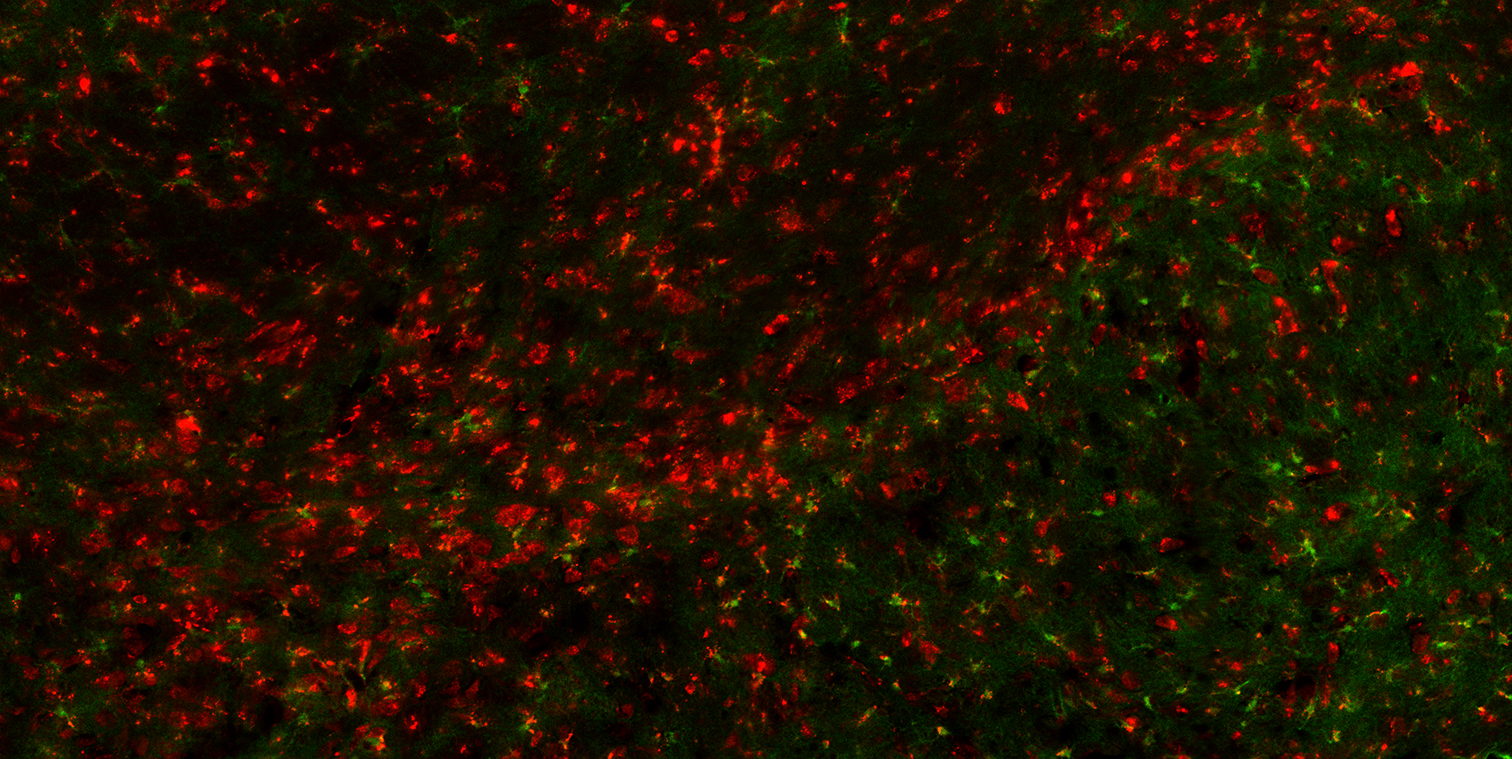

Supplement: Figure 4—source data 1. [file elife-75636-fig4-data1.zip › Fig4 source data 1 for Fig4 B/ASO PTB #71 YFP+CY3-1.jpg]

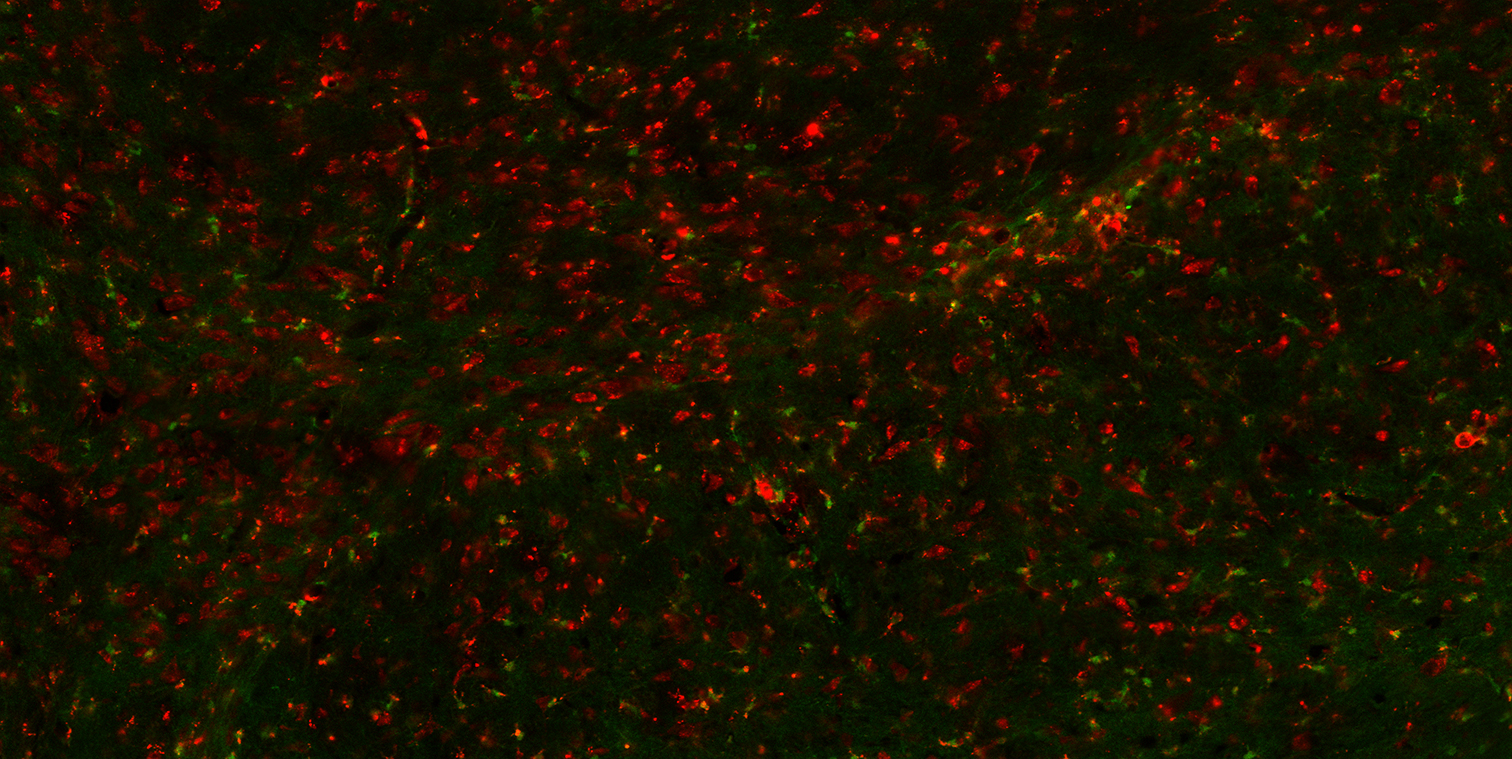

Supplement: Figure 4—source data 1. [file elife-75636-fig4-data1.zip › Fig4 source data 1 for Fig4 B/ASO PTB #71 YFP+CY3-2.jpg]

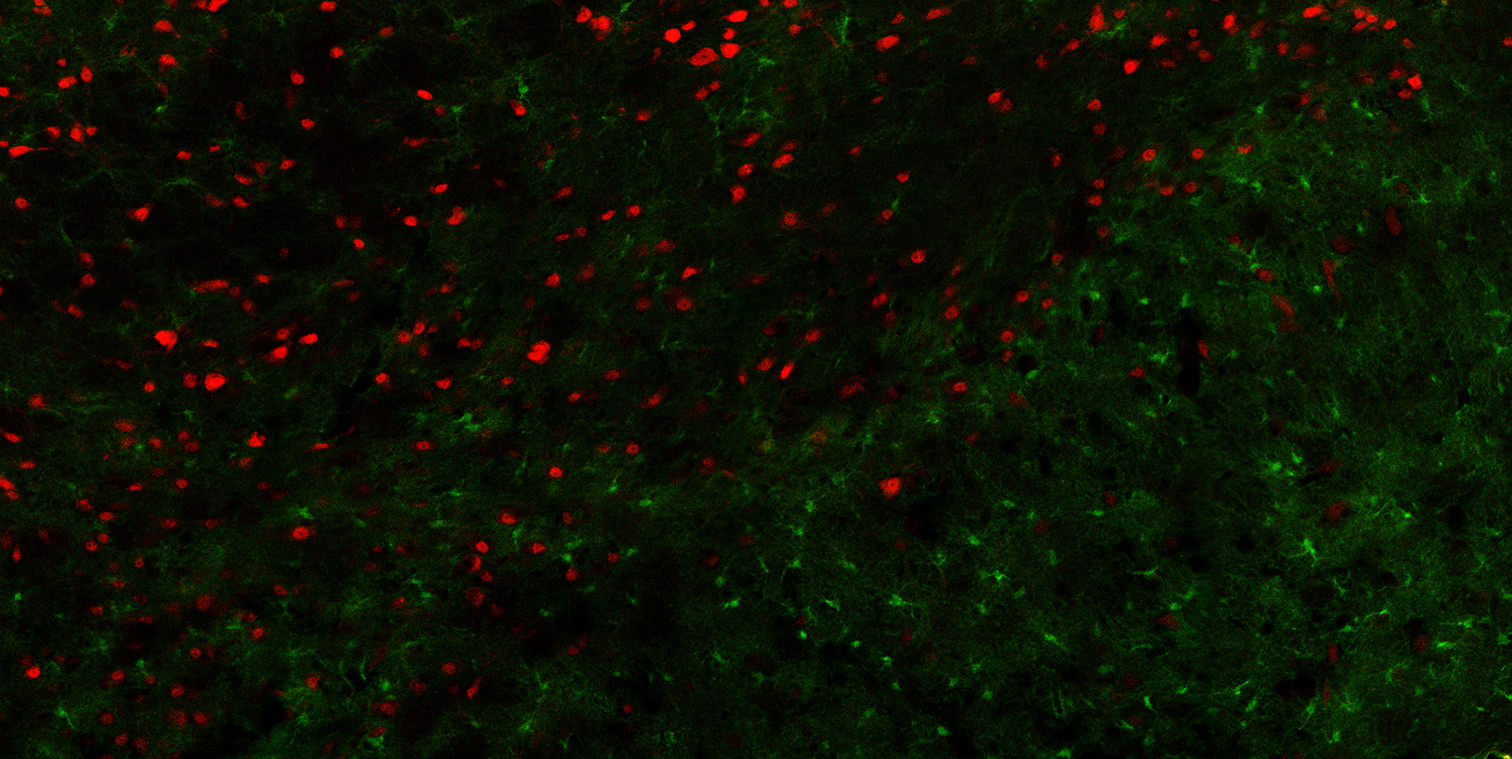

Supplement: Figure 4—source data 1. [file elife-75636-fig4-data1.zip › Fig4 source data 1 for Fig4 B/ASO PTB #71 YFP+NeuN.jpg]

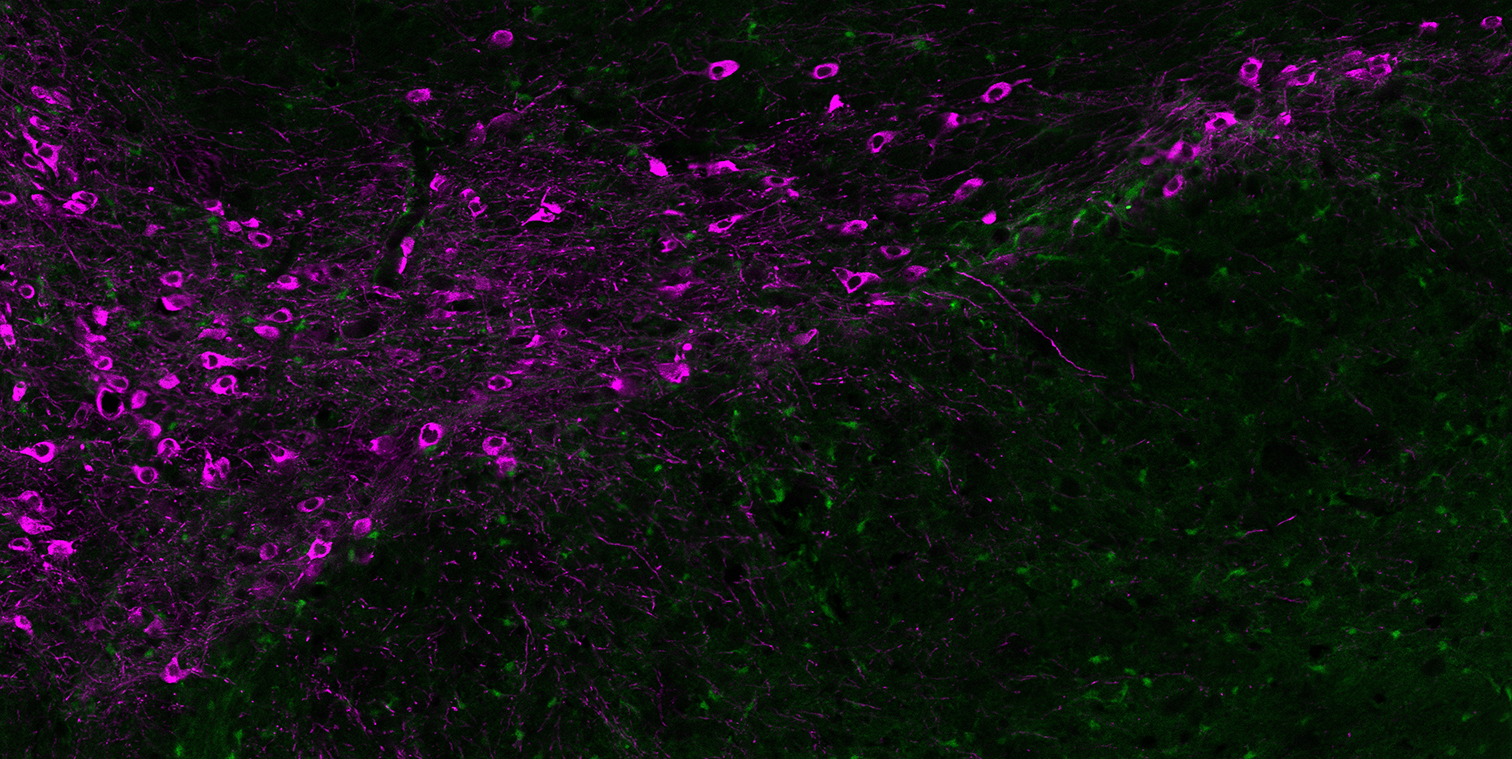

Supplement: Figure 4—source data 1. [file elife-75636-fig4-data1.zip › Fig4 source data 1 for Fig4 B/ASO PTB #71 YFP+TH.jpg]

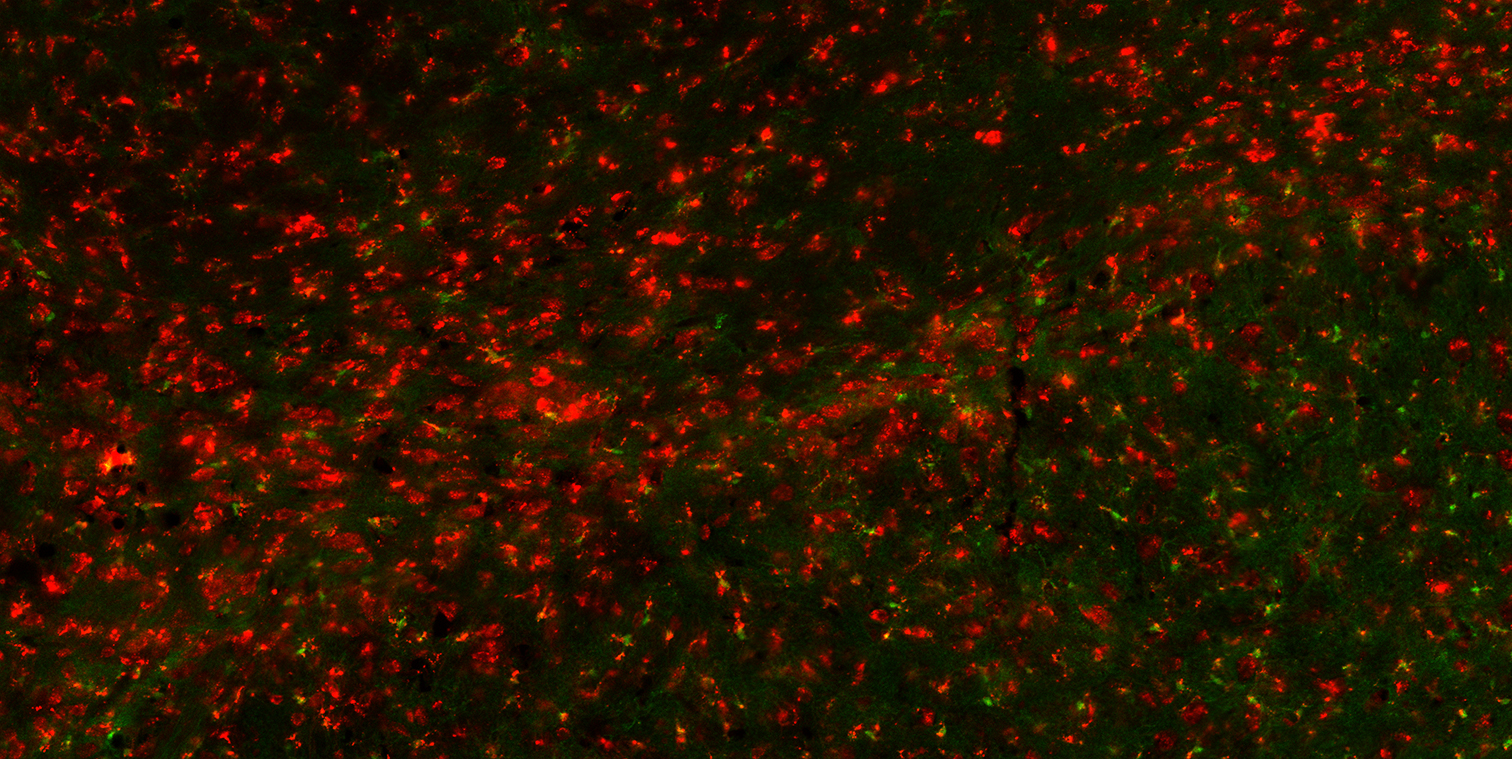

Supplement: Figure 4—source data 1. [file elife-75636-fig4-data1.zip › Fig4 source data 1 for Fig4 B/ASO PTB #72 YFP+CY3-1.jpg]

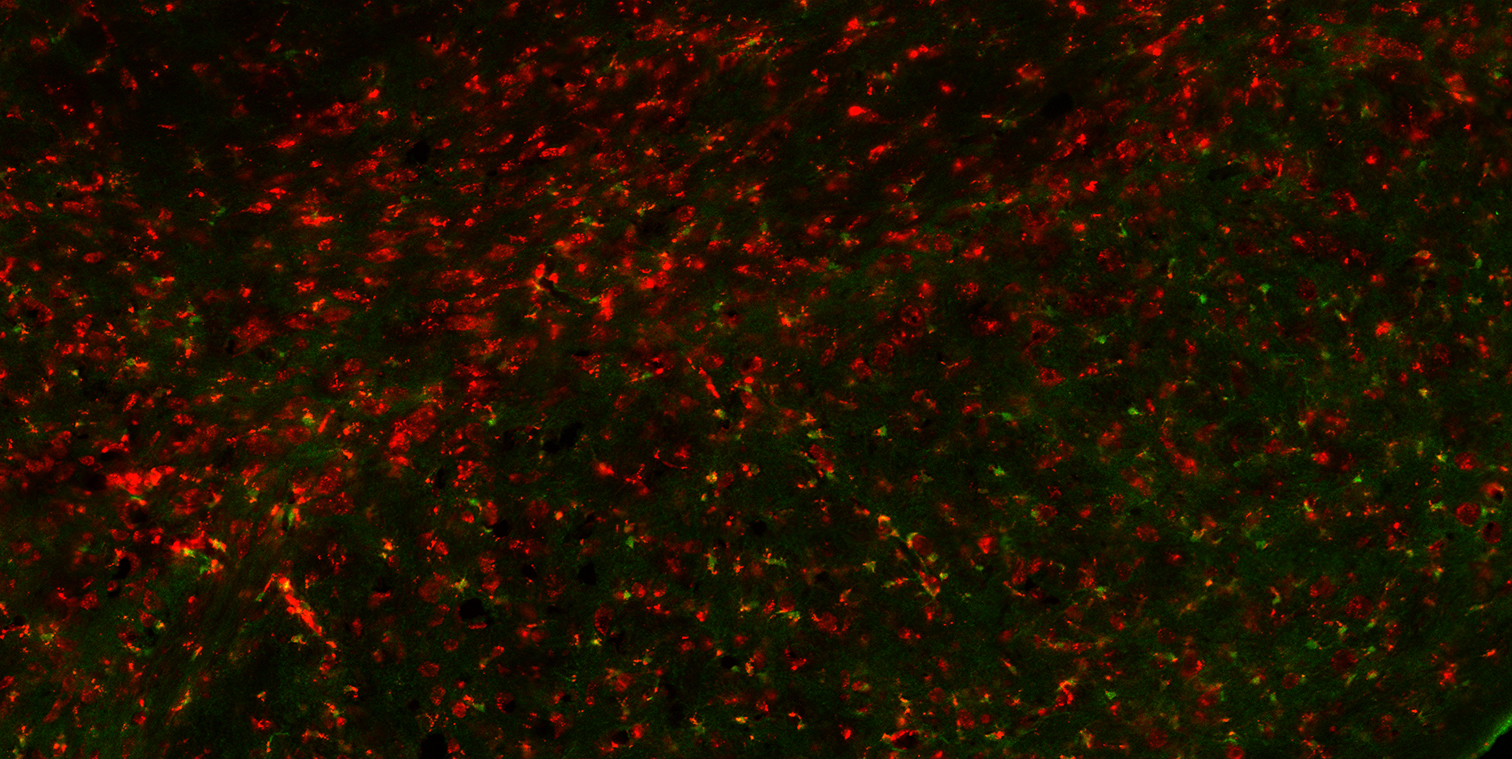

Supplement: Figure 4—source data 1. [file elife-75636-fig4-data1.zip › Fig4 source data 1 for Fig4 B/ASO PTB #72 YFP+CY3-2.jpg]

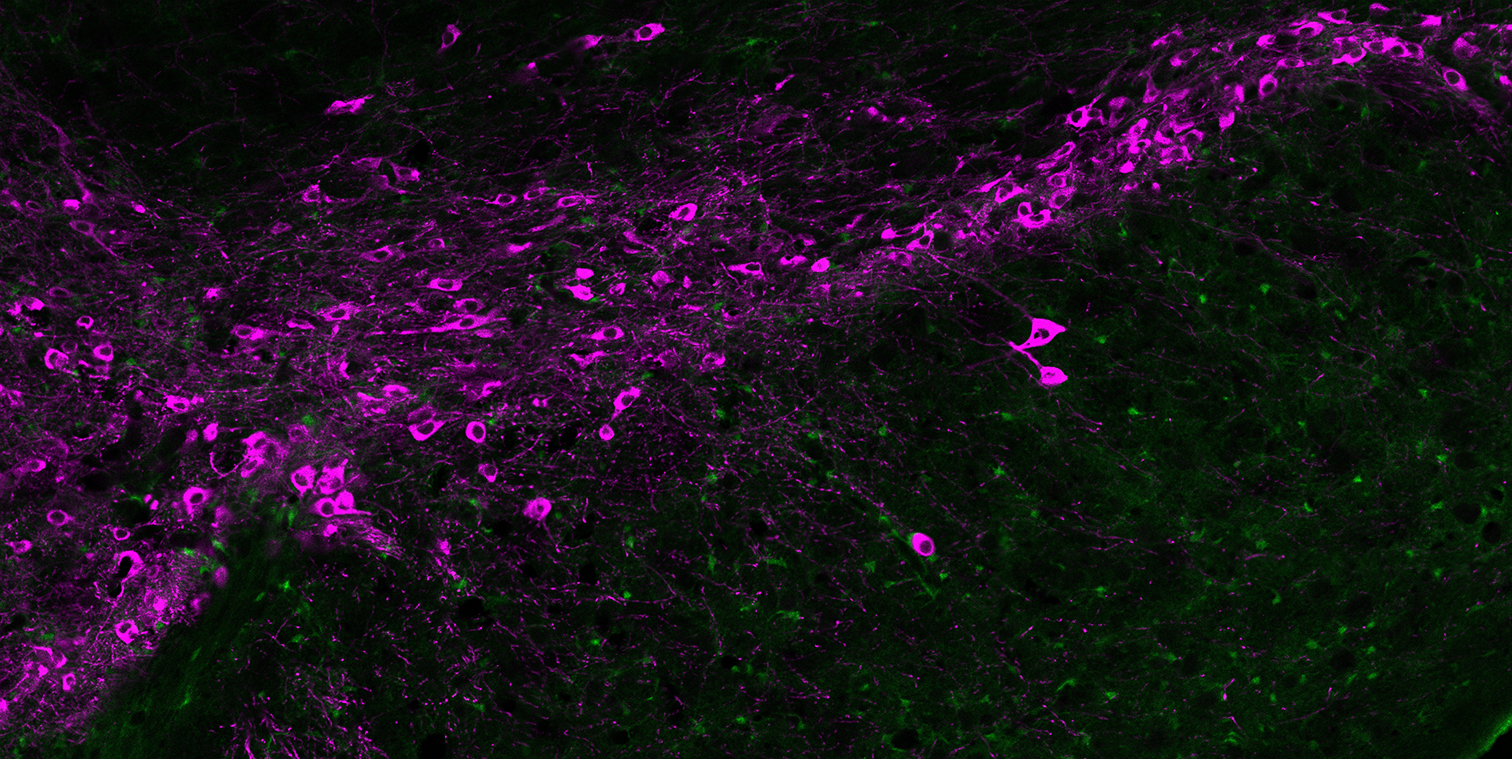

Supplement: Figure 4—source data 1. [file elife-75636-fig4-data1.zip › Fig4 source data 1 for Fig4 B/ASO PTB #72 YFP+TH.jpg]

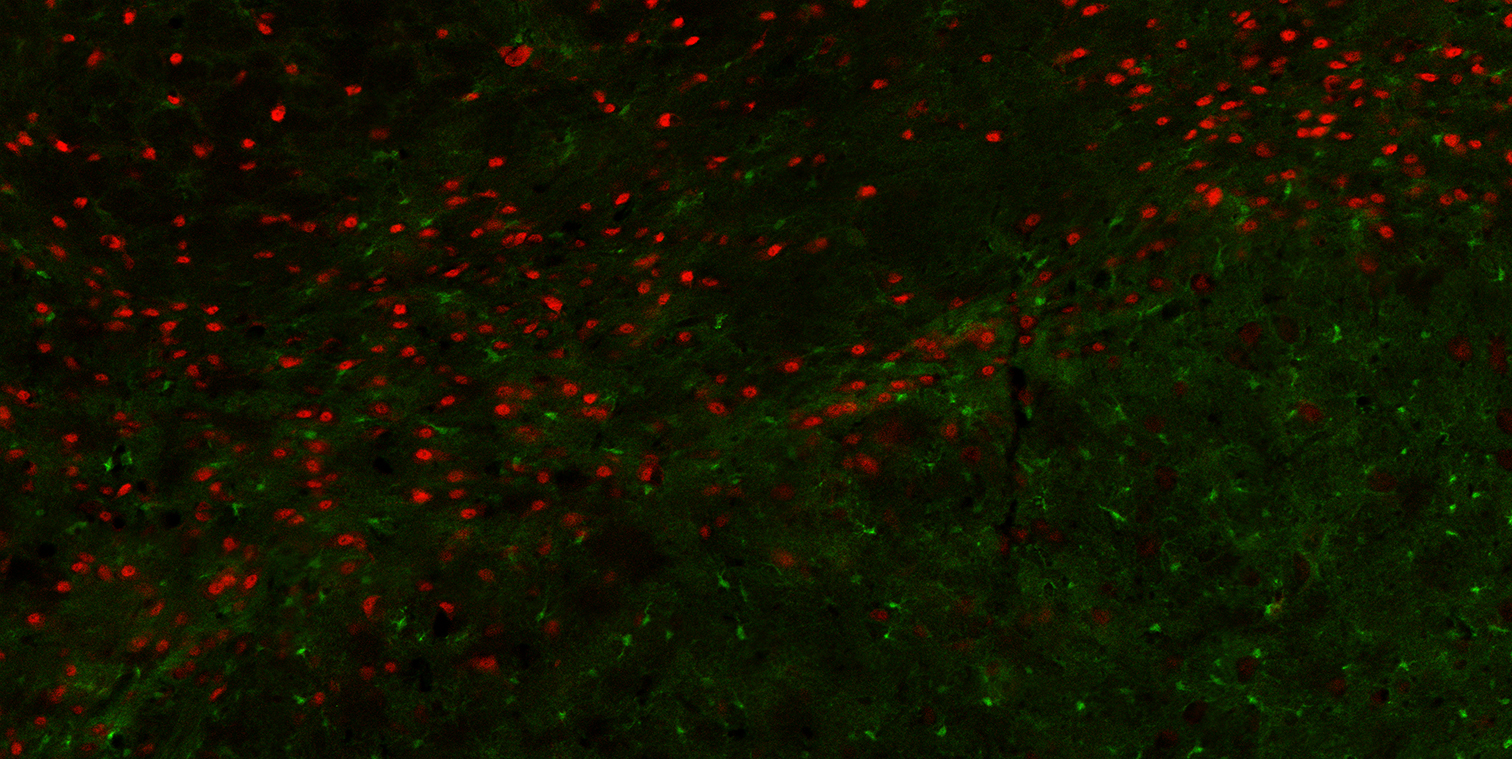

Supplement: Figure 4—source data 1. [file elife-75636-fig4-data1.zip › Fig4 source data 1 for Fig4 B/ASO PTB #73 YFP+NeuN.jpg]

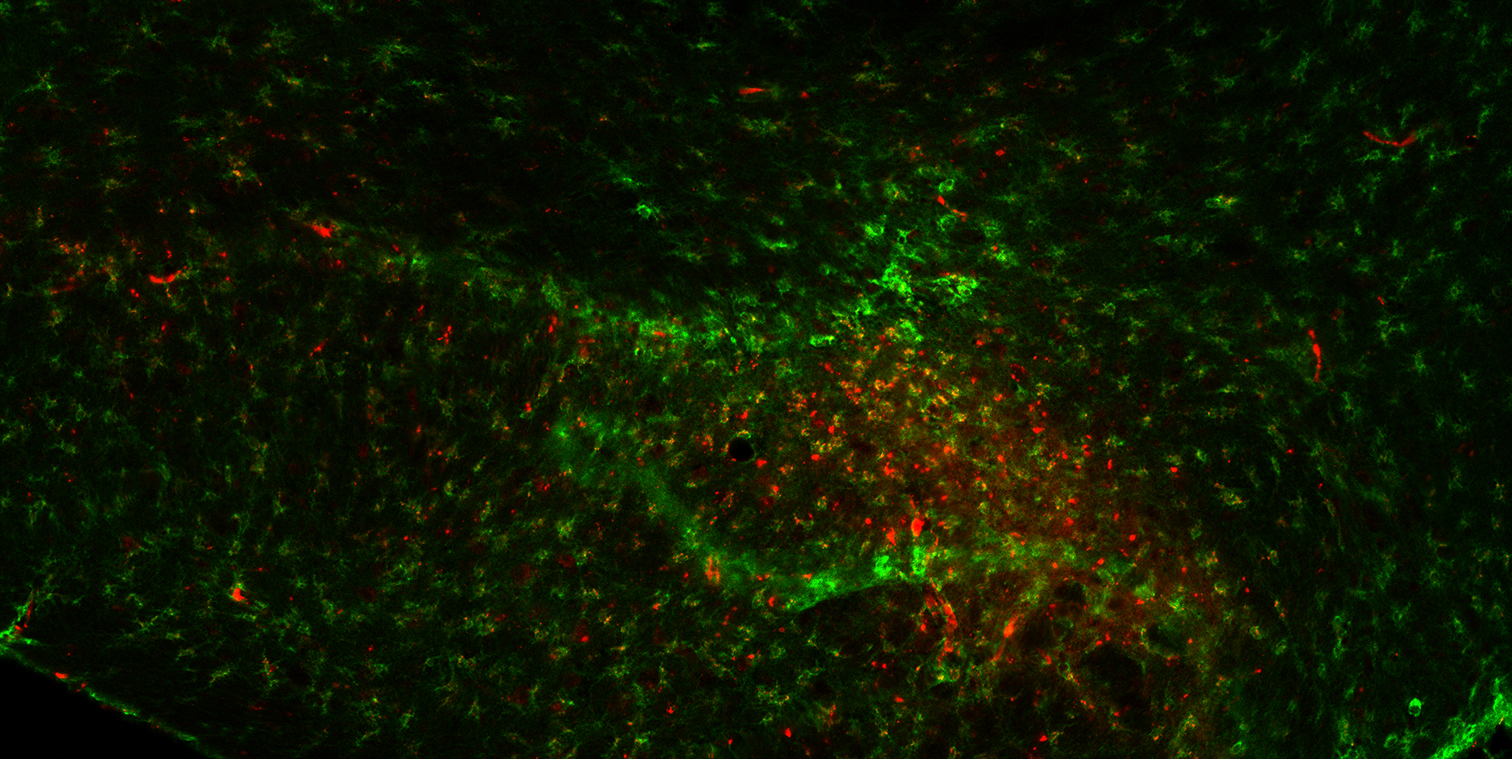

Supplement: Figure 4—source data 2. [file elife-75636-fig4-data2.zip › Fig4 source data 2 for Fig4 D/ASO CTRL #54 HA+CY3-1.jpg]

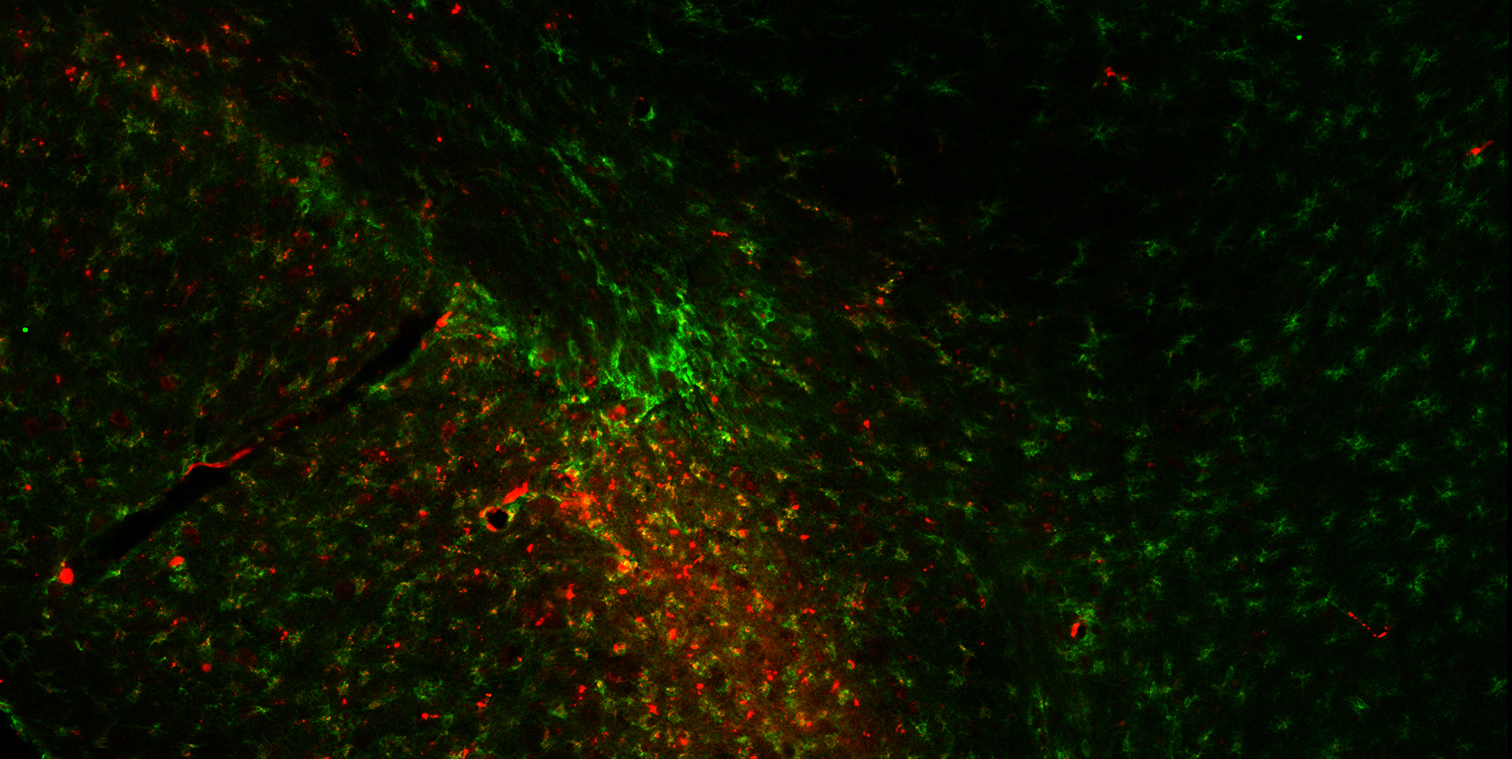

Supplement: Figure 4—source data 2. [file elife-75636-fig4-data2.zip › Fig4 source data 2 for Fig4 D/ASO CTRL #54 HA+CY3-2.jpg]

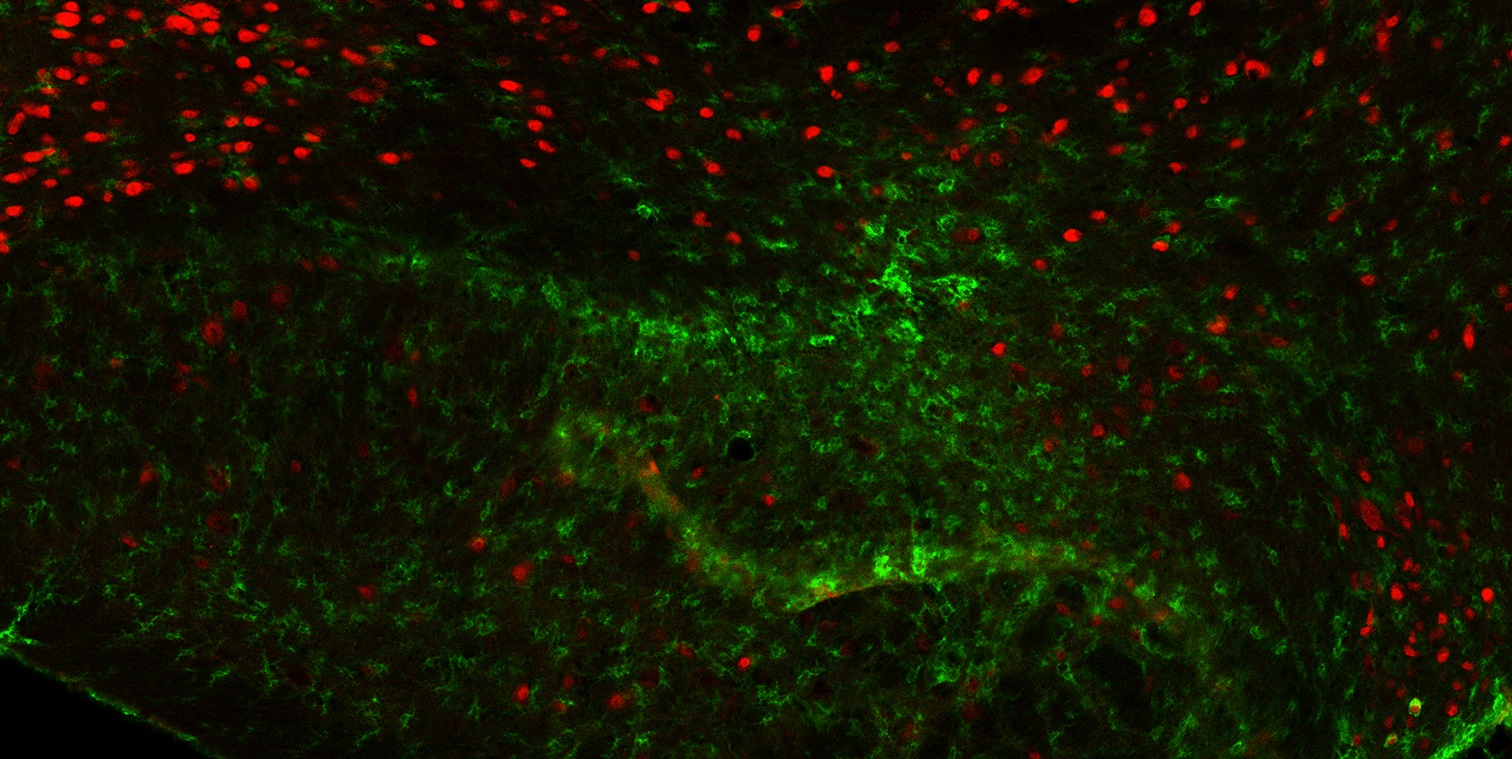

Supplement: Figure 4—source data 2. [file elife-75636-fig4-data2.zip › Fig4 source data 2 for Fig4 D/ASO CTRL #54 HA+NeuN.jpg]

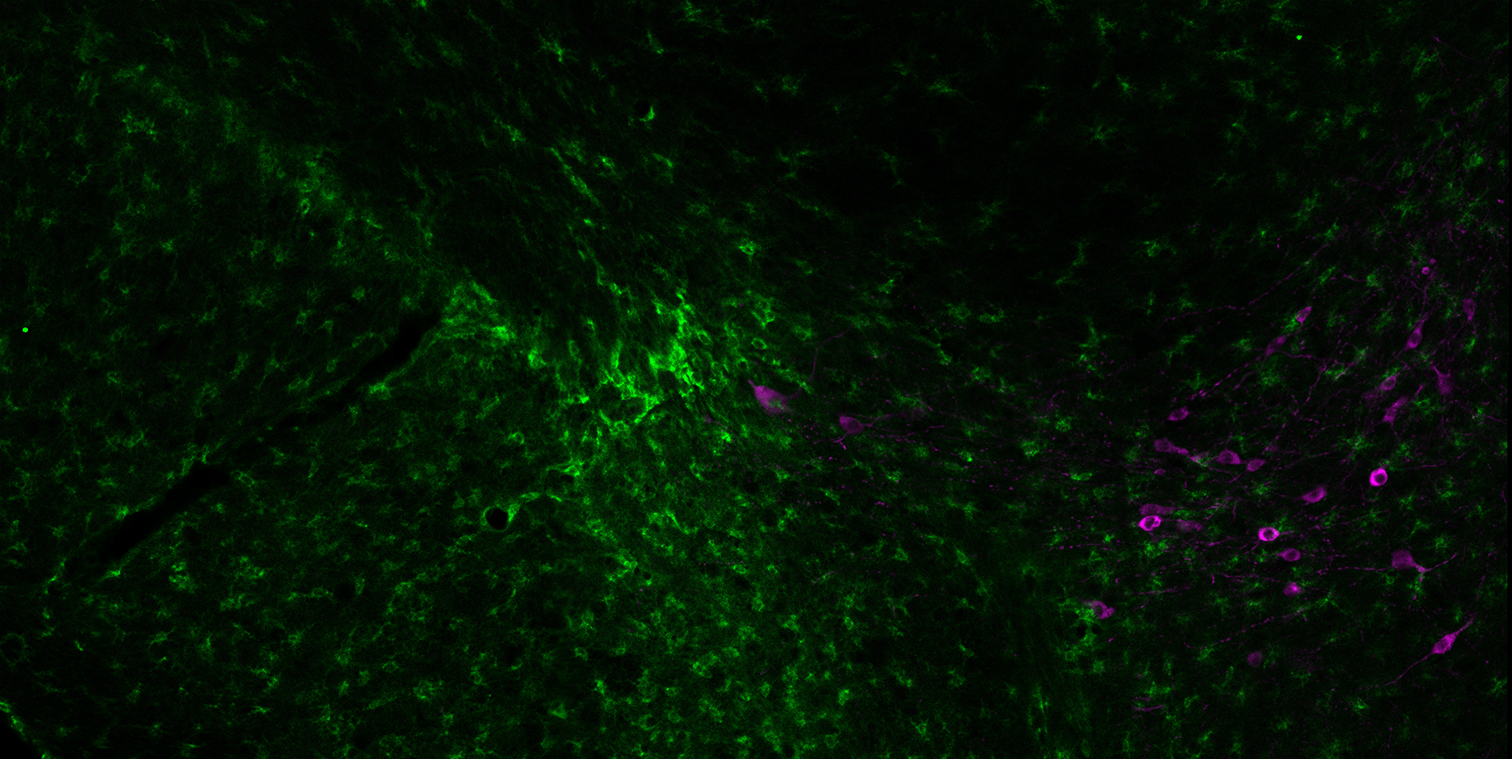

Supplement: Figure 4—source data 2. [file elife-75636-fig4-data2.zip › Fig4 source data 2 for Fig4 D/ASO CTRL #54 HA+TH.jpg]

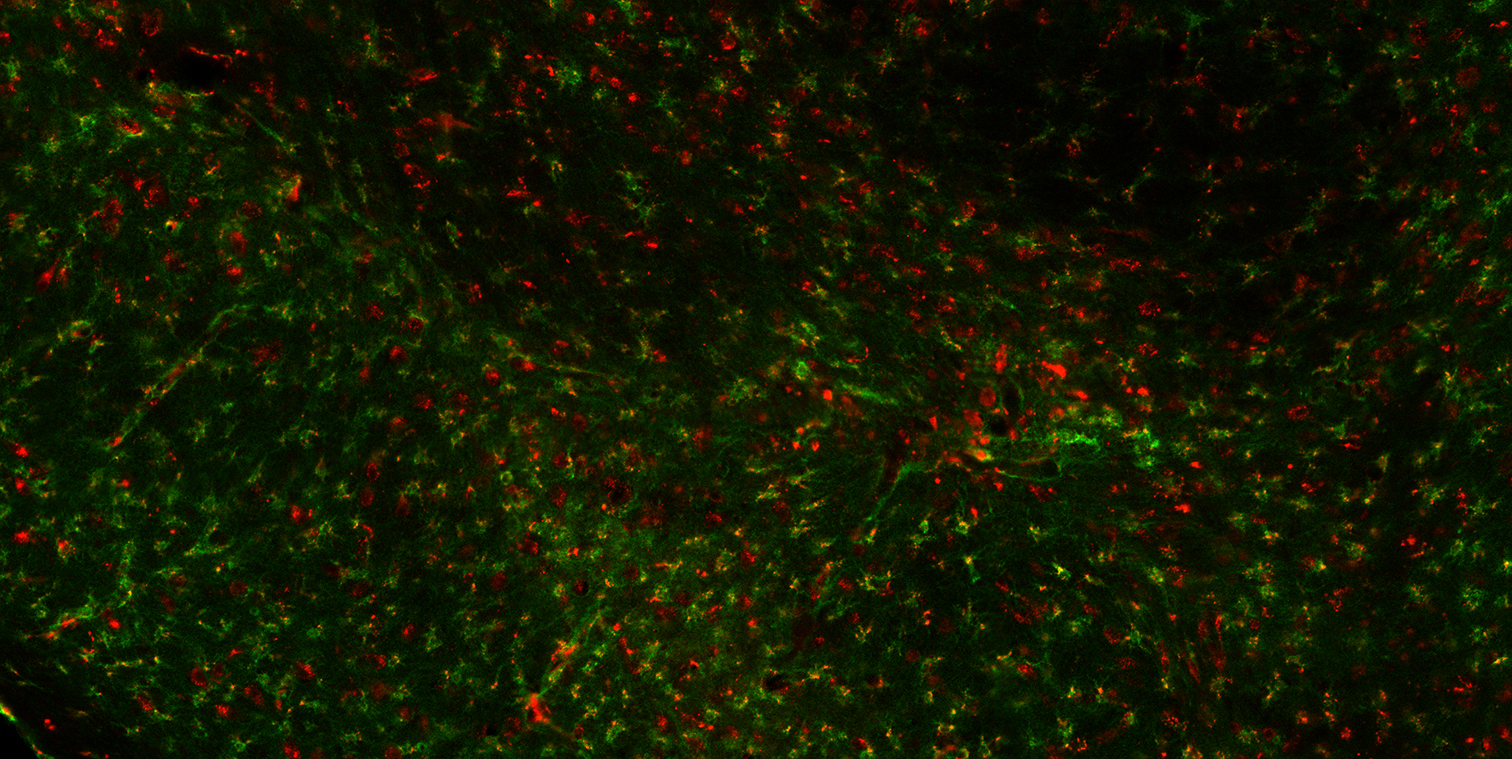

Supplement: Figure 4—source data 2. [file elife-75636-fig4-data2.zip › Fig4 source data 2 for Fig4 D/ASO CTRL #56 HA+CY3-1.jpg]

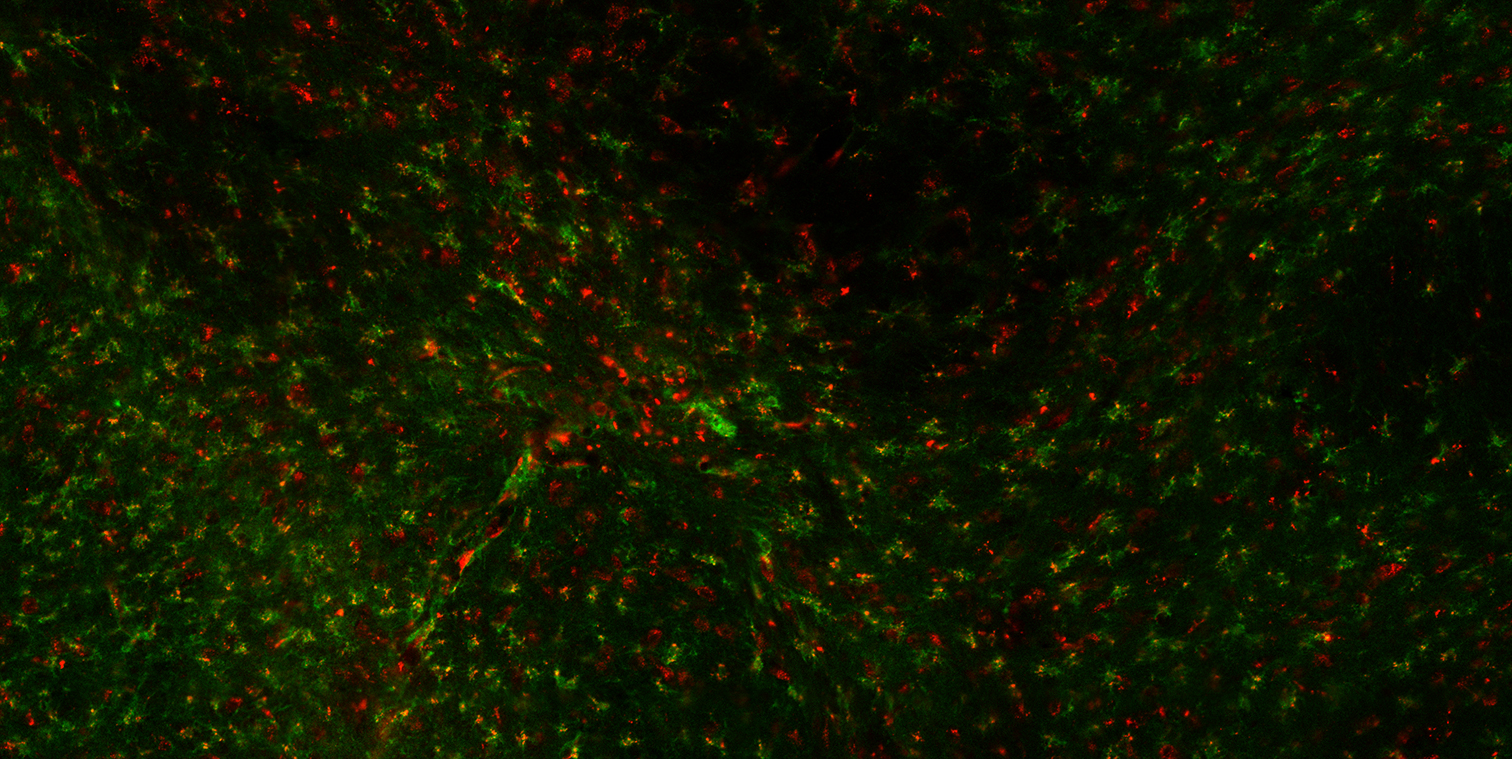

Supplement: Figure 4—source data 2. [file elife-75636-fig4-data2.zip › Fig4 source data 2 for Fig4 D/ASO CTRL #56 HA+CY3-2.jpg]

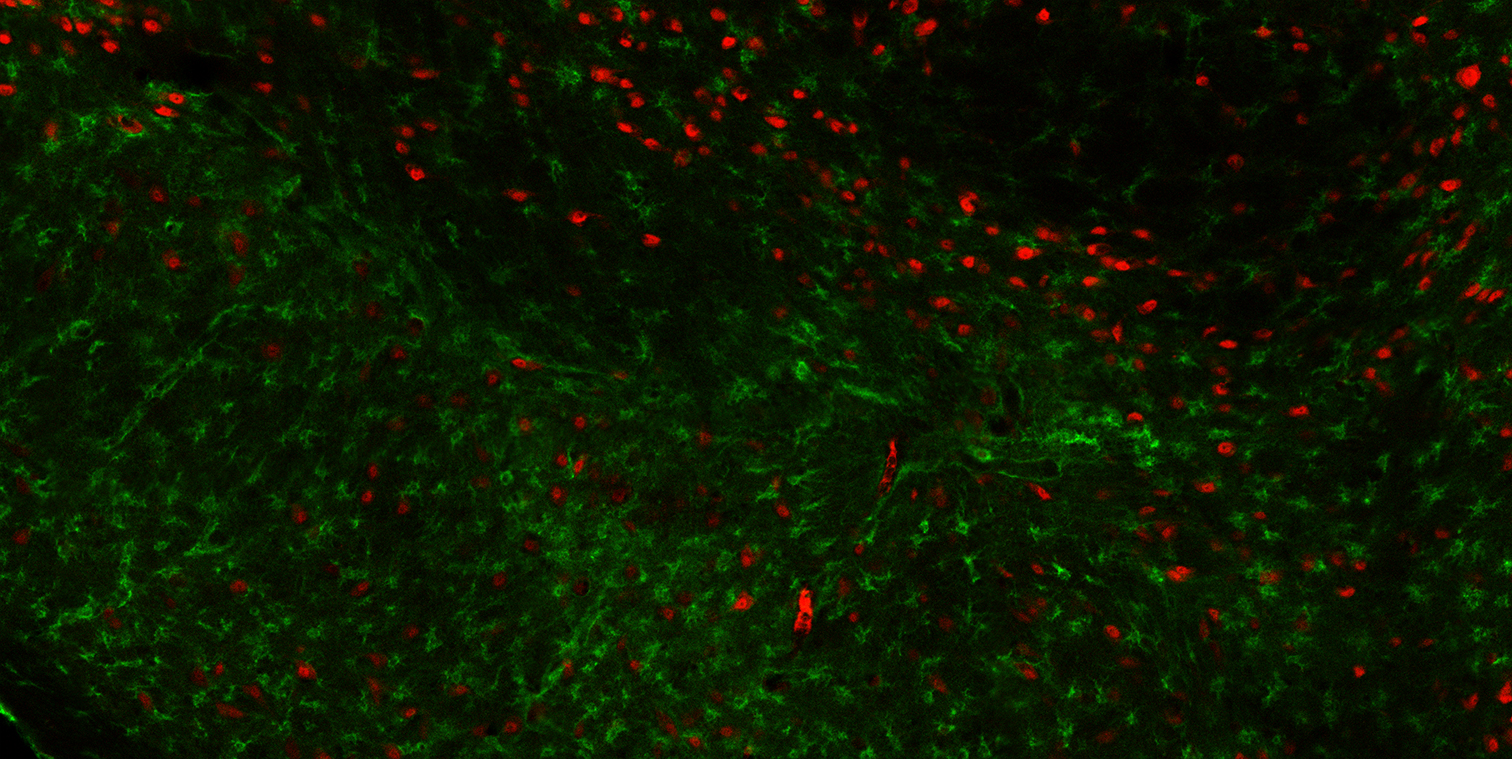

Supplement: Figure 4—source data 2. [file elife-75636-fig4-data2.zip › Fig4 source data 2 for Fig4 D/ASO CTRL #56 HA+NeuN.jpg]

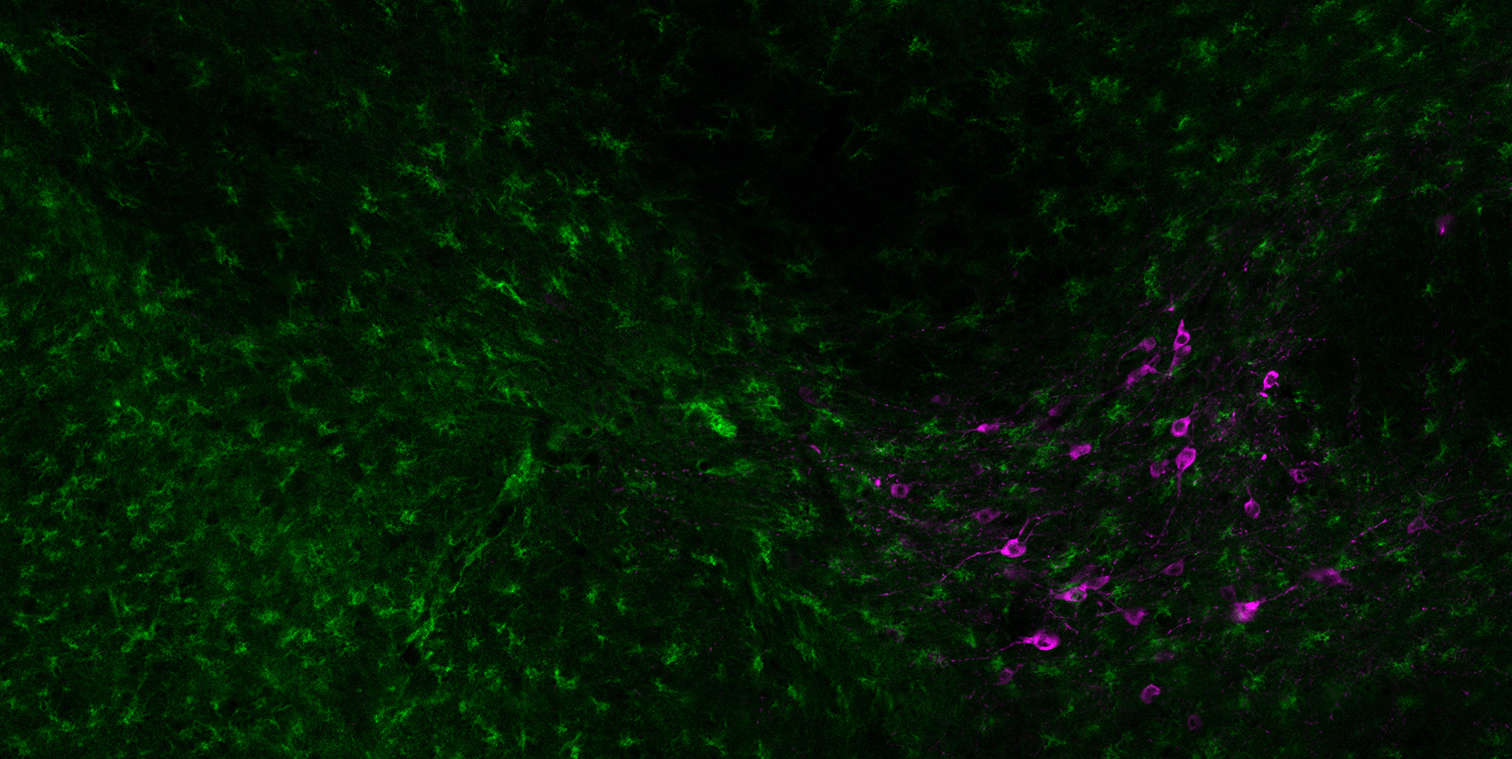

Supplement: Figure 4—source data 2. [file elife-75636-fig4-data2.zip › Fig4 source data 2 for Fig4 D/ASO CTRL #56 HA+TH.jpg]

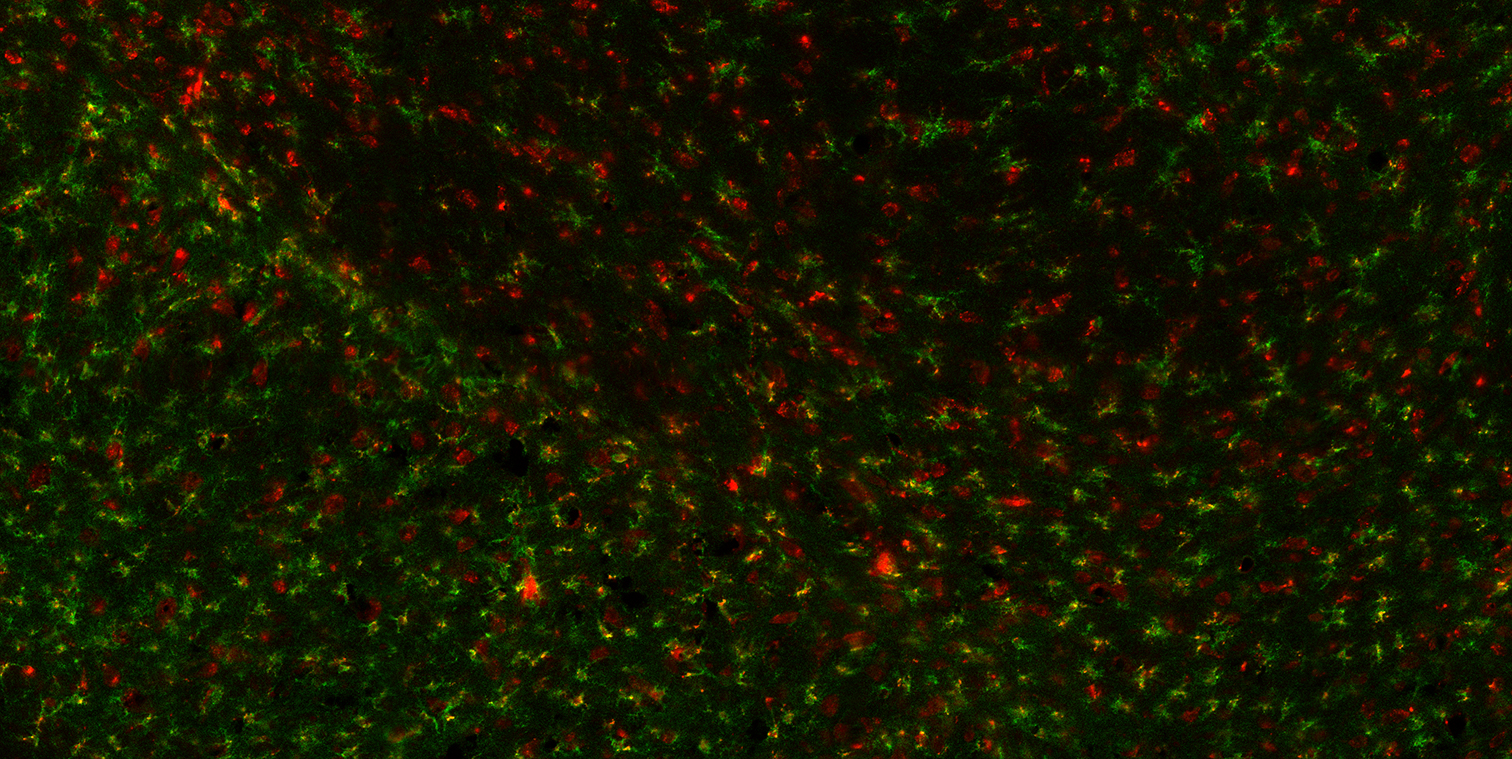

Supplement: Figure 4—source data 2. [file elife-75636-fig4-data2.zip › Fig4 source data 2 for Fig4 D/ASO PTB #85 HA+CY3-1.jpg]

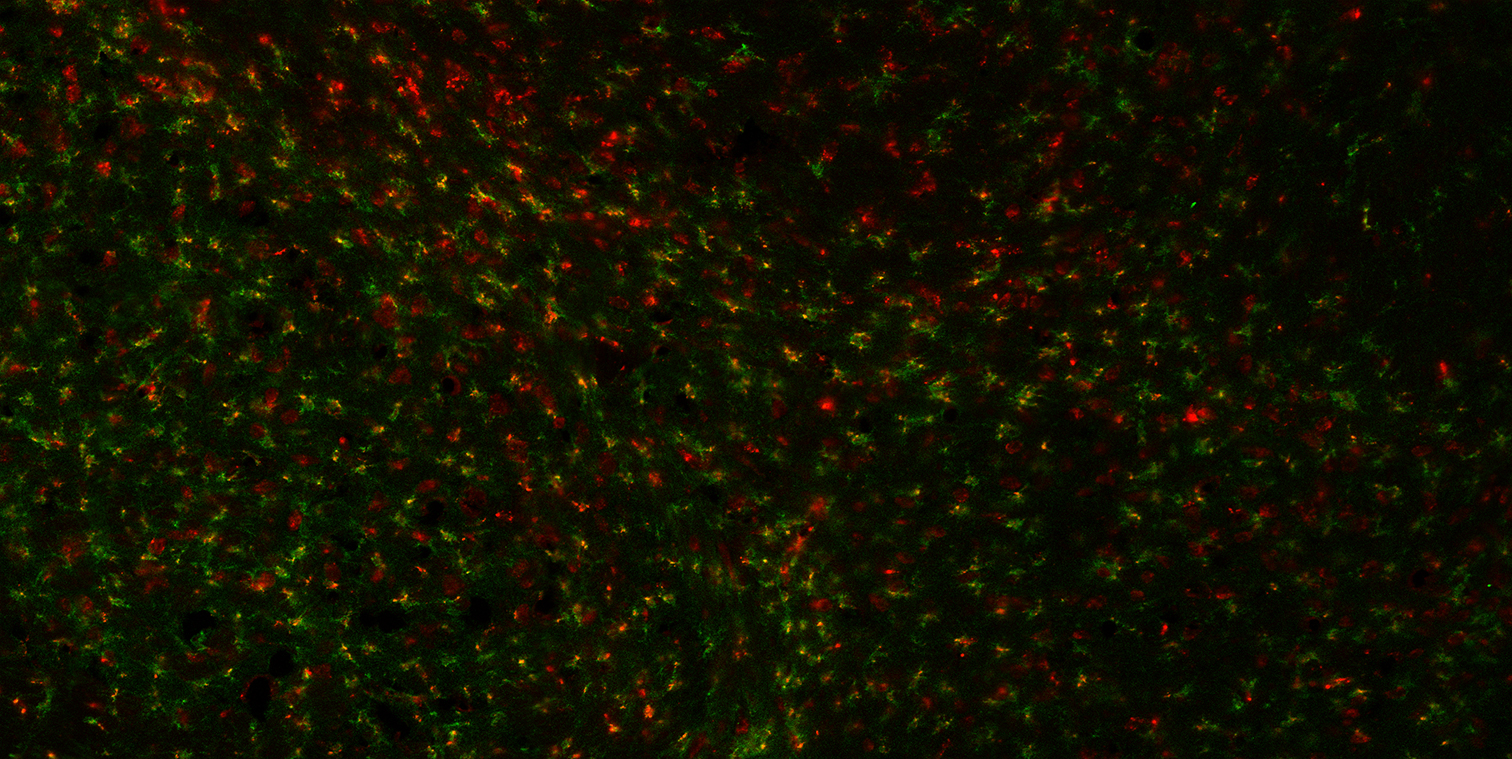

Supplement: Figure 4—source data 2. [file elife-75636-fig4-data2.zip › Fig4 source data 2 for Fig4 D/ASO PTB #85 HA+CY3-2.jpg]

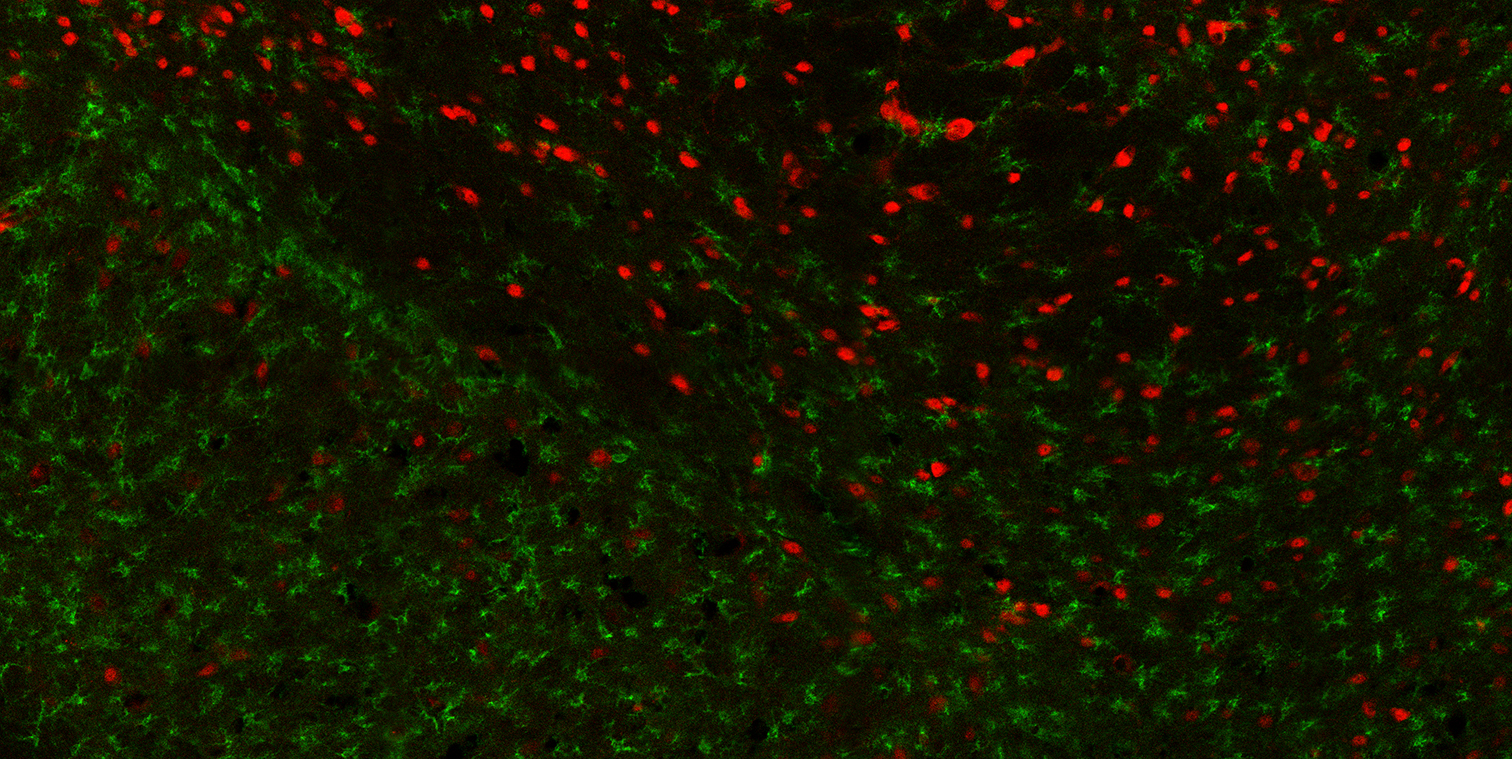

Supplement: Figure 4—source data 2. [file elife-75636-fig4-data2.zip › Fig4 source data 2 for Fig4 D/ASO PTB #85 HA+NeuN.jpg]

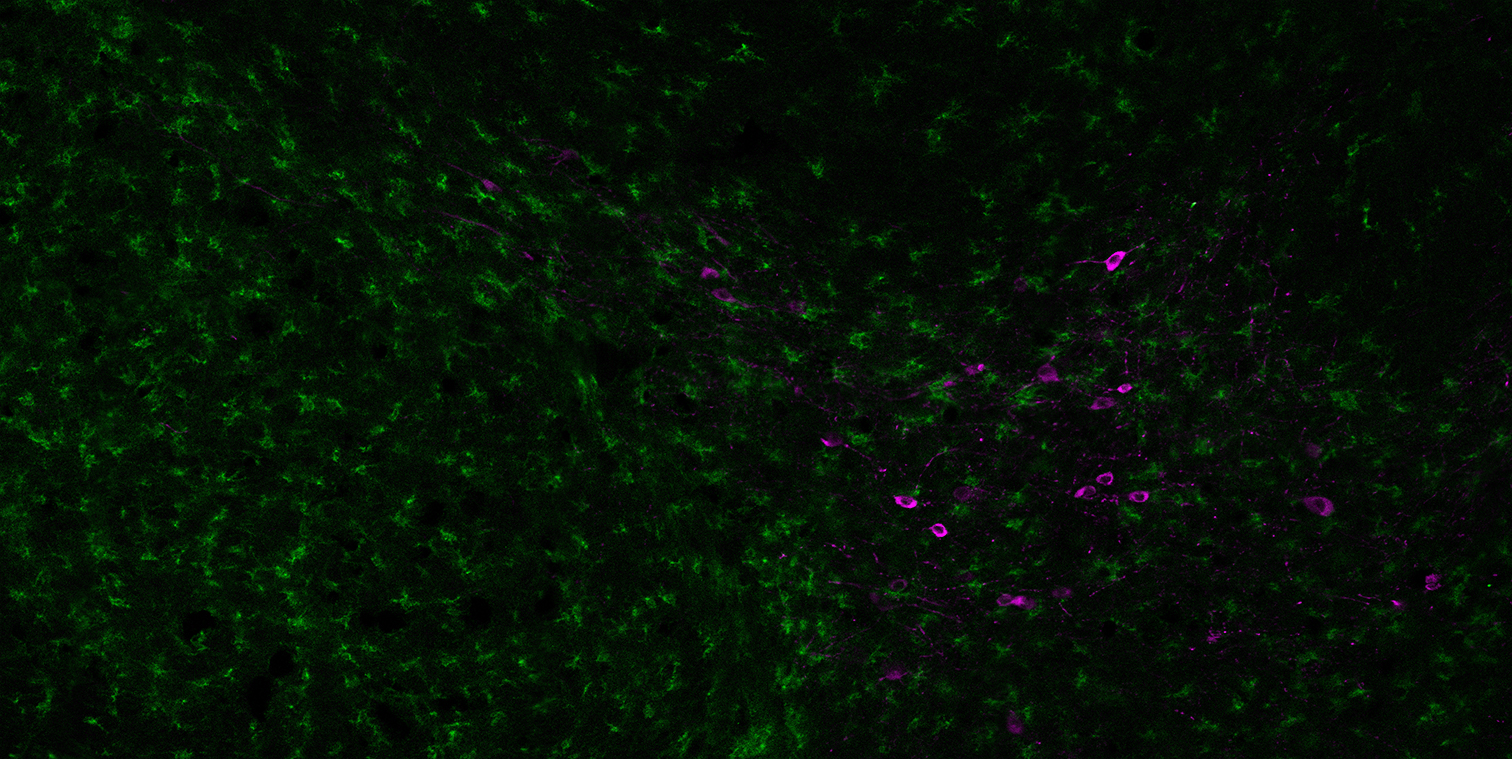

Supplement: Figure 4—source data 2. [file elife-75636-fig4-data2.zip › Fig4 source data 2 for Fig4 D/ASO PTB #85 HA+TH.jpg]

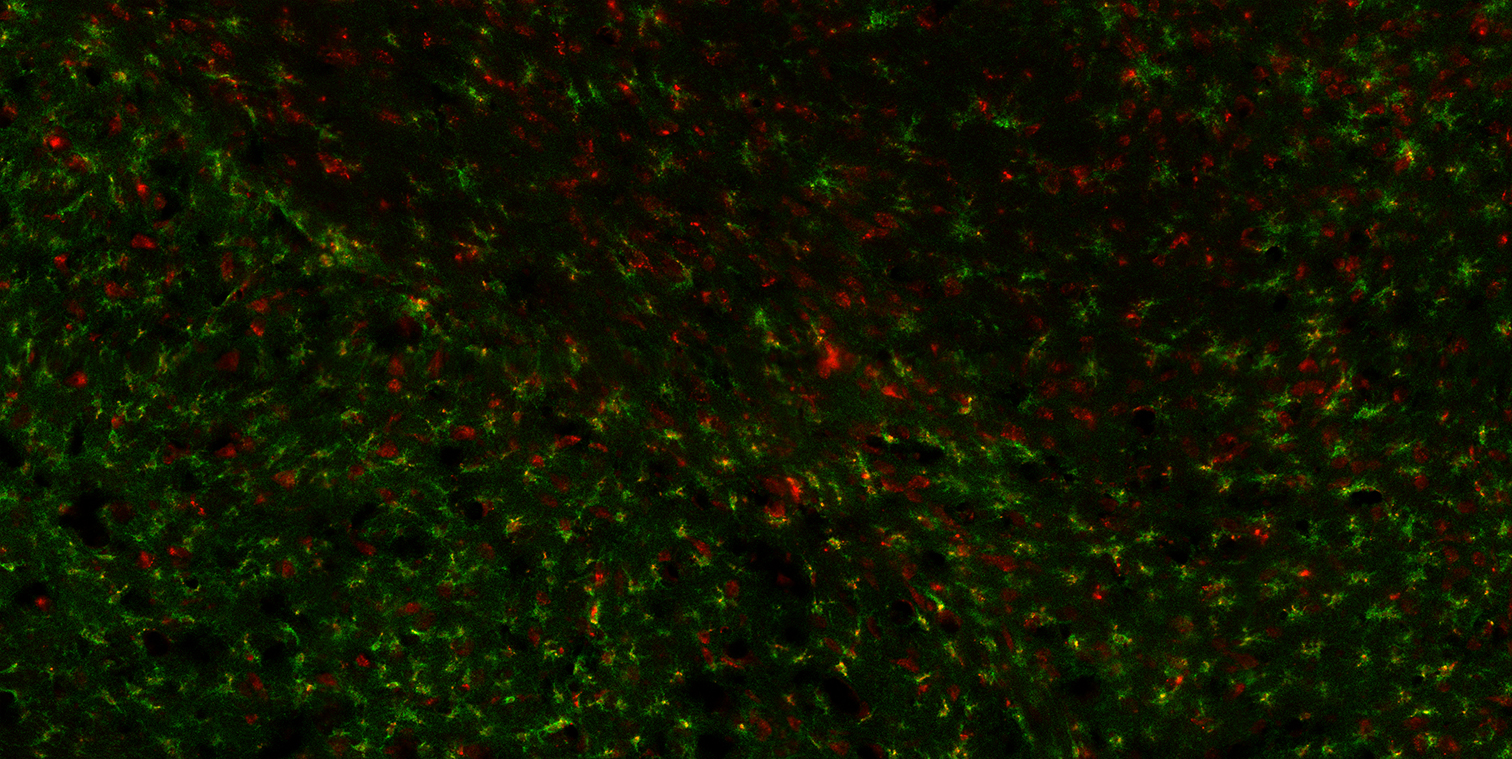

Supplement: Figure 4—source data 2. [file elife-75636-fig4-data2.zip › Fig4 source data 2 for Fig4 D/ASO PTB #90 HA+CY3-1.jpg]

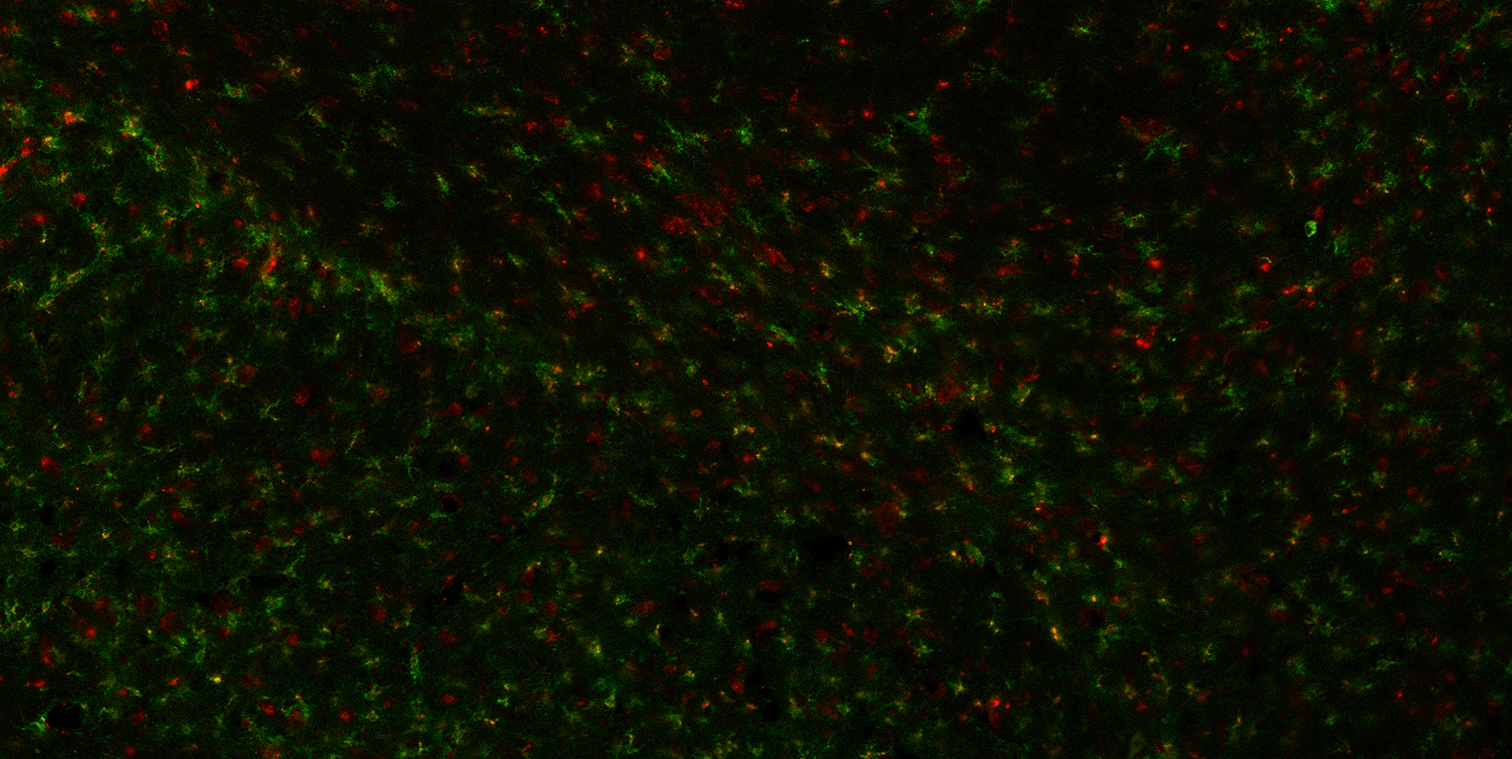

Supplement: Figure 4—source data 2. [file elife-75636-fig4-data2.zip › Fig4 source data 2 for Fig4 D/ASO PTB #90 HA+CY3-2.jpg]

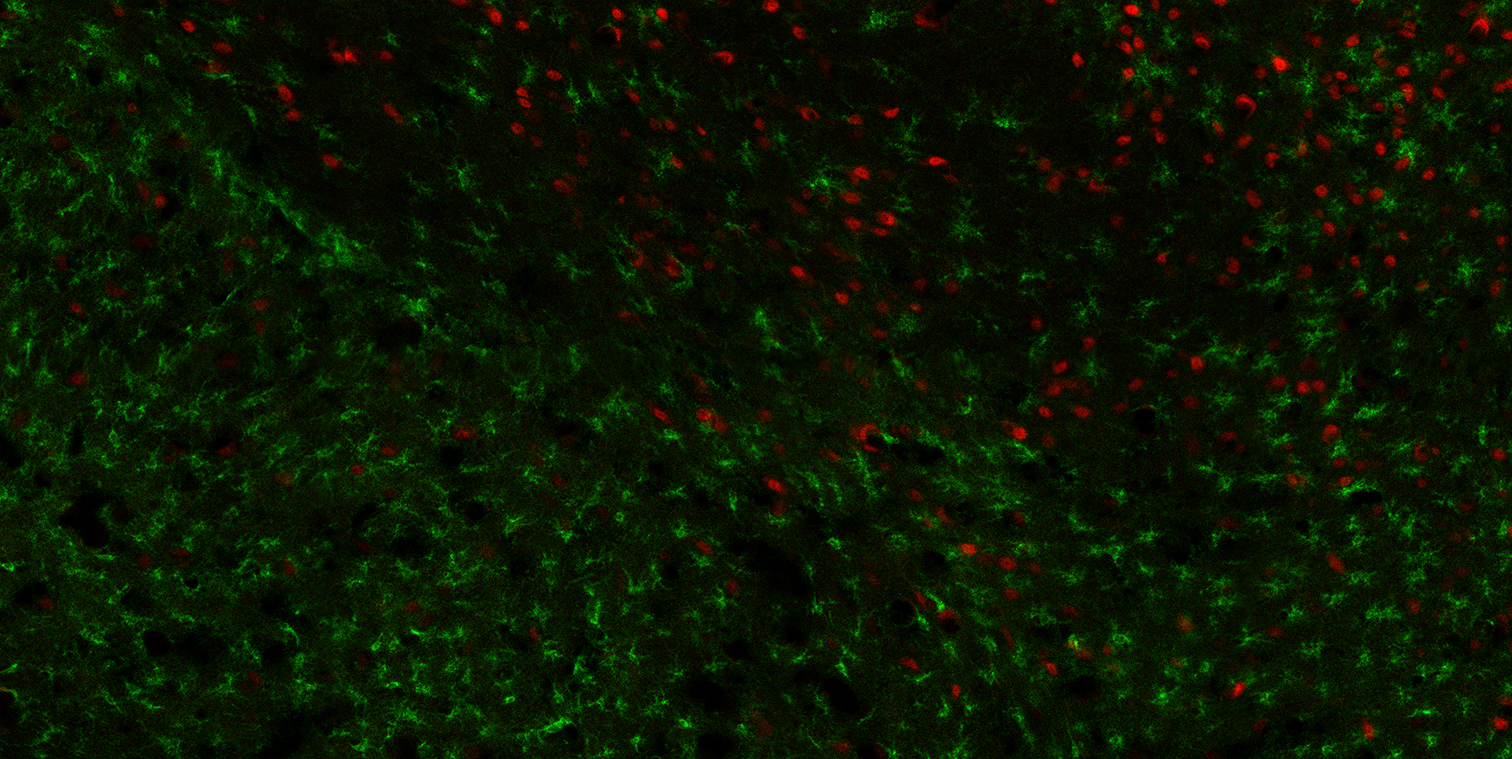

Supplement: Figure 4—source data 2. [file elife-75636-fig4-data2.zip › Fig4 source data 2 for Fig4 D/ASO PTB #90 HA+NeuN.jpg]

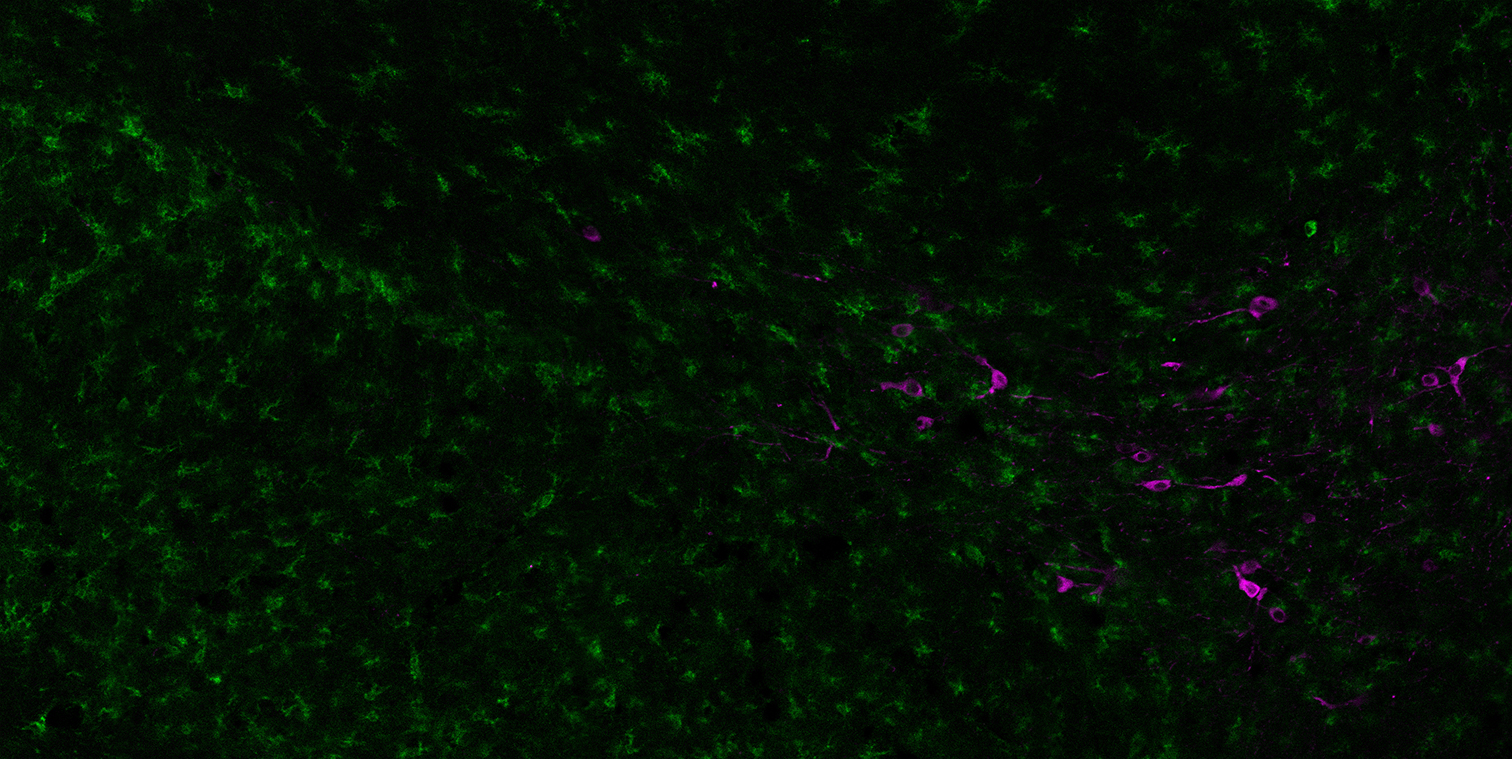

Supplement: Figure 4—source data 2. [file elife-75636-fig4-data2.zip › Fig4 source data 2 for Fig4 D/ASO PTB #90 HA+TH.jpg]

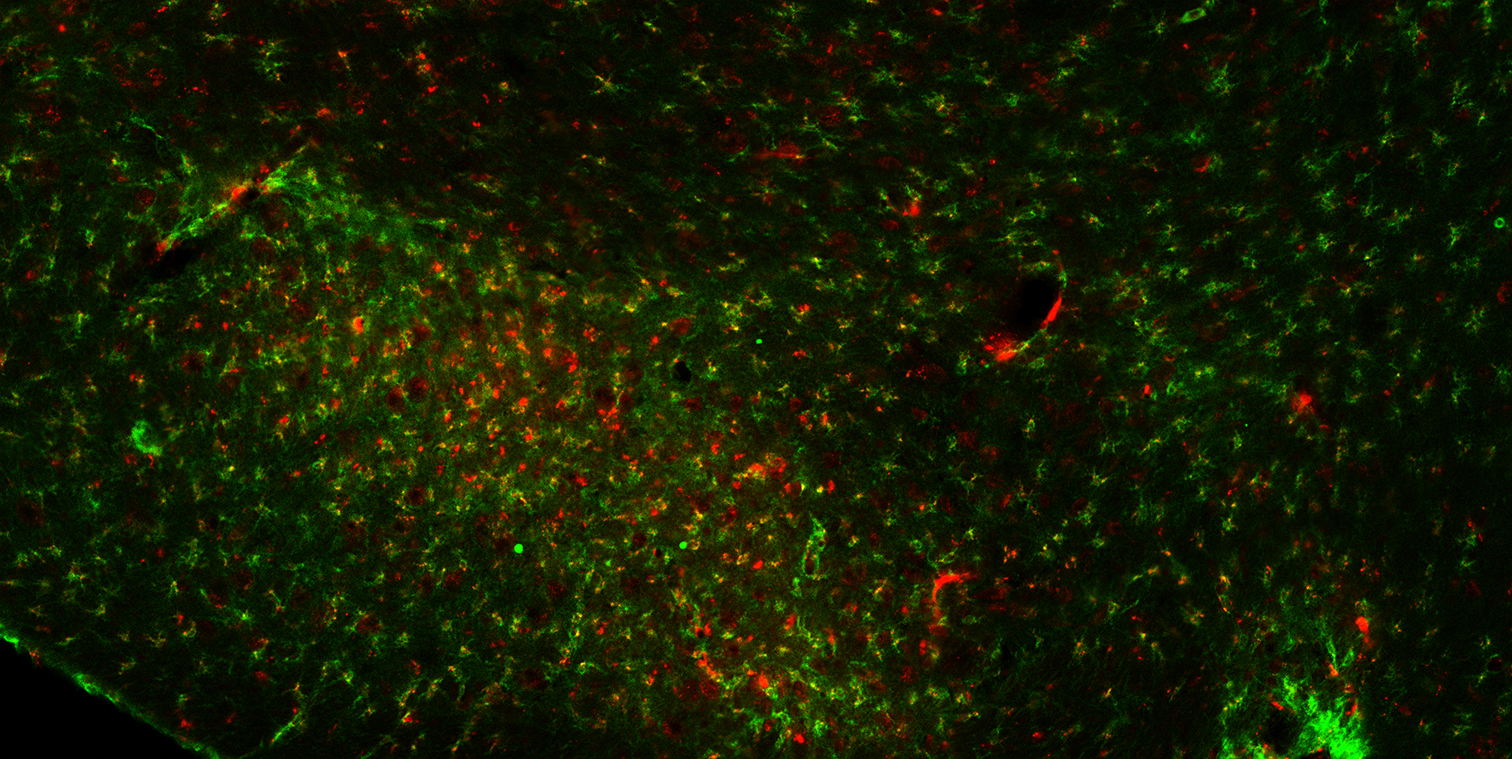

Supplement: Figure 4—source data 2. [file elife-75636-fig4-data2.zip › Fig4 source data 2 for Fig4 D/ASO PTB #97 HA+CY3-1.jpg]

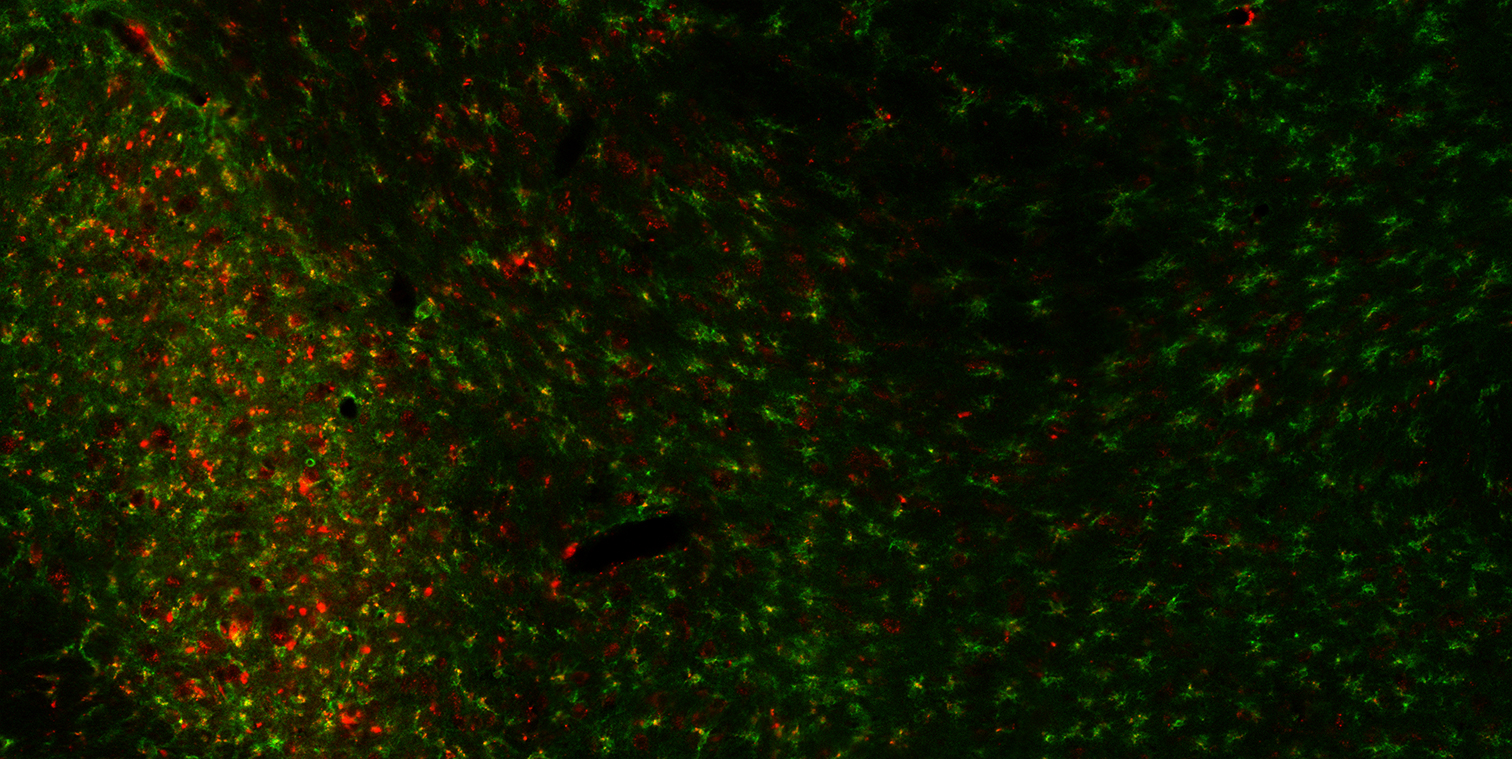

Supplement: Figure 4—source data 2. [file elife-75636-fig4-data2.zip › Fig4 source data 2 for Fig4 D/ASO PTB #97 HA+CY3-2.jpg]

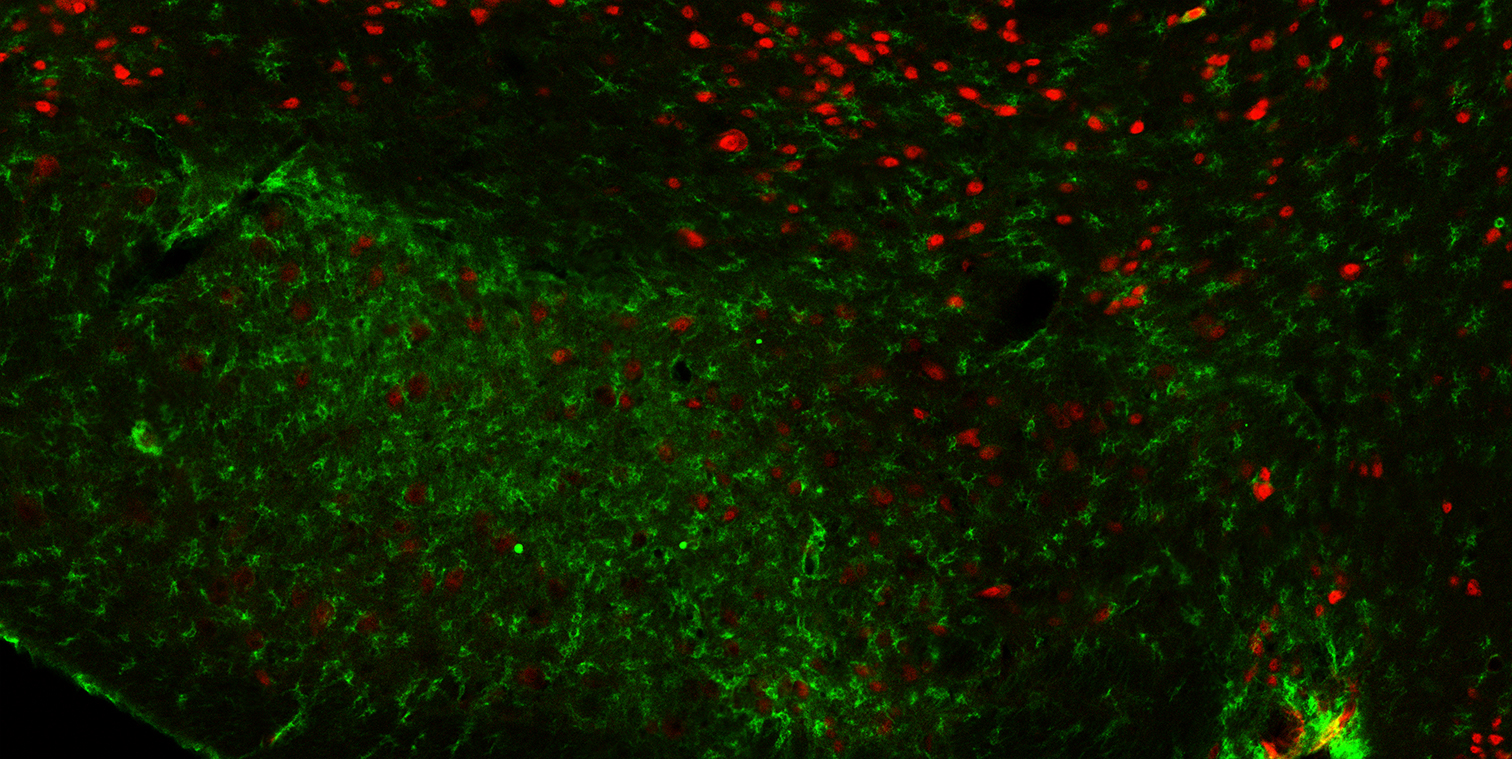

Supplement: Figure 4—source data 2. [file elife-75636-fig4-data2.zip › Fig4 source data 2 for Fig4 D/ASO PTB #97 HA+NeuN.jpg]

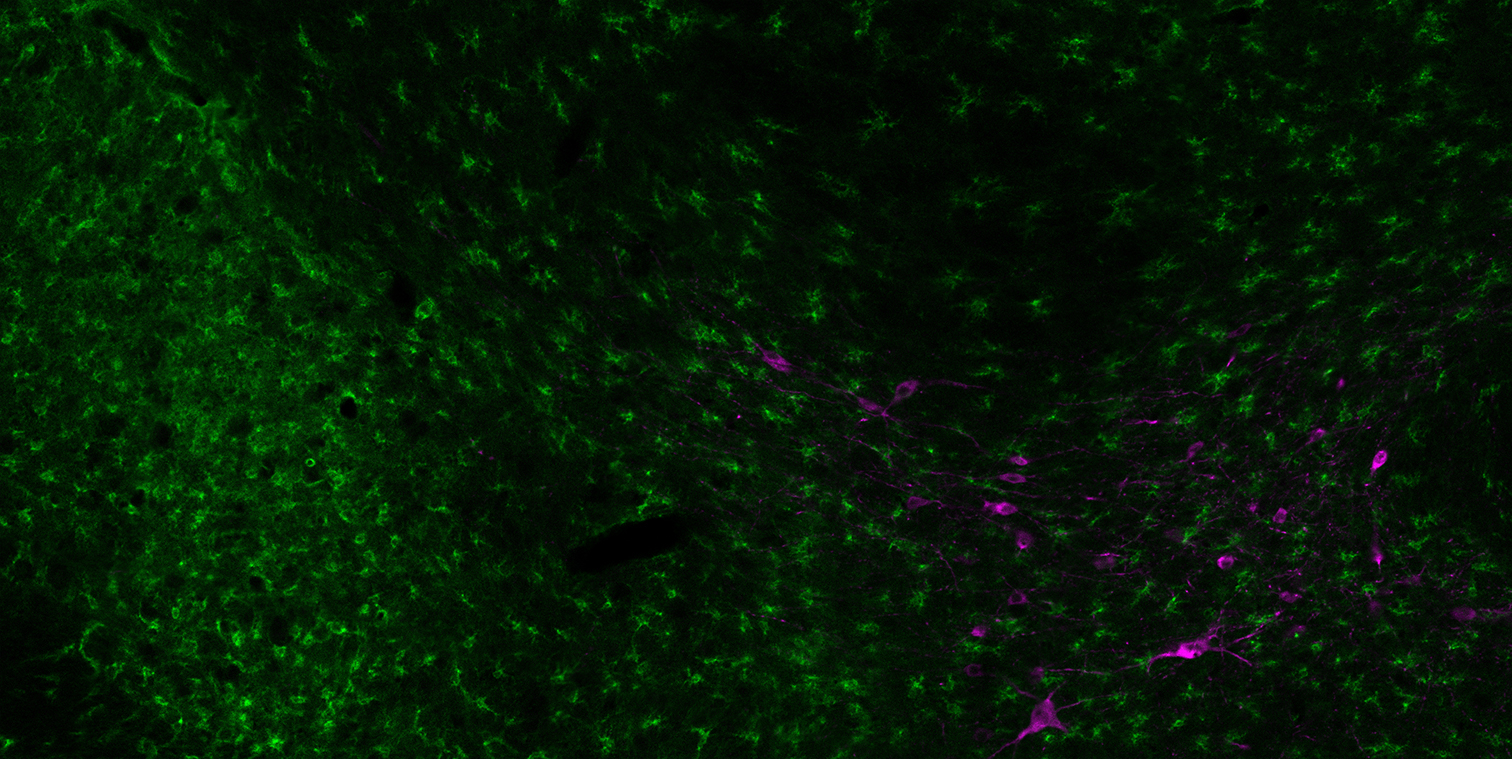

Supplement: Figure 4—source data 2. [file elife-75636-fig4-data2.zip › Fig4 source data 2 for Fig4 D/ASO PTB #97 HA+TH.jpg]

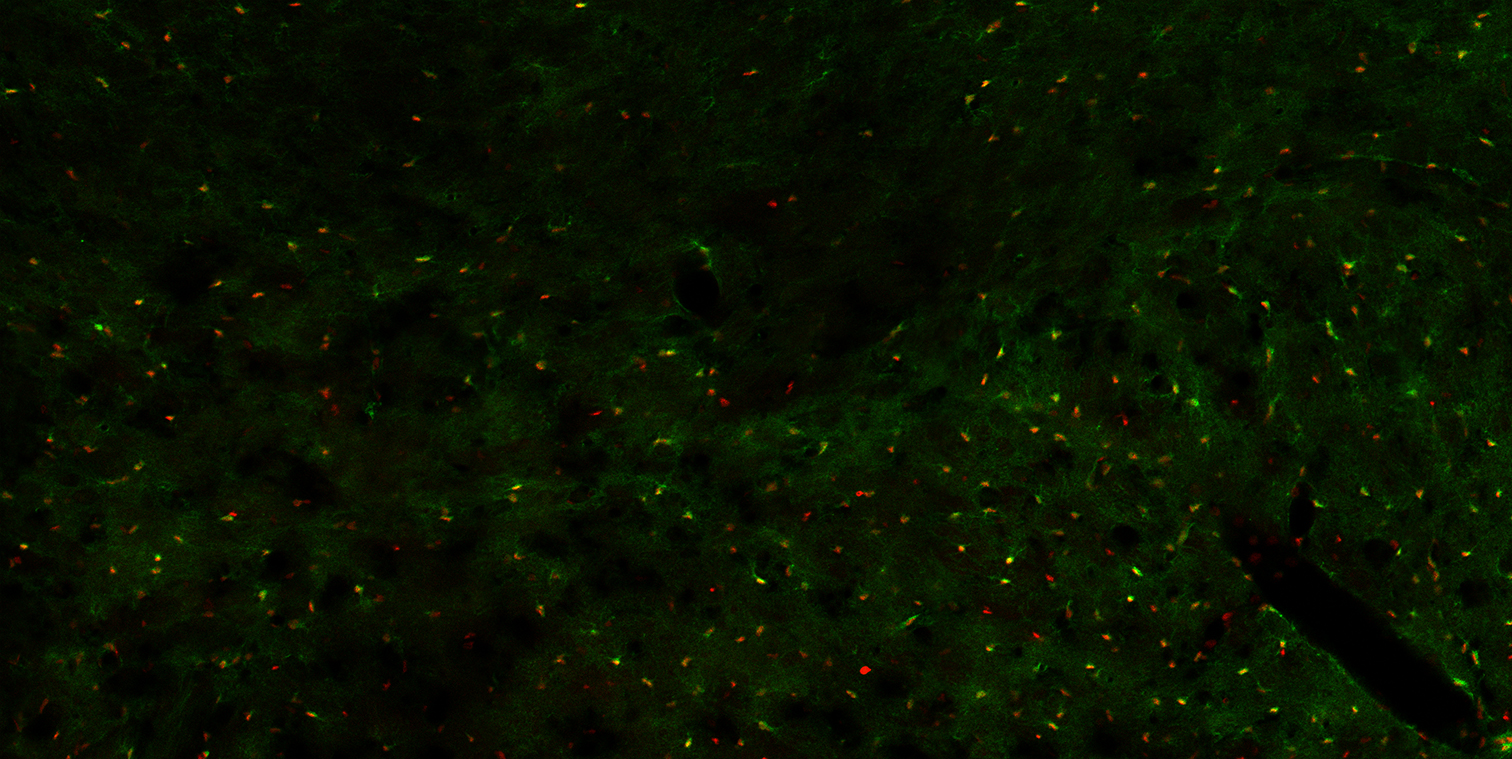

Supplement: Figure 4—figure supplement 1—source data 1. [file elife-75636-fig4-figsupp1-data1.zip › Fig4 source data 4 for Fig4 supplement 1/#23 ASO-ctrl YFP+PTBP1.jpg]

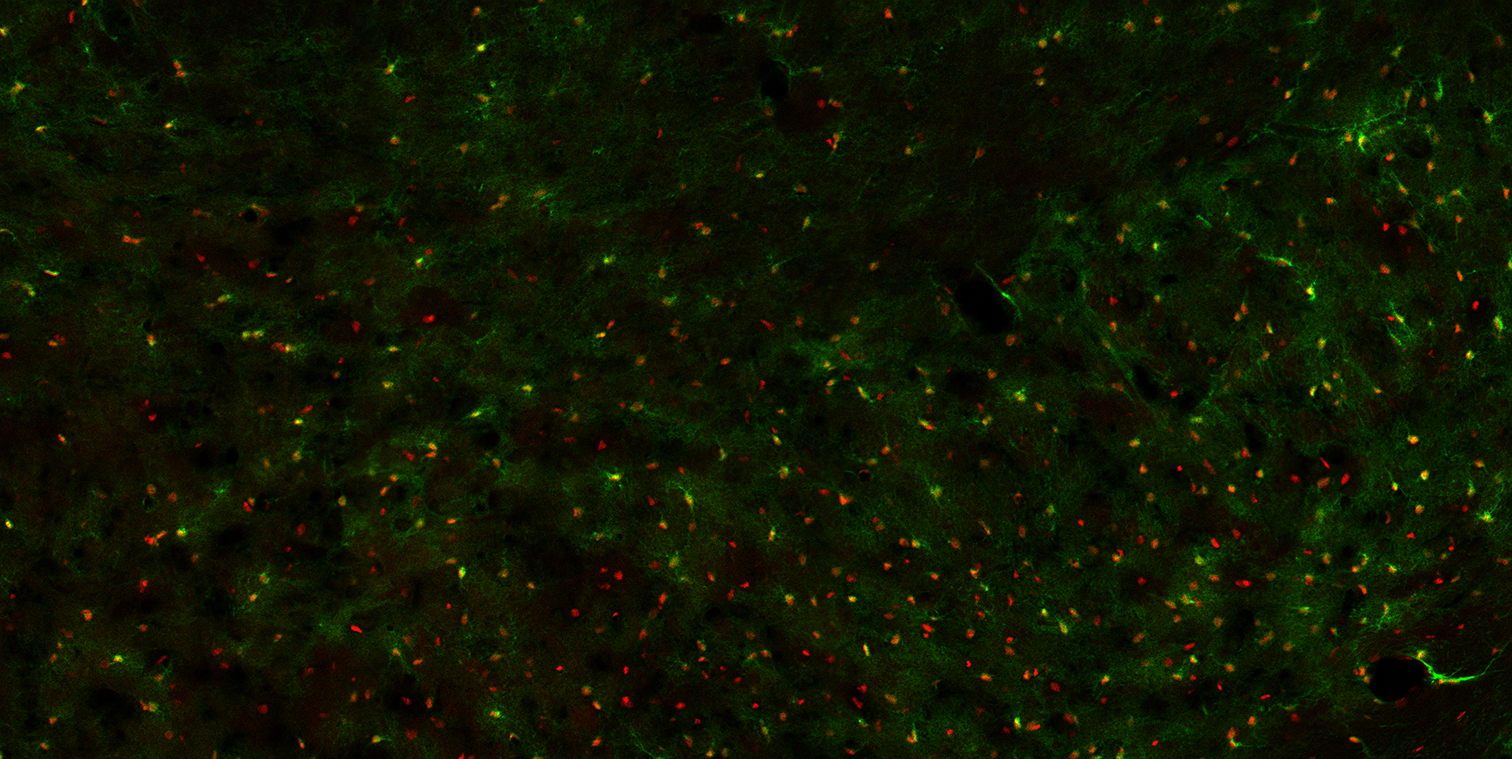

Supplement: Figure 4—figure supplement 1—source data 1. [file elife-75636-fig4-figsupp1-data1.zip › Fig4 source data 4 for Fig4 supplement 1/#24 ASO-ctrl YFP+PTBP1.jpg]

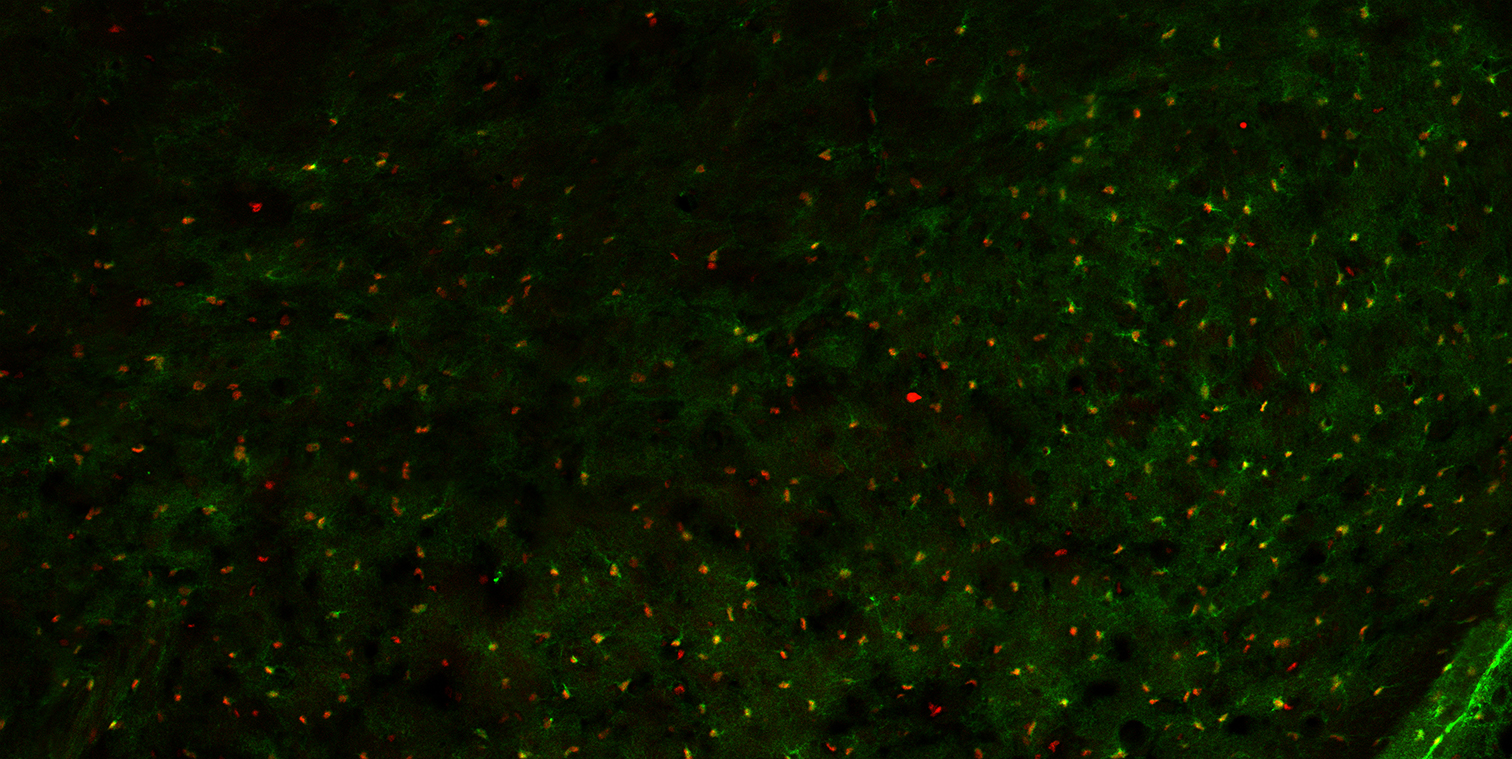

Supplement: Figure 4—figure supplement 1—source data 1. [file elife-75636-fig4-figsupp1-data1.zip › Fig4 source data 4 for Fig4 supplement 1/#66 ASO-ctrl YFP+PTBP1.jpg]

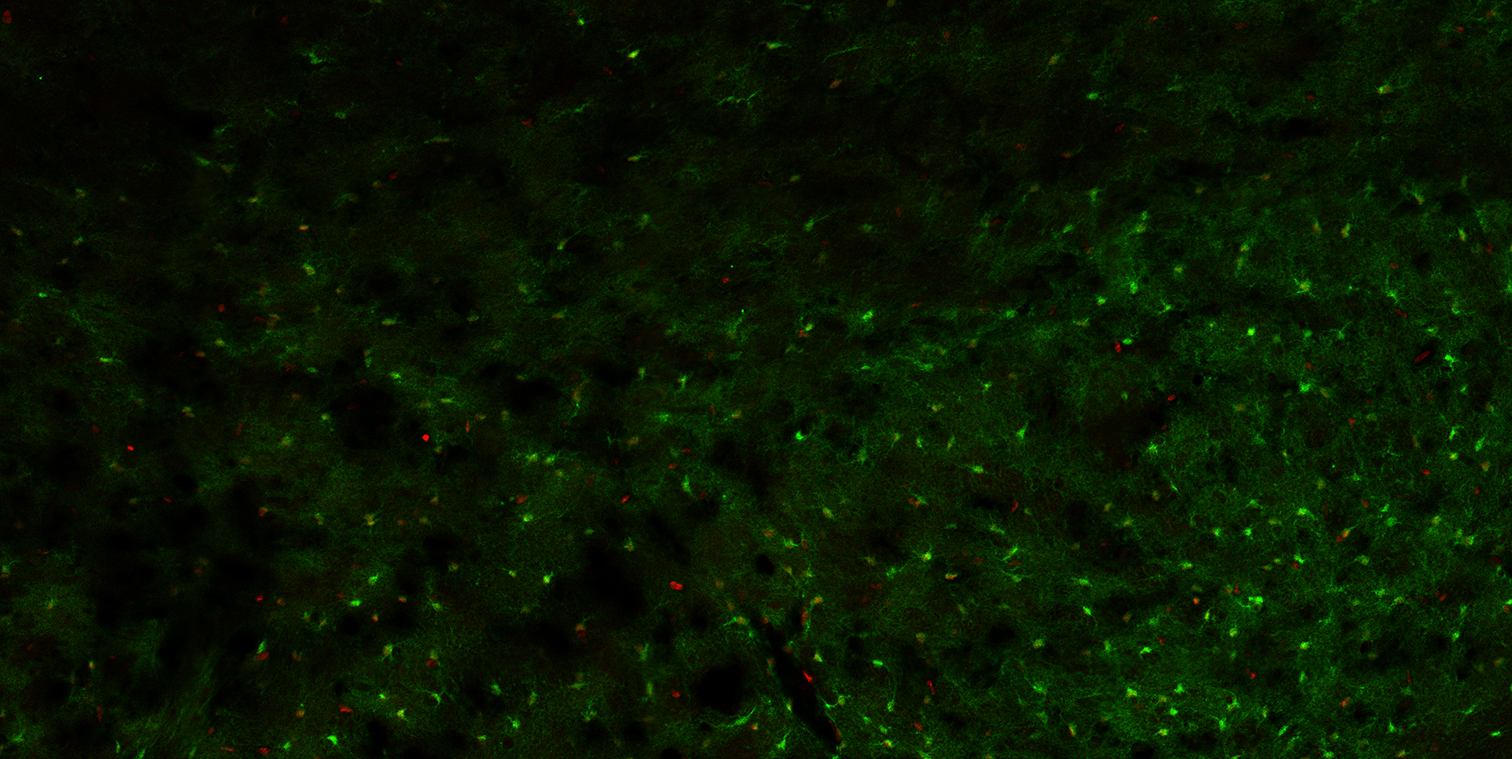

Supplement: Figure 4—figure supplement 1—source data 1. [file elife-75636-fig4-figsupp1-data1.zip › Fig4 source data 4 for Fig4 supplement 1/#68 ASO-PTB YFP+PTBP1.jpg]

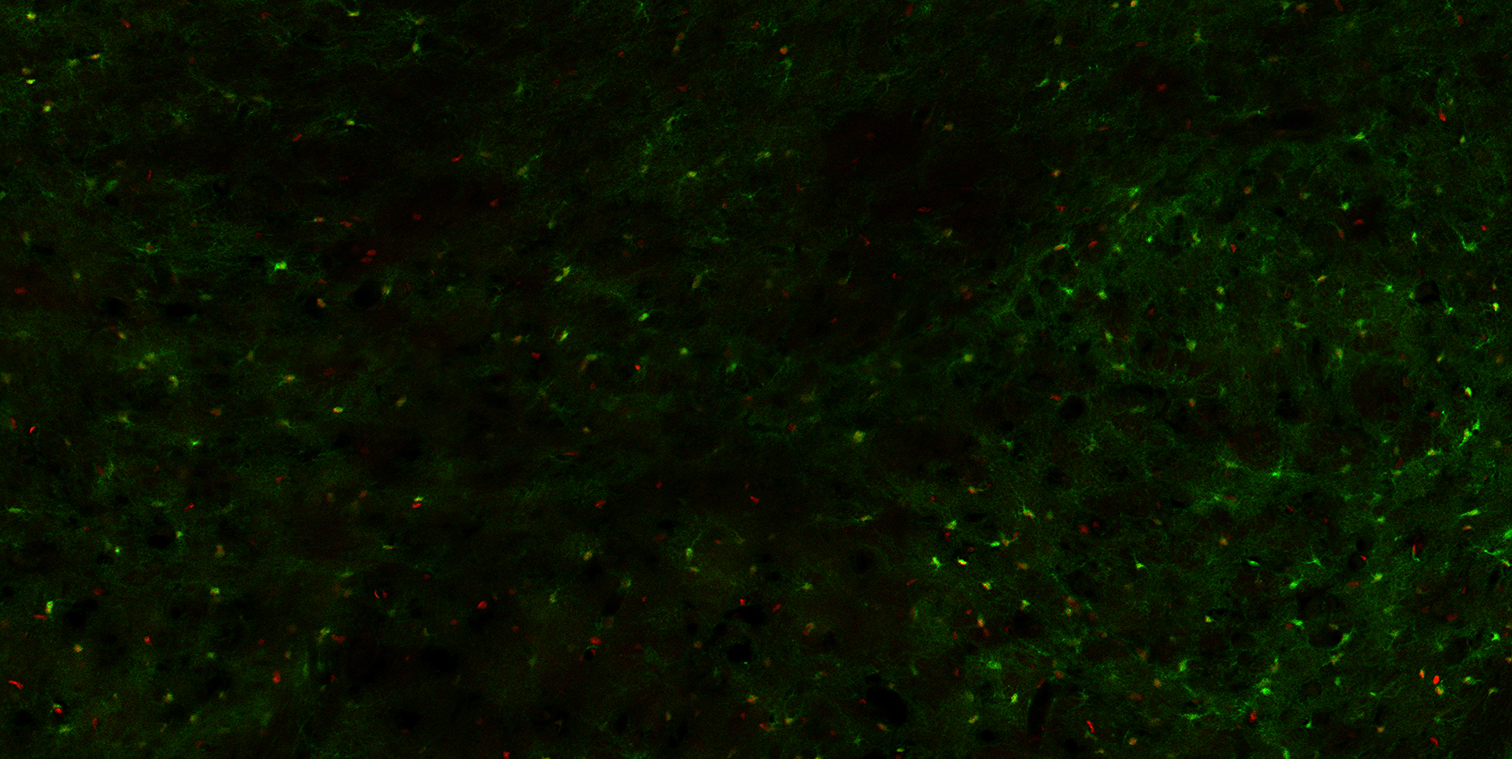

Supplement: Figure 4—figure supplement 1—source data 1. [file elife-75636-fig4-figsupp1-data1.zip › Fig4 source data 4 for Fig4 supplement 1/#69 ASO-PTB YFP+PTBP1.jpg]

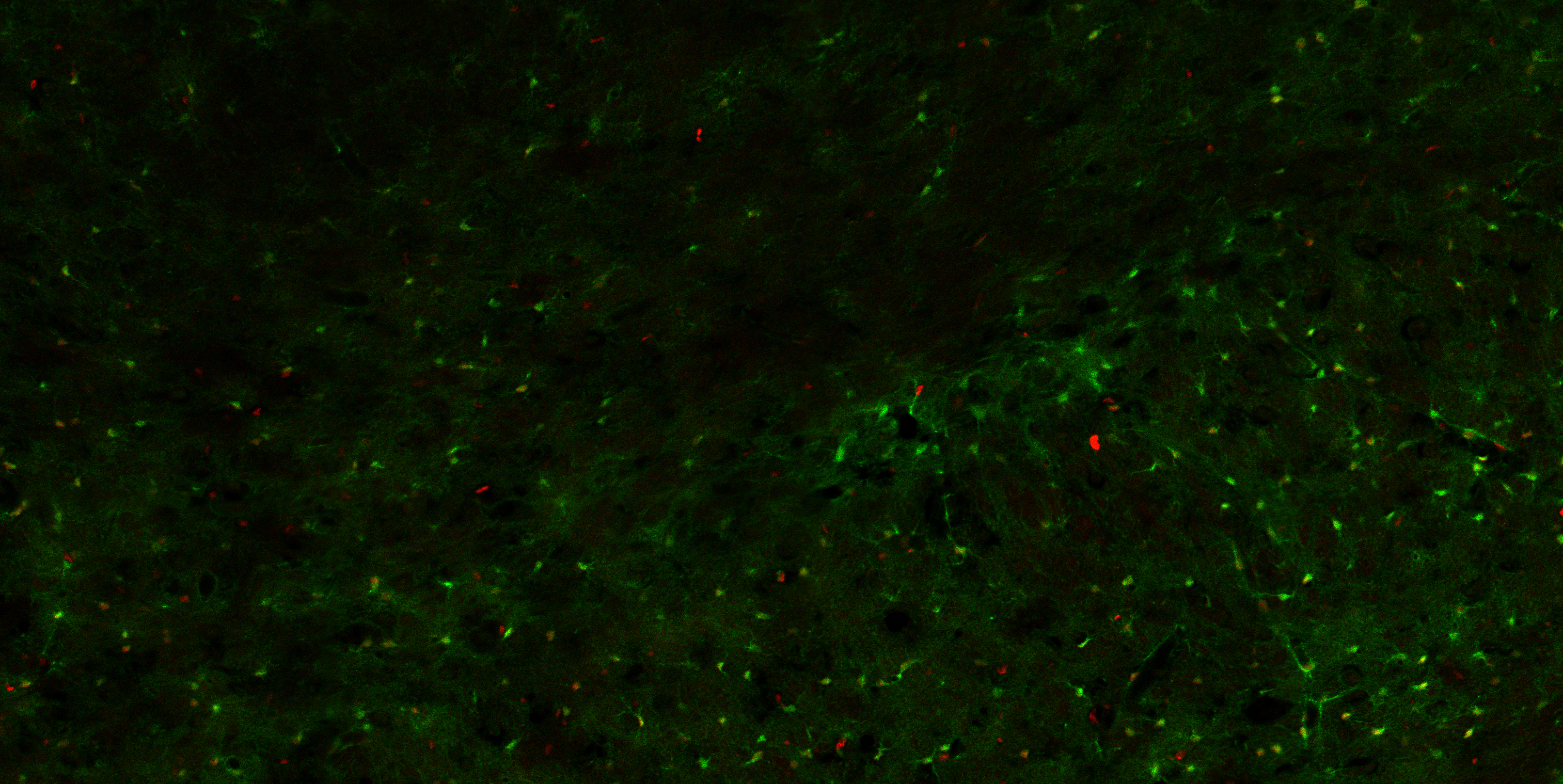

Supplement: Figure 4—figure supplement 1—source data 1. [file elife-75636-fig4-figsupp1-data1.zip › Fig4 source data 4 for Fig4 supplement 1/#70 ASO-PTB YFP+PTBP1.jpg]

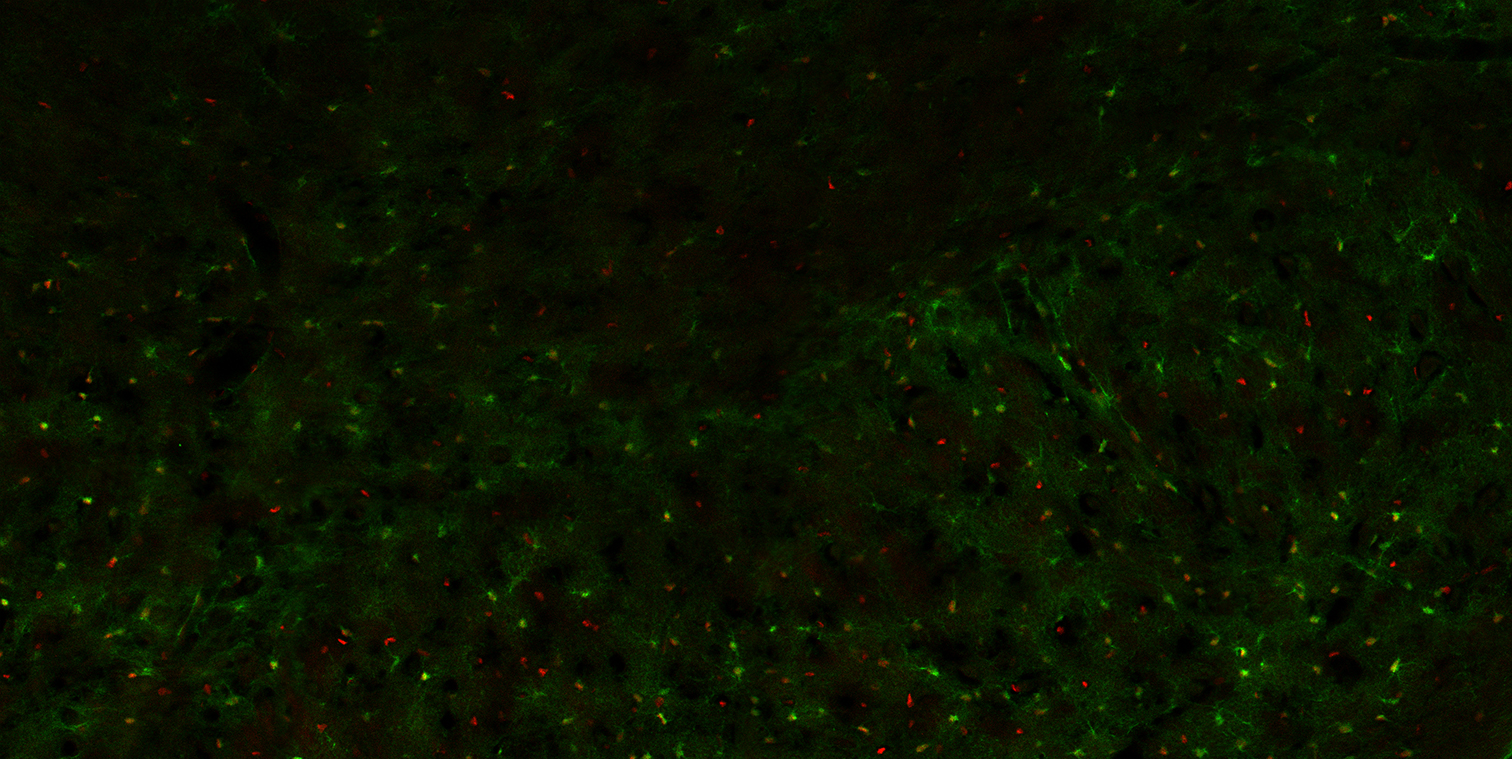

Supplement: Figure 4—figure supplement 1—source data 1. [file elife-75636-fig4-figsupp1-data1.zip › Fig4 source data 4 for Fig4 supplement 1/#71 ASO-PTB YFP+PTBP1.jpg]

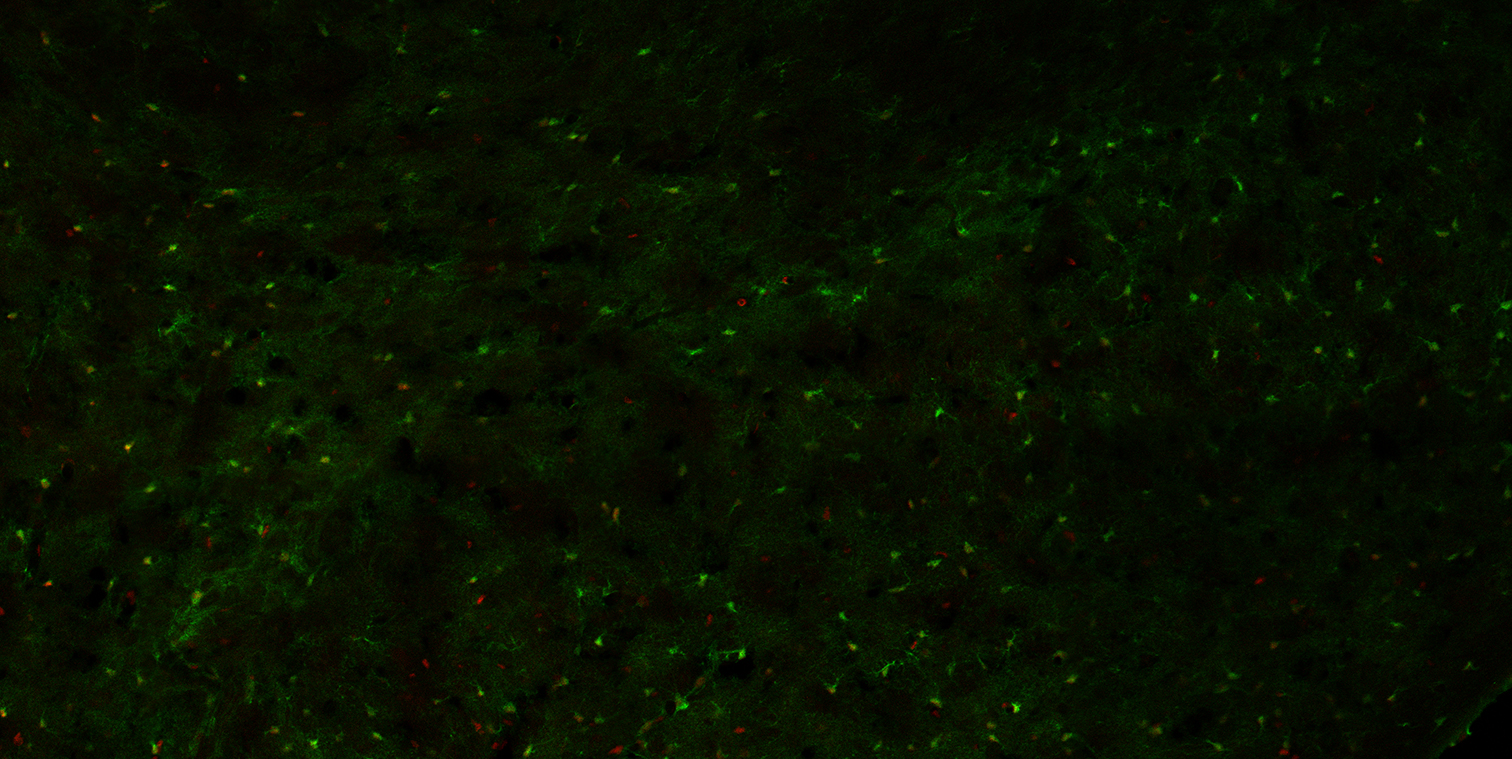

Supplement: Figure 4—figure supplement 1—source data 1. [file elife-75636-fig4-figsupp1-data1.zip › Fig4 source data 4 for Fig4 supplement 1/#72 ASO-PTB YFP+PTBP1.jpg]

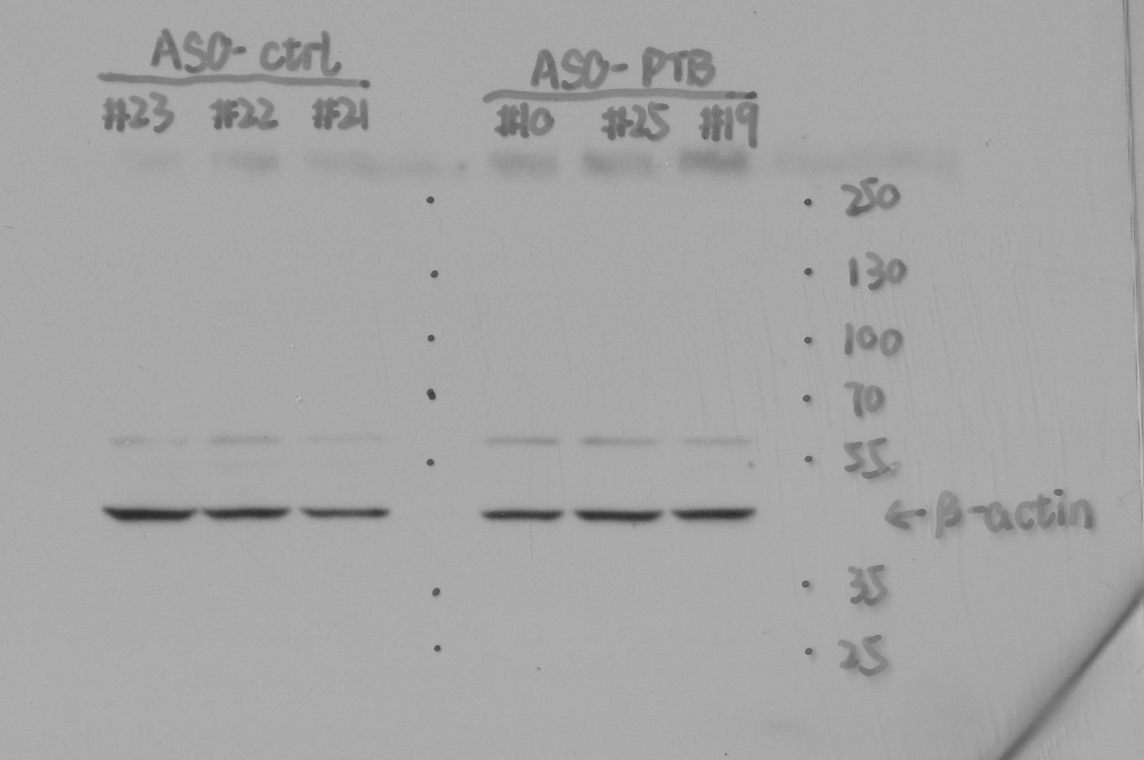

Supplement: Figure 4—figure supplement 1—source data 1. [file elife-75636-fig4-figsupp1-data1.zip › Fig4 source data 4 for Fig4 supplement 1/ASO ACTIN WB.jpg]

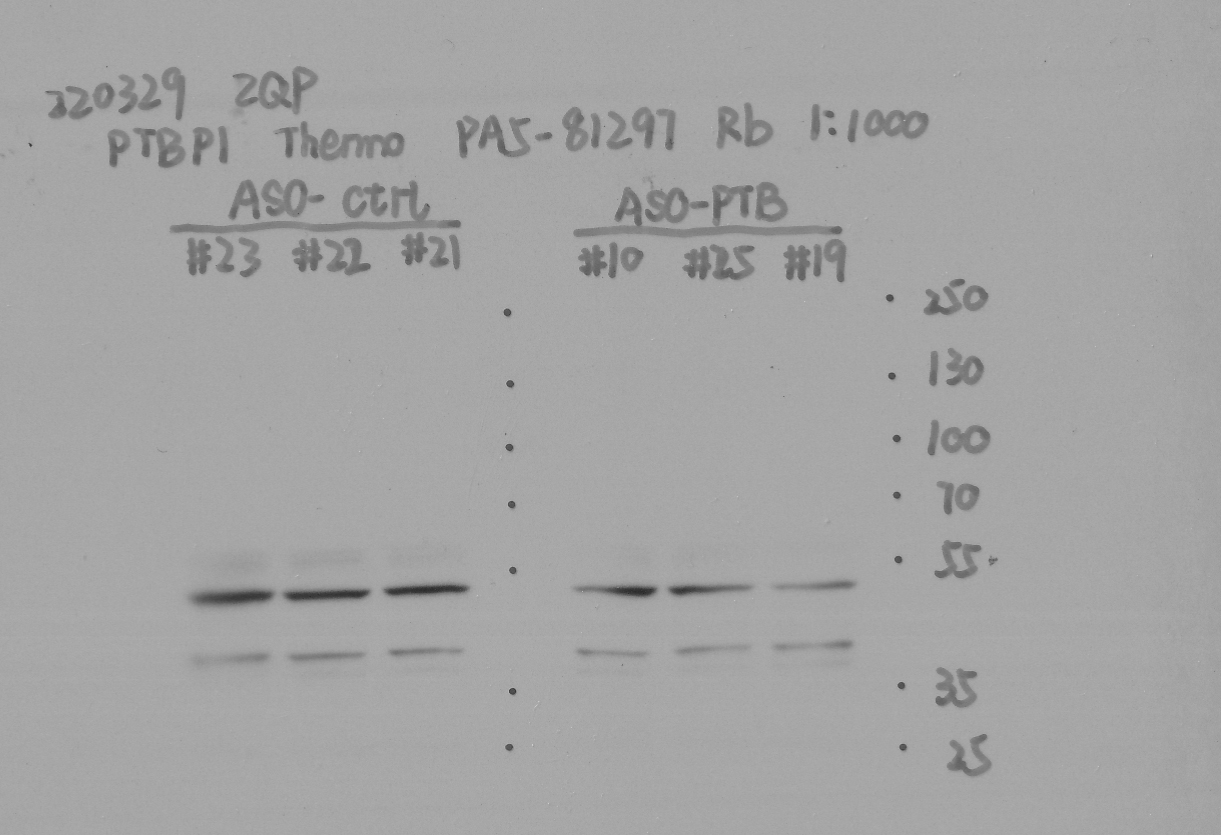

Supplement: Figure 4—figure supplement 1—source data 1. [file elife-75636-fig4-figsupp1-data1.zip › Fig4 source data 4 for Fig4 supplement 1/ASO PTBP1 WB.jpg]
