# Supplementary material for: Repressing PTBP1 fails to convert reactive astrocytes to dopaminergic neurons in a 6-hydroxydopamine mouse model of Parkinson’s disease
Source: eLife. 2022 May 10;11:e75636. doi: 10.7554/eLife.75636 (PMC9208759; doi:10.7554/eLife.75636)
Supplement: Figure 4—source data 2. [file elife-75636-fig4-data2.zip › Fig4 source data 2 for Fig4 D/description of source data for Fig4D.docx]

Brain slices of 6-OHDA lesioned *Aldh1l1-CreER^T2^*:*Rpl22^HA/HA^* mice after ASO-*Ptbp1* delivery in substantia nigra, co-stained with HA (green) and NeuN (red) or TH (purple).
