# Supplementary material for: Repressing PTBP1 fails to convert reactive astrocytes to dopaminergic neurons in a 6-hydroxydopamine mouse model of Parkinson’s disease
Source: eLife. 2022 May 10;11:e75636. doi: 10.7554/eLife.75636 (PMC9208759; doi:10.7554/eLife.75636)
Supplement: Figure 4—figure supplement 1—source data 1. [file elife-75636-fig4-figsupp1-data1.zip › Fig4 source data 4 for Fig4 supplement 1/description of source data for Fig4 supplement 1.docx]

Brain slices of *Aldh1l1-CreER^T2^*:*Rosa-YFP* mice co-stained with PTBP1 (purple), YFP (green) and Cy3 (red) after ASO-*Ptbp1* or ASO-Ctrl delivery in the substantia nigra.
